# Approach to Heterospirocycles for Medicinal Chemistry

**DOI:** 10.1021/acs.orglett.5c03125

**Published:** 2025-09-05

**Authors:** Carlos Rodríguez-Arias, Rubén Miguélez, Yuliia Holota, Pavel K. Mykhailiuk, Pablo Barrio

**Affiliations:** ‡ Departamento de Química Orgánica e Inorgánica and Instituto Universitario de Química Organometálica “Enrique Moles”, 16763 Universidad de Oviedo, Julian Clavería 8, 33006 Oviedo, Spain; § Enamine, Limited, Chervonotkatska 78, 02094 Kyiv, Ukraine; ∥ Chemistry Department, Taras Shevchenko National University of Kyiv, Volodymyrska 64, 01601 Kyiv, Ukraine

## Abstract

The gold­(I)-catalyzed cycloisomerization of aliphatic 1-bromoalkynes has been applied to the synthesis of heterospirocycles. The reactivity of the C­(sp^2^)–Br bond in the products allowed for further derivatization of the obtained scaffolds. In this way, spiroheterocycles decorated with a plethora of functional groups (i.e., CO_2_H, NH_2_, OH, and Bpin) were readily obtained. These spirocyclic compounds are valuable building blocks for modern drug discovery projects.

## Graphical abstract

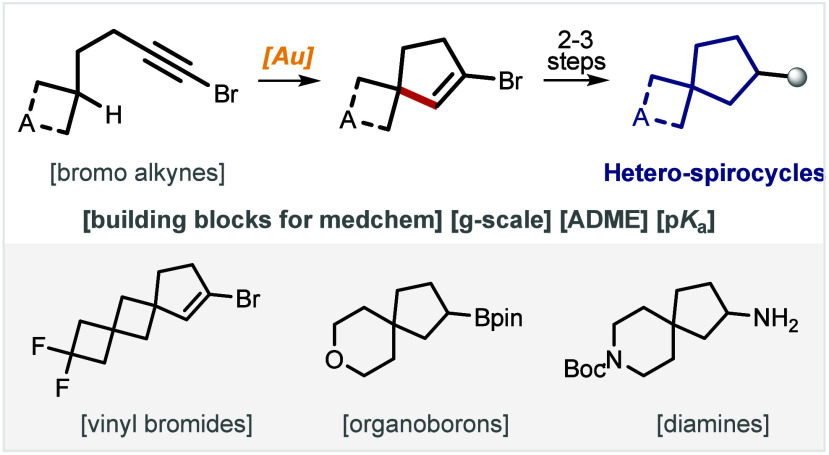

Heteroatom-containing spirocycles, particularly those incorporating atoms, such as nitrogen and oxygen, have played a pivotal role in the advancement of modern drug discovery.^1^ These structural motifs are not only prevalent in biologically active molecules but also key components in a wide range of clinically approved pharmaceuticals (Figure 1). Their unique chemical and pharmacokinetic properties often translate into enhanced binding affinity, selectivity, and metabolic stability, making them valuable scaffolds in medicinal chemistry. Given their significance, the development of new and efficient synthetic methodologies to access such frameworks remains a high priority. In particular, oxa- and aza-spirocyclic compounds, spirocycles containing oxygen or nitrogen atoms, have attracted increasing attention due to their rigid three-dimensional architectures and potential to improve drug-like characteristics.^2,3^ In this context, we present a novel and practical approach for the synthesis of oxa/aza-spirocycles via a gold­(I)-catalyzed^4^ cycloisomerization of aliphatic 1-bromoalkynes.^5,6^ This strategy provides a straightforward and efficient route to access structurally diverse spirocyclic systems under mild conditions, expanding the synthetic toolbox available for constructing these valuable motifs.

**Figure 1:**
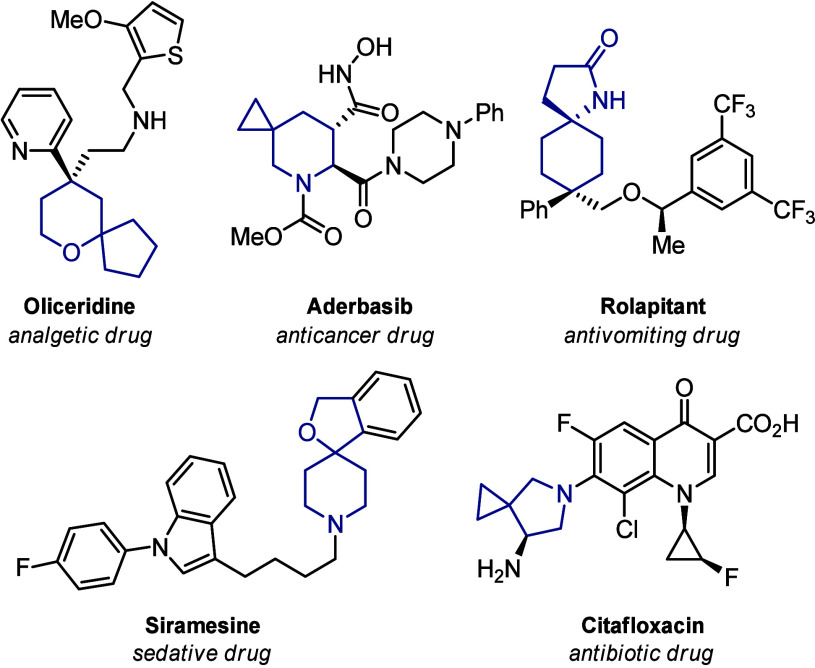
Heteroatom-substituted spirocycles in drugs.

Recently, we disclosed a new gold­(I)-catalyzed cycloisomerization reaction of aliphatic 1-bromoalkynes that entails the functionalization of an unbiased C­(sp^3^)–H bond.^7–9^ The reaction showed remarkable versatility in terms of scaffold accessibility: in addition to simple cyclopentene derivatives, bridged, fused, and spirocyclic bicyclic frameworks were also obtained by introducing carbocycles into starting materials (Scheme 1A). However, the main limitation was a narrow functional group tolerance. In a subsequent study, we determined that at least seven carbon atoms should separate the functional group and the bromine atom in a linear substrate for the reaction to proceed (Schemes 1B and 2).^10^ In this work, we disclose that a limited number of specific heterocyclic substrates can also participate in this reaction, affording value-added heterospirocyclic building blocks for medicinal chemistry (Scheme 1C).

**Scheme 1:**
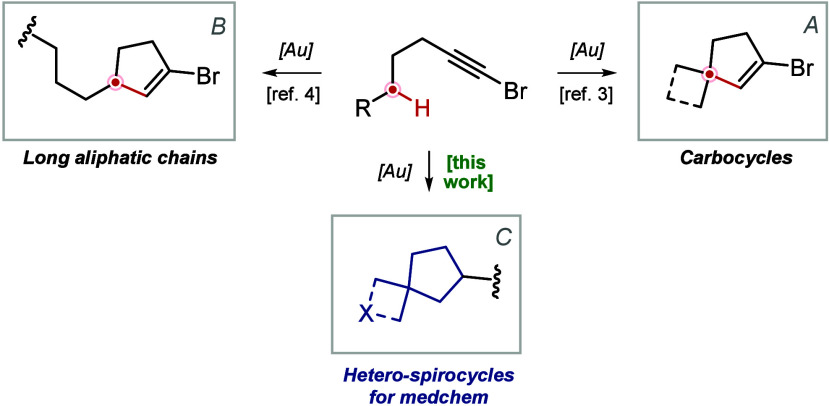
Previous Results and This Work

**Scheme 2:**
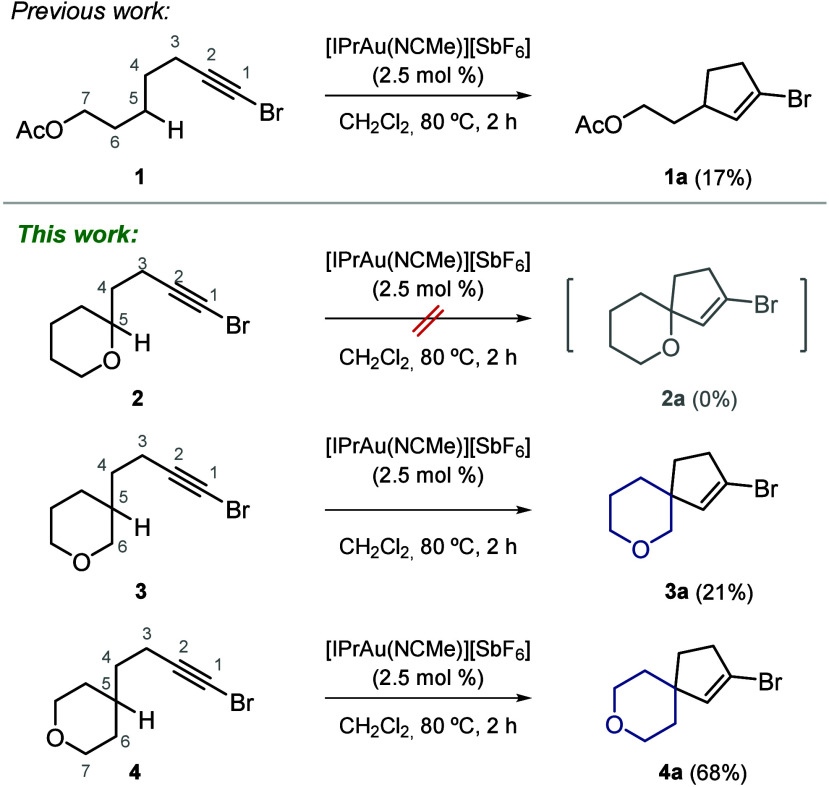
Preliminary Results on Pyrane Derivatives

## First Results

Previously, we demonstrated that compound **1** participated in a gold­(I)-catalyzed cycloisomerization reaction to afford vinyl bromide **1a**, albeit with a low yield of 17% (see Scheme 2).^9^ Motivated by this observation, we hypothesized that cyclic oxygen-containing substrates might similarly undergo a cycloisomerization process. However, compound **2** was recovered unchanged under the reaction conditions. Conversely, compound **3** yielded a low but encouraging 21% yield of the desired product (see Scheme 2). This outcome with the cyclic substrate corroborates our initial findings with linear analogues, indicating that only substrates bearing a heteroatom sufficiently separated from the bromine atom are amenable to the cycloisomerization process. However, within this cyclic series, a trend emerged whereby reactivity appeared to be less influenced by proximity to the reactive center.

While in the linear series the heteroatom must be positioned at least at position 7, we observed comparable yields when the heteroatom was located at position 6 when embedded in a heterocycle. We attribute this shift to conformational constraints imposed by the cyclic structure of the substrate. The most significant result was obtained with isomeric bromoalkyne **4**, which produced targeted spirocyclic product **4a** in a substantially higher yield of 68% (see Scheme 2). Once again, the increased yield showcases the above-mentioned distance-dependent tolerance of heteroatoms.

## Scalability

Having prepared interesting spirocyclic compound **4a** in milligram quantities, we decided to check if this protocol was scalable. Indeed, under the standard conditions with no further optimization, product **4a** was also easily obtained in a 1.1 g quantity in just one run (Scheme 3). The yield of the synthesis (81%) was even higher than that obtained before on a milligram scale (68%).

**Scheme 3:**
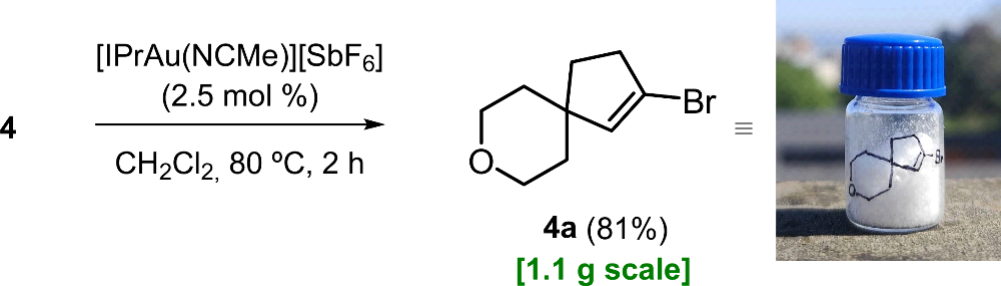
Gram-Scale Synthesis of Compound **4a**

## Scope

These preliminary results set the stage for the study of the scope and limitations of this strategy; only spirocyclizations at remote locations will be considered. First, in addition to pyrane **4a**, we assayed six-membered carbocycles bearing other oxygen-containing functional groups, such as ketal **5a** or ketone **6a**. After showing the compatibility of several oxygenated functionalities, we turned our attention to *N*-heterocycles. In this case, an additional variable needed to be considered, the protective group on nitrogen. After several of them were screened (**7**–**9**), the best result was obtained with trifluoroacetamide **7a**. We then turned to six-membered rings bearing other pharmacophores in position 4, such as difluoro **10a**, sulfone **11a**, or *gem*-dimethyl **12a**. While difluoromethylene and *gem*-dimethyl units worked in moderate to high yields (**10a** and **12a**; Scheme 4), sulfone derivative **11a** was obtained in only a poor 13% yield. To test our hypothesis that the relative position between bromoalkyne and the heteroatom is the key factor that determines the reactivity, we decided to increase the ring size from six to seven. Oxepane derivative **13a** was obtained in a moderate yield (32%). Satisfyingly, the corresponding seven-membered lactone afforded product **14a** in an improved yield of 76% yield. We then envisioned that using spiro[3.3]­heptane-derived bromoalkynes might lead to similar results, since the relative position of bromoalkyne and the polar group would be maintained. This feature is behind the use of such scaffolds as bioisosteres of six-membered heterocycles. 2,2-Difluorospiro[3.3]­heptane **15** afforded the desired product **15a** in an improved yield (74%) compared to the six-membered analogue **10a** (50%) (Scheme 4).^200^ The introduction of a carbocycle in the tether between the bromoalkyne unit and the spirocycle allowed us to afford bis-spirocyclic compound **16a** in a moderate yield (51%). In this case, a slight modification in the reaction conditions (60 °C and 12 h instead of 80 °C and 2 h) was required to minimize the formation of an undesired byproduct. Finally, several three-substituted cycles were used to preliminarily assess the generality of the trend observed with the pyrane ring (see Scheme 2). To our delight, azepane, ketone, and *gem*-difluoro derivatives **17a**–**19a** were obtained in moderate to good yields. These results reinforce the above-mentioned rationale that heteroatom base functional groups are tolerated at closer positions in cyclic systems.

**Scheme 4:**
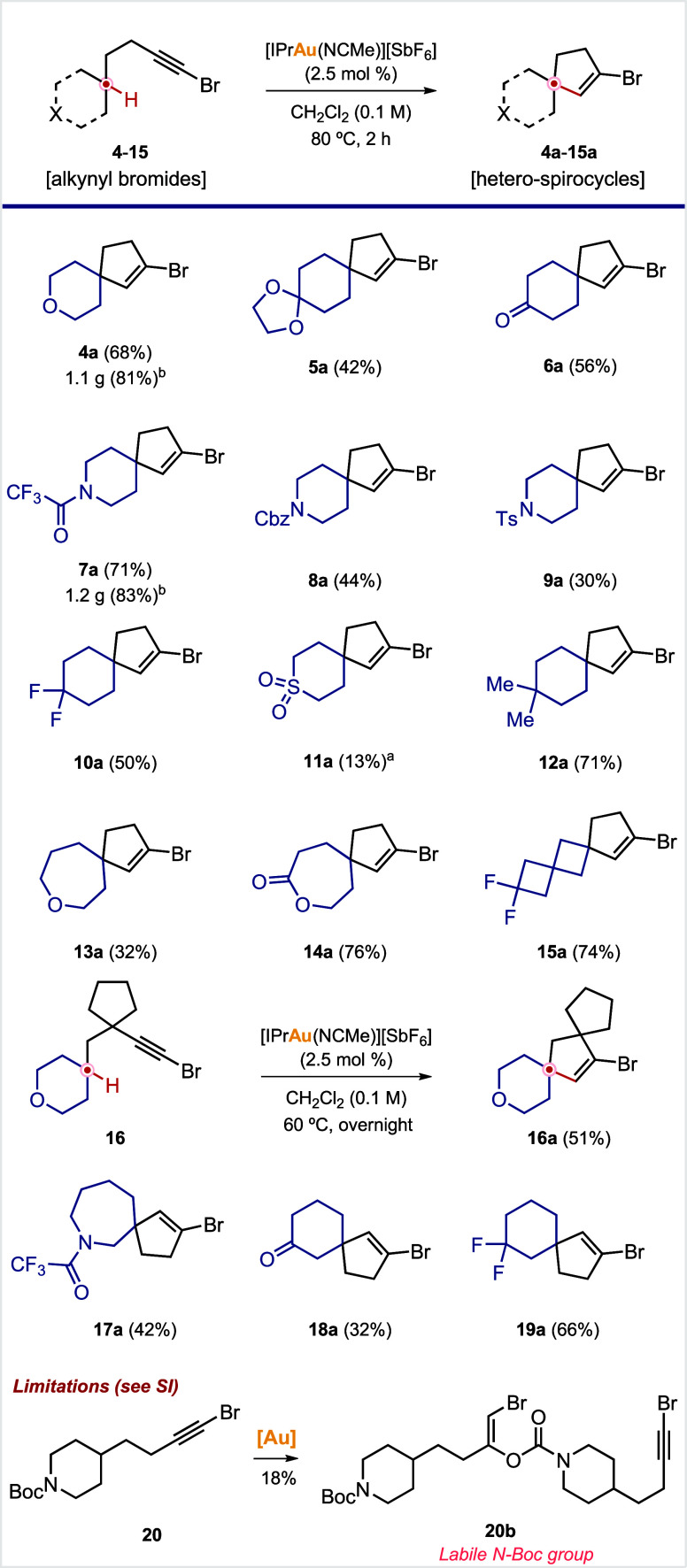
Scope and Limitations

a Not isolated.

## Limitations

We have encountered three main limitations (Scheme 4, bottom, and the Supporting Information). First, the *N*-Boc group proved to be unsuitable. The intermolecular nucleophilic addition product **20b** was the only identifiable product obtained, although in a poor 18% yield.^11^ Then, as mentioned above, the proximity of the heteroatom to the reactive center precludes the reactivity of smaller rings (azetidine **38a** and tetrahydropyrane **41a**; see the Supporting Information) or substrates bearing a heteroatom in the tether (**39a**; see the Supporting Information). Following the same reasoning as above, we aimed to use a 2-oxaspiro[3.3]­heptane-derived substrate as an isostere of the pyrane ring. Unfortunately, the corresponding derivative **40a** was not obtained because the starting material experienced decomposition under the reaction conditions (Supporting Information).

## Modifications

Once the scope and limitations of the gold-catalyzed spirocyclization had been delineated, we proceeded to investigate the reactivity of the versatile C­(sp^2^)–Br bond present in our synthesized products (Scheme 5). Among the many possible reactions, we selected those leading to derivatives bearing functional groups commonly used in drug discovery, such as carboxylic acid, amine, ketone, or boronic ester (Scheme 5). For this derivatization study, we have only used O and N derivatives **4a**, **7a**, and **20a**. Carboxylation followed by catalytic hydrogenation afforded carboxylic acid derivatives **21a**/**21b** (Scheme 5). The versatility of the carboxylic group allowed us to afford the corresponding alcohol **22a** and two different classes of amine derivatives **23a**/**23b** and **24a**/**24b**, either by reduction of amide or by Curtius rearrangement. Homologated carboxylic acid **25a** could also be obtained under Arndt–Eistert conditions. Finally, Miyaura borylation followed by catalytic hydrogenation afforded boronate ester derivative **27a** (Scheme 5). Oxidation of the C–B bond allowed us to access ketones **28a**/**28b** from which trifluoromethyl alcohol **29a** was readily synthesized.

**Scheme 5:**
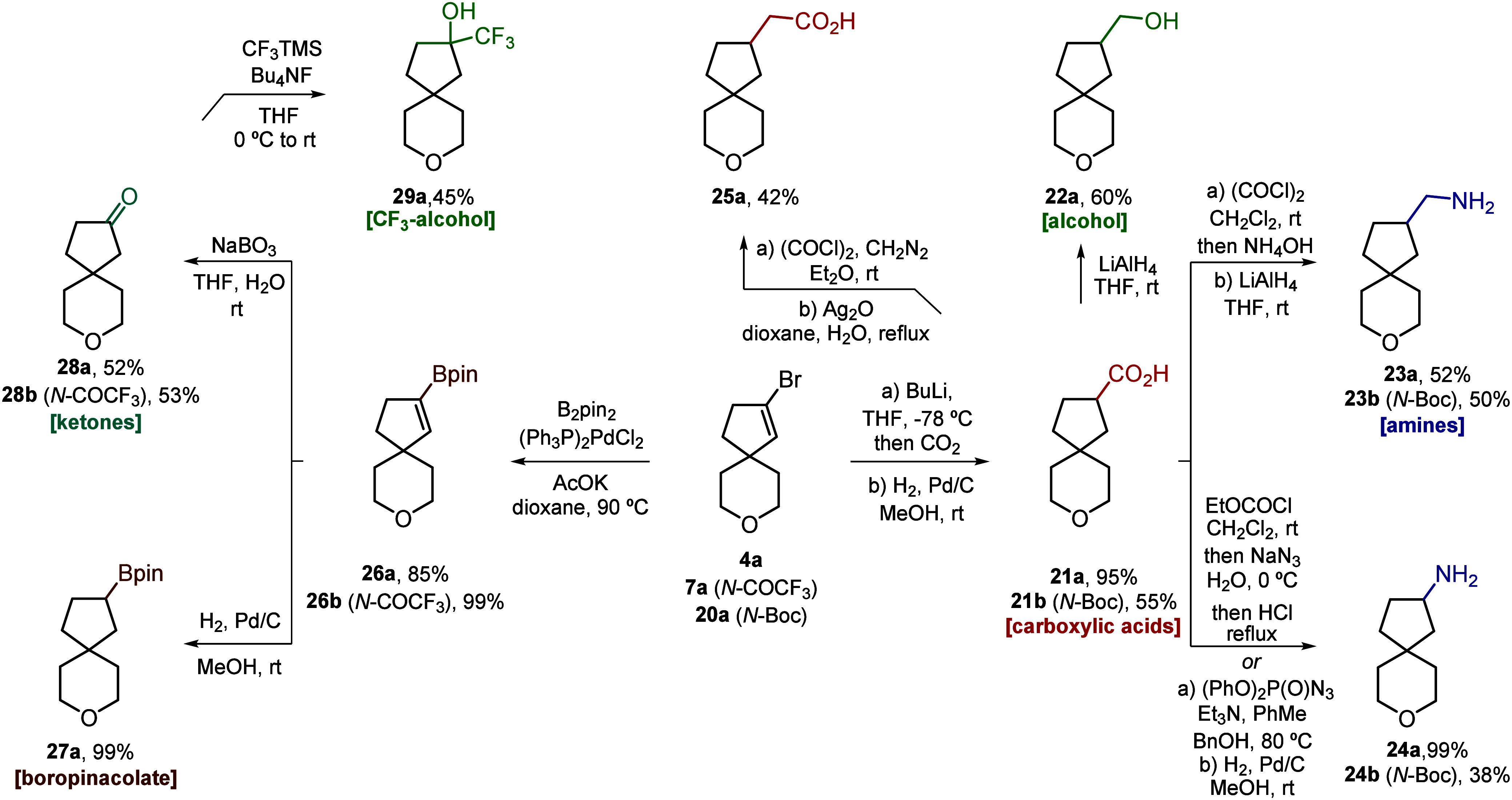
Synthesis of Heteroatom-Substituted Spirocyclic Building Blocks for Medicinal Chemistry

## Acidity of Functional Groups

We also examined the effect of substituting the pyrane ring with its spirocyclic analogue on the electronic properties. To this end, we experimentally determined the p*K*_a_ values of carboxylic acids **21a** and **28** as well as amine hydrochlorides **24a** and **29** (see Figure 2). Pyrane-containing carboxylic acid **28** was 0.5 p*K*_a_ units more acidic than spirocyclic carboxylic acid **21a**: p*K*_a_ = 4.2 (**28**) vs 4.7 (**21a**). A similar trend was observed with amines. Pyrane-containing amine hydrochloride **29** was 0.8 p*K*_a_ more acidic than the spirocyclic amine **24a**: p*K*_a_ = 9.7 (**29**·HCl) vs 10.5 (**21a**·HCl).

**Figure 2:**
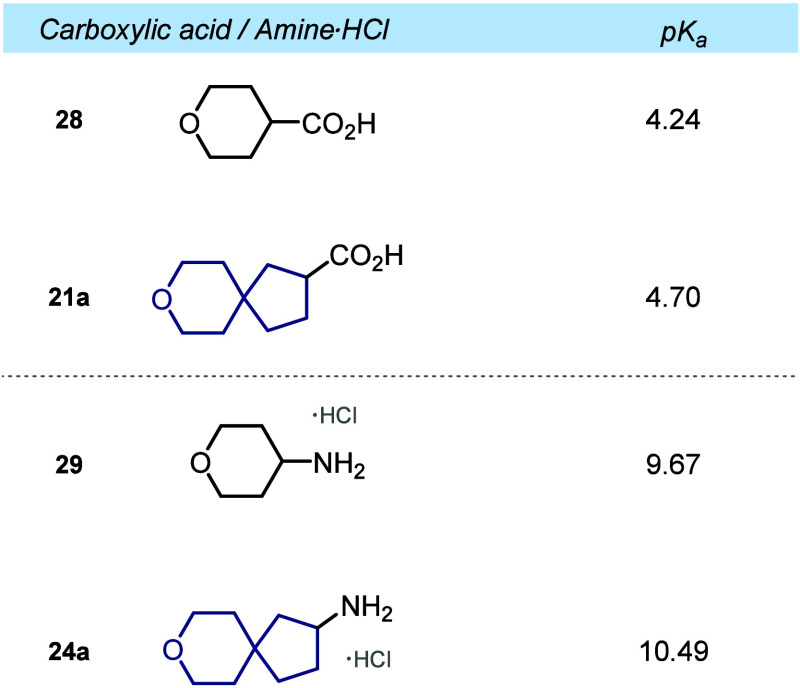
Experimental p*K*_a_ values of carboxylic acids **21a** and **28** and amine hydrochlorides **24a** and **29**.

## Physicochemical and Pharmacokinetic Properties

Finally, we wanted to understand the impact of the monocycle-to-spirocycle replacement on the experimental physicochemical and pharmacokinetic properties of organic compounds: water solubility, lipophilicity, and metabolic stability. For that, we synthesized and studied four model amides **30**–**33**.

An effect of the pyrane to spirocyclic analogue replacement on the experimental water solubility (sol.) and metabolic stability (*CL*_int_) was not observed due to the too high solubility and too high stability of all four compounds, outside of the sensitivity range of the experimental method (Figure 3).

**Figure 3:**
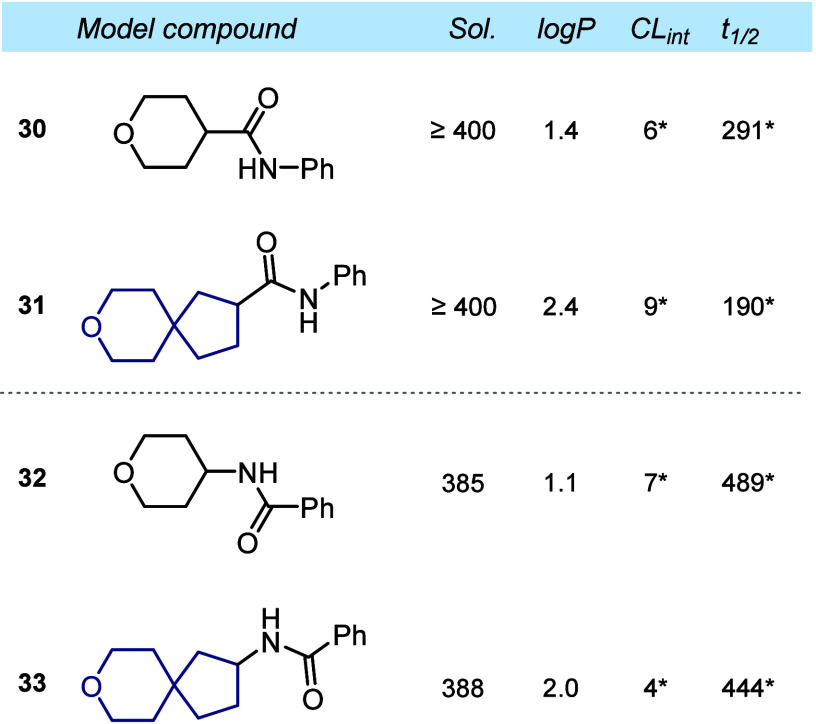
Physicochemical and pharmacokinetic properties of compounds **30** and **32** and their spirocyclic analogues **31** and **33**. Solubility, experimental kinetic solubility in phosphate-buffered saline at pH 7.4 (μM); log *P*, experimental distribution coefficient in *n*-octanol/water, with reliable log *P* values obtained within a range of 1.0–4.0; *CL*_int_, experimental metabolic stability in human liver microsomes (μL min^–1^ mg^–1^); and *t*_1/2_, experimental half-time of a metabolic decomposition (min). (∗) The parameter should be considered as approximate due to the high stability of compounds.

Replacement of the pyrane ring with the larger spirocyclic analogue increased lipophilicity by ca. 1 log *P* unit: 1.4 (**30**) vs 2.4 (**31**) and 1.1 (**32**) vs 2.0 (**33**) (Figure 3).

In conclusion, here, we have developed a novel practical approach toward heterospirocycles via the gold­(I)-catalyzed cycloisomerization of unique heterocyclic 1-bromoalkynes. Cyclic substrates show better tolerance to a heteroatom in proximity than linear ones. The presence of a readily functionalizable C­(sp^2^)–Br bond in the products has been leveraged to introduce an array of common functional groups: CO_2_H, NH_2_, OH, and Bpin. The obtained heterospirocyclic derivatives are valuable building blocks for drug discovery projects. Further developments aimed at finding more tolerant reaction conditions that allow expansion of the range of heterospirocycles affordable with this methodology are currently under study.

## Supplementary Material



## Data Availability

The data underlying this study are available in the published article and its Supporting Information.

## Notes

Experimental procedures and characterization of all new compounds, copies of ^1^H, ^13^C, and ^19^F NMR spectra, titration curves for the determination of p*K*_a_ values, and assays for the determination of ADME properties (solubility, log *P*, *CL*_int_, and *t*_1/2_) (PDF)

## Notes

Dedicated to the memory of our colleague Prof. Ivan Lavandera.
