# Supplementary material for: Approach to Heterospirocycles for Medicinal Chemistry
Source: Org Lett. 2025 Sep 5;27(37):10342–7. doi: 10.1021/acs.orglett.5c03125 (PMC12455649; doi:10.1021/acs.orglett.5c03125)

Supporting Information

An Approach to Hetero-Spirocycles for Medicinal Chemistry

Carlos Rodríguez-Arias, Rubén Miguélez,[a,‡] Yuliia Holota,[b] Pavel K. Mykhailiuk*[b] and Pablo Barrio*[a]

Correspondence to: barriopablo@uniovi.es

pavel.mykhailiuk@gmail.com

This PDF file includes:

Materials and Methods

Experimental procedures (synthesis and determination of pK_a and ADME properties)

Characterization of new compounds

Copy of ^{1}H, ^{13}C and ^{19}F NMR spectra

Table of Contents

1. Equipment and Instruments...S3

2. *Methods*..S4

3. *Chemicals*..S4

4. *Synthesis procedures and characterizations*...S5

4.1. *Synthesis of 1-bromoalkynes*………………………………………………………...S7

4.2. *Gold-catalyzed reactions* ……………………..……………………….……………S30

4.3. *Derivatizations*……………………………………………………………………..……..…S37

4.4. *Limitations*………………………………………………………………………………….*S55*

5. *Determination of pK_a values*...S56

6. *Determination of Aqueous Solubility*……………………………………………….S60

7. *Determination of Distribution Coefficient (LogP)*…...S65

9. *Assessment of Metabolic Stability in Human Liver Microsomes* …....................S68

9. *Interpretation of microsomal stability assay data*……......................................S73

10. NMR spectra

10.1 Bromoalkynes and intermediates…………………………………………………S74

10.2 Gold(I)-catalyzed reactions……..……………………………………………….…….S133

10.3 Derivatizations……..………………………………………………….……………………S156

10.4 Bromoalkynes and intermediates for failed reactions……………………………S188

1. *Equipment and Instruments*

Nuclear Magnetic Resonance (NMR) spectroscopy:

NMR spectra were recorded on a Bruker AV 600 spectrometer operating at 600.15 (^{1}H), 150.91 MHz (^{13}C), using a 5 mm PATXI ^{1}H/D-^{13}C/^{15}N inverse probe with a z-gradient coil, or on a Bruker AV 400 spectrometer operating at 400.54 (^{1}H) and 100.72 MHz (^{13}C), using a 5 mm PABBI 1H/D-BB inverse probe with a z-gradient coil, or on a Bruker AV 300 spectrometer operating at 300.13 (^{1}H), 75.46 MHz (^{13}C), using a 5 mm QNP ^{1}H/^{13}C/^{19}F/^{31}P/D probe with a z-gradient coil and equipped with an automatic sample changer. The NMR samples were prepared in $CDCl_3$ and measured at 298K (unless otherwise stated). Data are reported as follows: chemical shift (δ) in parts per million (ppm) relative to tetramethylsilane (TMS), multiplicity (s: singlet, d: doublet, t: triplet, q: quartet, sep: septet, non: nonet, dd: double doublet, dt: double triplet, m: multiplet), coupling constants (J) in Hertz (Hz) and integration. ^{13}C multiplicities were assigned by DEPT experiments. The residual solvent signals of deuterated solvents were used as internal references. All the experiments were acquired with the TOPSPIN 2.1 Bruker NMR software and the spectra analysis was conducted via the NMR processing softwares TOPSPIN 2.1 or MestReNova v.14.2.1-27684.

Mass Spectrometry (MS):

High resolution mass spectra (HRMS) were measured on a high-resolution mass spectrometer IMPACT II, BRUKER (Servicios Ciefntifico Técnicos, Universidad de Oviedo) with a quadrupole and a Time-Of-Flight (TOF) tube as analyzer, using conventional Electrospray Ion Source (ESI), in full scan mode (4 eV) and positive ion polarity. The equipment employes N_2 at the nebulization step (2.4 Bar), and as drying gas (250 ºC, 6.0 L/min). Alternatively, and due to the low polarity of these compounds, some HRMS spectra were determined on an Agilent equipment using APCI(+) ionization (6545 Q-TOF, AGILENT, MS Spectrometer, Servicios Cientifico Técnicos). In some cases, HRMAS could not be obtained, since the molecules failed to ionize under any of these conditions.

2. *Methods*

All reactions discussed as results of this work were carried out using oven dried glassware under an atmosphere of argon (99.999%) using standard Schlenk techniques. Glassware was evacuated and further dried by heating with a heat-gun. Electric heating-stirring plates with either oil baths were used for reactions at elevated temperatures. For reactions below room temperature, the reaction vessel was cooled using a JULABO FT902-Cryostat. Reaction temperatures refer to the external bath temperature. Cannulas and syringes were used for the transfer of reagents and solvents, which were flooded with inert gas (3×) before use. Purification by column chromatography was performed using manual air pressure on Geduran© Si60 silica gel (40-63 µm) from Merck KGaA. Silica gel F254 TLC plates from Merck KGaA were used for monitoring reactions, analyzing fractions of column chromatography, and measuring Rf values. To visualize the analytes, TLC plates were treated with appropriate staining solutions followed by subsequent heating.

3. *Chemicals*

Commercial reagents were purchased with the best quality affordable from Sigma Aldrich, TCI, Alfa Aesar and Acros Organics. Solvents purchased in technical grade quality were distilled under reduced pressure and used for purification procedures. and used without further purification unless otherwise stated.1,2-dichloroethane was distilled from CaH_2 and THF from sodium/benzophenone. Other anhydrous solvents were purchased from commercial sources. TLC was performed on aluminum-backed plates coated with silica gel 60, with F245 indicator, and developed with phosphomolybdic acid or potassium permanganate stains. Solvents used in column chromatography were obtained from commercial suppliers and used without further purification.

4. *Experimental procedures*

General Procedure A1: *Synthesis of alcohols*

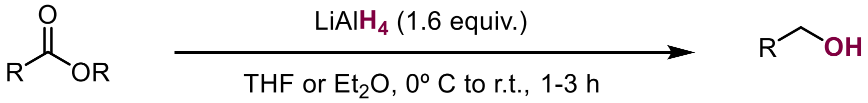

At 0 ºC, a solution of the corresponding carboxylic acid in dry THF (1.00 M) was added dropwise to a suspension of $LiAlH_4$ (1.6 equiv.) in dry THF (0.40 M). After the addition was complete the reaction mixture was stirred for 1-3 hours at room temperature. Then, the reaction was carefully quenched by dropwise addition of water (1.0 mL per gram of LAH), NaOH 15% (1.0 mL per gram of LAH) and water (3.0 mL per gram of LAH) at 0 ºC. Solids were filtered through alternate pads of celite /silica gel/ celite and by the removal of the solvents by rotary evaporation the crude alcohol was afforded and used in the next step without further purification.

General Procedure A2: *Synthesis of alcohols*

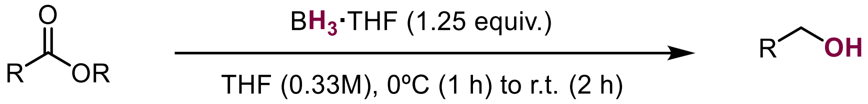

At 0 ºC, a solution of borane in THF (1.00 M) was added dropwise to a solution of the corresponding carboxylic acid (1.0 equiv.) in dry THF (0.33 M). After the addition was complete the reaction mixture was stirred for 1 hour at 0ºC. Then, the reaction was taken to room temperature and stirred for 2 additional hours. After that, the reaction was quenched by dropwise addition of H_2O:AcOH (1: 1, 1.0 mL per mL of borane solution), THF was evaporated and the reaction mixture was extracted with DCM (x3), then washed with $NaHCO_3$ sat. and brine. The organic layer was dried over Na_2SO_4 and the solvent was removed under reduced pressure affording the crude alcohol that was used in the next step without further purification.

General Procedure B1: *Synthesis of 1-bromoalkanes*

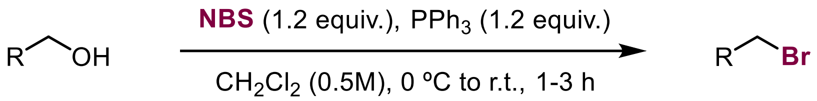

In a Schlenk flask under argon, PPh_3 (1.2 equiv.) was added to a solution of the corresponding alcohol in anhydrous DCM (0.50 M). The mixture was taken to 0 ºC and NBS (1.2 equiv.) was added portion wise. Then, the reaction was taken to room temperature and monitored by TLC and upon finishing (1-3 hours) the solvent was removed under vacuum. The obtained crude mixture was purified by flash column chromatography on silica gel using hexane/EtOAc mixtures as eluent to afford the corresponding primary bromide.

General Procedure B2: *Synthesis of 1-bromoalkanes*

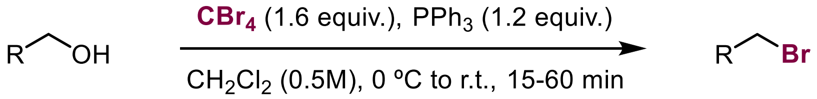

In a Schlenk flask under argon, CBr_4 (1.6 equiv.) was added to a solution of the corresponding alcohol in anhydrous DCM (0.50 M). The mixture was taken to 0 ºC and PPh_3 (1.2 equiv.) was added portion wise. Then, the reaction was taken to room temperature and monitored by TLC and upon finishing (15-60 minutes) the solvent was removed under vacuum. The obtained crude mixture was purified by flash column chromatography on silica gel using hexane/EtOAc mixtures as eluent to afford the corresponding primary bromide.

General Procedure C: *Synthesis of 1-alkynes*

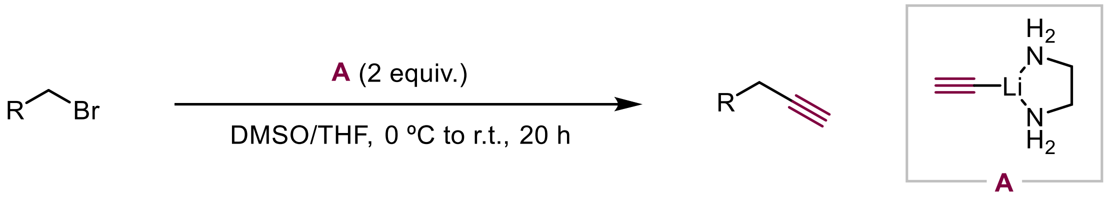

Lithium acetylide ethylenediamine complex **A** (2.0 equiv.) was weighed in a flame dried Schlenk flask under argon.Anhydrous DMSO (1.0 mL per mmol of B) and THF (0.5 mL per mmol of B) were added, and the reaction mixture was taken to 0 ºC. A solution of the corresponding primary bromide in THF (0.5 mL per mmol) was added at once. The reaction mixture was then stirred overnight at room temperature. The day after, the reaction was quenched with saturated NH_4Cl solution, extracted with Et_2O (x3) and washed with brine (x5). The organic layer was dried over Na_2SO_4 and the solvent was removed under reduced pressure. The obtained crude mixture was purified by flash column chromatography on silica gel using hexane/EtOAc mixtures as eluent to afford the corresponding alkynes.

General Procedure D: *Synthesis of 1-bromoalkynes*

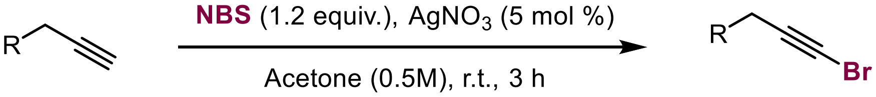

In a Schlenk flask under Ar, NBS (1.2 equiv.) was added to a solution of terminal alkyne (typically 1 mmol) in acetone (0.50 M). Then, $AgNO_3$ (5 mol %) was added and the reaction mixture was stirred for 3h in the dark. Acetone was removed in a rotatory

evaporator and the crude reaction mixture purified by means of flash column chromatography using hexane/EtOAc mixtures as eluent to afford the corresponding bromoalkynes.

General Procedure E: *Cycloisomerization of 1-bromoalkynes*

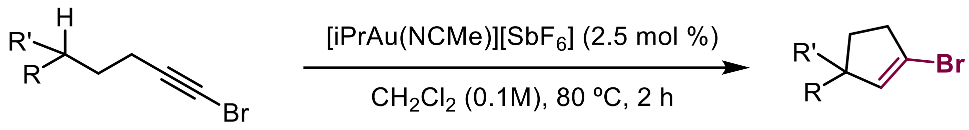

In a flame-dried 5 mL Young tube under Ar, [IPrAu(NCMe)][SbF_6] (2.5 mol%) was added and dissolved in the minimum amount of dry DCM . Then, the 1-bromoalkyne (1.0 equiv.) was added. Finally, the remaining dry DCM (0.10 M) was added and the reaction mixture was stirred at 80 °C for 2 hours. When the reaction time was finished it was cooled down to room temperature. The mixture was concentrated on a rotary evaporator under reduced pressure at 40 °C. The crude was purified by flash chromatography using hexane/EtOAc mixtures as eluent to afford the corresponding products. For one gram scale, the same protocol using a 25 mL Young tube was followed.

4.1. Synthesis of 1-bromoalkynes

2-(tetrahydro-2H-pyran-2-yl)ethan-1-ol (2-OH)

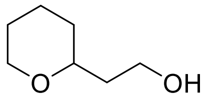

The title compound was synthesized using General Procedure A1, starting from 4 mmol (577 mg) of the proper carboxylic acid, yielding the crude alcohol (***2- OH***) as a colorless liquid (465 mg, 4.00 mmol, 100%). This compound was used without further purification. **^{1}H NMR** (300 MHz, $CDCl_3$) δ 3.96 (dt, *J* = 11.4, 2.3 Hz, 1H), 3.76 (t, *J* = 5.5 Hz, 2H), 3.59 – 3.36 (m, 2H), 2.69 – 2.55 (m, 1H), 1.81 (dt, *J* = 10.4, 3.1 Hz, 1H), 1.77 – 1.63 (m, 2H), 1.60 – 1.48 (m, 4H), 1.43 – 1.28 (m, 1H). **^{13}C NMR** (75 MHz, $CDCl_3$) δ 78.0, 68.4, 61.0, 38.4, 32.0, 25.9, 23.3. **HRMS**: non purified.

2-(2-bromoethyl)tetrahydro-2H-pyran (2-Br)

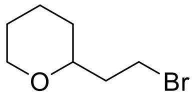

The title compound was synthesized using General Procedure B1, starting from 4 mmol (465 mg) of the corresponding alcohol (***2-OH***), yielding the bromoalkane (***2-Br***) as a colorless liquid (450 mg, 2.30 mmol, 58%). The crude was purified by flash column chromatography using hexane/EtOAc (10:1) as eluent.
^{1}H NMR (300 MHz, $CDCl_3$) δ 3.98 – 3.87 (m, 1H), 3.57 – 3.32 (m, 4H), 2.05 – 1.74 (m, 3H), 1.51 (dtt, *J* = 9.6, 7.7, 3.0 Hz, 4H), 1.35 – 1.16 (m, 1H). **^{13}C NMR** (75 MHz, $CDCl_3$) δ 75.2, 68.3, 39.5, 31.6, 30.1, 26.0, 23.3. **HRMS**: unstable.

2-(but-3-yn-1-yl)tetrahydro-2H-pyran (2-CCH)

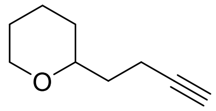

The title compound was synthesized using General Procedure C, starting from 2.3 mmol (450 mg) of the bromoalkane (***2-Br***), yielding the alkyne (***2-CCH***) as a colorless liquid (41 mg, 0.30 mmol, 13%). The crude was purified by flash column chromatography using hexane/EtOAc (10:1) as eluent.

^{1}H NMR (300 MHz, $CDCl_3$) δ 3.94 (dq, *J* = 11.6, 2.2 Hz, 1H), 3.45 – 3.31 (m, 2H), 2.27 (td, *J* = 7.2, 2.6 Hz, 2H), 1.91 (t, *J* = 2.7 Hz, 1H), 1.85 – 1.75 (m, 1H), 1.72 – 1.44 (m, 6H), 1.33 – 1.14 (m, 1H). **^{13}C NMR** (75 MHz, $CDCl_3$) δ 84.4, 76.1, 68.4, 68.0, 35.2, 31.7, 26.1, 23.4, 14.6. **HRMS**: non-ionizable.

2-(4-bromobut-3-yn-1-yl)tetrahydro-2H-pyran (2)

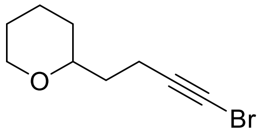

The title compound was synthesized using General Procedure D, starting from 0.30 mmol (41 mg) of 2-(but-3-yn-1-yl)tetrahydro-2H-pyran (**2-*CCH***), yielding the bromoalkyne (**2**) as a colorless liquid (37 mg, 0.17 mmol, 58%). The crude was purified by flash column chromatography using Hexane/EtOAc (10:1) as eluent.

1H NMR (300 MHz, $CDCl_3$) δ 3.94 (dt, J = 12.0, 2.4 Hz, 1H), 3.52 – 3.16 (m, 2H), 2.30 (t, J = 7.2 Hz, 2H), 1.91 – 1.75 (m, 1H), 1.77 – 1.41 (m, 6H), 1.24 (q, J = 5.6, 3.9 Hz, 1H). **^{13}C NMR** (75 MHz, $CDCl_3$) δ 80.1, 76.0, 68.4, 37.5, 35.0, 31.7, 26.1, 23.4, 15.9. **HRMS** (ESI-TOF) m/z: [M]+ Calcdd for C9H13BrO 216.0150; Found 216.0152.

2-(tetrahydro-2H-pyran-3-yl)ethan-1-ol (3-OH)

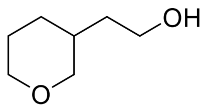

The title compound was synthesized using General Procedure A1, starting from 3.50 mmol (500 mg) of the proper carboxylic acid, yielding the crude alcohol (**3- *OH***) as a colorless liquid (419 mg, 3.20 mmol, 91%). This compound was used without further purification.

^{1}H NMR (300 MHz, $CDCl_3$) δ 3.82 – 3.73 (m, 2H), 3.53 (d, *J* = 6.8 Hz, 2H), 3.29 (dt, *J* = 11.4, 6.9 Hz, 1H), 3.01 (t, *J* = 10.5 Hz, 2H), 1.86 – 1.74 (m, 1H), 1.70 – 1.61 (m, 1H), 1.51 (dt, *J* = 7.4, 3.9 Hz, 2H), 1.31 (tt, *J* = 13.8, 7.2 Hz, 2H), 1.09 (dt, *J* = 16.6, 10.4 Hz, 1H). **^{13}C NMR** (75 MHz, $CDCl_3$) δ 73.1, 68.2, 59.8, 35.1, 32.7, 29.7, 25.5. **HRMS**: non purified

3-(2-bromoethyl)tetrahydro-2H-pyran (3-Br)

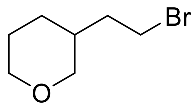

The title compound was synthesized using General Procedure B1, starting from 3.2 mmol (419 mg) of the corresponding alcohol (***3-OH***), yielding the bromoalkane (***3-Br***) as a colorless liquid (476 mg, 2.50 mmol, 78%). The crude was purified by flash column chromatography using hexane/EtOAc (10:1) as eluent.

^{1}H NMR (300 MHz, $CDCl_3$) δ 3.81 (dq, *J* = 10.7, 3.0, 2.3 Hz, 2H), 3.47 – 3.25 (m, 3H), 3.08 (dd, *J* = 11.2, 8.6 Hz, 1H), 1.92 – 1.68 (m, 4H), 1.61 – 1.51 (m, 2H), 1.16 (dddd, *J* = 18.6, 9.2, 6.9, 4.5 Hz, 1H). **^{13}C NMR** (75 MHz, $CDCl_3$) δ 72.4, 68.3, 35.2, 34.5, 30.8, 29.5, 25.2. **HRMS**: unstable.

3-(but-3-yn-1-yl)tetrahydro-2H-pyran (3-CCH)

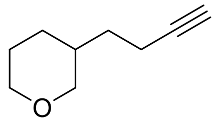

The title compound was synthesized using General Procedure C, starting from 2.5 mmol (476 mg) of the bromoalkane (***3-Br***), yielding the alkyne (***3-CCH***) as a colorless liquid (166 mg, 1.20 mmol, 48%). The crude was purified by flash column chromatography using hexane/EtOAc (10:1) as eluent.

^{1}H NMR (300 MHz, $CDCl_3$) δ 3.84 (dt, *J* = 11.0, 3.0 Hz, 2H), 3.42 – 3.28 (m, 1H), 3.05 (t, *J* = 10.5 Hz, 1H), 2.18 (td, *J* = 7.2, 2.2 Hz, 2H), 1.93 (d, *J* = 2.7 Hz, 1H), 1.89 – 1.82 (m, 1H), 1.72 (ddd, *J* = 10.5, 6.9, 3.5 Hz, 1H), 1.58 (dd, *J* = 7.8, 3.9 Hz, 2H), 1.38 (ddt, *J* = 21.1, 13.6, 6.7 Hz, 2H), 1.12 (dddd, *J* = 15.8, 10.4, 7.5, 5.4 Hz, 1H). **^{13}C NMR** (75 MHz, $CDCl_3$) δ 84.0, 72.8, 68.5, 68.4, 34.9, 31.0, 29.4, 25.5, 15.7. **HRMS** (ESI-TOF) m/z: Calcd. for $[M-H]^+$: 137.0966; Found: 137.0965.

3-(4-bromobut-3-yn-1-yl)tetrahydro-2H-pyran (3)

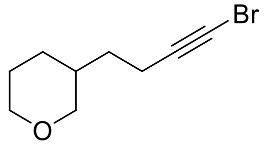

The title compound was synthesized using General Procedure D, starting from 1.1 mmol (166 mg) of the alkyne (***3-CCH***), yielding the bromoalkyne (***3***) as a colorless liquid (190 mg, 0.80 mmol, 70%). The crude was purified by flash column chromatography using hexane/EtOAc (10:1) as eluent.

^{1}H NMR (300 MHz, $CDCl_3$) δ 3.89 – 3.79 (m, 2H), 3.34 (dp, *J* = 11.9, 5.2, 3.6 Hz, 1H), 3.08 – 2.97 (m, 1H), 2.20 (t, *J* = 7.4 Hz, 2H), 1.89 – 1.81 (m, 1H), 1.74 – 1.53 (m, 3H), 1.37 (tq, *J* = 13.6, 6.6, 5.9 Hz, 2H), 1.12 (ddt, *J* = 10.2, 7.6, 3.9 Hz, 1H).**^{13}C NMR** (75

MHz, $CDCl_3$) δ 79.7, 72.8, 68.4, 38.0, 34.9, 30.8, 29.4, 25.5, 17.0. **HRMS** (ESI-TOF) m/z: $[M-H]^+$ Calcd. for $C_9H_{12}Br$ 215.0072; Found: 215.0065.

2-(tetrahydro-2H-pyran-4-yl)ethan-1-ol (4-OH)

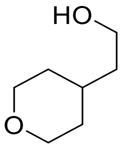

The title compound was synthesized using General Procedure A1, starting from 10 mmol (1.44 g) of the proper carboxylic acid, yielding the crude alcohol (***4-OH***) as a colorless liquid (976 mg, 7.50 mmol, 75%). This compound was used without further purification. **^{1}H NMR** (300 MHz, $CDCl_3$) δ 3.94 (dd, J = 10.0, 4.2 Hz, 2H), 3.69 (q, J = 6.2 Hz, 2H), 3.38 (td, J = 11.8, 2.1 Hz, 2H), 1.67 – 1.41 (m, 6H), 1.30 (qd, J = 11.8, 4.5 Hz, 2H). **^{13}C NMR** (75 MHz, $CDCl_3$) δ 68.0, 60.0, 39.6, 33.0, 31.5. **IR**: 3376, 2929, 2916, 2850, 2242, 1018, 1091, 911, 735, 647. **HRMS** (ESI-TOF) m/z: $[M]^+$Calcd. for $C_7H_{14}O_2$ 130.0993; Found: 130.0994.

4-(2-bromoethyl)tetrahydro-2H-pyran (4-Br)

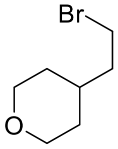

The title compound was synthesized using General Procedure B1, starting from 7.5 mmol (976 mg) of the corresponding alcohol (***4-OH***), yielding the bromoalkane (***4-Br***) as a colorless liquid (1.35 g, 7.00 mmol, 93%). The crude was purified by flash column chromatography using hexane/EtOAc (10:1) as eluent.

^{1}H NMR (300 MHz, $CDCl_3$) δ 3.93 (ddd, J = 11.7, 4.6, 1.7 Hz, 2H), 3.54 – 3.27 (m, 4H), 1.78 (dd, J = 8.5, 4.7 Hz, 3H), 1.59 (dt, J = 13.6, 2.6 Hz, 2H), 1.27 (qd, J = 12.1, 4.4 Hz, 2H). **^{13}C NMR** (75 MHz, $CDCl_3$) δ 67.7, 39.4, 33.2, 32.2, 30.8. **IR**: 2961, 2929, 2843, 2244, 1105, 910, 738, 649. **HRMS**: unstable.

4-(but-3-yn-1-yl)tetrahydro-2H-pyran (4-CCH)

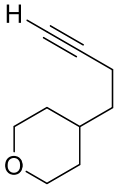

The title compound was synthesized using General Procedure C, starting from 7 mmol (1.35 g) of the bromoalkane (***4-Br***), yielding the alkyne (***4-CCH***) as a colorless liquid (774 mg, 5.60 mmol, 80%). The crude was purified by flash column chromatography using hexane/EtOAc (10:1) as eluent.

^{1}H NMR (300 MHz, $CDCl_3$) δ 3.97 (dd, J = 11.0, 4.0 Hz, 2H), 3.40 (td, J = 11.8, 2.0 Hz, 2H), 2.24 (td, J = 7.3, 2.6 Hz, 2H), 1.96 (t, J = 2.6 Hz, 1H), 1.76 – 1.58 (m, 3H), 1.50 (q,

J = 7.0 Hz, 2H), 1.29 (dddd, J = 17.5, 13.0, 7.9, 3.4 Hz, 2H). **^{13}C NMR** (75 MHz, $CDCl_3$) δ 84.2, 68.3, 67.9, 35.2, 33.7, 32.6, 15.4. **IR**: 3290, 2917, 2840, 1133, 1094, 1013, 640. **HRMS** (ESI-TOF) m/z: $[M-H]^+$Calcd. for $C_9H_{13}O$ 137.0963; Found: 137.0966.

4-(4-bromobut-3-yn-1-yl)tetrahydro-2H-pyran (4)

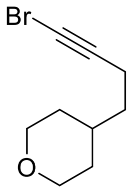

The title compound was synthesized using General Procedure D, starting from 5.6 mmol (774 mg) of the alkyne (***4-CCH***), yielding the bromoalkyne (***4***) as a colorless liquid (961 mg, 4.40 mmol, 79%). The crude was purified by flash column chromatography using hexane/EtOAc (10:1) as eluent.

^{1}H NMR (300 MHz, $CDCl_3$) δ 3.97 – 3.87 (m, 2H), 3.35 (td, J = 11.8, 1.9 Hz, 2H), 2.21 (td, J = 7.3, 1.6 Hz, 2H), 1.57 (d, J = 11.0 Hz, 3H), 1.44 (q, J = 6.9 Hz, 2H), 1.23 (tdd, J = 16.0, 8.1, 3.5 Hz, 2H). **^{13}C NMR** (75 MHz, $CDCl_3$) δ 79.8, 67.8, 37.8, 35.0, 33.0, 32.5, 16.6. **IR**: 3052, 2929, 2845, 1265, 1089, 740, 705. **HRMS** (ESI-TOF) m/z: $[M-H]^+$ Calcd. for $C_9H_{12}BrO$ 216.0150; Found: 216.0136.

8-(2-bromoethyl)-1,4-dioxaspiro[4.5]decane (5-Br)

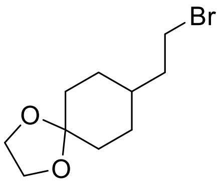

The title compound was synthesized using General Procedure B2, starting from 10 mmol (1.86 g) of the corresponding alcohol, yielding the bromoalkane (***5-Br***) as a colorless liquid (1.34 g, 5.40 mmol, 54%). The crude was purified by flash column chromatography using hexane/EtOAc (10:1) as eluent.

^{1}H NMR (300 MHz, $CDCl_3$) δ 3.93 (s, 4H), 3.44 (t, J = 7.1 Hz, 2H), 1.84 – 1.66 (m, 6H), 1.52 (dd, J = 13.9, 4.6 Hz, 3H), 1.30 – 1.09 (m, 2H). **^{13}C NMR** (75 MHz, $CDCl_3$) δ 108.9, 64.3, 39.2, 34.9, 34.4, 31.9, 29.6. **IR** (cm^{-1}): 2923, 2879, 2358, 1740, 1713, 1244, 1092, 1033, 668. **HRMS** (ESI-TOF) m/z: $[M+H]^+$ Calcd. for $C_{10}H_{18}BrO_2$ 249.0490; Found: 249.0485.

8-(but-3-yn-1-yl)-1,4-dioxaspiro[4.5]decane (5-CCH)

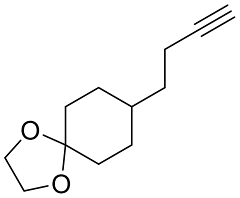

The title compound was synthesized using General Procedure C, starting from 5.4 mmol (1.35 g) of the corresponding bromoalkane (***5-Br***), yielding the alkyne (***5-CCH***) as a colorless liquid (867 mg, 4.50 mmol, 83%). The crude was purified by flash column chromatography using hexane/EtOAc (10:1) as eluent.
^{1}H NMR (300 MHz, $CDCl_3$) δ 3.91 (s, 4H), 2.19 (td, *J* = 7.1, 2.5 Hz, 2H), 1.92 (t, *J* = 2.6 Hz, 1H), 1.73 – 1.64 (m, 4H), 1.55 – 1.38 (m, 5H), 1.24 – 1.12 (m, 2H). **^{13}C NMR** (75 MHz, $CDCl_3$) δ 108.9, 84.5, 68.1, 64.1, 35.1, 34.7, 34.3, 29.6, 16.1. **IR**: 3056, 2952, 2321, 1739, 1716, 1434, 1264, 736, 704. **HRMS** (ESI-TOF) m/z: $[M]^+$ Calcd. for $C_{12}H_{18}O_2$ 194.1307; Found: 194.1300.

8-(4-bromobut-3-yn-1-yl)-1,4-dioxaspiro[4.5]decane (5)

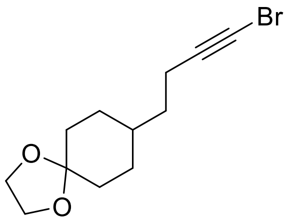

The title compound was synthesized using General Procedure D, starting from 4.5 mmol (867 mg) of the corresponding alkyne (***5-CCH***), yielding the bromoalkyne (***5***) as a colorless liquid (911 mg, 3.30 mmol, 74%). The crude was purified by flash column chromatography using hexane/EtOAc (10:1) as eluent.
^{1}H NMR (300 MHz, $CDCl_3$) δ 3.92 (s, 4H), 2.21 (t, *J* = 7.1 Hz, 2H), 1.70 (p, *J* = 3.4, 2.7 Hz, 4H), 1.56 – 1.36 (m, 5H), 1.28 – 1.16 (m, 2H). **^{13}C NMR** (75 MHz, $CDCl_3$) δ 108.9, 80.2, 64.2, 37.6, 35.1, 34.5, 34.3, 29.6, 17.4. **IR**: 3054, 2986, 1265, 743. **HRMS** (ESI-TOF) m/z: $[M]^+$ Calcd. for $C_{12}H_{17}BrO_2$ 272.0412; Found: 272.0406.

4-(4-bromobut-3-yn-1-yl)cyclohexan-1-one (6)

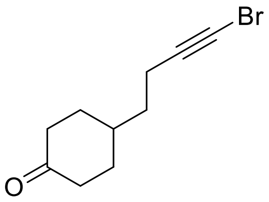

To the solution of **5** (92 mg, 0.34 mmol) in THF (0.25M) at room temperature, an equal volume of a 3M HCl solution was added. The reaction mixture was stirred magnetically for 3 hours. After this time, the reaction was extracted with Et_2O (x3) and the combined organic layers washed with $NaHCO_3$ sat. and brine. The organic layer was dried over Na_2SO_4, filtered and the solvent removed in vacuum. The obtained crude mixture was purified, if necessary, by flash column chromatography on silica gel using hexane/EtOAc (10:1) as eluent to afford **6** as a colorless liquid (77 mg, 0.34 mmol, 100%).
^{1}H NMR (300 MHz, $CDCl_3$) δ 2.40 – 2.23 (m, 6H), 2.11 – 2.00 (m, 2H), 1.85 (ddt, *J* = 11.1, 7.5, 3.7 Hz, 1H), 1.53 (q, *J* = 7.1 Hz, 2H), 1.39 (td, *J* = 13.4, 11.9, 7.0 Hz, 2H). **^{13}C NMR** (75 MHz, $CDCl_3$) δ 211.7, 79.6, 40.5, 38.2, 34.7, 33.7, 32.1, 17.5 **IR**: 3054, 2929,

2884, 2854, 1718, 1171, 1090, 797, 7381. **HRMS** (ESI-TOF) m/z: $[M+Na]^+$ Calcd. for $C_{10}H_{13}BrNaO$ 251.0047; Found: 251.0042.

tert-butyl 4-(2-hydroxyethyl)piperidine-1-carboxylate (7-9/20-OH)

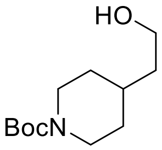

The title compound was synthesized using General Procedure A2, starting from 10 mmol (2.43 g) of carboxylic acid, yielding the crude alcohol (**7-9/20-_OH_**) as a colorless liquid (2.29 g, 10.00 mmol, 100%). This compound was used without further purification.
^{1}H NMR (300 MHz, $CDCl_3$) δ 4.02 – 3.93 (m, 2H), 3.58 (t, *J* = 6.3 Hz, 2H), 2.79 (s, 1H), 2.68 – 2.54 (m, 2H), 1.80 – 1.26 (m, 13H), 1.21 – 0.93 (m, 2H). **^{13}C NMR** (75 MHz, $CDCl_3$) δ 154.8, 79.2, 59.6, 43.8, 39.0, 32.3, 31.9, 28.3. **HRMS**: non purified

tert-butyl 4-(2-bromoethyl)piperidine-1-carboxylate (7-9/20-Br)

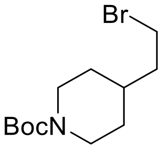

The title compound was synthesized using General Procedure B2, starting from 5 mmol (2.29 g) of the alcohol (**7-9/20-_OH_**), yielding the bromoalkane (**7-9/20-_Br_**) as a colorless liquid (1.58 g, 5.40 mmol, 54%). The crude was purified by flash column chromatography using hexane/EtOAc (10:1) as eluent.
^{1}H NMR (300 MHz, $CDCl_3$) δ 4.13 (dt, *J* = 13.2, 4.7 Hz, 2H), 3.46 (t, *J* = 6.9 Hz, 2H), 2.72 (t, *J* = 12.8 Hz, 2H), 1.82 (q, *J* = 6.7 Hz, 2H), 1.74 – 1.65 (m, 2H), 1.47 (s, 9H), 1.28 (t, *J* = 7.1 Hz, 1H), 1.21 – 1.04 (m, 2H). **^{13}C NMR** (75 MHz, $CDCl_3$) δ 154.6, 79.4, 43.5, 38.7, 38.5, 30.8, 28.4. **IR**: 2970, 2879, 1695, 1408, 1366, 1266, 1148, 741. **HRMS** (ESI-TOF) m/z: $[M+Na]^+$ Calcd. for $C_{12}H_{22}BrNNaO_2$ 314.0732; Found: 314.0726.

tert-butyl 4-(but-3-yn-1-yl)piperidine-1-carboxylate (7-9/20-CCH)

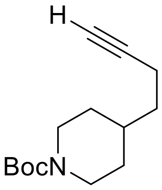

The title compound was synthesized using General Procedure C, starting from 5.4 mmol (1.58 g) of the bromoalkene (**7-9/20-_Br_**), yielding the alkyne (**7-9/20-_CCH_**) as a colorless

liquid (973 mg, 4.10 mmol, 76%). The crude was purified by flash column chromatography using hexane/EtOAc (10:1) as eluent.

^{1}H NMR (300 MHz, $CDCl_3$) δ 4.07 (s, 2H), 2.69 (d, *J* = 12.9 Hz, 2H), 2.21 (td, *J* = 7.2, 2.3 Hz, 2H), 1.93 (td, *J* = 2.6, 0.8 Hz, 1H), 1.83 – 1.31 (m, 13H), 1.06 (qd, *J* = 12.4, 4.2 Hz, 2H), 0.85 (ddt, *J* = 9.2, 6.8, 3.6 Hz, 1H). **^{13}C NMR** (75 MHz, $CDCl_3$) δ 154.8, 84.2, 79.2, 68.4, 43.6, 34.9, 34.8, 31.7, 28.4, 15.6. **IR**: 3308, 2932, 2247, 1677, 1428, 1167, 913, 738. **HRMS** (ESI-TOF) m/z: $[M+Na]^+$ Calcd. for $C_{14}H_{23}NNaO_2$ 260.1616; Found: 260.1621.

1-(4-(4-bromobut-3-yn-1-yl)piperidin-1-yl)-2,2,2-trifluoroethan-1-one (7-9-NH)

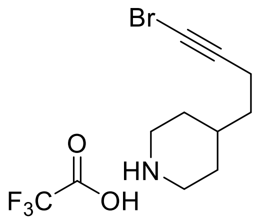

To a stirred solution of the corresponding **20** (1.10 g, 3.50 mmol) in DCM (0.12M) TFA (10 equiv) was added. The reaction mixture was stirred at room temperature for 16 hours and concentrated *in vacuo* to give **7-9-NH** (1.16 g, 3.50 mmol, 100%) as a brown oil that was used without further purification.

^{1}H NMR (300 MHz, $CDCl_3$) δ 8.15 (bs, 1H), 7.57 (bs, 1H), 3.53 (d, *J* = 12.7 Hz, 2H), 2.99 (q, *J* = 12.2 Hz, 2H), 2.29 (t, *J* = 6.9 Hz, 2H), 1.97 (d, *J* = 14.6 Hz, 2H), 1.76 (dt, *J* = 10.5, 3.4 Hz, 1H), 1.63 – 1.43 (m, 4H). **^{13}C NMR** (75 MHz, $CDCl_3$) δ 78.8, 44.8, 39.0, 33.7, 32.4, 28.3, 16.6. **^{19}F NMR** (282 MHz, $CDCl_3$) δ -76.0. **IR**: 3449, 3051, 2927, 2852, 2743, 1779, 1668, 1634, 1171, 798, 708. **HRMS** (ESI-TOF) m/z: $[M]^+$ Calcd. for $C_{11}H_{15}BrF_3NO_2$ 216.0310; Found: 216.0382.

4-(4-bromobut-3-yn-1-yl)piperidine 2,2,2-trifluoroacetate (7)

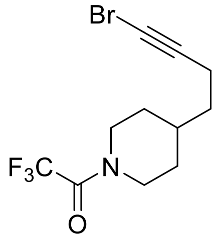

In a round-bottom flask, a solution of **7-9-*NH*** (157 mg, 0.50 mmol) and triethylamine (2.0 equiv.) in DCM (0.35M) was prepared and cooled to 0°C. Trifluoroacetic anhydride (TFFA, 1.1 equiv.) was then added dropwise with continuous stirring. An additional 0.5 equiv. of TFFA was added at 0ºC to ensure complete conversion. After that, the reaction mixture was allowed to warm to room temperature and stirred for 4 hours. After completion of the reaction (check by TLC), the mixture was quenched with water and extracted with DCM (x3). The combined organic layer was washed brine and dried over anhydrous Na_2SO_4, filtered, and concentrated under reduced pressure using a rotary evaporator. The resulting crude product was purified by flash chromatography using hexane/EtOAc (10:1) as eluent yielding **7** as a colorless liquid (137 mg, 0.44 mmol, 88%).

^{1}**H NMR** (300 MHz, $CDCl_3$) δ 4.51 (d, *J* = 13.3 Hz, 1H), 4.05 – 3.88 (m, 1H), 3.23 – 2.98 (m, 2H), 2.76 (t, *J* = 12.8 Hz, 1H), 2.25 (td, *J* = 7.1, 1.4 Hz, 2H), 1.88 – 1.61 (m, 3H), 1.48 (q, *J* = 7.1 Hz, 2H), 1.29 – 1.06 (m, 2H). ^{13}**C NMR** (75 MHz, $CDCl_3$) δ 155.1 (q, *J* = 35.9 Hz), 116.5 (q, *J* = 287.9 Hz), 79.3, 45.8, 43.6, 38.3, 34.5, 34.1, 32.0, 31.0, 16.7. ^{19}**F NMR** (282 MHz, $CDCl_3$) δ -68.82. **IR**: 3054, 2940, 2920, 1687, 1266, 1200, 1160, 1145, 738, 705. **HRMS** (ESI-TOF) m/z: $[M+H]^+$ Calcd. for $C_{11}H_{14}BrF_3NO_2$ 312.0211; Found: 312.0205.

benzyl 4-(4-bromobut-3-yn-1-yl)piperidine-1-carboxylate (8)

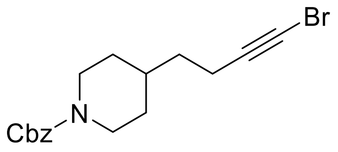

A solution of benzyl chloroformate (1.2 equiv.) in THF (1 M) was added dropwise to a solution of **7-9-** ***NH*** (157 mg, 0.50 mmol) and sodium hydrogen carbonate (1.4 equiv.) in water (1 M) at 0°C. The reaction mixture was then allowed to warm to room temperature and stirred vigorously for 16 hours. After completion of the reaction, the mixture was quenched with a saturated ammonium chloride solution and extracted with ethyl acetate (x2). The combined organic layers were washed with brine dried over anhydrous Na_2SO_4 and filtered. The solvents were removed under reduced pressure and the obtained crude mixture was purified by flash column chromatography on silica gel using hexane/EtOAc (10:1) as eluent to afford **8** as a colorless liquid (156 mg, 0.45 mmol, 89%).
^{1}**H NMR** (300 MHz, $CDCl_3$) δ 7.39 – 7.26 (m, 5H), 5.12 (s, 2H), 4.17 (d, *J* = 13.1 Hz, 2H), 2.78 (t, *J* = 12.7 Hz, 2H), 2.24 (t, *J* = 7.1 Hz, 2H), 1.82 – 1.61 (m, 3H), 1.48 (t, *J* = 6.9 Hz, 2H), 1.12 (td, *J* = 12.0, 11.6, 4.3 Hz, 2H). ^{13}**C NMR** (75 MHz, $CDCl_3$) δ 155.2), 136.9, 128.4, 127.9, 127.8, 79.8, 66.9, 44.0, 34.7, 34.6, 31.5, 16.9. **HRMS** (ESI-TOF) m/z: $[M]^+$ Calcd. for $C_{17}H_{20}BrNO_2$ 349.0677; Found: 349.0699.

4-(4-bromobut-3-yn-1-yl)-1-tosylpiperidine (9)

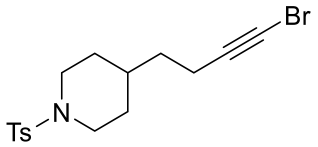

p-Toluenesulfonyl chloride (1.1 equiv.) was added to a solution of ***7-9-NH*** (157 mg, 0.50 mmol) and triethylamine (3.0 equiv.) in DCM (0.25 M) at 0°C. The reaction mixture was stirred at this temperature for 20 hours and then allowed to warm to room temperature. After completion of the reaction, the mixture was quenched with water and extracted with DCM (x3). The combined organic phases were washed with brine, dried over anhydrous Na_2SO_4, and filtered. The solvents were removed under reduced pressure, and the crude product was purified by flash chromatography using hexane/EtOAc (10:1) as the eluent, yielding the ***9*** as a colorless liquid (170 mg, 0.46 mmol, 92%).
^{1}**H NMR** (300 MHz, $CDCl_3$) δ 7.63 (d, *J* = 8.0 Hz, 2H), 7.31 (d, *J* = 8.0 Hz, 2H), 3.76 (dd, *J* = 11.9, 3.8 Hz, 2H), 2.42 (s, 3H), 2.30 – 2.14 (m, 4H), 1.76 – 1.67 (m, 2H), 1.49 –

1.21 (m, 5H). **^{13}C NMR** (75 MHz, $CDCl_3$) δ 143.4, 133.0, 129.5, 127.7, 79.5, 46.3, 38.0, 34.1, 33.7, 30.9, 21.5, 16.8. **HRMS** (ESI-TOF) m/z: $[M]^+$ Calcd. for $C_{16}H_{21}BrNSO_2$ 369.0398; Found: 369.0403. **m.p.** = 149-152.

4-(4-bromobut-3-yn-1-yl)-1,1-difluorocyclohexane (10)

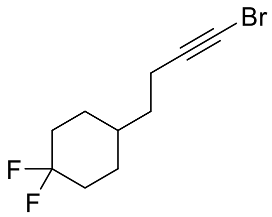

DAST (2 equiv) was added to a solution of **6** (92 mg, 0.4 mmol) in anhydrous DCM (0.50 M) at 0 ºC. The reaction mixture was stirred for 18 hours at room temperature (followed by TLC). Then, the reaction was quenched carefully with H_2O, extracted with DCM and finally the combined organic layers washed with brine. The organic layer was dried over Na_2SO_4, filtered and the solvent removed in vacuum. The crude obtained was purified by flash column chromatography on silica gel using hexane as eluent to afford **10** as a colorless liquid (60 mg, 0.24 mmol, 60%).

1H NMR (300 MHz, $CDCl_3$) δ 2.25 (t, J = 7.0 Hz, 2H), 2.07 (dtd, J = 13.7, 7.1, 6.4, 3.8 Hz, 2H), 1.83 – 1.73 (m, 4H), 1.49 (td, J = 5.8, 5.4, 3.1 Hz, 3H), 1.32 – 1.19 (m, 2H). **^{13}C NMR** (75 MHz, $CDCl_3$) δ 123.8 (t, *J* = 241.0 Hz), 79.9, 38.2, 34.7, 33.8, 28.7, 28.6, 17.5. **^{19}F NMR** (282 MHz, CDC13) δ -91.65 (d, *J* = 235.6 Hz, 1F), -102.10 (dd, *J* = 237.7, 34.1 Hz, 1F). **HRMS** (ESI-TOF) m/z: $[M-H]^+$ Calcd. for $C_{10}H_{14}BrF_2$ 249.0090; Found: 249.0081.

4-(2-bromoethyl)tetrahydro-2H-thiopyran 1,1-dioxide (11-Br)

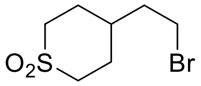

The title compound was synthesized using General Procedure B1, starting from 2 mmol (357 mg) of the corresponding alcohol, yielding the bromoalkane (***11-Br***) as a colorless liquid (304 mg, 1.26 mmol, 63%). The crude was purified by flash column chromatography using hexane/EtOAc (10:1) as eluent.

^{1}H NMR (300 MHz, $CDCl_3$) δ 3.37 (t, *J* = 6.3 Hz, 2H), 2.96 (t, *J* = 4.0 Hz, 4H), 2.02 (dt, *J* = 8.6, 4.0 Hz, 2H), 1.78 (dq, *J* = 9.0, 4.8, 4.3 Hz, 5H). **^{13}C NMR** (75 MHz, $CDCl_3$) δ 50.4, 37.2, 33.1, 30.6, 29.0. **HRMS**: unstable

4-(but-3-yn-1-yl)tetrahydro-2H-thiopyran 1,1-dioxide (11-CCH)

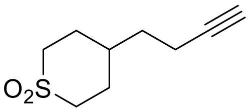

The title compound was synthesized using General Procedure C, starting from 1.3 mmol (304 mg) of the corresponding bromoalkane (***11-Br***), yielding the alkyne (***11-CCH***) as a colorless liquid (75 mg, 0.4 mmol, 31%). The crude was purified by flash column chromatography using hexane/EtOAc (10:1) as eluent.

^{1}H NMR (300 MHz, $CDCl_3$) δ 3.05 – 2.86 (m, 4H), 2.23 (td, J = 7.0, 2.7 Hz, 2H), 2.15 – 2.05 (m, 2H), 1.96 (t, J = 2.7 Hz, 1H), 1.91 – 1.72 (m, 3H), 1.53 (q, J = 6.8 Hz, 2H). **^{13}C NMR** (75 MHz, $CDCl_3$) δ 83.0, 69.2, 50.7, 33.7, 33.2, 29.4, 15.9. **HRMS** (ESI-TOF) m/z: $[M-CH_2CCH]^+$ Calcd. for $C_6H_{11}O_2S$ 147.0480; Found: 147.0480.

4-(4-bromobut-3-yn-1-yl)tetrahydro-2H-thiopyran 1,1-dioxide (11)

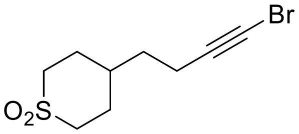

The title compound was synthesized using General Procedure D, starting from 0.4 mmol (75 mg) of the corresponding alkyne (***11-CCH***), yielding the bromoalkyne (***11***) as a colorless liquid (38 mg, 0.14 mmol, 36%). The crude was purified by flash column chromatography using hexane/EtOAc (10:1) as eluent.

^{1}H NMR (300 MHz, $CDCl_3$) δ 3.07 – 2.87 (m, 4H), 2.27 (t, J = 7.0 Hz, 2H), 2.17 – 2.03 (m, 2H), 1.90 – 1.66 (m, 3H), 1.54 (q, J = 6.8 Hz, 2H). **^{13}C NMR** (75 MHz, $CDCl_3$) δ 78.8, 50.7, 39.0, 33.9, 33.1, 29.4, 17.2. **HRMS** (ESI-TOF) m/z: $[M-Br]^+$ Calcd. for $C_9H_{13}SO_2$ 185.0636; Found: 185.0639.

4-(2-bromoethyl)-1,1-dimethylcyclohexane (12-Br)

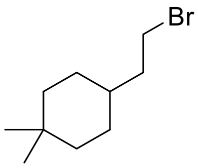

The title compound was synthesized using General Procedure B1, starting from 2 mmol (357 mg) of the corresponding alcohol, yielding the bromoalkane (***12-Br***) as a colorless liquid (304 mg, 2.00 mmol, 63%). The crude was purified by flash column chromatography using hexane/EtOAc (10:1) as eluent.

^{1}H NMR (300 MHz, $CDCl_3$) δ 3.44 (t, J = 7.2 Hz, 2H), 1.78 (q, J = 7.1 Hz, 2H), 1.59 – 1.50 (m, 2H), 1.42 – 1.33 (m, 3H), 1.28 – 1.07 (m, 4H), 0.88 (d, J = 10.1 Hz, 6H). **^{13}C NMR** (75 MHz, $CDCl_3$) δ 40.0, 38.8, 36.1, 32.7, 32.0, 30.0, 28.4, 24.4. **HRMS**: unstable.

4-(but-3-yn-1-yl)-1,1-dimethylcyclohexane (12-CCH)

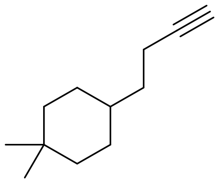

The title compound was synthesized using General Procedure C, starting from 2 mmol (438 mg) of the corresponding bromoalkane, yielding the alkyne (***12-CCH***) as a colorless

liquid (250 mg, 1.50 mmol, 76%). The crude was purified by flash column chromatography using n-hexane as eluent.
^{1}H NMR (300 MHz, $CDCl_3$) δ 2.20 (td, *J* = 7.4, 2.6 Hz, 2H), 1.92 (d, *J* = 2.7 Hz, 1H), 1.59 – 1.42 (m, 4H), 1.40 – 1.30 (m, 3H), 1.13 (ddd, *J* = 24.4, 15.0, 11.9 Hz, 5H), 0.89 (s, 3H), 0.86 (s, 3H). **^{13}C NMR** (75 MHz, $CDCl_3$) δ 84.9, 67.9, 39.0, 36.5, 35.6, 32.8, 30.1, 28.6, 24.4, 16.0. **HRMS**: non-ionizable.

4-(4-bromobut-3-yn-1-yl)-1,1-dimethylcyclohexane (12)

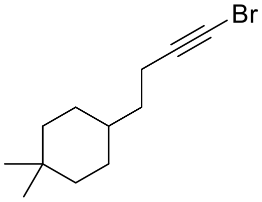

The title compound was synthesized using General Procedure D, starting from 1.5 mmol (250 mg) of the corresponding alkyne (***12-CCH***), yielding the bromoalkyne (***12***) as a colorless liquid (321 mg, 1.30 mmol, 88%). The crude was purified by flash column chromatography using n-hexane as eluent.
^{1}H NMR (300 MHz, $CDCl_3$) δ 2.22 (t, *J* = 7.4 Hz, 2H), 1.58 – 1.49 (m, 2H), 1.44 (q, *J* = 7.2 Hz, 2H), 1.39 – 1.31 (m, 2H), 1.23 – 1.00 (m, 5H), 0.89 (s, 3H), 0.85 (s, 3H). **^{13}C NMR** (75 MHz, $CDCl_3$) δ 80.6, 38.9, 37.3, 36.5, 35.4, 32.8, 30.1, 28.5, 24.4, 17.3. **HRMS** (ESI-TOF) m/z: $[M]^+$ Calcd. for $C_{12}H_{19}Br$ 242.0670; Found: 242.0664.

2-(oxepan-4-yl)ethan-1-ol (13-OH)

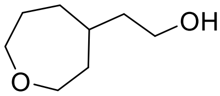

The title compound was synthesized using General Procedure A1, starting from 3.2 mmol (500 mg) of the proper carboxylic acid, yielding the crude alcohol (***13-OH***) as a colorless liquid (383 mg, 2.70 mmol, 84%). This compound was used without further purification.
^{1}H NMR (300 MHz, $CDCl_3$) δ 3.73 (ddd, *J* = 11.7, 7.5, 4.6 Hz, 2H), 3.66 – 3.48 (m, 4H), 2.39 – 2.15 (m, 1H), 1.72 (tdd, *J* = 18.2, 8.9, 4.3 Hz, 5H), 1.60 – 1.23 (m, 9H). **^{13}C NMR** (75 MHz, $CDCl_3$) δ 69.8, 68.1, 60.8, 40.2, 37.1, 35.1, 32.8, 28.9. **HRMS** (ESI-TOF) m/z: $[M]^+$ Calcd. for $C_8H_{16}O_2$ 144.1150; Found: 144.1146.

4-(2-bromoethyl)oxepane (13-Br)

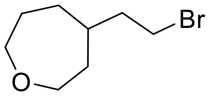

The title compound was synthesized using General Procedure B1, starting from 2.7 mmol (383 mg) of the alcohol (***13-OH***), yielding the bromoalkane (***13-Br***) as a colorless liquid (438 mg, 2.10 mmol, 79%). The crude was purified by flash column chromatography using hexane/EtOAc (10:1) as eluent.

^{1}H NMR (300 MHz, $CDCl_3$) δ 3.83 – 3.47 (m, 4H), 3.41 (t, *J* = 7.0 Hz, 2H), 1.86 – 1.63 (m, 7H), 1.37 (dd, *J* = 20.2, 10.5 Hz, 2H). **^{13}C NMR** (75 MHz, $CDCl_3$) δ 69.8, 67.9, 40.1, 37.1, 36.4, 32.1, 32.1, 28.8. **HRMS**: unstable

4-(but-3-yn-1-yl)oxepane (13-CCH)

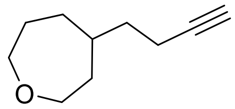

The title compound was synthesized using General Procedure C, starting from 2.1 mmol (438 mg) of the corresponding bromoalkane (***13-Br***), yielding the alkyne (***13-CCH***) as a colorless liquid (277 mg, 1.80 mmol, 86%). The crude was purified by flash column chromatography using hexane/EtOAc (10:1) as eluent.
^{1}H NMR (300 MHz, $CDCl_3$) δ 3.73 (dd, *J* = 11.9, 6.1 Hz, 2H), 3.67 – 3.48 (m, 2H), 2.18 (t, *J* = 7.6 Hz, 2H), 1.91 (d, *J* = 2.7 Hz, 1H), 1.72 (q, *J* = 11.1, 6.5 Hz, 5H), 1.56 – 1.20 (m, 4H). **^{13}C NMR** (75 MHz, $CDCl_3$) δ 84.3, 69.6, 68.2 67.9, 37.3, 36.5, 35.7, 32.1, 28.7, 16.2. **HRMS** (ESI-TOF) m/z: [M]+ Calcdd for C10H16BrO 152.1201; Found 152.1210.

4-(4-bromobut-3-yn-1-yl)oxepane (13)

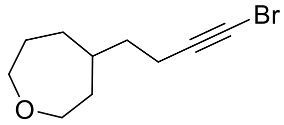

The title compound was synthesized using General Procedure D, starting from 2.1 mmol (277 mg) of the corresponding alkyne (***13-CCH***), yielding the bromoalkyne (***13***) as a colorless liquid (375 mg, 1.70 mmol, 81%). The crude was purified by flash column chromatography using hexane/EtOAc (10:1) as eluent.
^{1}H NMR (300 MHz, $CDCl_3$) δ 3.73 (dt, *J* = 7.8, 4.0 Hz, 2H), 3.59 (ddd, *J* = 15.7, 12.4, 9.4 Hz, 2H), 2.20 (td, *J* = 7.3, 2.3 Hz, 2H), 1.83 – 1.61 (m, 5H), 1.57 – 1.19 (m, 4H). **^{13}C NMR** (75 MHz, $CDCl_3$) δ 80.0, 69.7, 67.9, 37.7, 37.3, 36.4, 35.5, 32.1, 28.7, 17.5. **HRMS** (ESI-TOF) m/z: $[M]^+$ Calcd. for $C_{10}H_{15}BrO$ 230.0306; Found: 230.0312.

5-(4-bromobut-3-yn-1-yl)oxepan-2-one (14)

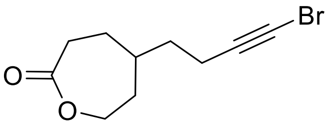

Under inert atmosphere, m-CPBA (77% maximum purity, 2 equiv.) was gradually added with stirring to a solution of **6** (92 mg, 0.40 mmol) in dichloromethane (0.05M). The reaction mixture was stirred at room temperature for 24 hours. After the reaction was finished (check by TLC), the mixture was filtered to remove any solid impurities. The organic layer was then washed twice with an aqueous solution of sodium dithionite, followed by washing with a saturated solution of potassium carbonate. The organic phase was dried over anhydrous sodium sulfate and subsequently filtered. The solvents were

removed under reduced pressure. The obtained crude mixture was purified by flash column chromatography on silica gel using hexane/EtOAc (10:1) as eluent to afford **14** as a colorless liquid (74 mg, 0.30 mmol, 75%).

^{1}**H NMR** (300 MHz, $CDCl_3$) δ 4.30 – 4.05 (m, 2H), 2.63 – 2.55 (m, 2H), 2.20 (t, *J* = 7.1 Hz, 2H), 1.98 – 1.83 (m, 2H), 1.70 (dtt, *J* = 13.8, 7.0, 3.6 Hz, 1H), 1.50 – 1.36 (m, 3H), 1.32 – 1.14 (m, 1H). ^{13}**C NMR** (75 MHz, $CDCl_3$) δ 175.7, 79.1, 67.7, 38.5, 38.4, 34.7, 34.3, 32.8, 28.2, 16.9. **IR**: 2931, 2851, 1716, 766. **HRMS** (ESI-TOF) m/z: $[M+Na]^+$ Calcd. for $C10H_{13}BrNaO_2$ 266.9997; Found: 266.9991.

2-(6,6-difluorospiro[3.3]heptan-2-yl)ethan-1-ol (15-OH)

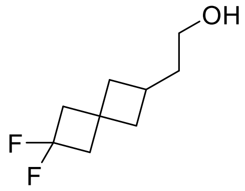

The title compound was synthesized using General Procedure A1, starting from 2.60 mmol (500 mg) of the proper carboxylic acid, yielding the crude alcohol (***15-OH***) as a colorless liquid (471 mg, 2.60 mmol, 100%). This compound was used without further purification.

^{1}**H NMR** (300 MHz, $CDCl_3$) δ 3.75 – 3.62 (m, 1H), 3.51 (t, *J* = 6.7 Hz, 2H), 2.48 (dt, *J* = 34.4, 12.4 Hz, 3H), 2.31 – 2.09 (m, 4H), 1.86 – 1.70 (m, 2H), 1.61 (q, *J* = 6.8 Hz, 2H). ^{13}**C NMR** (75 MHz, $CDCl_3$) δ 119.8 (t, *J* = 280.0 Hz), 67.9, 60.8, 47.9 (t, *J* = 21.5 Hz), 47.0 (t, *J* = 21.4 Hz), 40.0, 39.4, 28.9 (t, *J* = 8.8 Hz), 26.9, 25.5. ^{19}**F NMR** (282 MHz, $CDCl_3$) δ -90.82. **HRMS** (ESI-TOF) m/z: $[M-H_2CH_2OH]^+$ Calcd. for $C_8H_9F_2$ 143.0672; Found: 143.0671.

6-(2-bromoethyl)-2,2-difluorospiro[3.3]heptane (15-Br)

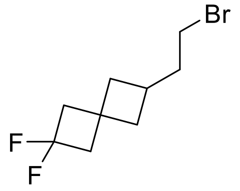

The title compound was synthesized using General Procedure B1, starting from 2.6 mmol (471 mg) of the corresponding alcohol (***15-OH***), yielding the bromoalkane (***15-Br***) as a colorless liquid (469 mg, 2.00 mmol, 77%). The crude was purified by flash column chromatography using n-hexane as eluent.

^{1}**H NMR** (300 MHz, $CDCl_3$) δ 3.30 (t, *J* = 6.9 Hz, 2H), 2.59 (t, *J* = 12.4 Hz, 2H), 2.52 – 2.30 (m, 3H), 2.30 – 2.15 (m, 2H), 1.94 (q, *J* = 7.0 Hz, 2H), 1.80 (dt, *J* = 11.6, 5.7 Hz, 2H). ^{13}**C NMR** (75 MHz, $CDCl_3$) δ 119.7 (t, *J* = 279.9 Hz), 47.8 (t, *J* = 21.6 Hz), 46.9 (t, *J* = 21.5 Hz), 39.6, 39.5, 31.2, 29.0, 28.8 (t, *J* = 8.8 Hz). ^{19}**F NMR** (282 MHz, $CDCl_3$) δ -90.87. **HRMS**: unstable

6-(but-3-yn-1-yl)-2,2-difluorospiro[3.3]heptane (15-CCH)

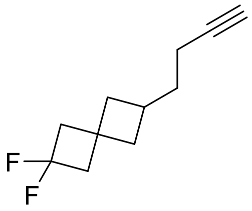

The title compound was synthesized using General Procedure C, starting from 2 mmol (469 mg) of the corresponding bromoalkane (***15-Br***), yielding the alkyne (***15-CCH***) as a colorless liquid (320 mg, 1.70 mmol, 76%). The crude was purified by flash column chromatography using n-hexane as eluent.

^{1}H NMR (300 MHz, $CDCl_3$) δ 2.51 (dt, *J* = 37.0, 12.4 Hz, 4H), 2.36 – 2.15 (m, 3H), 2.09 (t, *J* = 7.2 Hz, 2H), 1.91 (t, *J* = 2.7 Hz, 1H), 1.76 (dt, *J* = 11.6, 5.6 Hz, 2H), 1.60 (qd, *J* = 7.2, 1.9 Hz, 2H). **^{13}C NMR** (75 MHz, $CDCl_3$) δ 119.8 (t, *J* = 280.0 Hz), 84.2, 68.2, 47.8 (t, *J* = 21.5 Hz), 46.9 (t, *J* = 21.5 Hz), 39.7, 35.3, 29.4, 28.6 (t, *J* = 8.8 Hz), 16.3. **^{19}F NMR** (282 MHz, $CDCl_3$) δ -90.74. **HRMS** (ESI-TOF) m/z: $[M]^+$ Calcd. for $C_{11}H_{14}F_2$ 184.1064; Found: 184.1054.

6-(4-bromobut-3-yn-1-yl)-2,2-difluorospiro[3.3]heptane (15)

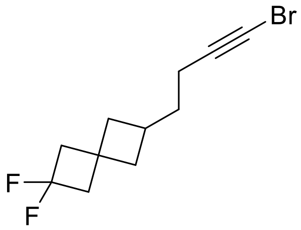

The title compound was synthesized using General Procedure D, starting from ***15-CCH*** (320 mg, 1.70 mmol), yielding ***15*** as a colorless liquid (325 mg, 1.20 mmol, 71%). The crude was purified by flash column chromatography using n-hexane as eluent.

^{1}H NMR (300 MHz, $CDCl_3$) δ 2.51 (dt, *J* = 38.0, 12.4 Hz, 4H), 2.35 – 2.05 (m, 5H), 1.79 – 1.71 (m, 2H), 1.59 (q, *J* = 7.1 Hz, 2H). **^{13}C NMR** (75 MHz, $CDCl_3$) δ 119.82 (t, *J* = 280.0 Hz), 79.9, 47.9 (*J* = 21.5 Hz), 46.9 (t, *J* = 21.5 Hz), 39.7, 37.7, 35.1, 29.5, 28.6 (t, *J* = 8.7 Hz), 17.6. **^{19}F NMR** (282 MHz, $CDCl_3$) δ -90.79. **IR**: 2915, 2851, 1724, 1619, 766. **HRMS** (ESI-TOF) m/z: $[M-Br]^+$ Calcd. for $C_{11}H_{13}F_2$ 183.0974; Found: 183.0985.

1-((tetrahydro-2H-pyran-4-yl)methyl)cyclopentane-1-carboxylic acid (16-CO_2H)

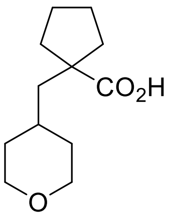

A solution of cyclopentanecarboxylic acid (1.14g, 10.00 mmol) in dry THF (0,2M), was treated with LDA (1.00 M, 24.0ml, 2.4 equiv) at -78 °C, and the mixture was stirred 15 minutes at this temperature. Then 4-(iodomethyl)tetrahydro-2H-pyran (2,26 g, 10.00 mmol, 1 equiv.) in dry THF (10 mL), was added and the reaction mixture was allowed to

reach room temperature and stirred overnight. Upon completion (judged by TLC), water was carefully added, and the crude reaction mixture was extracted with Et_2O (3x100 mL) and the combined organic layer washed with water and brine and then dried with anhydrous Na_2SO_4, filtered and concentrated. The crude product was purified by flash column chromatography using hexane/EtOAc (3:1) as eluent, yieding the title compound as a colorless oil (1,73 g, 83%).

^{1}H NMR (300 MHz, $CDCl_3$) δ 3.88 (dd, *J* = 11.6, 3.3 Hz, 1H), 3.31 (dt, *J* = 13.7, 6.8 Hz, 1H), 2.41 (dt, *J* = 8.2, 3.6 Hz, 1H), 2.38 – 2.12 (m, 1H), 2.16 – 1.82 (m, 3H), 1.73 (dd, *J* = 6.8, 4.0 Hz, 1H), 1.60 – 1.44 (m, 2H), 1.40 – 1.00 (m, 1H). **^{13}C NMR** (75 MHz, $CDCl_3$) 183.2, 67.8, 47.2, 45.2, 33.2, 31.5, 25.1, 16.1. **HRMS** (ESI-TOF) m/z: $[M]^+$ Calcdd for $C_{12}H_{20}O_3$ 212.1412; Found 212.1416.

(1-((tetrahydro-2H-pyran-4-yl)methyl)cyclopentyl)methanol (16-OH)

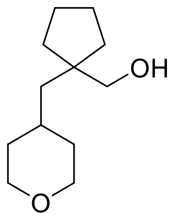

The title compound was synthesized using General Procedure A1, starting from ***16-CO₂H*** (1.06 g, 5.00 mmol) of the proper carboxylic acid, yielding the crude alcohol (***16-OH***) as a colorless liquid (0.99 g, 5.00 mmol, 100%). This compound was used without further purification.

^{1}H NMR (300 MHz, $CDCl_3$) δ 3.87 (dd, *J* = 11.6, 2.6 Hz, 2H), 3.59 (s, 2H), 3.32 (td, *J* = 11.7, 2.1 Hz, 2H), 2.05 (bs, 1H), 1.89 – 1.70 (m, 2H), 1.67 – 1.52 (m, 4H), 1.51 – 1.40 (m, 5H), 1.30 (td, *J* = 13.3, 12.6, 4.5 Hz, 2H). **^{13}C NMR** (75 MHz, $CDCl_3$) 67.7, 67.0, 44.1, 42.3, 33.8, 31.6, 29.7, 15.1. **HRMS**: non purified

1-((tetrahydro-2H-pyran-4-yl)methyl)cyclopentane-1-carbaldehyde (16-CHO)

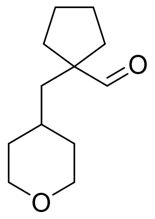

In a Schlenk flask under Ar, DMSO (2.8 equiv.) was added dropwise to a solution of oxalyl chloride (1.40 equiv.) in dry DCM (0.50 M) at -78 ºC. Then, **16-*OH*** (0.99g, 5.00 mmol) dissolved in dry DCM (1M) was added dropwise to the reaction mixture at the same temperature. After stirring for 15 minutes triethylamine (5.0 equiv.) was added dropwise and the reaction was allowed to reach room temperature. Then, water was added, and the crude reaction mixture was extracted with Et_2O (x3), the combined organic layers were washed with 1N HCl, saturated aqueous $NaHCO_3$ and saturated aqueous NaCl. The combined organic layers were dried over Na_2SO_4 and concentrated under

vacuum. Crude **16-*CHO*** was used in the following step without further purification (643mg, 66%).

^{1}H NMR (300 MHz, $CDCl_3$) δ 9.45 (s, 1H), 3.83 (dd, *J* = 11.4, 4.4 Hz, 2H), 3.25 (td, *J* = 11.9, 2.1 Hz, 2H), 2.25 – 1.86 (m, 2H), 1.76 – 0.94 (m, 13H). **^{13}C NMR** (75 MHz, $CDCl_3$) 205.0, 67.8, 58.1, 43.7, 34.0, 32.9, 32.8, 24.8. **HRMS**: non purified.

4-((1-(2,2-dibromovinyl)cyclopentyl)methyl)tetrahydro-2H-pyran (16-C=CBr$_2$)

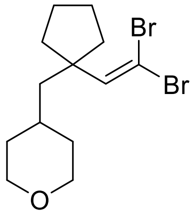

To a solution of **16-*CHO*** (643 mg, 3.28mmol) in anhydrous dichloromethane (0.10 M), CBr_4 (1.5 equiv.) was added followed by PPh_3 (3.0 equiv.). The reaction mixture was stirred for 15-30 minutes and then hexane was added. Solids were filtered through alternate pads of celite /silica gel/ celite. Removal of solvents under vacuum followed by flash column chromatography on silica gel using n-hexane as eluent, yielded **16-*C=CBr$_2$*** as a colorless gum (472mg, 41%).

^{1}H NMR (300 MHz, $CDCl_3$) δ 7.25 (s, 1H), 4.57 (d, *J* = 10.5 Hz, 1H), 4.02 (tdd, *J* = 11.7, 3.4, 1.9 Hz, 4H), 2.77 – 2.45 (m, 3H), 2.43 – 2.09 (m, 20H), 2.09 – 1.85 (m, 3H). **^{13}C NMR** (75 MHz, $CDCl_3$) 146.7, 86.6, 68.0, 50.6, 45.0, 39.6, 34.5, 32.9, 23.7. **HRMS**: unstable.

4-((1-ethynylcyclopentyl)methyl)tetrahydro-2H-pyran (16-CCH)

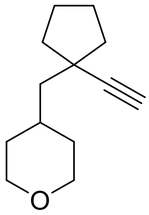

BuLi (1.6 M in hexanes, 1.8 mL, 2.1 equiv.) was added dropwise to a solution of **16-*C=CBr$_2$*** (472mg, 1.34 mmol) in dry THF (0.1M) at -78 ºC. The mixture was stirred for 1h at this temperature and then quenched with MeOH (6 mL) and allowed to reach room temperature. After washing with brine, the organic layer was dried over Na_2SO_4, filtered and concentrated. Removal of solvents under vacuum followed by flash column chromatography on silica gel using n-hexane as eluent, yielded **16-*CCH*** as a colorless oil (231 mg, 90%).

^{1}H NMR (300 MHz, $CDCl_3$) δ 3.89 (ddd, *J* = 11.8, 4.2, 2.0 Hz, 2H), 3.38 (td, *J* = 11.8, 2.0 Hz, 2H), 2.08 (s, 1H), 2.01 – 1.53 (m, 9H), 1.53 – 1.20 (m, 6H). **^{13}C NMR** (75 MHz, $CDCl_3$) 91.6, 69.1, 68.1, 47.6, 42.1, 41.3, 34.5, 33.5, 24.0. **HRMS** (ESI-TOF) m/z: [M]+ Calcdd for C13H20O3 192.1514; Found 192.1511.

4-((1-(bromoethynyl)cyclopentyl)methyl)tetrahydro-2H-pyran (16)

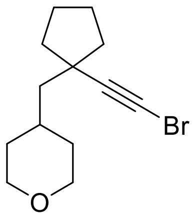

The title compound was synthesized using General Procedure D, starting from 1.2 mmol (231 mg) of the corresponding alkyne (***16-CCH***), yielding the bromoalkyne (***16***) as a colorless liquid (252 mg, 0.92 mmol, 77%). The crude was purified by flash column chromatography using hexane/EtOAc (10:1) as eluent.

^{1}**H NMR** (300 MHz, $CDCl_3$) δ 3.89 (ddd, *J* = 11.5, 3.8, 1.7 Hz, 2H), 3.37 (td, *J* = 11.8, 1.9 Hz, 2H), 2.06 – 1.52 (m, 9H), 1.50 – 1.12 (m, 6H). ^{13}**C NMR** (75 MHz, $CDCl_3$) 86.9, 68.1, 47.5, 43.4, 41.0, 38.8, 34.3, 33.4, 24.0. **HRMS** (ESI-TOF) m/z: [M]+ Calcdd for C13H19BrO 270.0619; Found 270.0622.

***tert*-butyl 3-(2-bromoethyl)azepane-1-carboxylate (17-Br)**

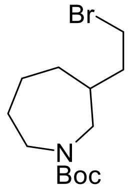

The title compound was synthesized using General Procedure B2, starting from 2.55 mmol (620 g) of the commercial alcohol, yielding the bromoalkane (***17-Br***) as a colorless liquid (553 mg, 1.81 mmol, 71%). The crude was purified by flash column chromatography using hexane/EtOAc (10:1) as eluent. Some signals in the ^{1}H and ^{13}C are duplicated due to the presence rotamers.

^{1}**H NMR** (300 MHz, $CDCl_3$) δ 3.62 – 3.50 (m, 1H), 3.43 (t, *J* = 6.8 Hz, 3H), 3.22 (td, *J* = 14.1, 7.1 Hz, 1H), 2.88 (ddd, *J* = 37.8, 14.1, 8.6 Hz, 1H), 1.93 – 1.64 (m, 8H), 1.46 (m, 14H), 1.21 – 1.08 (m, 1H). ^{13}**C NMR** (75 MHz, $CDCl_3$) δ 155.5, 79.3, 79.1, 50.8, 47.5, 46.9, 38.2, 38.1, 37.5, 37.3, 33.7, 32.6, 31.5, 28.5, 28.2, 27.9, 25.0, 24.4. **HRMS** (ESI-TOF) m/z: $[M]^+$ Calcd. for $C_{13}H_{24}BrNO_2$ 305.0990; Found: 305.1002.

***tert*-butyl 3-(but-3-yn-1-yl)azepane-1-carboxylate (17-CCH)**

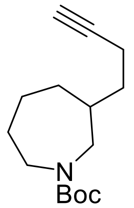

The title compound was synthesized using General Procedure C, starting from 1.81 mmol (553 mg) of the bromoalkene (***17-Br***), yielding the alkyne (***17-CCH***) as a colorless liquid (221 mg, 0.88 mmol, 49%). The crude was purified by flash column chromatography using hexane/EtOAc (10:1) as eluent. Some signals in the ^{1}H and ^{13}C are duplicated due to the presence rotamers.

^{1}H NMR (300 MHz, $CDCl_3$) δ 3.64 – 3.47 (m, 2H), 3.16 (ddd, J = 13.9, 10.7, 6.0 Hz, 1H), 2.79 (ddd, J = 30.6, 14.1, 9.4 Hz, 1H), 2.20 (td, J = 7.3, 2.6 Hz, 2H), 1.94 – 1.90 (m, 1H), 1.85 – 1.64 (m, 5H), 1.43 (m, 14H), 1.27 – 1.07 (m, 1H). **^{13}C NMR** (75 MHz, $CDCl_3$) δ 155.6, 84.3, 84.2, 79.1, 78.9, 68.3, 68.2, 51.4, 51.0, 47.3, 46.8, 38.6, 38.3, 33.9, 33.1, 32.9, 32.5, 28.4, 28.1, 27.8, 25.1, 24.4, 16.4. **IR**: 3306, 3052, 2930, 2859, 1681, 1418, 1264, 1160, 733, 703, 418. **HRMS** (ESI-TOF) m/z: $[M]^+$ Calcd. for $C_{15}H_{25}NO_2$ 251.1885; Found: 251.1888.

tert-butyl 3-(4-bromobut-3-yn-1-yl)azepane-1-carboxylate (17-NBoc)

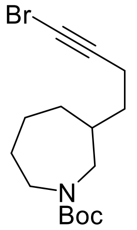

The title compound was synthesized using General Procedure D, starting from 0.88 mmol (221 mg) of the corresponding alkyne (***17-CCH***), yielding the bromoalkyne (***17-NBoc***) as a colorless liquid (261 mg, 0.79 mmol, 90%). The crude was purified by flash column chromatography using hexane/EtOAc (10:1) as eluent.

^{1}H NMR (300 MHz, $CDCl_3$) δ 3.63 – 3.52 (m, 2H), 3.17 (ddt, J = 19.3, 13.7, 5.8 Hz, 1H), 2.80 (ddd, J = 41.1, 14.0, 9.3 Hz, 1H), 2.25 (t, J = 7.3 Hz, 2H), 1.80 – 1.64 (m, 5H), 1.45 (d, J = 6.0 Hz, 14H), 1.19 – 1.06 (m, 1H).**^{13}C NMR** (75 MHz, $CDCl_3$) δ 155.6, 80.0, 79.2, 79.0, 51.3, 51.0, 47. , 46.8, 38.5, 38.4, 38.0, 34.0, 32.9, 32.7, 32.6, 28.5, 28.2, 27.9, 25.1, 24.4, 17.7. **HRMS** (ESI-TOF) m/z: $[M-CH_3]^+$ Calcd. for $C_{14}H_{21}BrNO_2$ 314.0756; Found: 314.0757

1-(3-(4-bromobut-3-yn-1-yl)azepan-1-yl)-2,2,2-trifluoroethan-1-one (17)

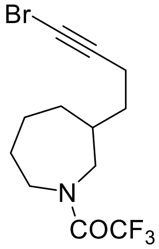

To a stirred solution of the corresponding N-Boc protected amine ***17-NBoc*** (261 mg, 0.79 mmol) in DCM (0.12M) TFA (10 equiv) was added. The reaction mixture was stirred at room temperature for 16 hours and concentrated *in vacuo* to give the corresponding deprotected amine as a brown oil that was used without further purification. In a round-bottom flask, a solution of the residue obtained in the previous step and triethylamine (2.0 equiv.) in DCM (0.35M) was prepared and cooled to 0°C. Trifluoroacetic anhydride (TFFA, 1.1 equiv.) was then added dropwise with continuous stirring. An additional 0.5 equiv. of TFFA was added at 0ºC to ensure complete conversion. After that, the reaction mixture was allowed to warm to room temperature and stirred for 4 hours. After completion of the reaction (check by TLC), the mixture was quenched with water and

extracted with DCM (x3). The combined organic layer was washed brine and dried over anhydrous Na_2SO_4, filtered, and concentrated under reduced pressure using a rotary evaporator. The resulting crude product was purified by flash chromatography using hexane/EtOAc mixtures as eluent, yielding the trifluoroacetamide protected amine (***17***) as a colorless liquid (200 mg, 0.61 mmol, 78%). The crude was purified by flash column chromatography using hexane/EtOAc (10:1) as eluent.

^{1}H NMR (300 MHz, $CDCl_3$) δ 3.80 (td, *J* = 13.1, 4.1 Hz, 1H), 3.69 – 3.59 (m, 1H), 3.55 – 3.27 (m, 1H), 3.00 (ddd, *J* = 14.3, 9.9, 6.4 Hz, 1H), 2.27 (dtd, *J* = 10.8, 7.3, 1.9 Hz, 2H), 2.00 – 1.62 (m, 5H), 1.62 – 1.34 (m, 3H), 1.32 – 1.13 (m, 1H). **^{13}C NMR** (75 MHz, $CDCl_3$) δ 156.9 (q, *J* = 35.7 Hz), 116.62 (q, *J* = 287.8 Hz)., 79.6, 79.1, 52.8, 52.2, 48.4, 48.3, 48.3, 39.6, 38.7, 38.2, 36.5, 33.1, 32.8, 32.7, 31.7, 28.9, 25.8, 25.4, 23.7, 17.5, 17.4. ^{19}F NMR (282 MHz, $CDCl_3$) δ -68.68, -68.95. **IR**: 2932, 2861, 1685, 1432, 1275, 1261, 1140, 750, 418. **HRMS** (ESI-TOF) m/z: $[M-C_2H_5]^+$ Calcd. for $C_{10}H_{10}BrF_3NO$ 295.9898; Found: 295.9896.

2-(1,4-dioxaspiro[4.5]decan-7-yl)acetic acid (18-CO_2H)

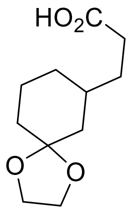

2-(3-Oxocyclohexyl)acetic acid (1.00 g, 6.34 mmol), ethylene glycol (0.76 mL, 113 mmol, 2 equiv) and p-toluenesulfonic acid monohydrate (54.6 mg, 0.315 mmol) were added sequentially to a 2 head 50 mL round-bottom flask, followed by the addition of toluene (20 mL). The mixture was refluxed overnight using a Dean–Stark apparatus to remove the water formed during the reaction. After cooling to room temperature, the mixture was washed with saturated aqueous $NaHCO_3$ (3 × 10 mL). The organic layer was dried over anhydrous Na_2SO_4, filtered, and concentrated under reduced pressure. The crude residue was used in the next step without further purification.

^{1}H NMR (300 MHz, $CDCl_3$) δ 9.79 (bs, 1H), 3.93 (s, 4H), 2.31 – 2.22 (m, 2H), 2.18 – 2.06 (m, 1H), 1.88 – 1.65 (m, 4H), 1.60 – 1.36 (m, 2H), 1.33 – 1.19 (m, 1H), 0.96 (qd, *J* = 12.8, 12.1, 3.5 Hz, 1H). **^{13}C NMR** (75 MHz, $CDCl_3$) δ 178.4, 108.8, 64.3, 64.2, 41.0, 34.6, 32.4, 31.4, 22.8. **HRMS** (ESI-TOF) m/z: [M]+ Calcdd for $C_{11}H_{18}O_4$ 214.1205; Found 214.1206.

2-(1,4-dioxaspiro[4.5]decan-7-yl)ethan-1-ol (18-OH)

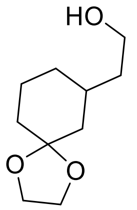

The title compound was synthesized using General Procedure A1, starting from the crude carboxylic acid ***18-CO_2H*** obtained in the previous step, yielding alcohol (***18-OH***) as a colorless liquid. The crude residue was used in the next step without further purification.

^{1}H NMR (300 MHz, $CDCl_3$) δ 3.90 (s, 4H), 3.63 (t, J = 6.8 Hz, 1H), 2.00 (bs, 1H), 1.77 – 1.63 (m, 5H), 1.53 – 1.34 (m, 4H), 1.28 – 1.12 (m, 1H), 0.99 – 0.79 (m, 1H). **^{13}C NMR** (75 MHz, $CDCl_3$) δ 109.2, 64.2, 64.0, 60.4, 41.6, 39.7, 34.7, 32.2, 31.8, 23.0. **HRMS**: non purified.

7-(2-bromoethyl)-1,4-dioxaspiro[4.5]decane (18-Br)

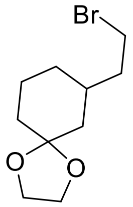

The title compound was synthesized using General Procedure B2, starting from the crude alcohol ***18-OH*** obtained in the previous step, yielding the bromoalkane (***18-Br***) as a colorless liquid (300 mg, 1.2 mmol, 19% for the 3 steps form 2-(3-Oxocyclohexyl)acetic acid). The crude was purified by flash column chromatography using hexane/EtOAc (10:1) as eluent.

^{1}H NMR (300 MHz, $CDCl_3$) δ 3.91 (s, 4H), 3.39 (t, J = 6.9 Hz, 2H), 1.83 – 1.66 (m, 7H), 1.56 – 1.31 (m, 2H), 1.28 – 1.09 (m, 1H), 0.90 – 0.80 (m, 1H). **^{13}C NMR** (75 MHz, $CDCl_3$) δ 108.9, 64.3, 64.1, 41.0, 39.7, 34.7, 34.3, 31.1, 31.0, 22.9. **HRMS** (ESI-TOF) m/z: $[M]^+$ Calcd. for $C_{10}H_{17}BrO_2$ 248.0412; Found: 248.0411

7-(but-3-yn-1-yl)-1,4-dioxaspiro[4.5]decane (18-CCH)

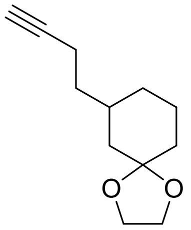

The title compound was synthesized using General Procedure C, starting from 1.2 mmol (300 mg) of the bromoalkene (***18-Br***), yielding the alkyne (***18-CCH***) as a colorless liquid (158 mg, 0.81 mmol, 68%). The crude was purified by flash column chromatography using hexane/EtOAc (10:1) as eluent.

^{1}H NMR (300 MHz, $CDCl_3$) δ 3.90 (s, 4H), 2.17 (td, J = 7.4, 2.6 Hz, 2H), 1.90 (t, J = 2.7 Hz, 1H), 1.78 – 1.61 (m, 5H), 1.49 – 1.38 (m, 4H), 1.15 (d, J = 12.4 Hz, 1H), 0.83 (t, J = 11.2 Hz, 1H). **^{13}C NMR** (75 MHz, $CDCl_3$) δ 109.1, 84.4, 68.2, 64.2, 64.1, 41.2, 35.4, 34.8, 34.6, 31.2, 23.0, 15.8. **IR**: 3303, 2986, 2936, 2888, 1682, 1419, 1264, 1150, 1073, 931, 764, 748, 704, 635. **HRMS** (ESI-TOF) m/z: $[M]^+$ Calcd. for $C_{12}H_{18}O_2$ 194.1307; Found: 194.1309.

7-(4-bromobut-3-yn-1-yl)-1,4-dioxaspiro[4.5]decane (18-acetal)

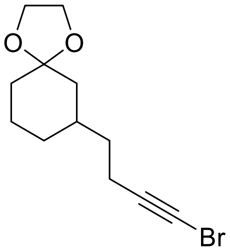

The title compound was synthesized using General Procedure D, starting from 0.81 mmol (158 mg) of the corresponding alkyne (***18-CCH***), yielding the bromoalkyne (***18-acetal***) as a colorless liquid (203 mg, 0.74 mmol, 92%). The crude was purified by flash column chromatography using hexane/EtOAc (10:1) as eluent.
^{1}H NMR (300 MHz, $CDCl_3$) δ 3.90 (s, 4H), 2.18 (t, *J* = 7.5 Hz, 2H), 1.74 – 1.63 (m, 5H), 1.46 – 1.33 (m, 4H), 1.11 (t, *J* = 12.3 Hz, 1H), 0.88 – 0.77 (m, 1H). **^{13}C NMR** (75 MHz, $CDCl_3$) δ 109.0, 80.1, 64.2, 64.1, 41.1, 37.6, 35.2, 34.8, 34.6, 31.2, 23.0, 17.1. **HRMS** (ESI-TOF) m/z: $[M]^+$ Calcd. for $C_{12}H_{17}BrO_2$ 272.0412; Found: 272.0409.

3-(4-bromobut-3-yn-1-yl)cyclohexan-1-one (18)

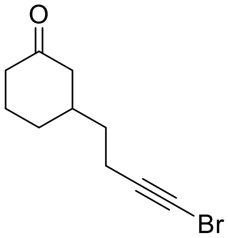

To the solution of ***18-acetal*** (137 mg, 0.5 mmol) in THF (0.25M) at room temperature, an equal volume of a 3M HCl solution was added. The reaction mixture was stirred magnetically for 3 hours. After this time, the reaction was extracted with Et_2O (x3) and the combined organic layers washed with $NaHCO_3$ sat. and brine. The organic layer was dried over Na_2SO_4, filtered and the solvent removed in vacuum. The obtained crude mixture was purified, if necessary, by flash column chromatography on silica gel using hexane/EtOAc (10:1) as eluent to afford **18** as a colorless liquid (80 mg, 0.35 mmol, 70%).

^{1}H NMR (300 MHz, $CDCl_3$) δ 2.48 – 2.29 (m, 2H), 2.30 – 2.18 (m, 3H), 2.09 – 1.84 (m, 4H), 1.77 – 1.59 (m, 1H), 1.55 (dq, *J* = 13.8, 7.1 Hz, 2H), 1.34 (ddd, *J* = 14.8, 8.7, 3.3 Hz, 1H). **^{13}C NMR** (75 MHz, $CDCl_3$) δ 211.1, 79.4, 47.5, 41.3, 38.3, 37.8, 34.7, 30.7, 25.0, 17.1. **IR**: 2930, 2861, 1708, 1418, 1275, 1263, 1091, 912, 764, 748. **HRMS** (ESI-TOF) m/z: $[M]^+$ Calcd. for $C_{10}H_{13}BrO$ 228.0150; Found: 228.0147.

3-(4-bromobut-3-yn-1-yl)-1,1-difluorocyclohexane (19)

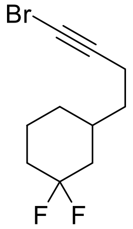

DAST (2 equiv) was added to a solution of **18** (58 mg, 0.25 mmol) in anhydrous DCM (0.50 M) at 0 °C. The reaction mixture was stirred for 18 hours at room temperature (followed by TLC). Then, the reaction was quenched carefully with H_2O, extracted with DCM and finally the combined organic layers washed with brine. The organic layer was dried over Na_2SO_4, filtered and the solvent removed in vacuum. The crude obtained was purified by flash column chromatography on silica gel using n-hexane as eluent to afford **19** as a colorless liquid (30 mg, 0.12 mmol, 48%).

^{1}H NMR (300 MHz, $CDCl_3$) δ 2.24 (t, *J* = 7.4 Hz, 2H), 2.16 – 2.02 (m, 2H), 1.77 (dt, *J* = 7.9, 2.7 Hz, 3H), 1.62 – 1.39 (m, 4H), 1.37 – 1.19 (m, 1H), 1.05 – 0.81 (m, 1H). **^{13}C NMR** (75 MHz, $CDCl_3$) δ 123.6 (t, *J* = 239.4 Hz), 79.6, 39.9 (dd, *J* = 25.4, 21.2 Hz), 38.2, 34.6, 34.0 (dd, *J* = 21.5, 5.4 Hz), 33.8, 30.5, 21.9 (d, *J* = 9.6 Hz), 17.1. **^{19}F NMR** (282 MHz, $CDCl_3$) δ -87.98, -88.82, -99.65, -100.49. **IR**: 3054, 2988, 2310, 1707, 1421, 1275, 1263, 913, 764, 749, 705, 445, 417. **HRMS** (ESI-TOF) m/z: $[M]^+$ Calcd. for $C_{10}H_{13}BrF_2$ 250.0169; Found: 250.0166 .

tert-butyl 4-(4-bromobut-3-yn-1-yl)piperidine-1-carboxylate (20)

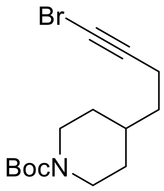

The title compound was synthesized using General Procedure D, starting from 4.1 mmol (973 mg) of the alkyne (**7-9/20-*CCH***), yielding the bromoalkyne (***20***) as a colorless liquid (1.10 g, 3.50 mmol, 85%). The crude was purified by flash column chromatography using hexane/EtOAc (10:1) as eluent.

^{1}H NMR (300 MHz, $CDCl_3$) δ 4.22 – 4.01 (m, 2H), 2.69 (t, *J* = 12.8 Hz, 2H), 2.25 (t, *J* = 7.0 Hz, 2H), 1.46 (s, 13H), 1.26 (t, *J* = 7.1 Hz, 1H), 1.16 – 1.01 (m, 2H).**^{13}C NMR** (75 MHz, $CDCl_3$) δ 154.8, 79.9, 79.2, 44.7, 37.9, 34.8, 34.7, 31.6, 28.4, 16.9. **IR**: 3054, 2979, 2934, 2857, 1683, 1425, 1265, 1165, 743, 706. **HRMS** (ESI-TOF) m/z: $[M+Na]^+$Calcd. for $C_{14}H_{22}BrNNaO_2$ 338.0732; Found: 338.0726.

4.2. Gold-catalyzed reactions

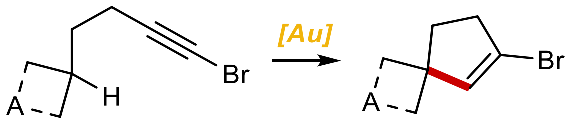

2-bromo-7-oxaspiro[4.5]dec-1-ene (3a)

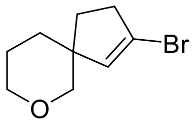

The title compound was synthesized using General Procedure E, starting from 0.20 mmol (43 mg) of the corresponding bromoalkyne (***3***), yielding the bromocyclopentene (***3a***) as

a colorless liquid (8.6 mg, 0.04 mmol, 21%). The crude was purified by flash column chromatography using hexane/EtOAc (10:1) as eluent.
^{1}H NMR (400 MHz, $CDCl_3$) δ 5.80 (t, *J* = 2.1 Hz, 1H), 3.72 – 3.62 (m, 1H), 3.56 (tt, *J* = 9.6, 2.8 Hz, 1H), 3.38 (s, 2H), 2.70 – 2.57 (m, 2H), 1.98 (ddd, *J* = 13.8, 8.0, 6.1 Hz, 1H), 1.79 – 1.51 (m, 5H). **^{13}C NMR** (101 MHz, $CDCl_3$) δ 136.2, 122.1, 75.0, 68.1, 49.7, 38.3, 34.8, 33.5, 23.3. **HRMS** (ESI-TOF) m/z: $[M]^+$ Calcd. for $C_9H_{13}BrO$ 216.0150; Found: 216.0148.

2-bromo-8-oxaspiro[4.5]dec-1-ene (4a)

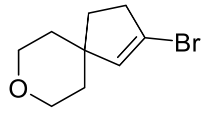

The title compound was synthesized using General Procedure E, starting from 0.20 mmol (43 mg) of the corresponding bromoalkyne (***4***), yielding the bromocyclopentene (***4a***) as a colorless solid with low melting point (30 mg, 0.14 mmol, 68%, 81% gram scale). The crude was purified by flash column chromatography using hexane/EtOAc (10:1) as eluent.
^{1}H NMR (300 MHz, $CDCl_3$) δ 5.85 (s, 1H), 3.90 – 3.45 (m, 4H), 2.76 – 2.55 (m, 2H), 1.87 (t, *J* = 7.3 Hz, 2H), 1.64 (ddd, *J* = 12.4, 8.1, 4.0 Hz, 2H), 1.53 – 1.39 (m, 2H). **^{13}C NMR** (75 MHz, $CDCl_3$) δ 138.0, 121.0, 65.0, 47.2, 38.2, 37.1, 35.7. **IR**: 2927, 2842, 1620, 1235, 1013, 913, 839, 798, 733. **HRMS** (ESI-TOF) m/z: $[M]^+$ Calcd. for $C_9H_{13}BrO$ 216.0150; Found: 216.0146.

10-bromo-1,4-dioxadispiro[4.2.4^{8}.2^{5}]tetradec-9-ene (5a)

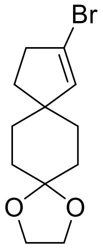

The title compound was synthesized using General Procedure E, starting from 0.20 mmol (55 mg) of the corresponding bromoalkyne (***5***), yielding the bromocyclopentene (***5a***) as a colorless liquid (23 mg, 0.08 mmol, 42%). The crude was purified by flash column chromatography using hexane/EtOAc (10:1) as eluent.
^{1}H NMR (300 MHz, $CDCl_3$) δ 5.86 (t, *J* = 2.0 Hz, 1H), 3.96 (s, 4H), 2.64 (ddd, *J* = 7.5, 6.7, 2.0 Hz, 2H), 1.85 (dd, *J* = 8.0, 6.7 Hz, 2H), 1.79 – 1.50 (m, 8H). **^{13}C NMR** (75 MHz, $CDCl_3$) δ 138.5, 120.4, 108.4, 64.2, 48.8, 38.4, 35.3, 34.5, 32.0. **IR**: 3052, 2929, 2882,

2857, 1711, 1618, 1443, 1265, 1088, 799, 739. **HRMS** (ESI-TOF) m/z: $[M+H]^+$ Calcd. for $C_{12}H_{18}BrO_2$ 273.0490; Found: 273.0485.

2-bromospiro[4.5]dec-1-en-8-one (6a)

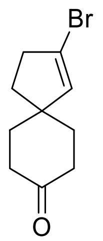

The title compound was synthesized using General Procedure E, starting from 0.2 mmol (46 mg) of the corresponding bromoalkyne (***6***), yielding the bromocyclopentene (***6a***) as a colorless liquid (26 mg, 0.11 mmol, 56%). The crude was purified by flash column chromatography using hexane/EtOAc (10:1) as eluent.

^{1}H NMR (300 MHz, $CDCl_3$) δ 5.92 (d, *J* = 2.0 Hz, 1H), 2.72 (td, *J* = 7.4, 2.0 Hz, 2H), 2.38 (t, *J* = 6.9 Hz, 4H), 2.04 – 1.93 (m, 2H), 1.85 (qt, *J* = 13.4, 6.6 Hz, 4H). **^{13}C NMR** (75 MHz, $CDCl_3$) δ 211.0, 136.8, 121.8, 48.8, 38.5, 38.4, 36.9, 35.1. **IR**: 2966, 2895, 1718, 1146. **HRMS** (ESI-TOF) m/z: $[M+Na]^+$ Calcd. for $C_{10}H_{13}BrNaO$ 251.0047; Found: 251.0042.

1-(2-bromo-8-azaspiro[4.5]dec-1-en-8-yl)-2,2,2-trifluoroethan-1-one (7a)

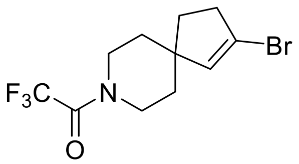

The title compound was synthesized using General Procedure E, starting from 0.2 mmol (62 mg) of the corresponding bromoalkyne (***7***), yielding the bromocyclopentene (***7a***) as a colorless liquid (44 mg, 0.14 mmol, 71%, 83% gram scale). The crude was purified by flash column chromatography using hexane/EtOAc (10:1) as eluent.

^{1}H NMR (300 MHz, $CDCl_3$) δ 5.81 (s, 1H), 3.86 – 3.68 (m, 1H), 3.59 (dd, *J* = 7.0, 3.9 Hz, 1H), 3.48 (td, *J* = 9.0, 4.4 Hz, 2H), 2.74 – 2.56 (m, 2H), 1.88 (td, *J* = 7.5, 1.7 Hz, 2H), 1.70 – 1.46 (m, 4H). **^{13}C NMR** (75 MHz, $CDCl_3$) δ 155.3 (d, *J* = 35.4 Hz), 136.5, 122.2, 116.5 (q, *J* = 288.1 Hz). 48.0, 43.2, 41.0, 38.2, 36.8, 35.8, 35.2. **^{19}F NMR** (282 MHz, $CDCl_3$) δ -68.9. **IR**: 3056, 1689, 1203, 737, 2934, 1464, 1195, 2857, 1266, 1143. **HRMS** (ESI-TOF) m/z: $[M+H]^+$ Calcd. for $C_{11}H_{14}BrFNO$ 312.0211; Found: 312.0205.

benzyl 2-bromo-8-azaspiro[4.5]dec-1-ene-8-carboxylate (8a)

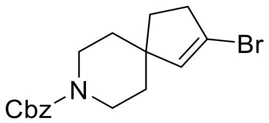

The title compound was synthesized using General Procedure E, starting from 0.1 mmol (35 mg) of the corresponding bromoalkyne (***8***), yielding the bromocyclopentene (***8a***) as

a colorless liquid (14 mg, 44%,). The crude was purified by flash column chromatography using Hexane/EtOAc (5:1) as eluent.

1H NMR (300 MHz, Chloroform-*d*) δ 7.41 – 7.29 (m, 5H), 5.82 (t, *J* = 2.0 Hz, 1H), 5.13 (s, 2H), 3.60 (ddd, *J* = 13.6, 6.8, 4.1 Hz, 2H), 3.38 (ddd, *J* = 13.6, 8.4, 3.8 Hz, 2H), 2.65 (ddd, *J* = 8.1, 6.7, 2.0 Hz, 2H), 1.85 (t, *J* = 7.3 Hz, 2H), 1.64 – 1.40 (m, 4H). **^{13}C NMR** (75 MHz, $CDCl_3$) δ 155.3, 137.4, 136.9, 128.5, 128.0, 127.9, 121.4, 67.0, 48.1, 41.3, 38.3, 36.3, 35.3. **IR**: 2926, 2857, 2321, 1698, 1621, 1429, 1364, 1275, 1234, 1158, 1089, 1011, 963, 797, 764, 750, 698. **HRMS** (ESI-TOF) m/z: $[M]^+$ Calcd. for $C_{17}H_{20}BrNO_2$ 349.0677; Found: 349.0708.

2-bromo-8-tosyl-8-azaspiro[4.5]dec-1-ene (9a)

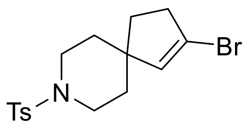

The title compound was synthesized using General Procedure E, starting from 0.20 mmol (62 mg) of the corresponding bromoalkyne (***9***), yielding the bromocyclopentene (***9a***). as a colorless solid (19 mg, 30%). The crude was purified by flash column chromatography using Hexane/EtOAc (5:1) as eluent.

^{1}H NMR (300 MHz, Chloroform-*d*) δ 7.64 (d, *J* = 8.3 Hz, 2H), 7.32 (d, *J* = 8.1 Hz, 2H), 5.68 (t, *J* = 2.0 Hz, 1H), 3.09 (ddd, *J* = 11.4, 7.2, 3.9 Hz, 2H), 2.93 (ddd, *J* = 11.9, 8.1, 3.7 Hz, 2H), 2.58 (ddd, *J* = 7.9, 6.7, 2.0 Hz, 2H), 2.44 (s, 3H), 1.76 – 1.65 (m, 4H), 1.59 (dd, *J* = 7.1, 3.7 Hz, 2H). **^{13}C NMR** (75 MHz, $CDCl_3$) δ 143.5, 136.9, 133.3, 129.7, 127.7, 121.7, 47.4, 43.5, 38.2, 35.9, 35.4, 21.5. **IR**: 3054, 2988, 2344, 2312, 1688, 1507, 1424, 1264, 1159, 913, 764, 748, 583. **HRMS** (ESI-TOF) m/z: $[M]^+$ Calcd. for $C_{16}H_{20}BrNO_2S$ 369.0398; Found: 369.0428. **m.p.** = 155-158.

2-bromo-8,8-difluorospiro[4.5]dec-1-ene (10a)

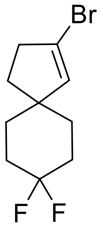

The title compound was synthesized using General Procedure E, starting from 0.20 mmol (50 mg) of the corresponding bromoalkyne (***10***), yielding the bromocyclopentene (***10a***) as a colorless liquid (25 mg, 0.10 mmol, 50%). The crude was purified by flash column chromatography using n-hexane as eluent.

^{1}H NMR (300 MHz, $CDCl_3$) δ 5.82 (t, *J* = 2.0 Hz, 1H), 2.66 (ddd, *J* = 7.5, 6.8, 2.0 Hz, 2H), 2.01 – 1.83 (m, 6H), 1.65 (ddt, *J* = 25.7, 13.7, 6.8 Hz, 4H). **^{13}C NMR** (75 MHz, $CDCl_3$) δ 137.2, 123.1 (t, *J* = 241.0 Hz), 121.4, 48.4, 38.4, 35.0, 33.3 (t, *J* = 5.0 Hz), 31.1 (*J* = 24.2 Hz), 29.7. **^{19}F NMR** (282 MHz, $CDCl_3$) δ -91.69 (d, *J* = 235.5 Hz), -

102.15 (dd, J = 237.6, 34.0 Hz). **IR**: 2932, 2861, 1734, 1623, 1265, 1122, 1079, 984, 738. **HRMS** (ESI-TOF) m/z: $[M]^+$ Calcd. for $C_{10}H_{13}BrF_2$ 250.0169; Found: 250.0164.

2-bromo-8-thiaspiro[4.5]dec-1-ene 8,8-dioxide (11a)

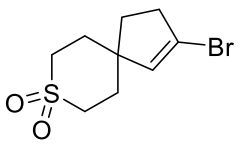

The title compound was synthesized using General Procedure E, starting from 0.20 mmol (53 mg) of the corresponding bromoalkyne (***11***), yielding the bromocyclopentene (***11a***) (13%, NMR yield using CH_2Br_2 as internal standard).

2-bromo-8,8-dimethylspiro[4.5]dec-1-ene (12a)

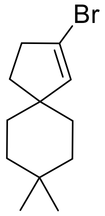

The title compound was synthesized using General Procedure E, starting from 0.20 mmol (49 mg) of the corresponding bromoalkyne (***12***), yielding the bromocyclopentene (***12a***) as a colorless liquid (37 mg, 0.14 mmol, 71%). The crude was purified by flash column chromatography using n-hexane as eluent.
^{1}H NMR (300 MHz, $CDCl_3$) δ 5.84 (s, 1H), 2.59 (ddd, J = 8.0, 6.9, 2.0 Hz, 2H), 1.77 (dd, J = 8.0, 6.8 Hz, 2H), 1.56 – 1.45 (m, 2H), 1.36 (dd, J = 12.6, 5.4 Hz, 2H), 1.27 (dd, J = 6.9, 5.0 Hz, 4H), 0.90 (s, 6H). **^{13}C NMR** (75 MHz, $CDCl_3$) δ 139.4, 119.7, 49.5, 38.3, 36.0, 33.2, 29.5. **HRMS** (ESI-TOF) m/z: $[M]^+$ Calcd. for $C_{12}H_{19}Br$ 242.0670; Found: 242.0665.

2-bromo-8-oxaspiro[4.6]undec-1-ene (13a)

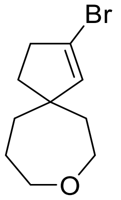

The title compound was synthesized using General Procedure E, starting from 0.20 mmol (46 mg) of the corresponding bromoalkyne (***13***), yielding the bromocyclopentene (***13a***) as a colorless liquid (16 mg, 0.07 mmol, 32%). The crude was purified by flash column chromatography using hexane/EtOAc (10:1) as eluent.
^{1}H NMR (300 MHz, $CDCl_3$) δ 5.91 (s, 1H), 3.68 (dt, J = 18.0, 5.4 Hz, 4H), 2.60 (td, J = 7.3, 2.0 Hz, 2H), 1.83 (t, J = 7.2 Hz, 2H), 1.75 – 1.53 (m, 6H). **^{13}C NMR** (75 MHz, $CDCl_3$) δ 139.1, 120.3, 69.5, 65.8, 51.9, 41.6, 38.3, 38.3, 37.7, 26.4. **HRMS** (ESI-TOF) m/z: $[M]^+$ Calcd. for $C_{10}H_{15}BrO$ 230.0306; Found: 230.0296.

2-bromo-8-oxaspiro[4.6]undec-1-en-9-one (14a)

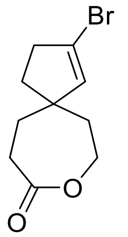

The title compound was synthesized using General Procedure E, starting from 0.20 mmol (49 mg) of the corresponding bromoalkyne (***14***), yielding the bromocyclopentene (***14a***) as a colorless liquid (37 mg, 0.15 mmol, 76%). The crude was purified by flash column chromatography using hexane/EtOAc (10:1) as eluent.

^{1}H NMR (300 MHz, $CDCl_3$) δ 5.85 (s, 1H), 4.23 (dd, *J* = 5.4, 4.1 Hz, 2H), 2.82 – 2.56 (m, 4H), 1.96 – 1.71 (m, 6H). **^{13}C NMR** (75 MHz, $CDCl_3$) δ 175.5, 136.8, 122.5, 65.1, 51.2, 40.0, 38.2, 36.1, 33.7, 30.5. **IR**: 2932, 2255, 1729, 1623, 1074, 912, 733, 651. **HRMS** (ESI-TOF) m/z: $[M+H]^+$ Calcdd for $C_{10}H_{14}BrO_2$ 245.0177; Found: 245.0172.

8-bromo-2,2-difluorodispiro[3.1.46.14]undec-7-ene (15a)

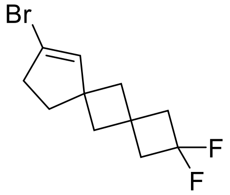

The title compound was synthesized using General Procedure E, starting from 0.20 mmol (53 mg) of the corresponding bromoalkyne (***15***), yielding the bromocyclopentene (***15a***) as a colorless liquid (39 mg, 0.15 mmol, 74%). The crude was purified by flash column chromatography using n-hexane as eluent.

^{1}H NMR (300 MHz, $CDCl_3$) δ 5.81 (s, 1H), 2.67 – 2.45 (m, 6H), 2.27 (d, *J* = 11.8 Hz, 2H), 2.20 – 1.98 (m, 4H). **^{13}C NMR** (75 MHz, $CDCl_3$) δ 138.3, 120.9, 119.5 (t, *J* = 279.7 Hz), 47.8 (t, *J* = 21.7 Hz), 47.2 (t, *J* = 21.7 Hz), 46.3, 38.9, 38.8, 27.1 (t, *J* = 8.9 Hz). **^{19}F NMR** (282 MHz, $CDCl_3$) δ -90.83. **HRMS** (ESI-TOF) m/z: $[M]^+$ Calcd. for $C_{11}H_{13}BrF_2$ 262.0169; Found: 262.0171.

14-bromo-10-oxadispiro[4.1.57.25]tetradec-13-ene (16a)

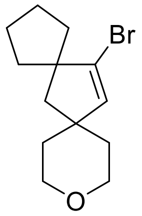

The title compound was synthesized using General Procedure E, starting from 0.20 mmol (54 mg) of the corresponding bromoalkyne (***16***), yielding the bromocyclopentene (***16a***) as a colorless liquid (28 mg, 0.10 mmol, 46%). The crude was purified by flash column chromatography using n-hexane as eluent.

^{1}H NMR (300 MHz, $CDCl_3$) δ 5.85 (s, 1H), 3.86 – 3.52 (m, 4H), 2.02 – 1.78 (m, 3H), 1.79 – 1.54 (m, 6H), 1.54 – 1.32 (m, 4H). **^{13}C NMR** (75 MHz, $CDCl_3$) δ 136.7, 132.1, 65.1, 58.1, 50.7, 45.7, 39.5, 38.8, 25.0. **HRMS** (ESI-TOF) m/z: $[M]^+$ Calcd. for $C_{13}H_{19}BrO$ 270.0619; Found: 270.0617.

1-(2-bromo-7-azaspiro[4.6]undec-1-en-7-yl)-2,2,2-trifluoroethan-1-one (17a)

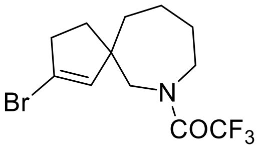

The title compound was synthesized using General Procedure E, starting from 0.1 mmol (33 mg) of the corresponding bromoalkyne (***17***), yielding the bromocyclopentene (***17a***) as a colorless liquid (13,9 mg, 0.042 mmol, 42%). The crude was purified by flash column chromatography using hexane/EtOAc (10:1) as eluent.

^{1}H NMR (300 MHz, $CDCl_3$) δ 5.80 (t, J = 2.0 Hz, 1H), 3.68 – 3.37 (m, 4H), 2.69 – 2.52 (m, 2H), 2.12 – 1.97 (m, 1H), 1.78 (tt, J = 9.7, 4.9 Hz, 4H), 1.71 – 1.59 (m, 3H). **^{13}C NMR** (101 MHz, $CDCl_3$) δ 157.34 (q, J = 35.6 Hz), 116.71 (q, J = 287.7 Hz), 137.3, 122.0, 116.71 (q, J = 287.7 Hz), 56.2, 53.7, 50.6, 50.6, 38.2, 38.0, 35.7, 29.6, 22.2. **^{19}F NMR** (282 MHz, $CDCl_3$) δ -67.31, -68.95. **IR**: 3735, 3005, 2989, 2319, 1687, 1507, 1458, 1275, 1261, 1159, 911, 749. **HRMS** (ESI-TOF) m/z: $[M]^+$ Calcd. for $C_{12}H_{15}BrF_3NO$ 325.0289; Found: 325.0297.

2-bromospiro[4.5]dec-1-en-7-one (18a)

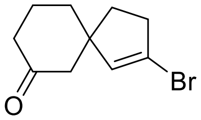

The title compound was synthesized using General Procedure E, starting from 0.1 mmol (23 mg) of the corresponding bromoalkyne (***18***), yielding the bromocyclopentene (***18a***) as a colorless liquid (7.4 mg, 0.032 mmol, 32%). The crude was purified by flash column chromatography using hexane/EtOAc (10:1) as eluent.

^{1}H NMR (300 MHz, $CDCl_3$) δ 5.72 (t, J = 2.0 Hz, 2H), 2.64 (tdd, J = 7.0, 5.5, 2.0 Hz, 2H), 2.41 – 2.21 (m, 4H), 2.06 – 1.75 (m, 5H), 1.76 – 1.62 (m, 1H). **^{13}C NMR** (101 MHz, $CDCl_3$) δ 210.2, 137.1, 122.0, 53.8, 52.1, 41.0, 38.5, 36.1, 35.7, 22.9. **IR**: 3052, 2937, 2859, 1705, 1622, 1421, 1275, 1263, 910, 764, 749, 419, 410. **HRMS** (ESI-TOF) m/z: $[M]^+$ Calcd. for $C_{10}H_{13}BrO$ 228.0150; Found: 228.0150.

2-bromo-7,7-difluorospiro[4.5]dec-1-ene (19a)

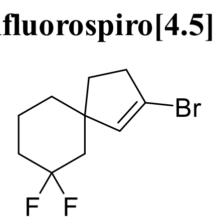

The title compound was synthesized using General Procedure E, starting from 0.1 mmol (25 mg) of the corresponding bromoalkyne (***19***), yielding the bromocyclopentene (***19a***)

as a colorless liquid (8.3 mg, 0.066 mmol, 66%). The crude was purified by flash column chromatography using n-hexane as eluent.

^{1}**H NMR** (300 MHz, Chloroform-*d*) δ 5.83 (s, 1H), 2.62 (ddd, *J* = 8.4, 5.7, 2.0 Hz, 2H), 1.98 – 1.77 (m, 6H), 1.70 (td, *J* = 6.8, 4.9 Hz, 2H), 1.57 – 1.37 (m, 2H). ^{13}**C NMR** (75 MHz, $CDCl_3$) δ 138.0, 123.6 (t, *J* = 241.3 Hz), 121.9, 49.9 (t, *J* = 4.4 Hz), 43.9 (t, *J* = 22.2 Hz), 38.5, 36.9, 35.9, 33.7 (t, *J* = 23.7 Hz), 19.8 (t, *J* = 5.0 Hz). ^{19}**F NMR** (282 MHz, $CDCl_3$) δ -90.19, -91.04, -91.52, -92.38. **IR**: 3009, 2989, 2358, 2308, 1600, 1416, 1275, 1263, 911, 764, 749, 472, 418, 411. **HRMS** (ESI-TOF) m/z: $[M]^+$ Calcd. for $C_{10}H_{13}BrF_2$ 250.0169; Found: 250.0173.

(*Z*)-1-bromo-4-(1-(tert-butoxycarbonyl)piperidin-4-yl)but-1-en-2-yl 4-(4-bromobut-3-yn-1-yl)piperidine-1-carboxylate (20b)

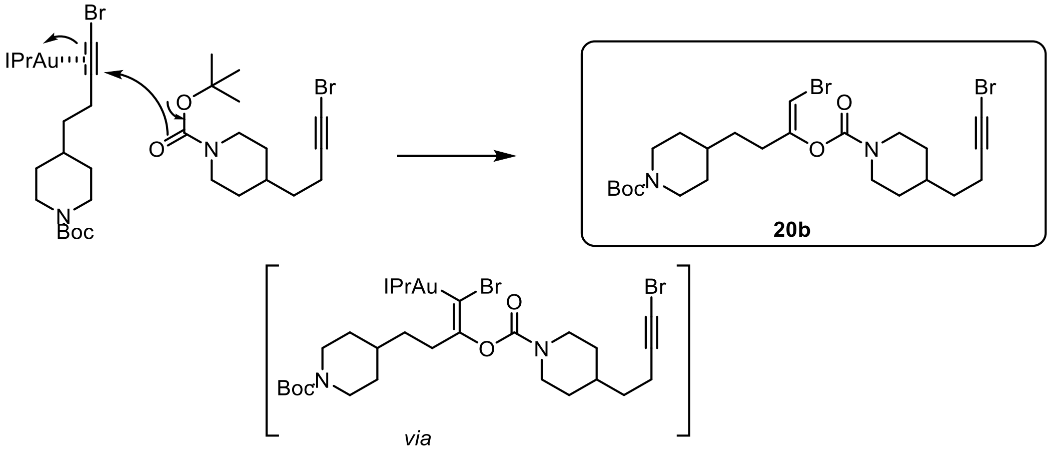

The title compound was synthesized using General Procedure E, starting from 0.20 mmol (63 mg) of the corresponding bromoalkyne (***20***), yielding the dimer (***20b***) as a colorless liquid (10 mg, 0.02 mmol, 18%). The crude was purified by flash column chromatography using hexane/EtOAc (10:1) as eluent.

^{1}**H NMR** (300 MHz, $CDCl_3$) δ 5.76 (s, 1H), 4.23 – 3.97 (m, 4H), 2.76 (dt, *J* = 57.5, 14.8 Hz, 4H), 2.31 (dt, *J* = 27.0, 7.1 Hz, 3H), 1.84 – 1.69 (m, 2H), 1.63 (d, *J* = 13.1 Hz, 4H), 1.50 (t, *J* = 7.0 Hz, 2H), 1.45 (s, 12H), 1.34 – 1.00 (m, 4H). ^{13}**C NMR** (75 MHz, $CDCl_3$) δ 154.8, 153.2, 151.4, 93.4, 79.7, 79.3, 77.2, 44.9, 44.4, 43.9, 35.4, 34.6, 34.6, 32.8, 31.9, 31.1, 28.4, 16.9. **HRMS**: unstable.

4.3. Derivatizations

8-oxaspiro[4.5]dec-1-ene-2-carboxylic acid (21a-C=C)

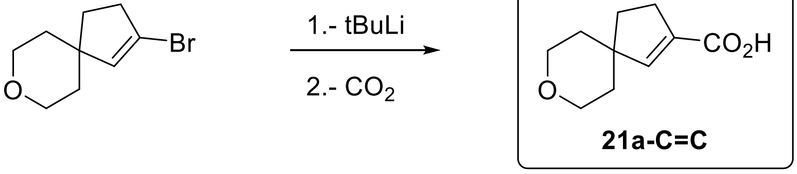

A solution of **4a** (217 mg, 1.00 mmol) in THF (0.10 M) was added to a Schlenk flask under an inert atmosphere and cooled to -78°C using a cryostat. Once this temperature was reached, ^{t}BuLi (1.7 M in THF, 2.1 equiv.) was added dropwise very carefully, and

the mixture was stirred for one hour. After that time, CO_2 was bubbled through the reaction mixture for fifteen minutes, then the mixture was allowed to warm to room temperature. The reaction was then quenched with a saturated Na_2CO_3 solution and was extracted three times with the same aqueous solution. The resulting aqueous phase was acidified with HCl (2 M), followed by three extractions with EtOAc. The organic phases were combined, dried over anhydrous Na_2SO_4, filtered, and the solvents were removed under reduced pressure. The corresponding acid **21a-C=C** was obtained in sufficient purity to be used in the next step without further purification (173 mg, 0.95 mmol, 95%). **^{1}H NMR** (300 MHz, $CDCl_3$) δ 9.67 (s, 1H), 6.81 (t, J = 1.9 Hz, 1H), 3.95 – 3.41 (m, 4H), 2.61 (ddd, J = 7.8, 6.7, 1.9 Hz, 2H), 1.91 (t, J = 7.3 Hz, 2H), 1.80 – 1.69 (m, 2H), 1.58 – 1.39 (m, 2H). **^{13}C NMR** (75 MHz, $CDCl_3$) δ 170.4, 152.0, 134.0, 64.9, 47.9, 36.1), 35.1, 29.4. **IR**: 3054, 2945, 2918, 2868, 1703, 1698, 1265, 740. **Mp=** 118-121 ºC. **HRMS** (ESI-TOF) m/z: $[M+H]^+$ Calcd. for $C_{10}H_{15}O_3$ 183.1021; Found: 183.1016.

8-oxaspiro[4.5]decane-2-carboxylic acid (21a)

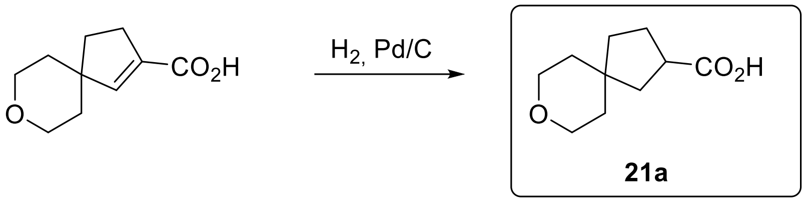

A round bottom flask with a magnetic stir bar and Pd/C (10% w/w) was evacuated to vacuum and filled with Ar. A solution of **21a-C=C** (547 mg, 3 mmol) in AcOEt (0.50 M) was added and hydrogen was bubbled for 15 minutes. The reaction mixture was maintained on a hydrogen atmosphere and stirred overnight at room temperature. The day after, the solids were filtered through a pad of celite. Removal of solvents under vacuum afforded **21a** (525 mg, 2.85 mmol, 95%) in sufficient purity to be used in the next step without further purification.

^{1}H NMR (300 MHz, $CDCl_3$) δ 3.65 (td, J = 5.3, 2.3 Hz, 4H), 2.90 (p, J = 8.5 Hz, 1H), 2.10 – 1.84 (m, 3H), 1.82 – 1.40 (m, 7H). **^{13}C NMR** (75 MHz, $CDCl_3$) δ 182.0, 65.5, 65.2, 42.5, 41.2, 40.7, 38.1, 37.6, 37.5, 28.0. **IR**: 3086, 2953, 2935, 2861, 2249, 1703, 1229, 1104, 912, 736. **Mp=** 78-80 ºC. **HRMS** (ESI-TOF) m/z: $[M+H]^+$ Calcd. for $C_{10}H_{17}O_3$ 185.1178; Found: 185.1172.

(8-oxaspiro[4.5]decan-2-yl)methanol (22a)

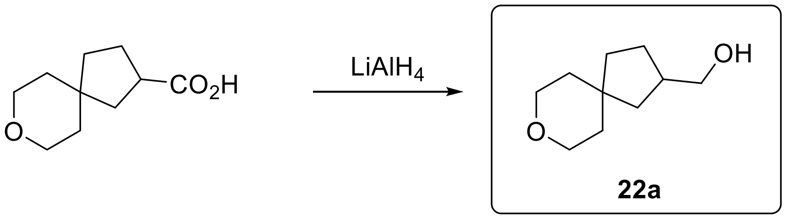

The title compound was synthesized using General Procedure 1A, starting from 0.20 mmol (37 mg) of the carboxylic acid (***21a***), yielding the crude alcohol (***22a***) as a colorless liquid (22 mg, 0.12 mmol, 60%).

^{1}H NMR (300 MHz, $CDCl_3$) δ 3.64 (q, *J* = 5.3 Hz, 4H), 3.53 (d, *J* = 6.8 Hz, 2H), 2.21 (dddd, *J* = 14.7, 9.4, 6.5, 1.4 Hz, 1H), 1.80 (dt, *J* = 13.2, 7.6 Hz, 2H), 1.64 (s, 1H), 1.59 – 1.41 (m, 6H), 1.35 (dd, *J* = 12.5, 8.0 Hz, 1H), 1.10 (dd, *J* = 12.9, 9.3 Hz, 1H). **^{13}C NMR** (75 MHz, $CDCl_3$) δ 67.5, 65.7, 65.3, 41.4, 40.9, 40.3, 39.1, 37.9, 37.6, 27.4. **IR**: 3381, 2932, 2859, 2247, 1105, 912, 738, 649. **HRMS** (ESI-TOF) m/z: $[M]^+$ Calcd. for $C_{10}H_{18}O_2$ 170.1307; Found: 170.1304.

8-oxaspiro[4.5]decane-2-carboxamide (21a-NH_2)

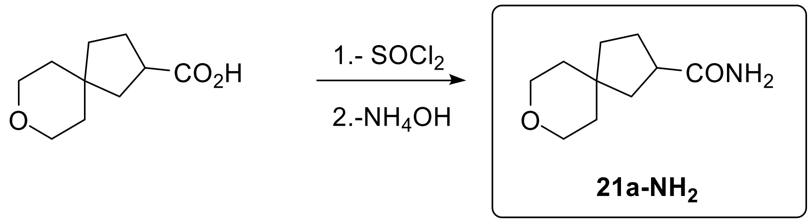

A solution of **21a** (276 mg, 1.5 mmol) and DMF (4.5 equiv.) and $CHCl_3$ (0.30 M) was prepared. Thionyl chloride (5 equiv.) was then added dropwise, and the mixture was cooled to 0°C. This solution was then added to another solution of NH_4OH (30% NH_3 in water, 3 equiv.), which was also maintained at 0°C. The reaction mixture was stirred at this temperature for 10 minutes and then allowed to warm to room temperature under continuous stirring for 1 hour. After completion of the reaction, the mixture was extracted three times with $CHCl_3$. The combined organic phases were dried over anhydrous Na_2SO_4, filtered, and the solvents were removed under reduced pressure. The crude product ***21a-NH$_2$*** was obtained as a colorless oil (272 mg, 1.50 mmol, 99%) and used in the next step without further purification.

^{1}H NMR (300 MHz, $CDCl_3$) δ: 5.93 (s, 1H), 5.64 (s, 1H), 3.65 – 3.60 (m, 4H), 2.72 (q, J = 8.6 Hz, 1H), 1.97 – 1.81 (m, 3H), 1.74 – 1.43 (m, 7H). **^{13}C NMR** (75 MHz, $CDCl_3$) δ: 178.5, 67.2, 65.7, 65.3, 44.0, 42.0, 40.9, 38.5, 37.9, 28.7. **HRMS** (ESI-TOF) m/z: $[M+H]^+$ Calcd. for $C_{10}H_{18}NO_2$ 184.1338; Found: 184.1332.

((8-oxaspiro[4.5]decan-2-yl)methyl)chloro-l5-azane (23a)

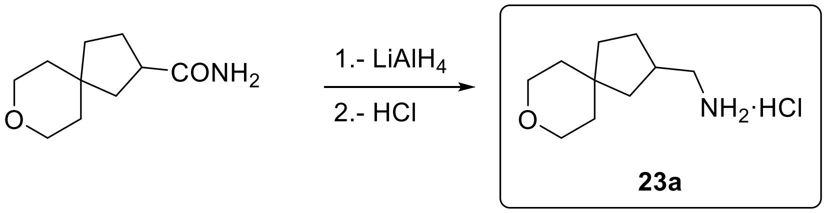

At 0 ºC solution of ***21-NH$_2$*** (272 mg, 1.50 mmol) in dry THF (1.00 M) was added dropwise to a suspension of $LiAlH_4$ (1.6 equiv.) in dry THF (0.40 M). After the addition was complete the reaction mixture was stirred for 1-3 hours at room temperature. Then, the reaction was carefully quenched by dropwise addition of water (1.0 mL per gram of LAH), NaOH 15% (1.0 mL per gram of LAH) and water (3.0 mL per gram of LAH) at 0 ºC. Solids were filtered through alternate pads of celite /silica gel/ celite and the solvents removed by rotary evaporation. The oily substance was dissolved in methyl tert-butyl ether (1.80 M), and the solution was cooled down to −10 °C. 15% Hydrogen chloride in Et_2O solution (0.85 equiv. to basic amine) was added dropwise. The precipitate was

filtered, and the solid was washed with cold diethyl ether yielding the amine hydrochloride ***23a*** (160 mg, 52%).

^{1}H NMR (300 MHz, $CDCl_3$) δ: 8.29 (s, 3H), 3.64 – 3.58 (m, 4H), 3.00 – 2.85 (m, 2H), 2.40 (sp, J = 7.8 Hz, 1H), 2.04 – 1.85 (m, 3H), 1.72 – 1.35 (m, 9H), 1.13 (dd, J = 13.0, 9.7 Hz, 1H). **^{13}C NMR** (75 MHz, $CDCl_3$) δ: 65.5, 65.2, 44.9, 42.9, 40.5, 39.0, 38.0, 37.3, 36.8, 29.1. **HRMS** (ESI-TOF) m/z: [M]+ Calcdd for $C_{10}H_{20}ClNO$ 205.1233; Found 205.1225.

8-oxaspiro[4.5]decan-2-amine hydrochloride (24a-NH$_2$)

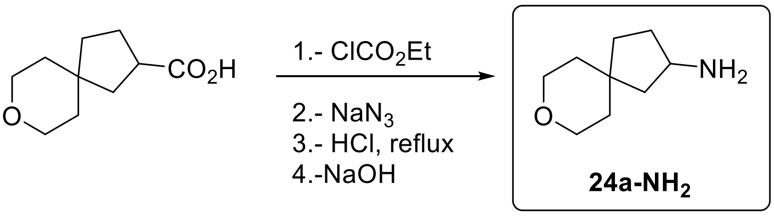

21a (37 mg, 0.2 mmol) was dissolved in dry acetone (0.90 M) along with triethylamine (1.5 equiv.) in a two-neck flask under an inert atmosphere at 0°C. Once the solution was formed, ethyl chloroformate (1.5 equiv.), previously dissolved in dry acetone (0.90 M), was added, and the reaction mixture was stirred for 30 minutes. After this time, a solution of sodium azide (1.3 equiv.) in distilled water (1.25 M) was added, and the reaction mixture was kept at 0°C under stirring for 3 hours. The reaction mixture was then poured into a beaker containing ice and extracted twice with $CHCl_3$ (6.0 mL per mmol of acid). The combined organic phases were collected, and 2M HCl (2.0 mL per mmol of acid) was added. The mixture was then refluxed for 12 hours. After completion of the reaction, the mixture was extracted three times with 1.00 M HCl, and the combined aqueous phases were basified with 1M NaOH. The product was then extracted with $CHCl_3$ (x3), and the combined organic phases were washed with a saturated NaCl solution. The organic layer was dried over anhydrous Na_2SO_4, filtered, and the solvents were removed under reduced pressure to obtain the crude product as a yellow oil (31 mg, 0.20 mmol, 99%).

^{1}H NMR (300 MHz, $CDCl_3$) δ 4.05 – 3.38 (m, 5H), 2.30 – 2.10 (m, 2H), 1.96 – 1.74 (m, 2H), 1.71 – 1.58 (m, 4H), 1.45 (dt, *J* = 6.2, 3.2 Hz, 2H). **^{13}C NMR** (75 MHz, $CDCl_3$) δ 65.4, 64.9, 51.4, 43.1, 40.1, 38.7, 37.8, 35.8, 30.0. **HRMS** (ESI-TOF) m/z: $[M+Na]^+$ Calcd. for $C_9H_{17}NNaO$ 178.1208; Found: 178.1202.

8-oxaspiro[4.5]decan-2-amine hydrochloride (24a)

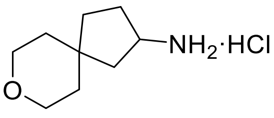

24a-NH$_2$ (31 mg, 0.20 mmol) was dissolved in tert-butyl methyl ether (1.80 M), and the solution was cooled down to −10 °C. 15% Hydrogen chloride in Et_2O solution (0.85 equiv. to basic amine) was added dropwise. The precipitate was filtered, and the solid was washed with cold diethyl ether yielding the corresponding hydrochloride (***24a***) as a colorless solid (34 mg, 0.18 mmol, 90%).

^{1}H NMR (300 MHz, $CDCl_3$): δ 8.30 (s, 3H), 3.72 – 3.50 (m, 5H), 2.22 – 2.11 (m, 2H), 1.92 – 1.76 (m, 2H), 1.68 – 1.54 (m, 4H), 1.48 – 1.32 (m, 2H). **^{13}C NMR** (75 MHz,

$CDCl_3$) δ: 65.3, 64.8, 51.3, 42.9, 40. , 38.6, 37.7, 35.7, 29.8. **HRMS** (ESI-TOF) m/z: [M-Cl]$^+$ Calcd. for $C_9H_{18}NO$156.1388; Found: 156.1383.

2-(8-oxaspiro[4.5]decan-2-yl)acetic acid (25a)

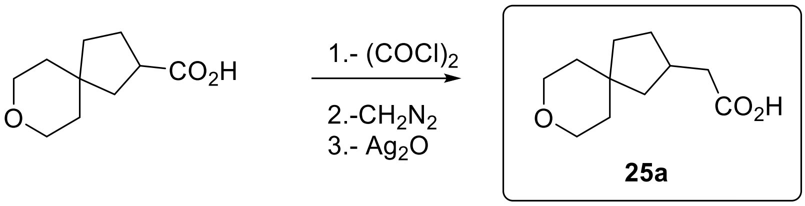

Oxalyl chloride (2 equiv.) is added to a round-bottom flask containing **21a** (92 mg, 0.50 mmol). The reaction mixture was stirred at room temperature for 40 minutes. Meanwhile, in an Erlenmeyer flask, KOH (45 equiv.) was dissolved in H_2O (6.4 mL per mmol of substrate), obtaining a 40% w/v solution. To this solution, Et_2O (6.8 mL per mmol of substrate) was added and the resulting biphasic mixture was cooled down to 0°C. N-nitroso-N-methylurea (9.0 equiv.) was then added portion wise carefully with vigorous stirring. The two phases were separated using an unground extraction funnel, and the organic phase dried over KOH. This solution was then carefully transferred to a Schlenk flask under inert atmosphere, where the preformed acid chloride was added dropwise. The reaction mixture was stirred until nitrogen bubbling ceased, after which acetic acid was added to quench the remaining diazomethane. All remaining phases involving diazomethane and glassware were carefully washed with acetic acid. Solvents were then removed under reduced pressure. The crude product was redissolved in dioxane (0.25 M):H_2O (1 M) and Ag_2O (0.01 equiv.) was added. The mixture was refluxed for 10 minutes and filtered through a Celite pad. Solvents were removed under reduced pressure to obtain the crude product. The product was purified by acid/base extraction yielding ***25a*** as a yellowish liquid (42 mg, 0.21 mmol, 42%).

^{1}H NMR (300 MHz, $CDCl_3$) δ 9.19 (s, 1H), 3.64 (p, J = 5.6 Hz, 4H), 2.35 (d, J = 3.0 Hz, 2H), 1.90 (dq, J = 12.3, 6.4, 5.2 Hz, 3H), 1.52 (dq, J = 21.3, 5.9, 5.1 Hz, 6H), 1.27 (ddt, J = 16.3, 13.5, 8.0 Hz, 1H), 1.05 (m, 1H). **^{13}C NMR** (75 MHz, $CDCl_3$) δ 178.5, 65.5, 65.3, 45.0, 40.2, 40.1, 39.4, 38.3, 37.6, 34.9, 31.1. **HRMS** (ESI-TOF) m/z: [M]$^+$ Calcd. for $C_{11}H_{18}O_3$ 198.1256; Found: 198.1249.

4,4,5,5-tetramethyl-2-(8-oxaspiro[4.5]dec-1-en-2-yl)-1,3,2-dioxaborolane (26a)

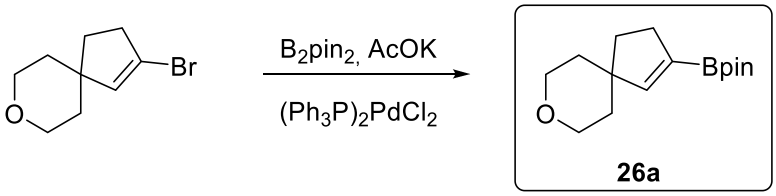

4a (182 mg, 1.00 mmol) was dissolved in dioxane (0.30 M) in a two-neck flask under an argon atmosphere. $(Ph_3P)_2PdCl_2$ (0.03 equiv.), B_2Pin_2 (2.0 equiv.), and KOAc (2.0 equiv.) were then added, and the reaction mixture was stirred at 90°C for 6 hours, monitoring the progress by TLC. Upon completion of the reaction, the mixture was filtered and then centrifuged to collect the supernatant. The solvent was evaporated, and the crude product

26a was purified by flash column chromatography using hexane/EtOAc (5:1) as eluent yielding the borolane derivative ***26a*** as a colorless solid (225 mg, 0.85 mmol, 85%).
^{1}H NMR (300 MHz, $CDCl_3$) δ 6.40 (t, *J* = 2.0 Hz, 1H), 3.75 (ddd, *J* = 11.5, 5.8, 4.0 Hz, 2H), 3.59 (ddd, *J* = 11.6, 8.7, 3.1 Hz, 2H), 2.45 (td, *J* = 7.2, 2.0 Hz, 2H), 1.82 – 1.70 (m, 2H), 1.64 (ddd, *J* = 13.0, 8.7, 4.0 Hz, 2H), 1.46 – 1.33 (m, 2H), 1.26 (s, 12H). **^{13}C NMR** (75 MHz, $CDCl_3$) δ 154.0, 83.4, 65.5, 48.8, 36.5, 36.0, 32.9, 24.8. **IR**: 2979, 2934, 2850, 2247, 1618, 1373, 1144, 1124, 912, 849, 738. **Mp=** 78-80 ºC. **HRMS** (ESI-TOF) m/z: $[M+H]^+$ Calcd. for $C_{15}H_{26}BO_3$ 265.1975; Found: 265.1970.

4,4,5,5-tetramethyl-2-(8-oxaspiro[4.5]decan-2-yl)-1,3,2-dioxaborolane (27a)

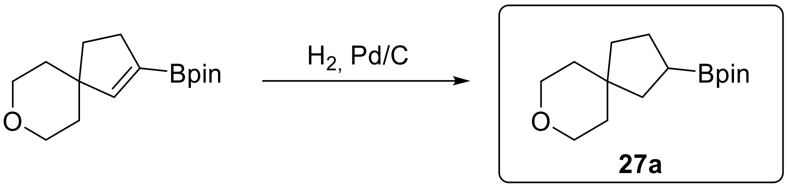

A round bottom flask with a magnetic stir bar and Pd/C (10% w/w) was evacuated to vacuum and filled with Ar. **26a** (66 mg, 0.25 mmol) was dissolved in AcOEt (0.50 M) was added and hydrogen was bubbled for 15 minutes. The reaction mixture was maintained on a hydrogen atmosphere and stirred overnight at room temperature. The day after, the solids were filtered through a pad of celite. Removal of solvents under vacuum followed by flash column chromatography using hexane/EtOAc (5:1) yielded the saturated borolane ***27a*** as a colorless solid (67 mg, 0.25 mmol, 99%).
^{1}H NMR (300 MHz, $CDCl_3$) δ 3.65 – 3.55 (m, 4H), 1.82 – 1.49 (m, 3H), 1.42 – 1.25 (m, 8H), 1.20 (s, 12H). **^{13}C NMR** (75 MHz, $CDCl_3$) δ 82.9, 65.8, 65.5, 41.1, 40.5, 39.0, 38.4, 37.6, 26.5, 24.7. **Mp=** 58-61 ºC. **HRMS** (ESI-TOF) m/z: $[M+H]^+$ Calcd. for $C_{15}H_{28}BO_3$ 267.2132; Found: 267.2126.

8-oxaspiro[4.5]decan-2-one (28a)

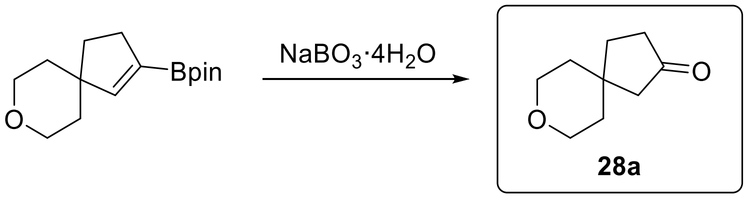

$NaBO_3·4H_2O$ (5 equiv.) was added to a solution of **26a** (106 mg, 0.40 mmol) in a THF/H_2O mixture (0.30 M). The reaction mixture was stirred at room temperature for 2 hours. After that time, the reaction was diluted with water. The mixture was then extracted times with EtOAc (x3), and the combined organic phases were washed with water. The organic layer was dried over anhydrous Na_2SO_4, filtered, and the solvents were removed under reduced pressure to obtain the crude product. The obtained crude mixture was purified by flash column chromatography on silica gel using hexane/EtOAc (3:1) as eluent to afford ketone ***28a*** as a colorless liquid (32 mg, 0.21 mmol, 52%).
^{1}H NMR (300 MHz, $CDCl_3$) δ 3.76 (ddd, *J* = 9.7, 5.6, 2.9 Hz, 2H), 3.55 (ddd, *J* = 11.9, 8.4, 3.4 Hz, 2H), 2.27 (t, *J* = 7.9 Hz, 2H), 2.18 (s, 2H), 1.87 (t, *J* = 7.9 Hz, 2H), 1.73 – 1.47 (m, 4H). **^{13}C NMR** (75 MHz, $CDCl_3$) δ 218.6, 64.7, 49.9, 37.7, 36.9, 35.9, 34.5. **IR**:

3054, 2961, 2925, 2852, 1739, 1266, 1107, 738. **HRMS** (ESI-TOF) m/z: $[M+H]^+$ Calcd. for $C_9H_{15}O_2$ 155.1072; Found: 155.1067.

2-(trifluoromethyl)-8-oxaspiro[4.5]decan-2-ol (29a)

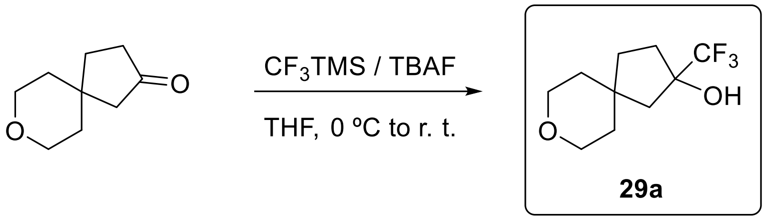

A mixture **28a** (27 mg, 0.18 mmol) and $TMSCF_3$ (12.0 equiv.) in THF (0.36 M) was cooled to 0 °C in an ice bath and treated with tetrabutylammonium fluoride, TBAF (1M in THF, 0.01 equiv.) and the reaction mixture is brought to room temperature and stirred for 2 hours (reaction was monitored by TLC). Upon completion, the reaction mixture was hydrolyzed to the corresponding alcohol with aqueous HCl (1.00 M, 3.3 equiv.) under stirring for 1 hour. After that, the reaction mixture was extracted with Et_2O (x3) and the combined organic layers washed with brine. The organic layer was dried over anhydrous Na_2SO_4, filtered, and the solvents were removed under reduced pressure to obtain the crude product. The obtained crude mixture was purified by flash column chromatography on silica gel using hexane/EtOAc (3:1) as eluent to afford **29a** as a colorless liquid (18 mg, 0.08 mmol, 45%).

^{1}H NMR (300 MHz, $CDCl_3$) δ 3.82 – 3.51 (m, 4H), 2.17 (s, 1H), 2.15 – 2.04 (m, 1H), 1.98 – 1.61 (m, 7H), 1.51 (t, J = 5.4 Hz, 2H). **^{13}C NMR** (75 MHz, $CDCl_3$) δ 126.0 (q, J = 282.6 Hz), 81.8 (q, J = 29.7 Hz), 65.3, 65.2, 46.3, 40.3, 38.7, 38.2, 36.4, 33.5. **^{19}F NMR** (282 MHz, $CDCl_3$) δ -81.9. **IR**: 3333, 2959, 2934, 2863, 1167, 912, 836, 735. **HRMS** (ESI-TOF) m/z: $[M+H]^+$ Calcd. for $C_{10}H_{16}F_3O$ 225.1102; Found: 225.1097.

2-bromo-8-azaspiro[4.5]dec-1-ene (7-NH)

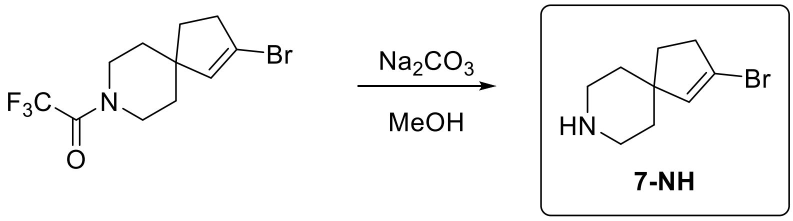

In a round-bottom flask, a solution **7a** (822 mg, 2.6 mmol) in MeOH (0.10 M) was prepared. Then, Na_2CO_3 (3.0 equiv.) was added portion wise, a reflux condenser was attached to the round-bottom flask and the reaction mixture was heated to 60 ºC for 2 hours. After completion of the reaction (checked by TLC), the reaction mixture was concentrated under reduced pressure, washed with water and extracted with DCM (x3). The combined organic layer was washed brine and dried over anhydrous Na_2SO_4, filtered, and concentrated under reduced pressure using a rotary evaporator. **7-NH** was obtained in sufficient purity to be used in the next step without further purification as a colorless liquid (500 mg, 2.30 mmol, 89%).

^{1}H NMR (300 MHz, $CDCl_3$) δ 5.82 (s, 1H), 2.82 (bs, 4H), 2.66 – 2.54 (m, 2H), 1.81 (dd, *J* = 8.0, 6.8 Hz, 2H), 1.49 (bd, *J* = 27.5 Hz, 5H). **^{13}C NMR** (75 MHz, $CDCl_3$) δ 138.9, 120.6, 48.5, 43.5, 38.4, 36.1. **HRMS** (ESI-TOF) m/z: $[M]^+$ Calcd. for $C_9H_{14}BrN$ 215.0310; Found: 215.0298.

***tert*-butyl 2-bromo-8-azaspiro[4.5]dec-1-ene-8-carboxylate (20a)**

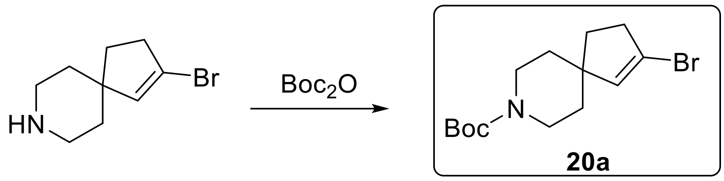

In a flame-dried Young tube under Ar atmosphere, Boc_2O (0.9 equiv.) was added and dissolved in the minimum amount of dry dioxane. Then, **7-NH** (500 mg, 2.30 mmol) dissolved in dry dioxane was added dropwise. The reaction mixture was heated to 50 ºC and stirred overnight. After completion of the reaction (checked by TLC), the reaction mixture was concentrated under reduced pressure. The crude reaction mixture was purified by flash chromatography using hexane/EtOAc (5:1) as eluent to afford **20a** as a colorless liquid (680 mg, 2.10 mmol, 93%).
^{1}H NMR (300 MHz, $CDCl_3$) δ 5.80 (t, *J* = 2.0 Hz, 1H), 3.49 (ddd, *J* = 13.6, 6.8, 4.1 Hz, 2H), 3.27 (ddd, *J* = 13.6, 8.4, 3.8 Hz, 2H), 2.63 (ddd, *J* = 7.9, 6.7, 2.0 Hz, 2H), 1.82 (dd, *J* = 8.0, 6.8 Hz, 2H), 1.60 – 1.47 (m, 2H), 1.44 (s, 11H). **^{13}C NMR** (75 MHz, $CDCl_3$) δ 154.8, 137.6, 121.2, 79.4, 48.1, 41.0, 38.3, 36.3, 35.2, 28.4. **HRMS** (ESI-TOF) m/z: $[M]^+$ Calcd. for $C_{14}H_{22}BrNO_2$ 315.0834; Found: 315.0828.

8-(*tert*-butoxycarbonyl)-8-azaspiro[4.5]dec-1-ene-2-carboxylic acid (21b-C=C)

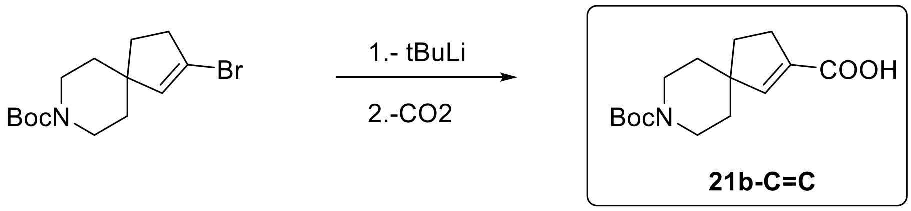

A solution of **20a** (190 mg, 0.60 mmol) in THF (0.10 M) was added to a Schlenk flask under an inert atmosphere and cooled to -78°C using a cryostat. Once this temperature was reached, ^{t}BuLi (1.7 M in THF, 2.1 equiv.) was added dropwise very carefully, and the mixture was stirred for one hour. After that time, CO_2 was bubbled through the reaction mixture for fifteen minutes, then the mixture was allowed to warm to room temperature. The reaction was then quenched with a saturated Na_2CO_3 solution and was extracted three times with the same aqueous solution. The resulting aqueous phase was acidified with HCl (2.00 M), followed by three extractions with EtOAc. The organic phases were combined, dried over anhydrous Na_2SO_4, filtered, and the solvents were removed under reduced pressure. The crude mixture was purified by flash column

chromatography using Hexane/EtOAc (2:1) as eluent yielding the carboxylic acid (***21b-C=C***) as a colorless liquid (93 mg, 0.33 mmol, 55%).

^{1}H NMR (300 MHz, $CDCl_3$) δ 6.76 (s, 1H), 3.61 (dt, J = 10.6, 3.2 Hz, 2H), 3.24 (ddd, J = 13.2, 8.9, 3.7 Hz, 2H), 2.61 (td, J = 6.6, 3.5 Hz, 2H), 1.86 (t, J = 7.3 Hz, 2H), 1.59 (td, J = 9.1, 4.4 Hz, 2H), 1.45 (s, 11H). **^{13}C NMR** (75 MHz, $CDCl_3$) δ 170.3, 155.0, 152.0, 134.8, 79.8, 48.9, 41.1, 35.5, 34.6, 29.6, 28.6. **HRMS** (ESI-TOF) m/z: $[M]^+$ Calcd. for $C_{15}H_{23}NO_4$ 281.1627; Found: 281.1622.

8-(*tert*-butoxycarbonyl)-8-azaspiro[4.5]decane-2-carboxylic acid (21b)

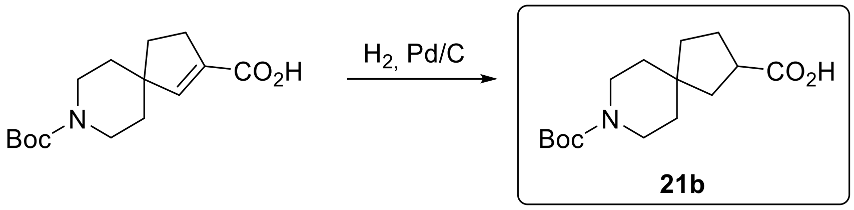

21b

A round bottom flask with a magnetic stir bar and Pd/C (10% w/w) was evacuated to vacuum and filled with Ar. A solution of **21b-C=C** (563 mg, 2.00 mmol) in AcOEt (0.50 M) was added and hydrogen was bubbled for 15 minutes. The reaction mixture was maintained on a hydrogen atmosphere and stirred overnight at room temperature. The day after, the solids were filtered through a pad of celite. Removal of solvents under vacuum afforded **21b** (567 mg, 2.00 mmol, 100%) in sufficient purity to be used in the next step without further purification.

^{1}H NMR (300 MHz, $CDCl_3$) δ 10.13 (s, 1H), 3.36 – 3.30 (m, 4H), 2.85 (p, J = 8.5 Hz, 1H), 2.04 – 1.88 (m, 3H), 1.75 – 1.48 (m, 3H), 1.41 (s, 9H). **^{13}C NMR** (75 MHz, $CDCl_3$) δ 181.8, 154.9, 79.4, 42.5, 41.5, 40.6, 37.2, 37.0, 36.6, 28.4, 28.1. **HRMS** (ESI-TOF) m/z: $[M]^+$ Calcd. for $C_{15}H_{25}NO_4$ 283.1784; Found: 283.1779.

***tert*-butyl 2-carbamoyl-8-azaspiro[4.5]decane-8-carboxylate (21b-NH$_2$)**

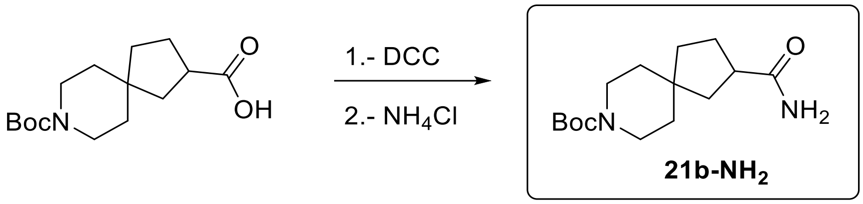

21b-NH$_2$

A solution of **21b** (142 mg, 0.50 mmol) in DCM (0.30 M) was prepared under inert atmosphere. N,N′-Dicyclohexylcarbodiimide (1.5 equiv.) was then added dropwise, and stirred for 30 minutes. After this time, ammonium chloride (1.5 equiv.) was added dropwise, the reaction was stirred at room temperature for 12 hours. After completion of the reaction, the mixture was quenched with H_2O and extracted with CH_2Cl (x3). The combined organic phases were dried over anhydrous Na_2SO_4, filtered, and the solvents were removed under reduced pressure yielding **21b-NH$_2$** as a colorless liquid (85 mg, 0.30 mmol, 60%). This compound was used without further purification.

^{1}H NMR (300 MHz, $CDCl_3$) δ 3.97 (bs, 1H), 3.67 (bs, 1H), 3.34 (t, J = 5.7 Hz, 4H), 2.99 (p, J = 8.4 Hz, 1H), 1.98 – 1.87 (m, 2H), 1.83 – 1.64 (m, 4H), 1.66 – 1.57 (m, 2H), 1.43

(s, 9H), 1.24 (t, $J = 7.1$ Hz, 2H). **^{13}C NMR** (75 MHz, $CDCl_3$) δ 154.9, 154.1, 79.2, 49.8, 43.0, 42.0, 41.7, 37.2, 32.7, 31.0, 29.5, 28.4, 26.2, 25.4, 25.3, 24.7. **HRMS**: non purified

tert-butyl 2-(aminomethyl)-8-azaspiro[4.5]decane-8-carboxylate (23b)

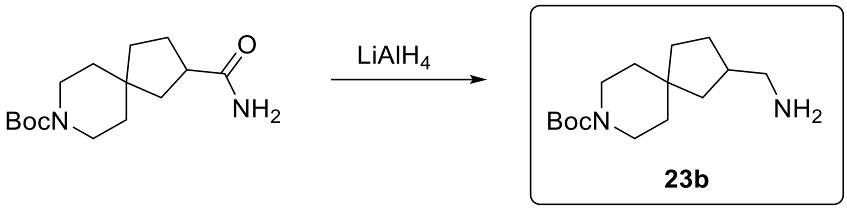

At 0 ºC solution of **21b-NH$_2$** (85 mg, 0.30 mmol) in dry THF (1.00 M) was added dropwise to a suspension of $LiAlH_4$ (1.6 equiv.) in dry THF (0.40 M). After the addition was complete the reaction mixture was stirred for 1-3 hours at room temperature. Then, the reaction was carefully quenched by dropwise addition of water (1.0 mL per gram of LAH), NaOH 15% (1.0 mL per gram of LAH) and water (3.0 mL per gram of LAH) at 0 ºC. Solids were filtered through alternate pads of celite /silica gel/ celite and the solvents removed by rotary evaporation. The crude was purified by acid-base extraction yielding **23b** as a colorless solid (45 mg, 0.15 mmol, 50%).

^{1}H NMR (300 MHz, MeOD) δ 3.49 – 3.26 (m, 5H), 2.30-2.11 (m, 1H), 1.91 – 1.62 (m, 5H), 1.56 – 1.30 (m, 14H), 1.28 – 1.00 (m, 3H). **^{13}C NMR** (75 MHz, MeOD) δ 153.7, 77.9, 64.6, 39.4, 39.3, 39.0, 35.2, 31.8, 25.8, 23.8, 23.1. **HRMS** (ESI-TOF) m/z: $[M]^+$ Calcd. for $C_{15}H_{28}N_2O_2$ 269.2229; Found: 269.1985.

tert-butyl 2-(((benzyloxy)carbonyl)amino)-8-azaspiro[4.5]decane-8-carboxylate (24b-int)

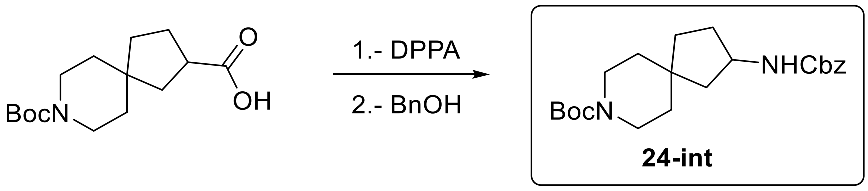

21b (397 mg, 1.4 mmol) and triethylamine (1.4 equiv.) were dissolved in anhydrous toluene (0.077 M), and the solution was heat up to 80 °C. Diphenyl phosphorazidate (1.3 equiv.) was added dropwise (CAUTION: moderate gas evolution). Immediately after the gas release ceased, benzyl alcohol (1.2 equiv.) was added, and the heating was continued overnight. After cooling, the mixture was washed with saturated sodium bicarbonate solution and water, dried over anhydrous Na_2SO_4, and the solvent was removed under reduced pressure yielding ***21b-int*** as a colorless liquid (404 mg, 1.00 mmol, 74%). This compound was used without further purification.

^{1}H NMR (300 MHz, $CDCl_3$) δ 7.43 – 7.27 (m, 5H), 5.06 (s, 2H), 4.67 (d, *J* = 3.7 Hz, 2H), 3.33 (h, *J* = 7.5 Hz, 4H), 2.28 – 1.77 (m, 2H), 1.43 (s, 17H).

***tert*-butyl 2-amino-8-azaspiro[4.5]decane-8-carboxylate (24b)**

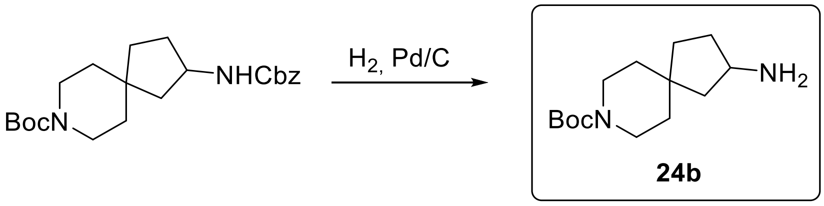

A round bottom flask with a magnetic stir bar and Pd/C (10% w/w) was evacuated and backfilled with Ar (x3). A solution of **24b-int** (404 mg, 1.00 mmol) in AcOEt (0.50 M) was added and hydrogen was bubbled for 15 minutes. The reaction mixture was maintained on a hydrogen atmosphere and stirred overnight at room temperature. The day after, the solids were filtered through a pad of celite. Removal of solvents under vacuum afforded **24b** as a colorless liquid (92 mg, 0.38 mmol, 38%).
^{1}H NMR (300 MHz, $CDCl_3$) δ 3.32 (ddt, *J* = 9.2, 7.2, 5.2 Hz, 5H), 2.00 – 1.80 (m, 2H), 1.75 (s, 2H), 1.59 (ddd, *J* = 12.1, 7.9, 3.7 Hz, 1H), 1.42 (s, 13H), 1.24 – 1.01 (m, 1H). **^{13}C NMR** (75 MHz, $CDCl_3$) δ 155.1, 79.3, 52.5, 48.1, 40.4, 39.1, 38.0, 36.3, 35.3, 28.6. **HRMS** (ESI-TOF) m/z: [M+H]+ Calcdd for $C_{10}H_{21}ClNO$ 205.1233; Found 205.1225.

2,2,2-trifluoro-1-(2-(4,4,5,5-tetramethyl-1,3,2-dioxaborolan-2-yl)-8-azaspiro[4.5]dec-1-en-8-yl)ethan-1-one (26b)

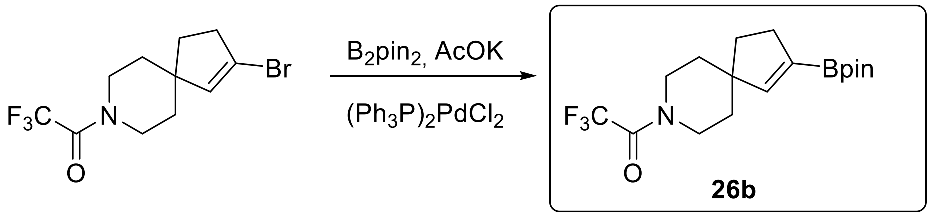

7a (156mg, 0,5 mmol) was dissolved in dioxane (0.30 M) in a two-neck flask under an argon atmosphere. $(Ph_3P)_2PdCl_2$ (0.03 equiv.), B_2Pin_2 (2.0 equiv.), and KOAc (2.0 equiv.) were then added, and the reaction mixture was stirred at 90°C for 6 hours, monitoring the progress by TLC. Upon completion of the reaction, the mixture was filtered and then centrifuged to collect the supernatant. The solvent was evaporated, and the crude product **26b** was purified by flash column chromatography using hexane/EtOAc (5:1) as eluent yielding the borolane derivative **26b** as a colorless solid (180 mg, 0.50 mmol, 99%).
^{1}H NMR (300 MHz, $CDCl_3$) δ 6.29 (s, 1H), 3.80 (ddd, *J* = 13.5, 6.6, 4.1 Hz, 1H), 3.61 (dt, *J* = 10.6, 4.7 Hz, 1H), 3.40 (ddd, *J* = 13.5, 8.4, 3.7 Hz, 2H), 2.43 (ddd, *J* = 7.9, 6.4, 2.0 Hz, 2H), 1.79 – 1.65 (m, 2H), 1.65 – 1.51 (m, 2H), 1.51 – 1.38 (m, 2H), 1.20 (s, 12H). **^{13}C NMR** (75 MHz, $CDCl_3$) δ 155.1 (q, *J* = 35.3 Hz), 151.7, 116.4 (q, *J* = 288.0 Hz), 83.2, 49.5, 43.5 (q, *J* = 3.8 Hz), 41.3, 36.2, 35.6, 35.2, 32.8, 24.8, 24.6. **^{19}F NMR** (282 MHz, $CDCl_3$) δ -68.96. **HRMS** (ESI-TOF) m/z: $[M]^+$ Calcd. for $C_{17}H_{25}BF_3NO_4$ 358.1916; Found: 358.1923.

8-(2,2,2-trifluoroacetyl)-8-azaspiro[4.5]decan-2-one (28b)

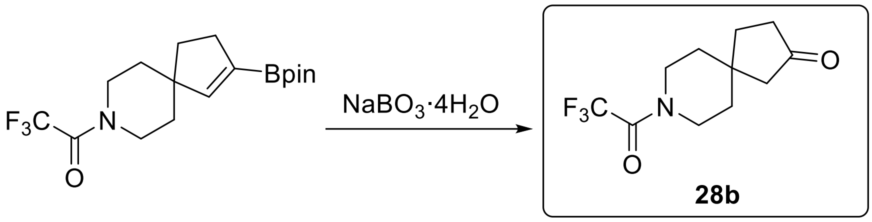

$NaBO_3{\cdot}4H_2O$ (5.0 equiv.) was added to a solution of **26b** (180 mg, 0.50 mmol) in a THF/H_2O mixture (0.30 M). The reaction mixture was stirred at room temperature for 2 hours. After that time, the reaction was diluted with water. The mixture was then extracted times with EtOAc (x3), and the combined organic phases were washed with water. The organic layer was dried over anhydrous Na_2SO_4, filtered, and the solvents were removed under reduced pressure to obtain the crude product. The obtained crude mixture was purified by flash column chromatography on silica gel using hexane/EtOAc (3:1) as eluent to afford **28b** as a colorless liquid (66 mg, 0.39 mmol, 53%).

^{1}H NMR (300 MHz, $CDCl_3$) δ 3.85 (dt, J = 13.8, 5.2 Hz, 1H), 3.72 – 3.58 (m, 1H), 3.36 (dq, J = 13.8, 6.7 Hz, 2H), 2.31 – 2.20 (m, 2H), 2.16 (s, 2H), 1.87 (t, J = 7.7 Hz, 2H), 1.59 (q, J = 5.1 Hz, 4H). **^{13}C NMR** (75 MHz, $CDCl_3$) δ 217.5, 155.2 (q, J = 35.6 Hz), 116.3 (q, J = 287.9 Hz), 49.2, 42.9, 40.6, 38.5, 36.5, 35.8, 35.4, 33.6. **^{19}F NMR** (282 MHz, $CDCl_3$) δ -68.92. **HRMS** (ESI-TOF) m/z: $[M]^+$ Calcd. for $C_{11}H_{14}F_3NO_2$ 249.0977; Found: 249.0975.

***N*-phenyl-8-oxaspiro[4.5]decane-2-carboxamide (31)**

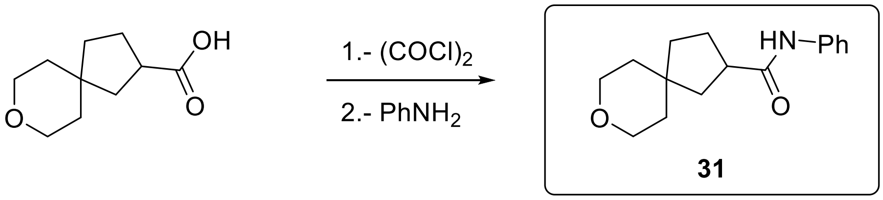

21a (92 mg, 0.50 mmol) was dissolved in dry toluene (0.20 M) and added to a flask under inert atmosphere. A solution of oxalyl chloride (6 equiv.) in dry toluene (0.20 M), was then added dropwise at 0°C, and the reaction mixture was stirred at 0ºC for 10 minutes. After this time, stirring was continued for 2 additional hours at room temperature. Meanwhile, a solution of aniline (1.4 equiv.) and triethylamine (2.0 equiv.) in dry DCM (0.33 M) was prepared and cooled to 0°C. After the 2-hour reaction time, toluene was evaporated, and the resulting acid chloride was redissolved in dry toluene (0.20 M). This solution was then added to the previously prepared aniline solution and stirred for 1 hour. After completion of the reaction, the solvents were removed, and the crude product was treated with chloroform and a saturated $NaHCO_3$ solution. The organic phase was extracted and washed with 1% HCl (x2) and with brine (x2). The organic layer was dried over anhydrous Na_2SO_4, filtered, and the solvents were removed under reduced pressure to obtain the crude product. The crude product was purified by column chromatography using silica gel as the stationary phase and a hexane/EtOAc (3:1) as eluent. The title compound **31** was obtained as a colorless liquid (79 mg, 0.31 mmol, 61%).

¹H NMR (300 MHz, $CDCl_3$) δ : 8.10 (s, 1H), 7.52 (d, J= 9.8 Hz, 2H), 7.25 (t, J= 7.9 Hz, 2H), 7.05 (t, J= 7.4 Hz, 1H), 3.60 (t, J= 5.4 Hz, 4H), 2.80 (q, J= 8.5 Hz, 1H), 2.00 – 1.89 (m, 2H), 1.80 (dd, J= 8.7, 4.0 Hz 2H), 1.67 – 1.34 (m, 6H). **¹³C NMR** (75 MHz, $CDCl_3$) δ : 174.5, 138.3, 128.9, 124.1, 120.0, 65.7, 65.3, 45.4, 42.0, 40.9, 38.2, 37.9, 37.8, 28.8. **HRMS** (ESI-TOF) m/z: [M]+ Calcdd for $C_{16}H_{21}NO_2$ 259.1572; Found 259.1574.

N-(8-oxaspiro[4.5]decan-2-yl)benzamide (33)

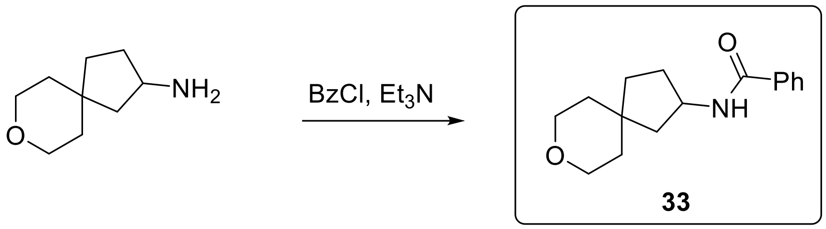

33

A solution of **24a** (58 mg, 0.30 mmol) and triethylamine (3.0 equiv.) in DCM (0.40 M) was prepared in a Schlenk flask under an inert atmosphere. The solution was cooled to 0°C, and benzoyl chloride (1.3 equiv.) was added dropwise. The reaction mixture was then stirred at room temperature for 12 hours. After completion of the reaction, water was added to the reaction mixture, and the aqueous phase was extracted with DCM (x3). The combined organic phases were washed first with a saturated $NaHCO_3$ solution and then with water. The organic layer was dried over anhydrous Na_2SO_4, filtered, and the solvents were removed under reduced pressure to obtain the crude product that was purified by flash column chromatography on silica gel using hexane/EtOAc (3:1) as eluent. The title compound **33** was obtained as a colorless liquid (47 mg, 0.18 mmol, 60%).
¹H NMR (300 MHz, $CDCl_3$) δ : 7.73 (d, J = 6.8 Hz, 2H), 7.47 – 7.33 (m, 3H), 6.49 (d, J = 7.5 Hz, 1H), 4.41 (s,1H), 3.77 – 3.51 (m, 4H), 2.21 – 2.05 (m, 2H), 1.69 – 1.40 (m, 7H), 1.33 (dd, J = 13.1, 8.7 Hz, 1H). **¹³C NMR** (75 MHz, $CDCl_3$) δ: 167.3, 134.6, 131.4, 128.5, 126.9, 65.3, 65.2, 50.5, 45.2, 39.5, 39.1, 38.4, 36.5, 31.7. **HRMS** (ESI-TOF) m/z: [M]+ Calcdd for $C_{16}H_{21}NO_2$ 259.1572; Found 259.1573.

N-((8-oxaspiro[4.5]decan-2-yl)methyl)benzamide (34)

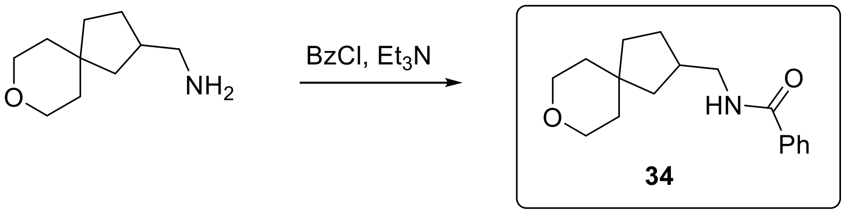

34

The title compound was synthesized following the same procedure as above, starting from **23a** (62 mg, 0.30 mmol), yielding **34** as a colorless liquid (51 mg, 0.19 mmol, 62%).
¹H NMR (300 MHz, $CDCl_3$) δ : 7.76 – 7.76 (m, 2H), 7.52 – 7.38 (m, 3H), 6.54 (s, 1H), 3.65 – 3.60 (m, 4H), 3.40 (dd, J = 7.2, 5.8 Hz, 2H), 2.29 (sp, J = 7.5 Hz, 1H), 1.94 – 1.76 (m, 2H), 1.68 – 1.42 (m, 6H), 1.42 – 1.32 (m, 1H), 1.13 (dd, J = 12.9, 9.5 Hz, 1H). **¹³C NMR** (75 MHz, $CDCl_3$) δ: 167.7, 134.8, 131.4, 128.5, 126.9, 65.6, 65.3, 45.2, 43.0, 40.3, 39.2, 38.7, 38.2, 37.5, 28.8. **HRMS** (ESI-TOF) m/z: $[M]^+$ Calcd. for $C_{17}H_{23}NO_2$ 273.1729; Found: 273.1727.

tert-butyl 2-(benzamidomethyl)-8-azaspiro[4.5]decane-8-carboxylate (35)

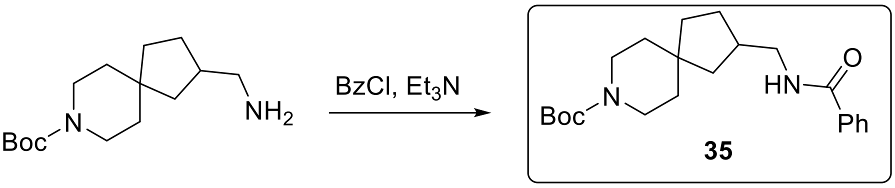

The title compound was synthesized following the same procedure as above, starting from **23b** (54 mg, 0.20 mmol) of the carboxylic acid, yielding **35** as a colorless liquid (38 mg, 0.10 mmol, 51%).
^{1}H NMR (300 MHz, $CDCl_3$) δ 8.08 – 7.99 (m, 2H), 7.61 – 7.52 (m, 1H), 7.44 (dd, *J* = 8.3, 6.8 Hz, 2H), 4.24 (d, *J* = 6.8 Hz, 2H), 3.37 (q, *J* = 5.7 Hz, 4H), 2.47 (p, *J* = 7.8 Hz, 1H), 1.86 (ddd, *J* = 30.3, 13.1, 7.3 Hz, 2H), 1.61 – 1.47 (m, 2H), 1.45 (s, 13H), 1.36 – 1.11 (m, 2H). **^{13}C NMR** (75 MHz, $CDCl_3$) δ 166.7, 155.0, 132.9, 130.4, 129.5, 128.4, 79.3, 68.9, 41.2, 41.2, 38.2, 37.7, 36.9, 28.5, 28.0. **HRMS** (ESI-TOF) m/z: $[M]^+$ Calcdd for $C_{22}H_{32}N_2O_3$ 372.2413; Found 372.2416.

tert-butyl 2-benzamido-8-azaspiro[4.5]decane-8-carboxylate (36)

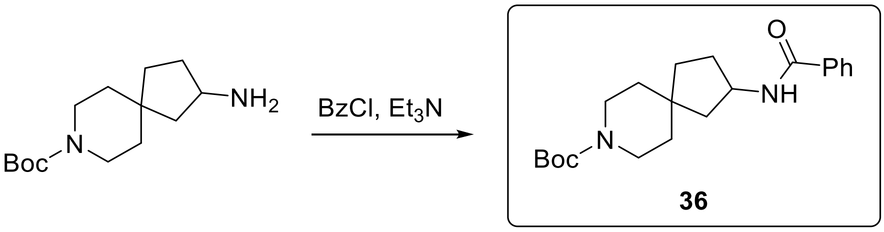

The title compound was synthesized following the same procedure as above, starting from **24b** (25 mg, 0.10 mmol), yielding **36** as a colorless liquid (22 mg, 0.06 mmol, 57%).
^{1}H NMR (300 MHz, $CDCl_3$) δ 7.78 – 7.68 (m, 2H), 7.51 – 7.36 (m, 3H), 6.15 (d, *J* = 7.4 Hz, 1H), 4.47 (h, *J* = 7.7 Hz, 1H), 3.47 – 3.22 (m, 4H), 2.28 – 2.07 (m, 2H), 1.64 – 1.25 (m, 15H), 0.97 – 0.77 (m, 2H). **^{13}C NMR** (75 MHz, $CDCl_3$) δ 167.4, 155.0, 134.8, 131.5, 128.7, 127.0, 79.5, 50.7, 44.9, 40.1, 38.8, 37.6, 36.1, 32.1, 28.6. **HRMS** (ESI-TOF) m/z: $[M]^+$ Calcdd for $C_{21}H_{30}N_2O_3$ 358.2256; Found 358.2260.

tert-butyl 2-(phenylcarbamoyl)-8-azaspiro[4.5]decane-8-carboxylate (37)

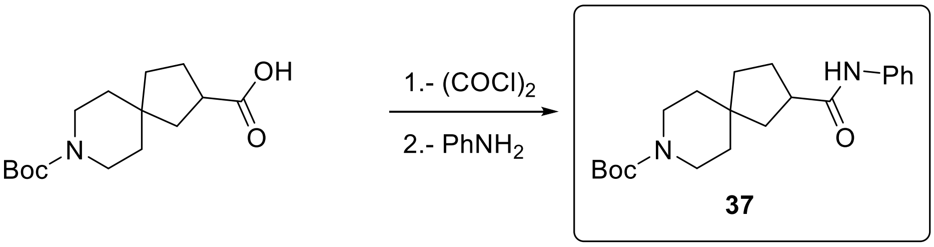

21b (57 mg, 0.2 mmol) was dissolved in dry toluene (0.20 M) and added to a flask under inert atmosphere. A solution of oxalyl chloride (6.0 equiv.) in dry toluene (0.20 M), was then added dropwise at 0°C, and the reaction mixture was stirred at 0ºC for 10 minutes.

After this time, stirring was continued for 2 additional hours at room temperature. Meanwhile, a solution of aniline (1.4 equiv.) and triethylamine (2.0 equiv.) in dry DCM (0.33 M) was prepared and cooled to 0°C. After the 2-hour reaction time, toluene was evaporated, and the resulting acid chloride was redissolved in dry toluene (0.20 M). This solution was then added to the previously prepared aniline solution and stirred for 1 hour. After completion of the reaction, the solvents were removed, and the crude product was treated with chloroform and a saturated $NaHCO_3$ solution. The organic phase was extracted and washed with 1% HCl (x2) and with brine (x2). The organic layer was dried over anhydrous Na_2SO_4, filtered, and the solvents were removed under reduced pressure to obtain the crude product. The crude product was purified by column chromatography using silica gel as the stationary phase and a hexane/EtOAc (3:1) as eluent. The title compound **31** was obtained as a colorless liquid (49 mg, 0.14 mmol, 68%).
^{1}H NMR (300 MHz, C_6D_6) δ 7.61 (d, *J* = 8.0 Hz, 2H), 7.43 – 7.31 (m, 2H), 7.16 (t, *J* = 7.3 Hz, 1H), 3.44 (t, *J* = 5.7 Hz, 4H), 2.90 (p, *J* = 8.5 Hz, 1H), 2.07 (td, *J* = 8.1, 5.3 Hz, 2H), 1.89 (dt, *J* = 7.7, 3.5 Hz, 2H), 1.80 – 1.64 (m, 2H), 1.63 – 1.56 (m, 2H), 1.53 (s, 9H). **^{13}C NMR** (75 MHz, C_6D_6) δ 174.0, 154.9, 138.1, 128.9, 124.1, 119.7, 79.3, 45.6, 41.7, 41.3, 37.3, 36.8, 28.9, 28.4. **HRMS** (ESI-TOF) m/z: $[M]^+$ Calcdd for $C_{21}H_{30}N_2O_3$ 358.2256; Found 358.2259.

***tert*-butyl 3-(2-hydroxyethyl)azetidine-1-carboxylate (38-OH)**

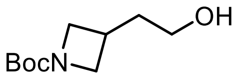

The title compound was synthesized using General Procedure A2, starting from 2-(1-(tert-butoxicarbonyl)azetidin-3-yl)acetic acid (1.70g, 8.00 mmol), yielding **38-*OH*** as a colorless oil (1.70 g, 99%).
^{1}H NMR (300 MHz, $CDCl_3$) δ 3.97 (td, J = 8.4, 4.2 Hz, 2H), 3.71 (p, J = 6.3 Hz, 1H), 3.61 – 3.47 (m, 3H), 2.59 (ttd, J = 13.1, 6.3, 4.9 Hz, 1H), 1.88 – 1.69 (m, 2H), 1.39 (s, 9H).. **^{13}C NMR** (75 MHz, $CDCl_3$) δ 156.4, 79.3, 60.2, 54.5, 37.1, 28.4, 26.0. **HRMS** (ESI-TOF) m/z: $[M]^+$ Calcd. for $C_{10}H_{19}NO_3$ 201.1365; Found: 201.1361.

***tert*-butyl 3-(2-bromoethyl)azetidine-1-carboxylate (38-Br)**

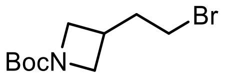

The title compound was synthesized using General Procedure A2, starting from **38-*OH*** (1.7g, 8.00 mmol), yielding **1738-*Br*** as a colorless oil (389 mg, 18%). The crude was purified by flash column chromatography using Hex:AcOEt (10:1) as eluent.
^{1}H NMR (300 MHz, $CDCl_3$) δ 4.84 (bs, 1H), 3.51 (dd, J = 10.9, 4.8 Hz, 2H), 3.50 – 3.38 (m, 2H), 3.16 (ddt, J = 20.9, 14.0, 6.7 Hz, 2H), 2.17 (t, J = 6.0 Hz, 1H), 1.92 (qd, J = 6.9, 4.2 Hz, 2H), 1.43 (s, 9H). **^{13}C NMR** (75 MHz, $CDCl_3$) δ 156.0, 79.6, 42.3, 38.2, 35.6, 33.2, 30.6, 28.4. **HRMS** (ESI-TOF) m/z: $[M]^+$ Calcd. for $C_{10}H_{18}BrNO_2$ 263.0521; Found: 263.0519.

***tert*-butyl 3-(4-(trimethylsilyl)but-3-yn-1-yl)azetidine-1-carboxylate (38-CCTMS)**

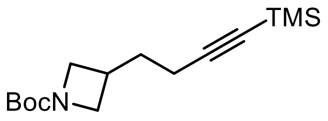

In a Schlenk flask under Ar, BuLi (0.75 mL, 1.2 mmol, 1.2 equiv., 1.6 M in *n*-hexane) was added dropwise to a solution of trimethylsilylacetylene (0.17 mL, 1.20 mmol, 1.2 equiv.) in dry THF (0.10 M) at -78 ºC. Then, HMPA (0.2 mL, 1.10 mmol , 1.1 equiv.) and **38-*Br*** (278 mg, 1.00 mmol, 1 equiv.) were added. The reaction mixture was stirred for 1h at this temperature and then was allowed to reach room temperature during 24h extra hours. The reaction was then quenched with a saturated solution of NH_4Cl at 0 ºC and extracted with AcOEt (×3). The combined organic layer was dried over anhydrous Na_2SO_2, Filtered and concentrated under reduced pressure. The crude reaction mixture was purified by flash column chromatography using Hex:AcOEt (10:1) as eluent, yielding **38-*CCTMS*** as a colorless oil (217 mg, 77%).

^{1}H NMR (300 MHz, $CDCl_3$) δ 3.99 (t, J = 8.4 Hz, 2H), 3.62 – 3.52 (m, 2H), 2.80 – 2.51 (m, 1H), 2.32 – 2.08 (m, 2H), 1.89 – 1.63 (m, 2H), 1.41 (s, 9H), 0.12 (s, 9H). **^{13}C NMR** (75 MHz, $CDCl_3$) δ 156.3, 105.9, 85.2, 79.1, 54.4, 32.9, 28.5, 28.4, 17.8, -0.0. **HRMS** (ESI-TOF) m/z: $[M]^+$ Calcd. for $C_{15}H_{27}NO_2Si$ 281.1811; Found: 281.1813.

tert-butyl 3-(4-(trimethylsilyl)but-3-yn-1-yl)azetidine-1-carboxylate (38-NBoc)

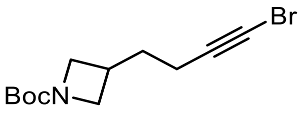

The title compound was synthesized using General Procedure D, starting from **17-*CCTMS*** (281 mg, 1.00 mmol), yielding **38-*NBoc*** as a colorless oil (175 mg, 60%). The crude was purified by flash column chromatography using hexane/EtOAc (10:1) as eluent.

^{1}H NMR (300 MHz, $CDCl_3$) δ 3.88 (t, J = 8.4 Hz, 2H), 3.43 (dd, J = 8.6, 5.6 Hz, 2H), 2.59 – 2.41 (m, 1H), 2.08 (t, J = 7.0 Hz, 2H), 1.68 (q, J = 7.2 Hz, 2H), 1.30 (s, 9H). **^{13}C NMR** (75 MHz, $CDCl_3$) δ 156.1, 79.0, 54.1, 38.6, 32.6, 28.3, 28.1, 17.4. **HRMS** (ESI-TOF) m/z: $[M-C_4H_8]^+$:Calcd. for $C_8H_{10}BrNO_2$ 230.9895; Found: 230.9891.

1-(3-(4-bromobut-3-yn-1-yl)azetidin-1-yl)-2,2,2-trifluoroethan-1-one (38)

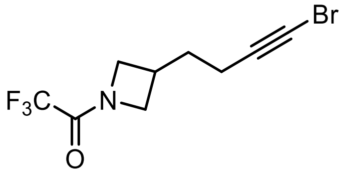

In a round bottom flask, trifluoroacetic acid (10.0 equiv.) was added to a solution of **38-*NBoc*** (181 mg, 0.60 mmol) in DCM (0.5M) at 0, ºC. The reaction mixture was stirred at room temperature for 2h and then, volatiles were removed under reduced pressure and the obtained crude product dried under high vacuum. The residue was taken in DCM, precipitated with Et_2O, washed several times with Et_2O and dried under high vacuum. The obtained trifluoroacetate salt was dissolved in DCM and trifluoroacetic anhydride (1.1 equiv.), Et_3N (3.0 equiv.) and DMAP (10 mol%) were successively added. After

stirring for 2h at room temperature, the reaction was diluted with DCM, the organic layer washed with water, dried over anhydrous Na_2SO_4, filtered and concentrated under vacuum. The title compound was purified by flash column chromatography using Hex:AcOEt (10:1) as eluent (143 mg, 84%).

^{1}H NMR (300 MHz, $CDCl_3$) δ 4.50 (t, J = 9.2 Hz, 1H), 4.25 (t, J = 9.7 Hz, 1H), 4.13 – 3.90 (m, 1H), 3.79 (dd, J = 10.9, 5.9 Hz, 1H), 2.87 (p, J = 7.4 Hz, 1H), 2.25 (t, J = 6.8 Hz, 2H), 1.86 (q, J = 7.1 Hz, 2H). **^{13}C NMR** (75 MHz, $CDCl_3$) δ 156.2 (q, *J*=37.0), 116.1 (q, *J*=288.1), 78.4, 56.9, 54.0, 39.3, 32.2, 29.2, 17.5. **HRMS** (ESI-TOF) m/z: $[M]^+$ Calcd. for $C_9H_9BrF_3NO$: 282.9820; Found: 282.9796.

ciclohexil ((3-bromoprop-2-in-1-il)oxi) (39)

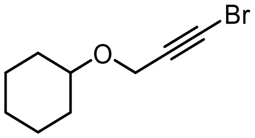

The title compound was synthesized using General Procedure D, starting from 1.4 mmol (192 mg) of the corresponding alkyne ((prop-2-yn-1-yloxy)cyclohexane), yielding the bromoalkyne (***39***) as a colorless oil (228 mg, 75%). The crude was purified by flash column chromatography using hexane/EtOAc (10:1) as eluent.

^{1}H NMR (300 MHz, $CDCl_3$) δ 4.22 (s, 2H), 3.45 (dq, J = 9.0, 4.3 Hz, 1H), 2.00 – 1.86 (m, 2H), 1.83 – 1.68 (m, 2H), 1.65 – 1.50 (m, 1H), 1.38 – 1.16 (m, 5H). **HRMS** (ESI-TOF) m/z: $[M]^+$ Calcdd for $C_9H_{13}BrO$ 216.0150; Found 216.0151.

2-(2-oxaspiro[3.3]heptan-6-yl)ethan-1-ol (40-OH)

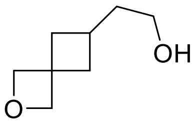

The title compound was synthesized using General Procedure A1, starting from 3.20 mmol (500 mg) of the proper carboxylic acid, yielding the crude alcohol (***40-OH***) as a colorless liquid (230 mg, 1.60 mmol, 50%). This compound was used without further purification.

^{1}H NMR (300 MHz, $CDCl_3$) δ 4.60 (d, *J* = 37.5 Hz, 4H), 3.48 (q, *J* = 6.0, 5.4 Hz, 3H), 2.42 – 2.24 (m, 3H), 1.88 – 1.73 (m, 2H), 1.54 (q, *J* = 6.9 Hz, 2H). **^{13}C NMR** (75 MHz, $CDCl_3$) δ 84.8, 83.4, 60.7, 40.1, 39.4, 38.2, 26.2. **HRMS** (ESI-TOF) m/z: $[M-OH]^+$ Calcd. for $C_8H_{13}O$ 125.0966; Found: 125.0960.

6-(2-bromoethyl)-2-oxaspiro[3.3]heptane (40-Br)

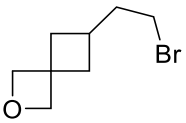

The title compound was synthesized using General Procedure B1, starting from 1.6 mmol (230 mg) of the corresponding alcohol (***40-OH***), yielding the bromoalkane (***40-Br***) as a

colorless liquid (48 mg, 0.23 mmol, 15%). This compound was used without further purification.

^{1}H NMR (300 MHz, $CDCl_3$) δ 3.67 (s, 2H), 3.52 (s, 2H), 3.37 – 3.26 (m, 2H), 2.13 – 1.88 (m, 5H), 1.65 – 1.47 (m, 2H). **^{13}C NMR** (75 MHz, $CDCl_3$) δ 85.0, 83.4, 71.7, 70.5, 68.0, 65.8, 42.0, 40.8, 40.7, 40.4, 40.2, 39.9, 37.9, 36.0, 35.1, 33.8, 33.0, 31.6, 31.5, 31.2, 31.1, 27.4, 27.1, 27.0, 26.5, 25.9, 23.5. **HRMS** (ESI-TOF) m/z: $[M-O]^+$ Calcd. for $C_8H_{13}Br$ 188.0201; Found: 188.0194.

6-(but-3-yn-1-yl)-2-oxaspiro[3.3]heptane (40-CCH)

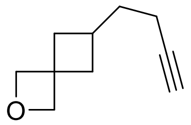

The title compound was synthesized using General Procedure C, starting from 0.23 mmol (48 mg) of the corresponding bromoalkene (***40-Br***), yielding the alkyne (***40-CCH***) as a colorless liquid (27 mg, 0.18 mmol, 78%). The crude was purified by flash column chromatography using hexane/EtOAc (10:1) as eluent.

^{1}H NMR (300 MHz, $CDCl_3$) δ 4.69 (s, 2H), 4.56 (s, 2H), 2.42 – 2.32 (m, 2H), 2.24 – 2.11 (m, 1H), 2.08 (td, J = 7.2, 2.6 Hz, 2H), 1.90 (t, J = 2.7 Hz, 1H), 1.54 (q, J = 7.3 Hz, 2H), 1.19 (t, J = 7.0 Hz, 1H). **^{13}C NMR** (75 MHz, $CDCl_3$) δ 84.8, 84.1, 83.4, 68.2, 39.8, 38.0, 35.3, 28.8, 16.2. **HRMS** (ESI-TOF) m/z: $[M]^+$ Calcdd for $C_{10}H_{14}O$ 150.1054; Found 150.1055.

6-(4-bromobut-3-yn-1-yl)-2-oxaspiro[3.3]heptane (40)

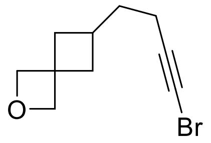

The title compound was synthesized using General Procedure D, starting from 0.18 mmol (27 mg) of the corresponding alkyne (***40-CCH***), yielding the bromoalkyne (***40***) as a colorless liquid (35 mg, 0.15 mmol, 85%). The crude was purified by flash column chromatography using hexane/EtOAc (10:1) as eluent.

^{1}H NMR (300 MHz, $CDCl_3$) δ 4.69 (s, 2H), 4.56 (s, 2H), 2.43 – 2.30 (m, 2H), 2.13 (dt, J = 14.1, 7.4 Hz, 3H), 1.84 – 1.66 (m, 2H), 1.53 (q, J = 7.2 Hz, 2H). **^{13}C NMR** (75 MHz, $CDCl_3$) δ 84.8, 83.4, 79.8, 39.9, 38.0, 37.7, 35.0, 28.8, 17.6. **HRMS** (ESI-TOF) m/z: $[M-CH_2Br]^+$ Calcd. for $C_9H_{11}O$135.0810; Found: 135.0811.

3-(but-3-yn-1-yl)tetrahydrofuran (41-CCH)

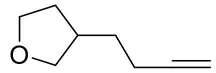

The title compound was synthesized using General Procedure C, starting from 650 mg (3.6 mmol) of commercial 3-(2-bromoethyl)oxolane, yielding the alkyne (***41-CCH***) as a colorless liquid. The crude product was used in the next step without further purification.

^{1}H NMR (300 MHz, $CDCl_3$) δ 3.94 – 3.77 (m, 2H), 3.73 (dt, *J* = 8.4, 7.4 Hz, 1H), 3.34 (dd, *J* = 8.3, 7.1 Hz, 1H), 2.32 (p, *J* = 7.4 Hz, 1H), 2.20 (ddd, *J* = 8.3, 4.9, 2.0 Hz, 2H), 2.05 (dtd, *J* = 12.2, 7.5, 4.7 Hz, 1H), 1.94 (t, *J* = 2.7 Hz, 1H), 1.60 (qd, *J* = 7.2, 2.4 Hz, 2H), 1.55 – 1.45 (m, 1H). **^{13}C NMR** (75 MHz, $CDCl_3$) δ 83.8, 72.9, 68.6, 67.8, 38.4, 32.1, 32.0, 17.5. **IR**: 3303, 2931, 2859, 1432, 1275, 1261, 1052, 913, 764, 749, 640, 410. **HRMS** (ESI-TOF) m/z: $[M]^+$ Calcd. for $C_8H_{12}O$ 123.0810; Found: 123.0810.

3-(4-bromobut-3-yn-1-yl)tetrahydrofuran (41)

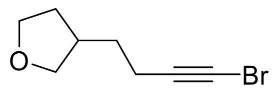

The title compound was synthesized using General Procedure D, starting the crude alkyne 41-***CCH*** obtained in the previous step, yielding the bromoalkyne (***41***) as a colorless liquid (559 mg, 2.75 mmol, 77% for the two steps from 3-(2-bromoethyl)oxolane). The crude was purified by flash column chromatography using hexane/EtOAc (10:1) as eluent.

^{1}H NMR (300 MHz, $CDCl_3$) δ 3.93 – 3.78 (m, 2H), 3.77 – 3.65 (m, 1H), 3.33 (t, *J* = 7.7 Hz, 1H), 2.26 (dt, *J* = 21.0, 7.3 Hz, 3H), 2.05 (dtd, *J* = 12.3, 7.6, 4.7 Hz, 1H), 1.60 (qd, *J* = 7.1, 2.4 Hz, 2H), 1.53 – 1.38 (m, 1H). **^{13}C NMR** (75 MHz, $CDCl_3$) δ 79.6, 72.9, 67.8, 38.5, 38.2, 32.1, 31.8, 18.8. **HRMS**: $[M]^+$ Calcd. for $C_8H_{11}BrO$ 201.9993; Found: 201.9989.

4.4. Limitations

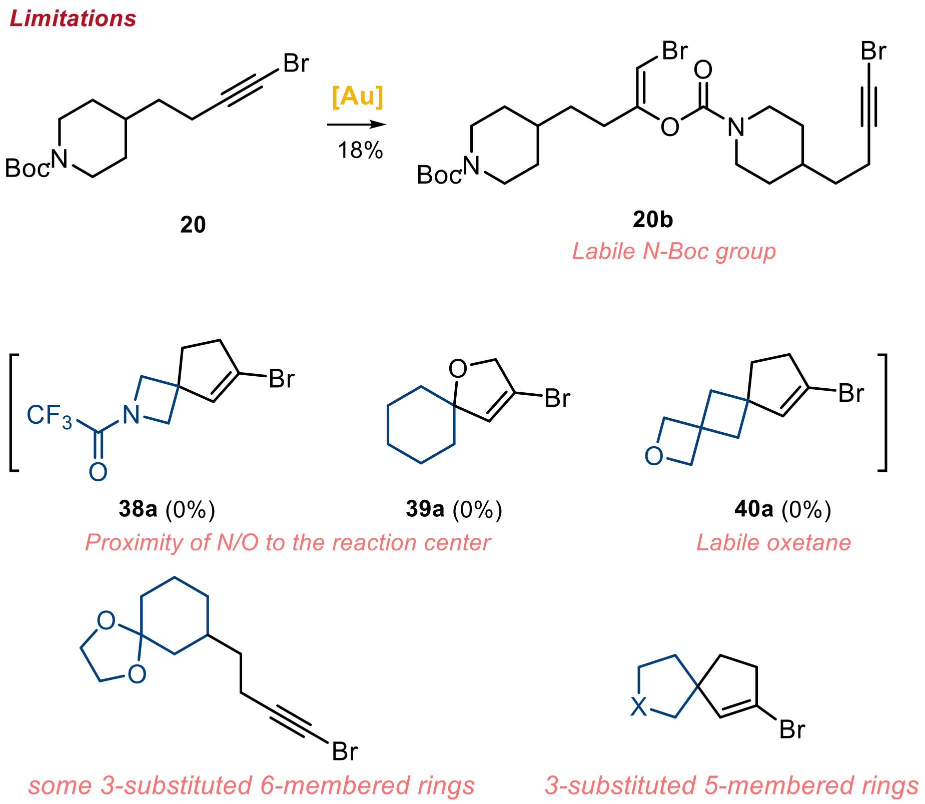

5. Determination of pKa values

The tendency of a compound to donate a proton is measured by its acid ionization constant (dissociation constant), or K_a. A more practical scale of representing acidity is pK_a which is the negative logarithm of K_a ($pK_a = -logK_a$). The pK_a of a test article is determined by pH-metric method based on potentiometric acid-base titration at 25 °C. The test compounds are dissolved in an acidified methanol-water (1:4) solution of NaCl (150 mM, pH 2) and slowly titrated with a 0.3M sodium hydroxide methanol-water (1:4) solution, while the pH of the solution is recorded as a function of the NaOH volume used during the titration (construction of the titration curve). Titration of the acidified NaCl solution in the absence of any compounds is used for blank plotting.

The buffering capacity is Calcdulated at each point of titration curve as the ratio of the NaOH flow rate (constant) to the pH rise velocity. The pK_a value is determined from the resulting plot of buffering capacity versus pH as the maximum buffering capacity. The pH-metric method allows to measure pK_a in the range of approximately between 2 and 12.

Acquisition and analysis of the data were performed using SmartLogger II 1.0.14 software (pH-meter, pHi®510 (Beckman Coulter, Canada; Cat# A58734). Data analysis was done using GraphPad Prism 6.01 software.

Reagents and consumables

Sodium hydroxide (Enamine, Ukraine; CAS # 1310-73-2)

Sodium chloride BioXtra, ≥99.5% (AT) (Sigma-Aldrich, USA; Cat # S7653)

Hydrochloric acid (Enamine, Ukraine; CAS # 7647-01-0)

Disposable pipette tips (Eppendorf, German; Matrix and Finntip, Thermo, USA)

Polypropylene graduated conical tubes, 50 mL (Kartell, Italy; Cat. # 84002)

Polypropylene syringe (10 mL) with tubing (Hemoplast, Ukraine)

Equipment

pH-meter, pHi®510 (Beckman Coulter, Canada; Cat# A58734)

Multichannel Electronic Pipettes 5-50 µL, 10-1000 µL (Thermo, USA)

Magnetic stirrer standard unit (IKA, USA)

Syringe Driver Mdl 100 (KDScientific, USA)

Table S1. Experimental pK_a values of carboxylic acids and amines.

Compound	pK_a (exp.)
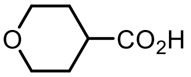 **28**	4.24
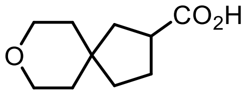 **18a**	4.70
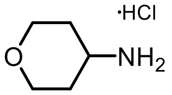 **29**	9.67
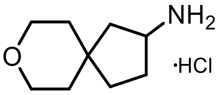 **21a**	10.49

Titration curves for compounds 28, 18a, 29, and 21a

Compound 28

Row	A	B	C	D	E	F	G	H	ml	pH
1	22.11.2024						1.064			
2		г/моль				г	моль			
3						0.065031	0.0005	0.649685		
4						0.0845	0.00065	47.5		
5	Количество	pH	ΔpH	ΔV	ΔpH/ΔV	Стехиометрически		pKa		`
6	титранта (0.1 н					[HA]	[A-]		ml	pH
7	0	3.026	0	0		0.013678	0		0	3.026
8	0.554	3.289	0.263	0.554	0.474729	0.01229	0.00123	4.288683	0.02	3.026
9	1.434	3.685	0.396	0.88	0.45	0.01016	0.003117	4.19819	0.04	3.033
10	1.828	3.827	0.142	0.394	0.360406	0.00923	0.003941	4.196626	0.06	3.037
11	2.78	4.137	0.31	0.952	0.32563	0.00704	0.005881	4.215096	0.1	3.054
12	3.298	4.305	0.168	0.518	0.324324	0.00588	0.00691	4.234926	0.18	3.092
13	3.794	4.466	0.161	0.496	0.324597	0.0048	0.007866	4.251492	0.34	3.181
14	4.68	4.801	0.335	0.886	0.378104	0.00291	0.009541	4.285306	0.554	3.289
15	5.294	5.137	0.336	0.614	0.547231	0.00164	0.010666	4.323841	0.824	3.423
16	5.848	5.742	0.605	0.554	1.092058	0.00051	0.011668	4.382565	1.124	3.563
17	6.538	11.152	5.41	0.69	7.84058	-0.00085	0.012873	########	1.434	3.685
18	7.172	11.629	0.477	0.634	0.752366	-0.00207	0.013953	########	1.828	3.827
19	Среднее							4.236689	2.304	3.989
20									2.78	4.137
21				V	%				3.298	4.305
22				6.114	100.13	**pKa**	**4.236689**		3.794	4.466
23									4.254	4.628
24									4.68	4.801
25									5.026	4.973
26									5.294	5.137
27									5.502	5.298
28									5.658	5.461
29									5.768	5.605
30									5.848	5.742
31									5.908	5.886
32									5.948	6.002
33									5.978	6.107
34									6.002	6.206
35									6.022	6.315
36									6.042	6.448
37									6.062	6.606
38									6.082	6.858
39									6.102	7.26

72-962 | *R3738381* | 130.063

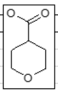

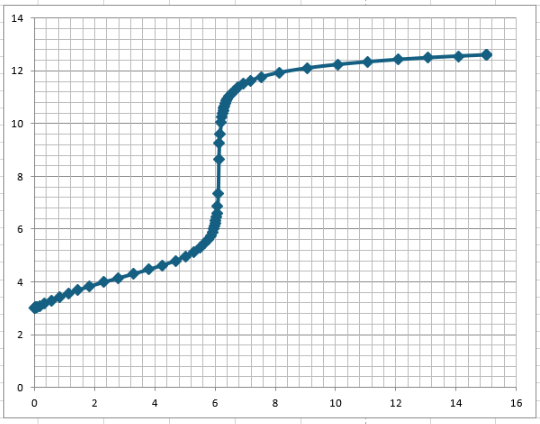

412-757 | 72-962 | 333-014 | 033-619 | 82-291 | 1373-791 | 1162-974 | 1693-939

Compound 18a

Row	A	B	C	D	E	F	G	H	ml	pH
1	27.11.2024						1.064			
2		г/моль				г	моль			
3						0.092055	0.0005	0.581718		
4						0.1071	0.000582	47.5		
5	Количество	pH	ΔpH	ΔV	ΔpH/ΔV	Стехиометрически		pKa		
6	титранта (0.1 н					[HA]	[A-]		ml	pH
7	0	3.117	0	0		0.012247	0		0	3.117
8	0.562	3.648	0.531	0.562	0.94484	0.01086	0.001243	4.58919	0.02	3.14
9	1.192	4.039	0.391	0.63	0.620635	0.00934	0.002607	4.593225	0.04	3.161
10	1.792	4.307	0.268	0.6	0.446667	0.00793	0.003871	4.618398	0.06	3.178
11	2.548	4.603	0.296	0.756	0.391534	0.00621	0.005413	4.662638	0.1	3.227
12	2.966	4.768	0.165	0.418	0.394737	0.00527	0.006257	4.69345	0.18	3.31
13	3.702	5.069	0.301	0.736	0.408967	0.00367	0.007691	4.74767	0.294	3.423
14	4.332	5.4	0.331	0.63	0.525397	0.00233	0.008893	4.818301	0.426	3.545
15	4.858	5.853	0.453	0.526	0.861217	0.00124	0.00987	4.952088	0.562	3.648
16	5.452	10.607	4.754	0.594	8.003367	0.00003	0.010956	8.044479	0.744	3.775
17	6.024	11.489	0.882	0.572	1.541958	-0.00111	0.011978	########	0.96	3.909
18	6.54	11.758	0.269	0.516	0.521318	-0.00211	0.012875	########	1.192	4.039
19	Среднее							4.708091	1.458	4.163
20									1.792	4.307
21				V	%				2.174	4.464
22				5.288	96.72102	pKa	4.708091		2.548	4.603
23									2.966	4.768
24									3.354	4.925
25									3.702	5.069
26									4.05	5.239
27									4.332	5.4
28									4.554	5.556
29									4.73	5.717
30									4.858	5.853
31									4.96	6
32									5.036	6.147
33									5.086	6.281
34									5.12	6.386
35									5.148	6.481
36									5.172	6.585
37									5.192	6.685
38									5.212	6.813
39									5.232	6.992

1162-974 | R2793043 | 184.1099

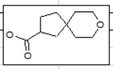

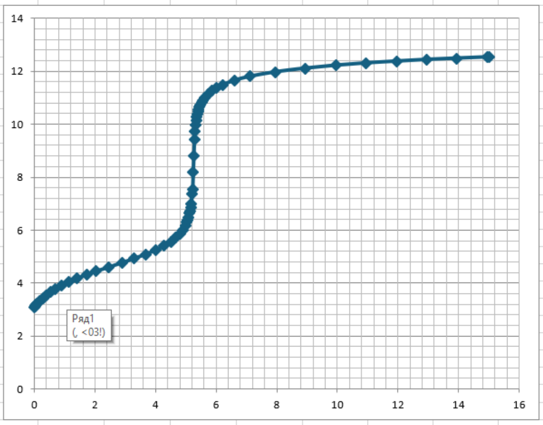

412-757 | 72-962 | 333-014 | 033-619 | 82-291 | 1373-791 | 1162-974 | 1693-939

Compound 29

	A	B	C	D	E	F	G	H	I	J	K	L	M
1	22.11.2024						1.064						
2		г/моль				г	моль				333-014	*R853887+H*	137.0607
3						0.06853	0.0005	0.553039					
4						0.0758	0.000553	47.5					
5	Количество	pH	ΔpH	ΔV	ΔpH/ΔV	Стехиометрически		pKa					
6	титранта (0.1 н					[HA]	[A-]		ml	pH			
7	0	6.119	0	0		0.011643	0		0	6.119			
8	0.34	8.411	2.292	0.34	6.741176	0.0108	0.00076	9.563502	0.02	6.932			
9	0.66	8.758	0.347	0.32	1.084375	0.01003	0.001453	9.596922	0.04	7.337			
10	1.3	9.156	0.398	0.64	0.621875	0.0085	0.002833	9.633207	0.06	7.552			
11	2.3	9.563	0.407	1	0.407	0.00619	0.004915	9.663149	0.1	7.821			
12	3.244	9.907	0.344	0.944	0.364407	0.0041	0.006799	9.687363	0.18	8.09			
13	3.824	10.142	0.235	0.58	0.405172	0.00285	0.007925	9.697821	0.34	8.411			
14	4.518	10.332	0.19	0.694	0.273775	0.00139	0.009242	9.509263	0.66	8.758			
15	4.934	10.5	0.168	0.416	0.403846	0.00054	0.010007	9.232075	1.3	9.156			
16	5.49	10.658	0.158	0.556	0.284173	-0.00059	0.011027	########	2.3	9.563			
17	6.07	10.801	0.143	0.58	0.246552	-0.00173	0.012054	########	3.244	9.907			
18	Среднее							9.670385	3.824	10.142			
19									4.222	10.332			
20				V	%				4.518	10.5			
21				5.207	100.1782	**pKa**	**9.670385**		4.75	10.658			
22									4.934	10.801			
23									5.088	10.933			
24									5.224	11.053			
25									5.356	11.172			
26									5.49	11.282			
27									5.64	11.392			
28									5.828	11.509			
29									6.07	11.636			
30									6.378	11.758			
31									6.848	11.901			
32									7.59	12.061			
33									8.59	12.21			
34									9.59	12.321			
35									10.59	12.41			
36									11.59	12.482			
37									12.59	12.543			
38									13.59	12.595			
39									14.59	12.641			

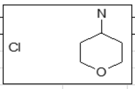

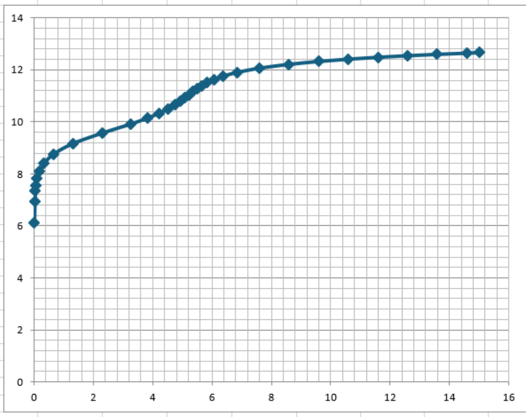

412-757 | 72-962 | 333-014 | 033-619 | 82-291 | 1373-791 | 1162-974 | 1693-939

Compound 21a

	A	B	C	D	E	F	G	H	I	J	K	L	M
1	22.11.2024						1.064						
2		г/моль				г	моль				1373-791	R1415769	191.1077
3						0.095554	0.0005	0.569836					
4						0.1089	0.00057	47.5					
5	Количество	pH	ΔpH	ΔV	ΔpH/ΔV	Стехиометрически		pKa					
6	титранта (0.1 н					[HA]	[A-]		ml	pH			
7	0	5.65	0	0		0.011997	0		0	5.65			
8	0.48	9.357	3.707	0.48	7.722917	0.01081	0.001067	10.36285	0.02	6.974			
9	0.928	9.754	0.397	0.448	0.886161	0.00973	0.002037	10.43319	0.04	7.677			
10	1.824	10.184	0.43	0.896	0.479911	0.00762	0.003933	10.47124	0.06	8.039			
11	2.824	10.561	0.377	1	0.377	0.00535	0.005973	10.51314	0.088	8.324			
12	4.112	11.006	0.445	1.288	0.345497	0.00256	0.008481	10.48581	0.144	8.649			
13	4.568	11.168	0.162	0.456	0.355263	0.00161	0.009334	10.40475	0.256	8.971			
14	4.984	11.323	0.155	0.416	0.372596	0.00075	0.010107	10.19343	0.48	9.357			
15	5.798	11.593	0.27	0.814	0.331695	-0.00088	0.011572	########	0.928	9.754			
16	6.31	11.732	0.139	0.512	0.271484	-0.00189	0.01248	########	1.824	10.184			
17	Среднее							10.49006	2.824	10.561			
18									3.582	10.819			
19				V	%				4.112	11.006			
20				4.694	87.64659	pKa	10.49006		4.568	11.168			
21									4.984	11.323			
22									5.38	11.461			
23									5.798	11.593			
24									6.31	11.732			
25									6.964	11.867			
26									7.964	12.023			
27									8.964	12.146			
28									9.964	12.241			
29									10.964	12.319			
30									11.964	12.385			
31									12.964	12.442			
32									13.964	12.492			
33									14.964	12.533			
34									14.998	12.55			
35									14.999	12.557			
36									15	12.565			
37													
38													

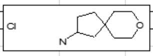

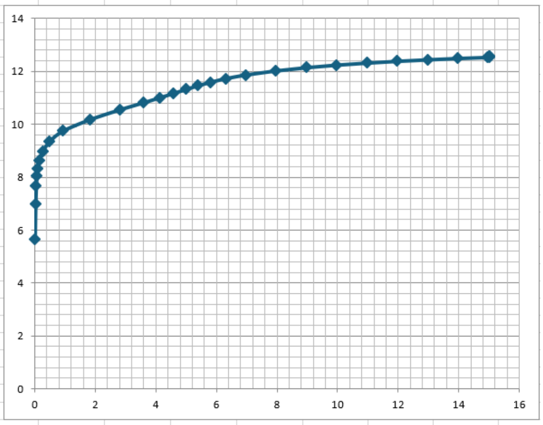

412-757 | 72-962 | 333-014 | 033-619 | 82-291 | 1373-791 | 1162-974 | 1693-939

6. Analysis of Aqueous Solubility

Test articles (**30**, **31**, **32**, and **33)**, and a reference compound (Ondansetron) were assessed for kinetic solubility in phosphate-buffered saline, pH 7.4.

Reagents and consumables

Phosphate buffered saline, pH 7.4 (Sigma-Aldrich, USA; Cat #P3813)
Acetonitrile Chromasolv, gradient grade, for HPLC, ≥99.9% (Sigma-Aldrich, USA; Cat #34851)
Methanol, for HPLC, ≥99.9% (Sigma-Aldrich, Cat #34860)
Ondansetron base powder (Enamine, Ukraine, Cat # EN300-117273)
Verapamil hydrochloride (Sigma Aldrich, USA; Cat #V4629)
DMSO (Sigma-Aldrich, USA; Cat # 34869)
Costar 96 Well Assay Blocks (Corning, USA; Cat # 3958)
MultiScreen HTS 96 Well Filter Plates (Millipore, Ireland; Cat # MSSLBPC10)
UV-Star® 96 Well Microplate (Greiner Bio-One, Germany; Cat #655801)
Matrix Disposable pipette tips (ThermoScientific, USA; Cat ## 8041, 7622, 7321)
Flex-Tubes Microcentrifuge Tubes, 1.5 mL (Eppendorf, Germany; Cat # 22364111)
Matrix Storage tubes, 1.4 mL (ThermoScientific, USA; Cat # 4247)
Agilent InfinityLab Poroshell 120 EC-C18, 2.1 × 50 mm, 4 μm (Cat #699770-902)

Equipment

Water purification system Millipore Milli-Q Gradient A10 (Sartorius Arium™ Mini)
Thermomixer R Block, 1.5 mL (Eppendorf, Germany; Cat # 5355)
Matrix Multichannel Electronic Pipette 2-125 μL, 5-250 μL, 15-1250 μL (Thermo Scientific, USA; Cat ## 2011, 2012, 2004)
MS/MS detector API 3000 PE with TurboIonSpray Electrospray module (PE Sciex, Canada)
SpectraMax Paradigm™ Reader (Multi-Mode Detection Platform, Product # 33270-1279)
Multi-Well Plate Vacuum Manifold (Pall Corporation, USA; Product # 5014)
Vacuum pump (Millipore, USA; Model # XX5500000)

Analytical System

The photometric measurements were performed using a SpectraMax Paradigm reader in UV-Vis mode. The data were acquired and analyzed using SoftMax Pro v.5.4 (Molecular Devices) and Excel 2010 data analysis software. The measurements for two test articles (**32** and **33**) were performed using the Shimadzu Prominence HPLC system including a vacuum degasser, gradient pumps, reverse phase column, column oven, and autosampler. The HPLC system was coupled with a tandem mass spectrometer API 3000 (PE Sciex). The data were acquired and analyzed using Analyst 1.6.3 software (PE Sciex).

Methods

Kinetic solubility assay was performed according to Enamine's aqueous solubility SOP. Briefly, using a 20 mM stock solution of the compound in 100% DMSO dilutions were prepared to a theoretical concentration of 400 µM in duplicates in phosphate-buffered saline pH 7.4 (138 mM NaCl, 2.7 mM KCl, 10 mM K-phosphate) with 2% final DMSO. The experimental compound dilutions in PBS were further allowed to equilibrate at 25 °C on a thermostatic shaker for two hours and then filtered through HTS filter plates using a vacuum manifold. The filtrates of test compounds were diluted 2-fold with acetonitrile with 2% DMSO before measuring.

In parallel, using a 20 mM stock solution of the compound in 100% DMSO dilutions were prepared to theoretical concentrations of 0 µM (blank), 10 µM, 25 µM, 50 µM, 100 µM, and 200 µM (1 µM, 10 µM, 25 µM, 50 µM, 100 µM, and 200 µM for test articles) in 50% acetonitrile/PBS with 2% final DMSO to generate calibration curves. Ondansetron was used as a reference compound to control proper assay performance. 200 µL of each sample was transferred to a 96-well plate and measured in the 230-550 nm range with a 5 nm step.

The concentrations of compounds in PBS filtrate are Calcdulated using a dedicated Microsoft Excel Calcdulation script. Proper absorbance wavelengths for Calcdulations are selected for each compound manually based on absorbance maximums (absolute absorbance unit values for the minimum and maximum concentration points within the 0 – 3 OD range). Each final dataset is visually evaluated by the operator, and goodness of fit (R^2) is Calcdulated for each calibration curve.

For test articles, the calibration solutions and incubation samples were diluted 20-fold with acetonitrile containing internal standard and were analyzed using the HPLC system coupled with a tandem mass spectrometer. The effective range of this assay is approximately 2-400 µM (1-400 µM for four test articles) and the compounds returning

values close to the upper limit of the range may have higher actual solubility (e.g. 5'-deoxy-5-fluorouridine).

Results

The solubility data of the test and reference compounds are listed in the table **S1** below. The calibration curves are shown in Appendix*.

Table S1. The solubility data

Compound ID	PBS solubility, pH 7.4, µM			SE
	Incubation 1	Incubation 2	Mean	
Ondansetron	118	116	**117****	1.1
**30**	424	424	**≥400**	-
**31**	425	425	**≥400**	-
**32**	384	386	**385**	0.5
**33**	401	374	**388**	6.8

*Goodness of fit (R^2) in all titration curves as well as the variations between repeat measurements indicates a high quality of the experimental data in the current batch of test articles.

**Ondansetron solubility data are consistent with those previously obtained.

APPENDIX

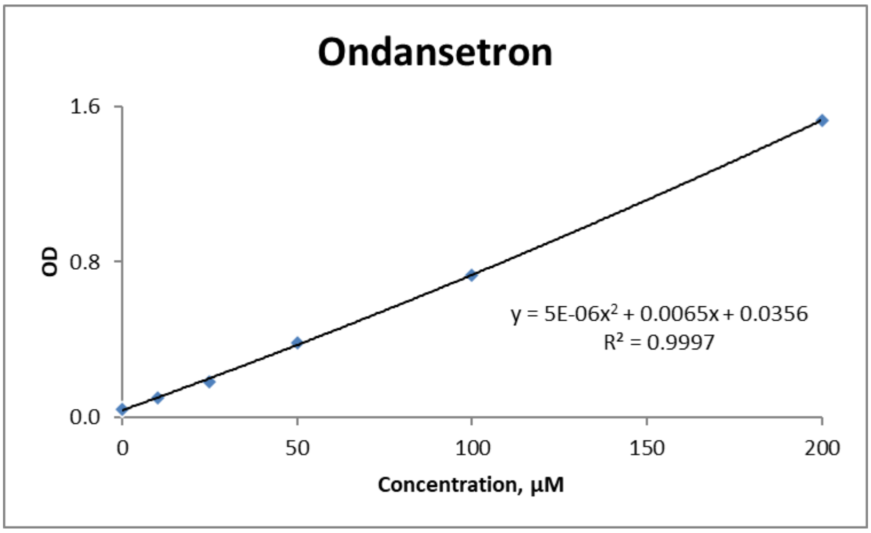

Figure S1. Calibration curve for **Ondansetron**.

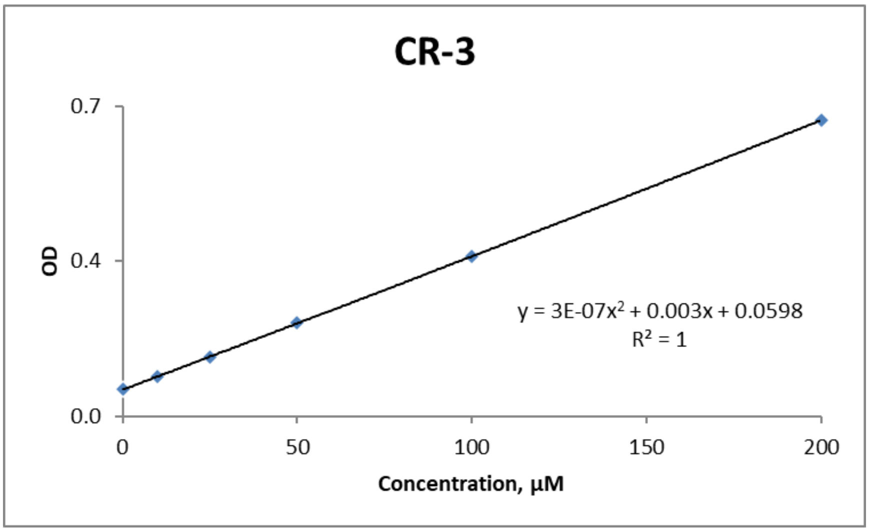

Figure S2. Calibration curve for compound **30**.

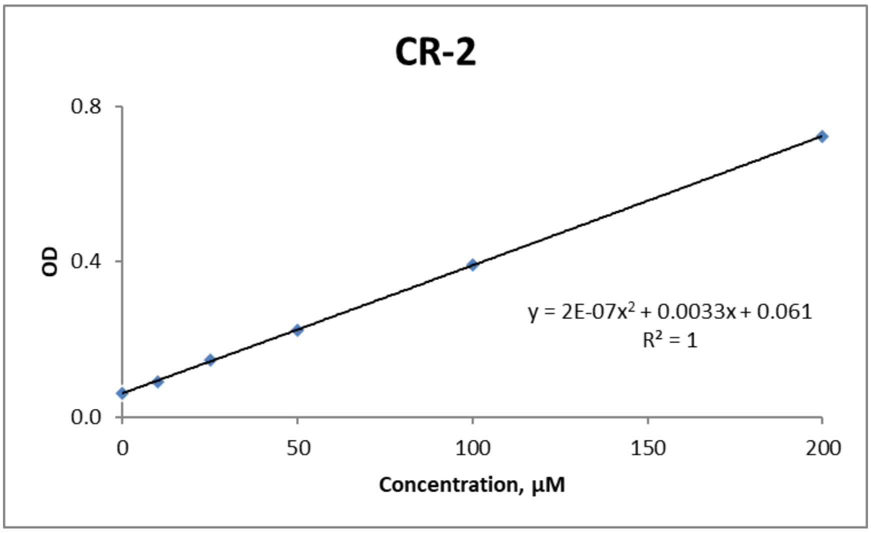

Figure S3. Calibration curve for compound **31**.

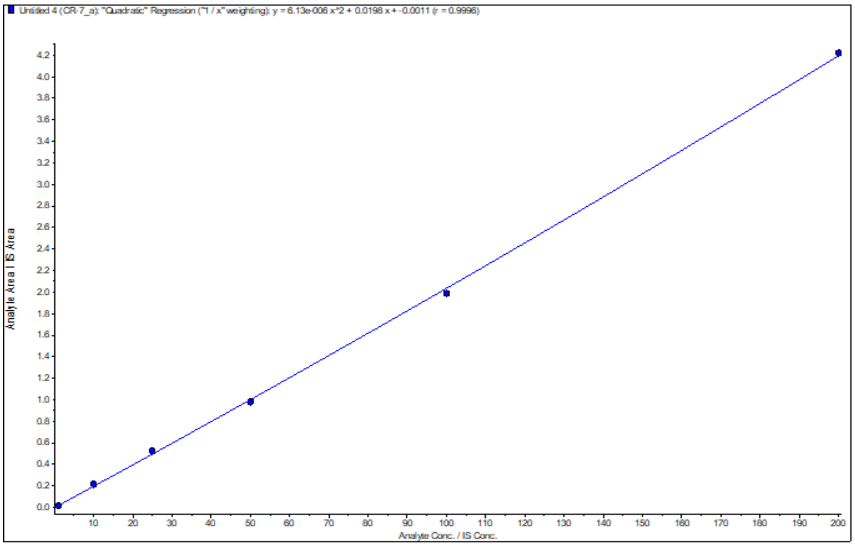

Figure S4. Calibration curve for compound **32**.

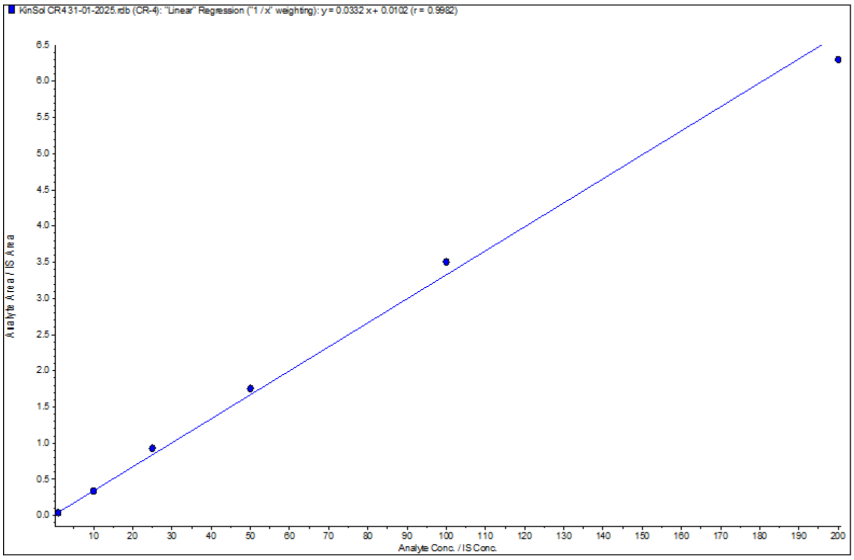

Figure S5. Calibration curve for compound **33**.

7. Determination of Distribution Coefficient (LogP)

The aim of this study was to determine distribution coefficients for the test articles **30**, **31**, **32**, and **33**, and reference compound (Mebendazole) in *n*-octanol – H_2O. The distribution coefficient (or LogP) is a logarithm of the ratio of drug concentrations in two immiscible solvents, water and n-octanol. It is a measure of the hydrophobic/hydrophilic properties of a given molecule. The partition of test compounds is determined using the shake-flask method, which involves mixing a certain amount of the solute of interest in defined volumes of n-octanol and an aqueous buffer of choice followed by equilibration of the mixture by incubation with efficient mixing. Then, the distribution of the compounds in each solvent was controlled using LC-MS/MS.

Reagents and consumables

DMSO Chromasolv Plus, HPLC grade, ≥99.7% (Sigma-Aldrich, USA; Cat #34869)
Acetonitrile Chromasolv, gradient grade, for HPLC, ≥99.9% (Sigma-Aldrich, USA; Cat #34851)
Formic acid for mass spectrometry, ~98% (Fluka, USA; Cat #94318)
1-Octanol ACS grade, ≥99% (Sigma-Aldrich, USA; Cat # 472328)
Mebendazole analytical standard, ≥ 98%, HPLC (Sigma-Aldrich, USA; Cat # M2523)
DMSO stock solutions of the test compounds 10 mM
InfinityLab Poroshell 120 EC-C18, 2.1 × 50 mm, 4 µm (Cat#699770-902)
1.4 mL microtubes in microracks, pipettor tips (Thermo Scientific, USA).
National Scientific MicroTube™ Rack (Thermo Fisher Scientific, USA; Cat # TN094612R)

Equipment

Agilent 1290 Infinity II (Agilent, USA)
Triple quadrupole mass-detector 4000 with Turbo V ion source (AB Sciex, Canada)
VWR Membrane Nitrogen Generators N2-04-L1466, nitrogen purity 99%+ (VWR, USA)
MTR22 Multi Mix Rotator (UNICO, USA)
Laboratory Centrifuge, Sigma 4-15C, Qiagen (SIGMA GmbH, Germany)

Water purification system Millipore Milli-Q Gradient A10 (Millipore, France)

Multichannel Electronic Pipettes 2-125 μL, 5-250 μL, 15-1250 μL, Matrix (Thermo Scientific, USA; Cat ## 2001, 2002, 2004)

Analytical System

All measurements were performed using the Agilent 1290 Infinity II HPLC system including vacuum degasser, gradient pumps, reverse phase column, column oven, and autosampler. Mass spectrometric analysis was performed using an API 4000 mass spectrometer from Applied Biosystems/ MDS Sciex (AB Sciex) with a Turbo V ion source. The Turbo V ion source was used in both positive and negative ion modes. Acquisition and analysis of the data were performed using Analyst 1.6.3 software.

Methods

Incubations were carried out in Eppendorf-type polypropylene microtubes in triplicates. A 2.5 μL aliquot of 20 mM DMSO stock of a test compound was added into the previously mutually saturated mixture containing 500 μL of H_2O and 500 μL of *n*-octanol. The solution was allowed to mix in a rotator for 2 hours at 30 rpm. Phase separation was assured by centrifugation for 2 min at 6000 rpm. The octanol phase was diluted 100-fold with 40% acetonitrile, and the aqueous phase was analyzed without dilution. The samples (both phases) were analyzed using an HPLC system coupled with a tandem mass spectrometer. Mebendazole was used as a reference compound.

Calcdulations of the partition ratios were carried out using the equation below.

$$P = \frac{p_o \cdot S_O}{p_p \cdot S_P}$$

where: S_O– peak area of the analyte in octanol phase
S_P– peak area of the analyte in water
p_o – dilution coefficient for octanol phase
p_p – dilution coefficient for aqueous phase

Results

LogP data for the reference compound (Mebendazole) and test compounds is provided in the table **S2** below.

Table S2. Experimental LogP

Compound ID	Incuba-tion	S_P	S_O	P	LogP	
Mebendazole	1	4.75E+04	3.66E+05	7.71E+02	2.89	2.9
	2	5.08E+04	3.72E+05	7.32E+02	2.86	
	3	4.75E+04	3.88E+05	8.17E+02	2.91	
**30**	1	4.26E+05	1.00E+05	2.35E+01	1.37	**1.4**
	2	4.24E+05	8.84E+04	2.08E+01	1.32	
	3	4.41E+05	9.73E+04	2.21E+01	1.34	
**31**	1	4.78E+04	1.13E+05	2.36E+02	2.37	**2.4**
	2	4.20E+04	1.13E+05	2.69E+02	2.43	
	3	4.01E+04	1.25E+05	3.12E+02	2.49	
**32**	1	3.64E+05	4.67E+04	1.28E+01	1.11	**1.1**
	2	3.72E+05	4.33E+04	1.16E+01	1.07	
	3	3.93E+05	4.82E+04	1.23E+01	1.09	
**33**	1	1.37E+05	1.35E+05	9.85E+01	1.99	**2.0**
	2	1.42E+05	1.48E+05	1.04E+02	2.02	
	3	1.33E+05	1.49E+05	1.12E+02	2.05	

*Reliable measurable range is approximately -1 to 4.5.

8. Assessment of Metabolic Stability in Human Liver Microsomes

The objective of this study was to determine metabolic stability of 4 test articles (**30**, **31**, **32**, and **33**) and reference compounds in human liver microsomes at five time points over 40 minutes using HPLC-MS. Metabolic stability is defined as the percentage of parent compound lost over time in the presence of a metabolically active test system.

Reagents and consumables

DMSO Chromasolv Plus, HPLC grade, ≥99.7% (Sigma-Aldrich, USA; Cat# 34869)
Acetonitrile Chromasolv, gradient grade, for HPLC, ≥99.9% (Sigma-Aldrich, USA; Cat# 34851)
Methanol, HiPerSolv, HPLC-gradient grade, ≥99.9% (VWR Chemicals, USA, Cat# 20864.320)
Potassium phosphate monobasic (Bio-Basic, Canada; Lot #N9016010)
Potassium phosphate dibasic (Bio-Basic, Canada; Lot #MA7100050)
Magnesium chloride hexahydrate (Santa Cruz Biotechnology, Inc., USA; sc-203126A)
Human Liver Microsomes: pooled, mixed gender (XenoTech, H0630/lot N#2310056)
Glucose-6-phosphate dehydrogenase from baker's yeast, type XV (Sigma-Aldrich, USA; Cat #G6378)
D-Glucose-6-phosphate monosodium salt (EMD Milipore Corp., USA; Cat #346764-5GM)
NADPH tetrasodium salt (BLD Pharmatech Ltd., Cat #BD116582)
Formic acid (Sigma-Aldrich, 94318)
Verapamil hydrochloride (Sigma Aldrich, USA; Cat #V4629)
Niclosamide (Sigma-Aldrich, USA; Cat #N3510)
DMSO stock solutions of the tested compounds 20mM
(+,-) Propranolol hydrochloride (Sigma-Aldrich, P0884)
Diclofenac sodium salt (Sigma-Aldrich, D6899)
InfinityLab Poroshell 120 EC-C18, 2.1 × 50 mm, 4 μm (Cat#699770-902)
Matrix™ 0.75 mL blank tubes (Cat #4170), pipettor tips (Thermo Scientific).

Equipment

Gradient HPLC system (Agilent Technologies)
Triple quadrupole mass-detector API 3000 with TurboIonSpray Ion Source (AB Sciex, Canada)

Nitrogen generator N2-04-L1466, nitrogen purity 99%+ (Whatman)
Environmental Incubator Shaker G24; Digital Refrigerated Incubator/Shaker Innova 4330 (New Brunswick Scientific)
Water purification system Millipore Milli-Q Gradient A10 (Millipore, France)
Multichannel pipettors 1-30 µL, 2-125 µL, 30-850 µL (Thermo Scientific)

Analytical System

All measurements were performed using Agilent HPLC system including vacuum degasser, gradient pumps, reverse phase HPLC column, column oven, and autosampler. Mass spectrometric analysis was performed using an API 3000 mass spectrometer from AB Sciex with TurboIonspray interface. The TurboIonSpray ion source was used in both positive and negative ion modes. The data acquisition and system control were performed using Analyst 1.6.3 software from AB Sciex.

Methods

Microsomal incubations were carried out in 96-well plates in 5 aliquots of 30 µL each (one for each time point). Liver microsomal incubation medium comprised of phosphate buffer (100 mM, pH 7.4), $MgCl_2$ (3.3 mM), NADPH (3 mM), glucose-6-phosphate (5.3 mM), glucose-6-phosphate dehydrogenase (0.67 units/mL) with 0.42 mg of liver microsomal protein per mL. In the control reactions, the NADPH-cofactor system was substituted with phosphate buffer. Test compounds (2 µM, final acetonitrile concentration 1.6%) were incubated with microsomes at 37°C, shaking at 100 rpm. Five time points over 40 minutes were analyzed. The reactions were stopped by adding

5 volumes of acetonitrile with internal standard to incubation aliquots, followed by protein sedimentation by centrifuging at 5500 rpm for 5 minutes. Each reaction was performed in duplicates. Supernatants were analyzed using the HPLC system coupled with a tandem mass spectrometer.

The elimination constant (k_{el}), half-life ($t_{1/2}$), and intrinsic clearance (Cl_{int}) were determined in a plot of ln(AUC) versus time, using linear regression analysis:[1]

$$k_{el} = -slope \qquad t_{1/2} = \frac{0.693}{k} \qquad Cl_{\text{int}} = \frac{0.693}{t_{1/2}} \times \frac{\mu l_{incubation}}{mg_{microsomes}}$$

[1] In order to indicate the quality of the linear regression analysis, the R^2 (determination coefficient) values are provided. In some cases, the last time point is excluded from the calculations to ensure acceptable logarithmic linearity of decay.

Results

Human microsomal stability data for reference and test compounds are provided in the table **S3** below.

Table S3. Human microsomal stability

Compound ID	Time, min	Analyte Peak Area		Analyte Peak Area, Mean of 2	% Remaining, Mean of 2	R^2	k_{el}, min^{-1}	$t_{1/2}$, min	Cl_{int}, µl/min/mg	% Remaining without cofactor, Mean of 2
		Inc. 1	Inc. 2							
Diclofenac human	0	5.03E-01	4.54E-01	4.79E-01	100	0.987	0.084	8.3	202	100
	7	3.14E-01	3.86E-01	3.50E-01	73	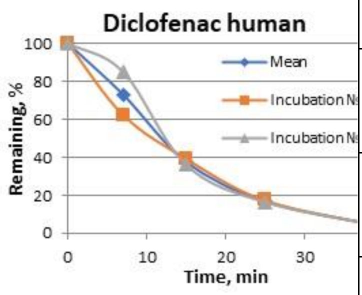				
	15	1.98E-01	1.64E-01	1.81E-01	38					
	25	8.79E-02	7.51E-02	8.15E-02	17					
	40	1.88E-02	1.64E-02	1.76E-02	4					120
Propranolol human	0	1.22E-01	1.10E-01	1.16E-01	100	0.899	0.010	69.6	24	100
	7	1.02E-01	1.01E-01	1.02E-01	88	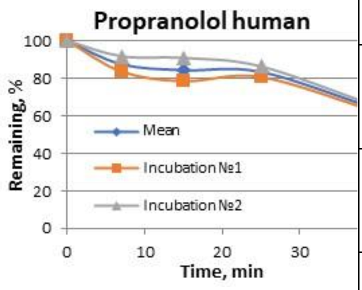				
	15	9.56E-02	1.00E-01	9.78E-02	84					
	25	9.84E-02	9.51E-02	9.68E-02	83					
	40	7.57E-02	7.18E-02	7.38E-02	64					114
**30 human**	0	5.76E-01	5.51E-01	5.64E-01	100	0.452	0.002*	291.3*	6*	100
	7	5.45E-01	4.75E-01	5.10E-01	91					
	15	5.36E-01	5.18E-01	5.27E-01	94					

Compound ID	Time, min	Analyte Peak Area		Analyte Peak Area, Mean of 2	% Re-maining, Mean of 2	R^2	k_{el}, min^{-1}	$t_{1/2}$, min	Cl_{int}, µl/min/mg	% Remaining without cofactor, Mean of 2
		Inc. 1	Inc. 2							
	25	4.82E-01	4.89E-01	4.85E-01	86	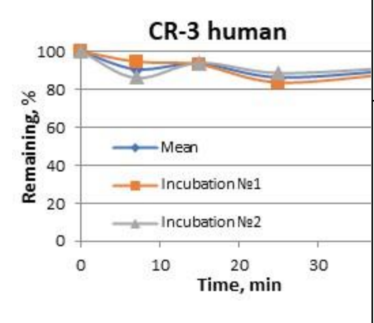				
	40	5.09E-01	5.04E-01	5.06E-01	90					101

**31 human**	0	1.21E-01	1.21E-01	1.21E-01	100	0.709	0.004*	189.7*	9*	100
	7	1.23E-01	1.23E-01	1.23E-01	102	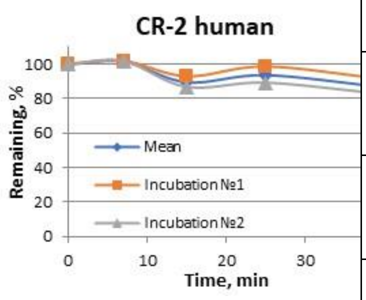				
	15	1.12E-01	1.05E-01	1.08E-01	90					
	25	1.19E-01	1.08E-01	1.13E-01	94					
	40	1.10E-01	1.00E-01	1.05E-01	87					103
**32 human**	0	4.60E-01	4.14E-01	4.37E-01	100	0.448	0.001*	488.8*	3*	100
	7	4.25E-01	4.08E-01	4.16E-01	95	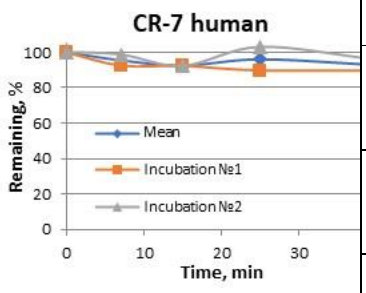				
	15	4.24E-01	3.81E-01	4.02E-01	92					
	25	4.12E-01	4.26E-01	4.19E-01	96					
	40	4.12E-01	3.96E-01	4.04E-01	92					108
**33 human**	0	2.56E-01	2.71E-01	2.64E-01	100	0.672	0.002*	444.4*	4*	100
	7	2.62E-01	2.84E-01	2.73E-01	104	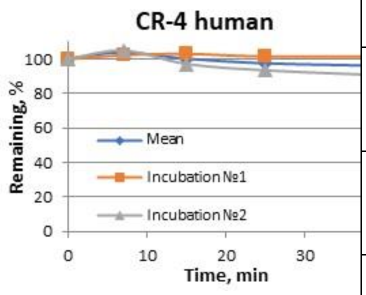				
	15	2.64E-01	2.63E-01	2.64E-01	100					
	25	2.60E-01	2.53E-01	2.57E-01	97					
	40	2.60E-01	2.45E-01	2.53E-01	96					96

*Parameter should be considered as approximate due to the high stability of the compound.

9. Interpretation of microsomal stability assay data

The test compounds can be classified in terms of their microsomal stability into low, medium and high clearance groups. Intrinsic Clearance (*in vitro*) can be reCalcdulated to Intrinsic Clearance (*in vivo*) using literature data for liver weight and liver blood flow with the next equation[S1-S4]:

$$Predicted\ in\ vivo\ CL_{int,u} = \frac{in\ vitro\ CL_{int} \times PBSF \times LW}{f_{u\ mic}\ or\ f_{u\ heps}}$$

where,

$in\ vivo\ CL_{int,u}$ – predicted *in vivo* intrinsic clearance, mL/min/kg

$in\ vitro\ CL_{int}$ – *in vitro* microsomal clearance, mL/min/mg

$PBSF$ – physiologically based scaling factor – the microsomal average recovery factor for microsomal predictions and hepatocellularity for hepatocyte predictions, mg/g

LW – liver weight/kg bodyweight, g/kg

$f_{u\ mic}\ or\ f_{u\ heps}$ – fraction unbound in either microsomes or hepatocytes (can be determined from Plasma Protein Binding study or assumed as 1, if it is unknown)

Using $in\ vivo\ Cl_{int}$ hepatic clearance can be predicted based on a "well-stirred" liver model using the next formula[S2]:

$$CL_H = \frac{Q_H \times f_u \times CL_{int}}{Q_H + f_u \times CL_{int}}$$

where,

CL_H – predicted hepatic clearance, mL/min/kg

Q_H – liver blood flow, mL/min/kg

f_u – fraction unbound in the blood

CL_{int} – predicted *in vivo* clearance, mL/min/kg

[S1] F. L. Wood, J. B. Houston, D. Hallifax. Clearance prediction methodology needs fundamental improvement: Trends common to rat and human hepatocytes/microsomes and implications for experimental methodology. *Drug Metab. Dispos.* **2017**, *45*, 1178-1188.
[S2] T. Laveé, C. Funk. In vivo absorption, distribution, metabolism, and excretion studies in Discovery and Development. *Comprehensive Medicinal Chemistry II*, **2007**, 31.
[S3] Z. E. Barter, M. K. Bayliss, P. H. Beaune, A. R. Boobis, D. J. Carlile, R. J. Edwards, J. B. Houston, B. G. Lake, J. C. Lipscomb, O. R. Pelkonen, G. T. Tucke, A. Rostami-Hodjegan. Scaling factors for the extrapolation of *in vivo* metabolic drug clearance from *in vitro* data: reaching a consensus on values of human microsomal protein and hepatocellularity per gram of liver. *Curr. Drug Metab.* ***2007***, *8*, 33-45.
[S4] T. *Iwatsubo, H. Suzuki, Y. Sugiyama.* Prediction of species differences (rats, dogs, humans) in the *in vivo* metabolic clearance of YM796 by the liver from *in vitro* data. *J. Pharmacol. Exp. Ther.* ***1997***, **283,** 462-469.

SYNTHESIS
OF
1-BROMOALKYNES

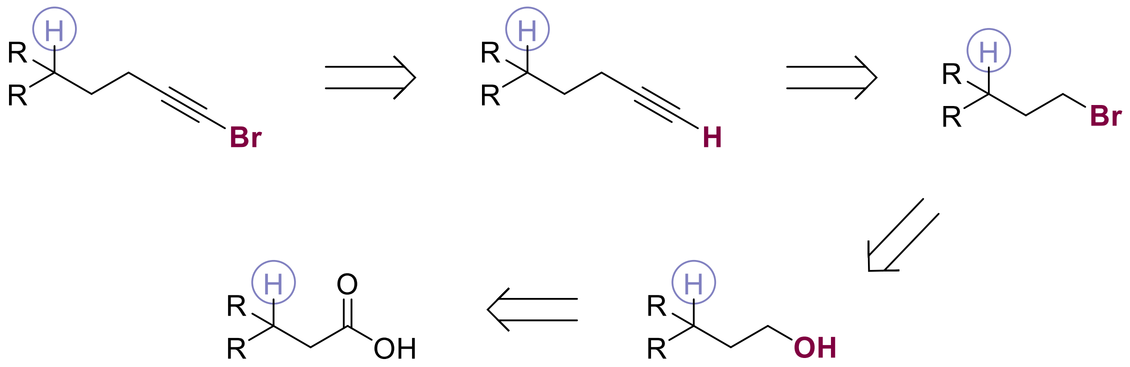

2-(tetrahydro-2H-pyran-2-yl)ethan-1-ol (2-OH)

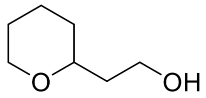

1H NMR CDCl3 300 MHz

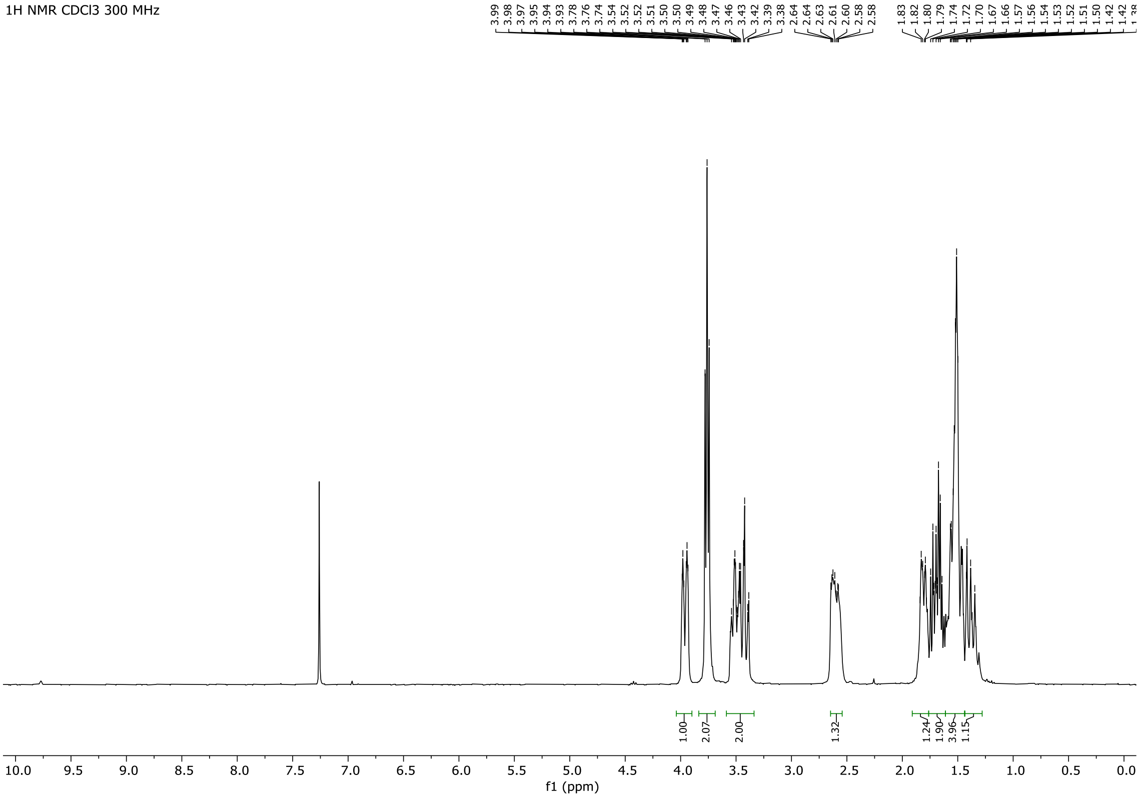

13C NMR CDCl3 300 MHz

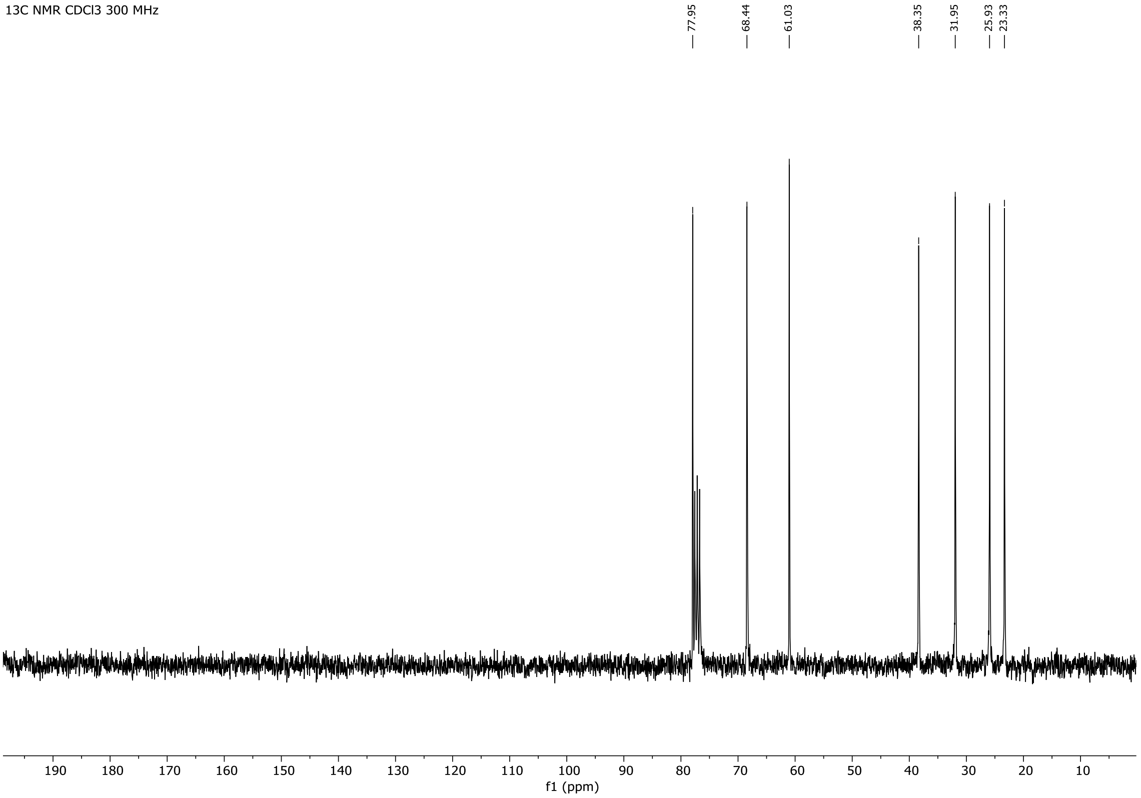

2-(2-bromoethyl)tetrahydro-2H-pyran (2-Br)

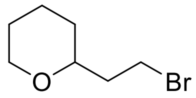

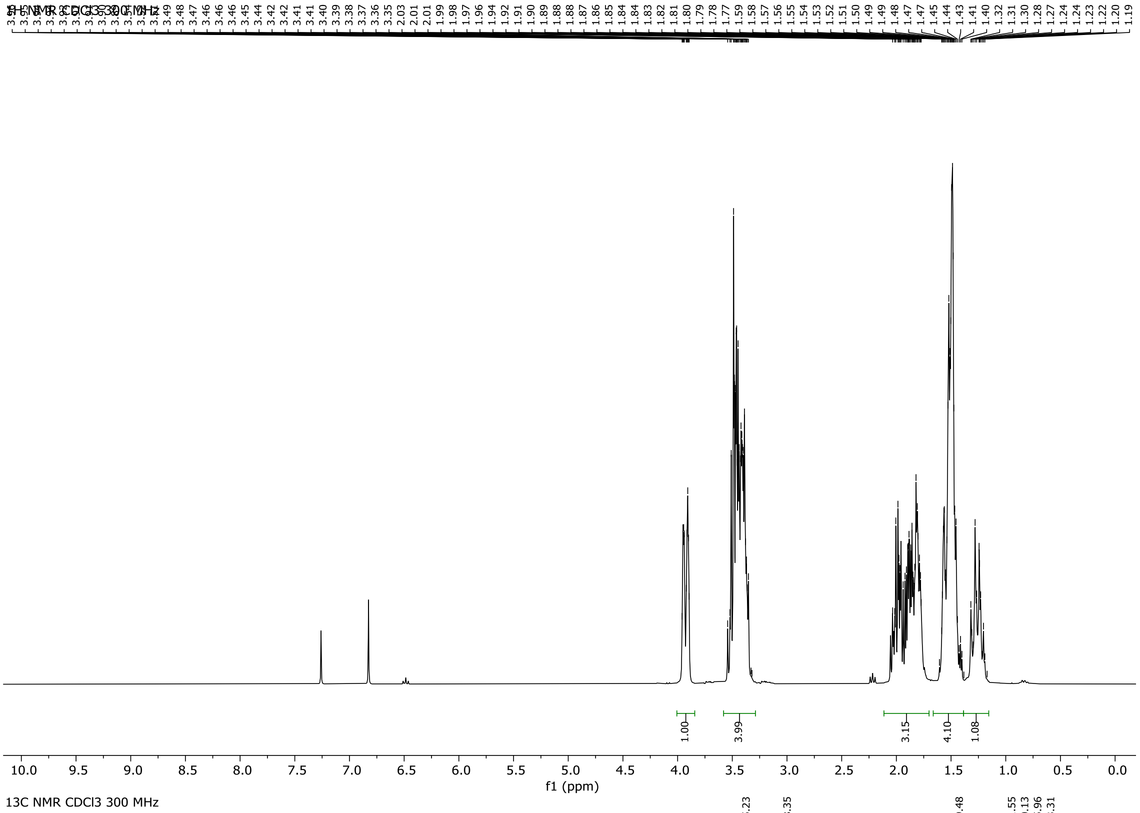

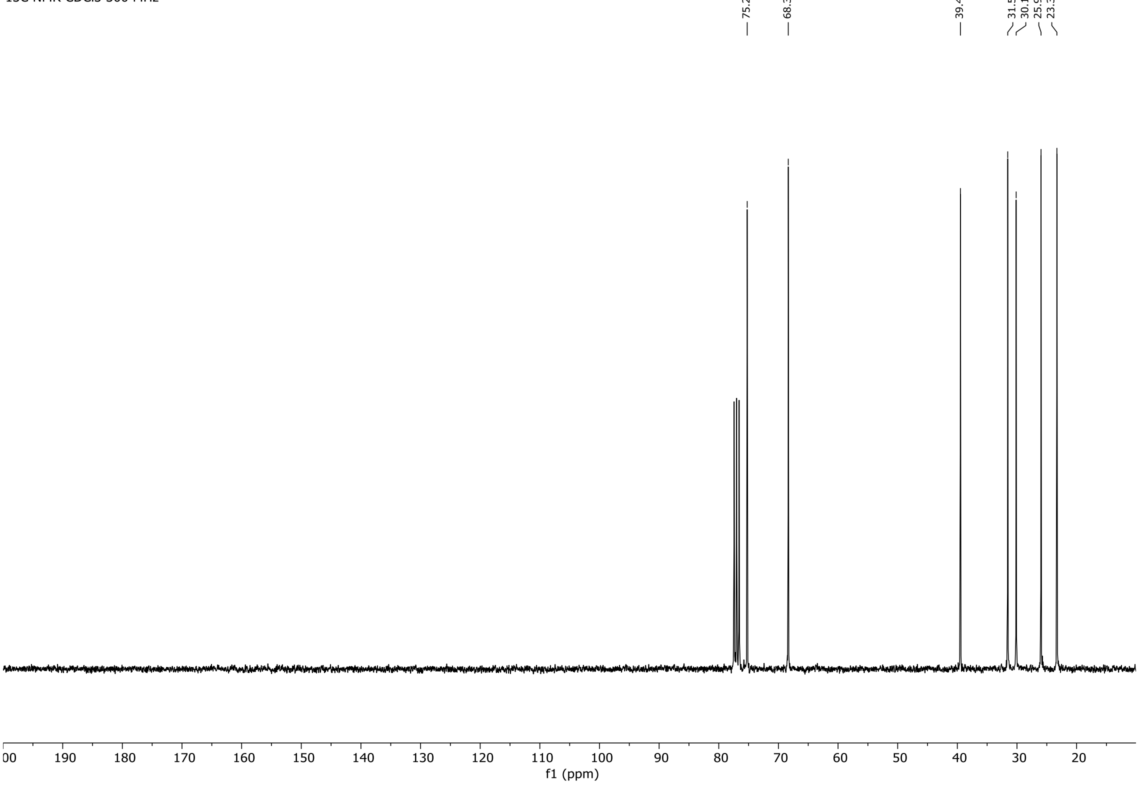

2-(but-3-yn-1-yl)tetrahydro-2H-pyran (2-CCH)

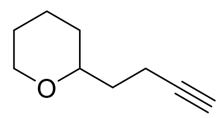

1H NMR CDCl3 300 MHz

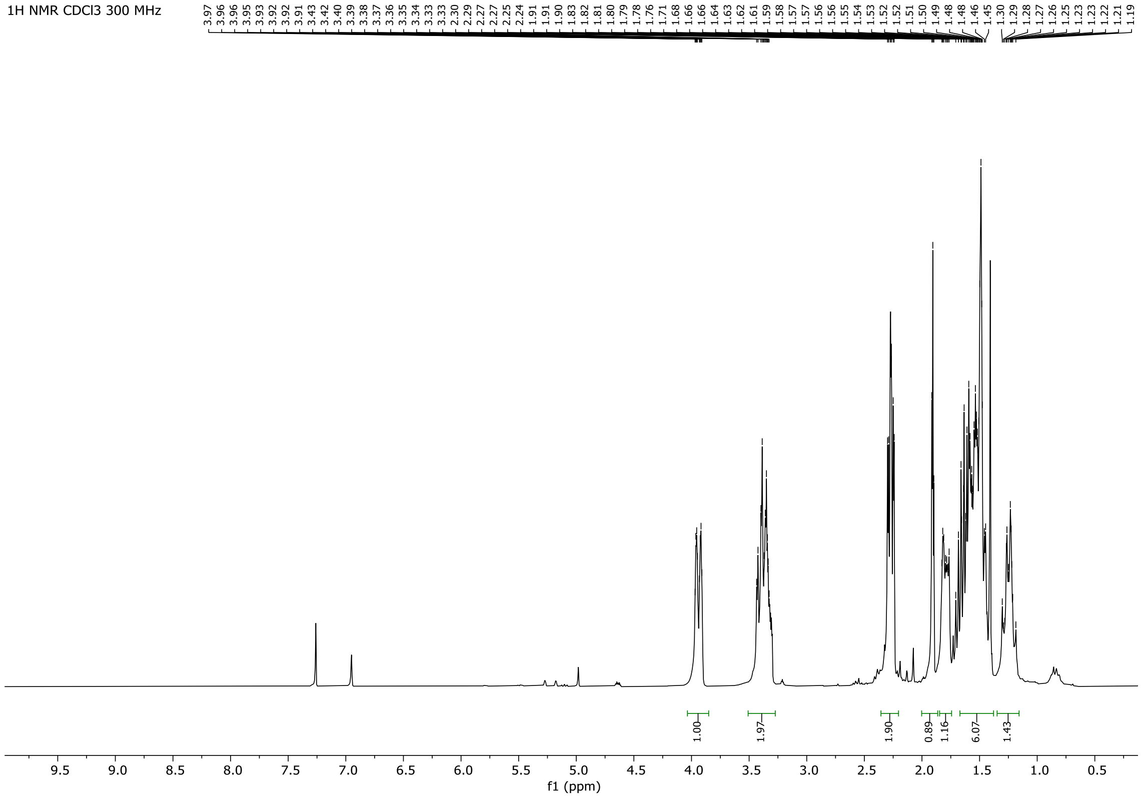

13C NMR CDCl3 300 MHz

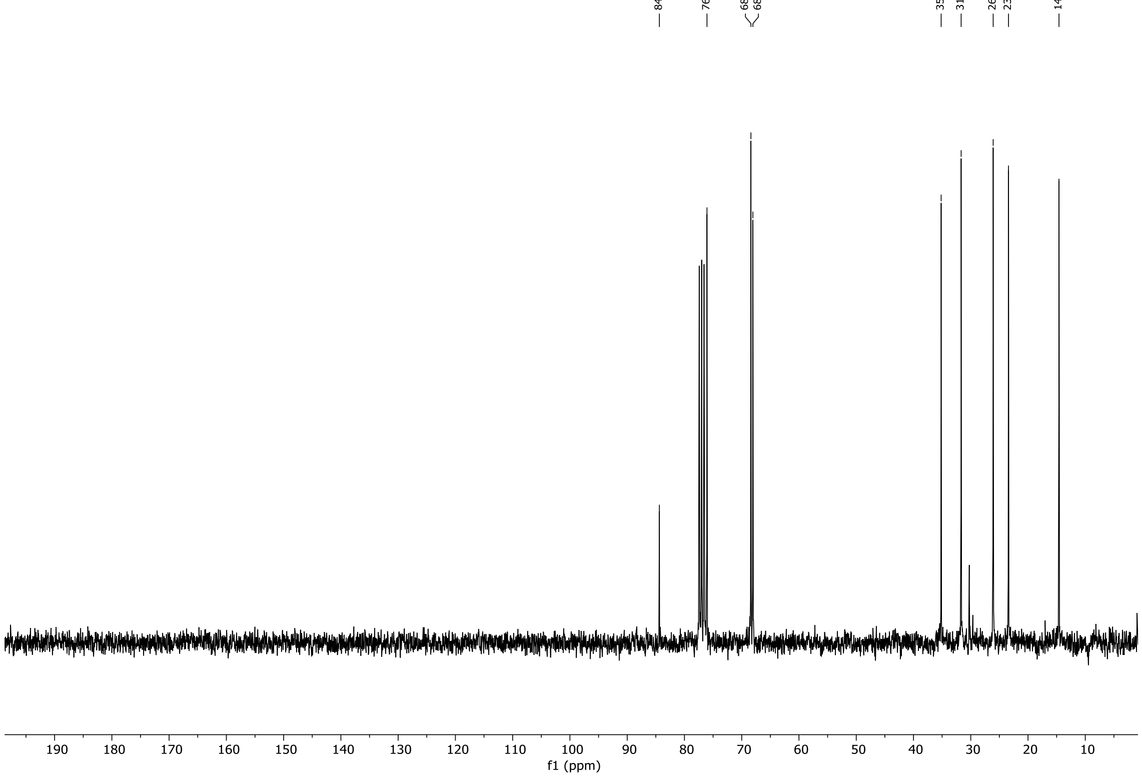

2-(tetrahydro-2H-pyran-3-yl)ethan-1-ol (3-OH)

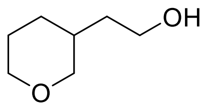

1H NMR CDCl3 300 MHz

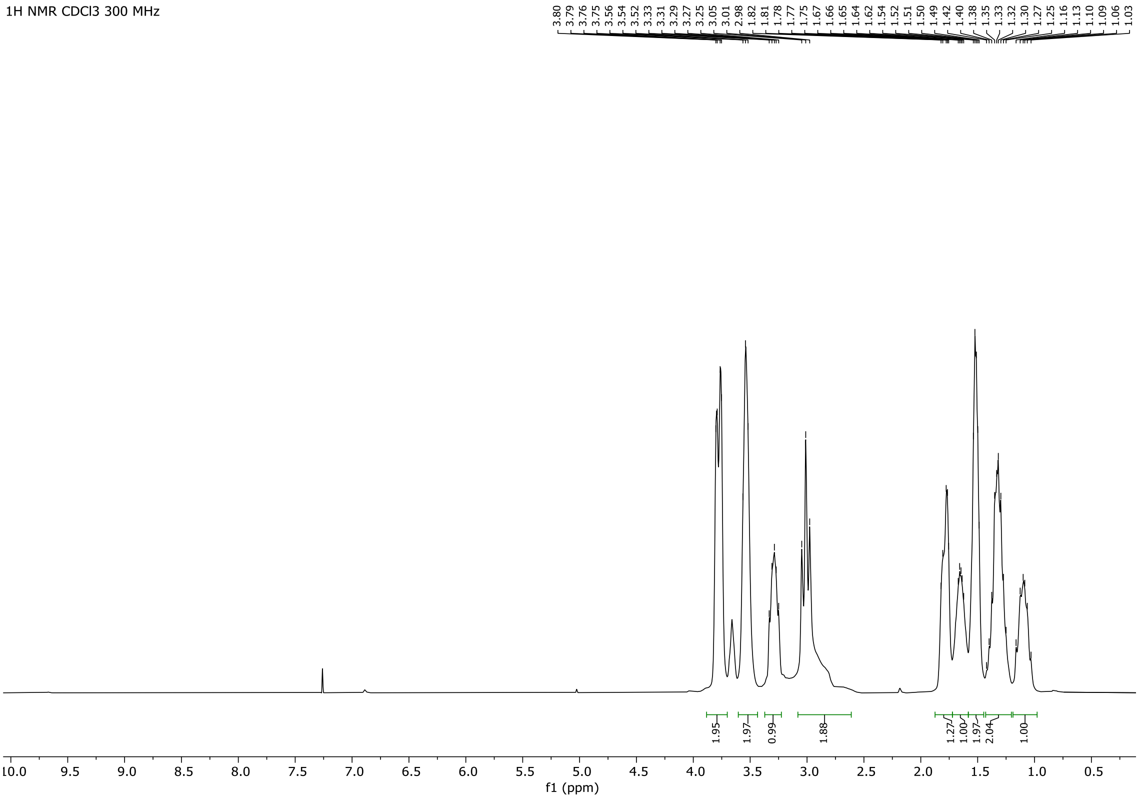

13C NMR CDCl3 300 MHz

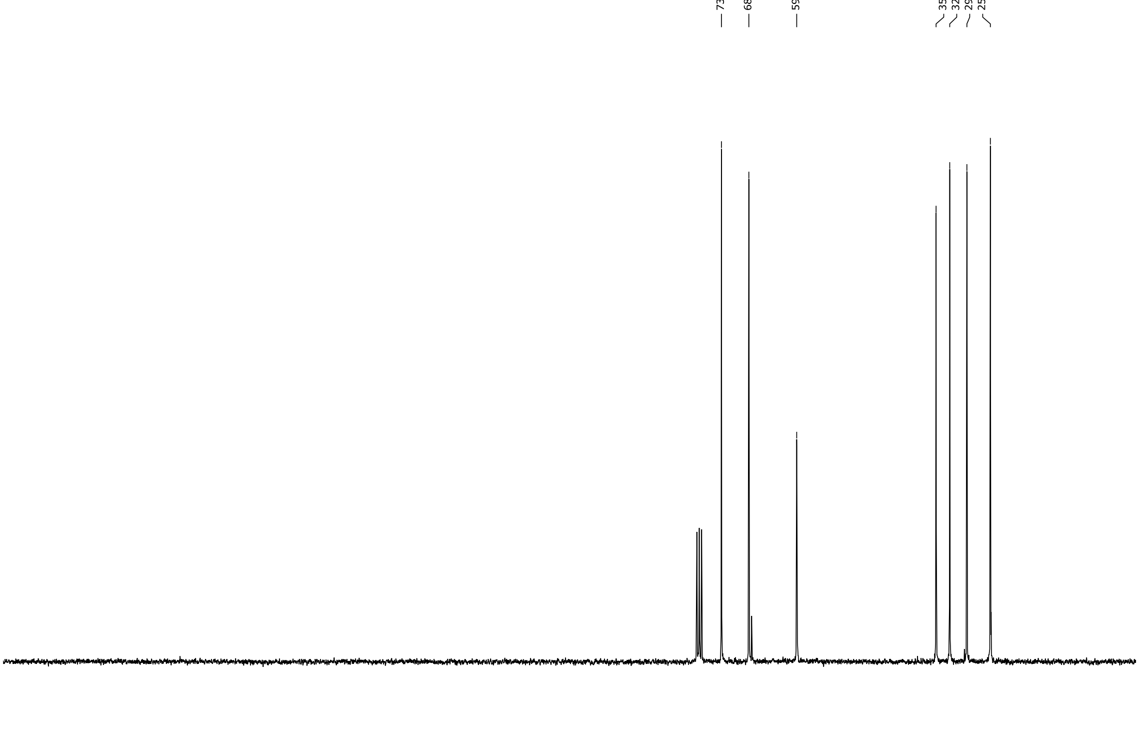

3-(2-bromoethyl)tetrahydro-2H-pyran (3-Br)

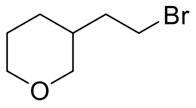

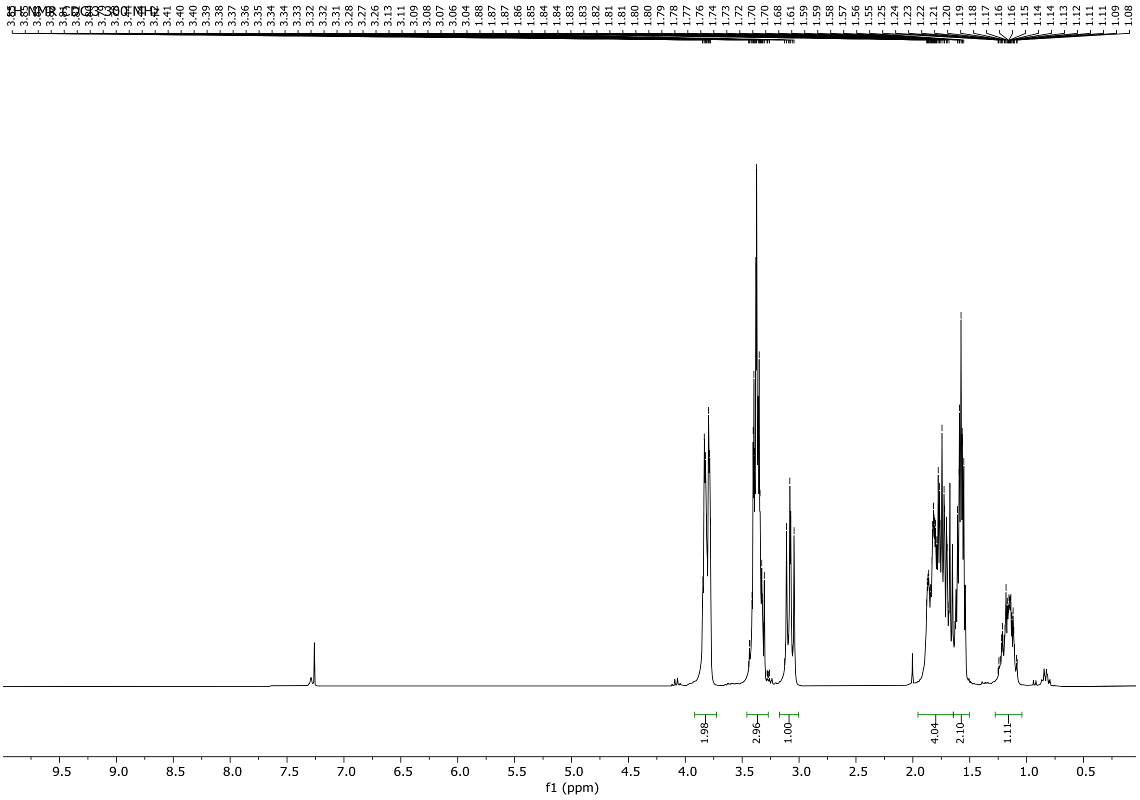

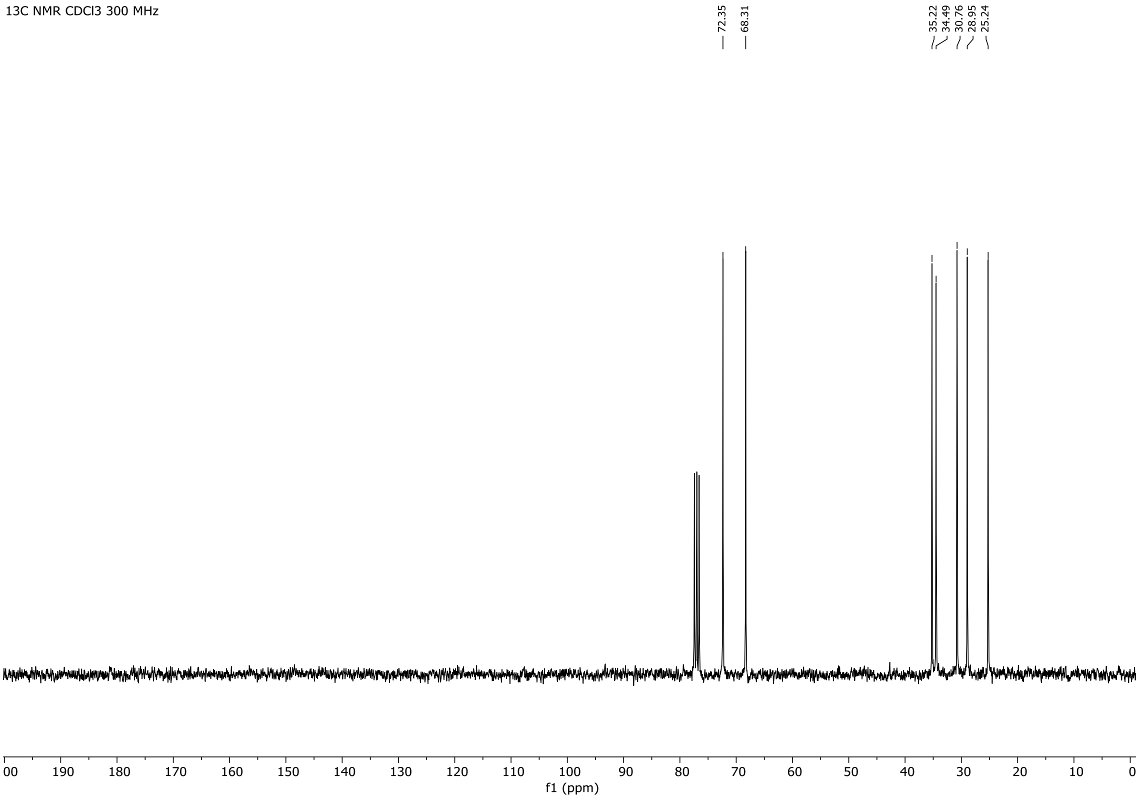

3-(but-3-yn-1-yl)tetrahydro-2H-pyran (3-CCH)

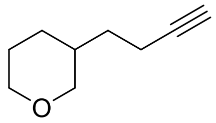

1H NMR CDCl3 300 MHz

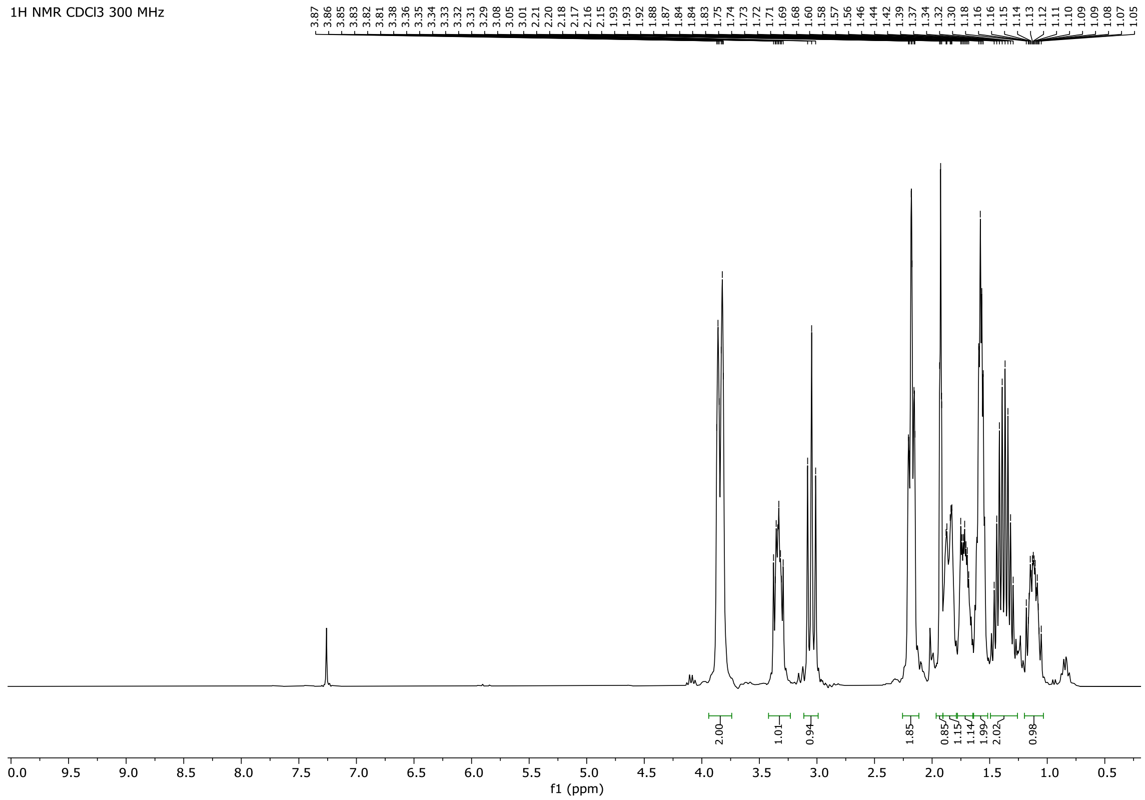

13C NMR CDCl3 300 MHz

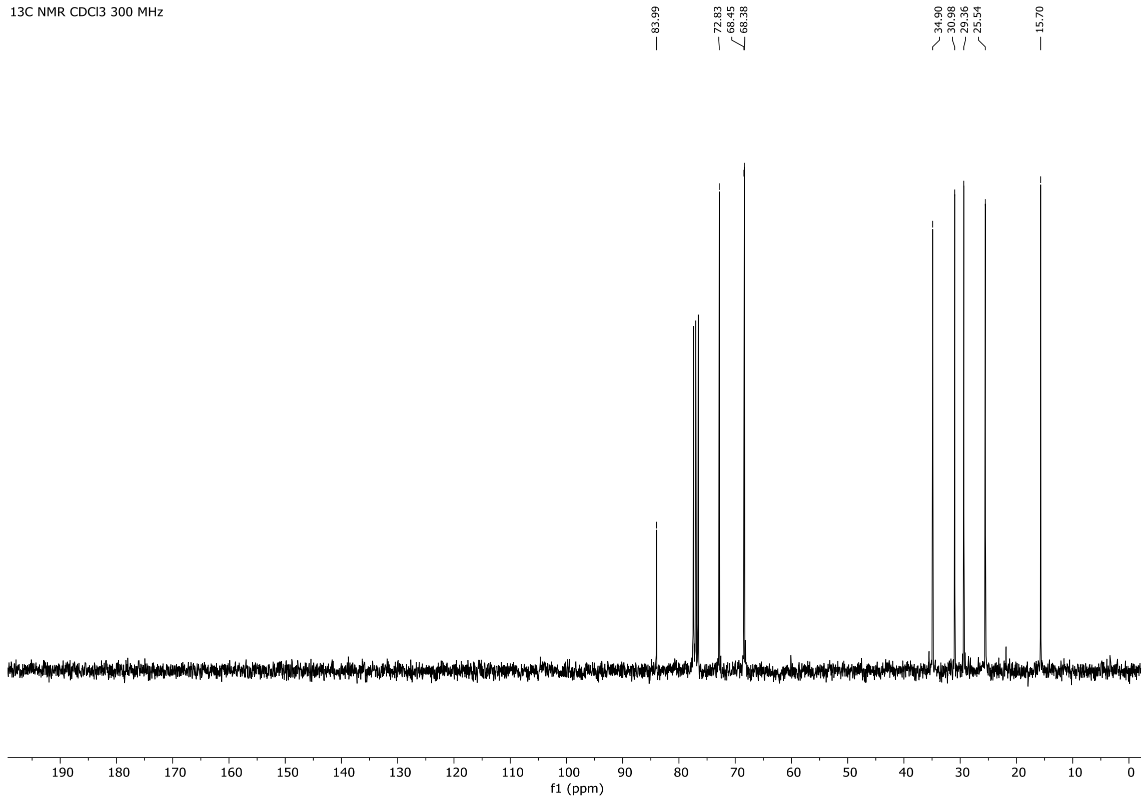

3-(4-bromobut-3-yn-1-yl)tetrahydro-2H-pyran (3)

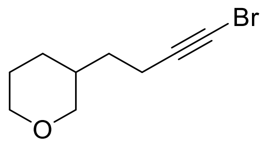

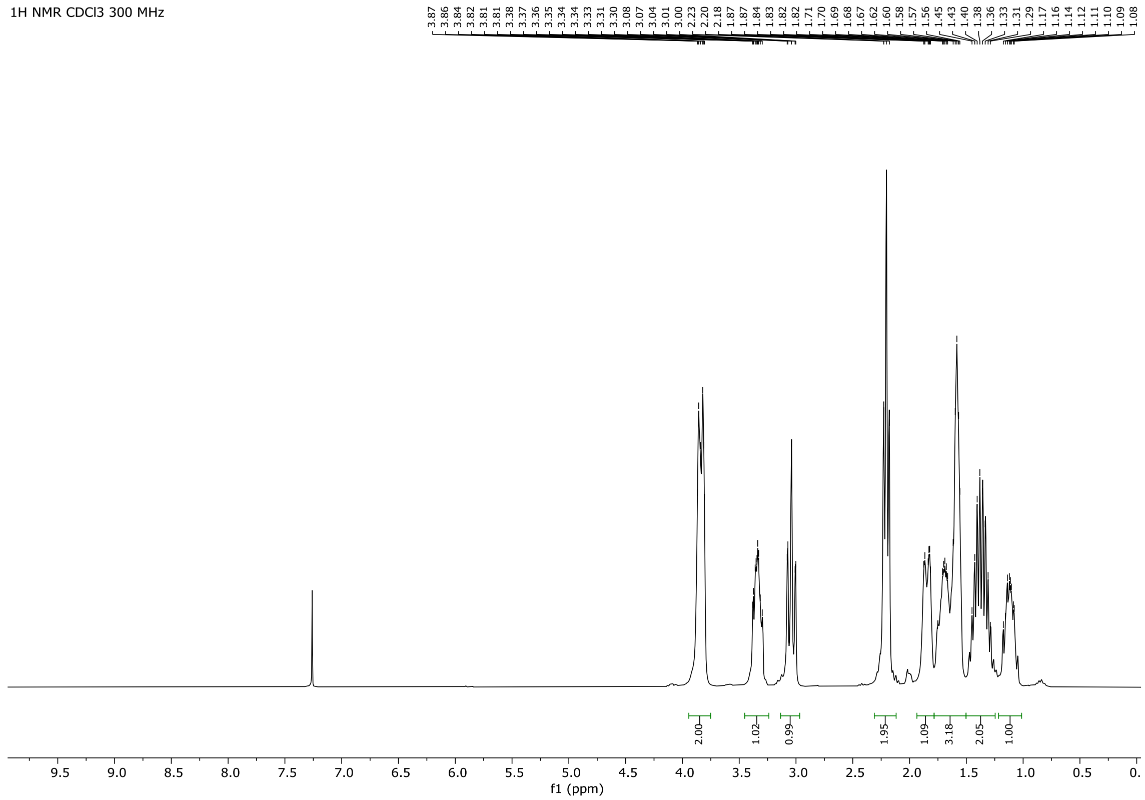

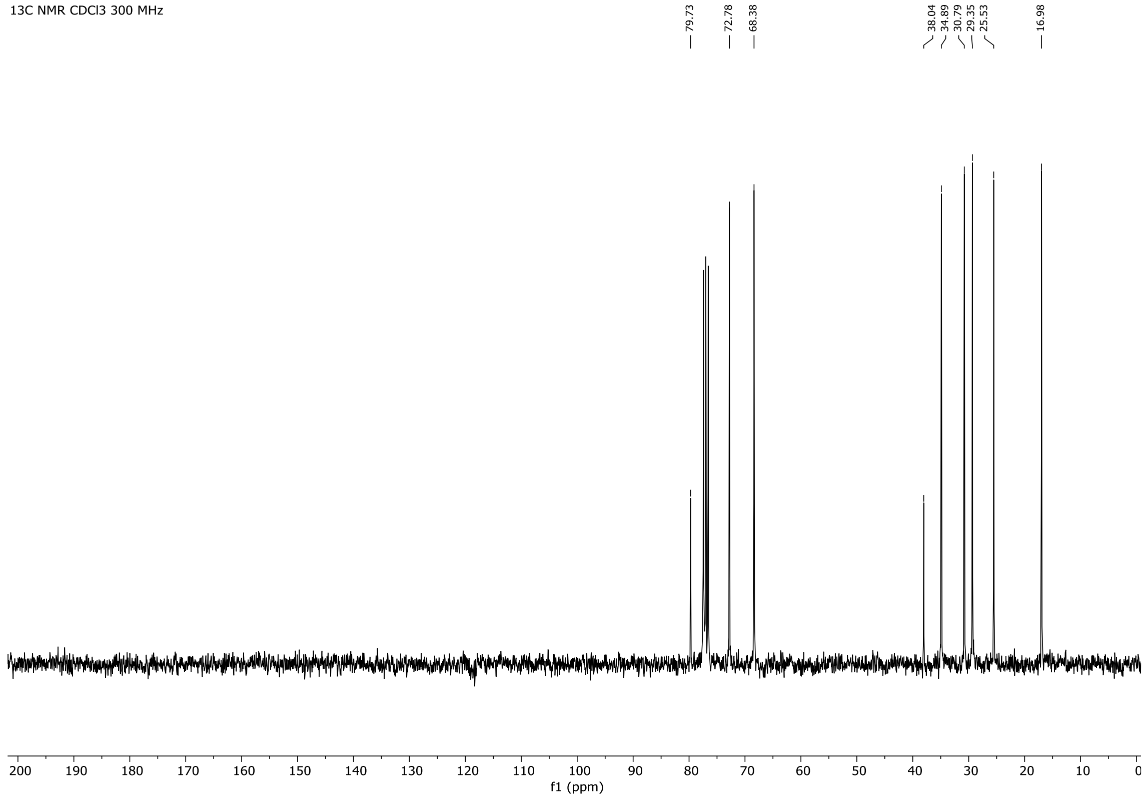

2-(tetrahydro-2H-pyran-4-yl)ethan-1-ol (4-OH)

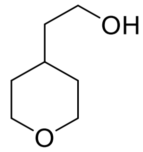

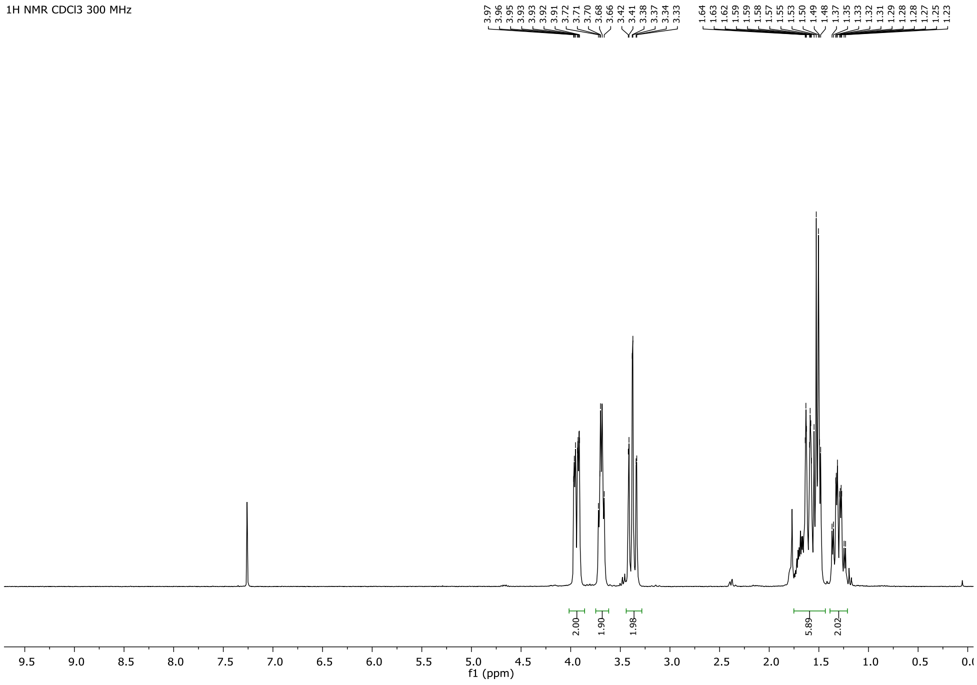

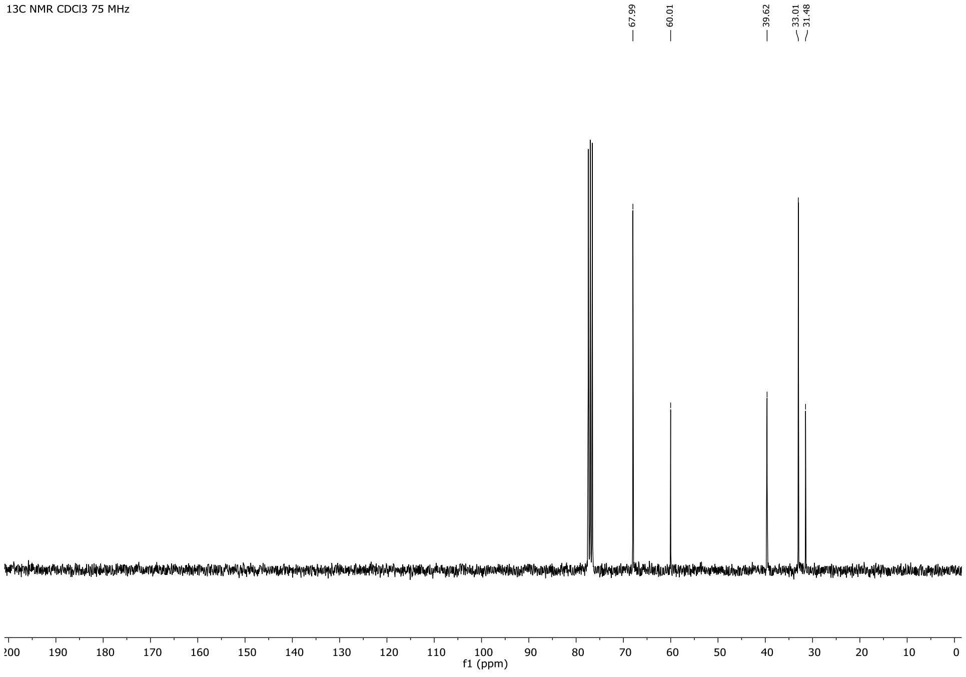

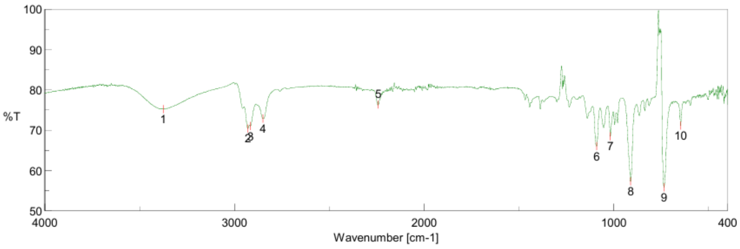

4-(2-bromoethyl)tetrahydro-2H-pyran (4-Br)

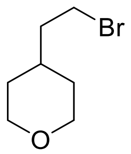

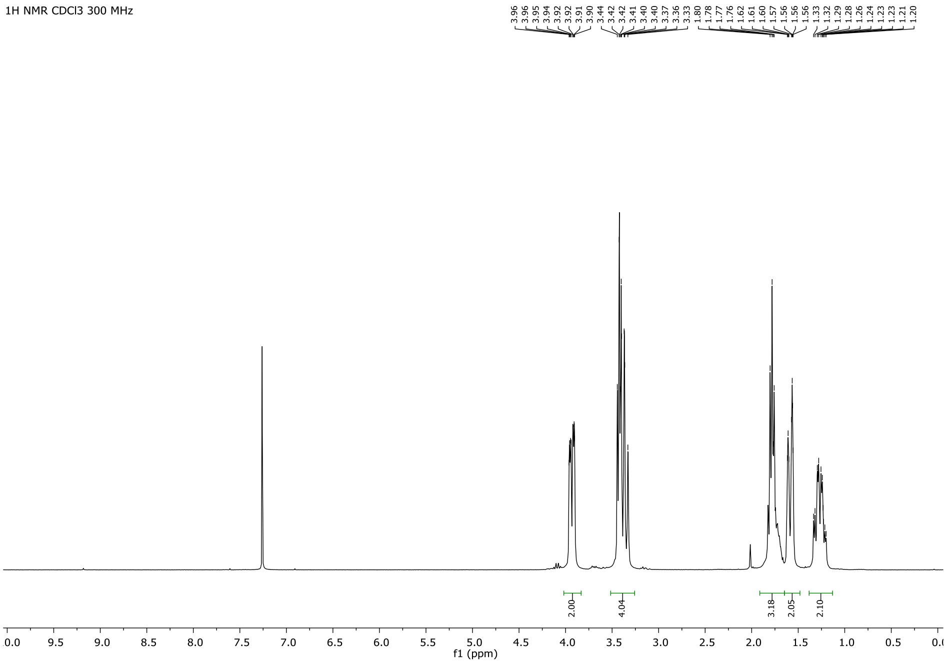

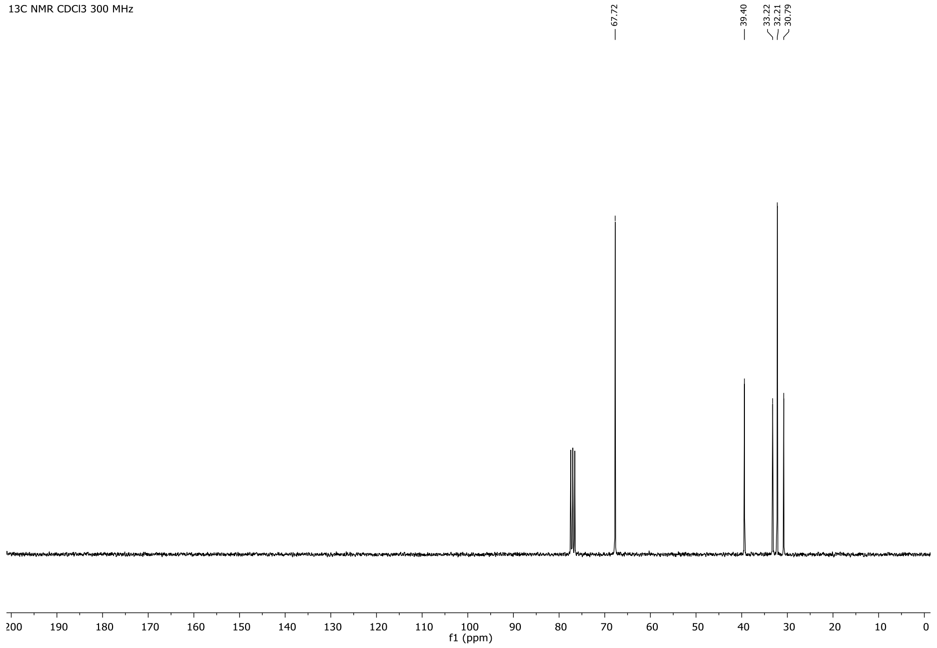

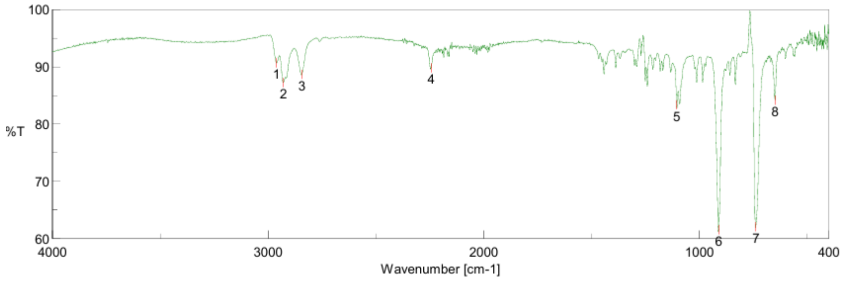

4-(but-3-yn-1-yl)tetrahydro-2H-pyran (4-CCH)

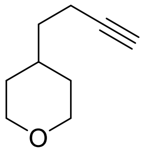

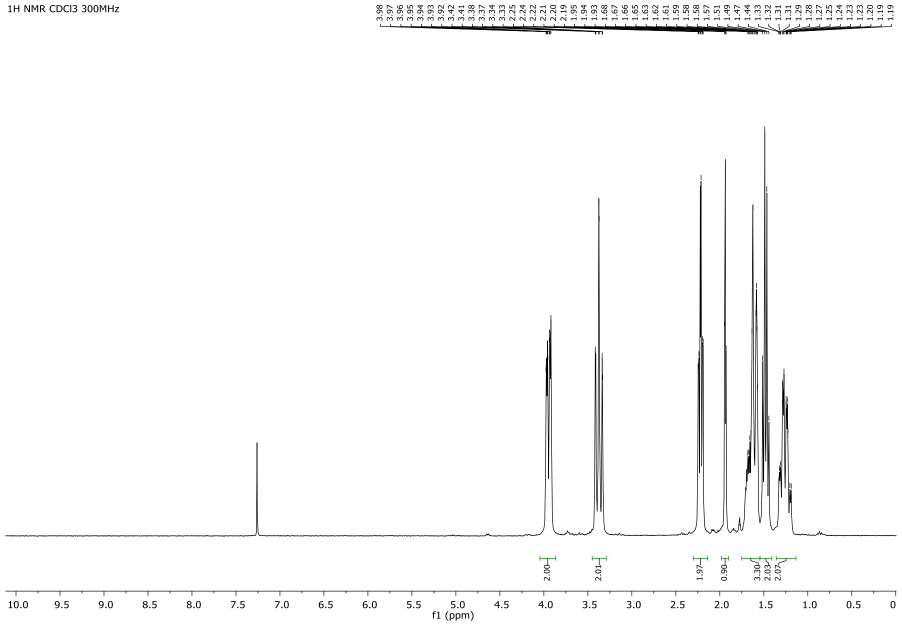

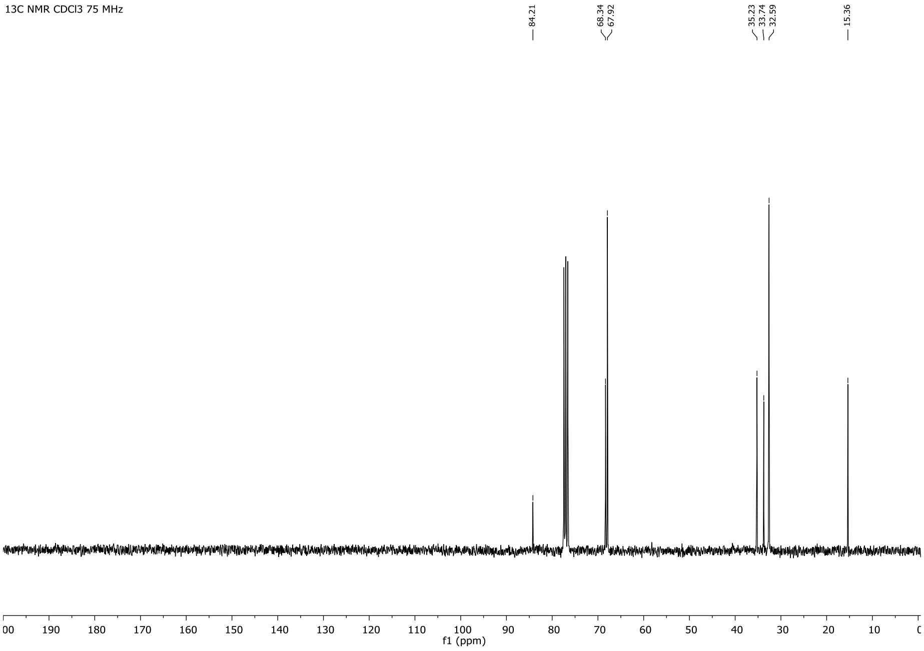

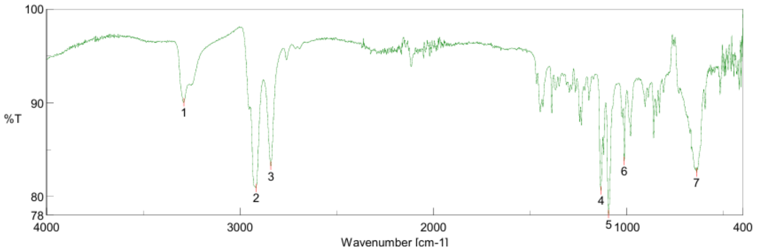

4-(4-bromobut-3-yn-1-yl)tetrahydro-2H-pyran (4)

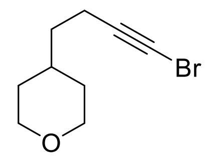

1H NMR CDCl3 300 MHz

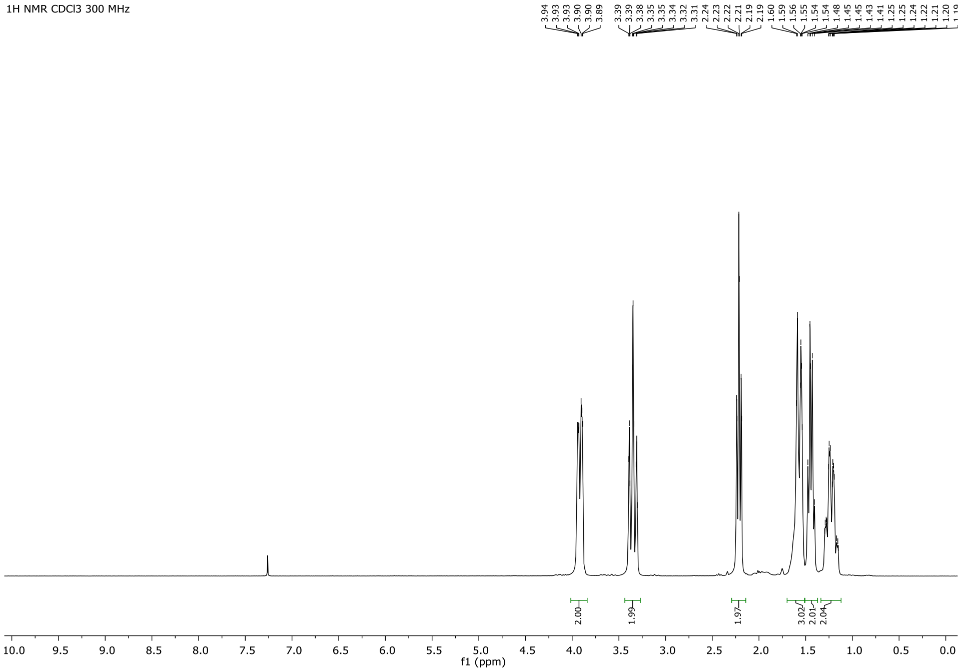

13C NMR CDCl3 75 MHz

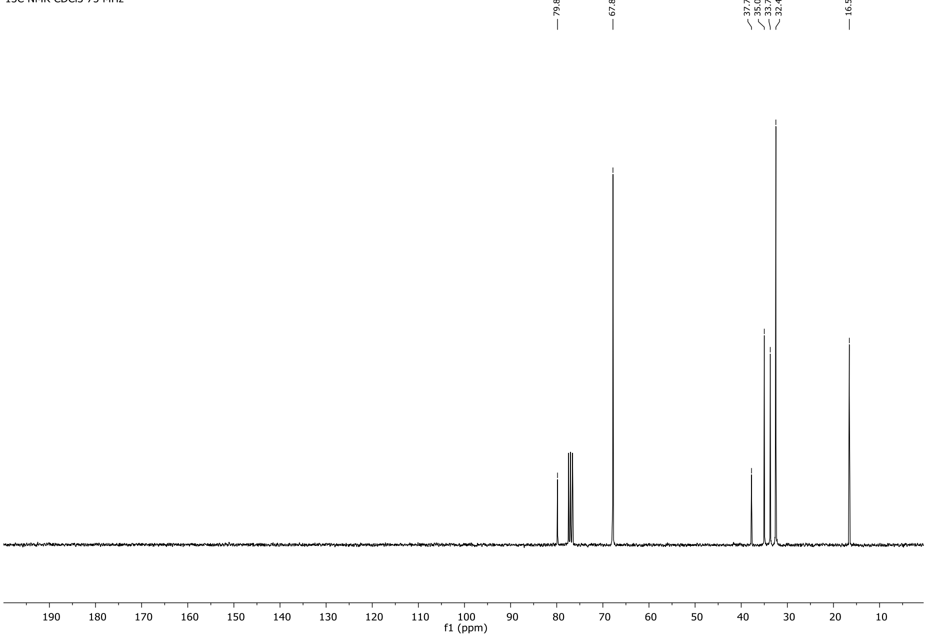

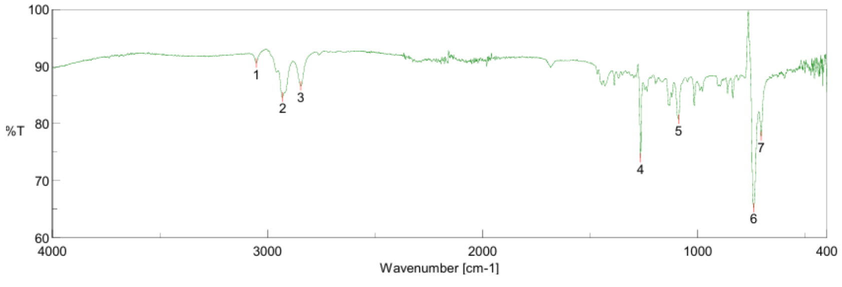

8-(2-bromoethyl)-1,4-dioxaspiro[4.5]decane (5-Br)

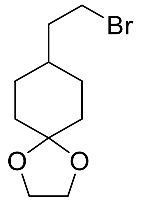

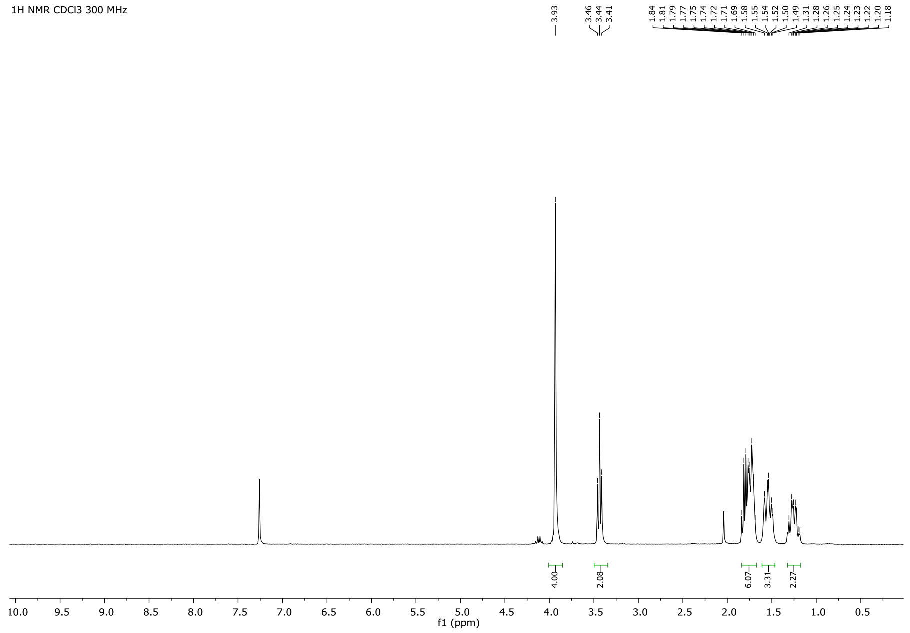

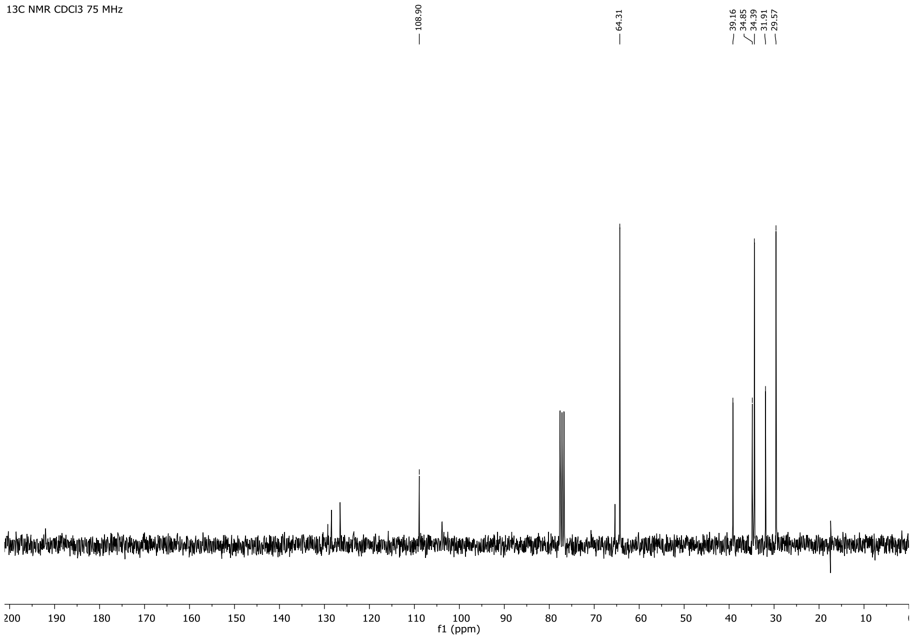

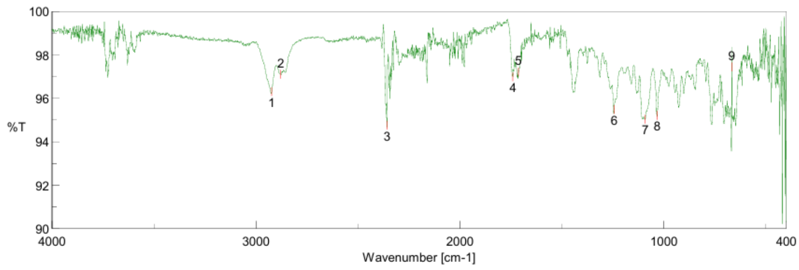

8-(but-3-yn-1-yl)-1,4-dioxaspiro[4.5]decane (5-CCH)

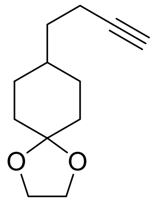

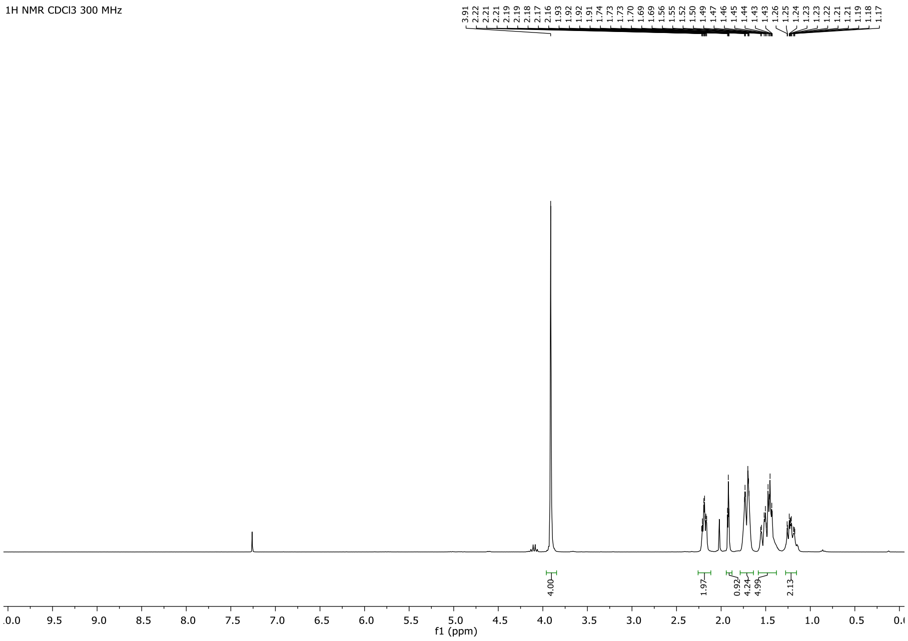

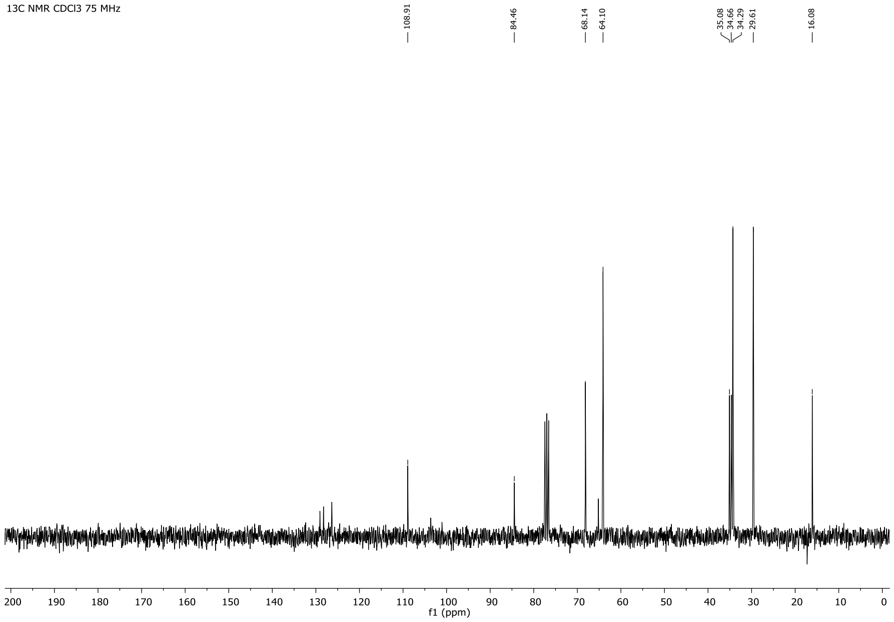

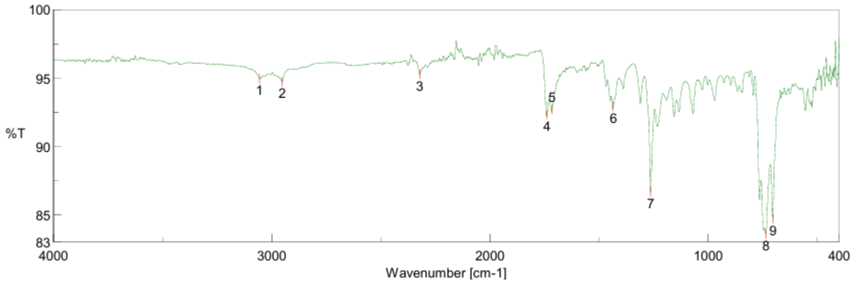

8-(4-bromobut-3-yn-1-yl)-1,4-dioxaspiro[4.5]decane (5)

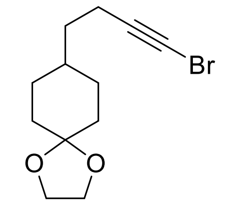

1H NMR CDCl3 300 MHz

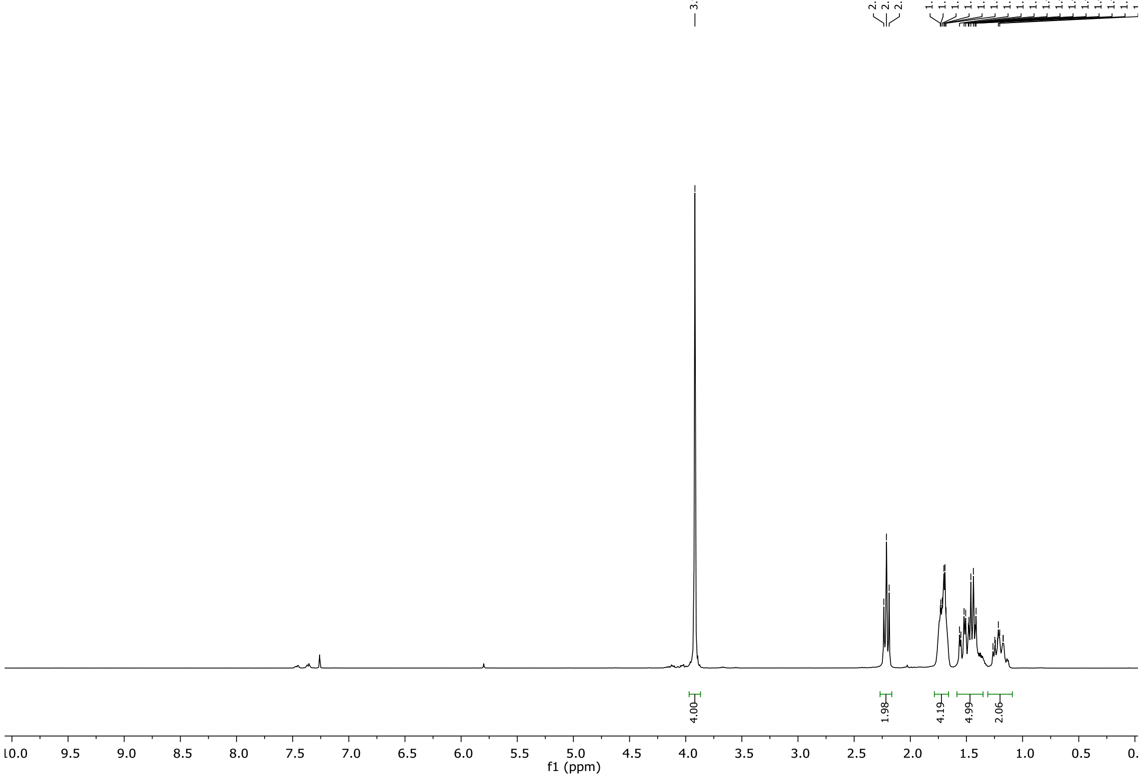

13C NMR CDCl3 75 MHz

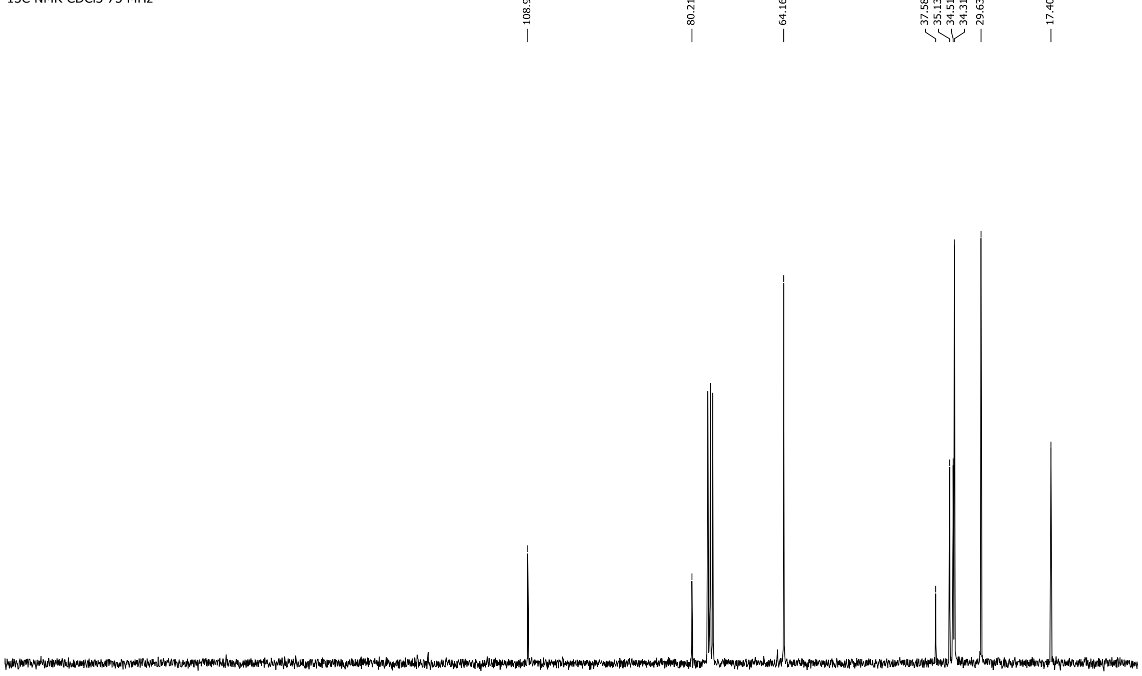

4-(4-bromobut-3-yn-1-yl)cyclohexan-1-one (6)

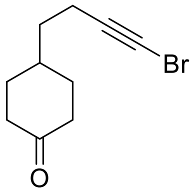

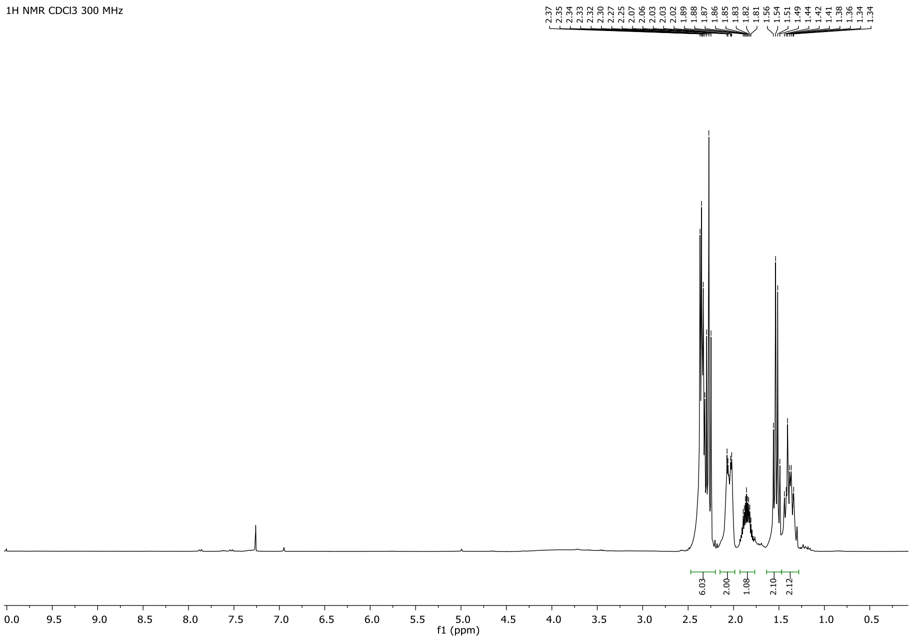

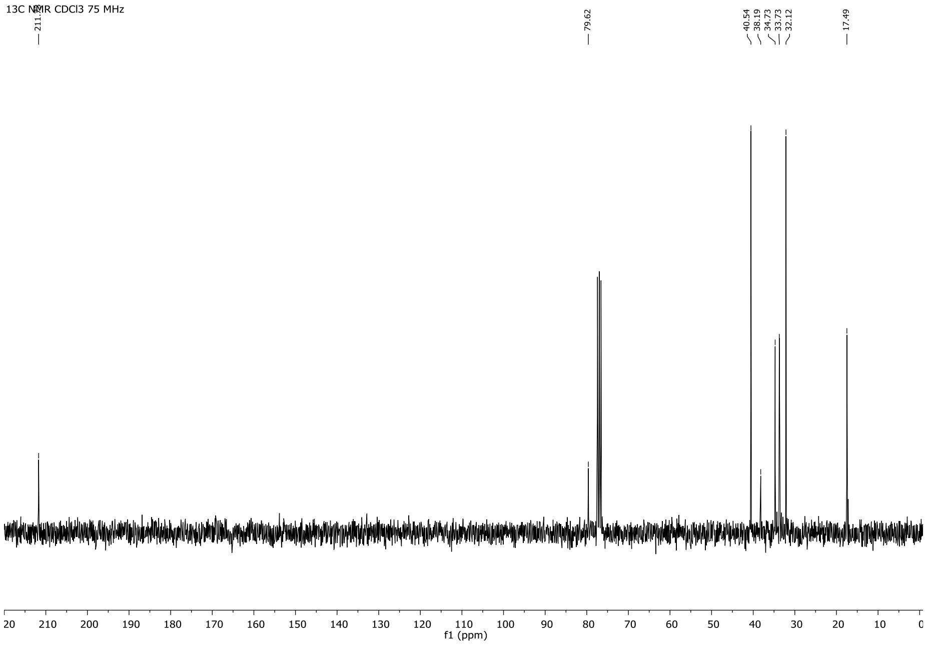

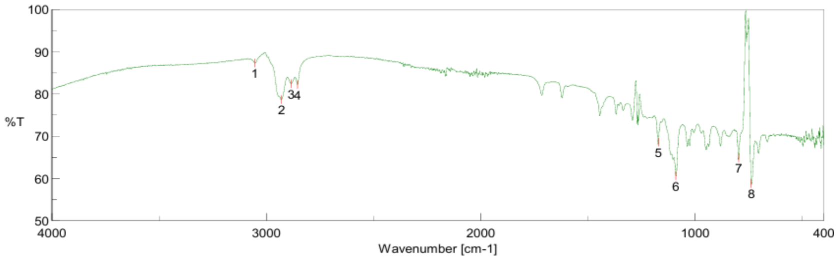

tert-butyl 4-(2-hydroxyethyl)piperidine-1-carboxylate (7-9/20-OH)

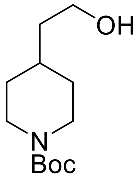

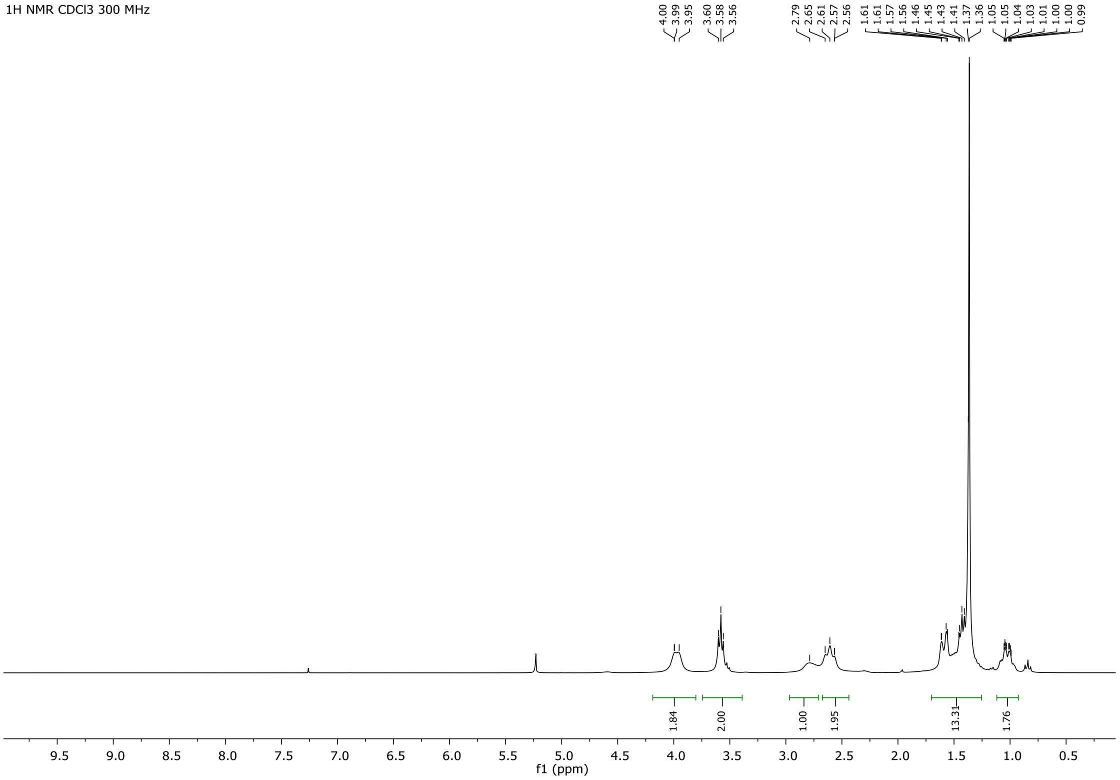

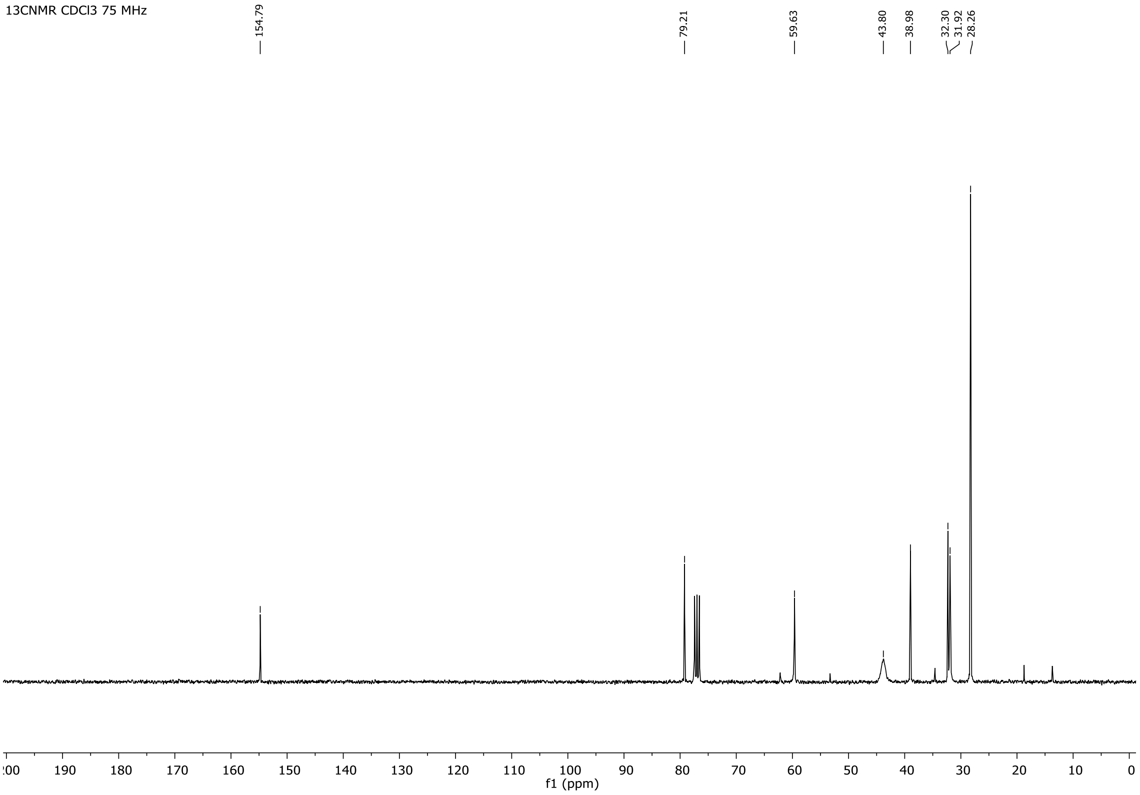

tert-butyl 4-(2-bromoethyl)piperidine-1-carboxylate (7-9/20-Br)

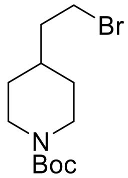

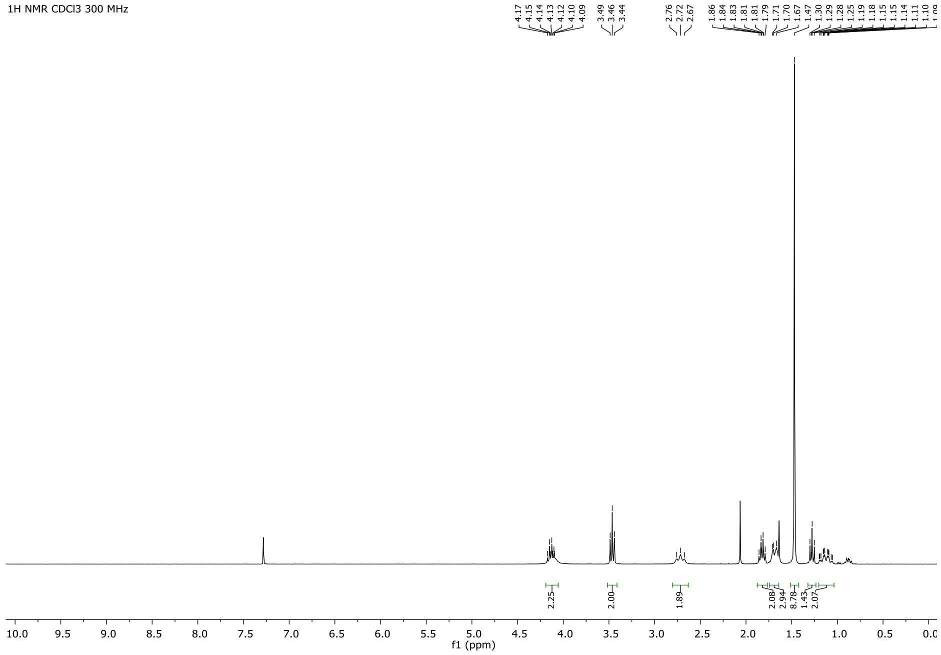

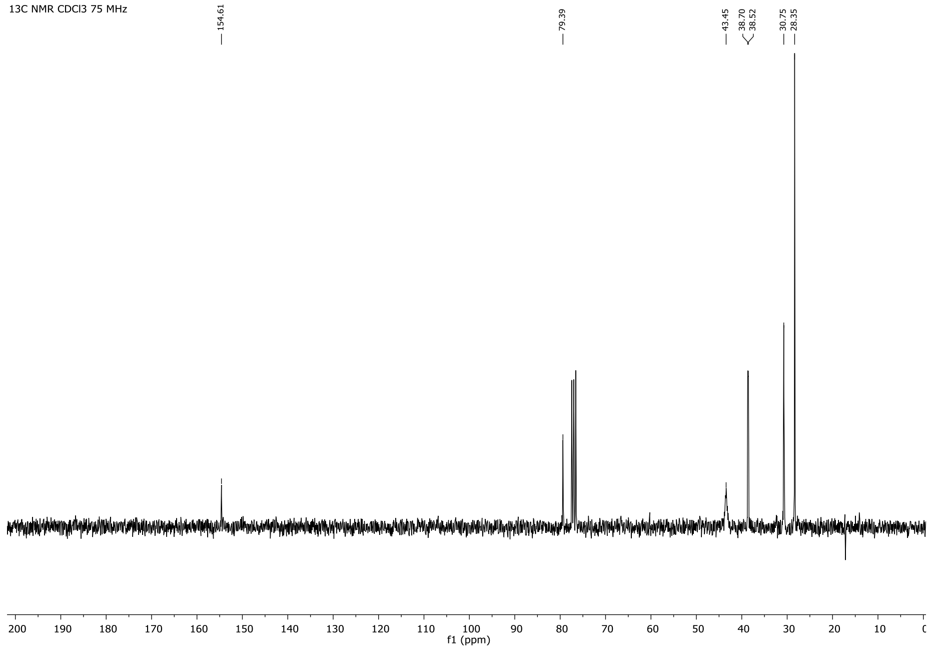

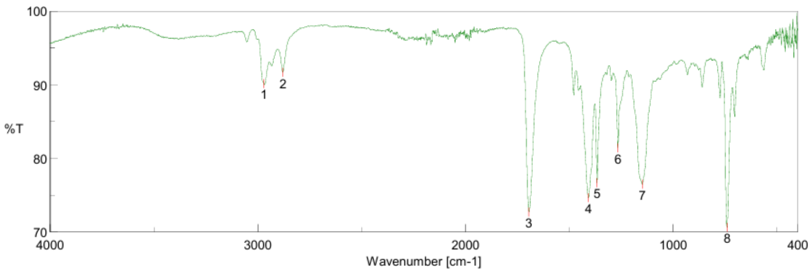

tert-butyl 4-(but-3-yn-1-yl)piperidine-1-carboxylate (7-9/20-CCH)

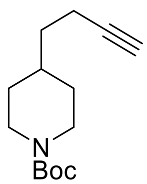

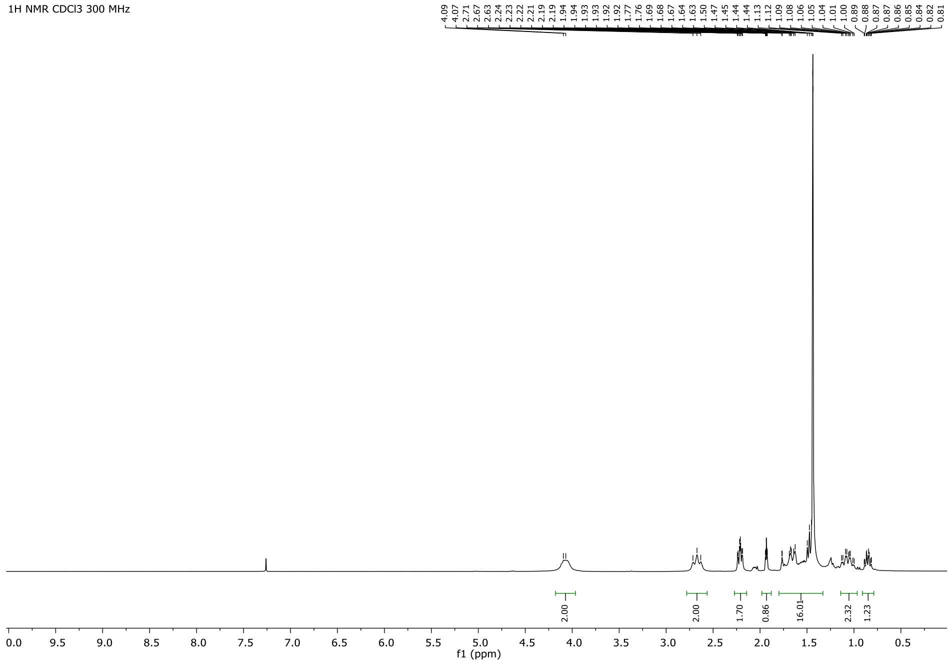

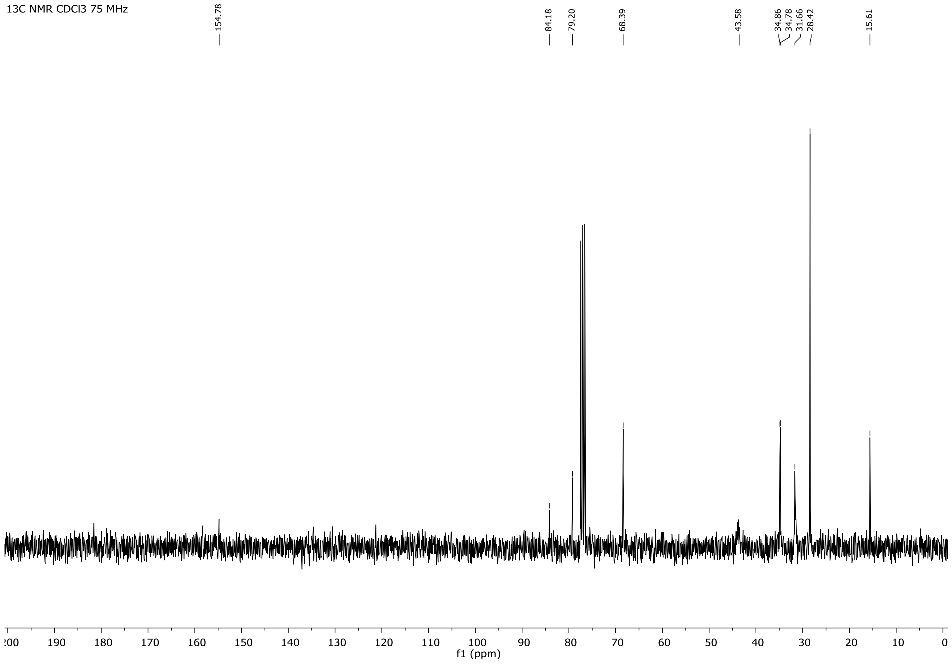

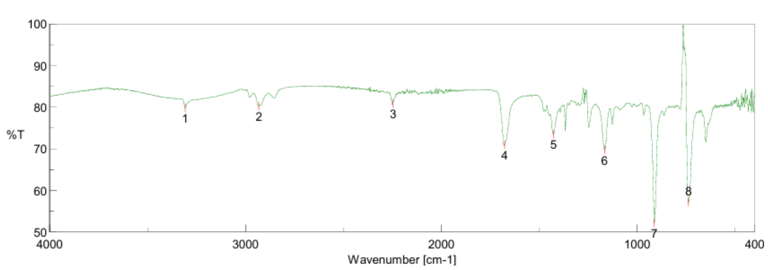

1-(4-(4-bromobut-3-yn-1-yl)piperidin-1-yl)-2,2,2-trifluoroethan-1-one (7, 8, 9-NH)

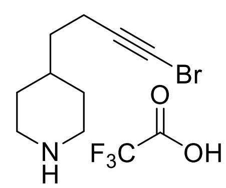

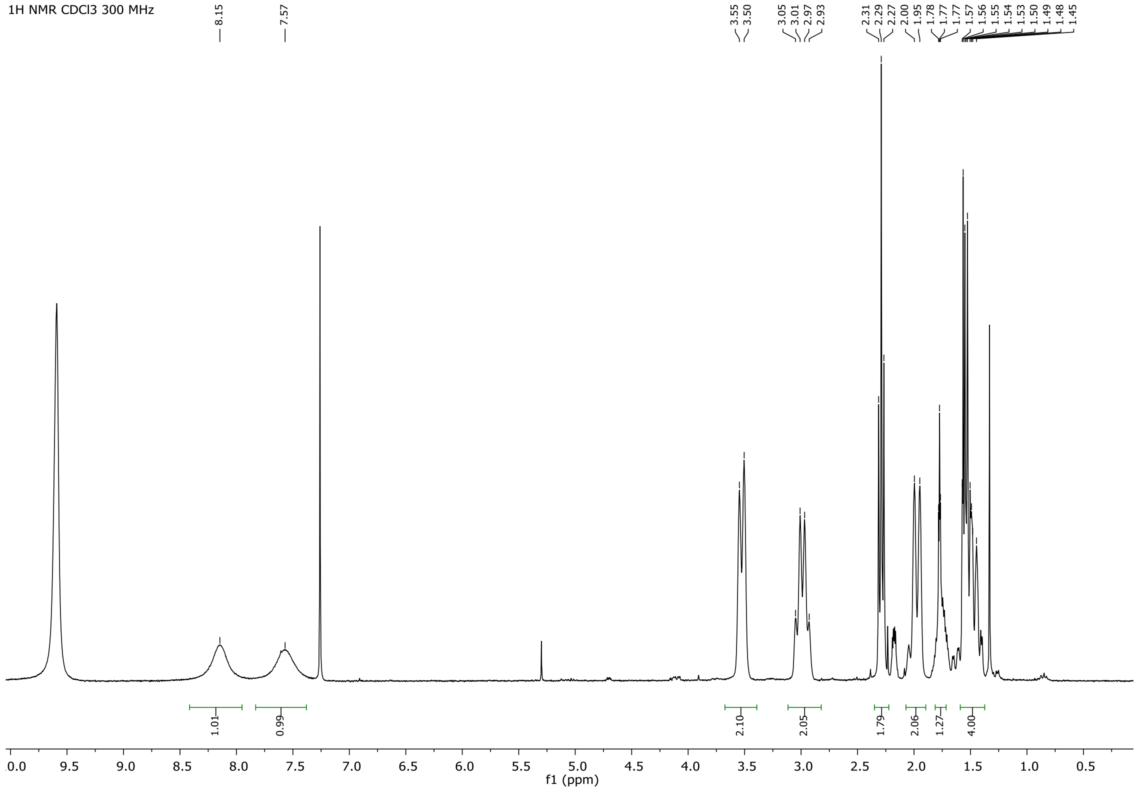

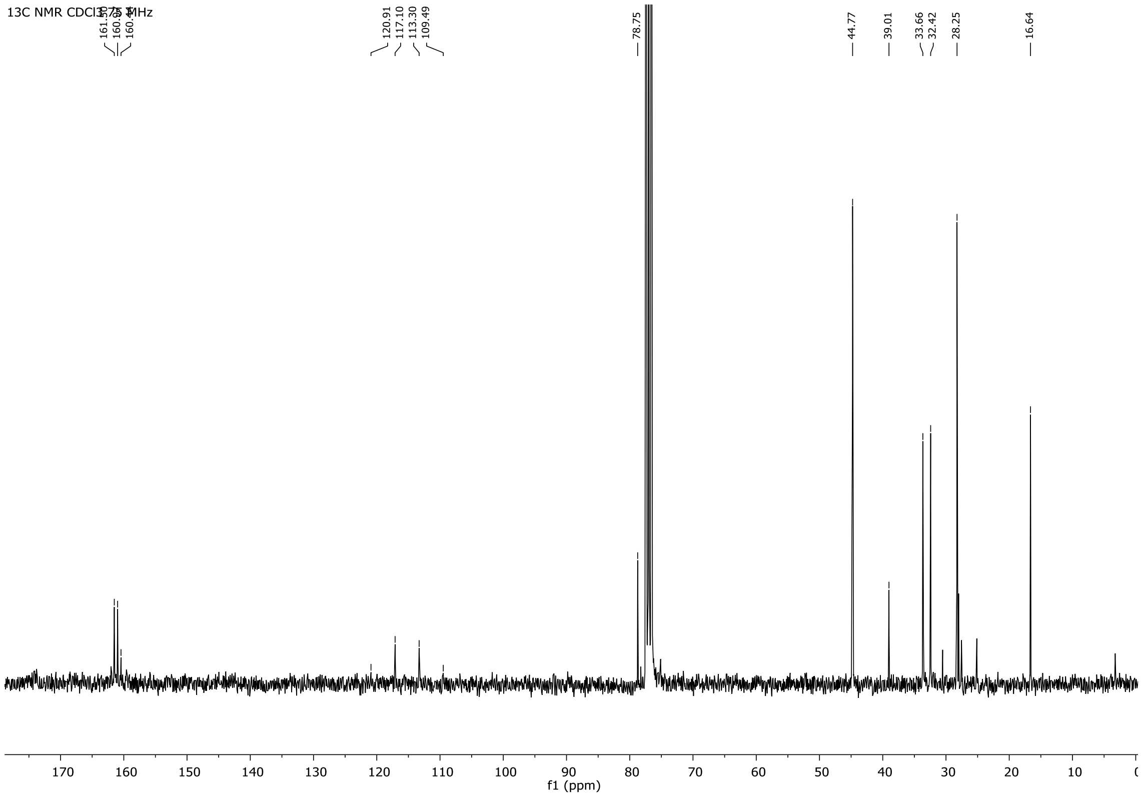

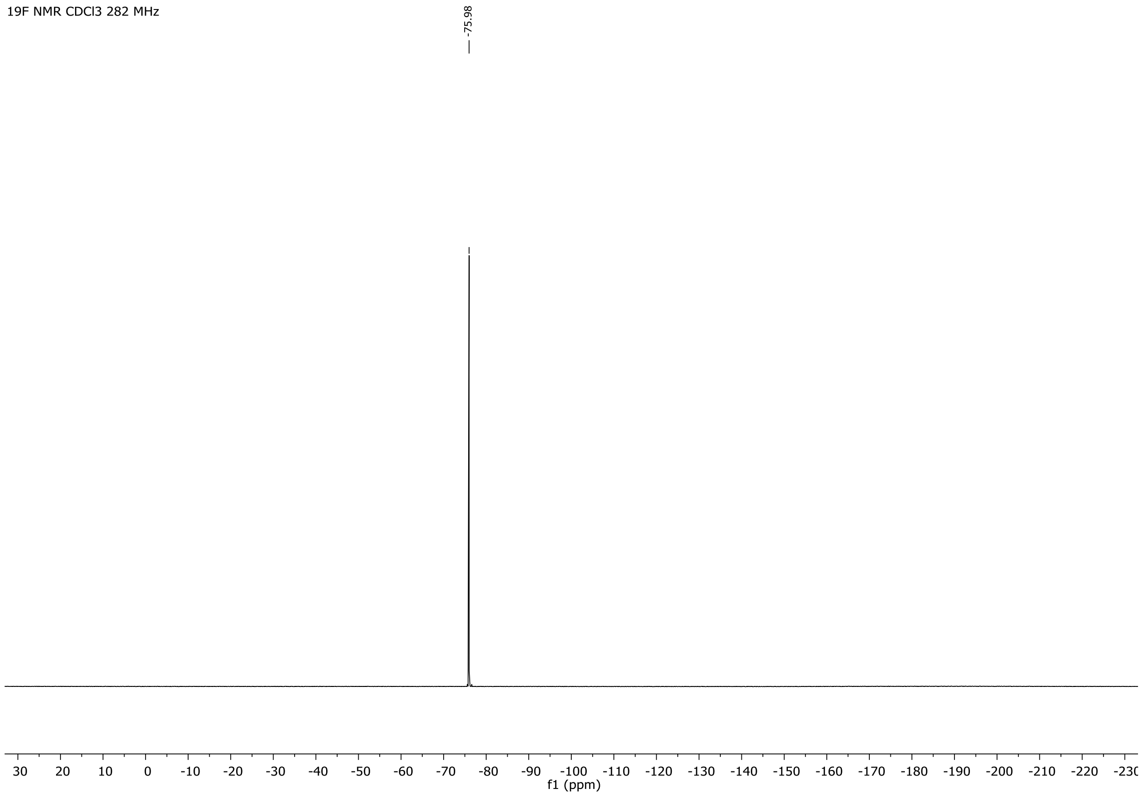
19F NMR CDCl3 282 MHz
-75.98
30 20 10 0 -10 -20 -30 -40 -50 -60 -70 -80 -90 -100 -110 -120 -130 -140 -150 -160 -170 -180 -190 -200 -210 -220 -230
f1 (ppm)

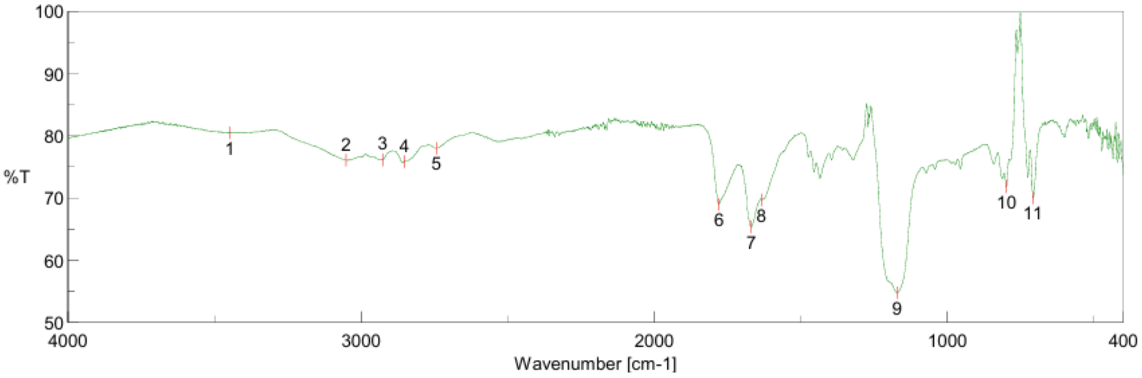
100
90
80
70
60
50
%T
1 2 3 4 5 6 7 8 9 10 11
4000 3000 2000 1000 400
Wavenumber [cm-1]

4-(4-bromobut-3-yn-1-yl)piperidine 2,2,2-trifluoroacetate (7)

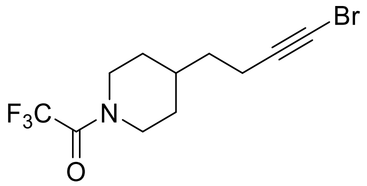

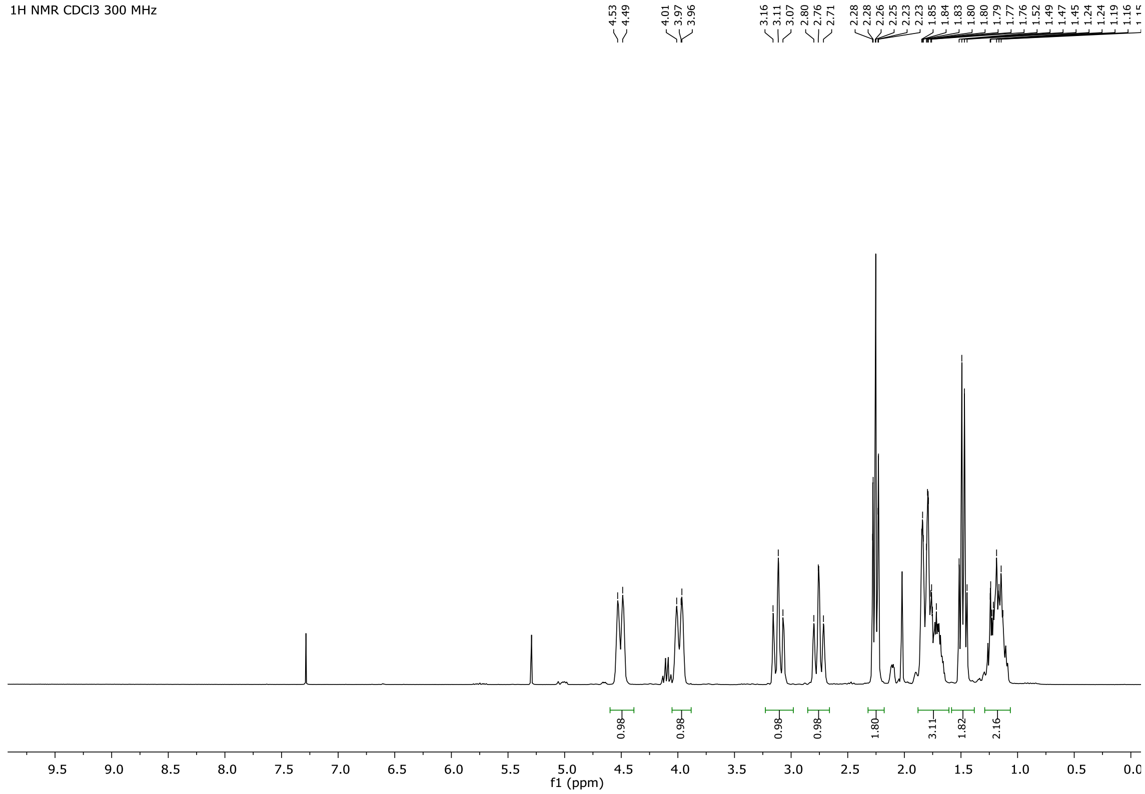

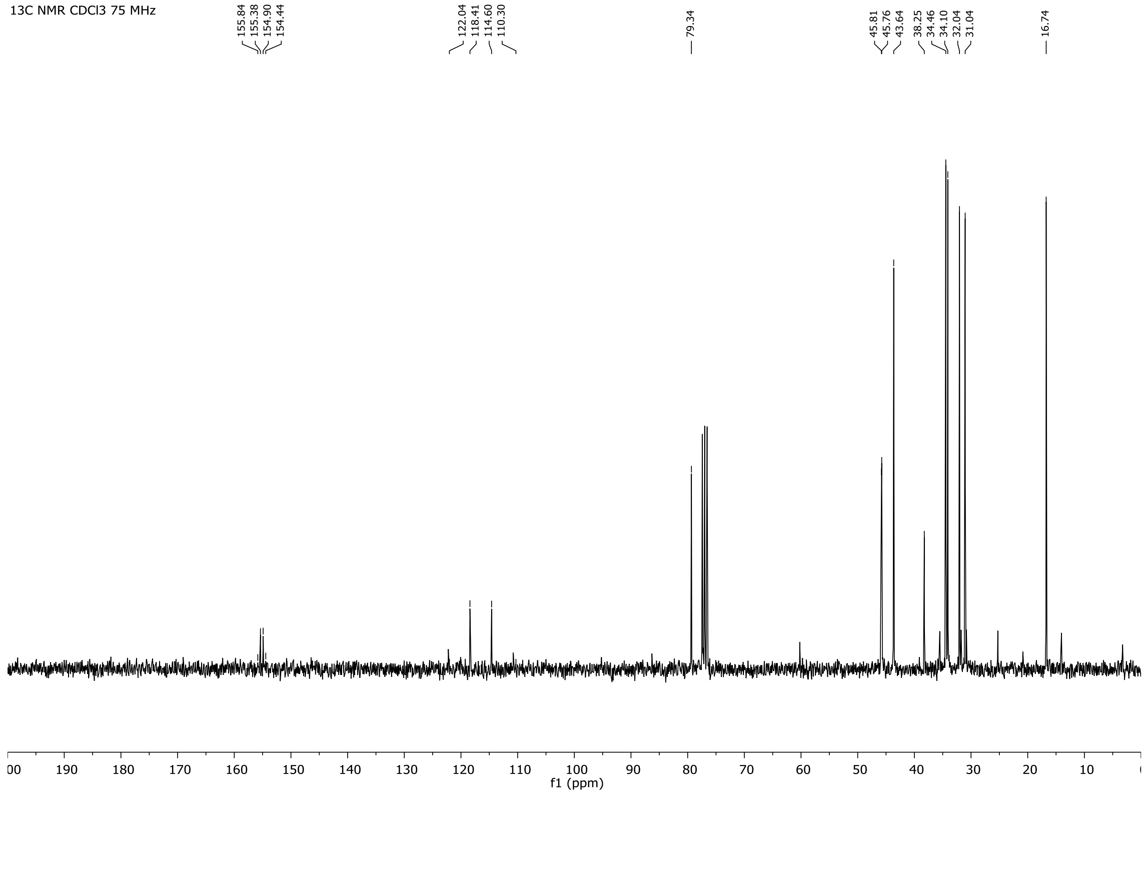

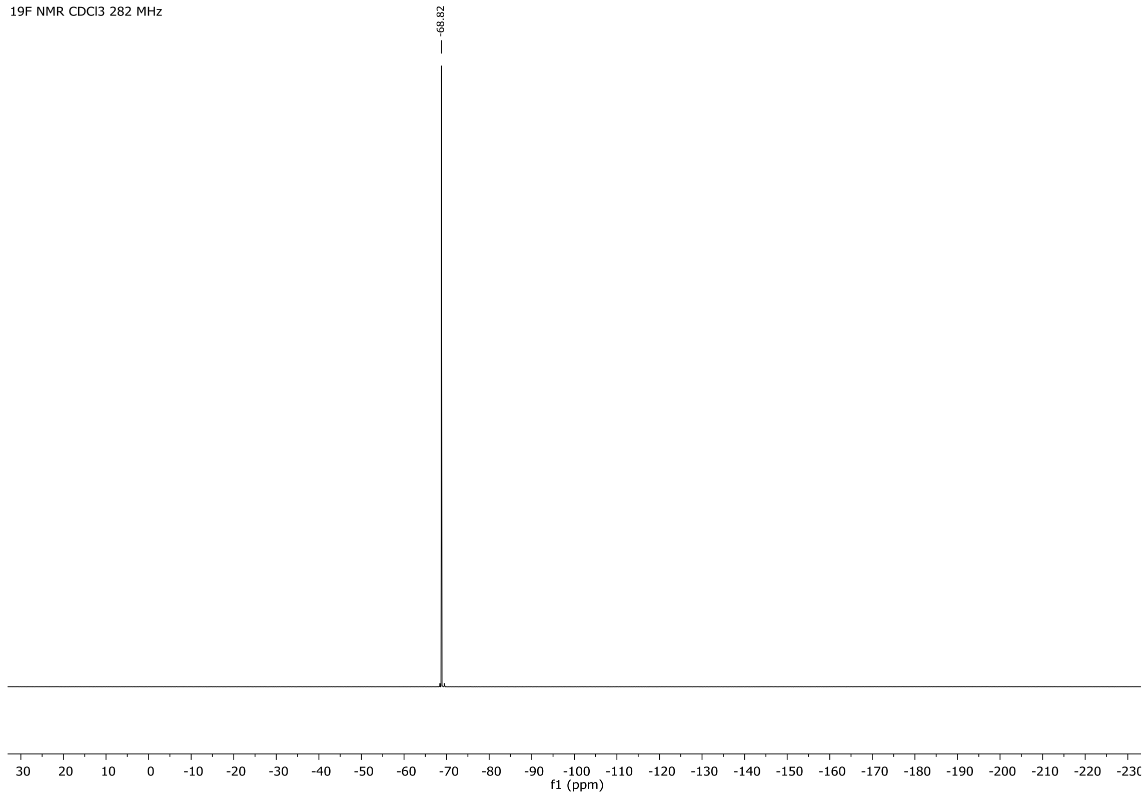
19F NMR CDCl3 282 MHz
-68.82
30
20
10
0
-10
-20
-30
-40
-50
-60
-70
-80
-90
-100
-110
-120
-130
-140
-150
-160
-170
-180
-190
-200
-210
-220
f1 (ppm)

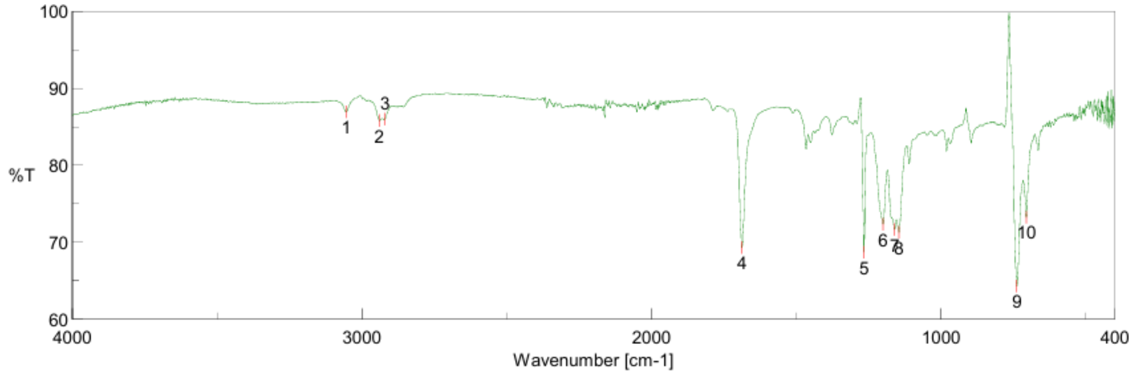
100
90
80
70
60
%T
1
2
3
4
5
6
7
8
9
10
4000
3000
2000
1000
400
Wavenumber [cm-1]

benzyl 4-(4-bromobut-3-yn-1-yl)piperidine-1-carboxylate (8)

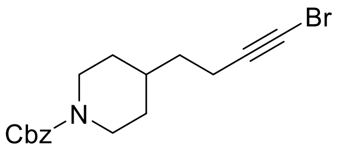

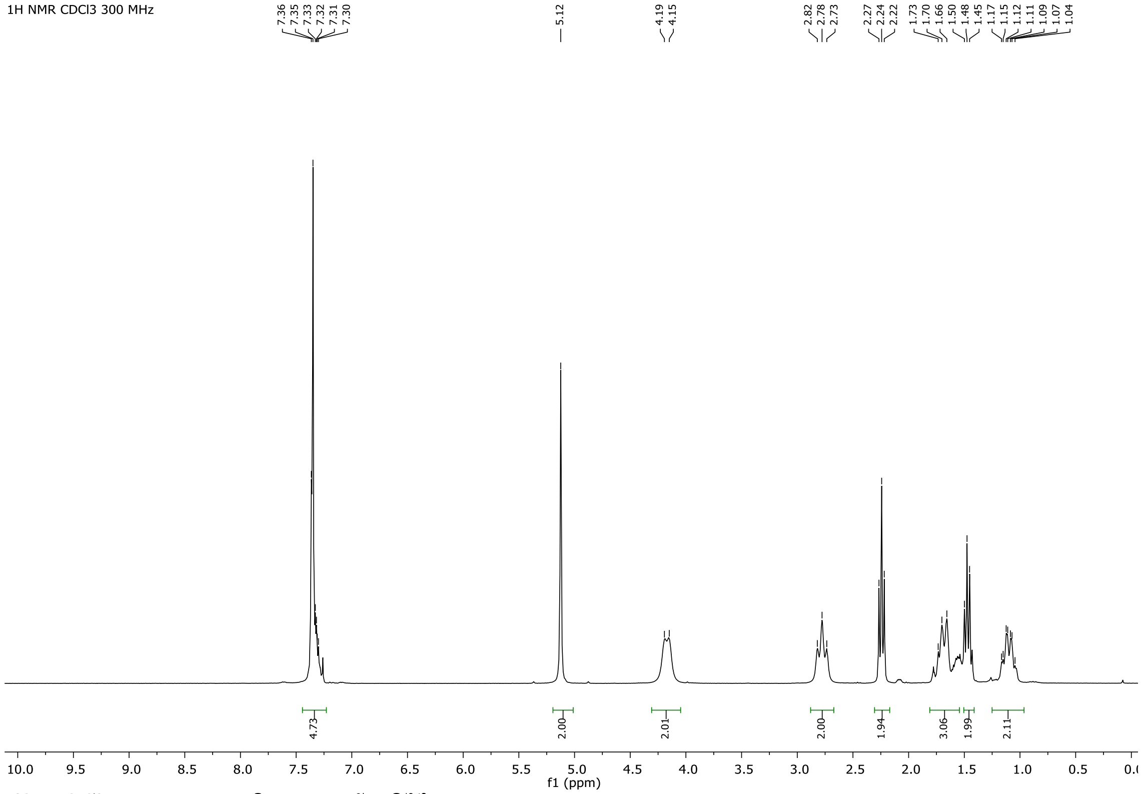

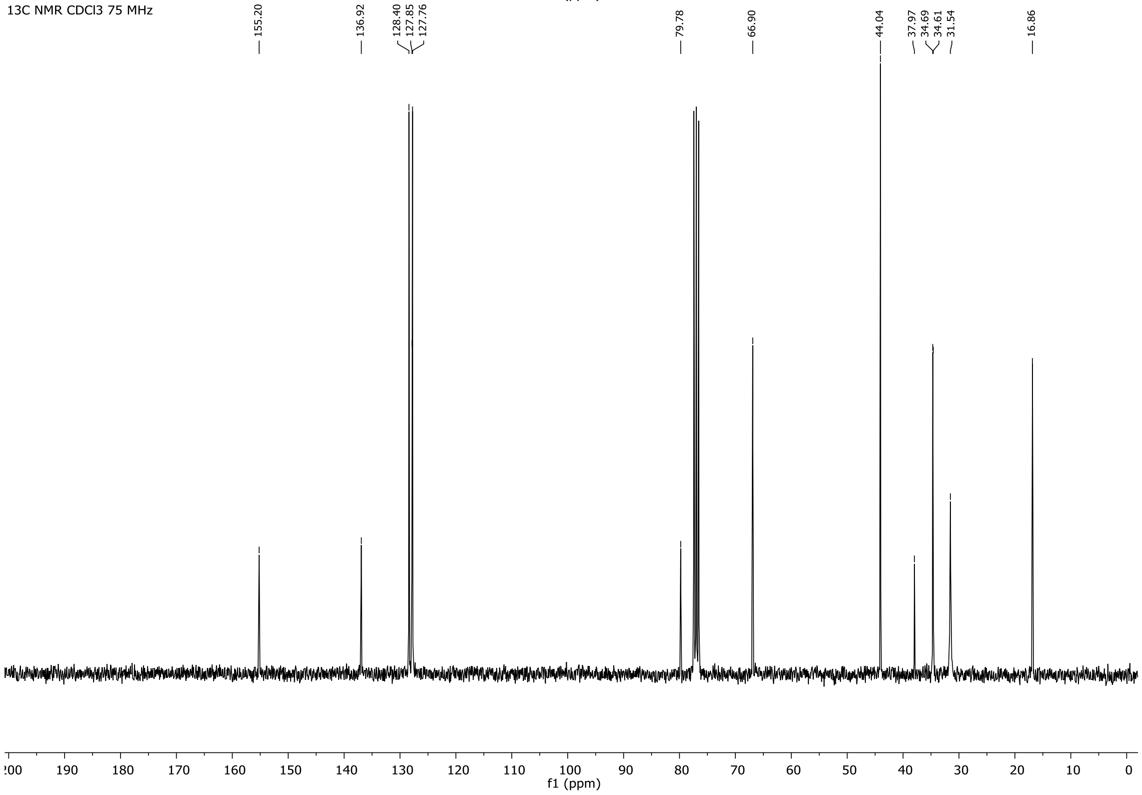

4-(4-bromobut-3-yn-1-yl)-1-tosylpiperidine (9)

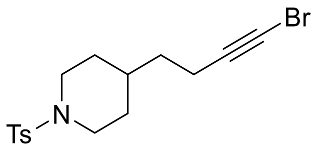

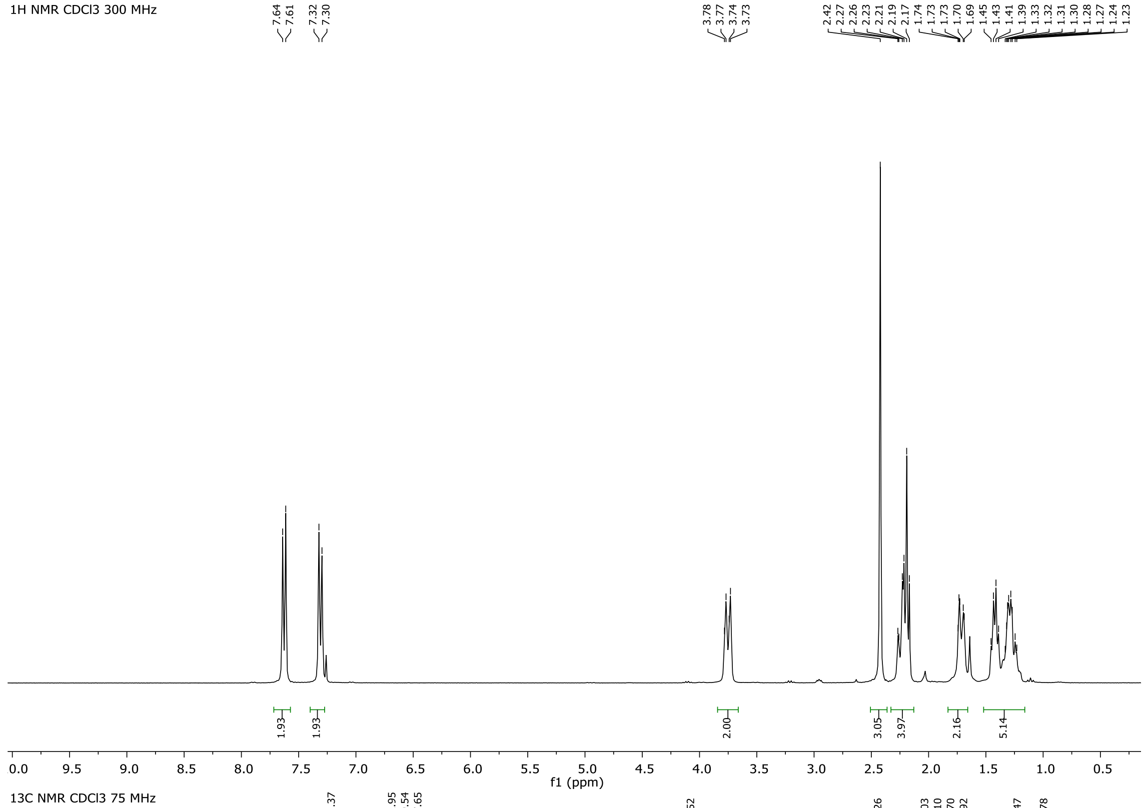

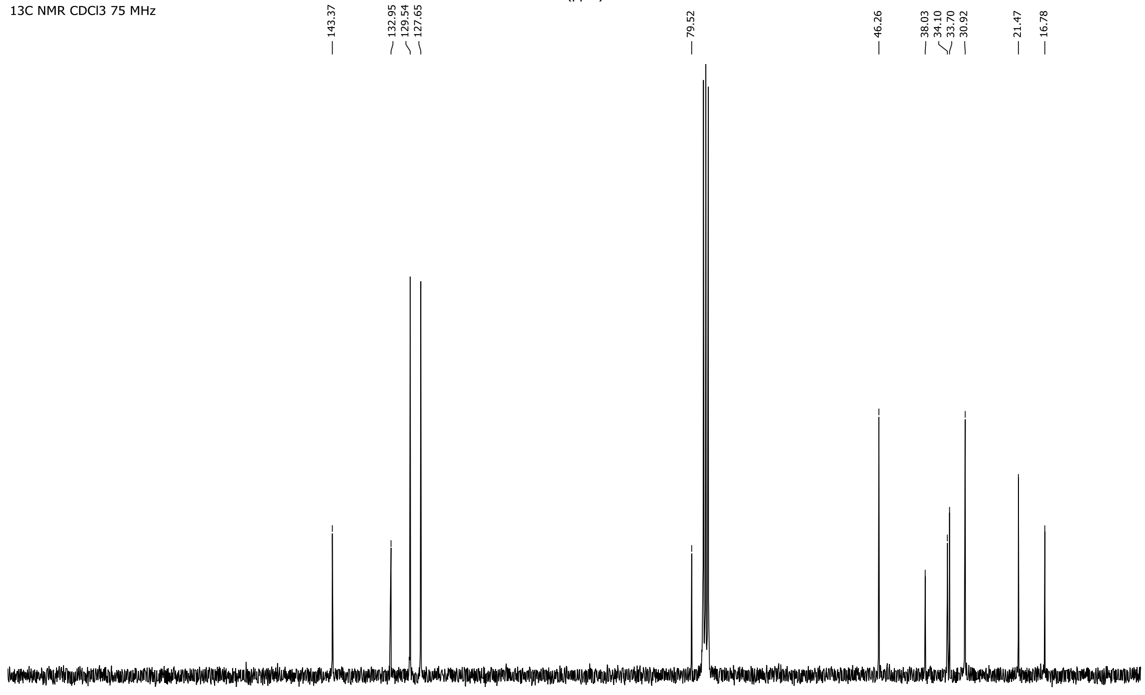

4-(4-bromobut-3-yn-1-yl)-1,1-difluorocyclohexane (10)

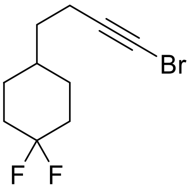

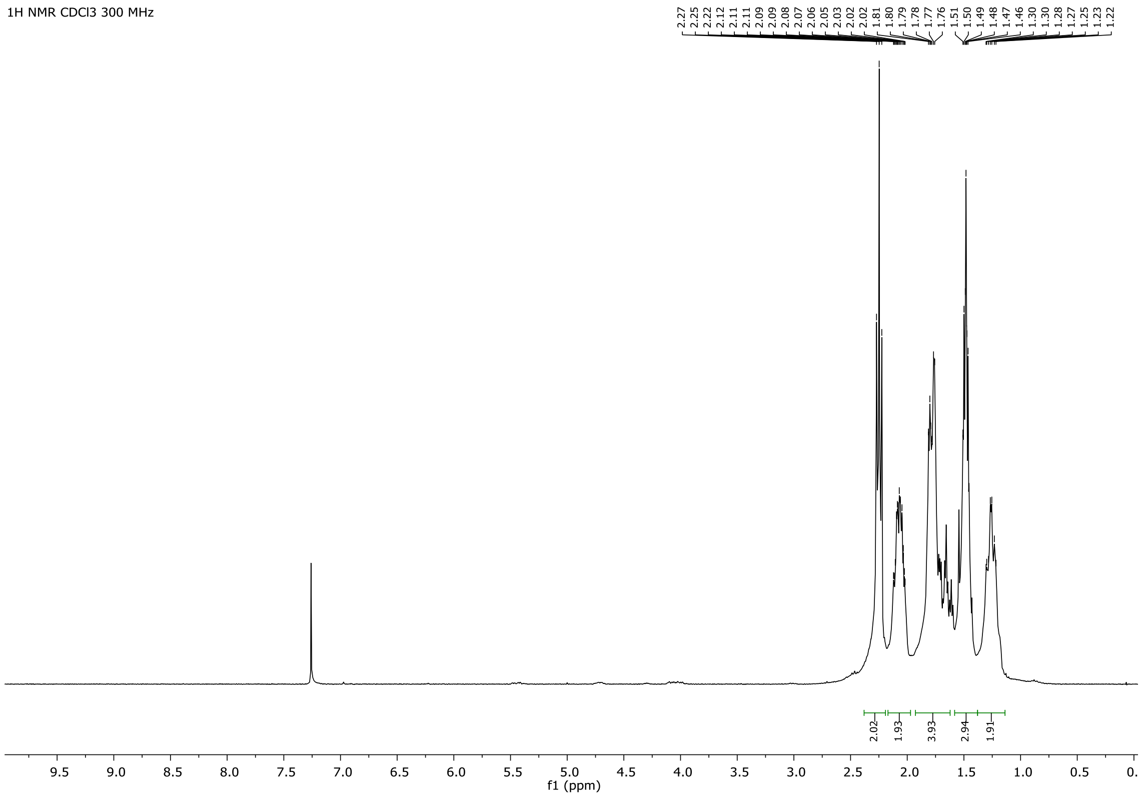

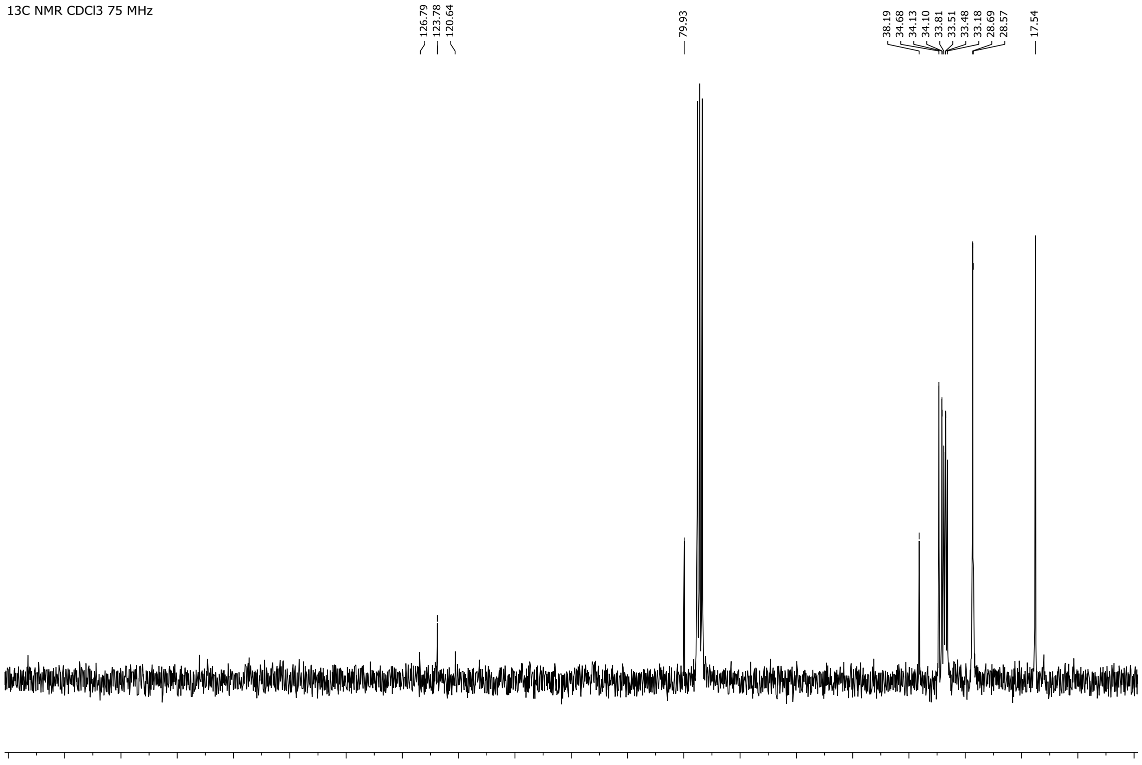

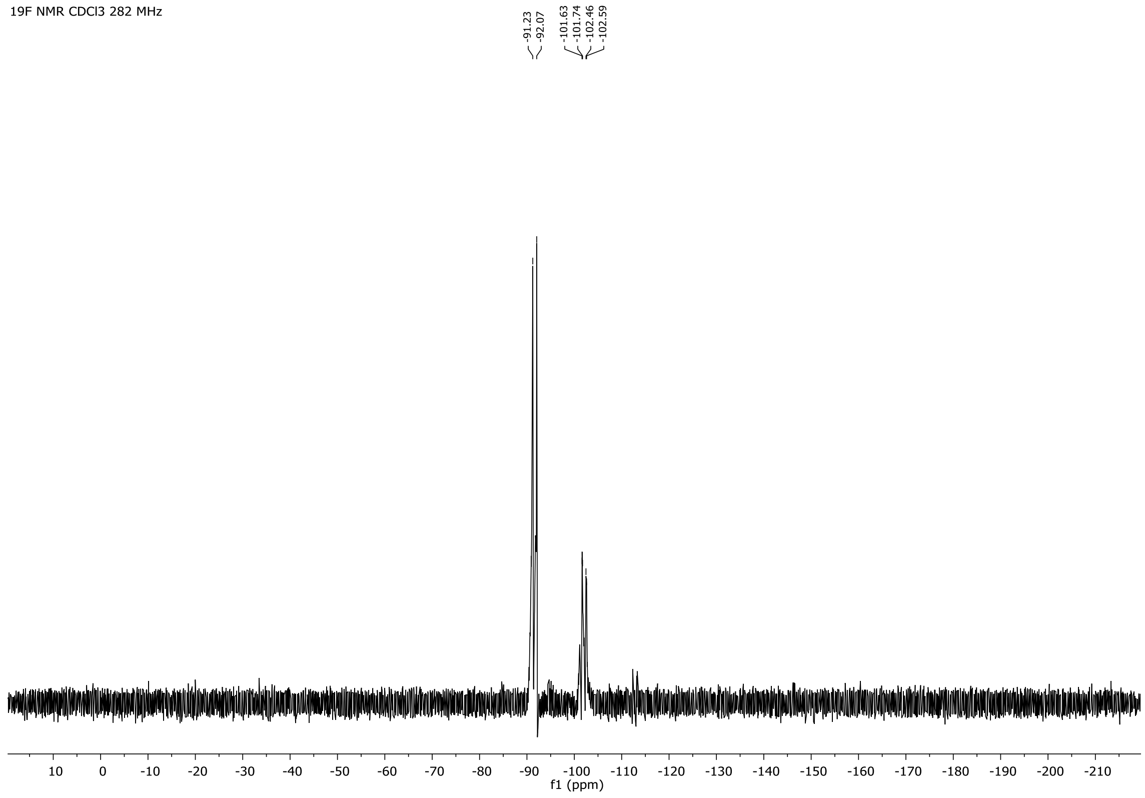

19F NMR CDCl3 282 MHz
-91.23
-92.07
-101.63
-101.74
-102.46
-102.59
10 0 -10 -20 -30 -40 -50 -60 -70 -80 -90 -100 -110 -120 -130 -140 -150 -160 -170 -180 -190 -200 -210
f1 (ppm)

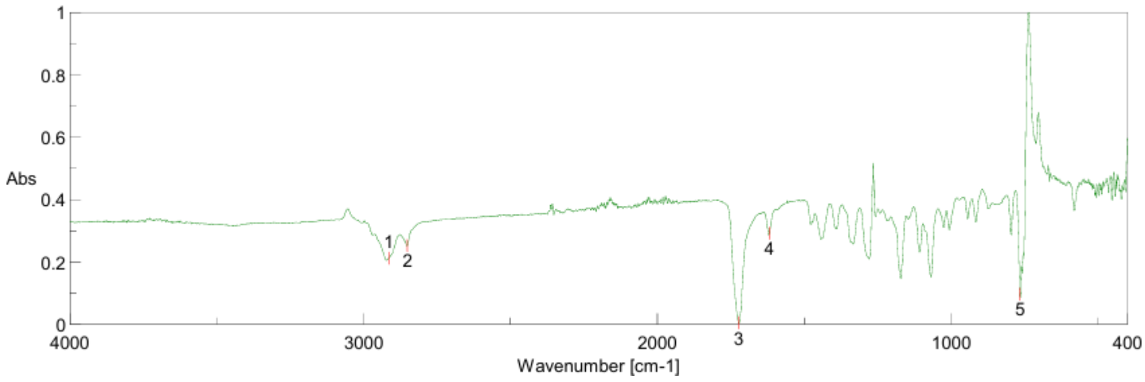

Abs
1
0.8
0.6
0.4
0.2
0
1
2
3
4
5
4000 3000 2000 1000 400
Wavenumber [cm-1]

4-(2-bromoethyl)tetrahydro-2H-thiopyran 1,1-dioxide (11-Br)

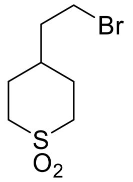

1H NMR CDCl3 300 MHz

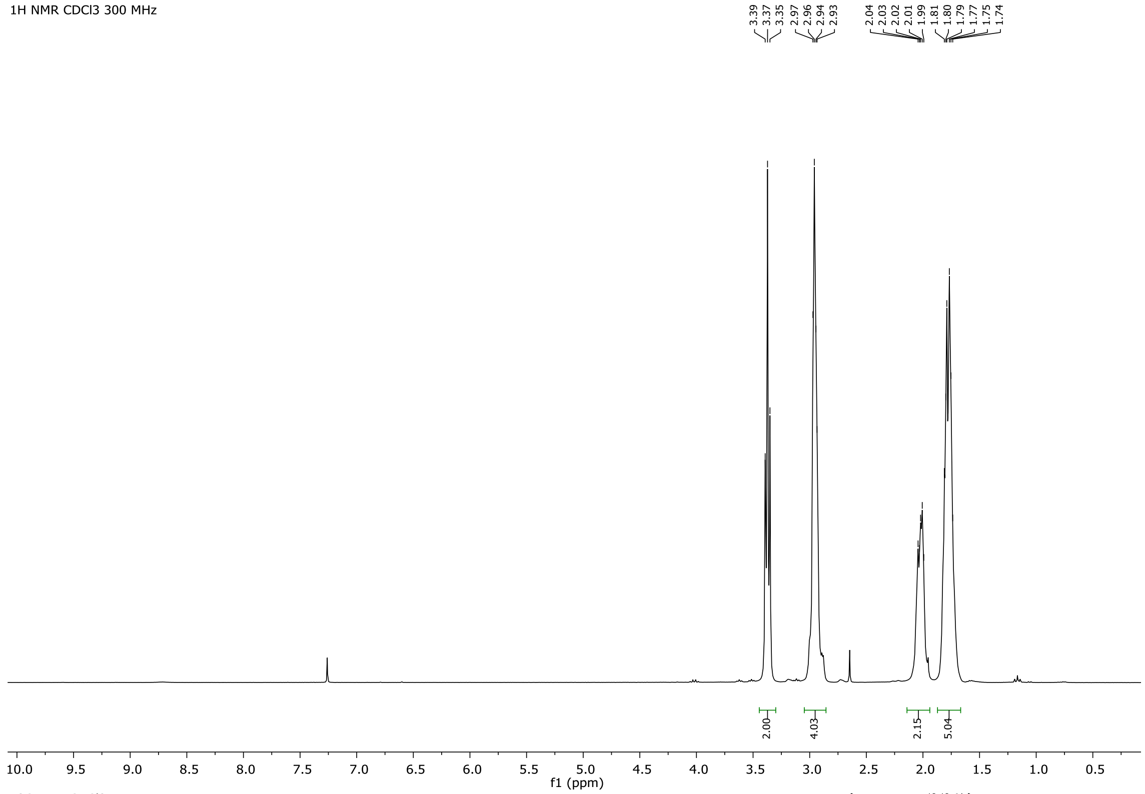

13C NMR CDCl3 75 MHz

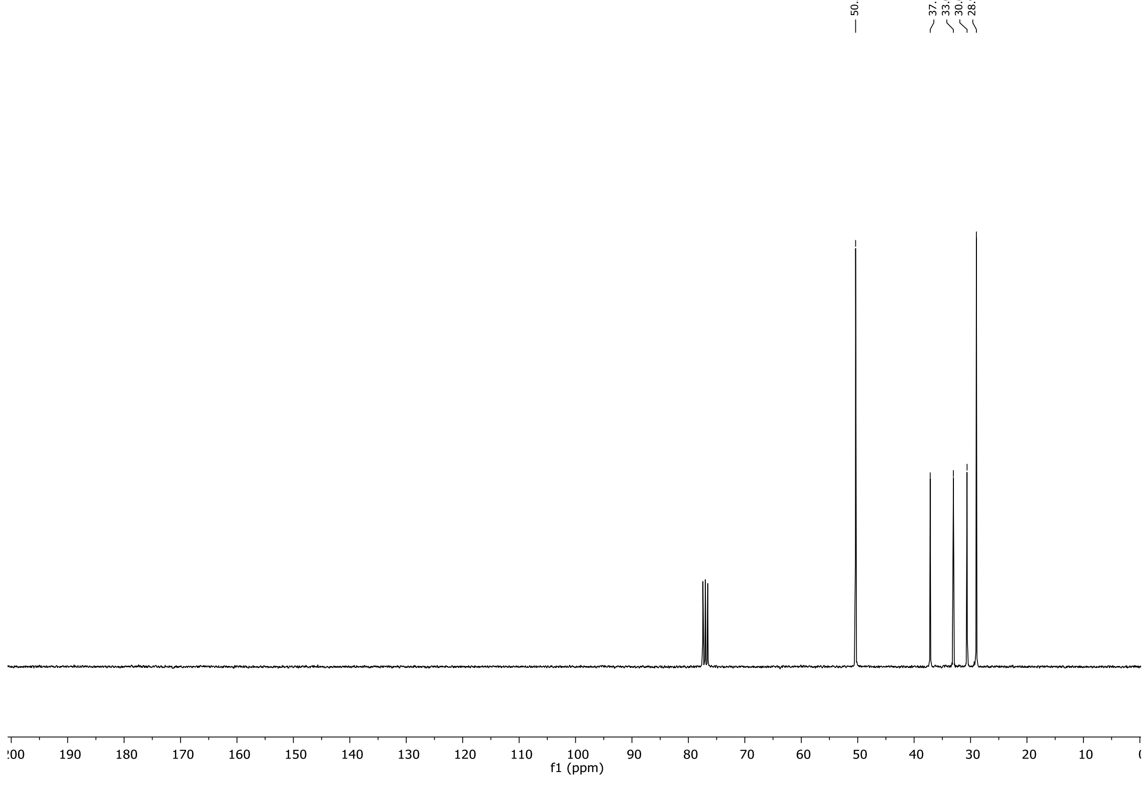

4-(but-3-yn-1-yl)tetrahydro-2H-thiopyran 1,1-dioxide (11-CCH)

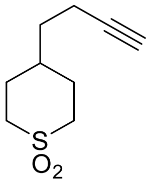

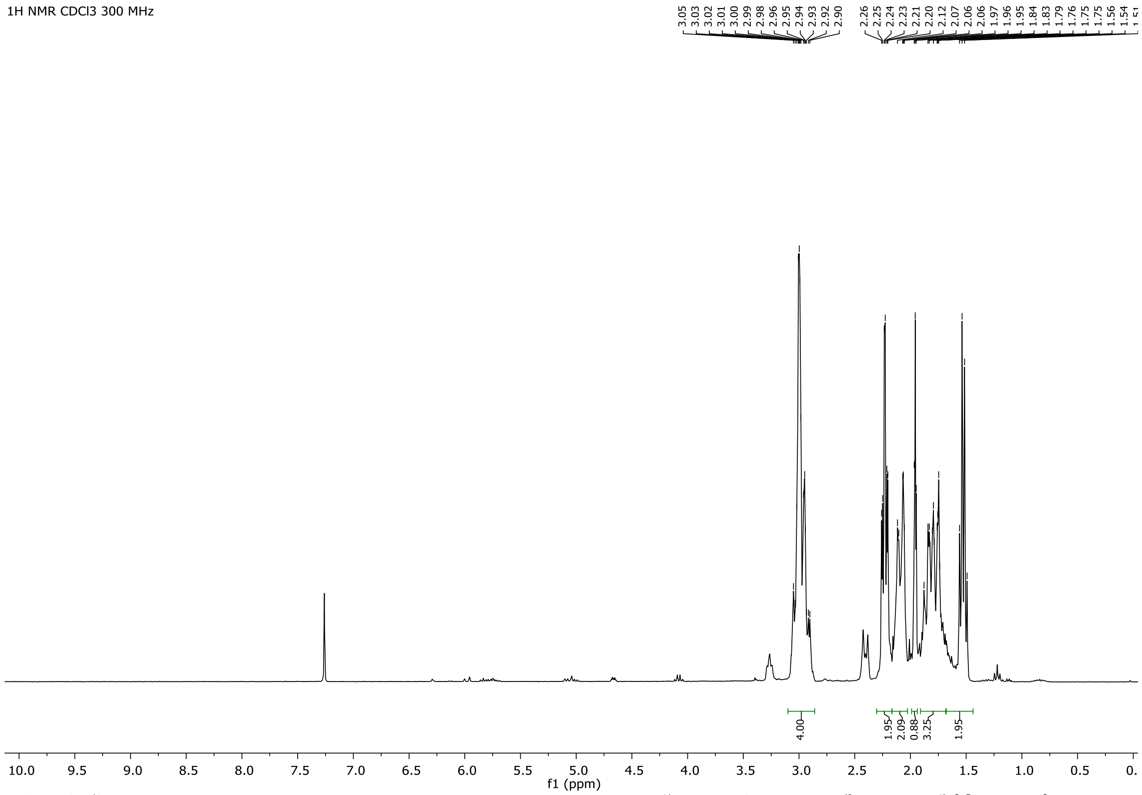

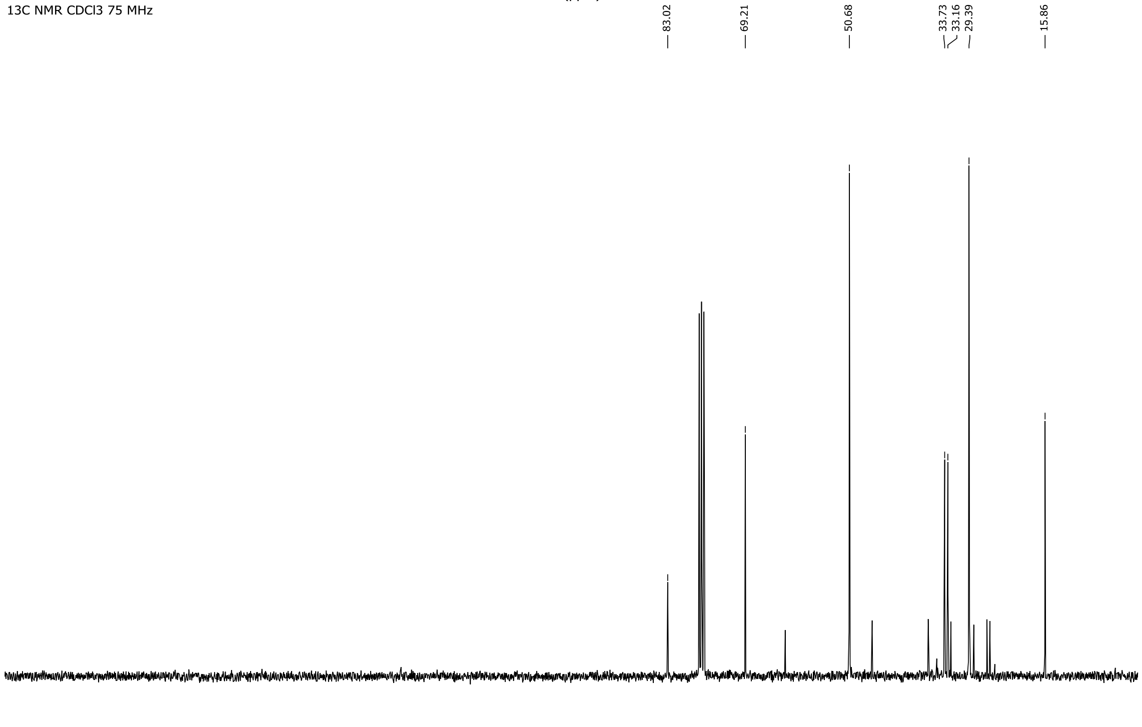

4-(4-bromobut-3-yn-1-yl)tetrahydro-2H-thiopyran 1,1-dioxide (11)

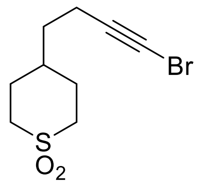

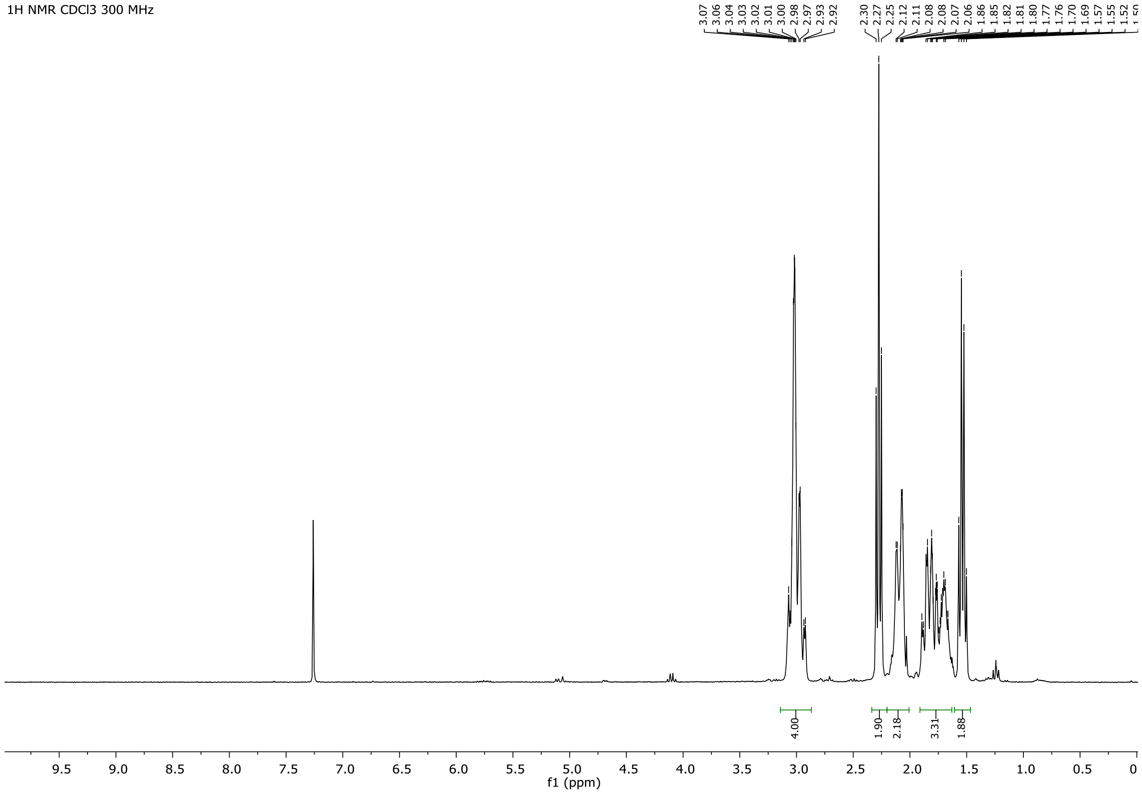

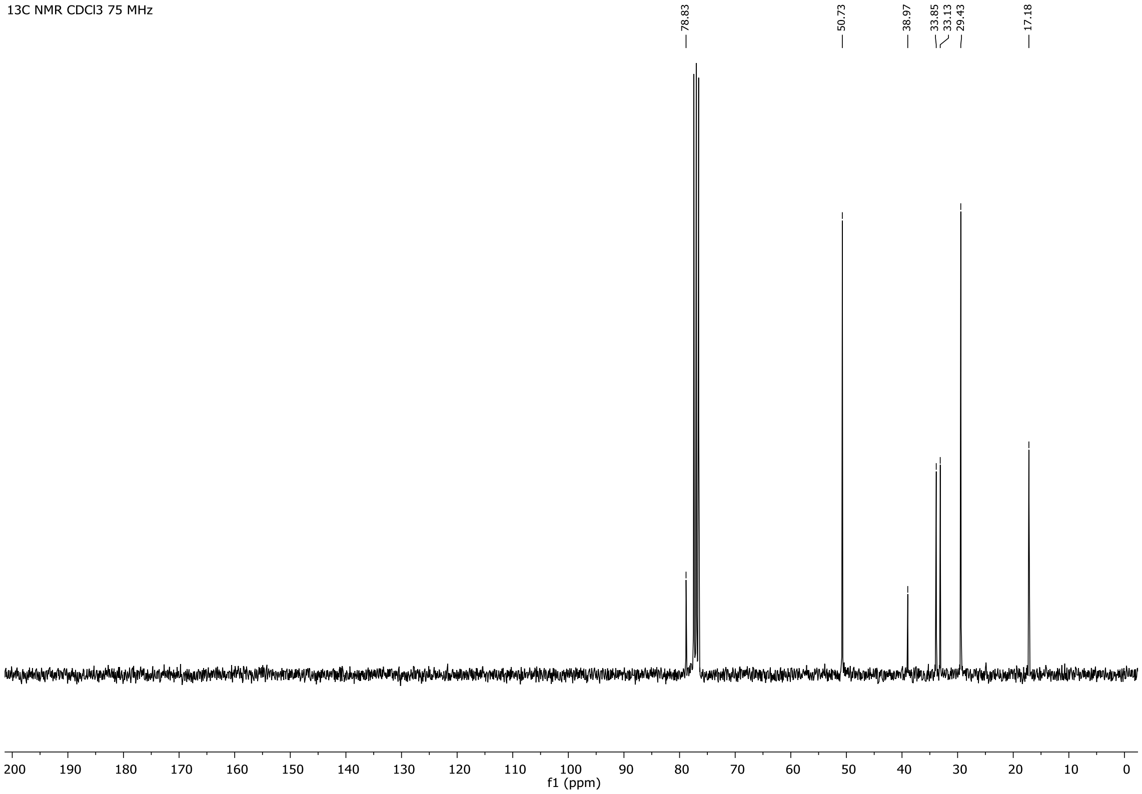

4-(2-bromoethyl)-1,1-dimethylcyclohexane (12-Br)

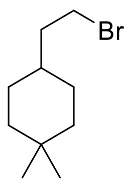

1H NMR CDCl3 300 MHz

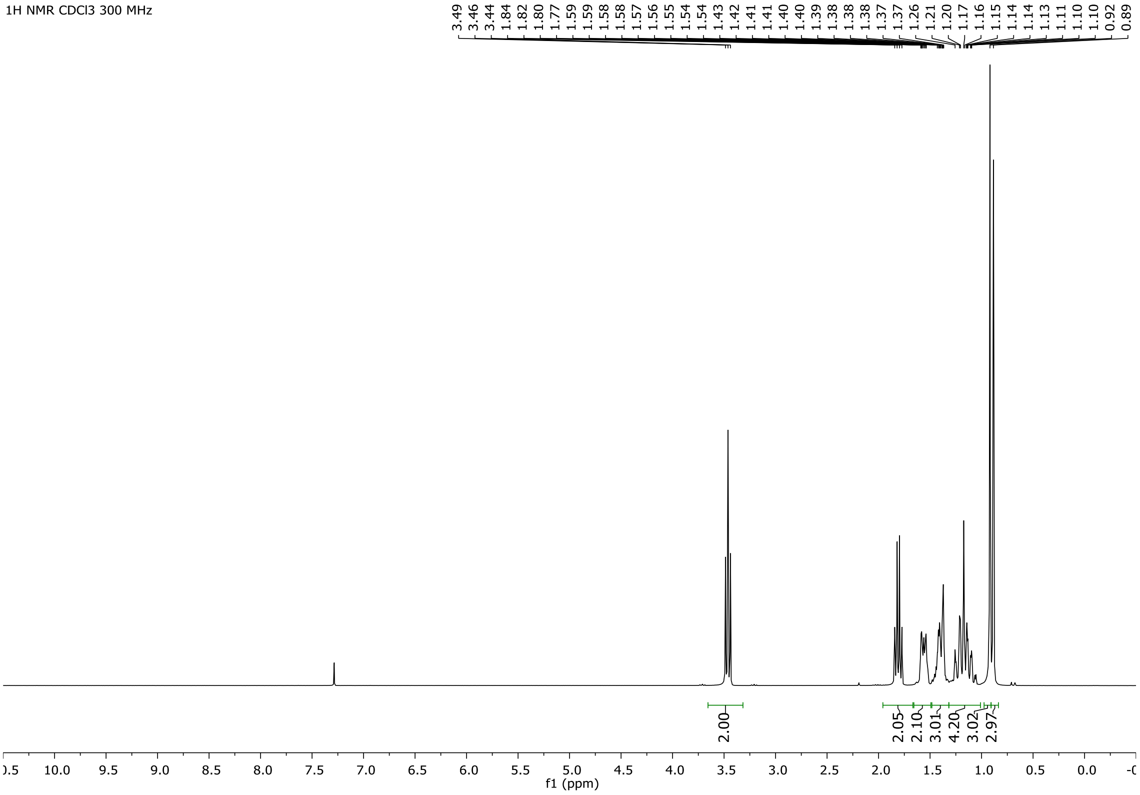

13C NMR CDCl3 75 MHz

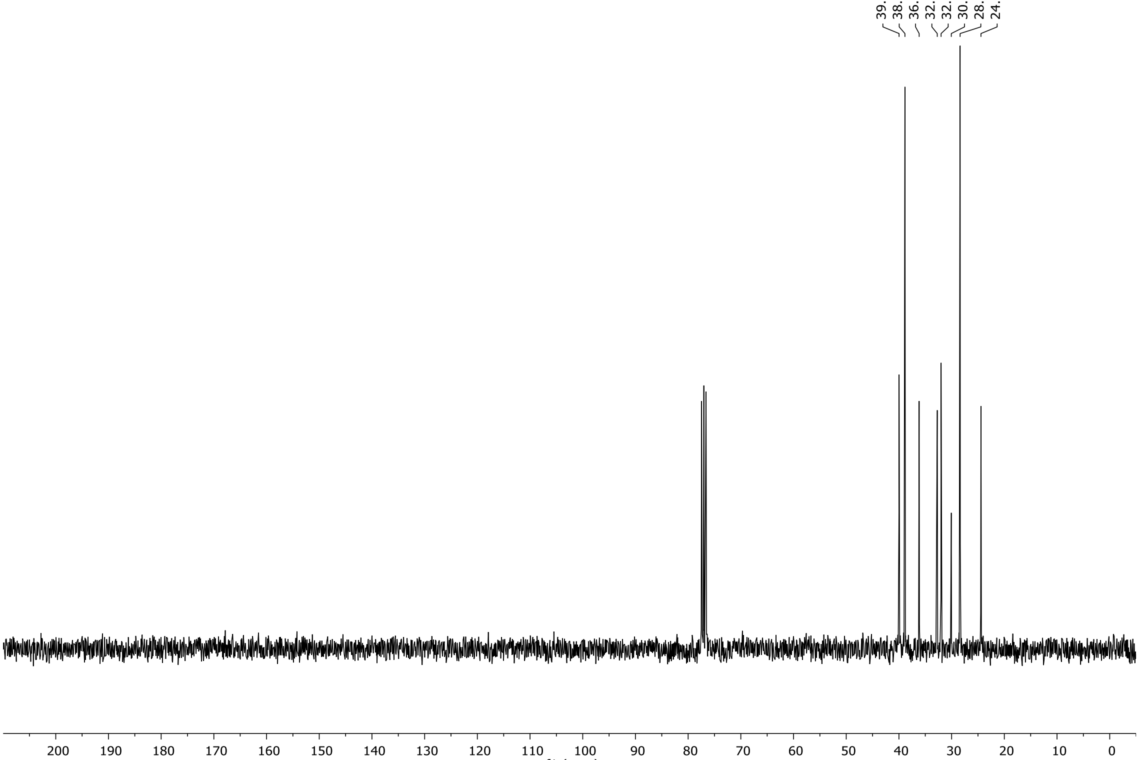

4-(but-3-yn-1-yl)-1,1-dimethylcyclohexane (12-CCH)

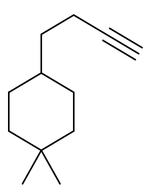

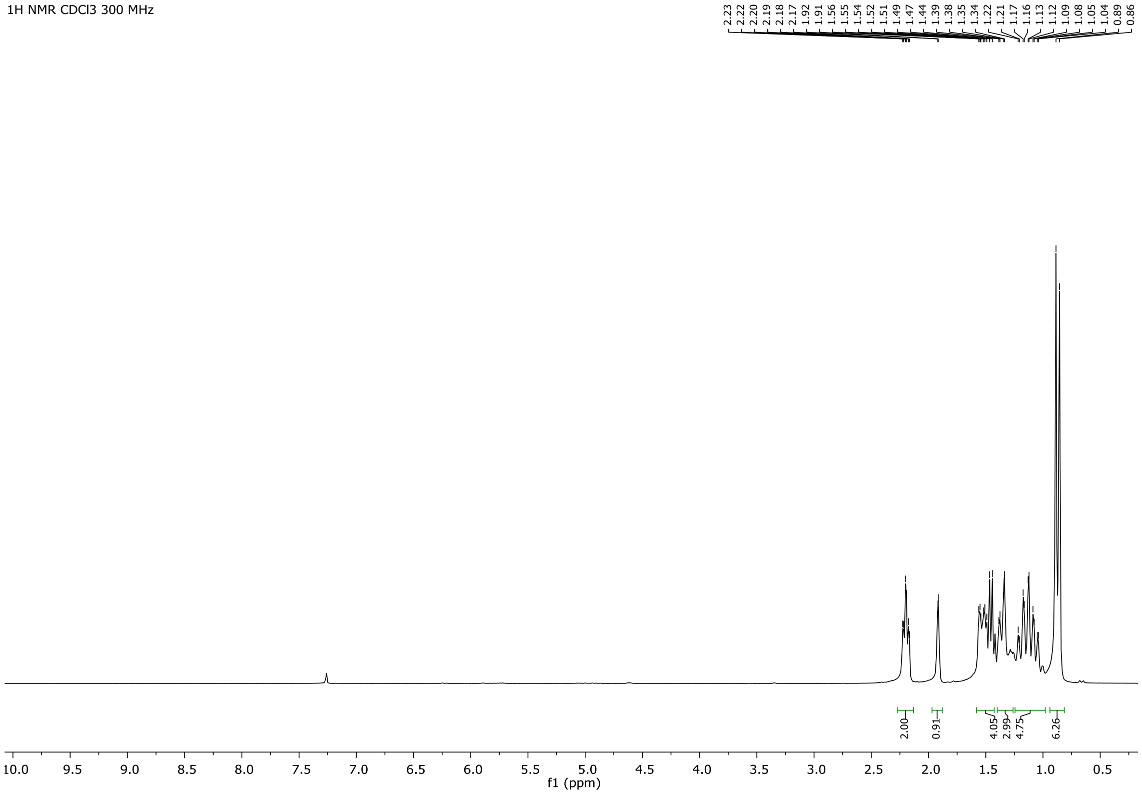

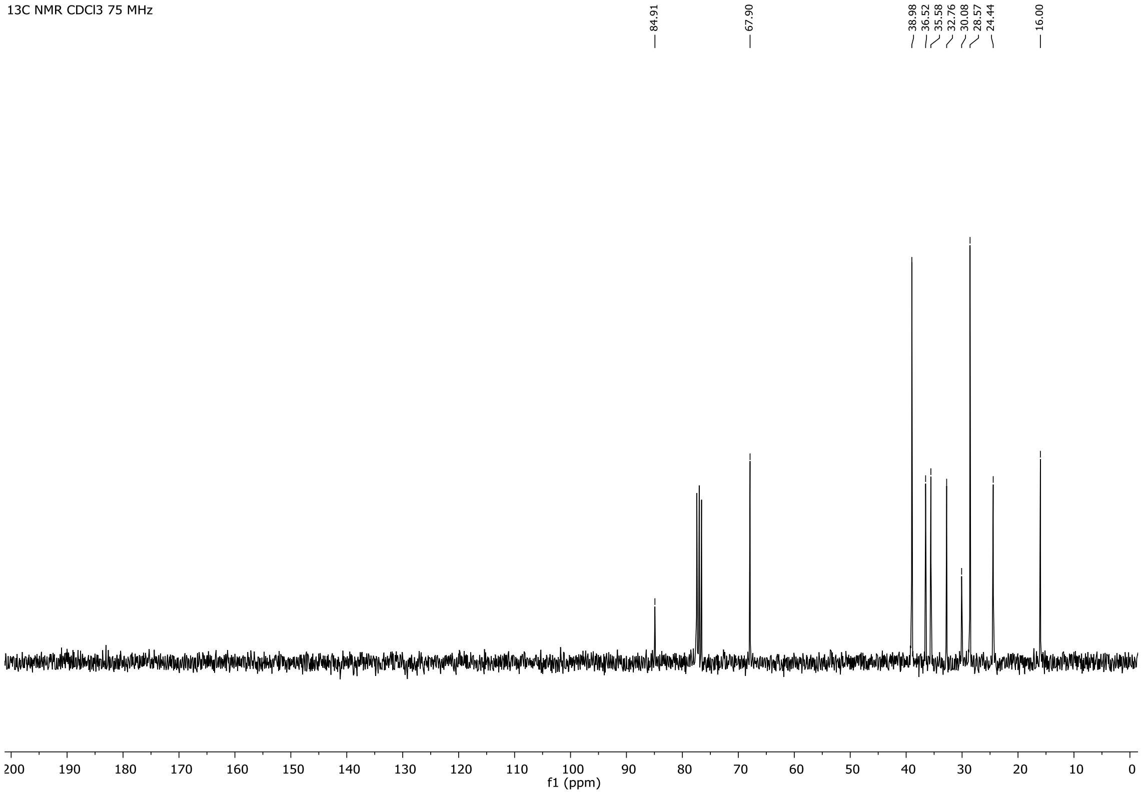

4-(4-bromobut-3-yn-1-yl)-1,1-dimethylcyclohexane (12)

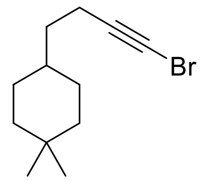

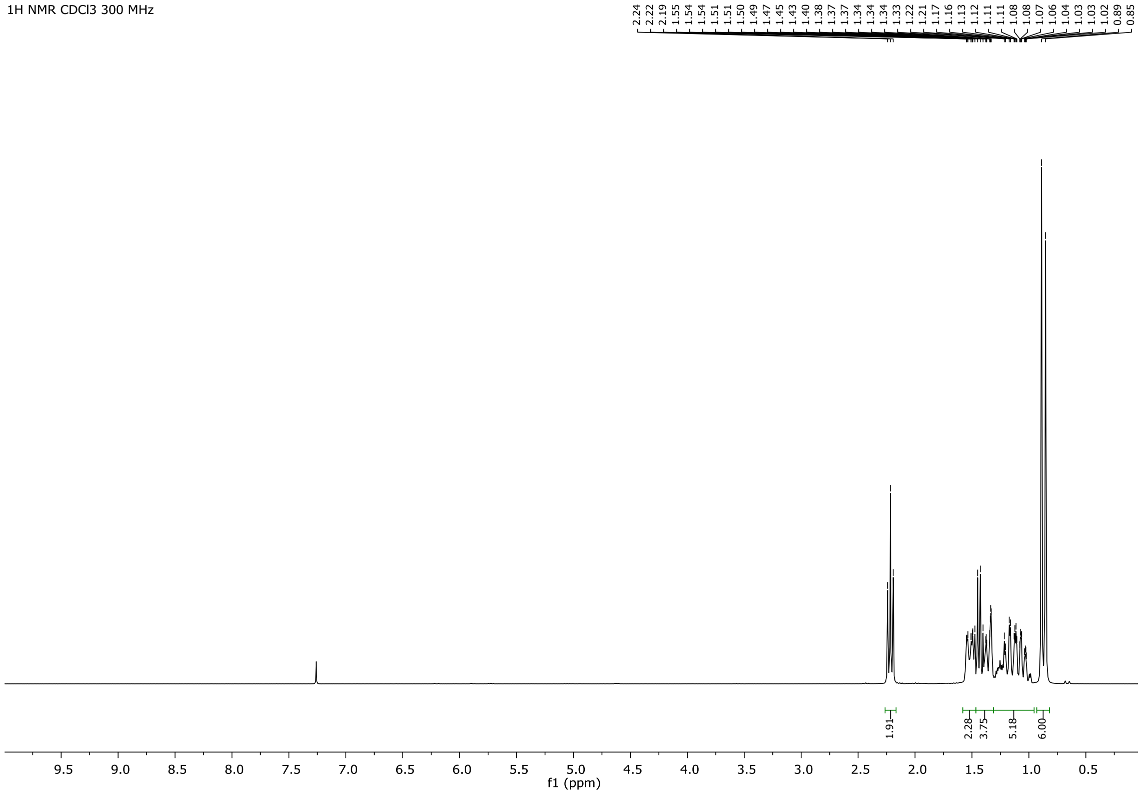

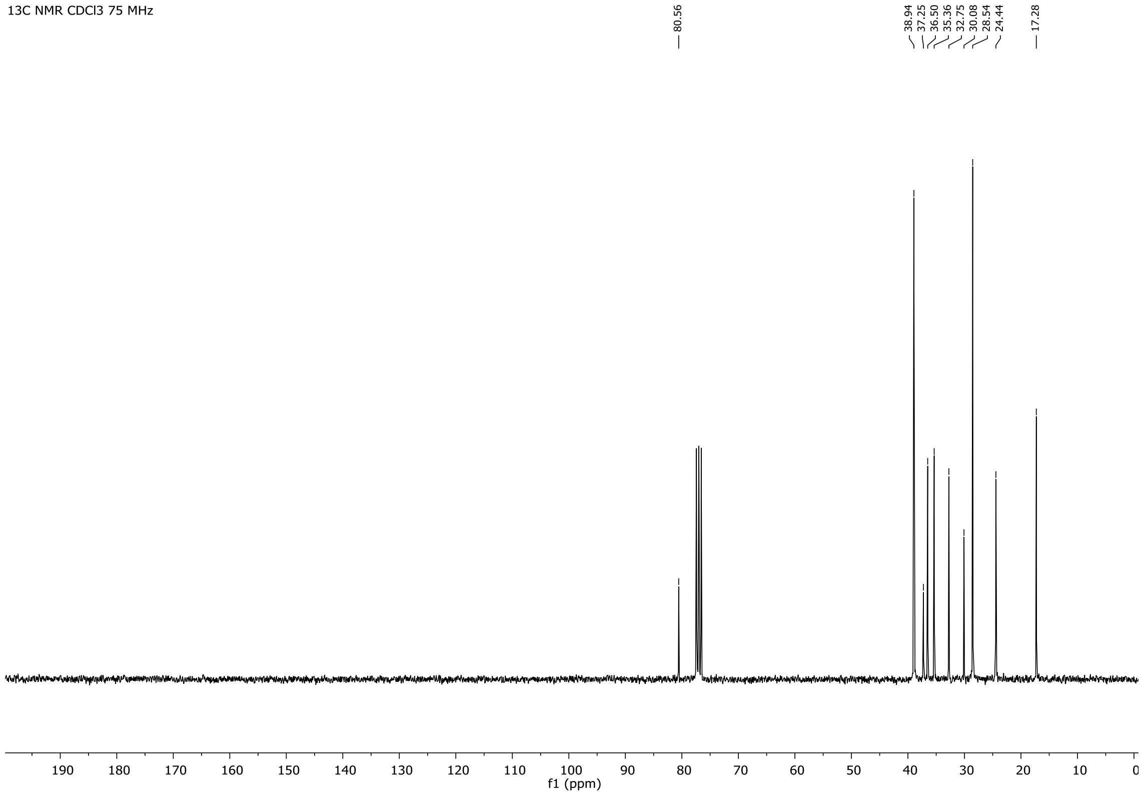

2-(oxepan-4-yl)ethan-1-ol (13-OH)

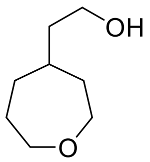

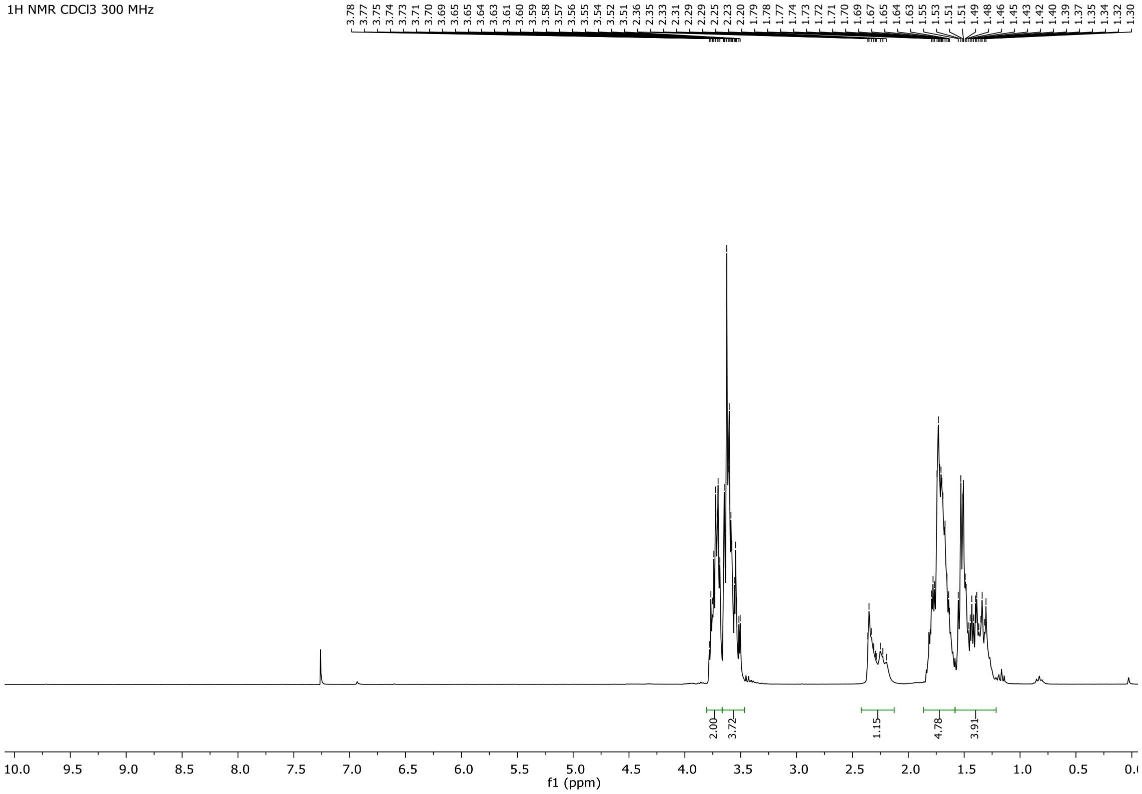

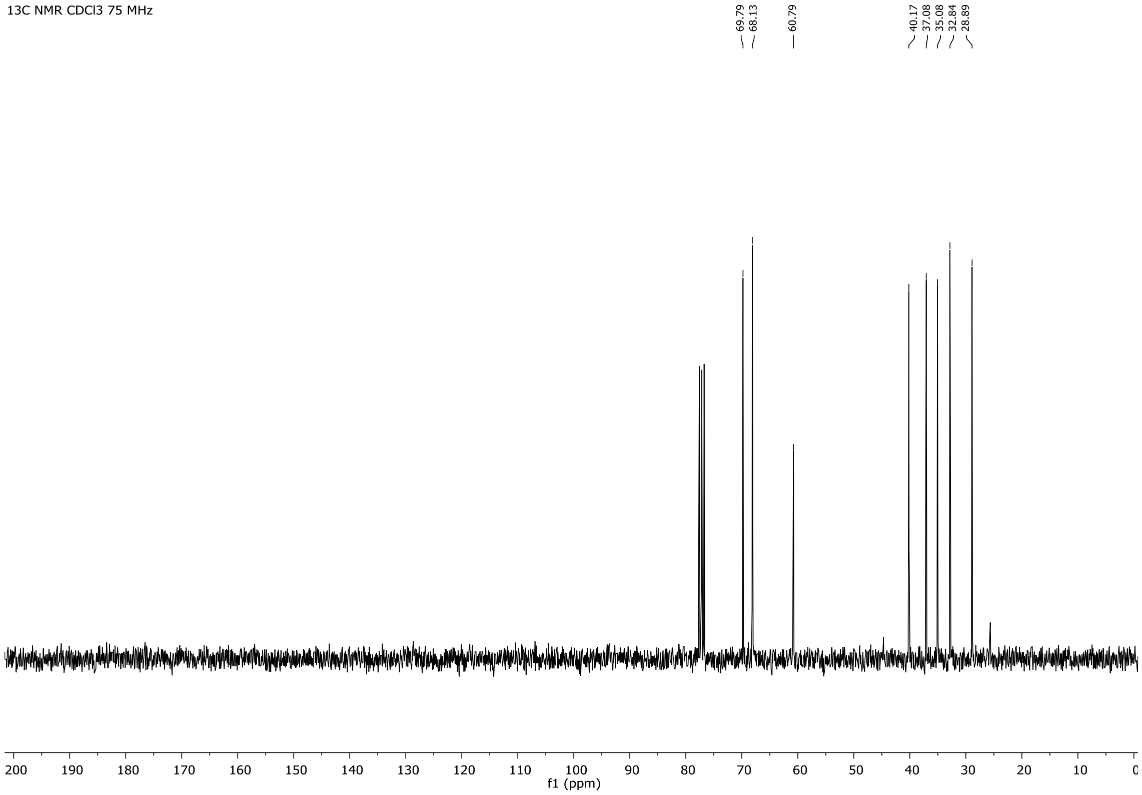

4-(2-bromoethyl)oxepane (13-Br)

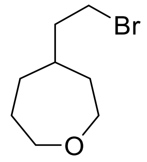

1H NMR CDCl3 300 MHz

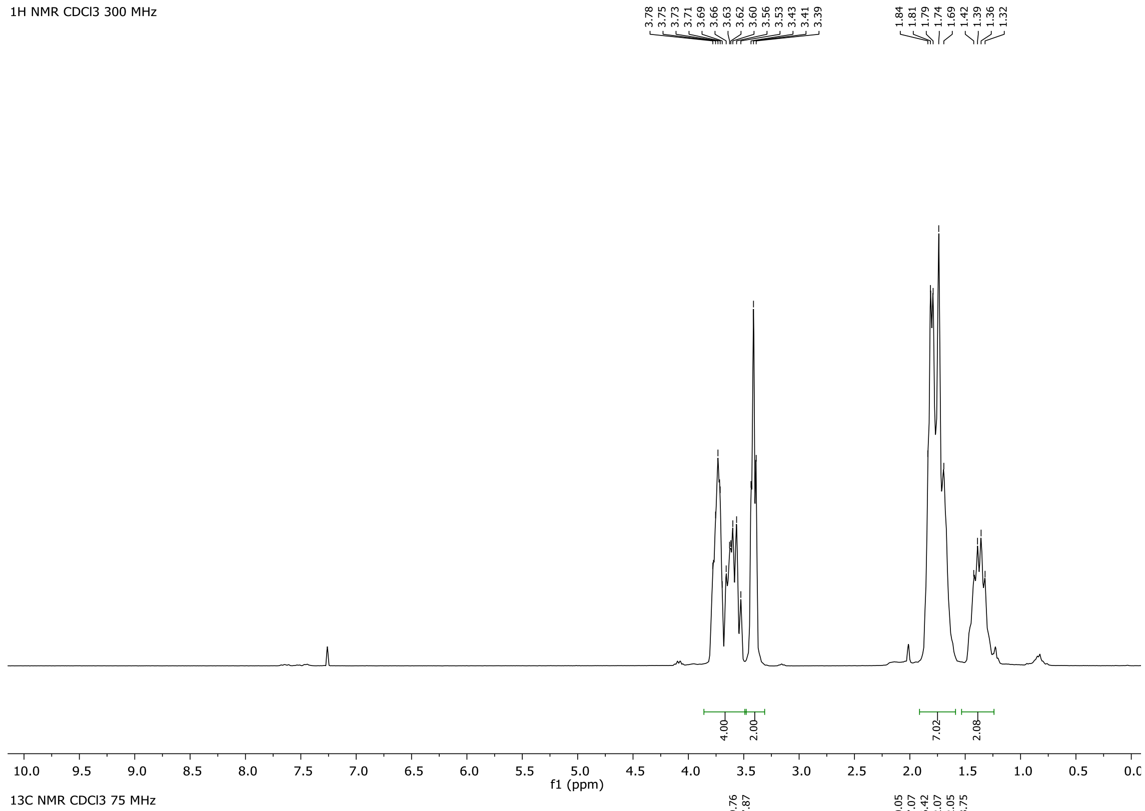

13C NMR CDCl3 75 MHz

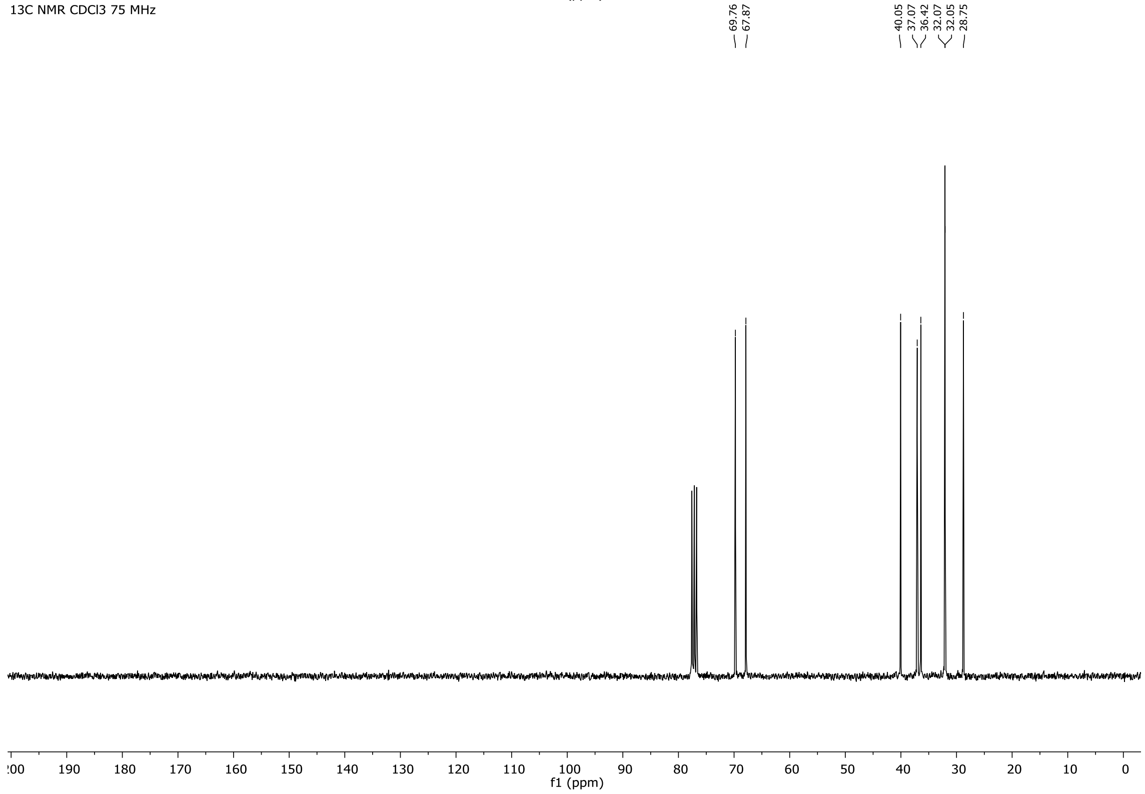

4-(but-3-yn-1-yl)oxepane (13-CCH)

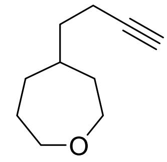

1H NMR CDCl3 300 MHz

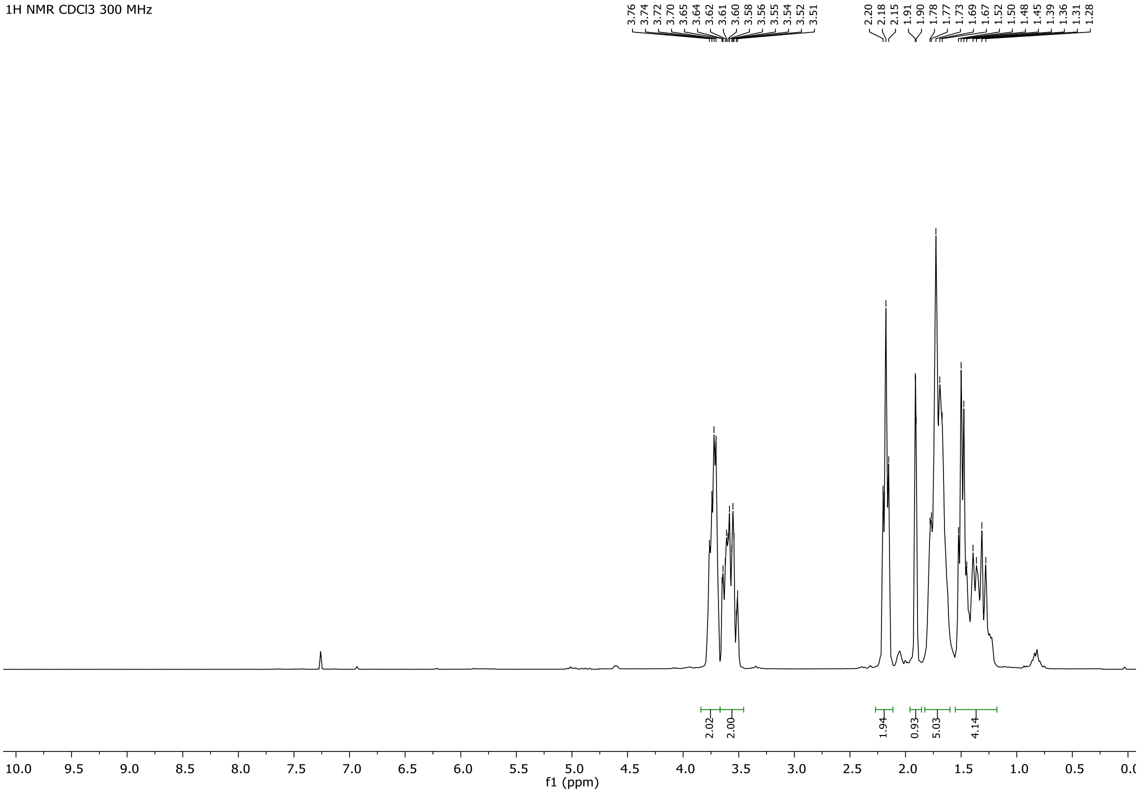

13C NMR CDCl3 75 MHz

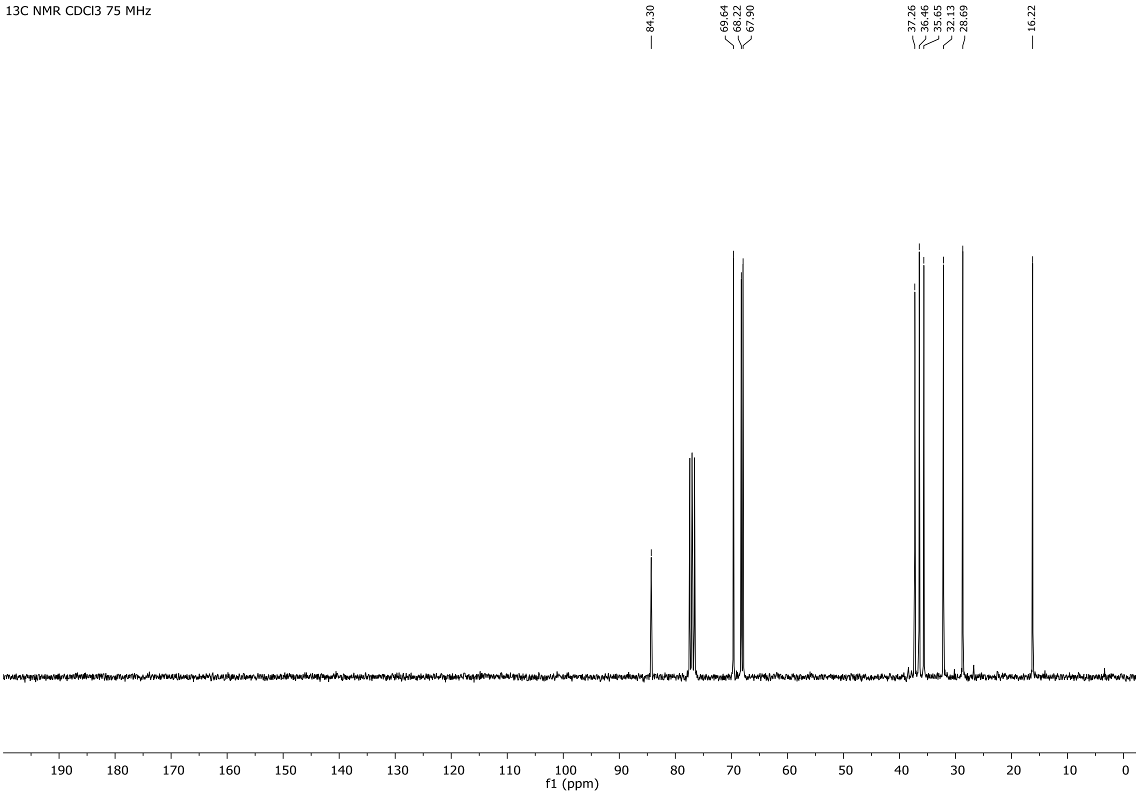

4-(4-bromobut-3-yn-1-yl)oxepane (13)

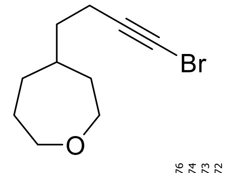

1H NMR CDCl3 300 MHz

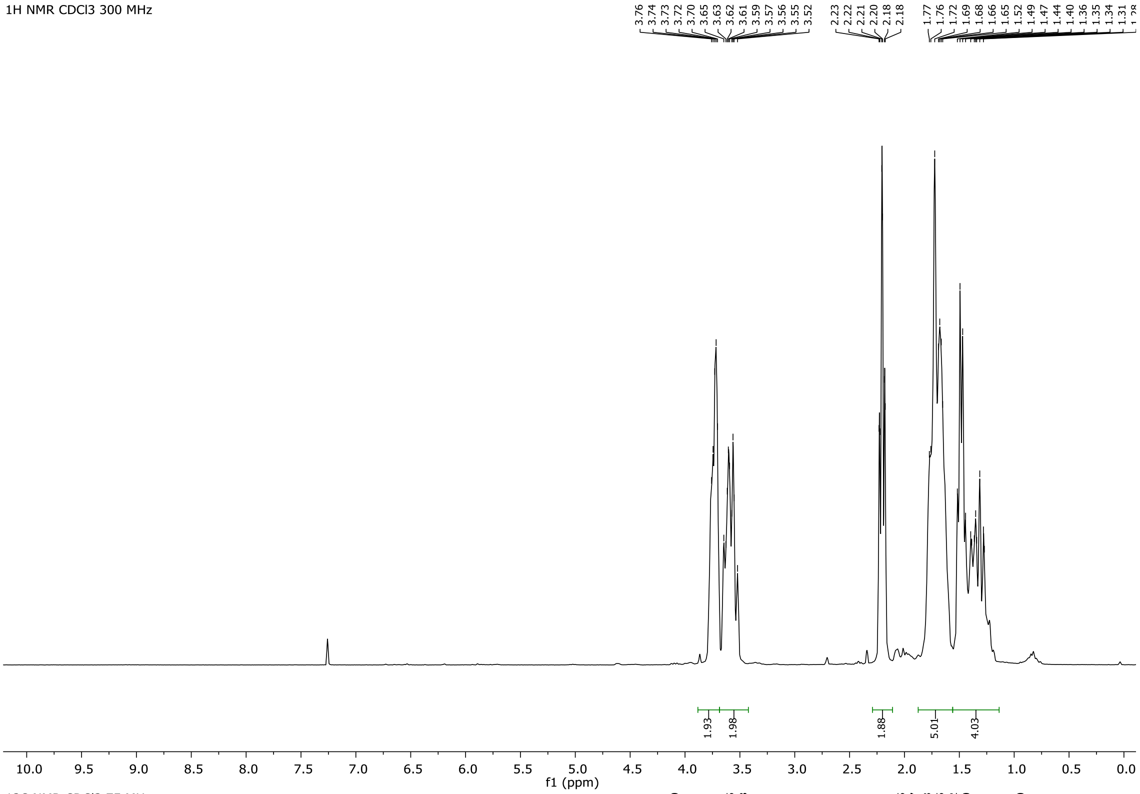

13C NMR CDCl3 75 MHz

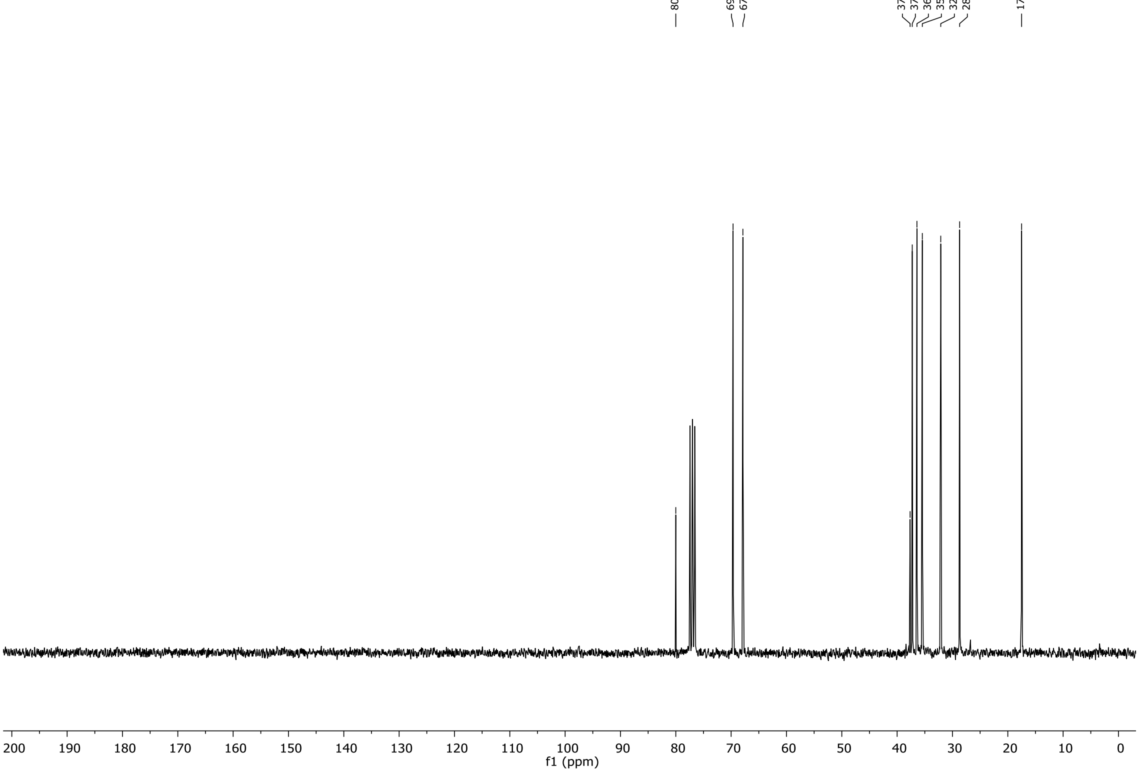

5-(4-bromobut-3-yn-1-yl)oxepan-2-one (14)

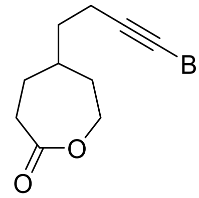

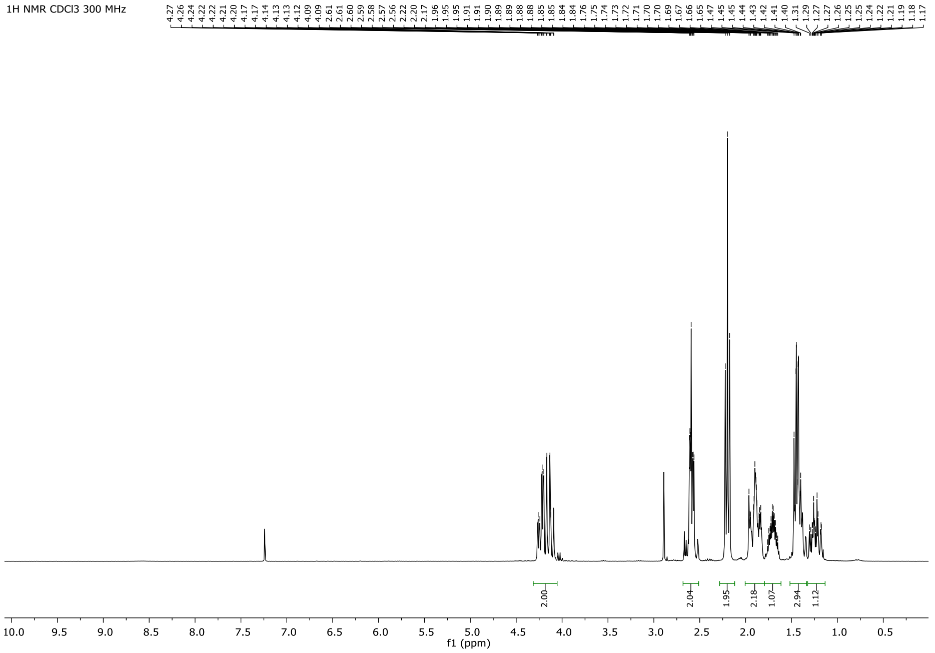

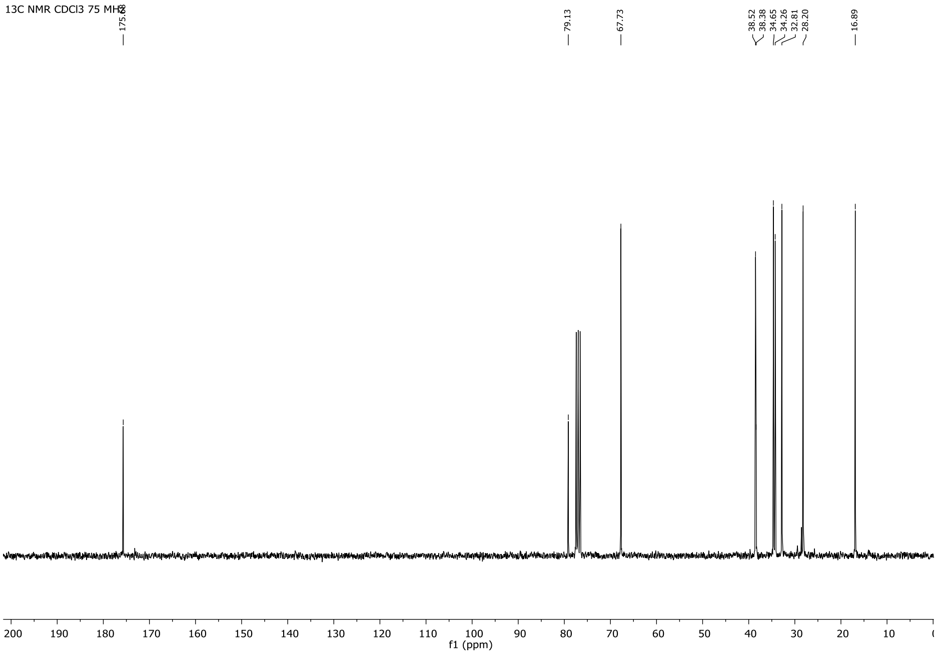

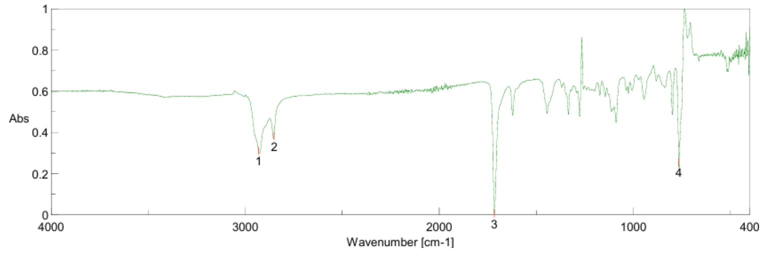

2-(6,6-difluorospiro[3.3]heptan-2-yl)ethan-1-ol (15-OH)

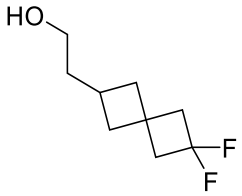

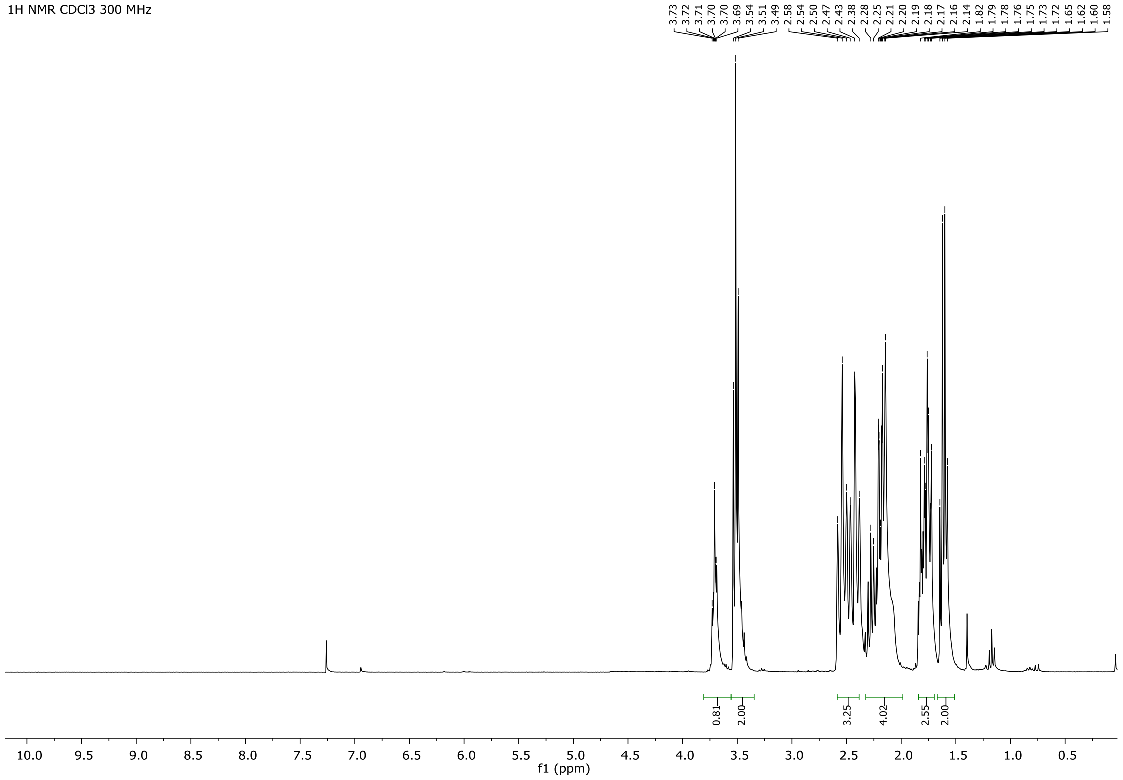

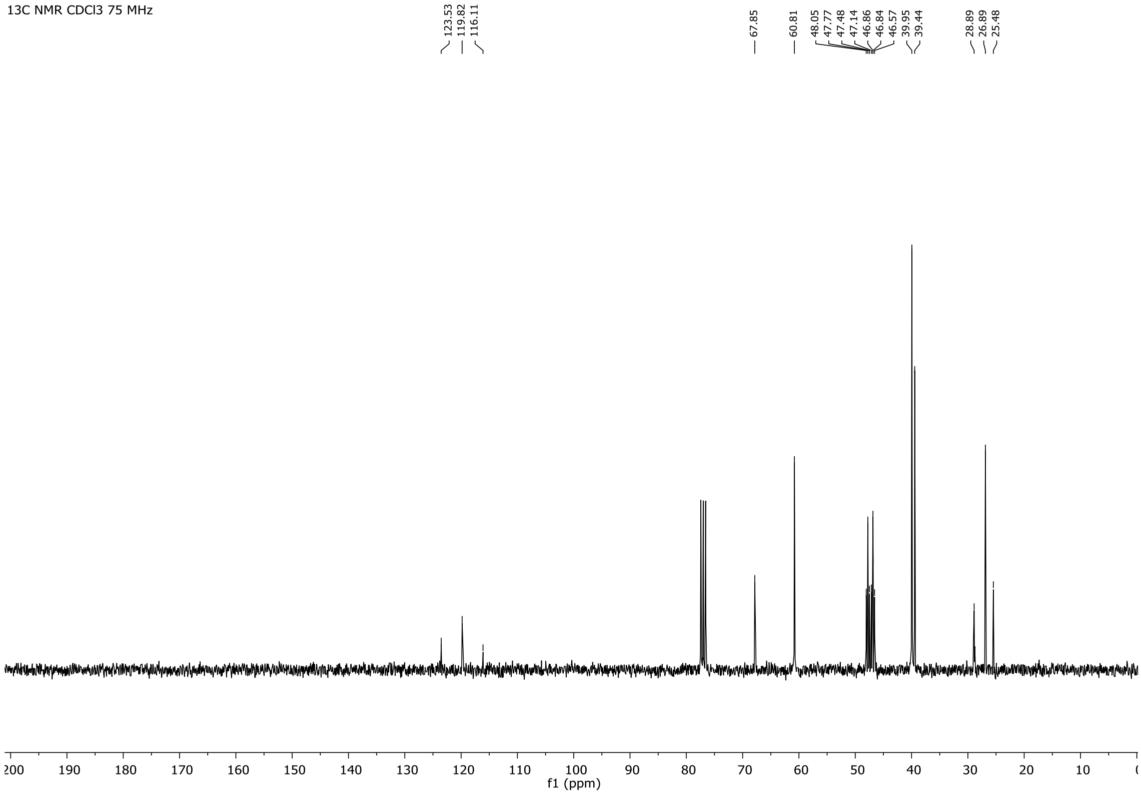

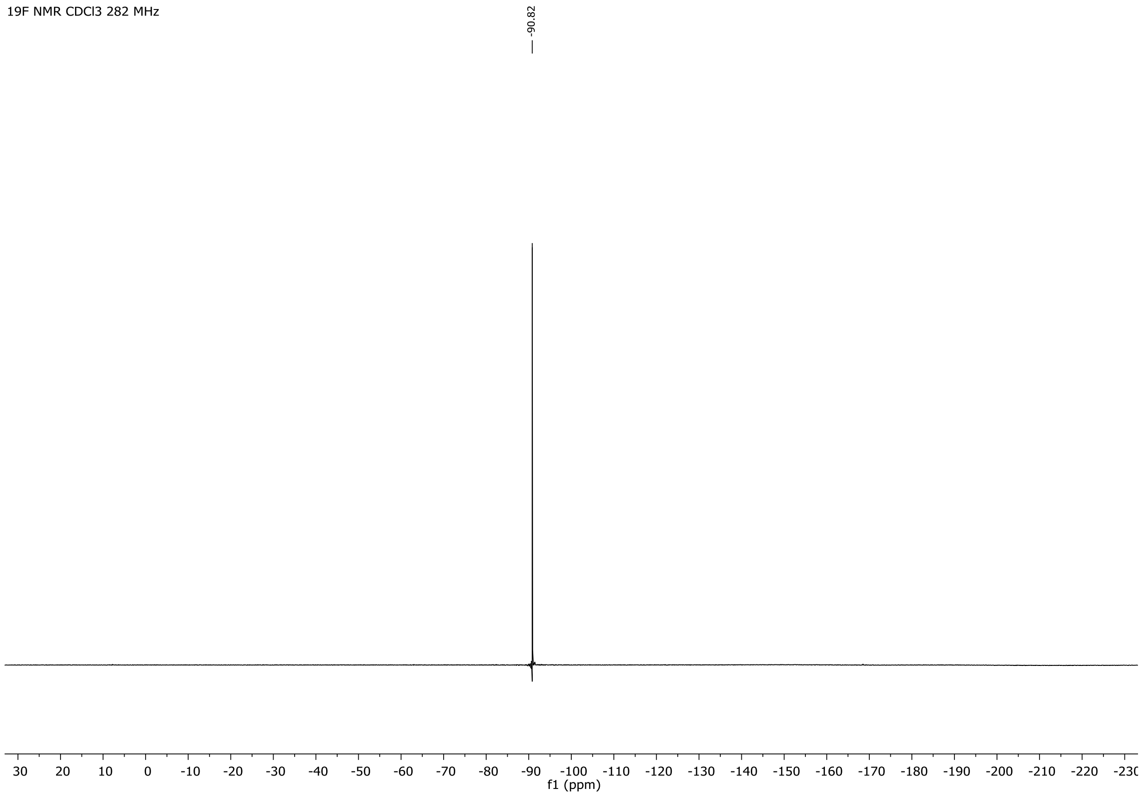
19F NMR CDCl3 282 MHz
-90.82
30 20 10 0 -10 -20 -30 -40 -50 -60 -70 -80 -90 -100 -110 -120 -130 -140 -150 -160 -170 -180 -190 -200 -210 -220 -230
f1 (ppm)

6-(2-bromoethyl)-2,2-difluorospiro[3.3]heptane (15-Br)

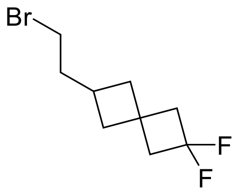

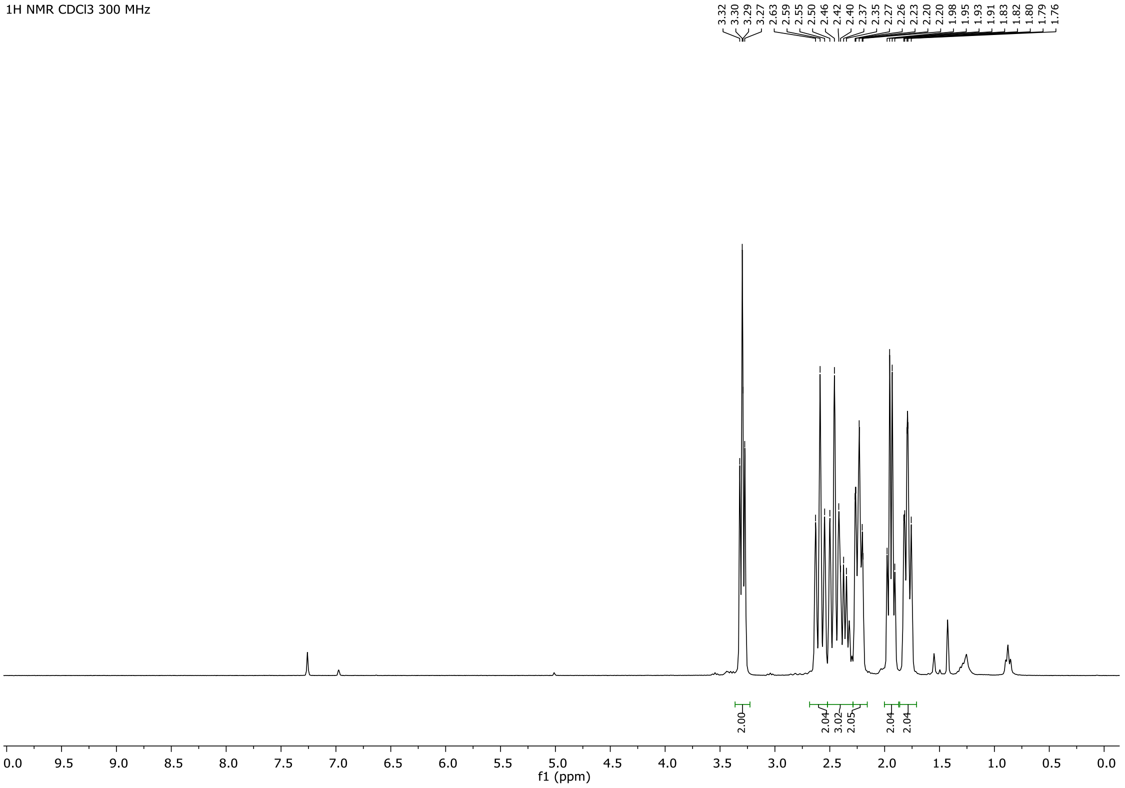

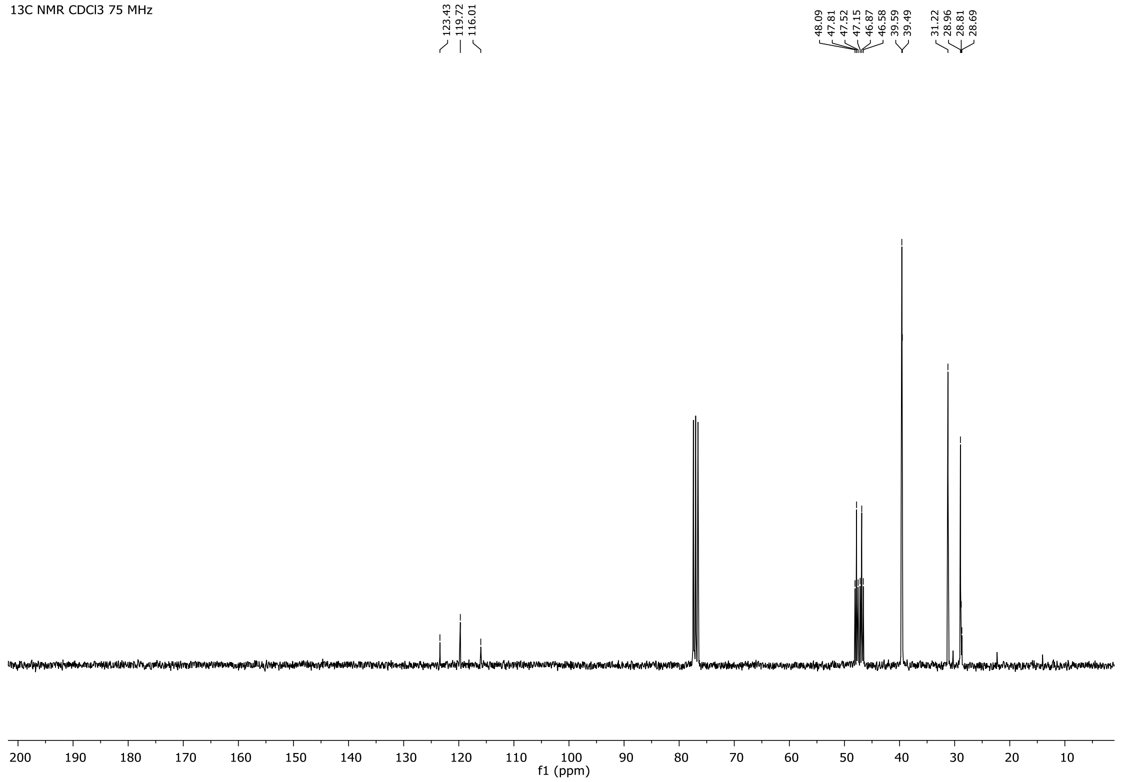

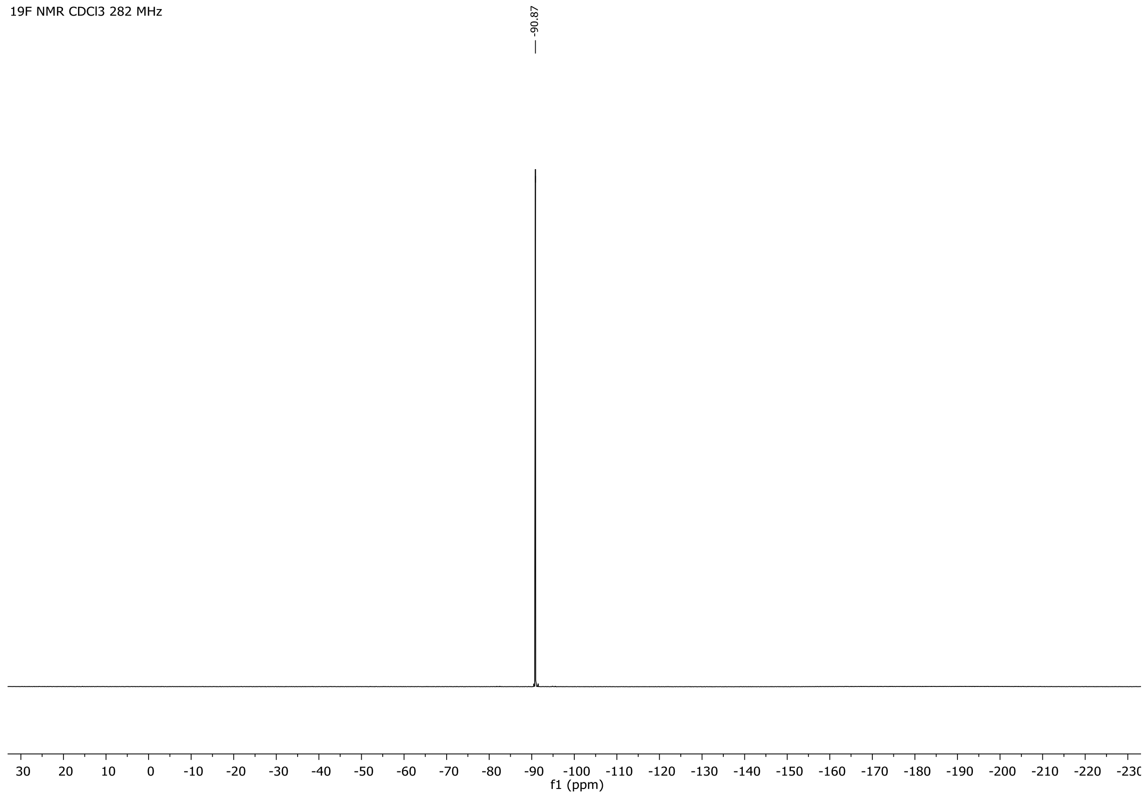
19F NMR CDCl3 282 MHz
-90.87
30
20
10
0
-10
-20
-30
-40
-50
-60
-70
-80
-90
-100
-110
-120
-130
-140
-150
-160
-170
-180
-190
-200
-210
-220
-230
f1 (ppm)

6-(but-3-yn-1-yl)-2,2-difluorospiro[3.3]heptane (15-CCH)

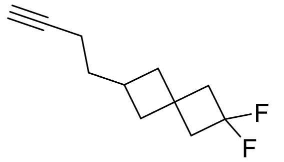

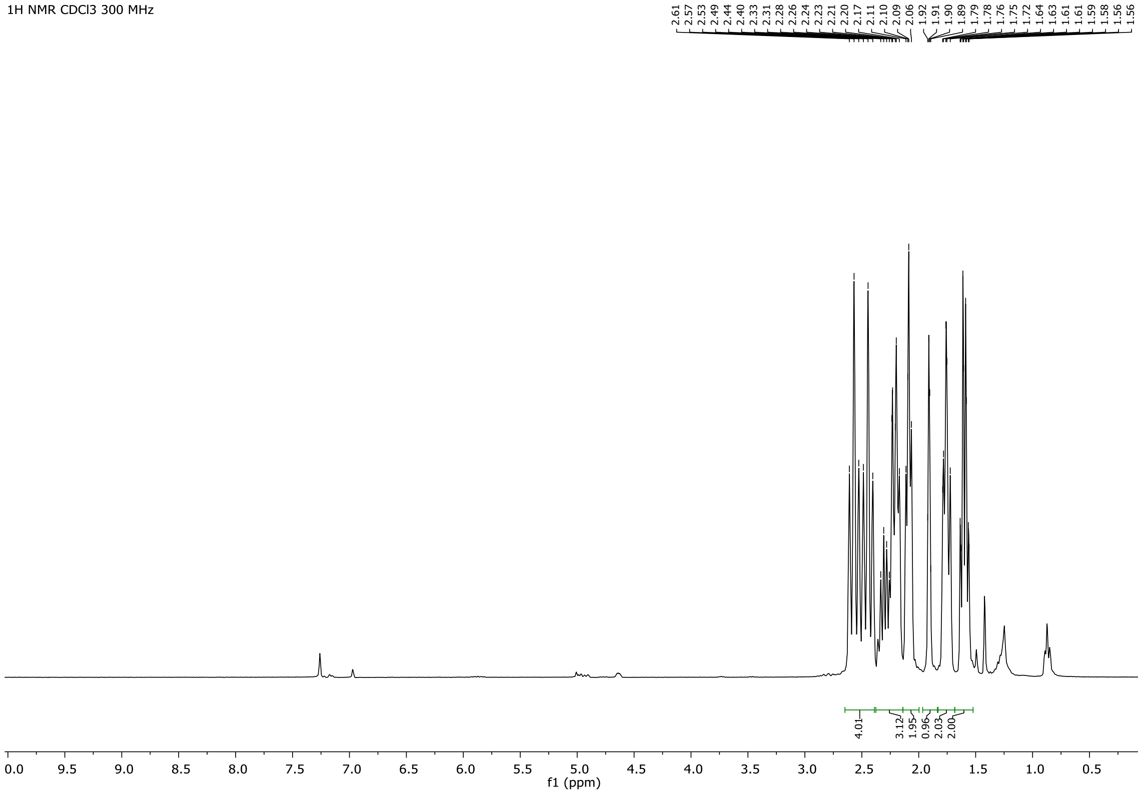

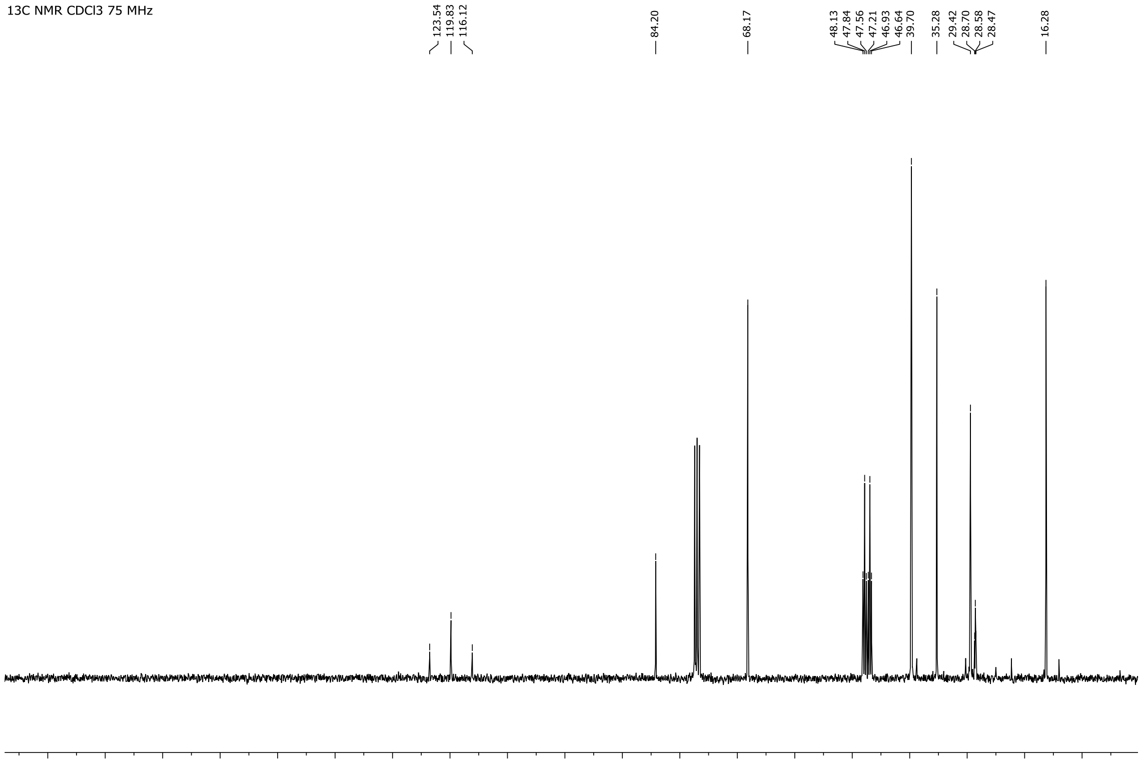

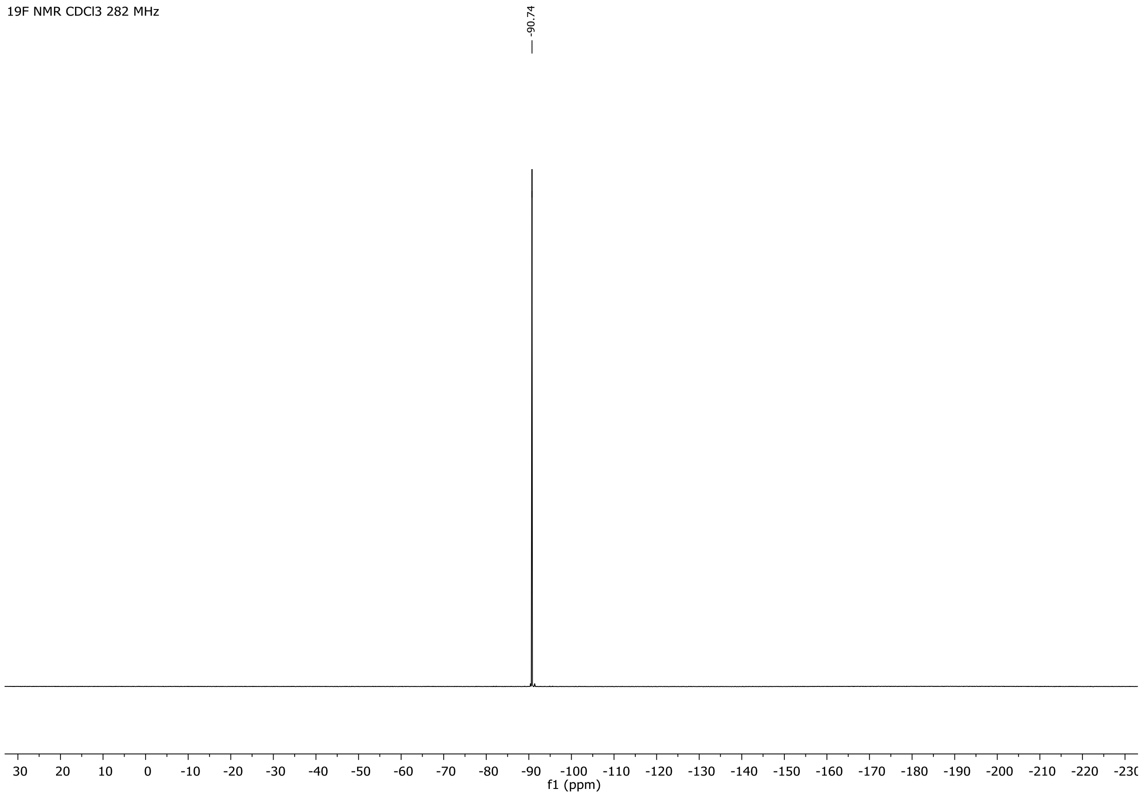
19F NMR CDCl3 282 MHz
-90.74
30
20
10
0
-10
-20
-30
-40
-50
-60
-70
-80
-90
-100
-110
-120
-130
-140
-150
-160
-170
-180
-190
-200
-210
-220
-230
f1 (ppm)

6-(4-bromobut-3-yn-1-yl)-2,2-difluorospiro[3.3]heptane (15)

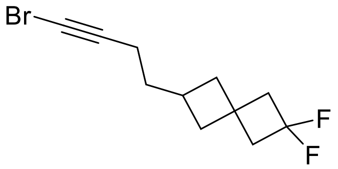

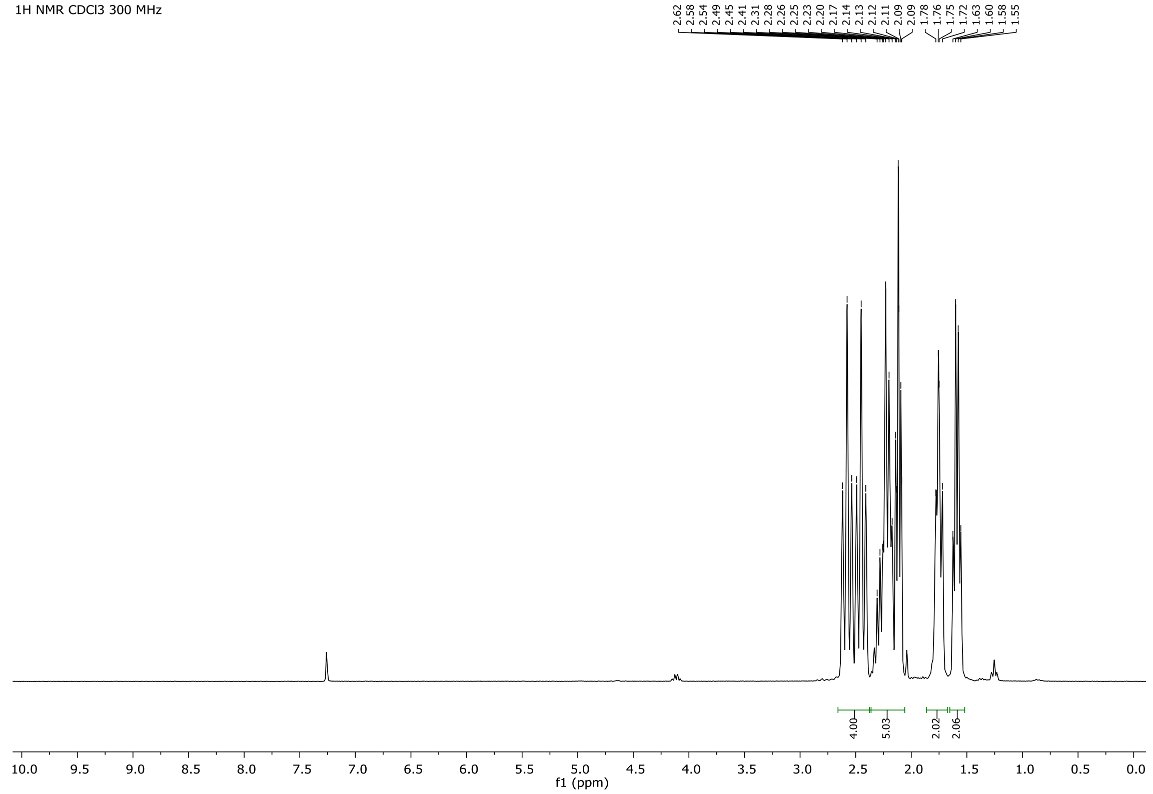

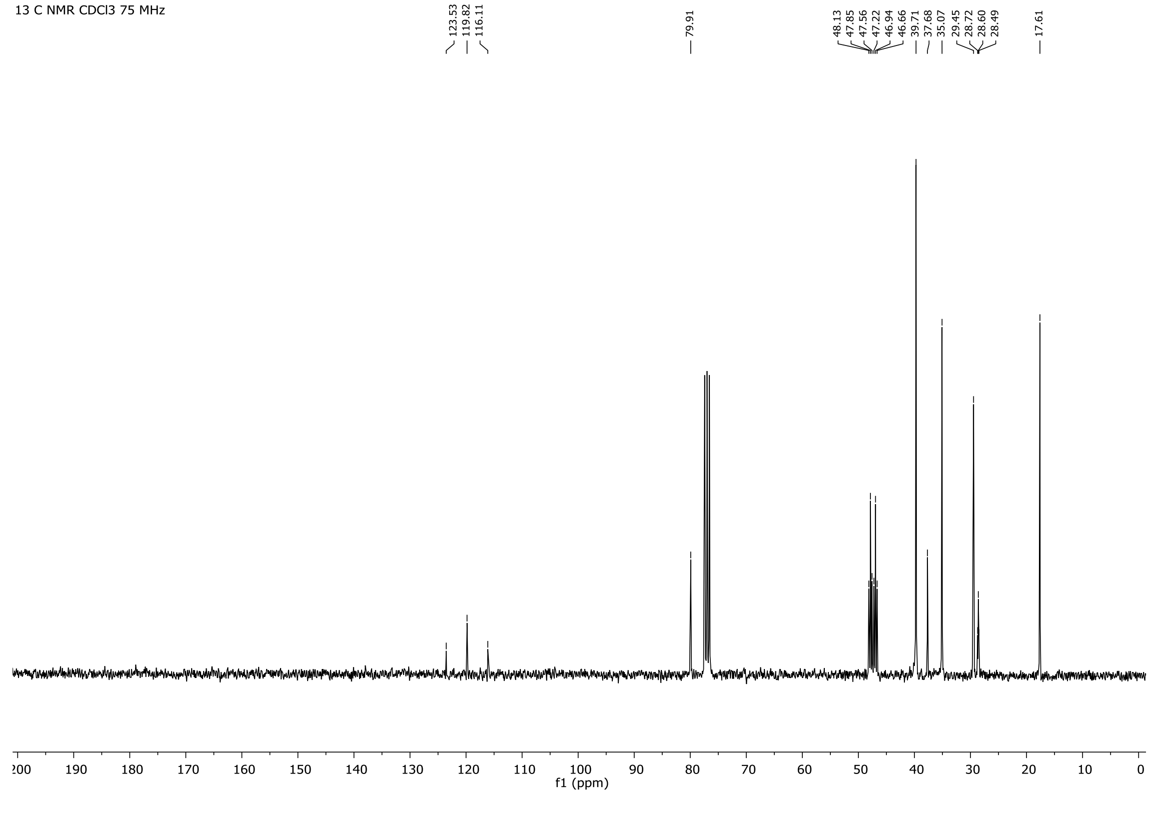

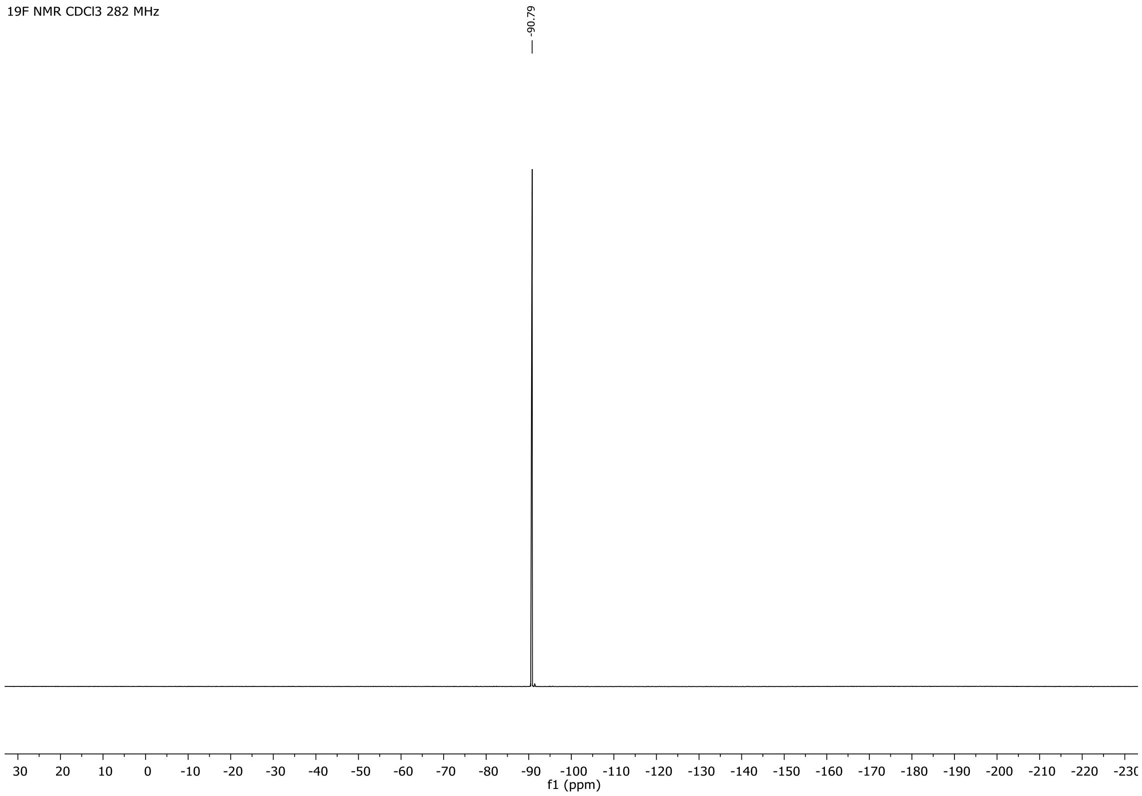

19F NMR CDCl3 282 MHz
-90.79
30 20 10 0 -10 -20 -30 -40 -50 -60 -70 -80 -90 -100 -110 -120 -130 -140 -150 -160 -170 -180 -190 -200 -210 -220 -230
f1 (ppm)

1-((tetrahydro-2H-pyran-4-yl)methyl)cyclopentane-1-carboxylic acid (16-CO_2H)

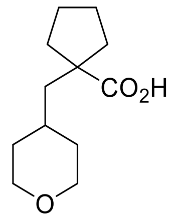

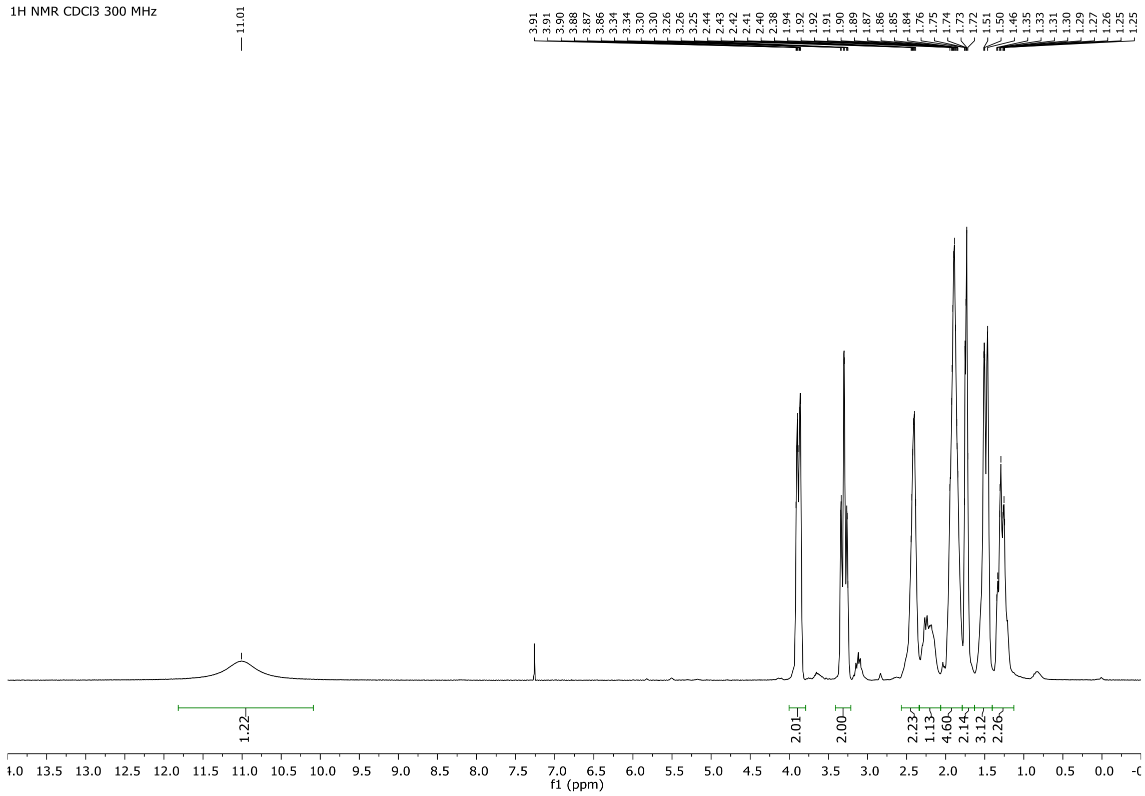

(1-((tetrahydro-2H-pyran-4-yl)methyl)cyclopentyl)methanol (16-OH)

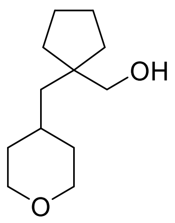

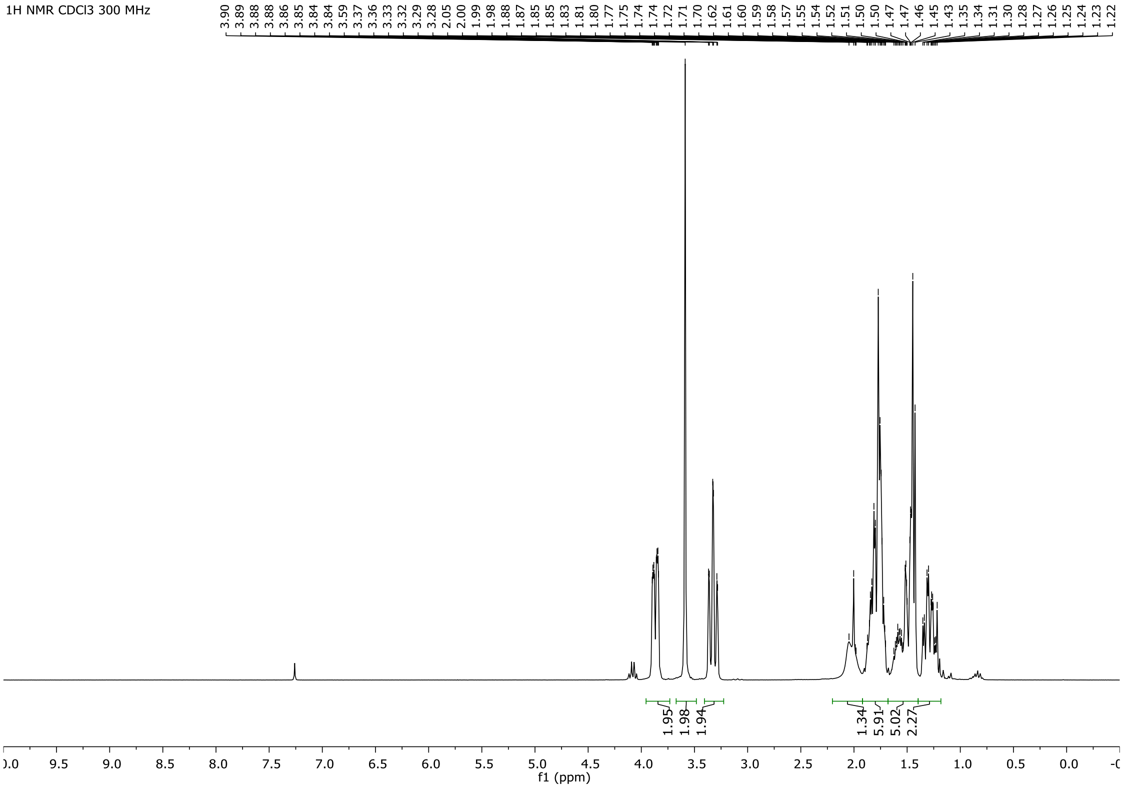

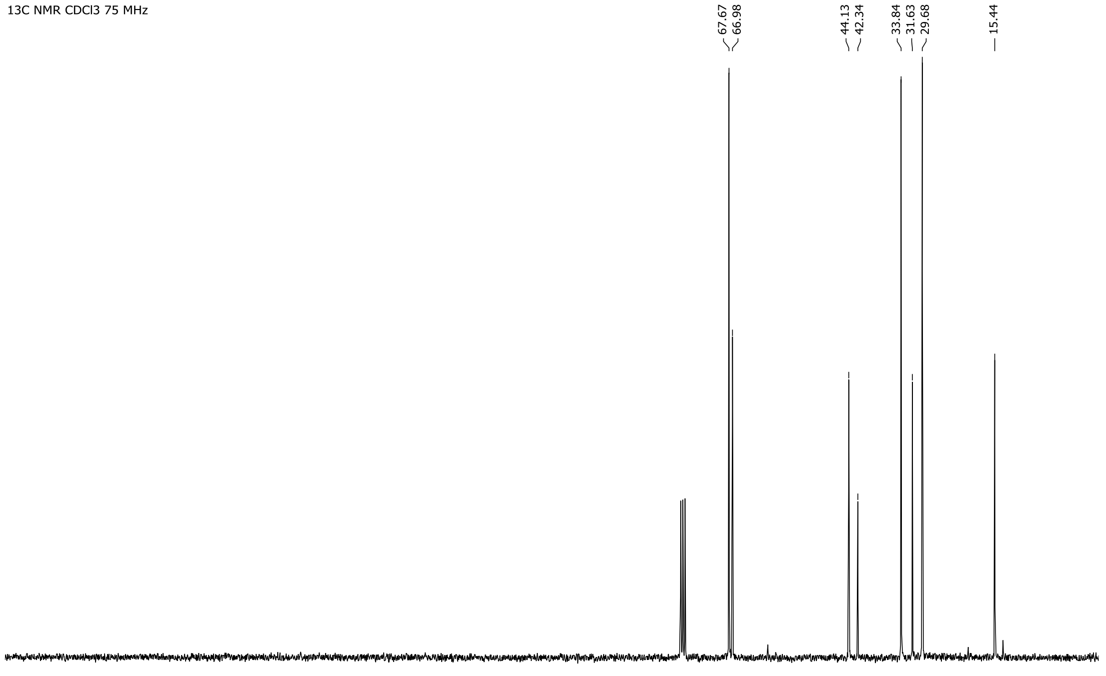

1-((tetrahydro-2H-pyran-4-yl)methyl)cyclopentane-1-carbaldehyde (16-CHO)

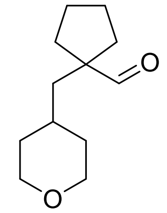

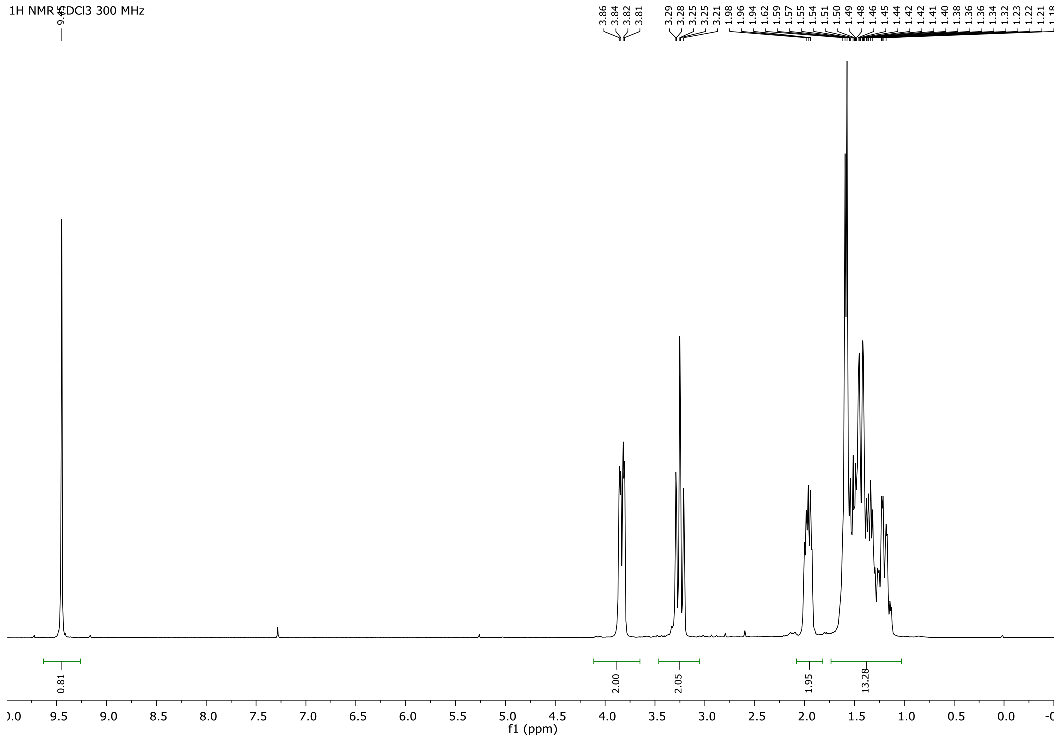

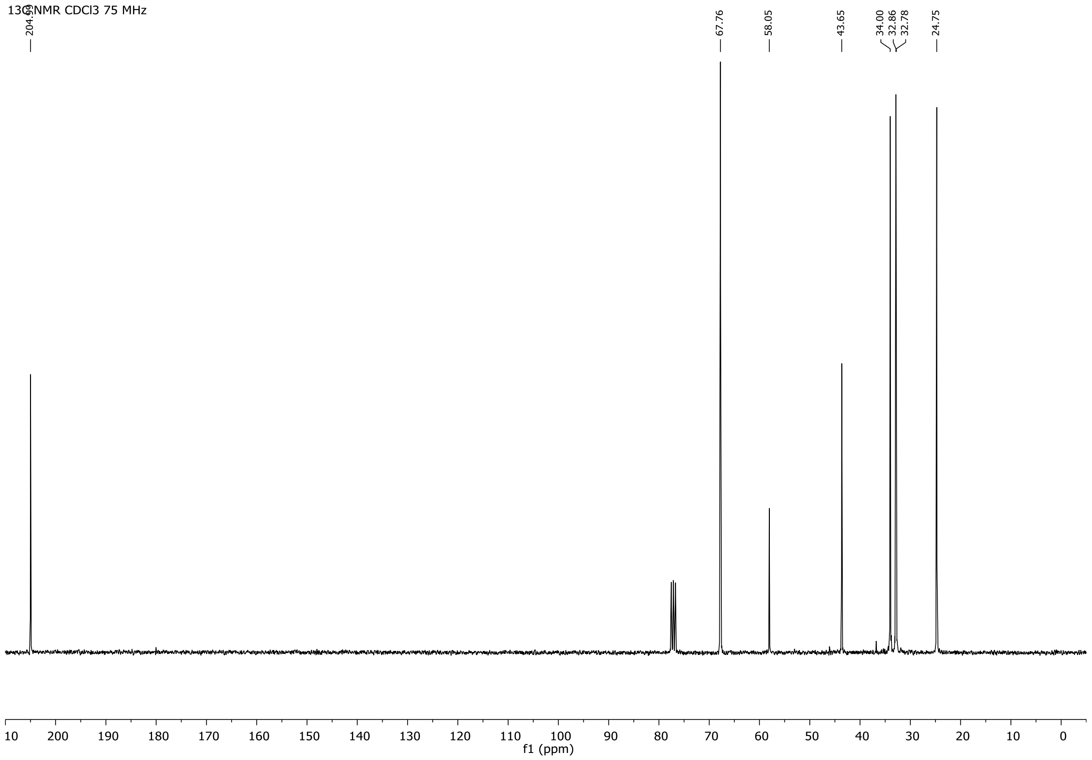

4-((1-(2,2-dibromovinyl)cyclopentyl)methyl)tetrahydro-2H-pyran (16-C=CBr$_2$)

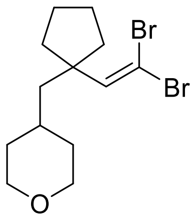

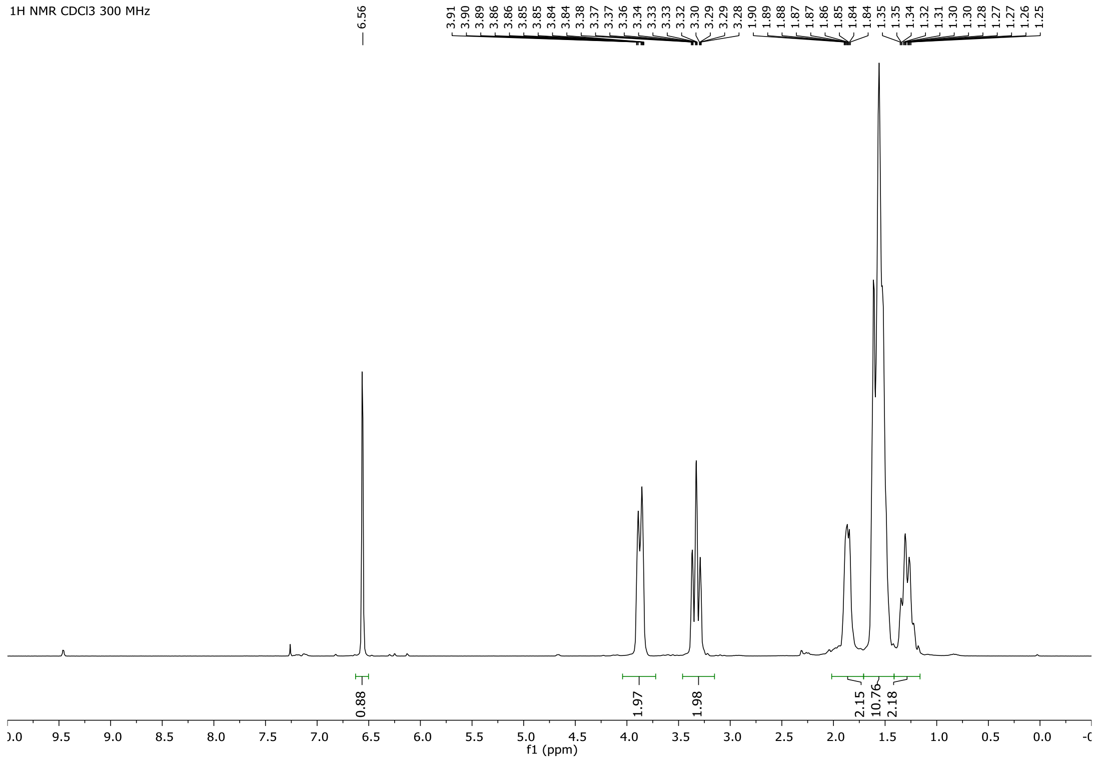

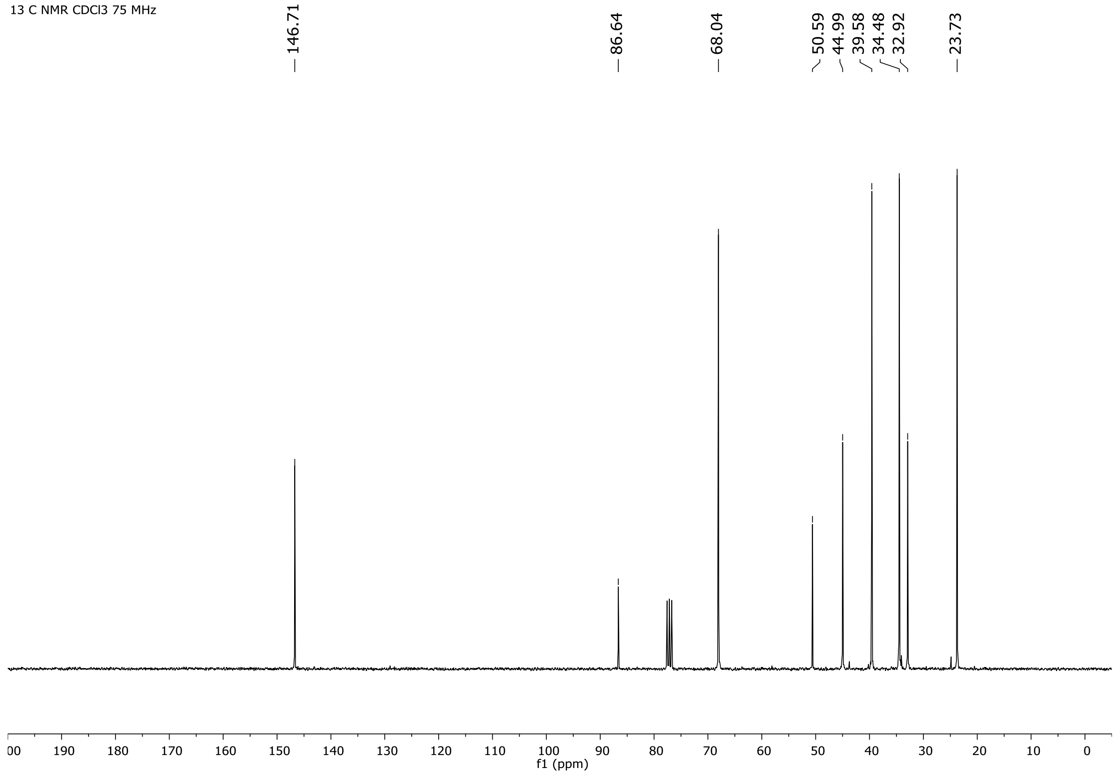

4-((1-ethynylcyclopentyl)methyl)tetrahydro-2H-pyran (16-CCH)

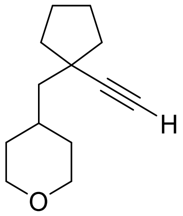

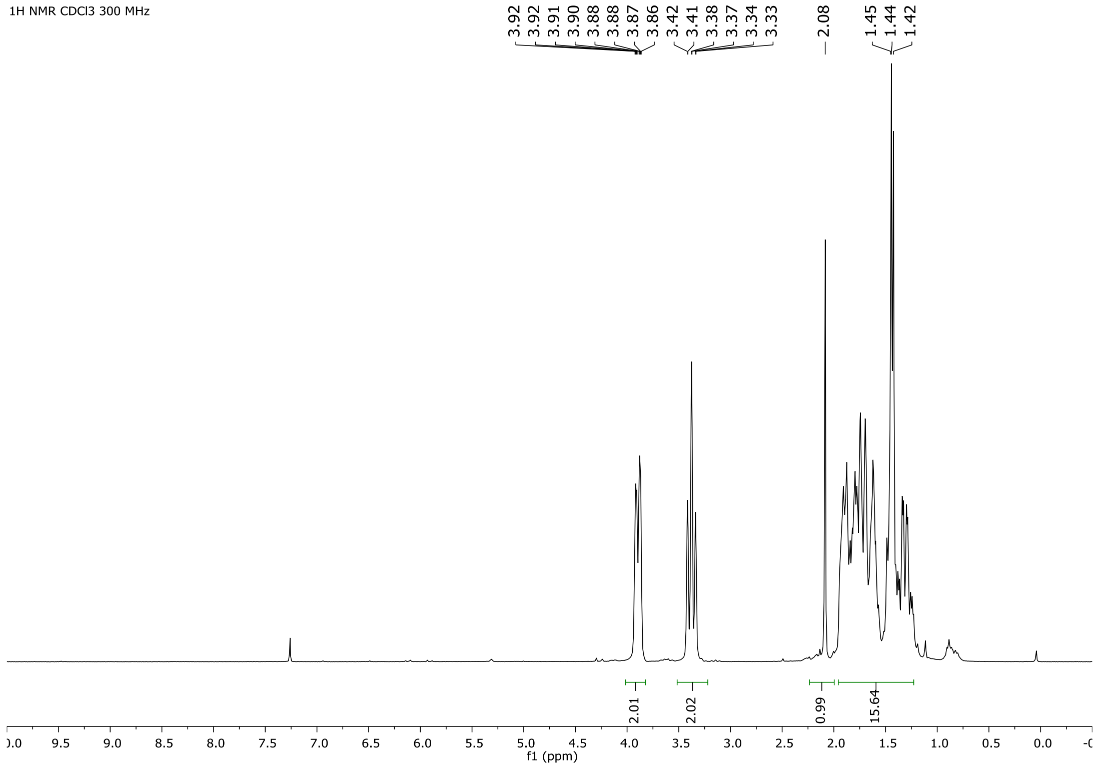

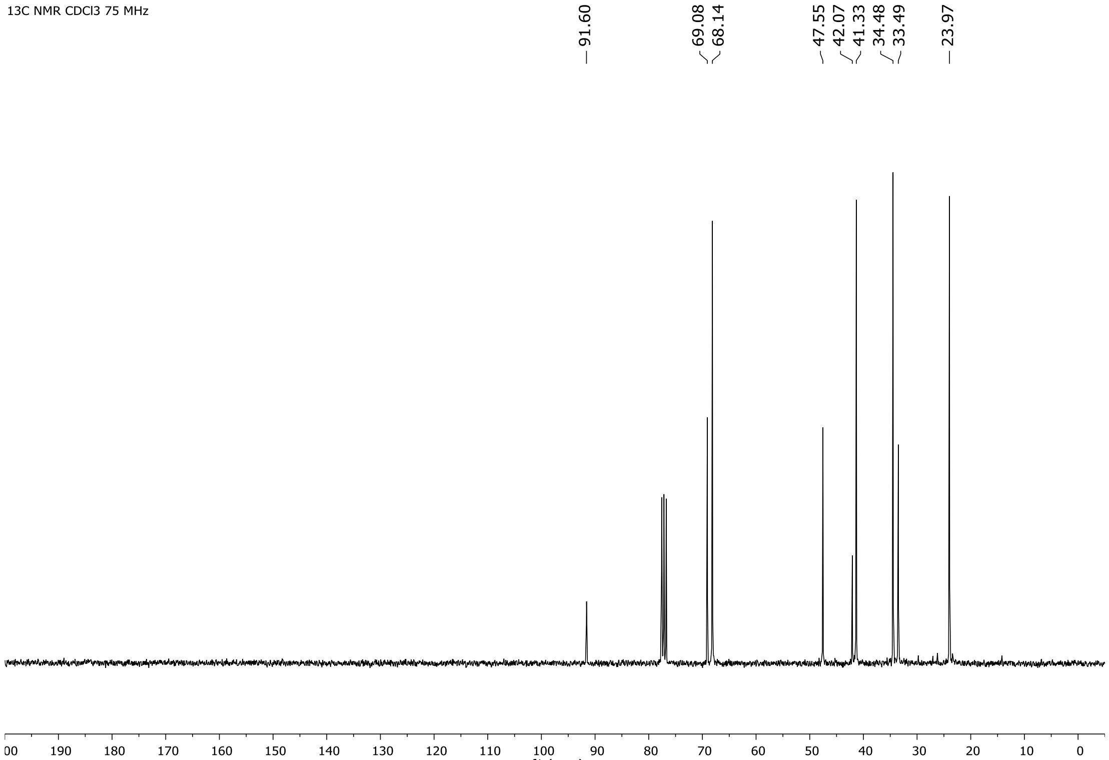

4-((1-(bromoethynyl)cyclopentyl)methyl)tetrahydro-2H-pyran (16)

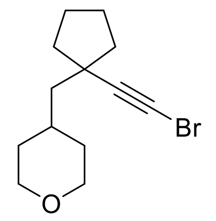

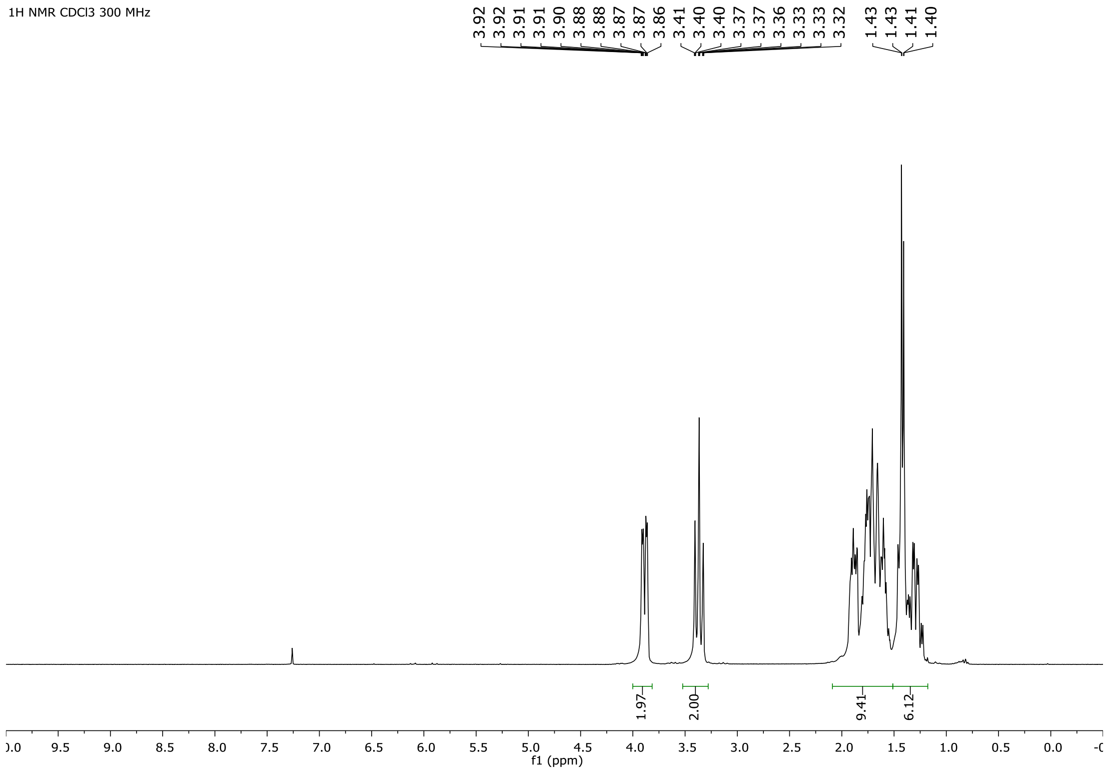

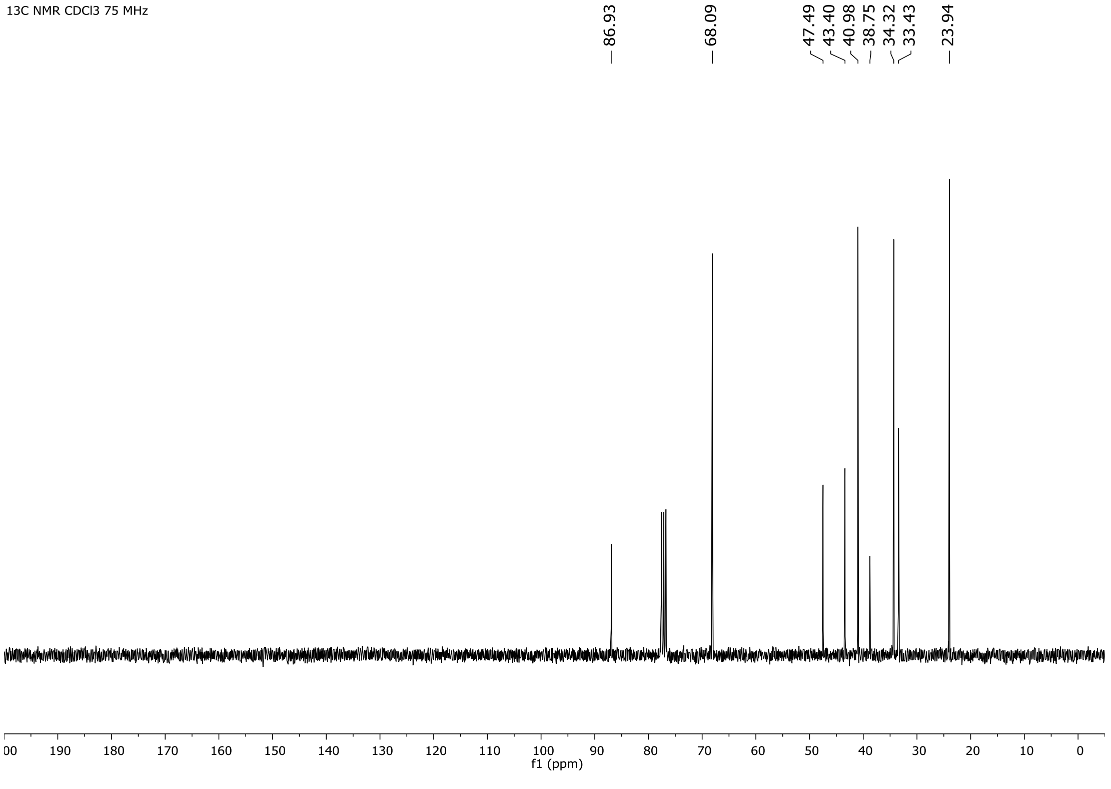

tert-butyl 3-(2-bromoethyl)azepane-1-carboxylate (17-Br)

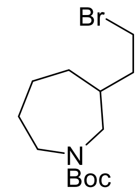

1H NMR CDCl3 300MHz

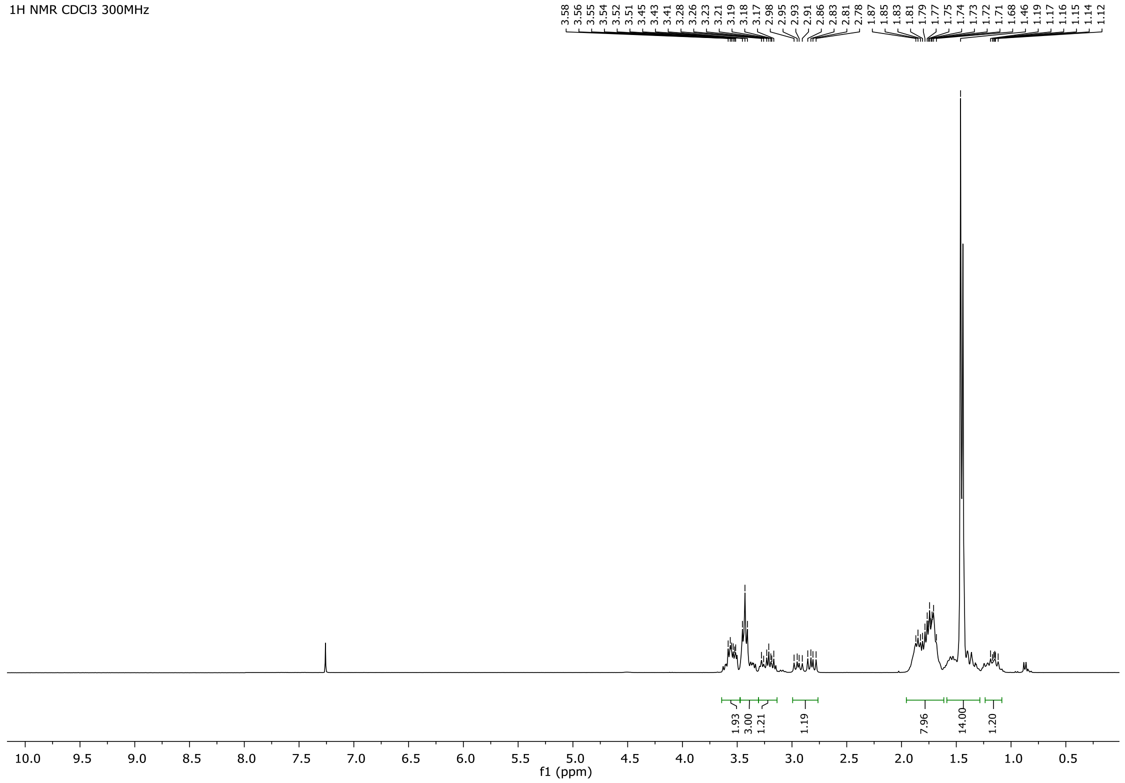

13C NMR CDCl3 75 MHz

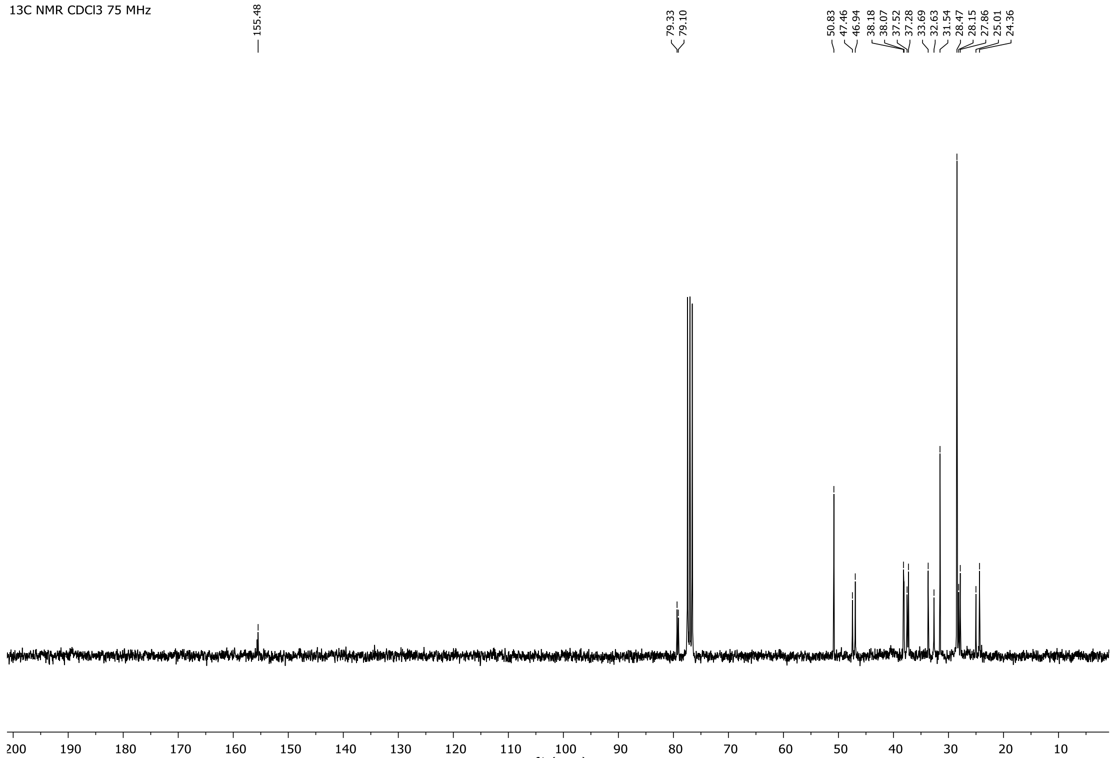

tert-butyl 3-(2-bromoethyl)azepane-1-carboxylate (17-CCH)

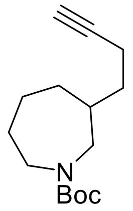

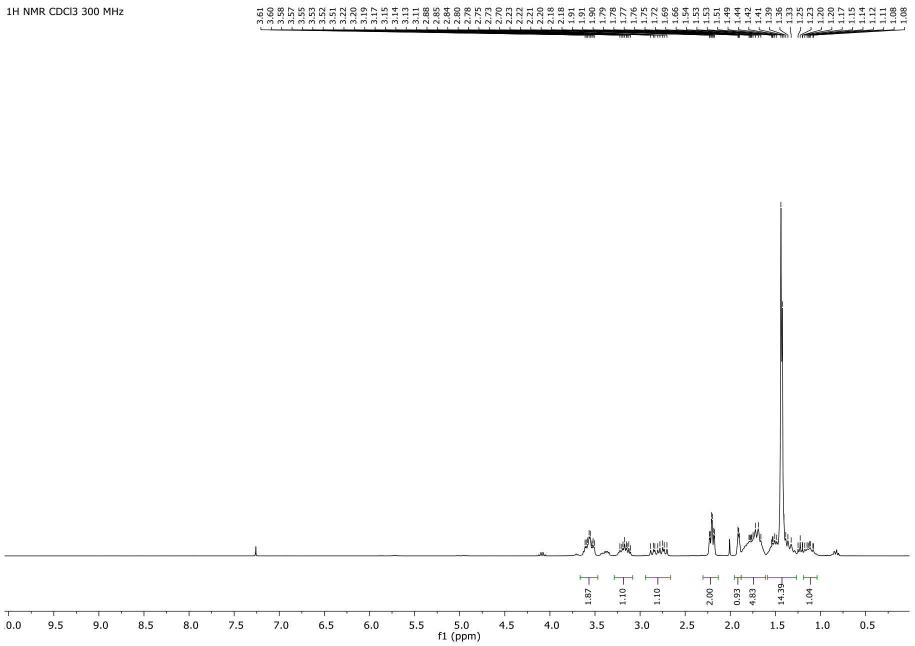

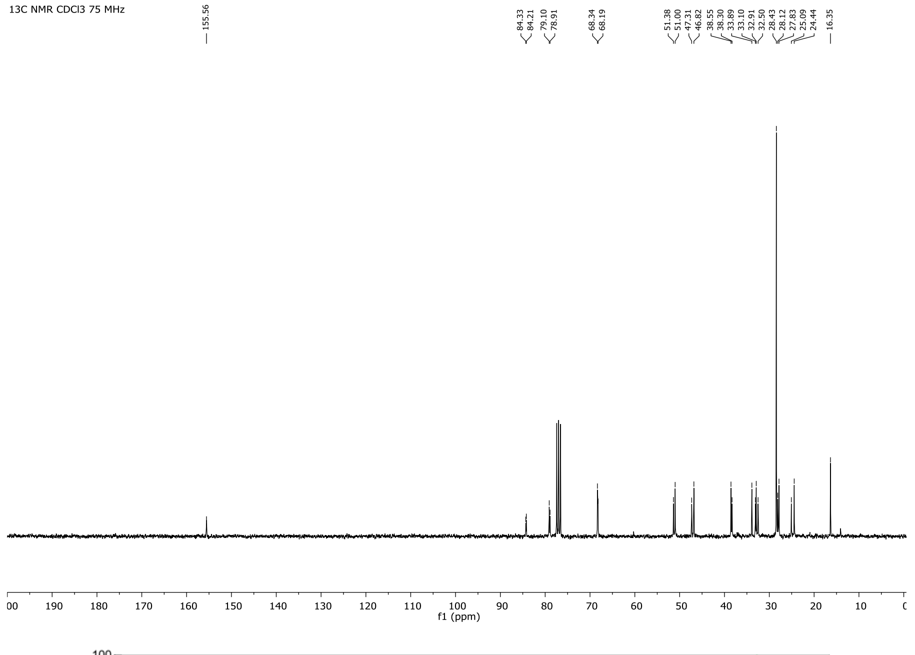

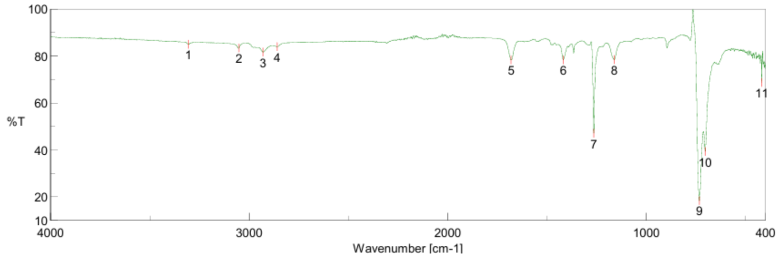

tert-butyl 3-(4-bromobut-3-yn-1-yl)azepane-1-carboxylate (17-NBoc)

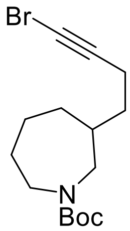

1h NMR CDCl3 300 MHz

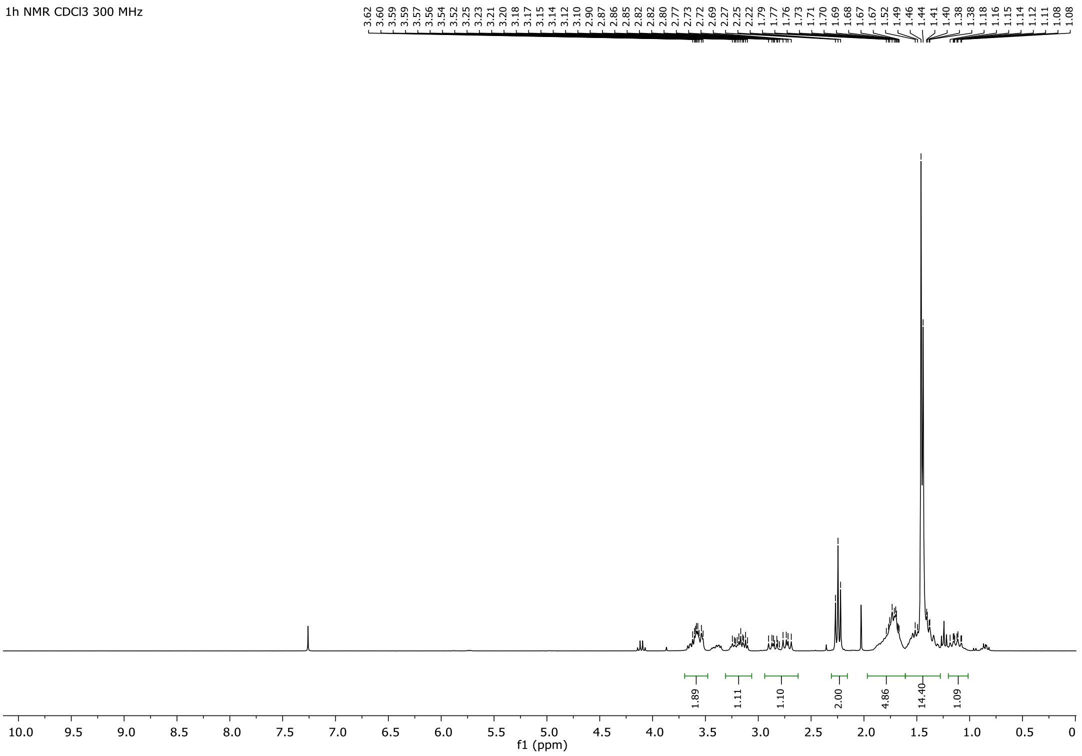

13C NMR CDCl3 75 MHz

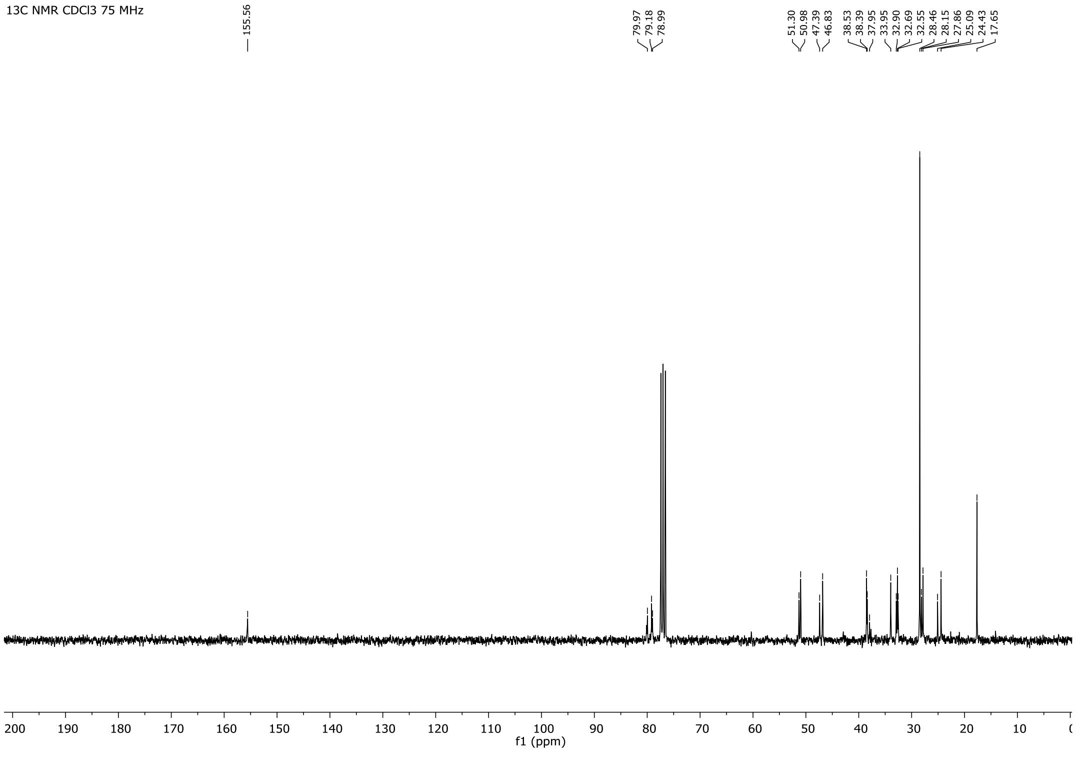

1-(3-(4-bromobut-3-yn-1-yl)azepan-1-yl)-2,2,2-trifluoroethan-1-one (17)

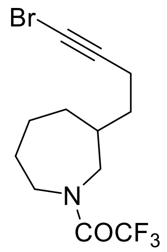

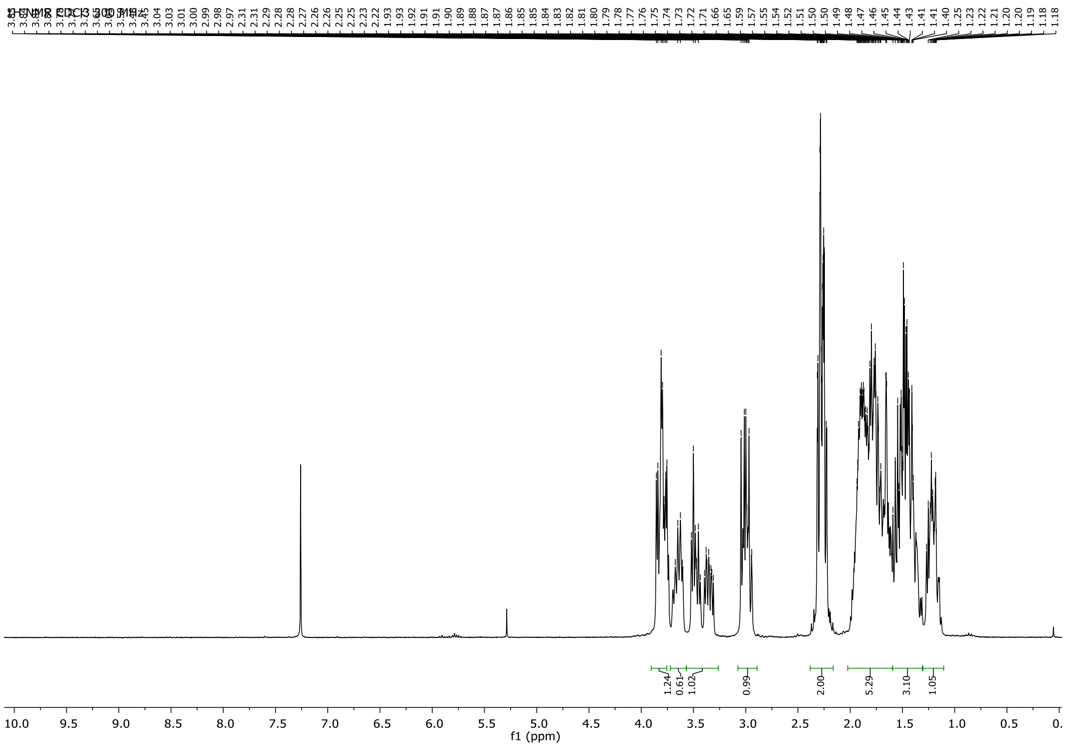

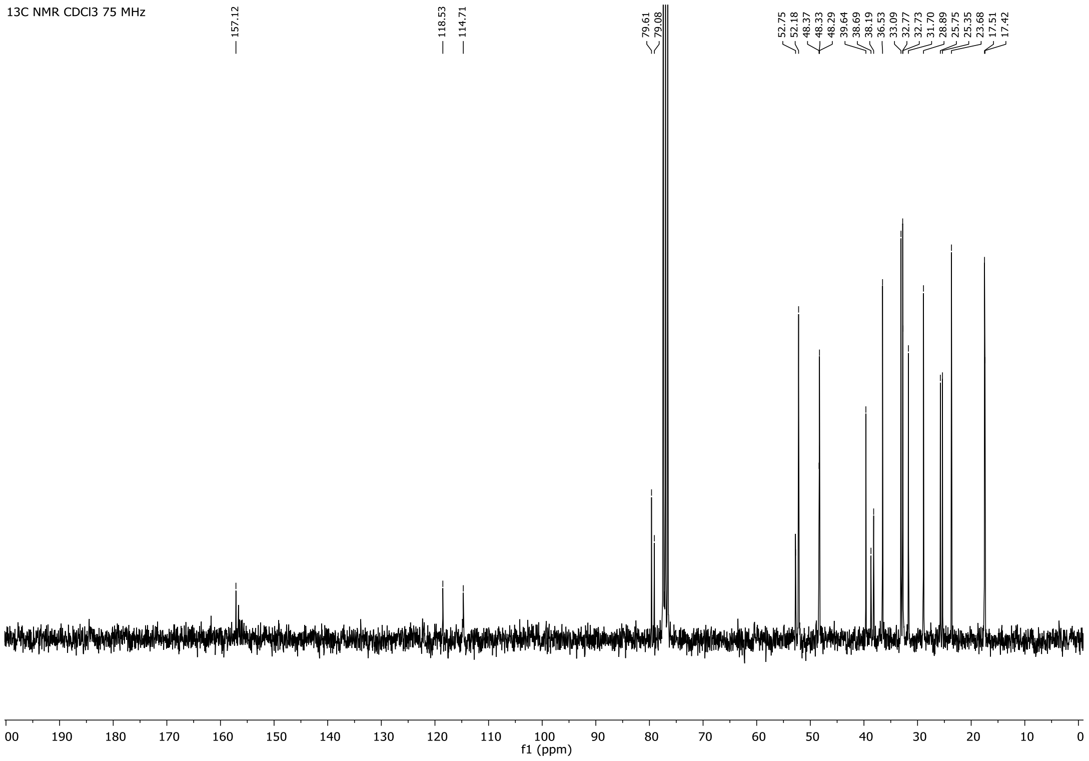

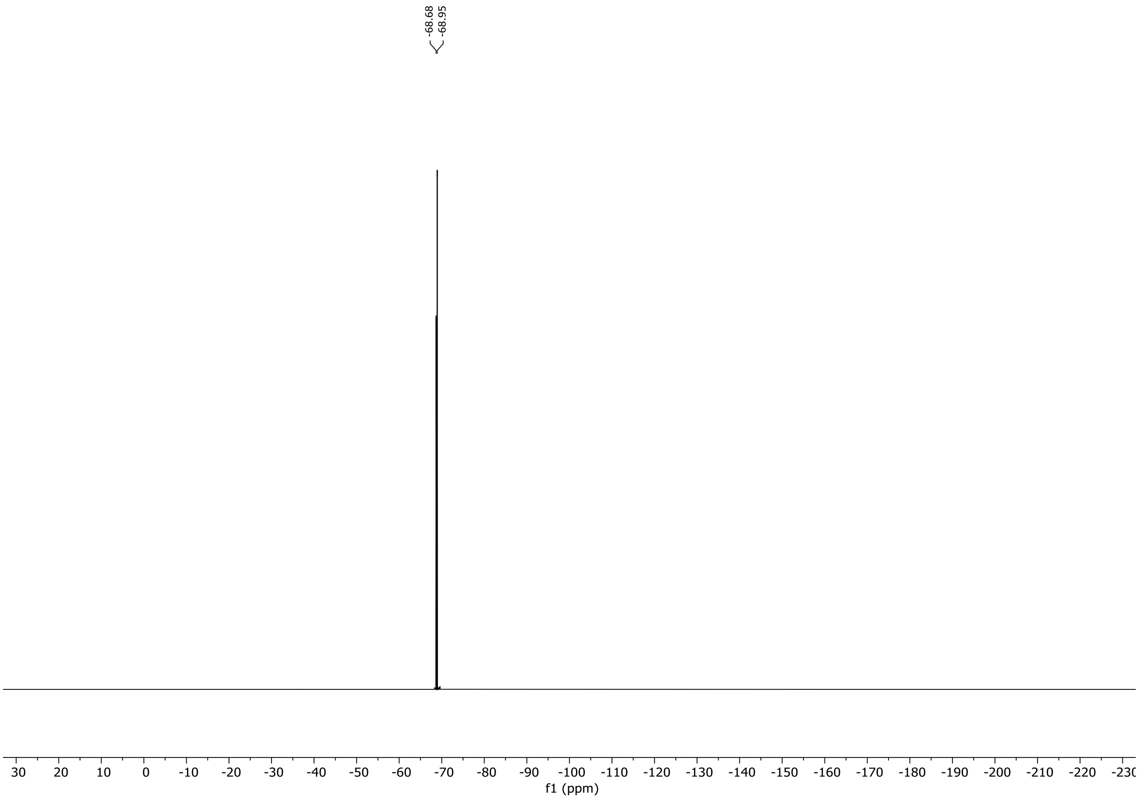

-68.68
-68.95
30 20 10 0 -10 -20 -30 -40 -50 -60 -70 -80 -90 -100 -110 -120 -130 -140 -150 -160 -170 -180 -190 -200 -210 -220 -230
f1 (ppm)

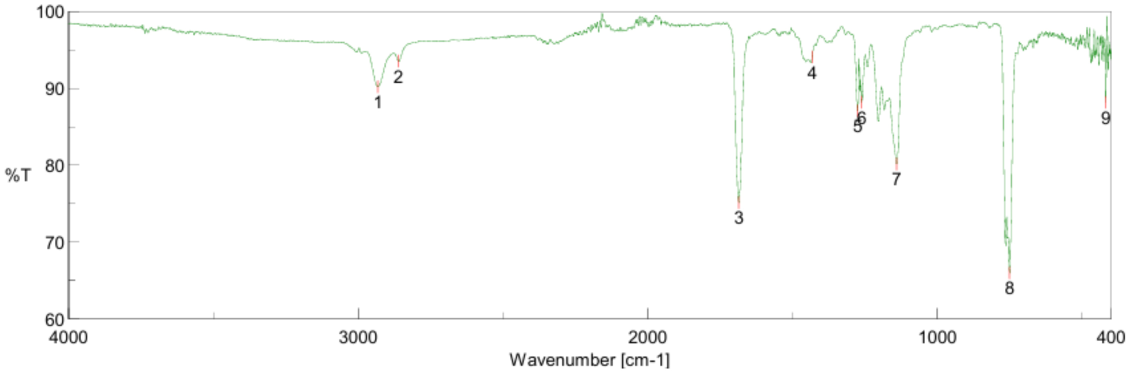

100
90
80
70
60
%T
1
2
3
4
5
6
7
8
9
4000
3000
2000
1000
400
Wavenumber [cm-1]

2-(1,4-dioxaspiro[4.5]decan-7-yl)acetic acid (18-CO_2H)

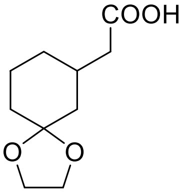

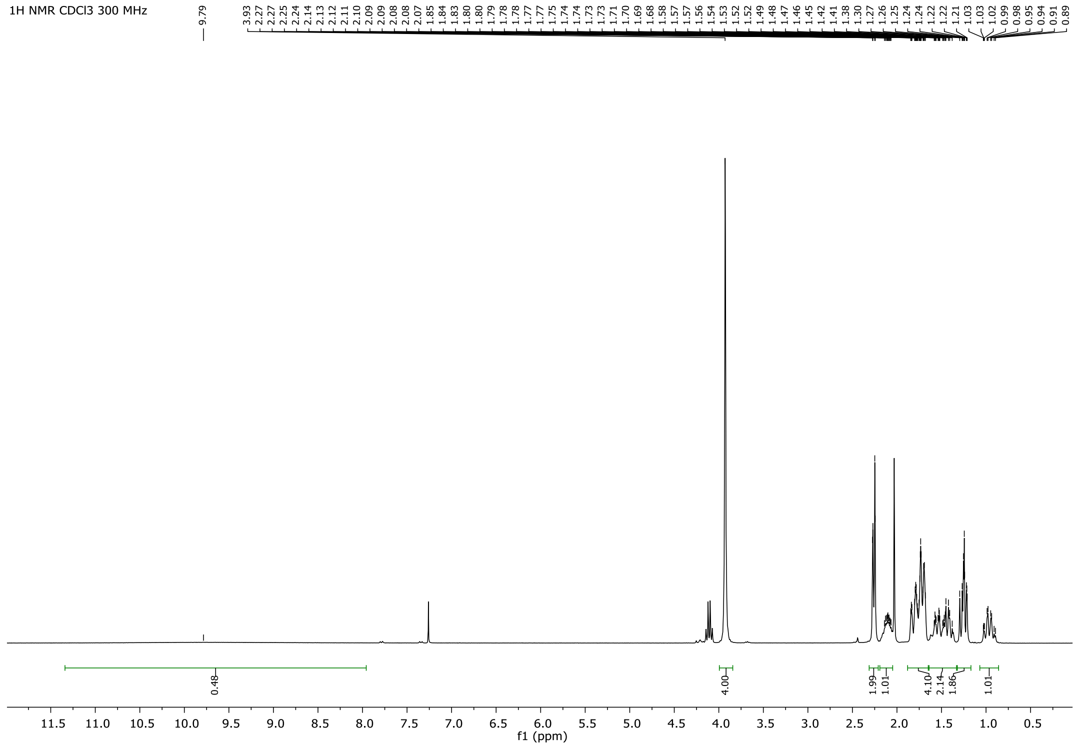

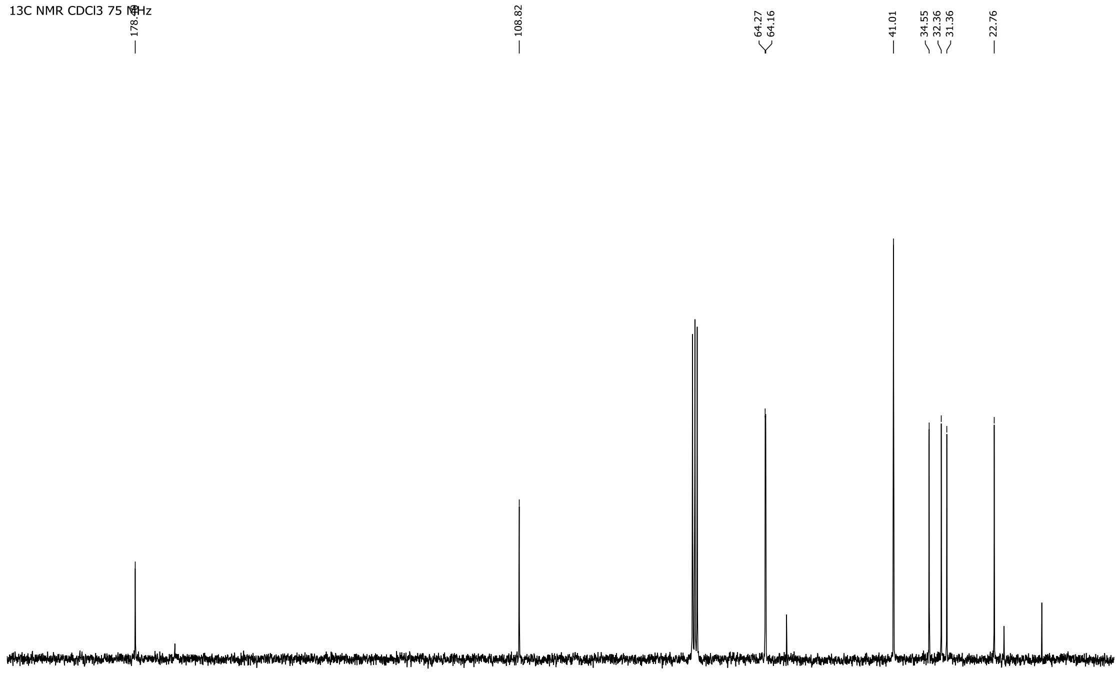

2-(1,4-dioxaspiro[4.5]decan-7-yl)ethan-1-ol (18-OH)

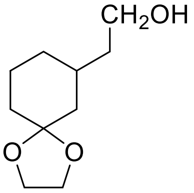

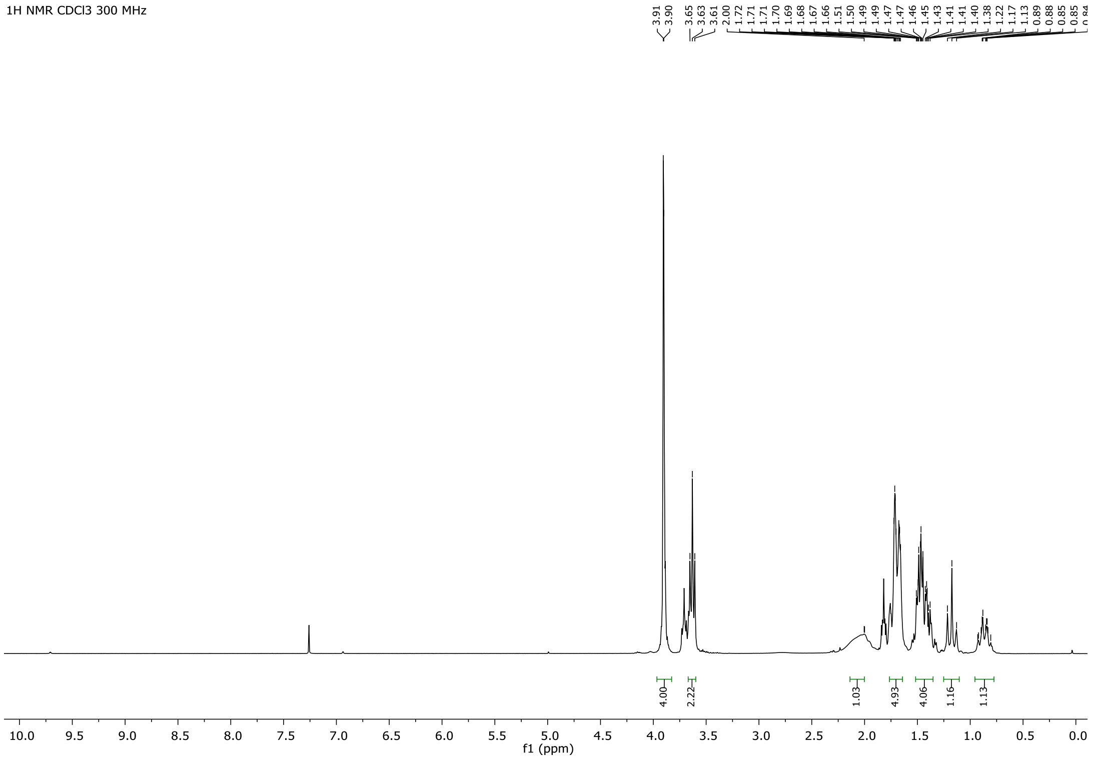

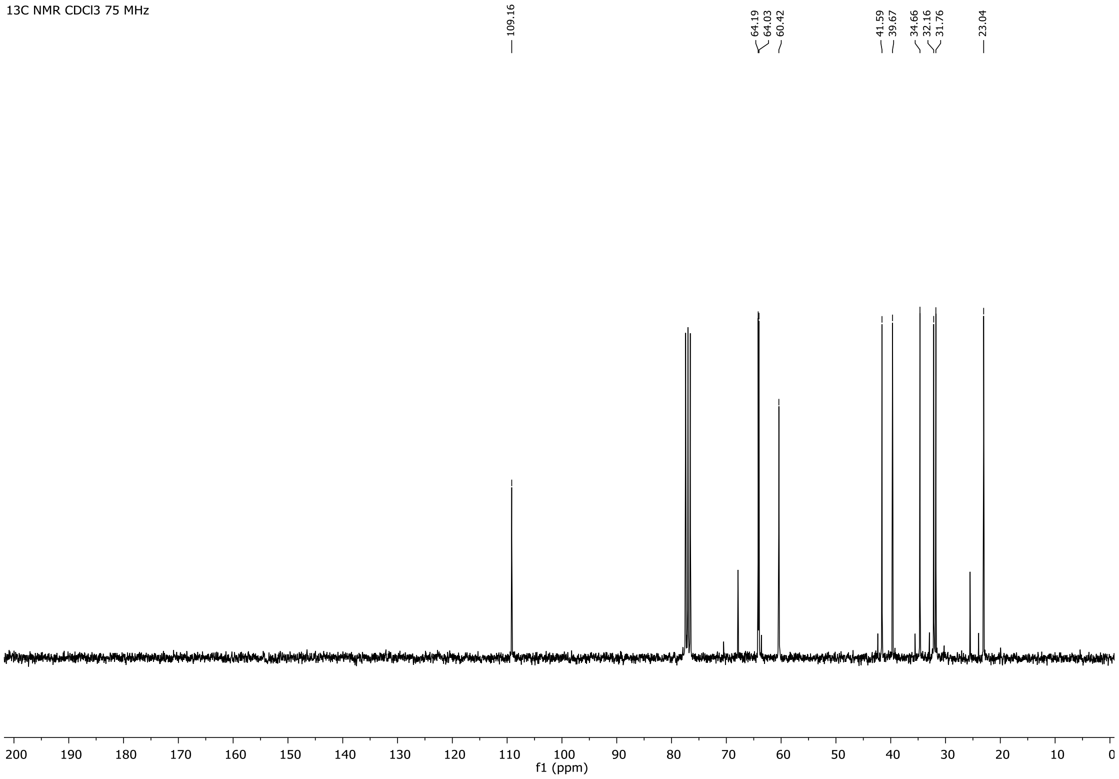

7-(2-bromoethyl)-1,4-dioxaspiro[4.5]decane (18-Br)

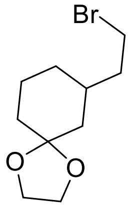

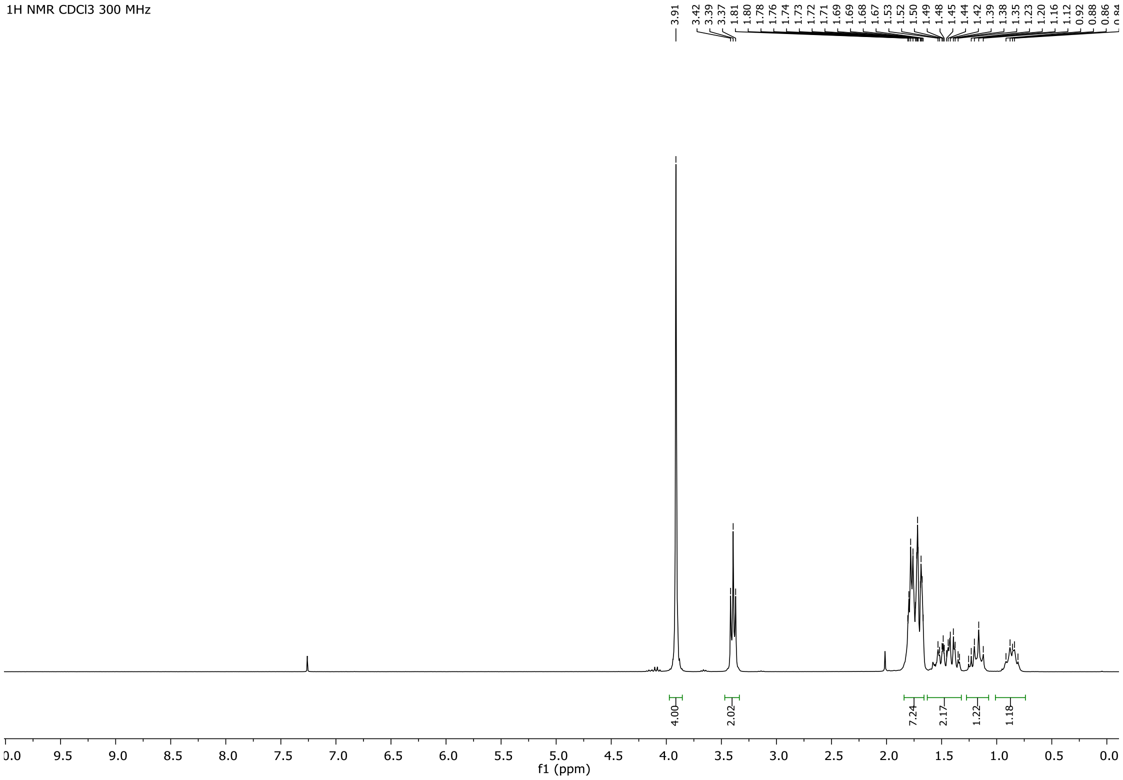

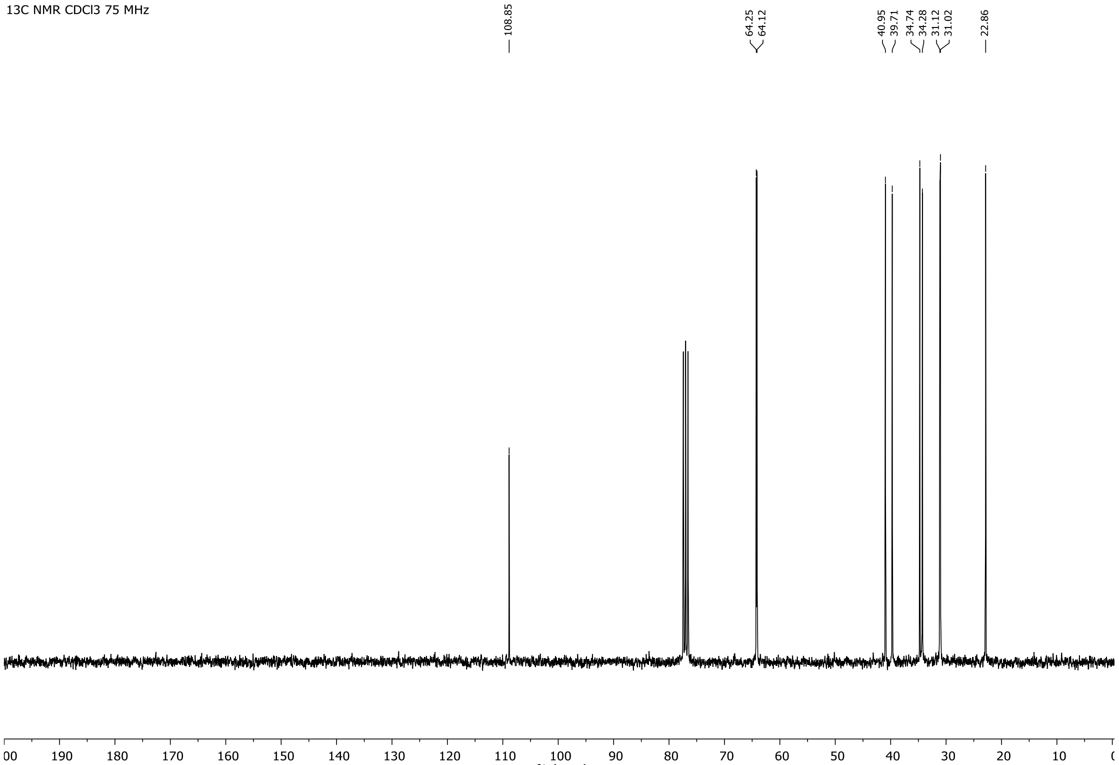

7-(but-3-yn-1-yl)-1,4-dioxaspiro[4.5]decane (18-CCH)

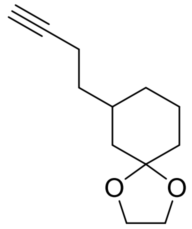

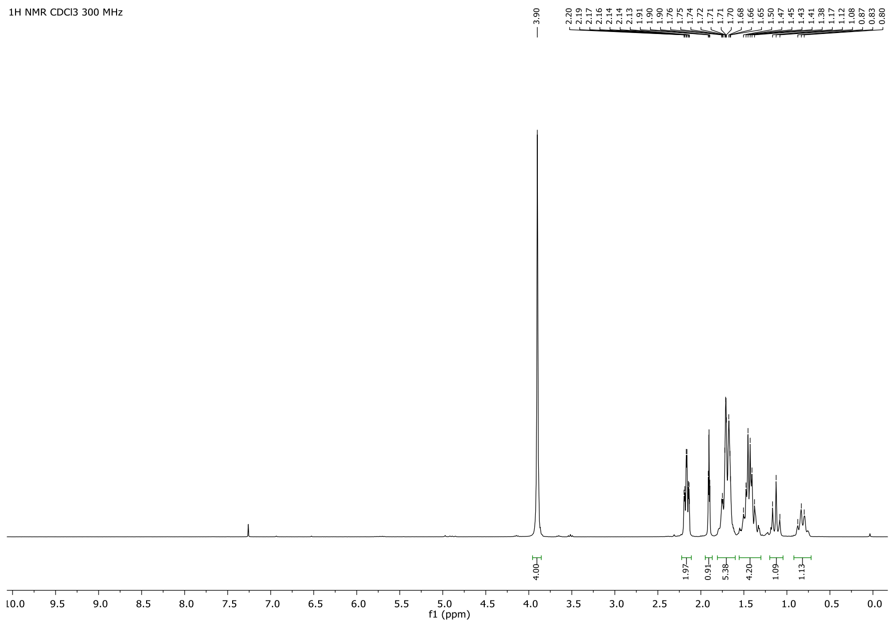

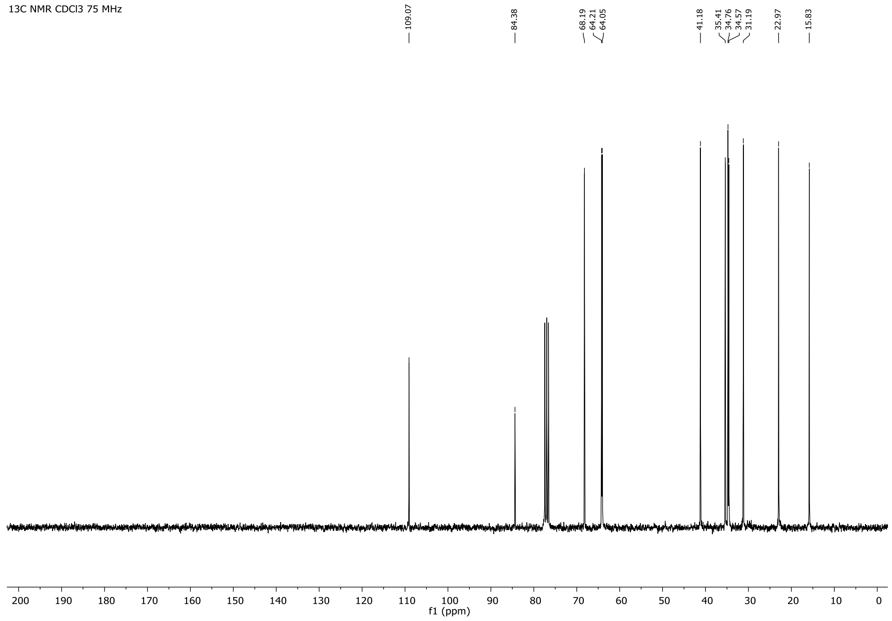

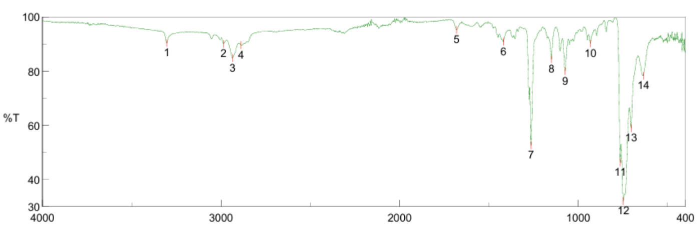

7-(4-bromobut-3-yn-1-yl)-1,4-dioxaspiro[4.5]decane (18-acetal)

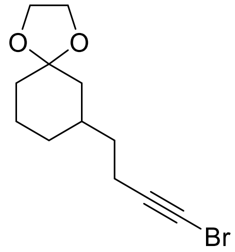

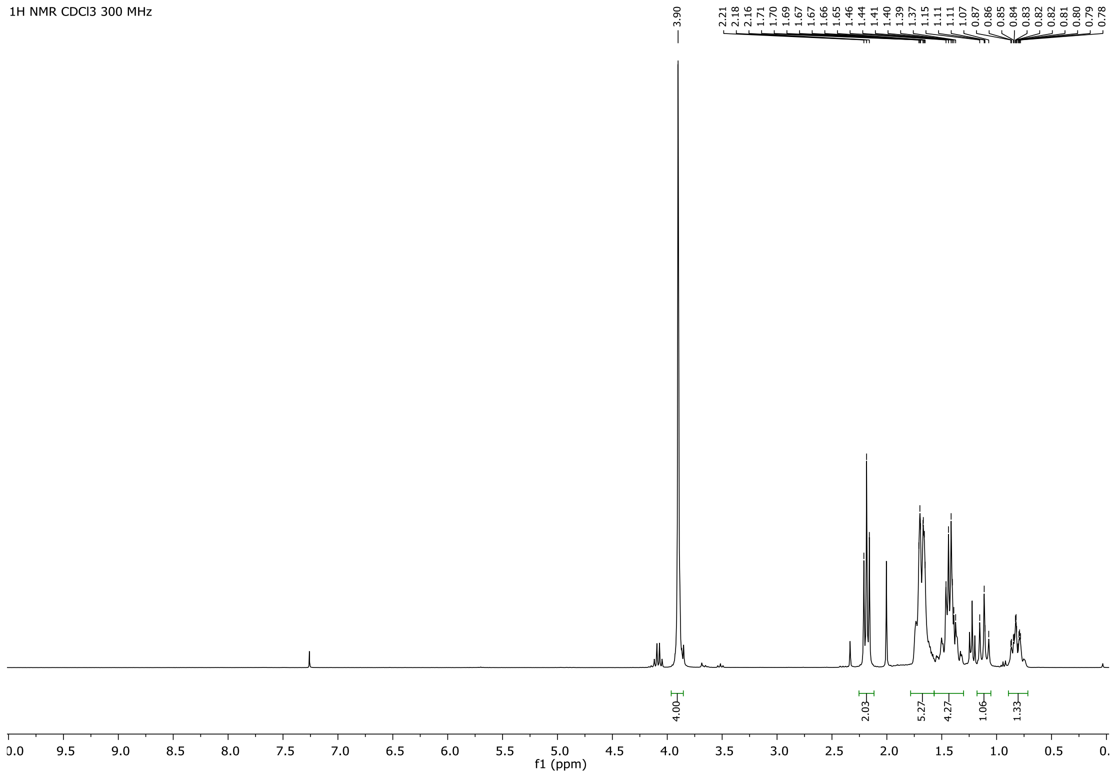

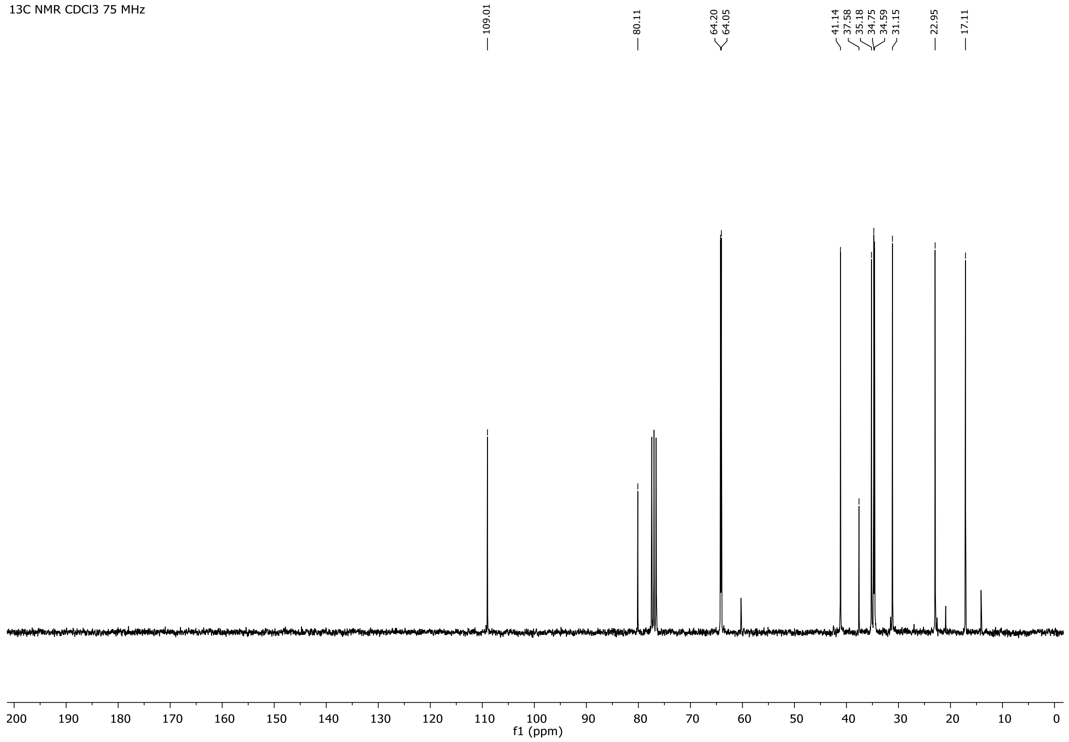

3-(4-bromobut-3-yn-1-yl)cyclohexan-1-one (18)

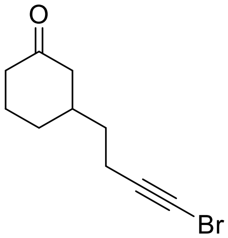

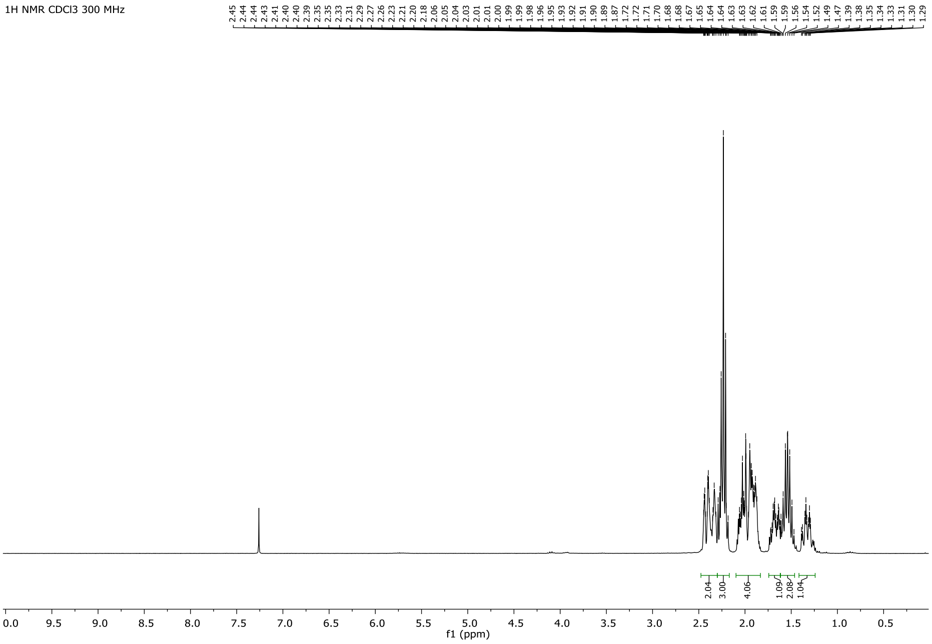

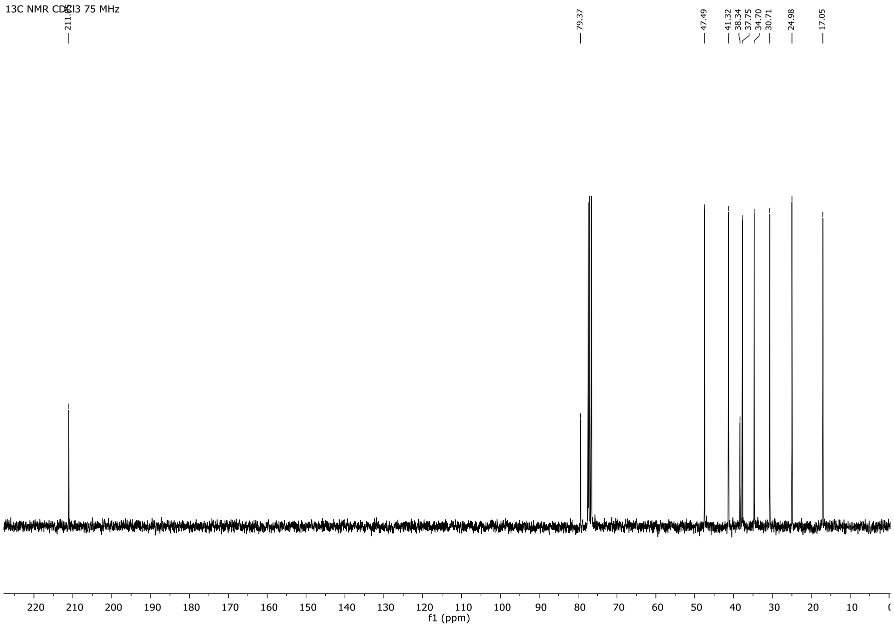

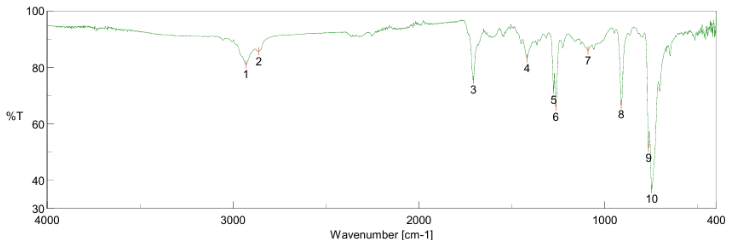

3-(4-bromobut-3-yn-1-yl)-1,1-difluorocyclohexane (19)

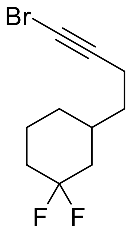

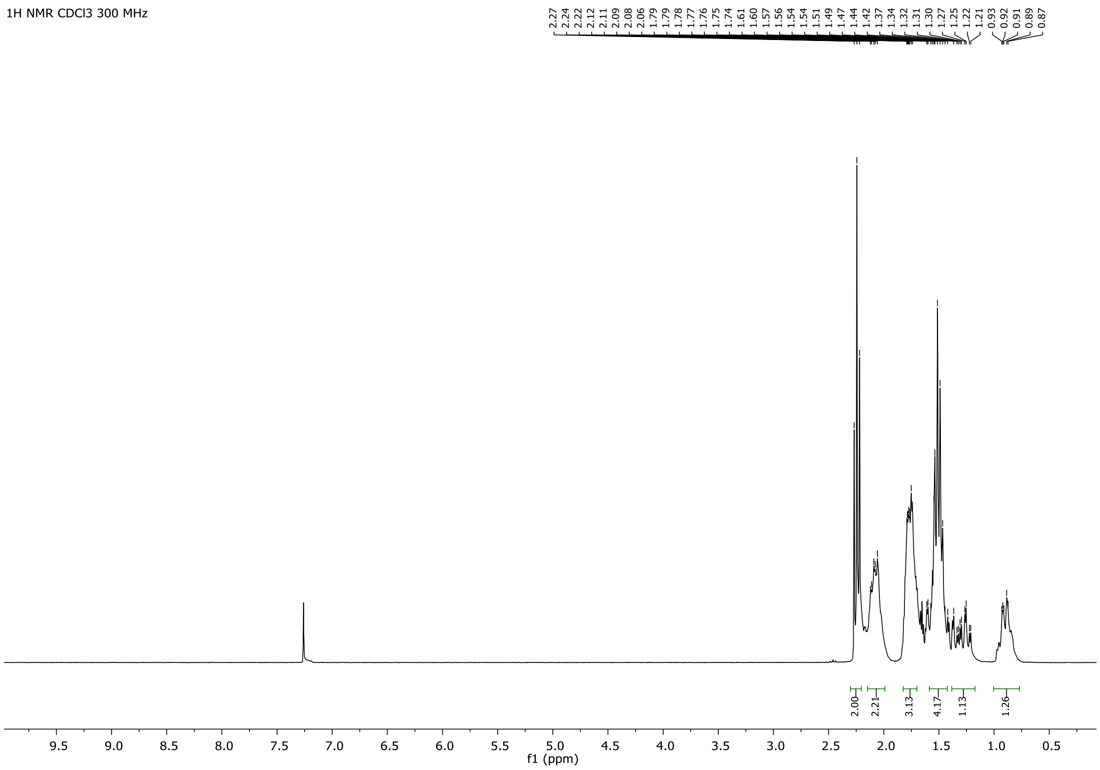

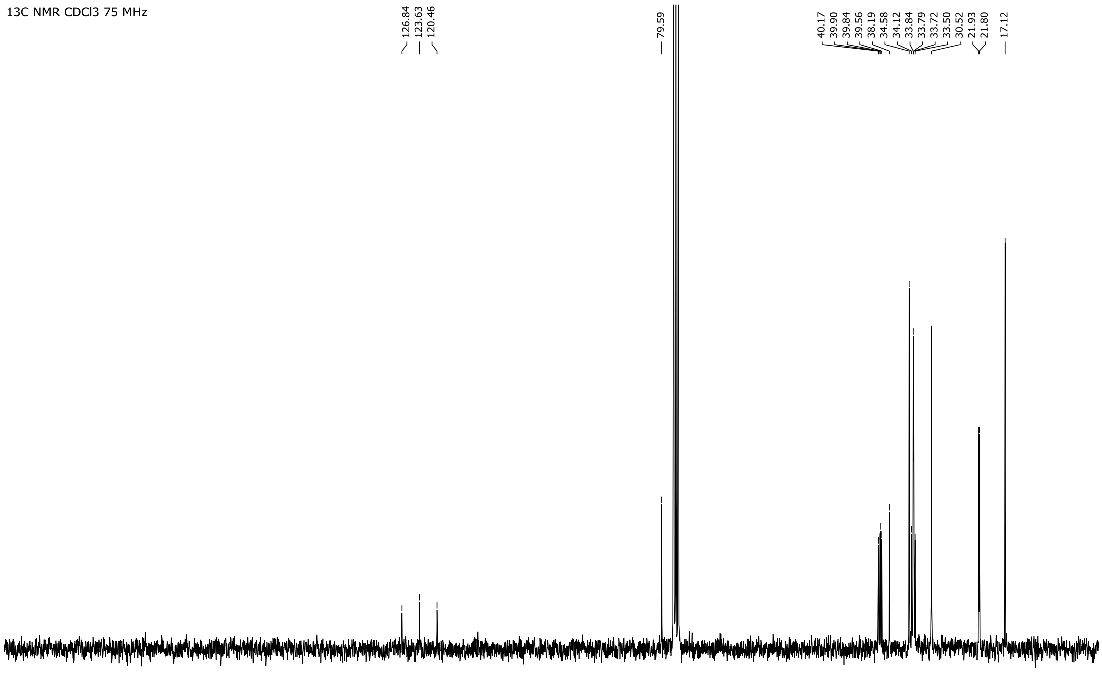

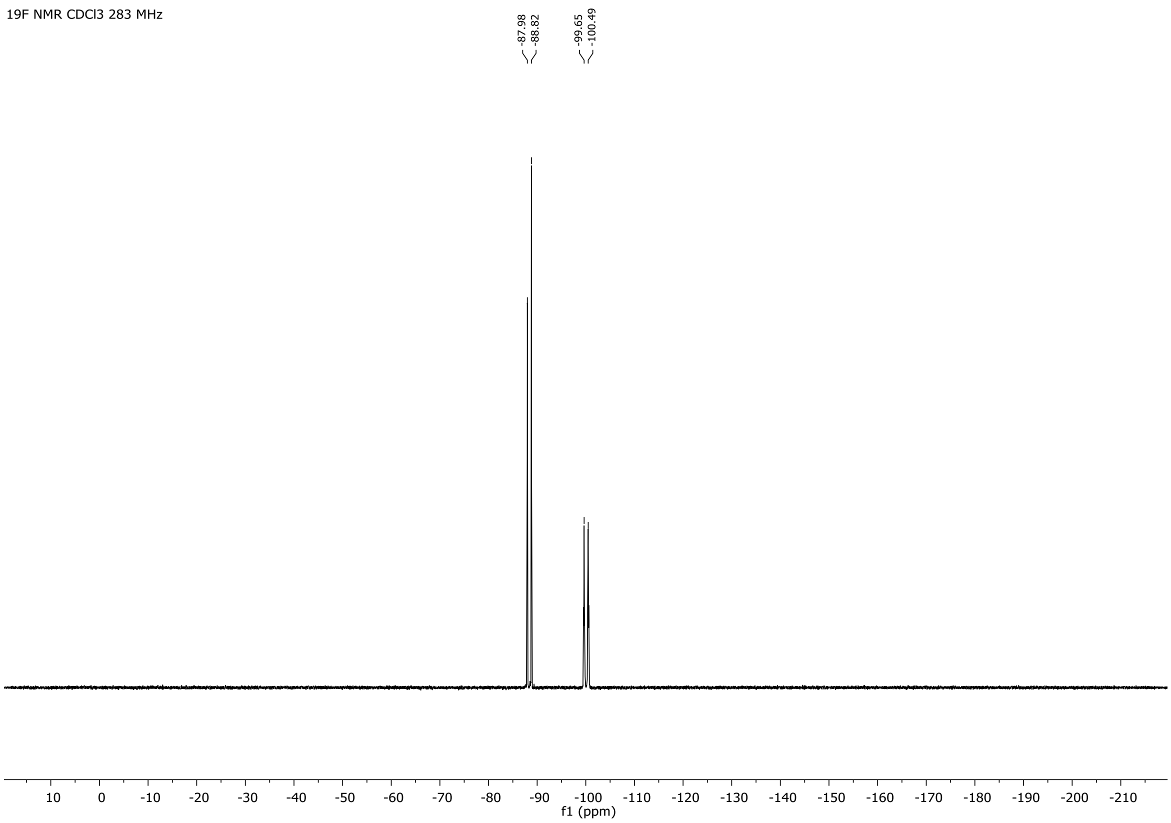

19F NMR CDCl3 283 MHz
-87.98
-88.82
-99.65
-100.49
10
0
-10
-20
-30
-40
-50
-60
-70
-80
-90
-100
-110
-120
-130
-140
-150
-160
-170
-180
-190
-200
-210
f1 (ppm)

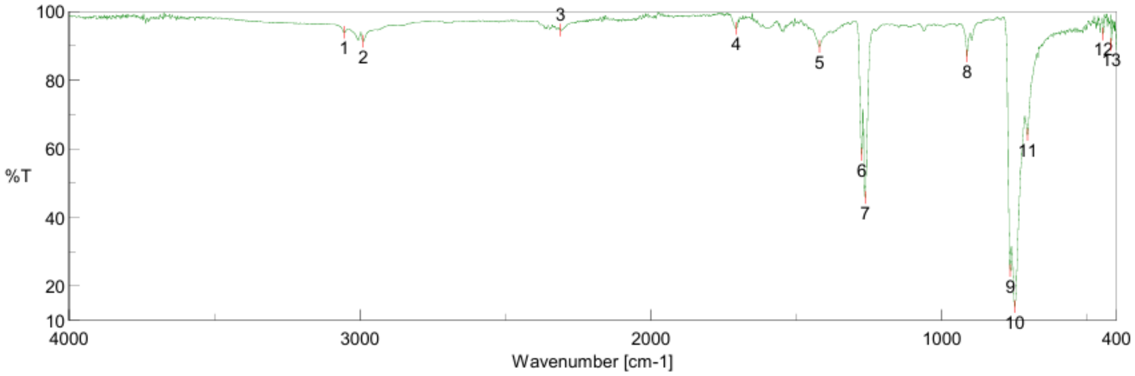

%T
100
80
60
40
20
10
4000
3000
2000
1000
400
Wavenumber [cm-1]
1
2
3
4
5
6
7
8
9
10
11
12
13

tert-butyl 4-(4-bromobut-3-yn-1-yl)piperidine-1-carboxylate (20)

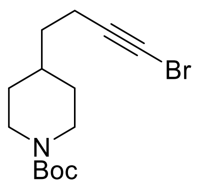

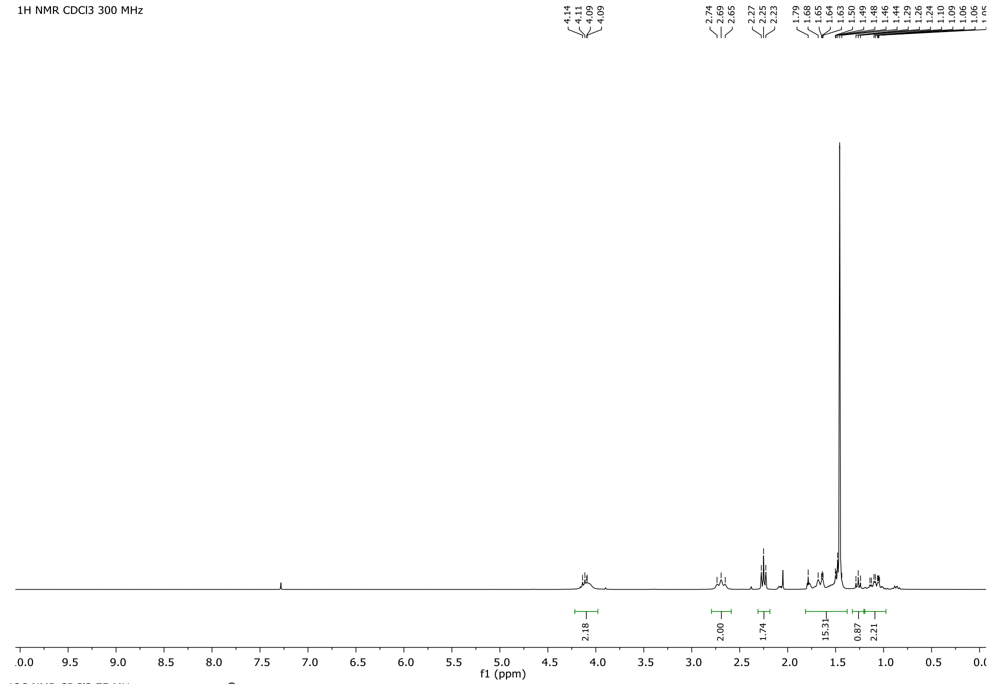

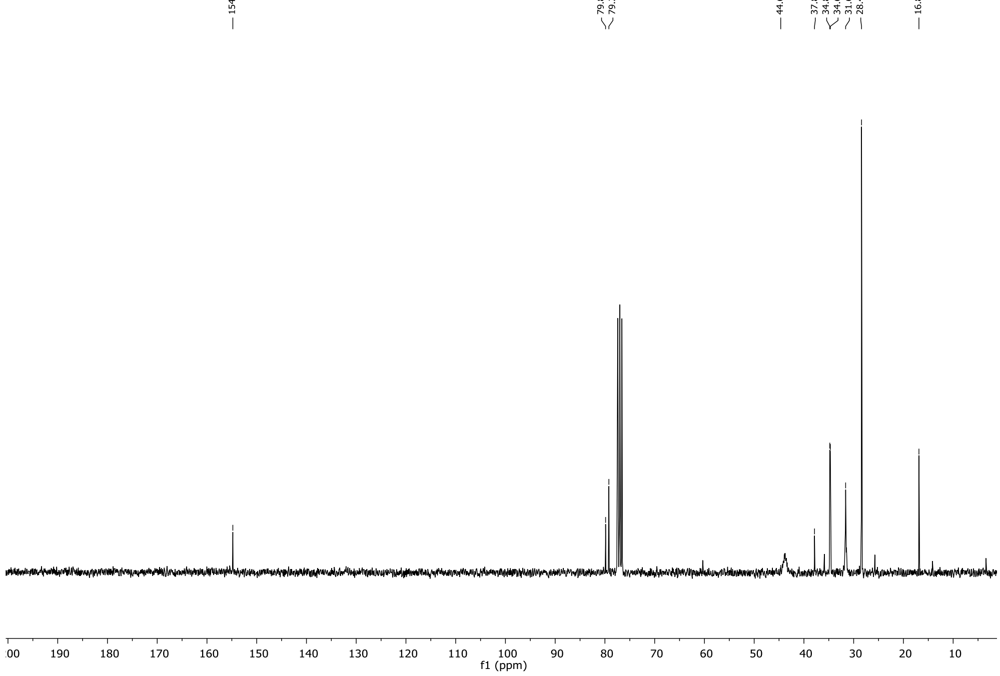

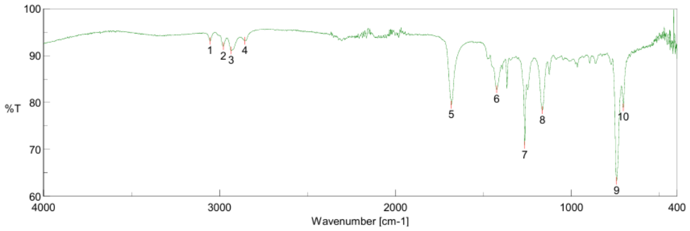

GOLD(I)-CATALYZED REACTIONS

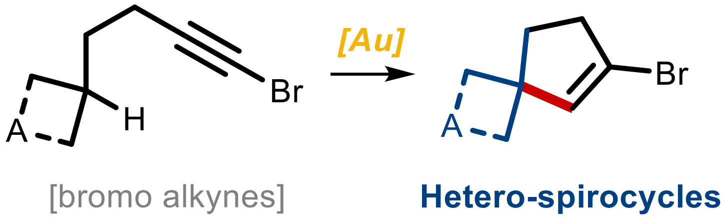

2-bromo-7-oxaspiro[4.5]dec-1-ene (3a)

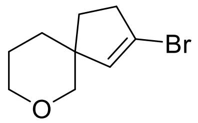

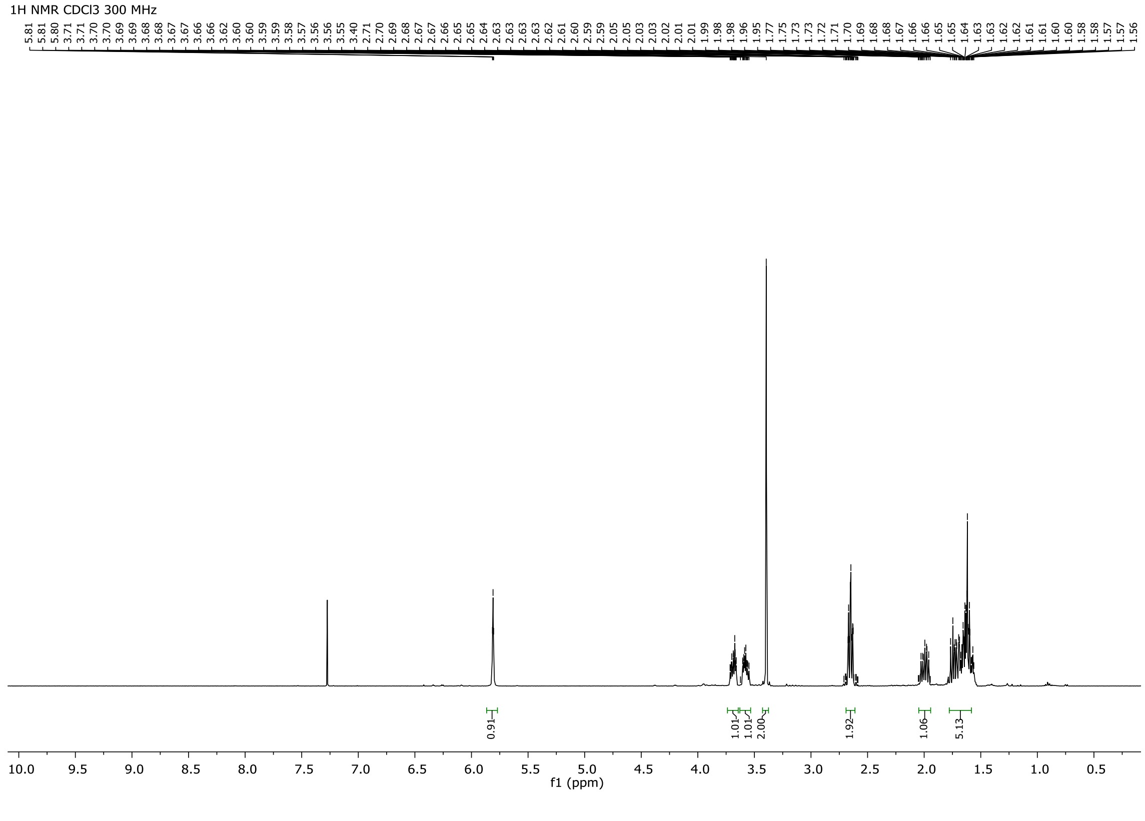

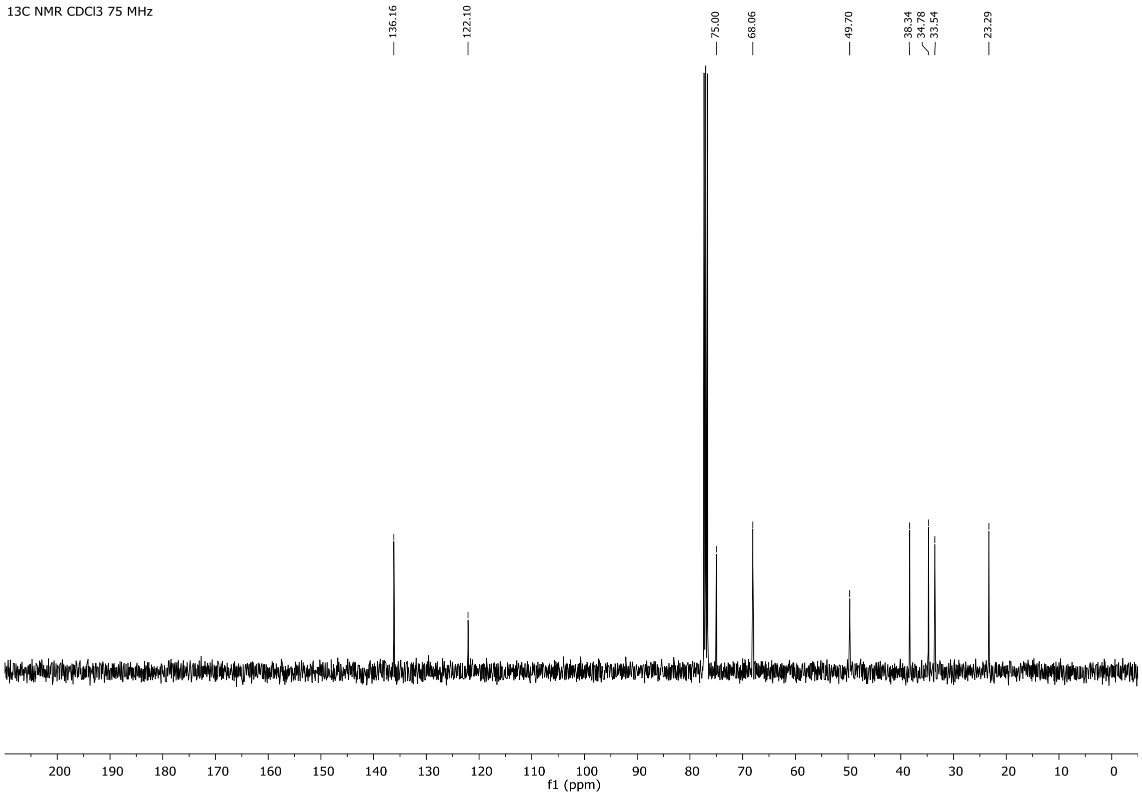

2-bromo-8-oxaspiro[4.5]dec-1-ene (4a)

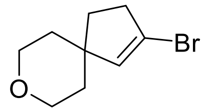

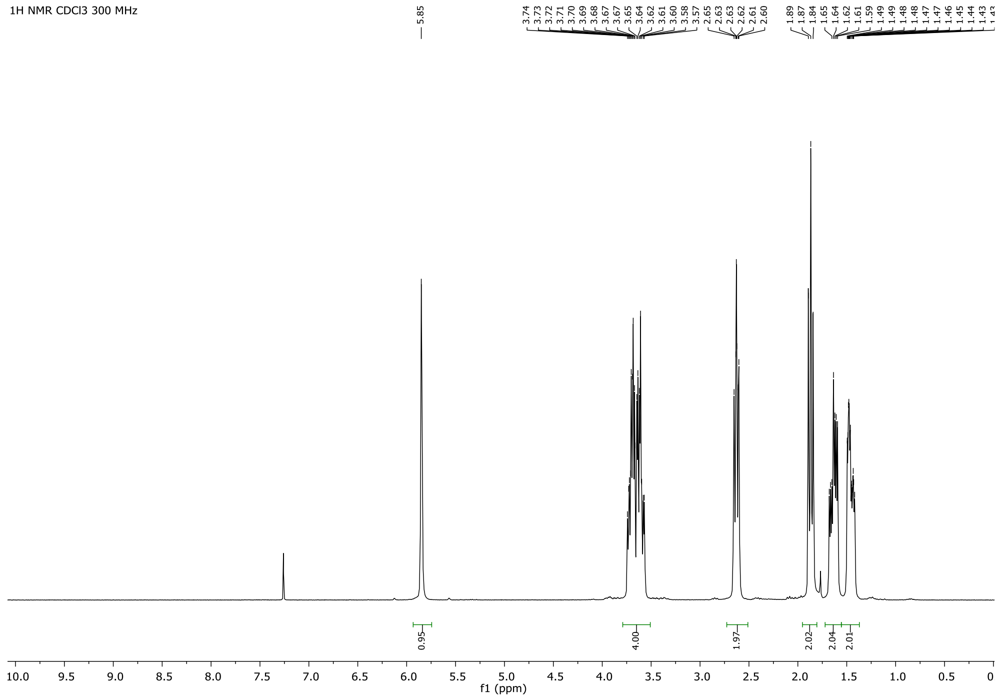

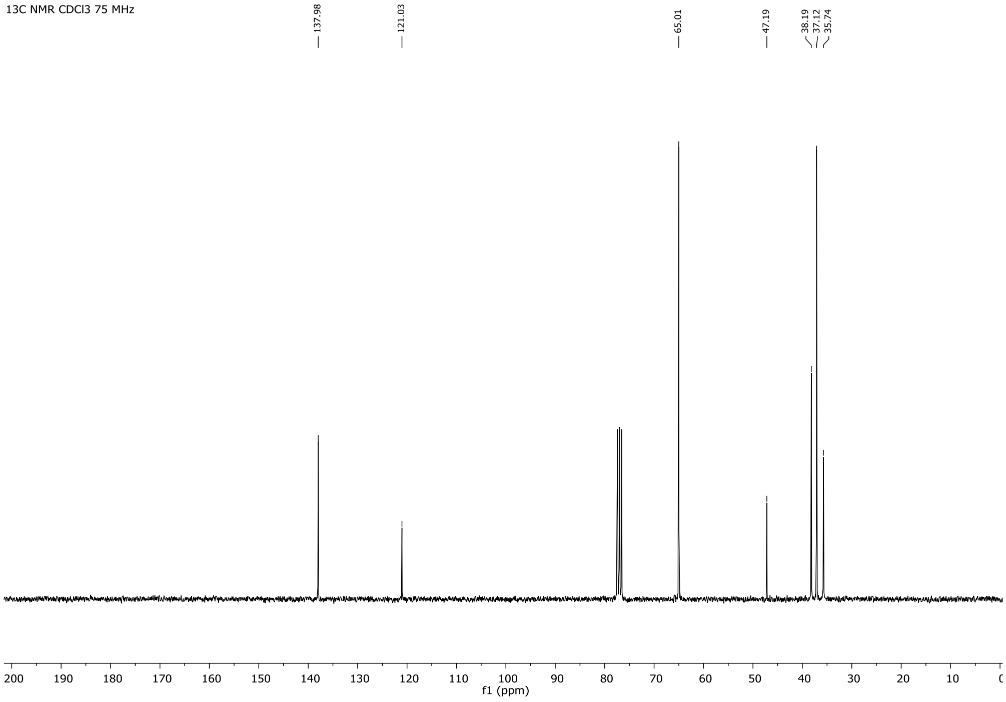

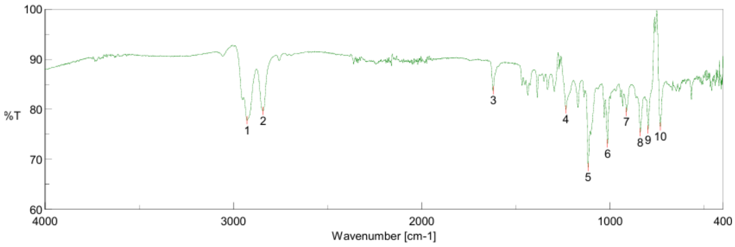

10-bromo-1,4-dioxadispiro[4.2.4^{8}.2^{5}]tetradec-9-ene (5a)

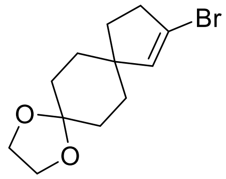

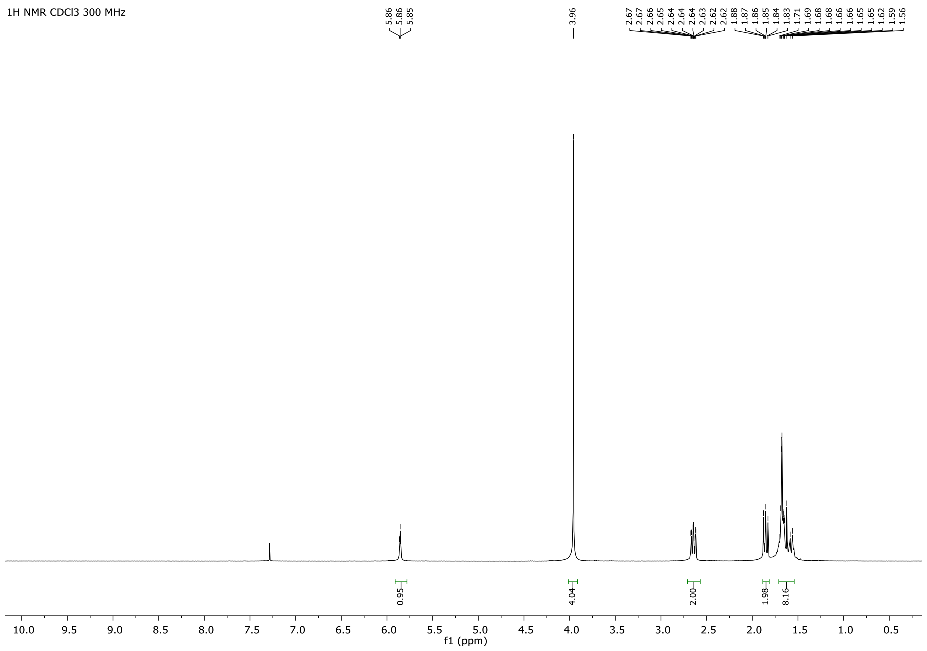

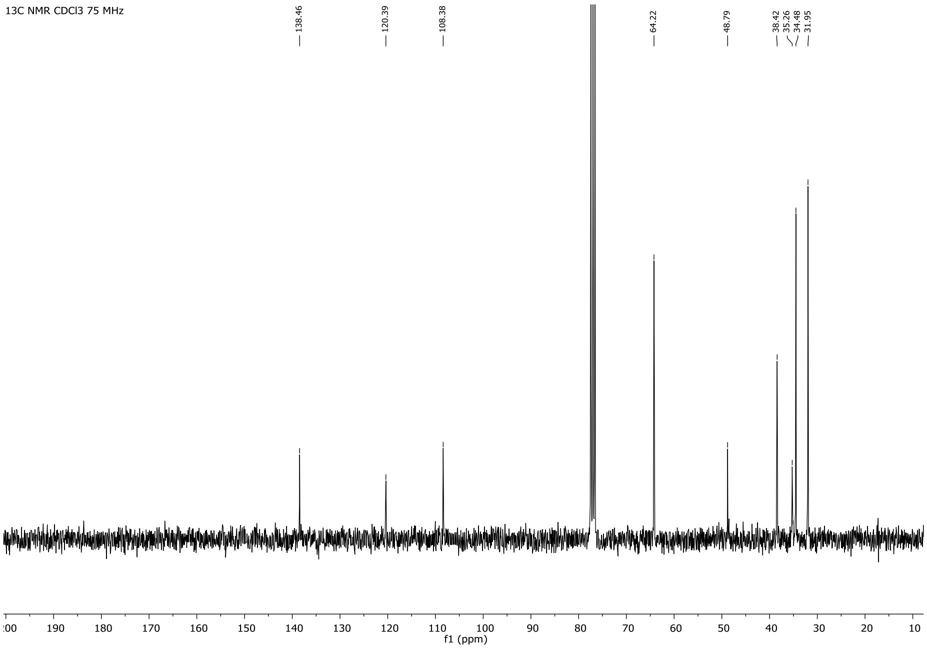

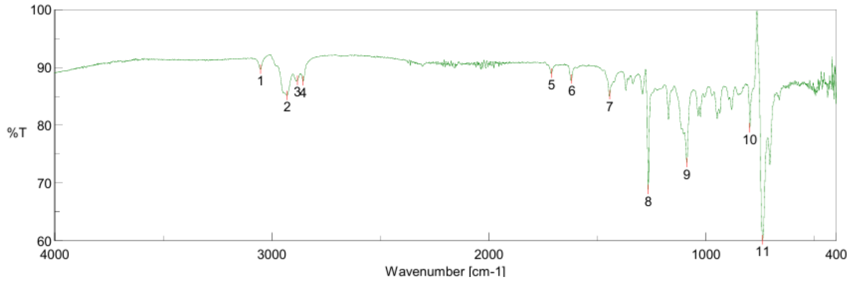

2-bromospiro[4.5]dec-1-en-8-one (6a)

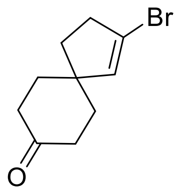

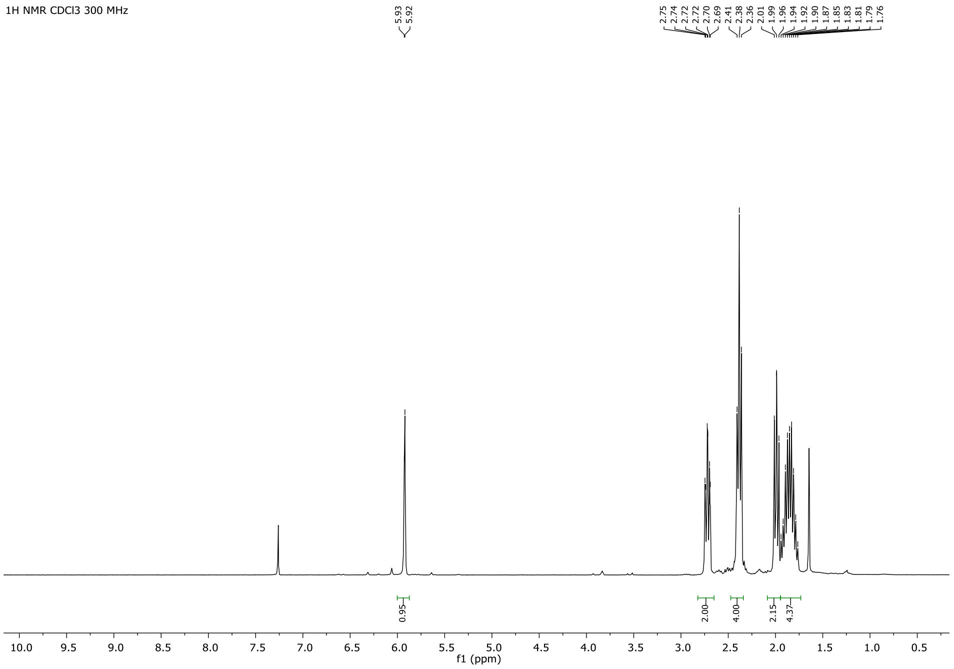

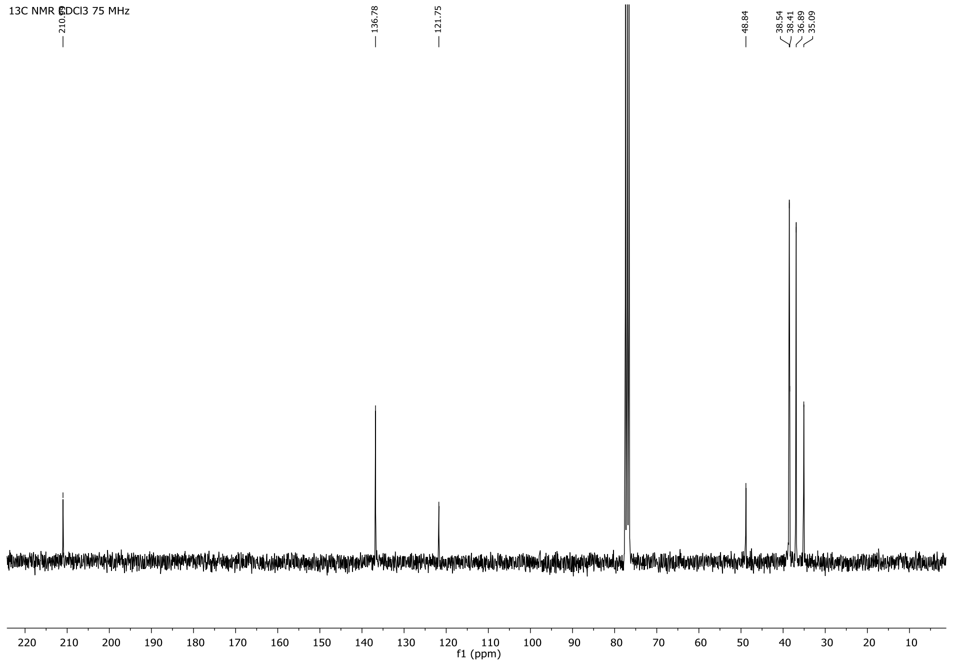

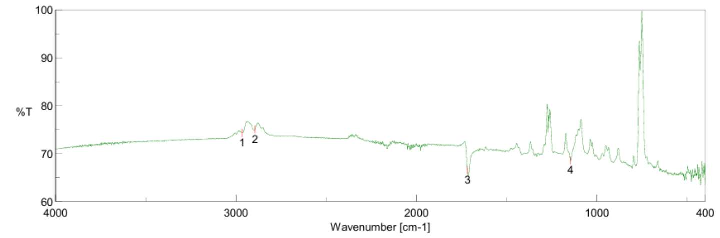

1-(2-bromo-8-azaspiro[4.5]dec-1-en-8-yl)-2,2,2-trifluoroethan-1-one (7a)

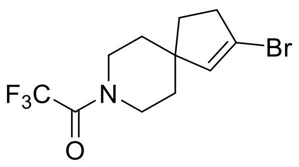

1H NMR CDCl3 300 MHz

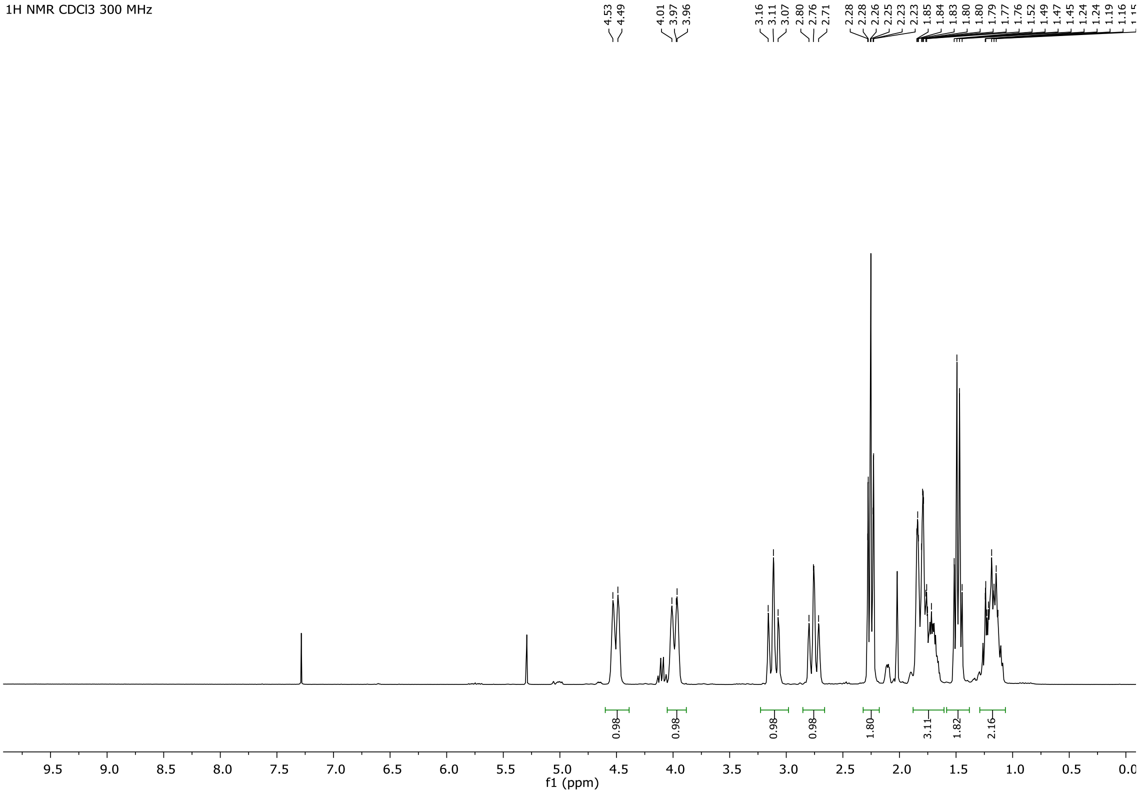

13C NMR CDCl3 75 MHz

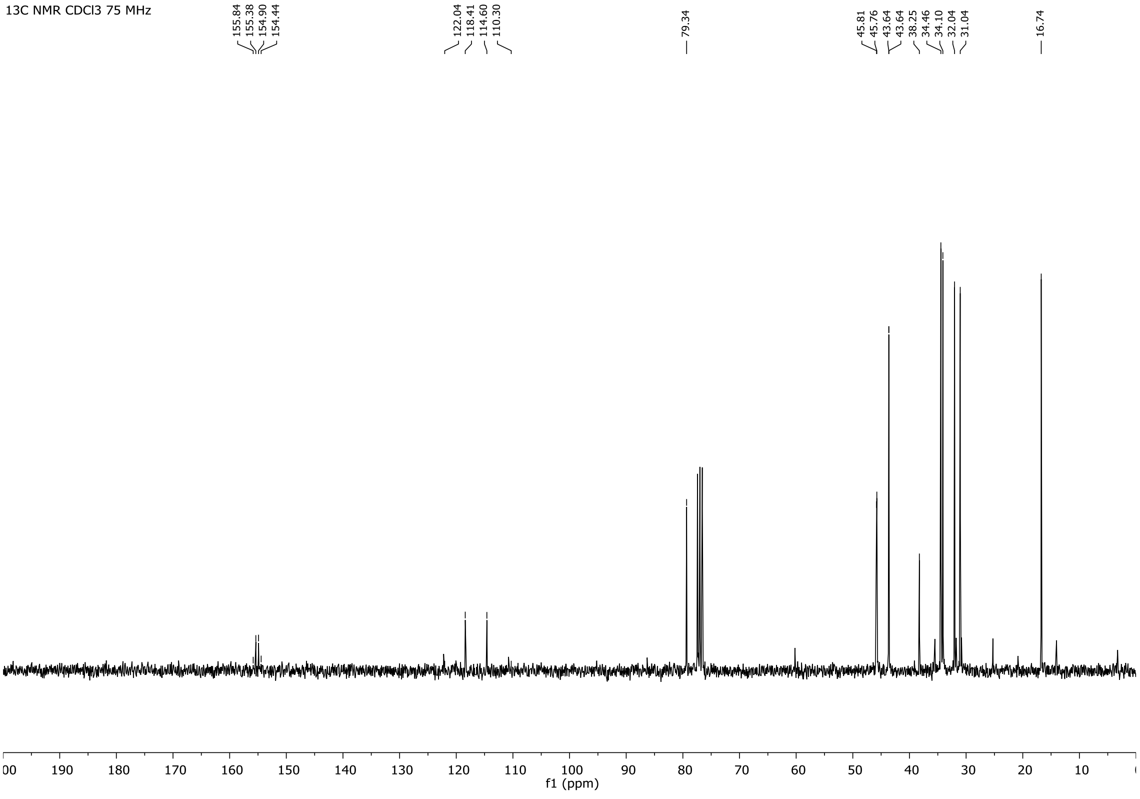

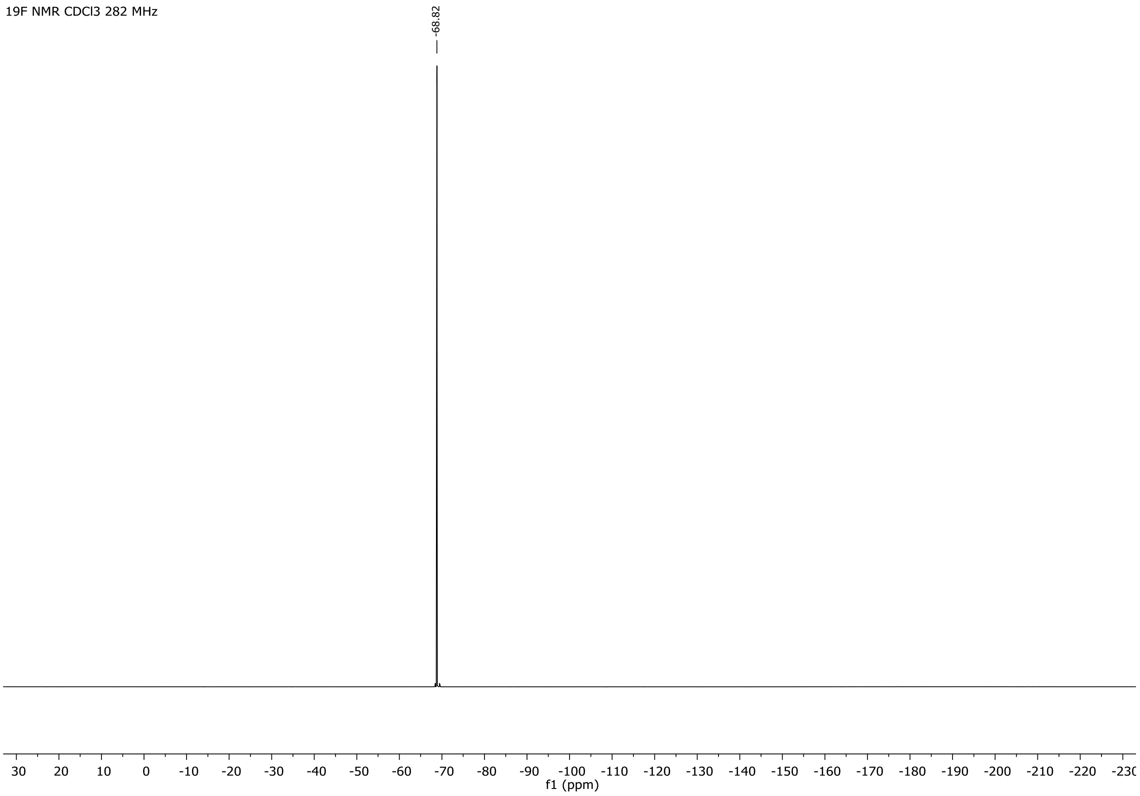
19F NMR CDCl3 282 MHz
-68.82
30 20 10 0 -10 -20 -30 -40 -50 -60 -70 -80 -90 -100 -110 -120 -130 -140 -150 -160 -170 -180 -190 -200 -210 -220 -230
f1 (ppm)

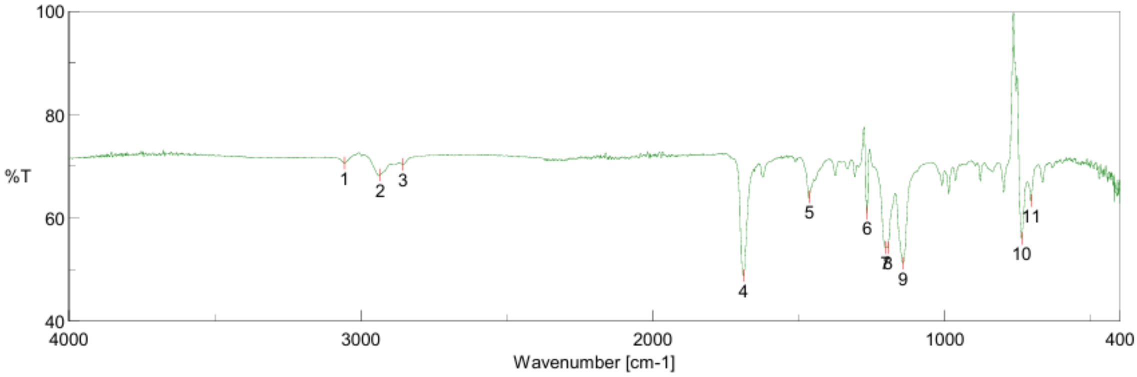
100
80
%T
60
40
1
2
3
4
5
6
7
8
9
10
11
4000
3000
2000
1000
400
Wavenumber [cm-1]

benzyl 2-bromo-8-azaspiro[4.5]dec-1-ene-8-carboxylate (8a)

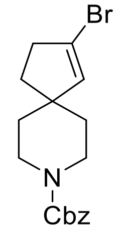

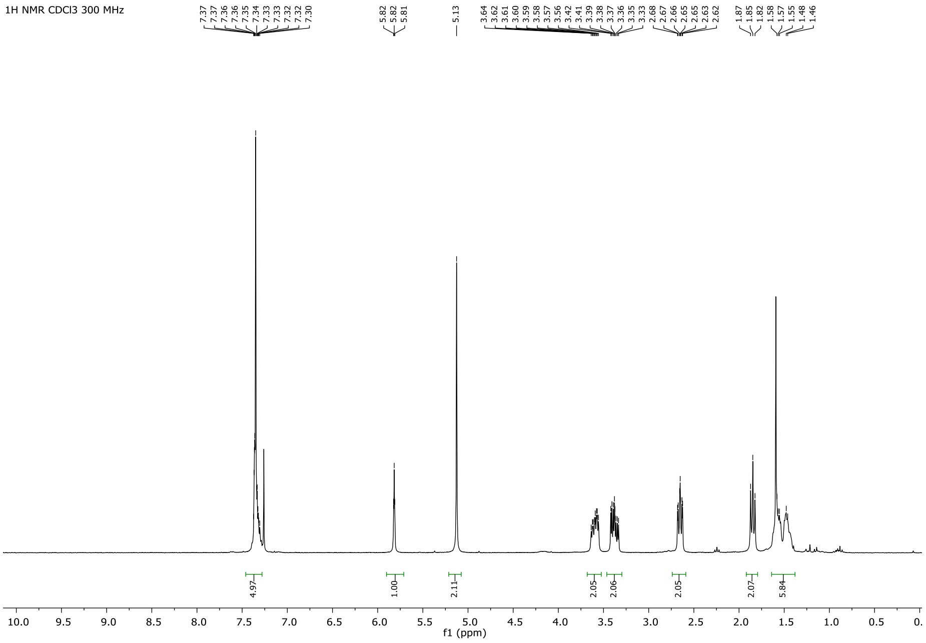

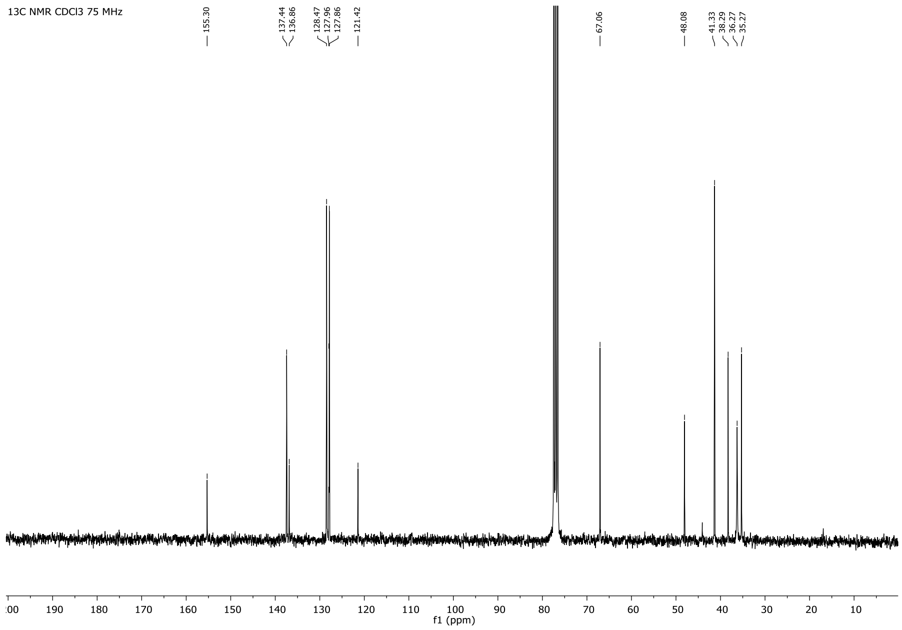

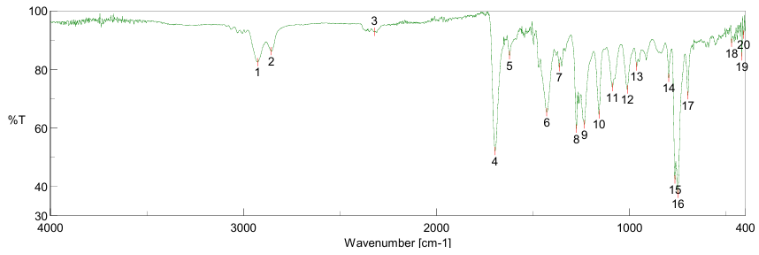

2-bromo-8-tosyl-8-azaspiro[4.5]dec-1-ene (9a)

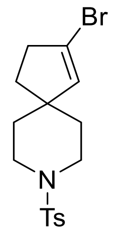

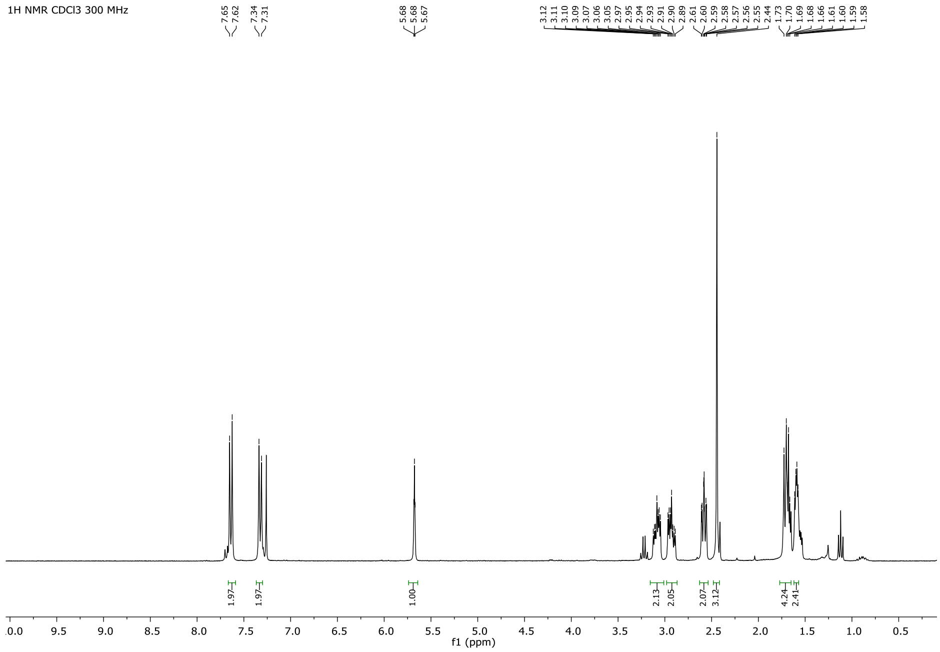

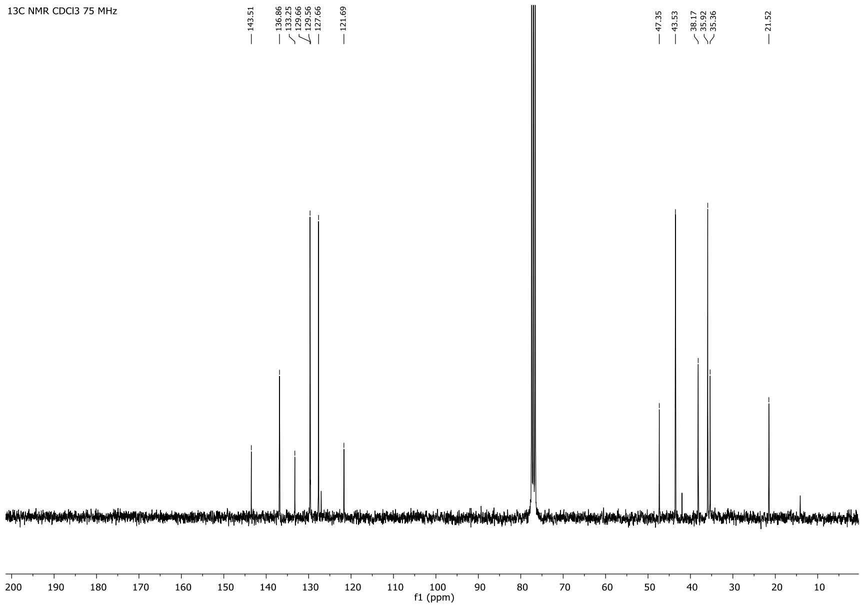

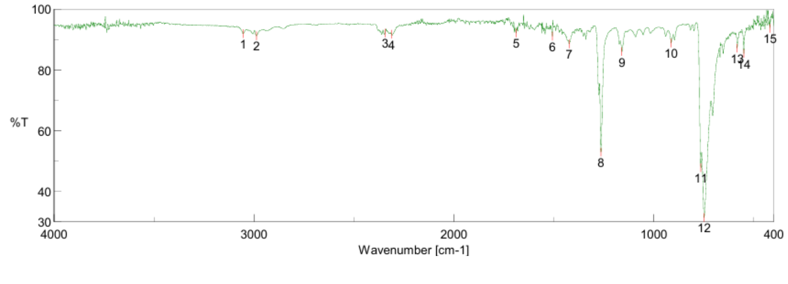

2-bromo-8,8-difluorospiro[4.5]dec-1-ene (10a)

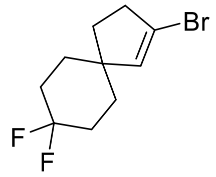

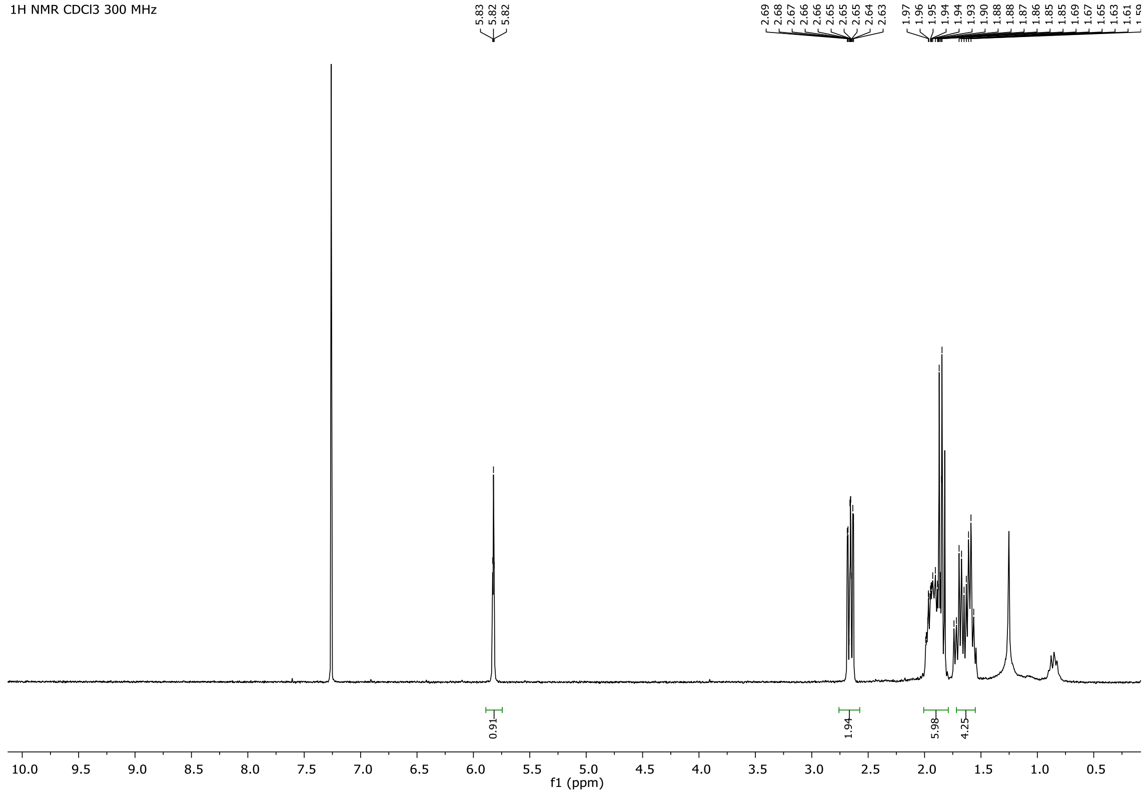

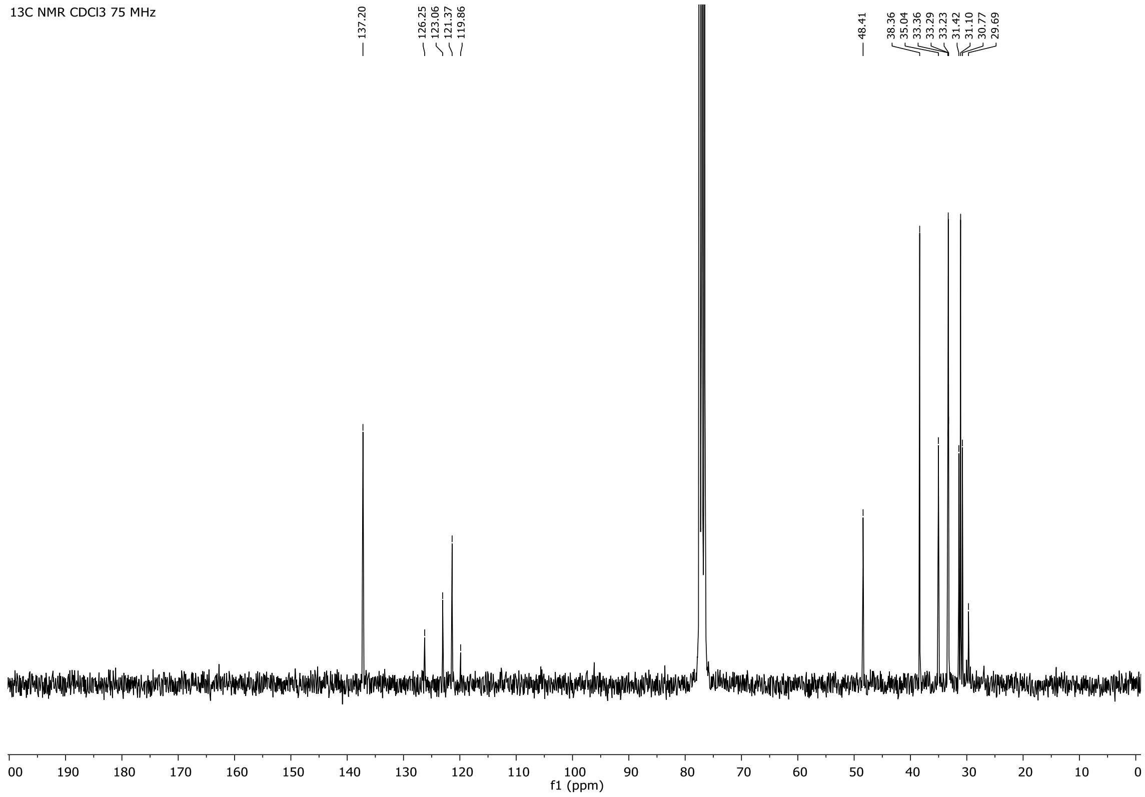

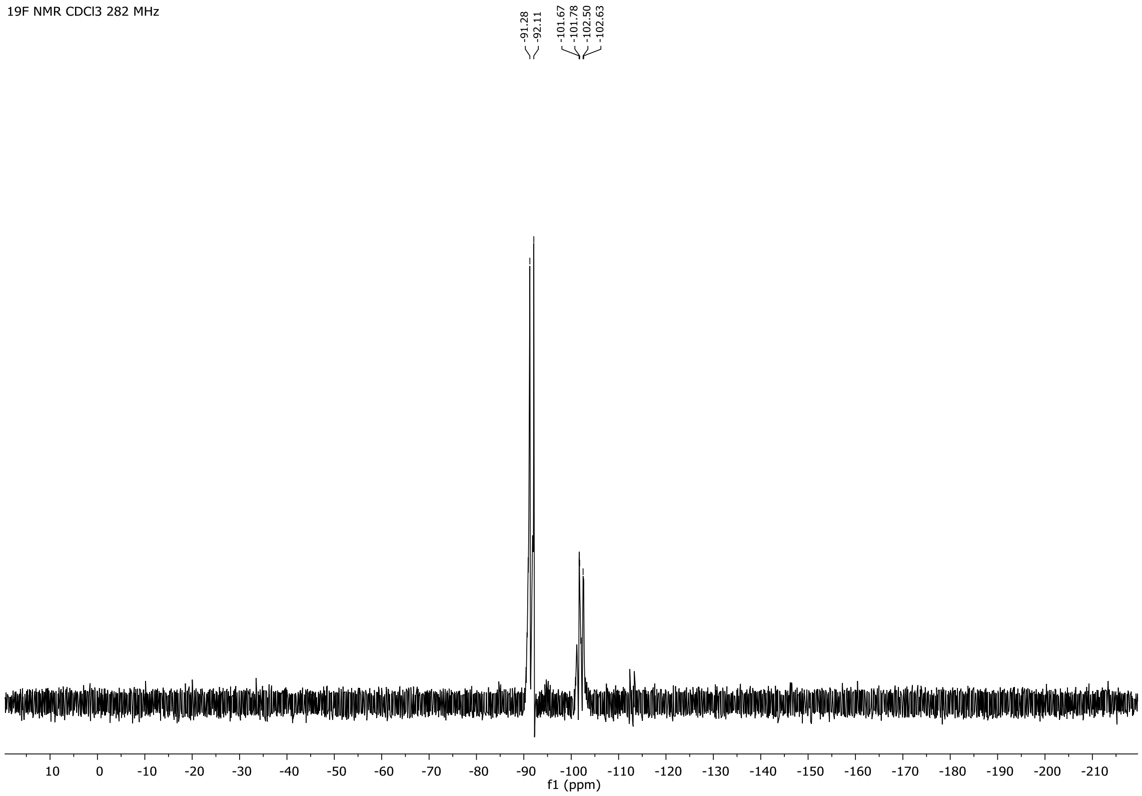
19F NMR CDCl3 282 MHz
-91.28
-92.11
-101.67
-101.78
-102.50
-102.63
10
0
-10
-20
-30
-40
-50
-60
-70
-80
-90
-100
-110
-120
-130
-140
-150
-160
-170
-180
-190
-200
-210
f1 (ppm)

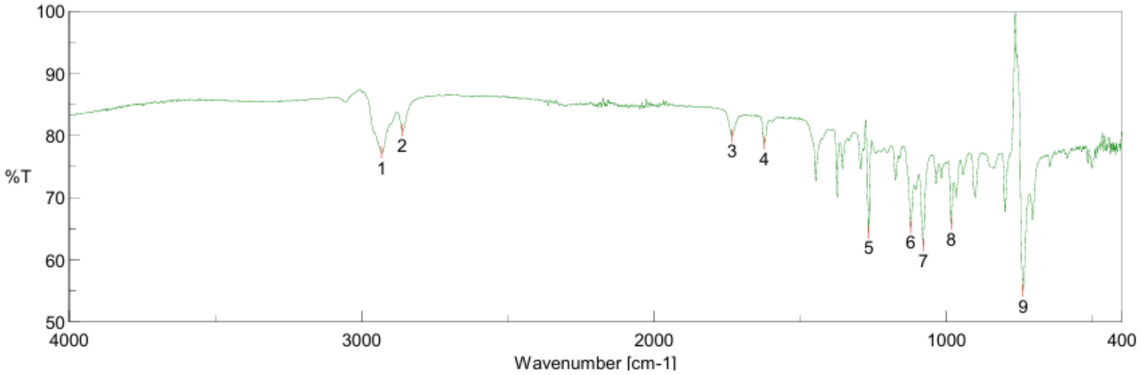
100
90
80
70
60
50
%T
1
2
3
4
5
6
7
8
9
4000
3000
2000
1000
400
Wavenumber [cm-1]

2-bromo-8-thiaspiro[4.5]dec-1-ene 8,8-dioxide (11a)

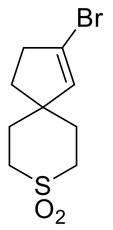

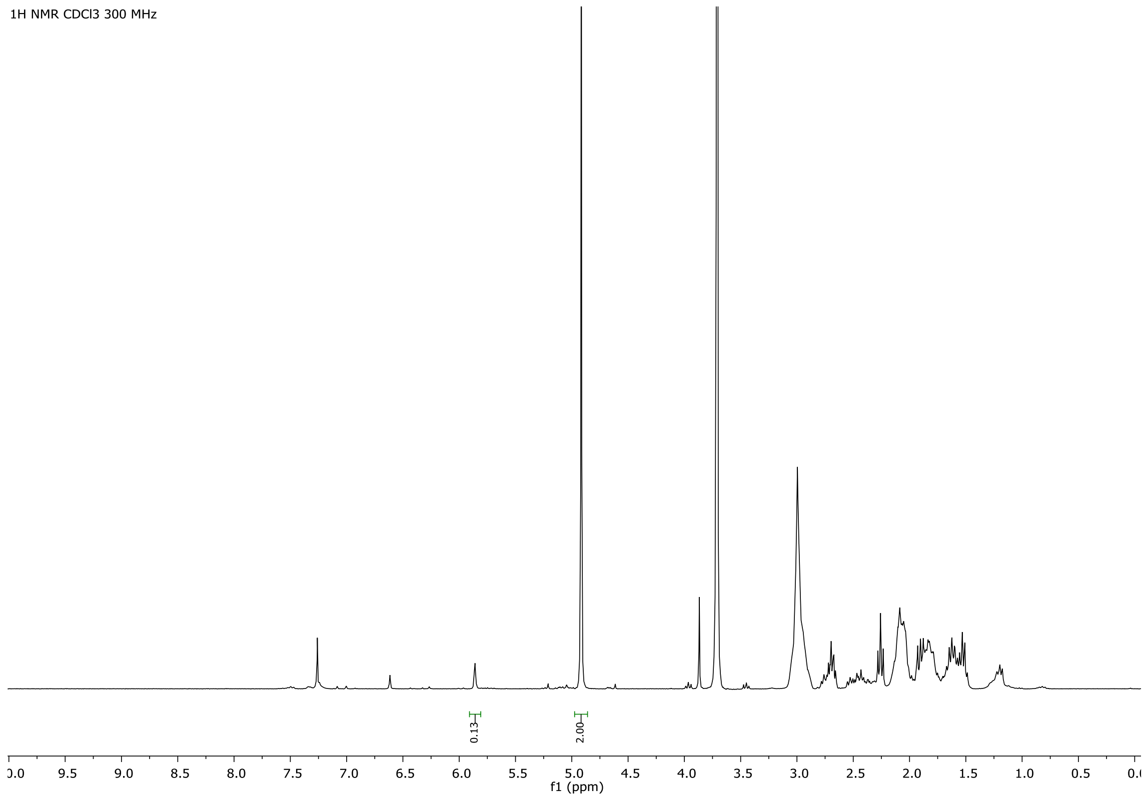

2-bromo-8,8-dimethylspiro[4.5]dec-1-ene (12a)

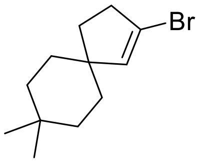

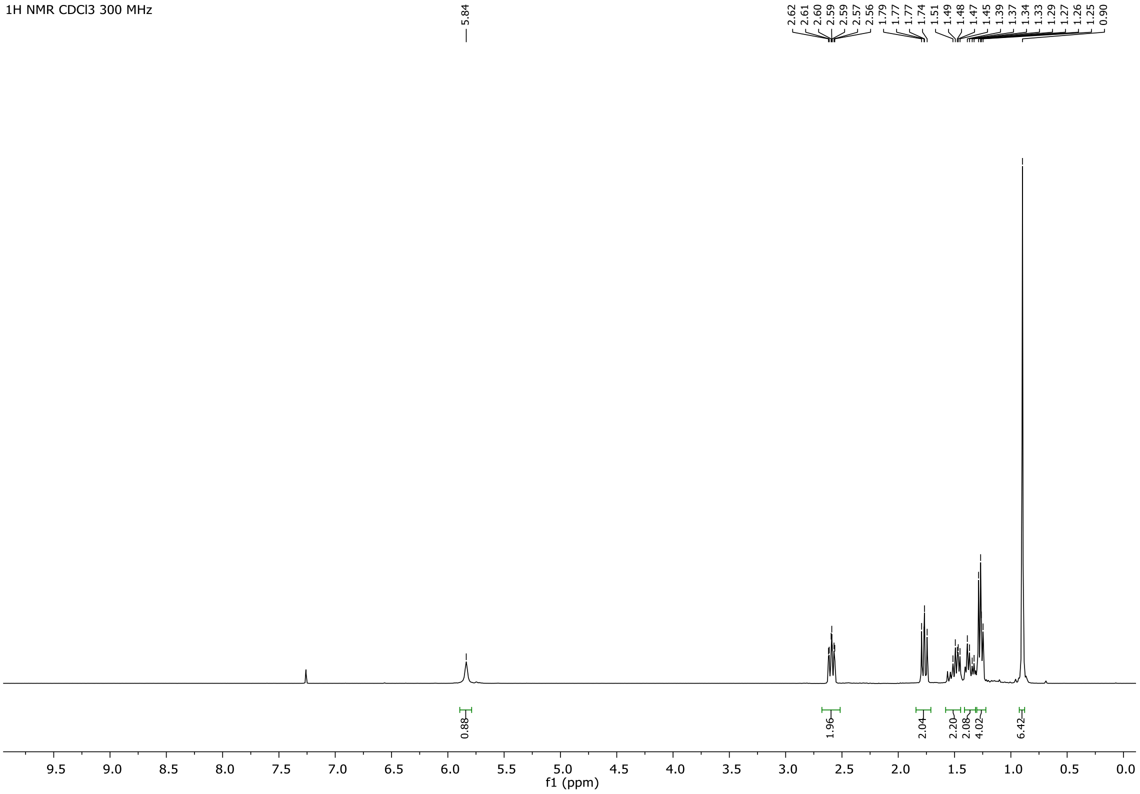

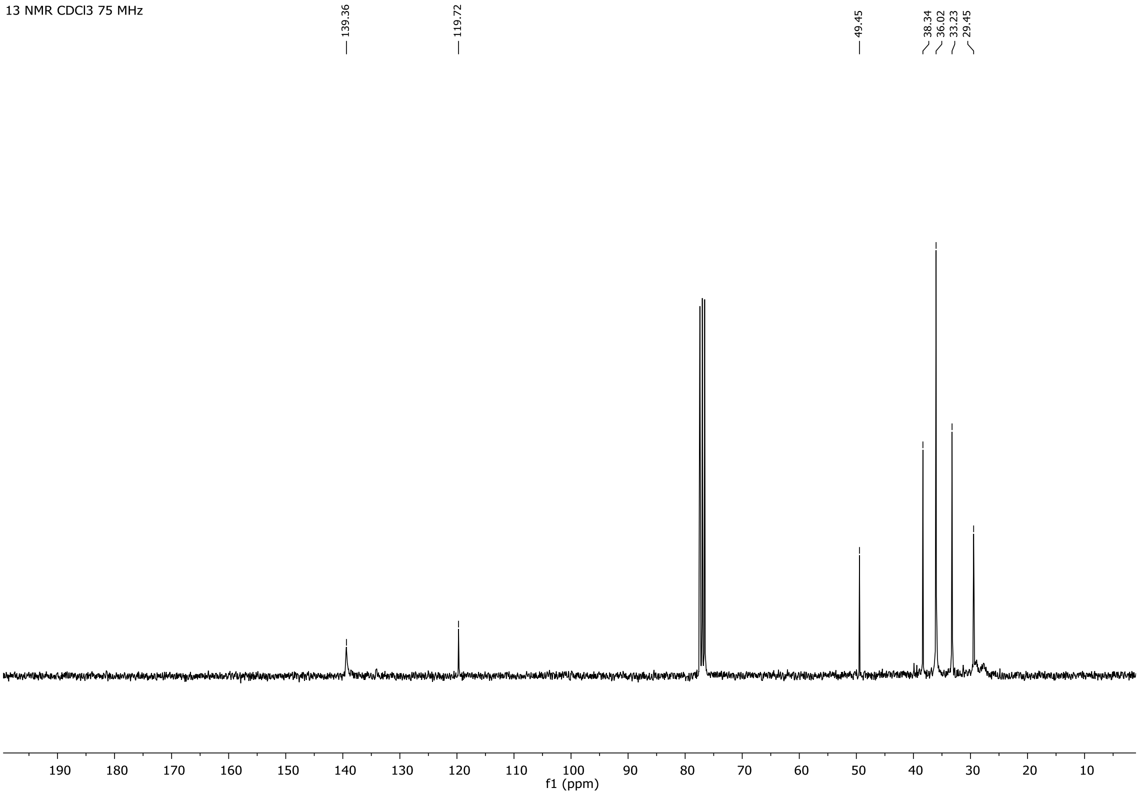

2-bromo-8-oxaspiro[4.6]undec-1-ene (13a)

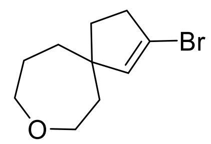

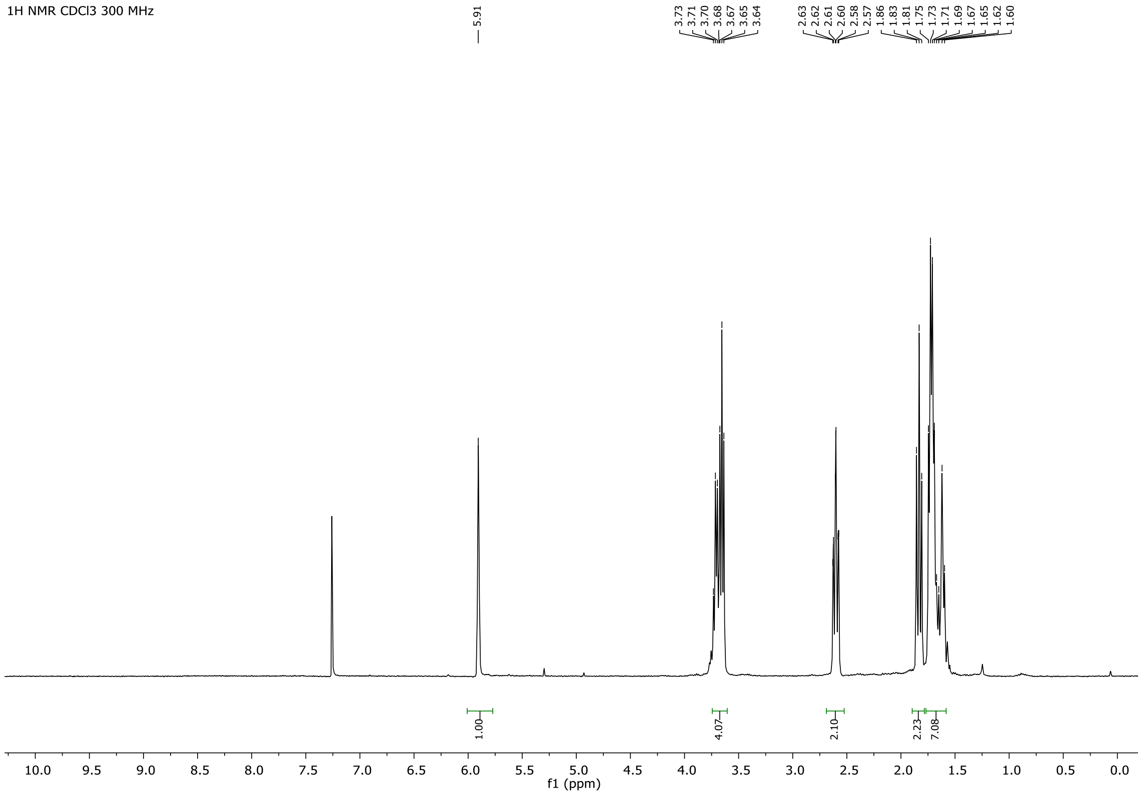

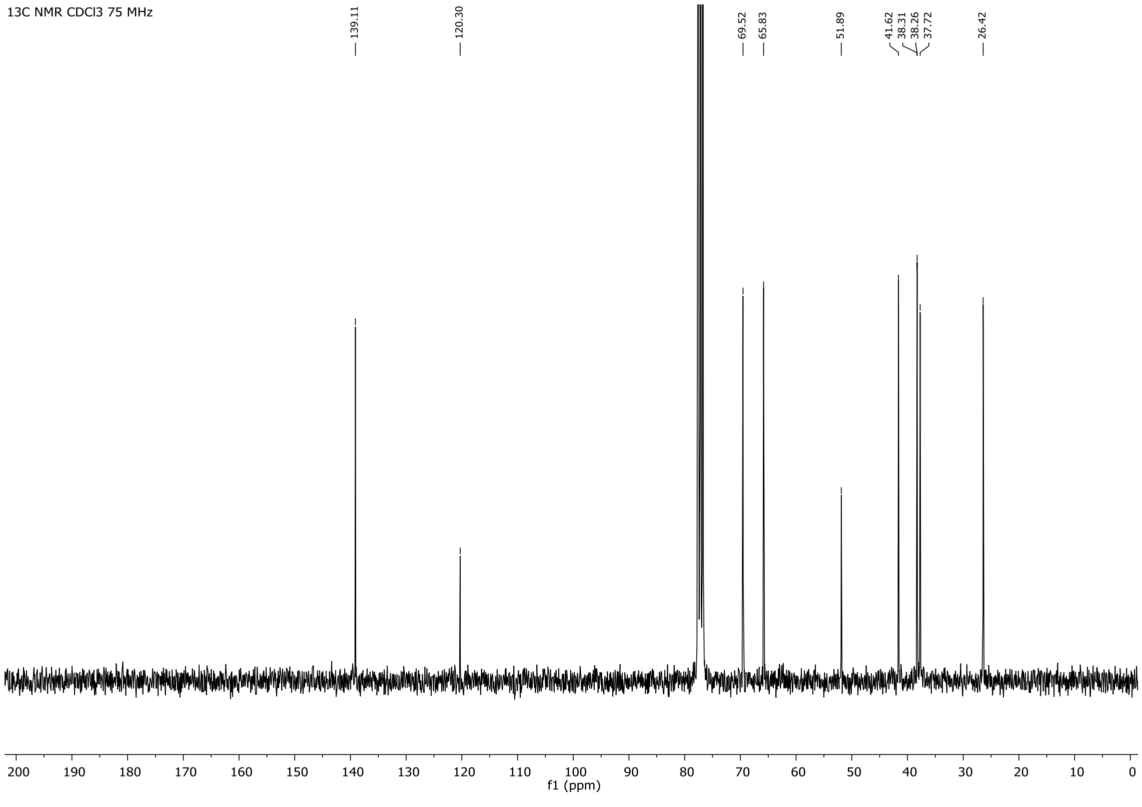

2-bromo-8-oxaspiro[4.6]undec-1-en-9-one (14a)

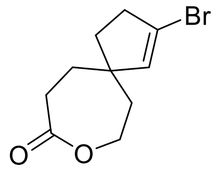

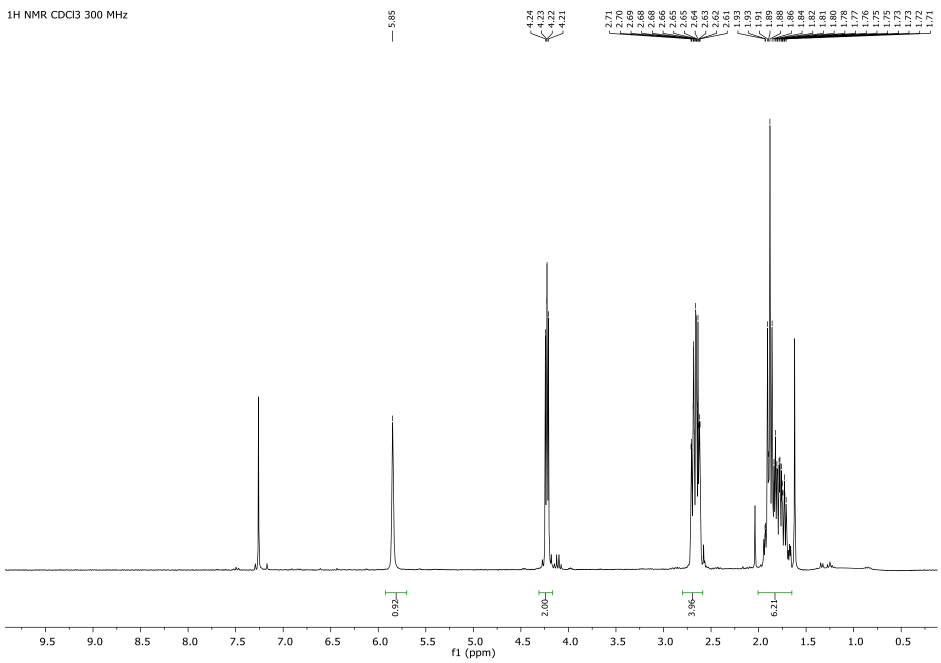

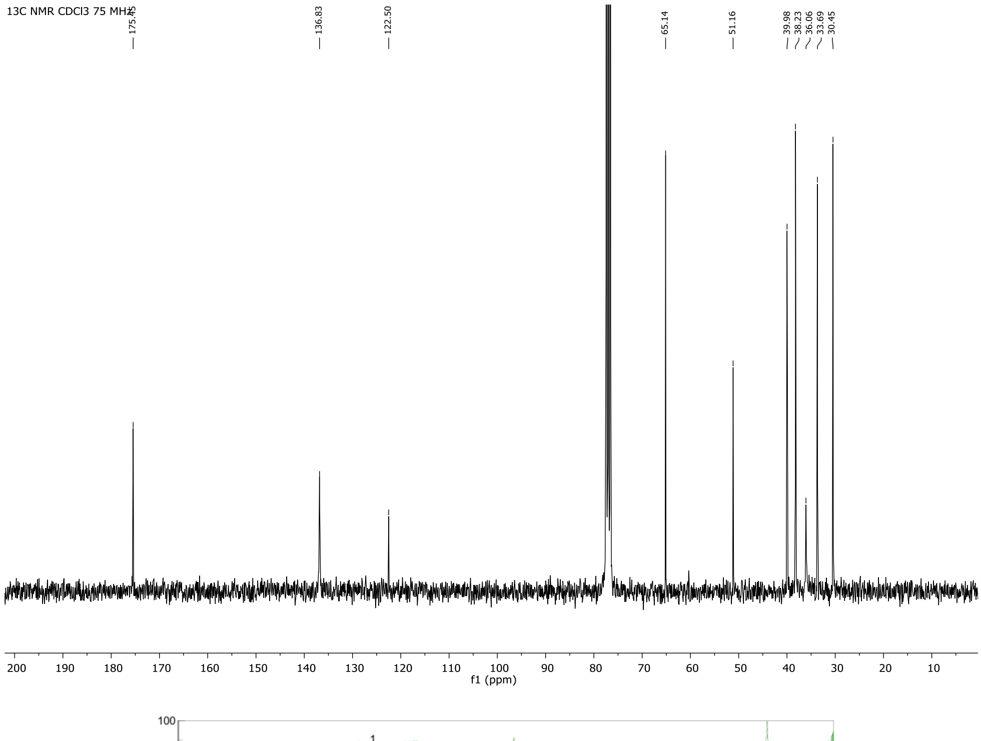

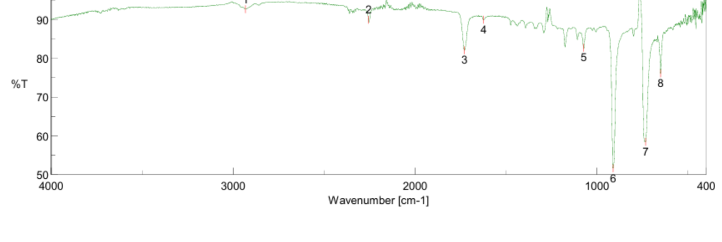

8-bromo-2,2-difluorodispiro[3.1.46.14]undec-7-ene (15a)

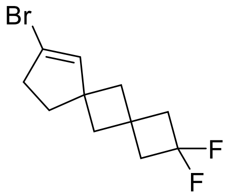

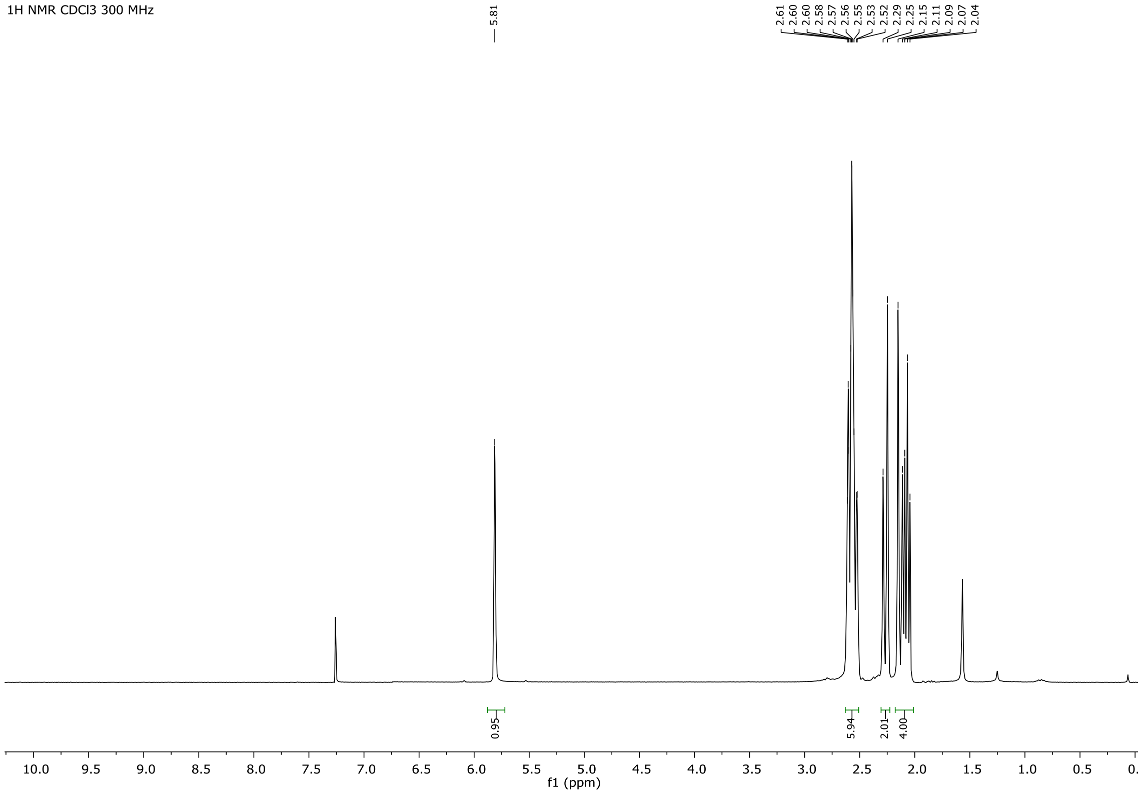

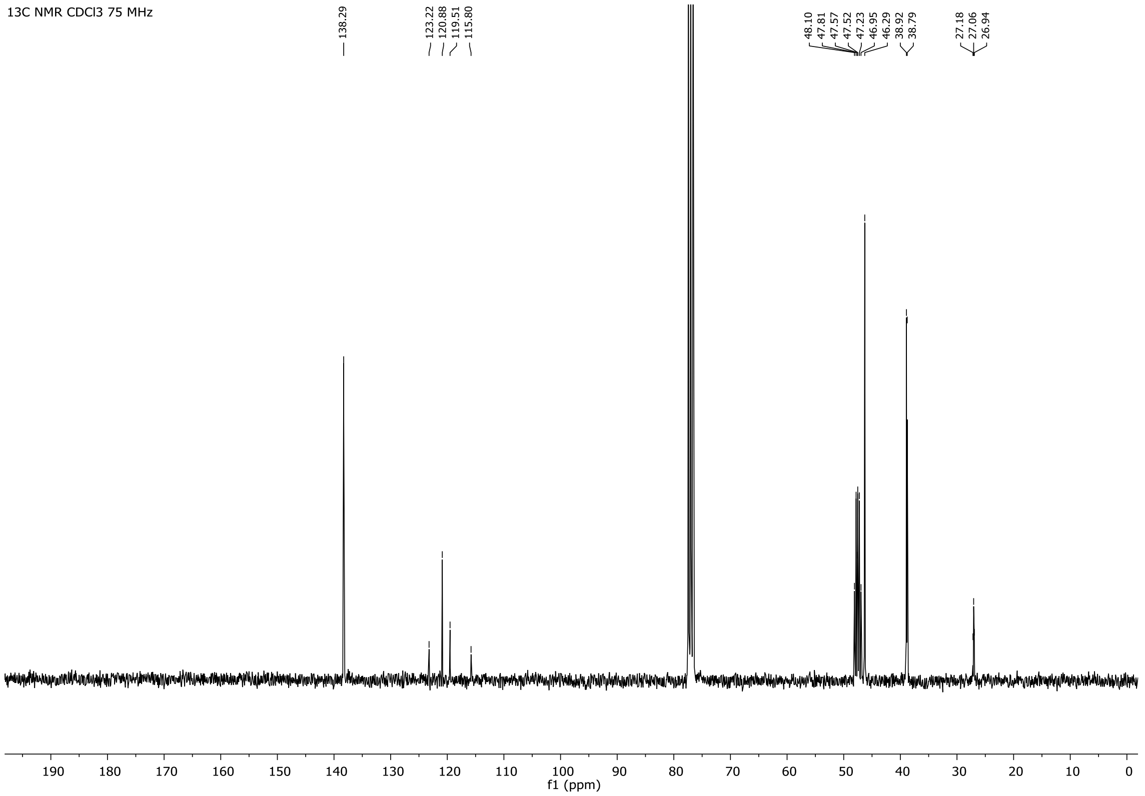

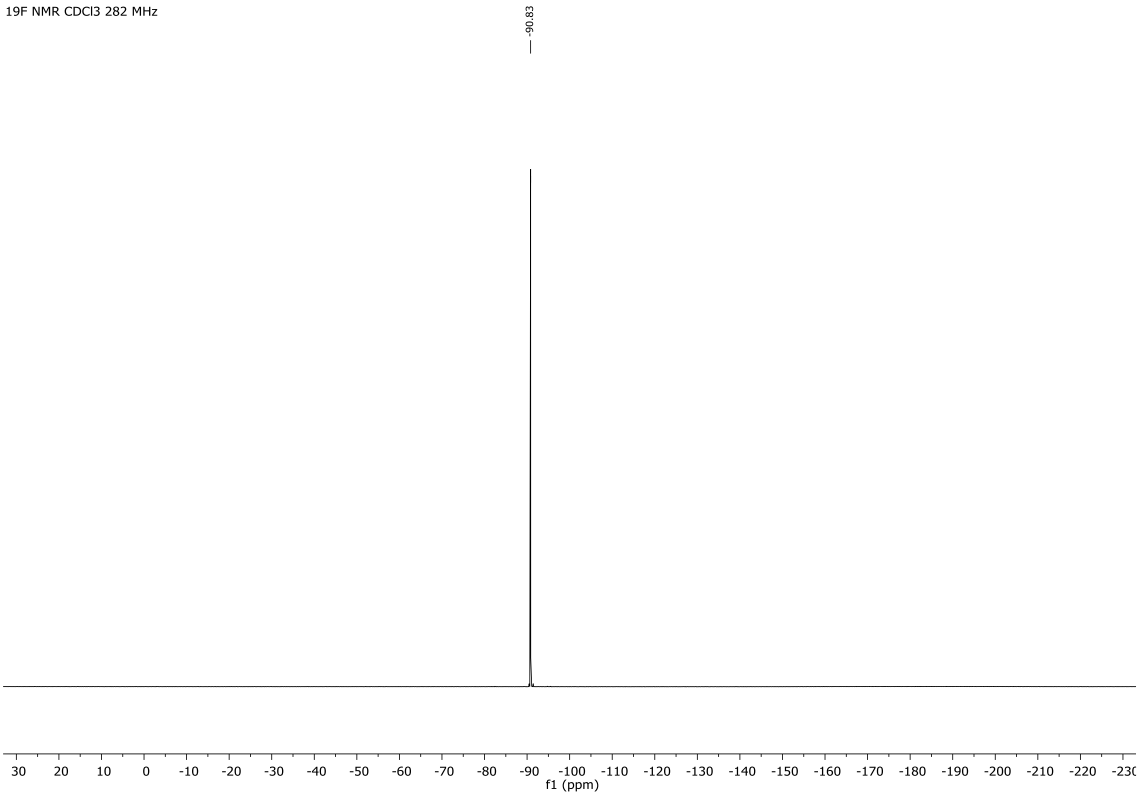
19F NMR CDCl3 282 MHz
-90.83
30 20 10 0 -10 -20 -30 -40 -50 -60 -70 -80 -90 -100 -110 -120 -130 -140 -150 -160 -170 -180 -190 -200 -210 -220 -230
f1 (ppm)

14-bromo-10-oxadispiro[4.1.57.25]tetradec-13-ene (16a)

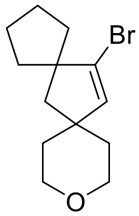

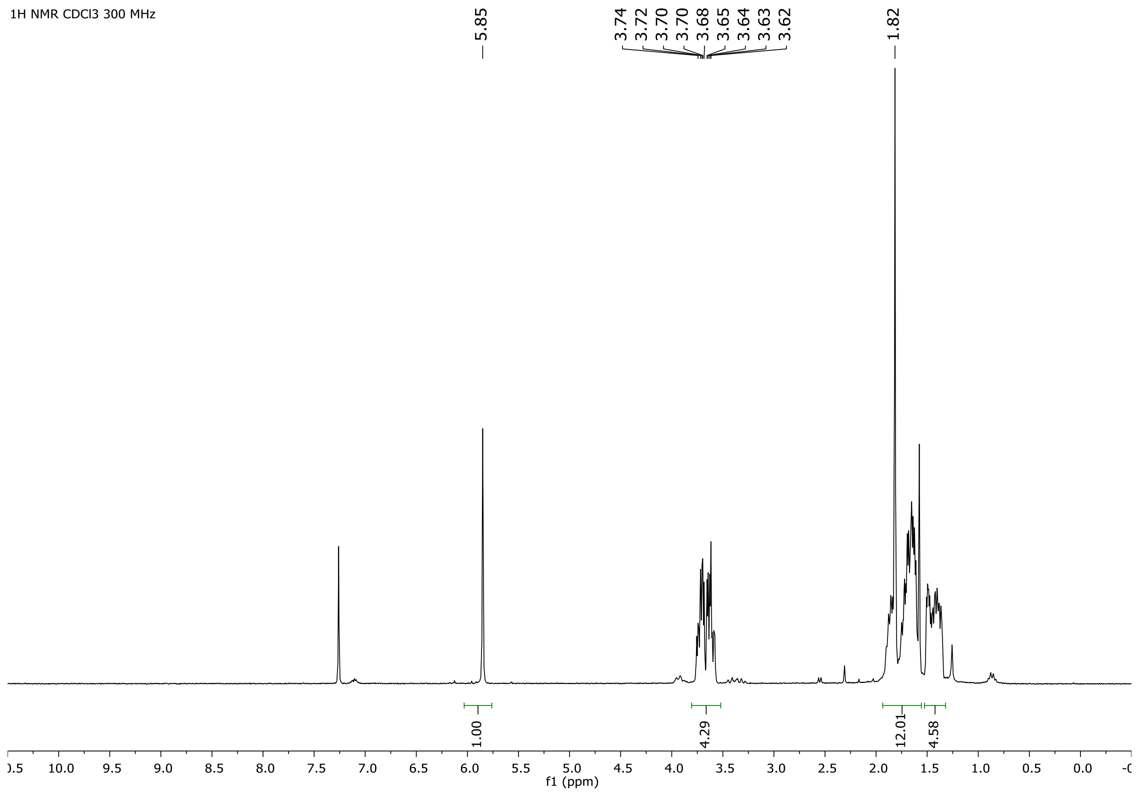

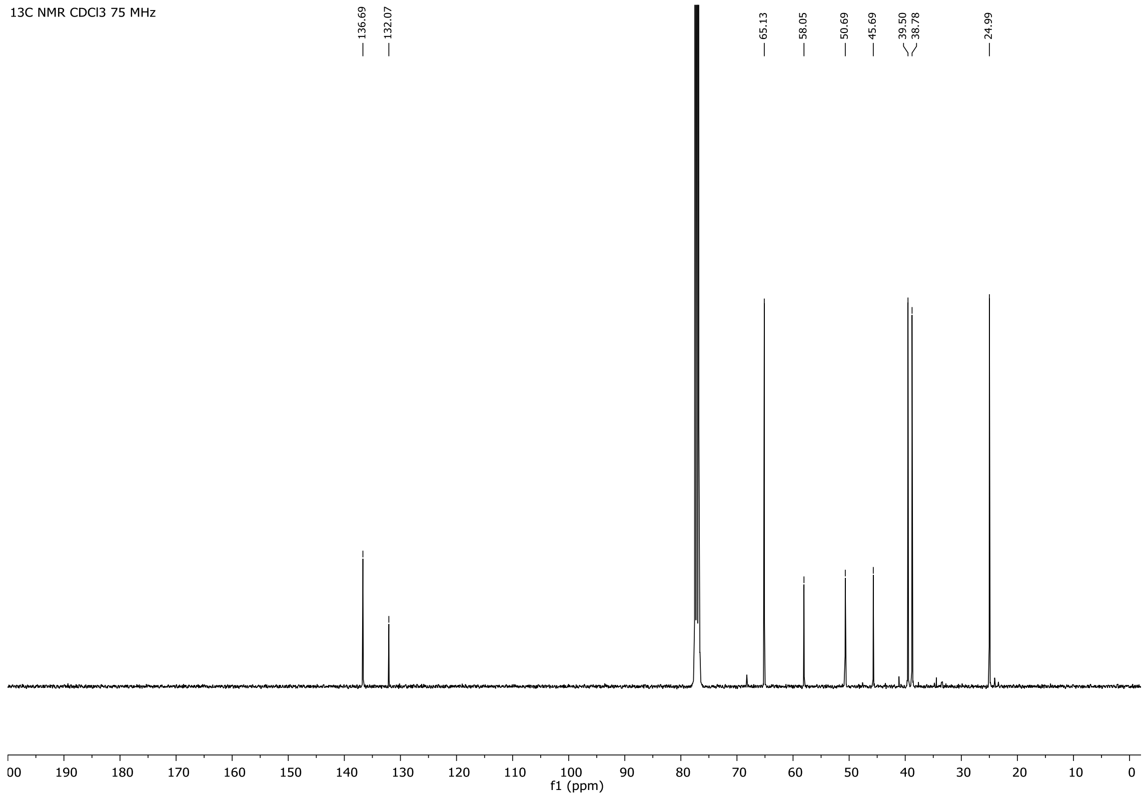

1-(2-bromo-7-azaspiro[4.6]undec-1-en-7-yl)-2,2,2-trifluoroethan-1-one (17a)

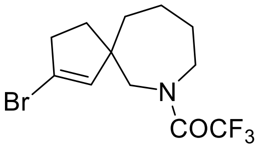

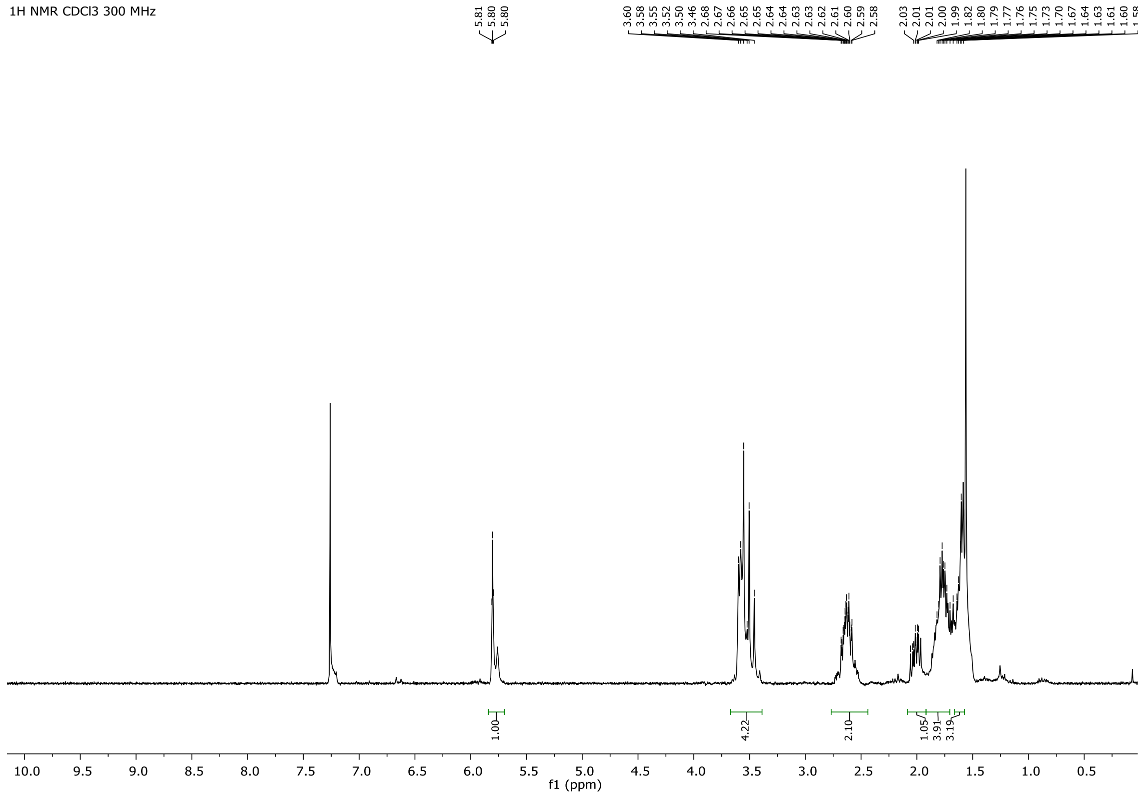

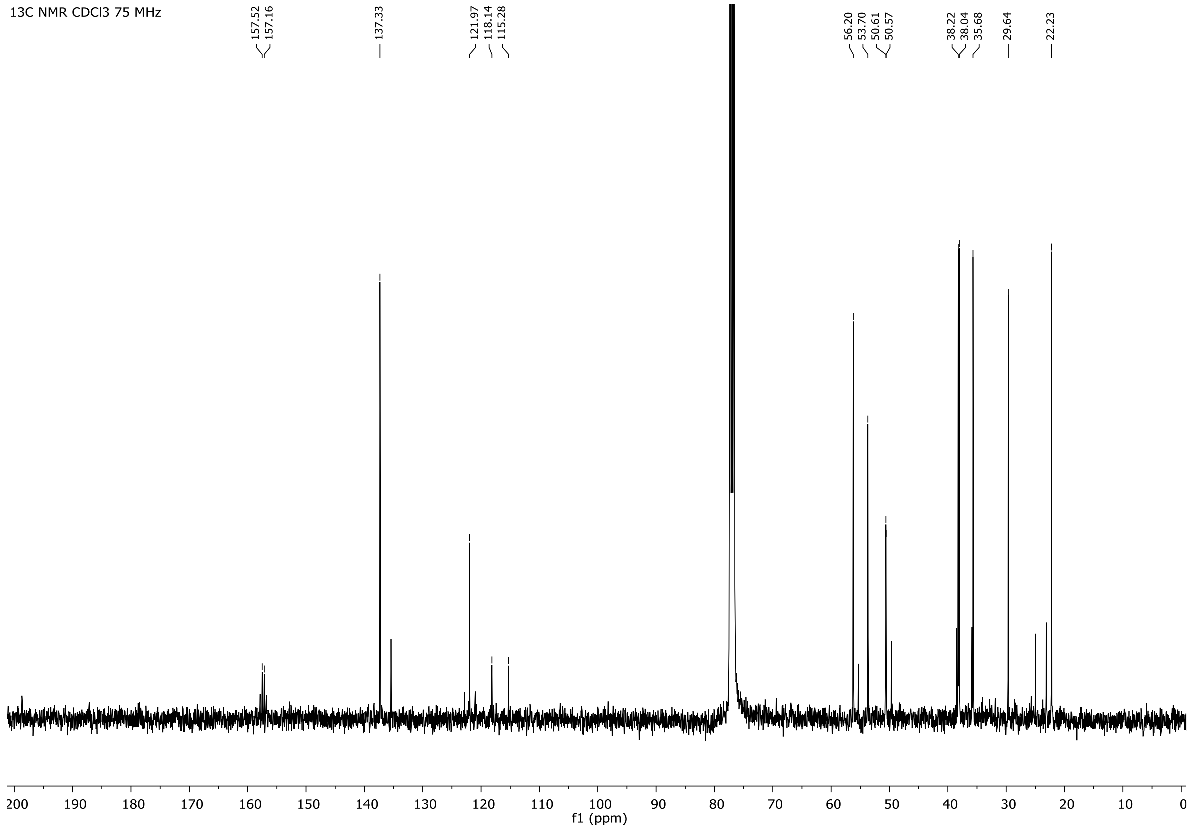

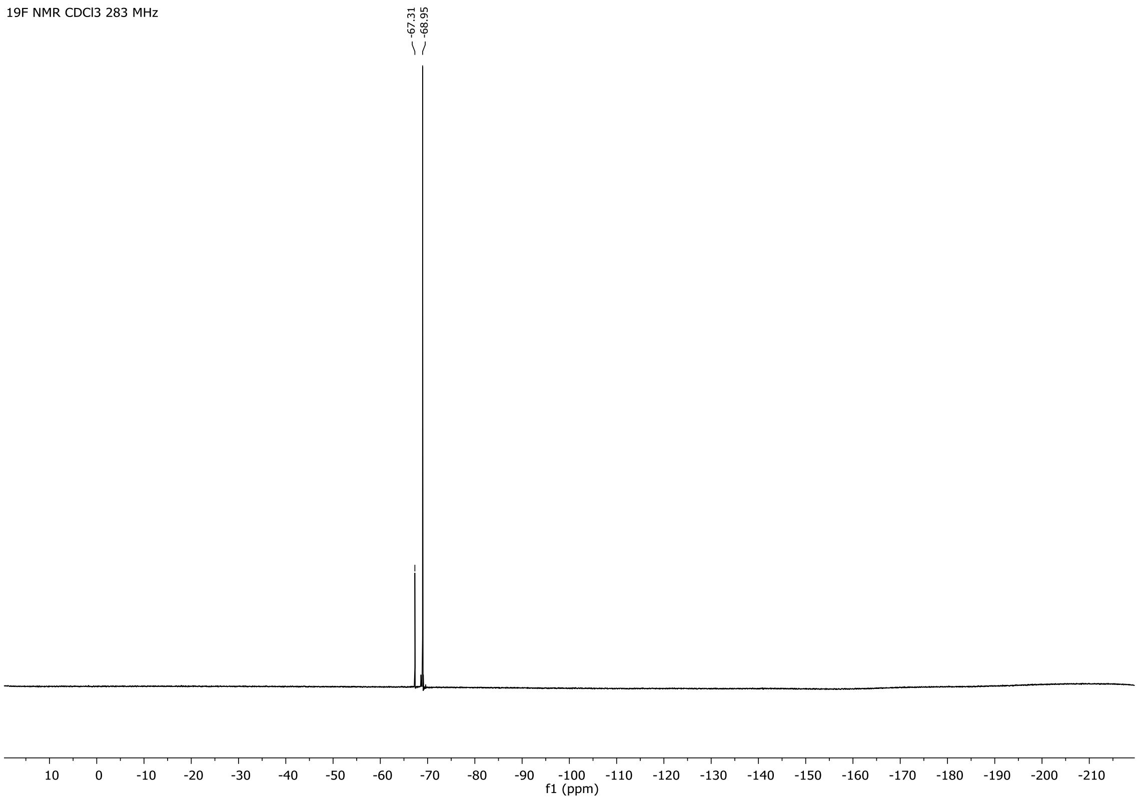

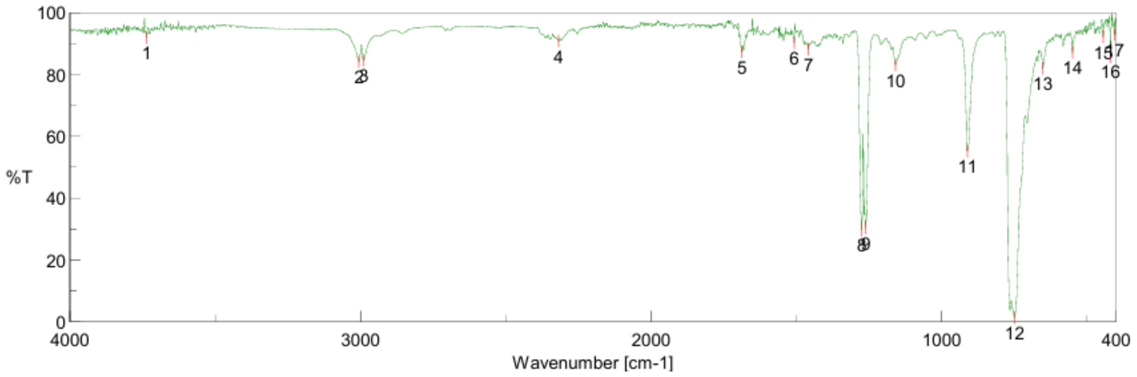

2-bromospiro[4.5]dec-1-en-7-one (18a)

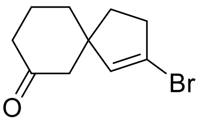

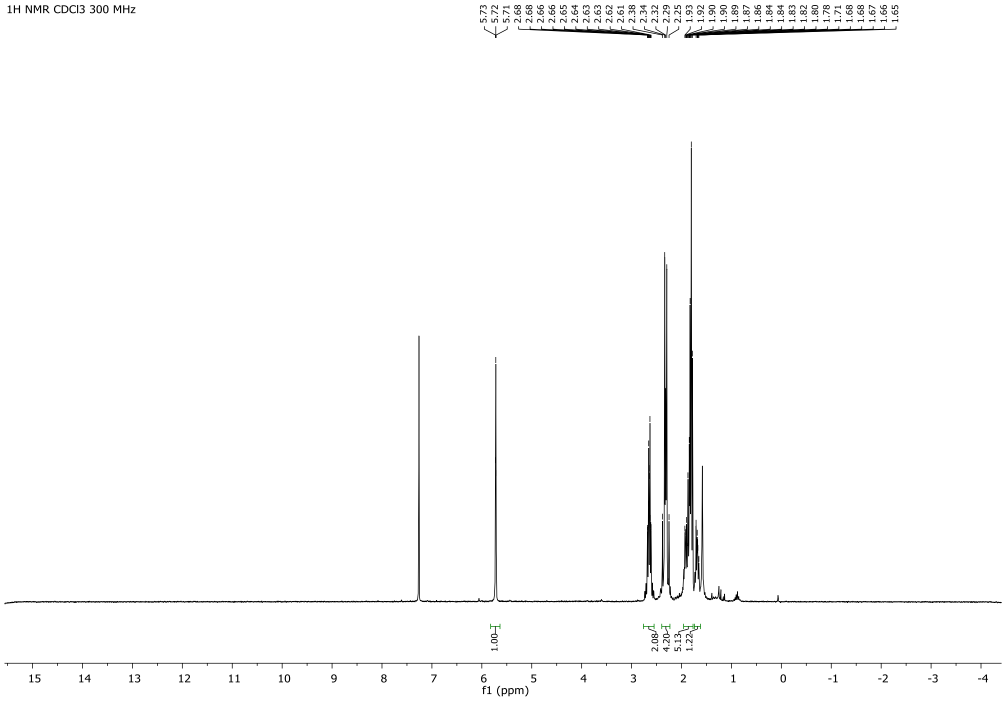

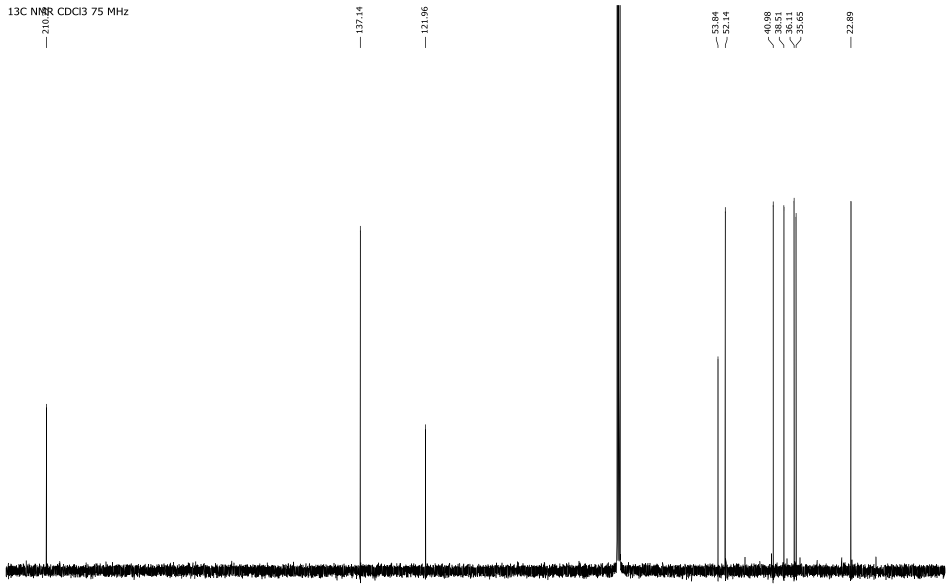

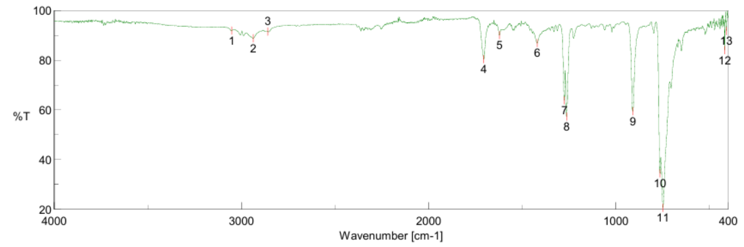

2-bromo-7,7-difluorospiro[4.5]dec-1-ene (19a)

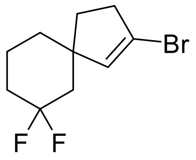

1H NMR CDCl3 300 MHz

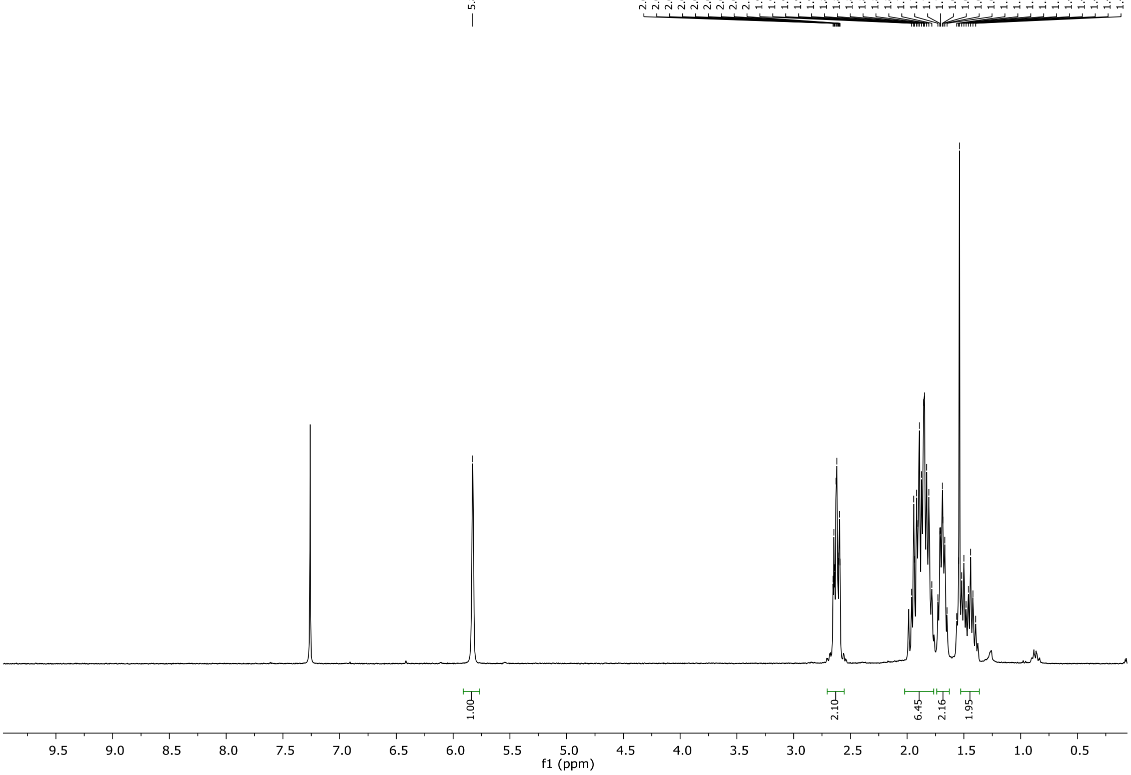

13C NMR CDCl3 75 MHz

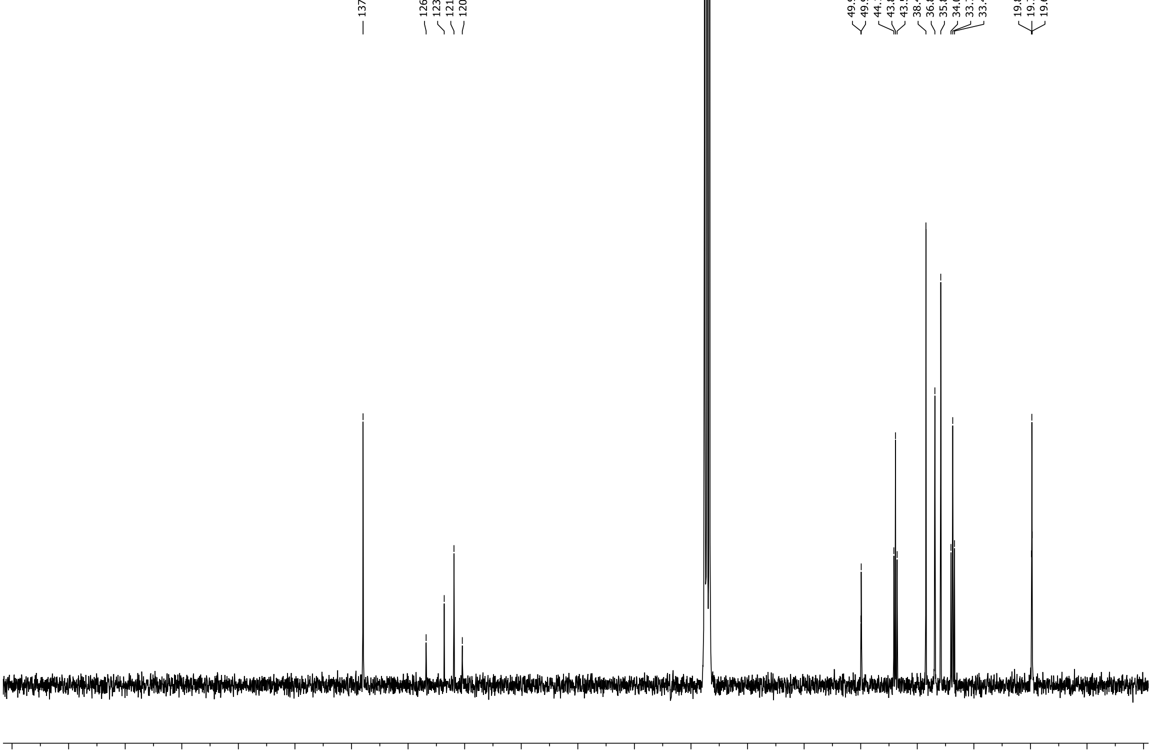

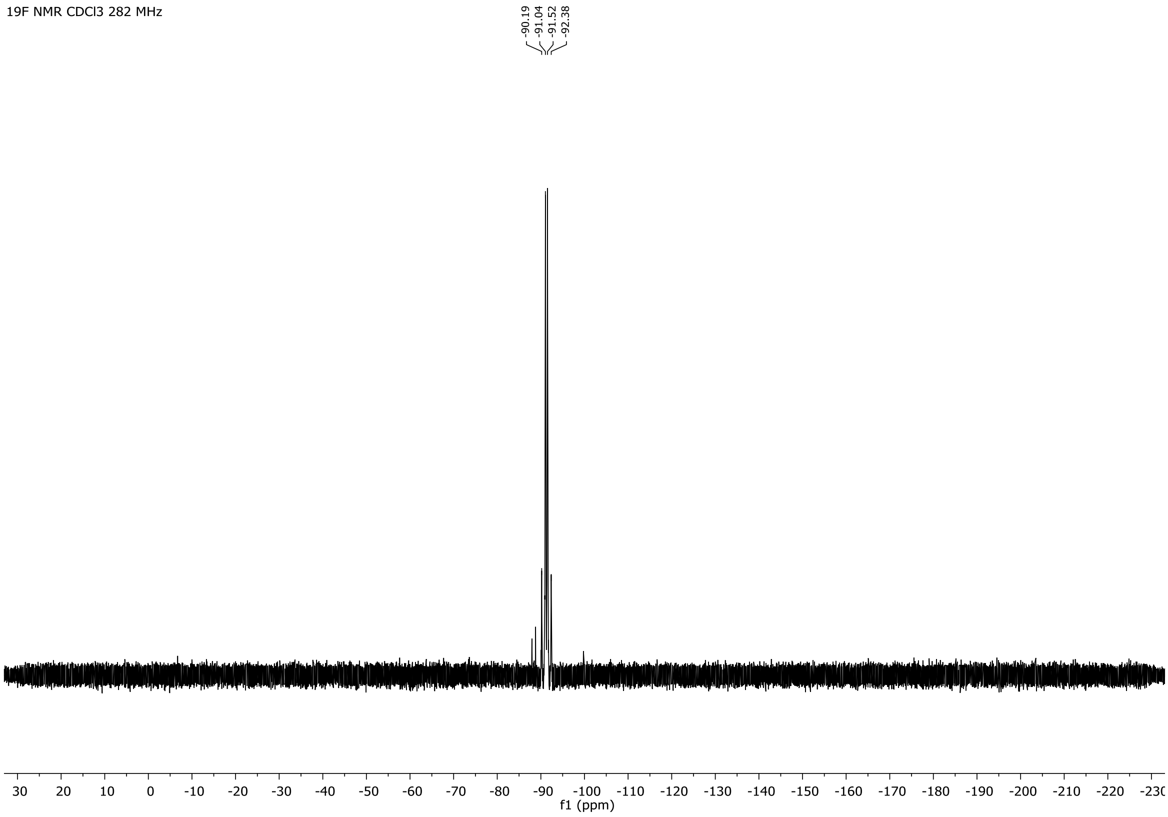
19F NMR CDCl3 282 MHz
-90.19
-91.04
-91.52
-92.38
f1 (ppm)

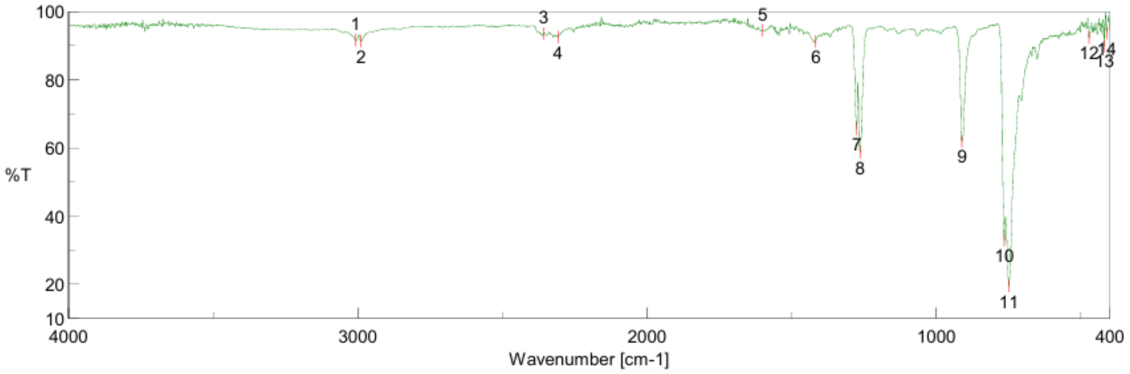
%T
Wavenumber [cm-1]

(Z)-1-bromo-4-(1-(tert-butoxycarbonyl)piperidin-4-yl)but-1-en-2-yl 4-(4-bromobut-3-yn-1-yl)piperidine-1-carboxylate (20b)

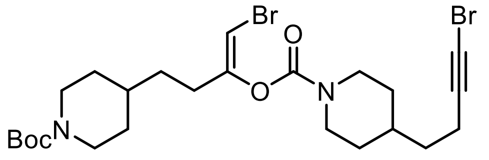

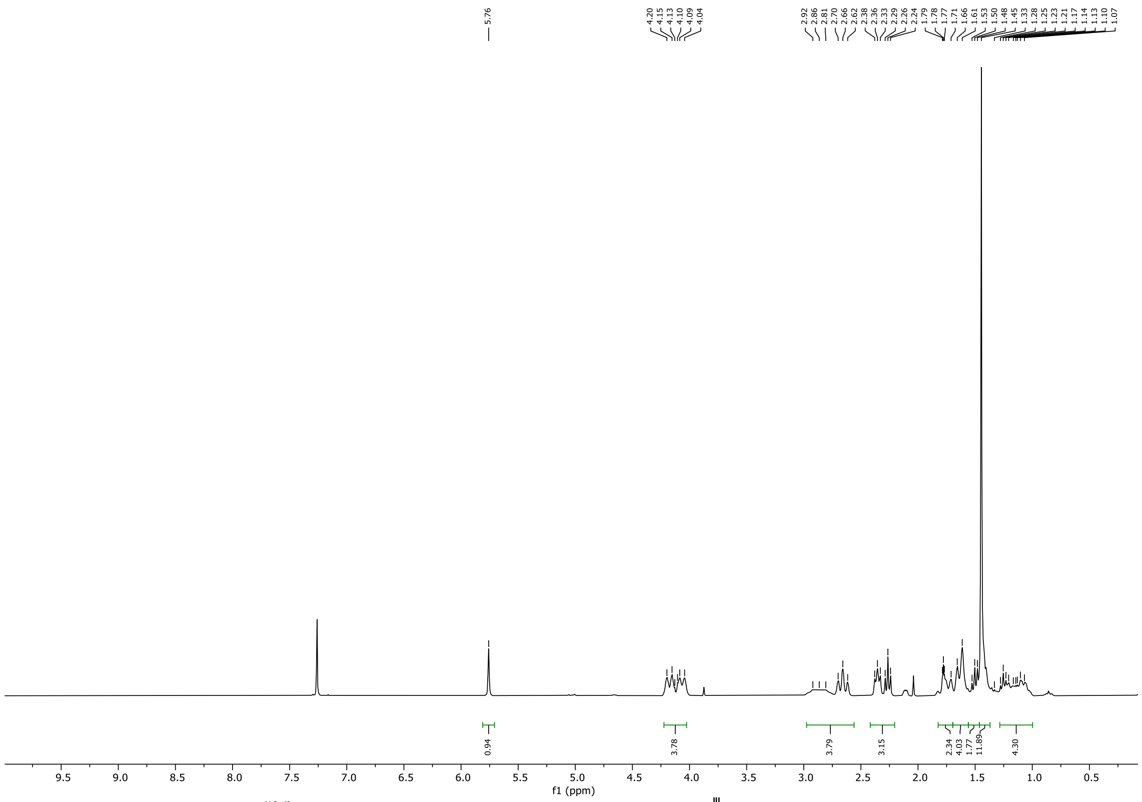

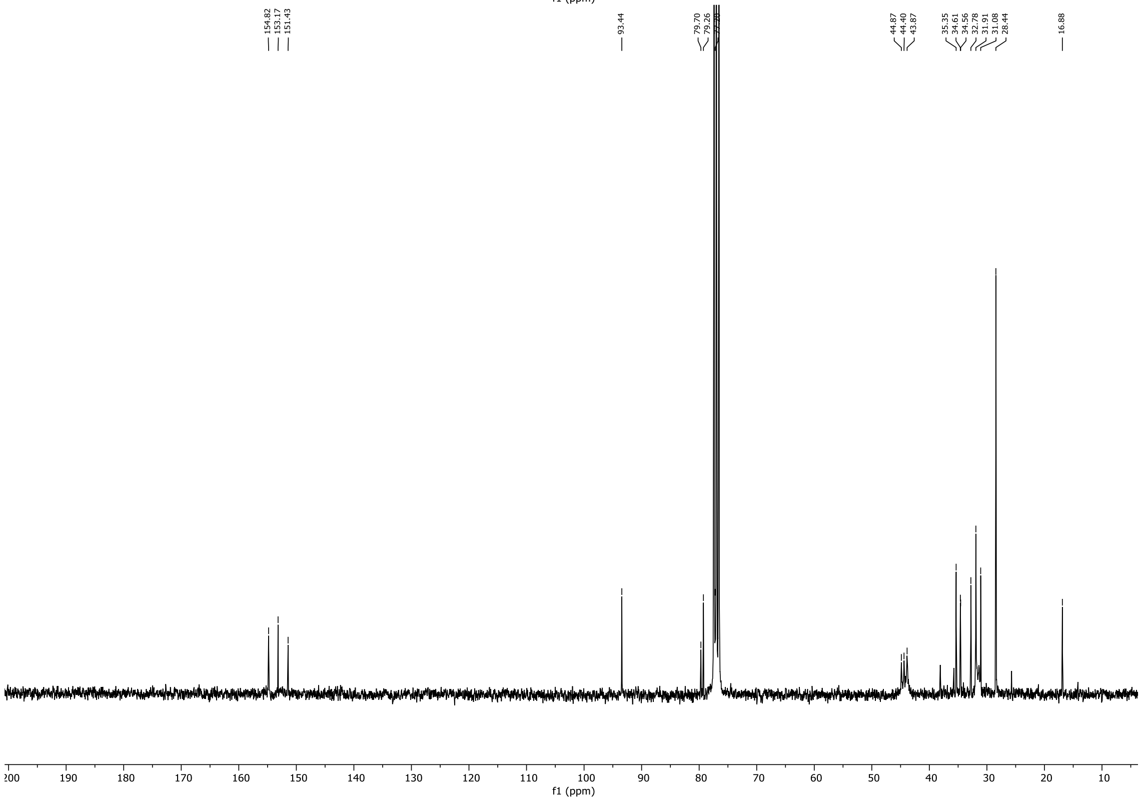

DERIVATIZATIONS

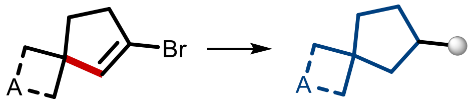

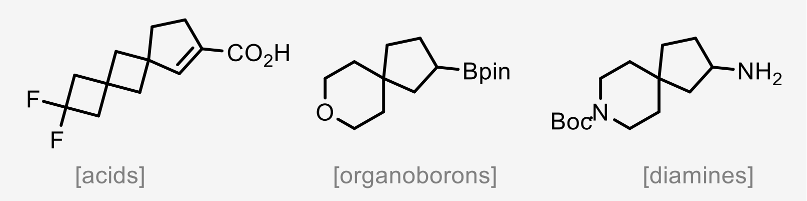

8-oxaspiro[4.5]dec-1-ene-2-carboxylic acid (21a-C=C)

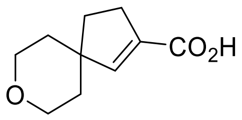

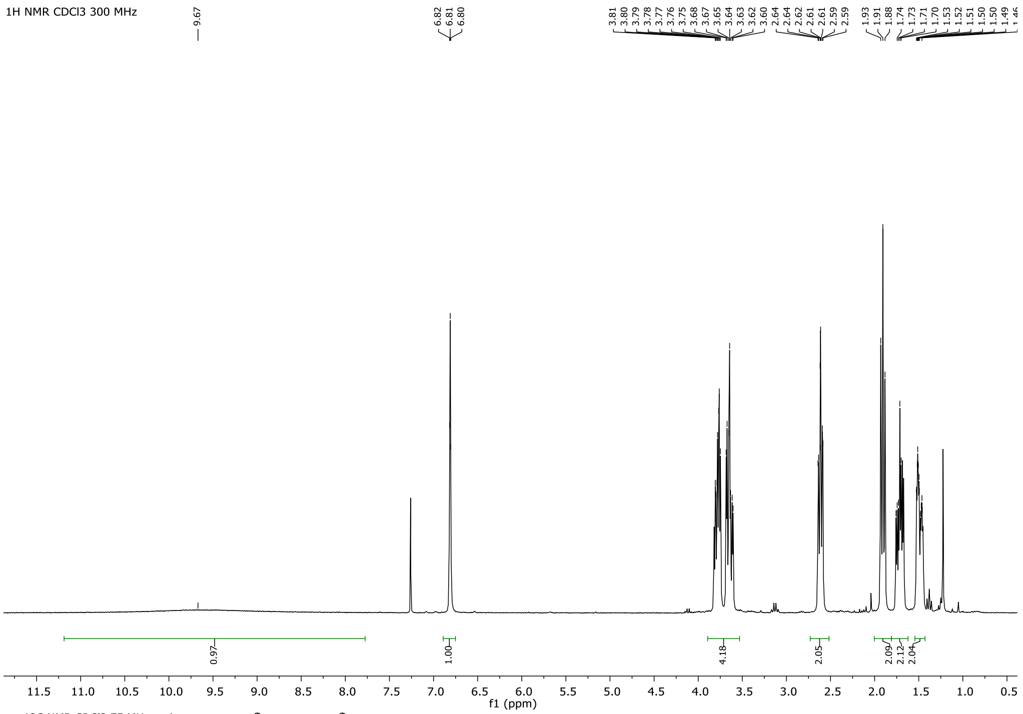

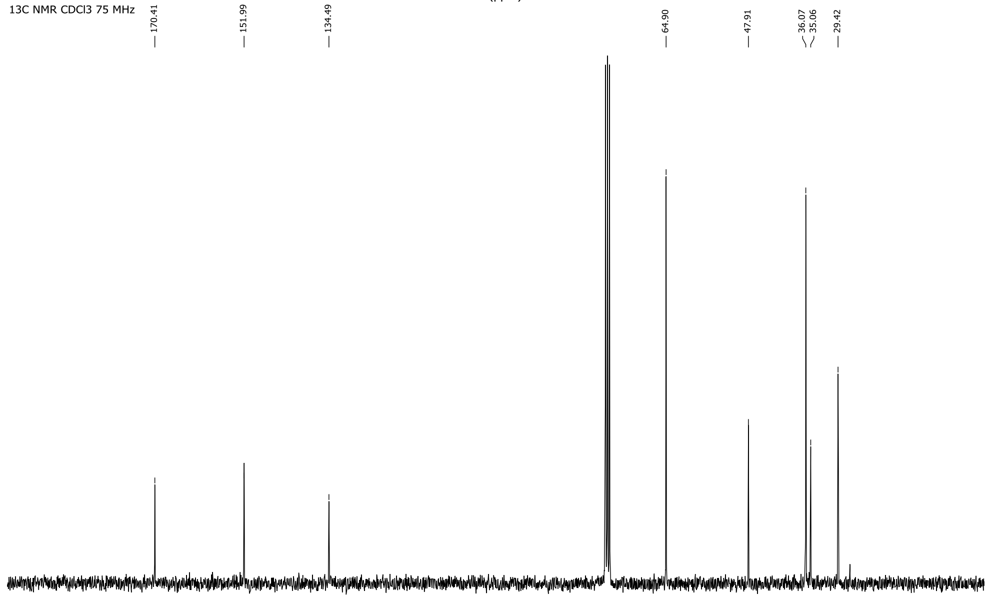

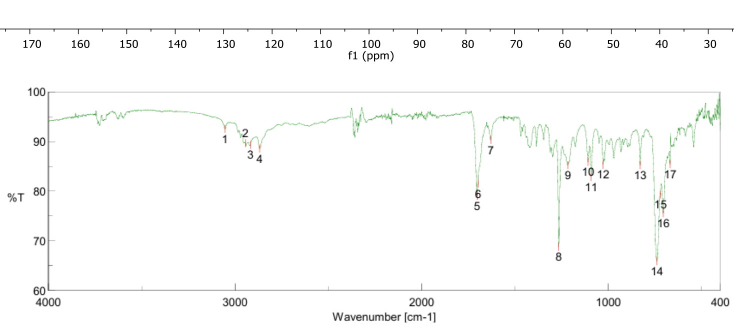

8-oxaspiro[4.5]decane-2-carboxylic acid (21a)

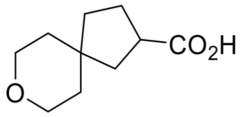

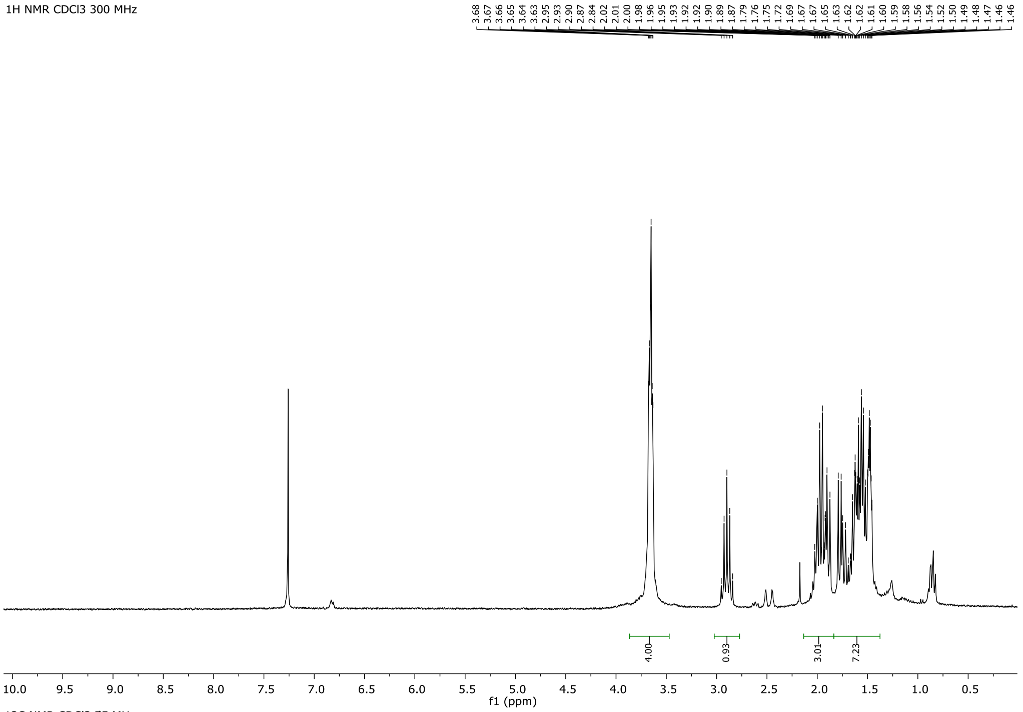

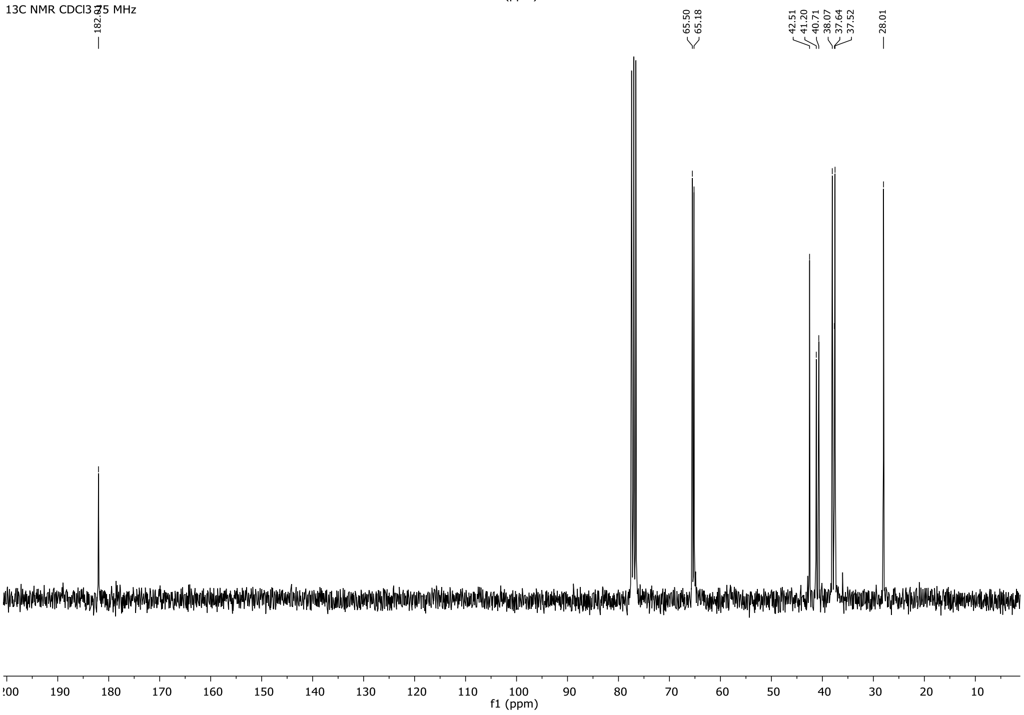

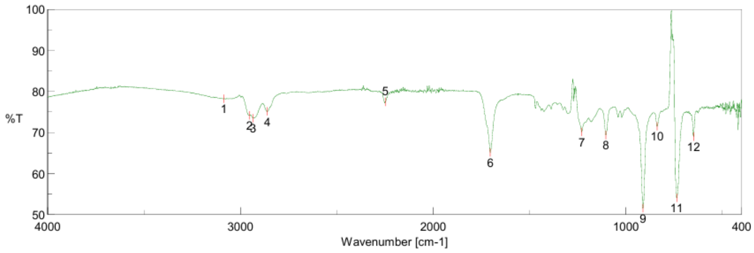

(8-oxaspiro[4.5]decan-2-yl)methanol (22a)

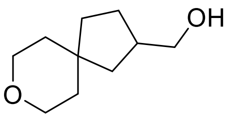

1H NMR CDCl3 300 MHz

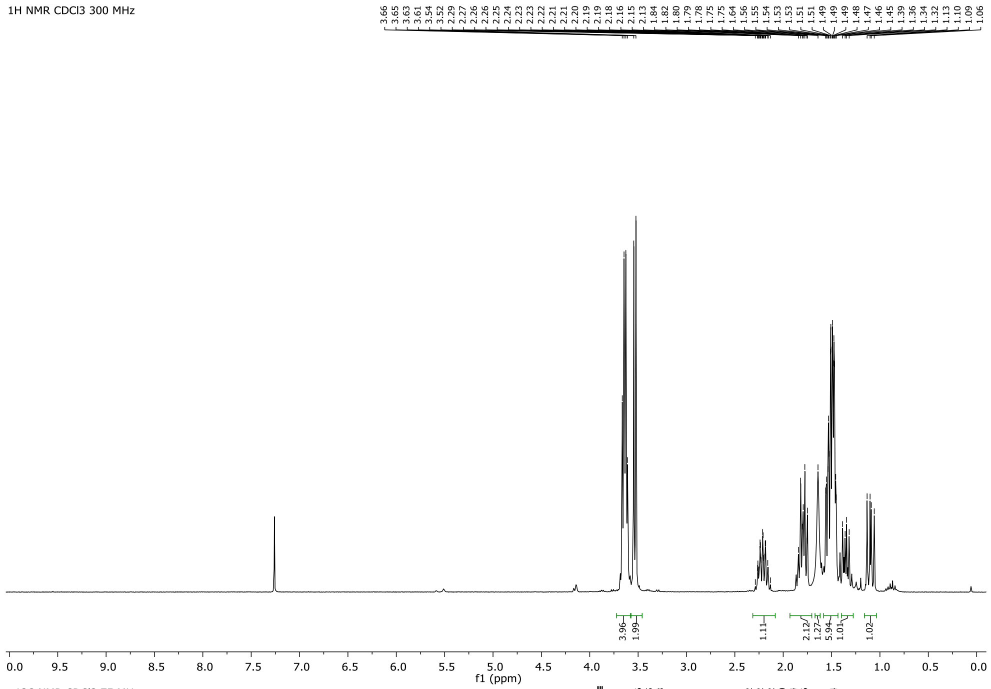

13C NMR CDCl3 75 MHz

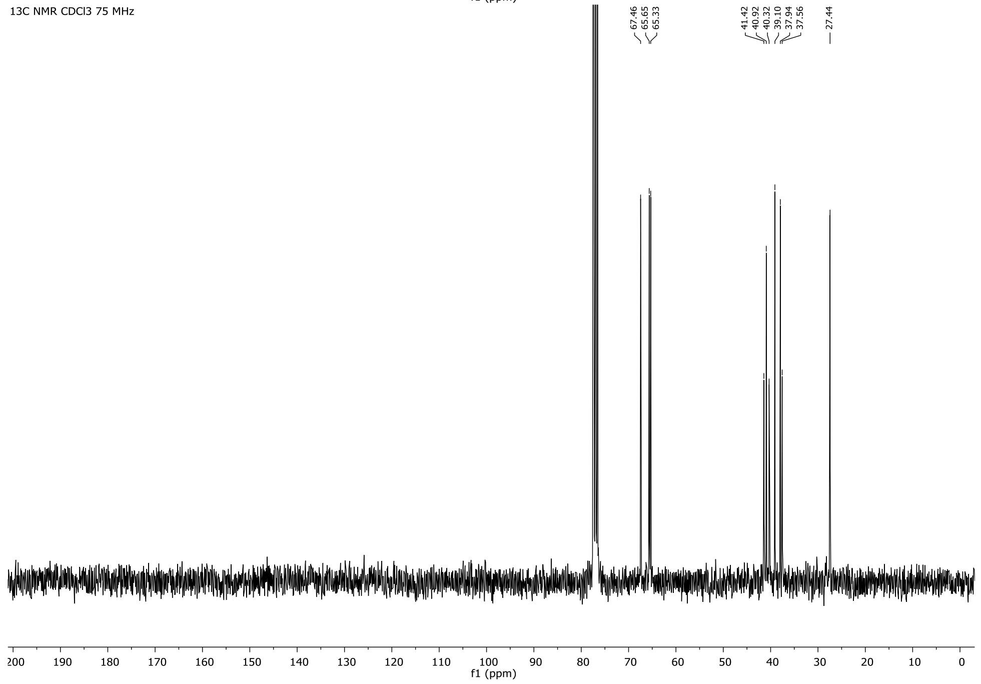

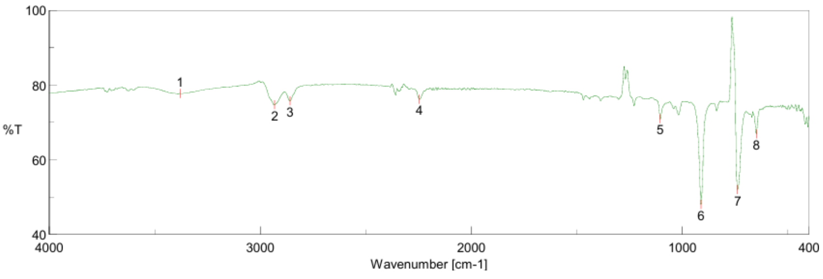

8-oxaspiro[4.5]decane-2-carboxamide (21a-NH[2])

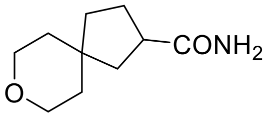

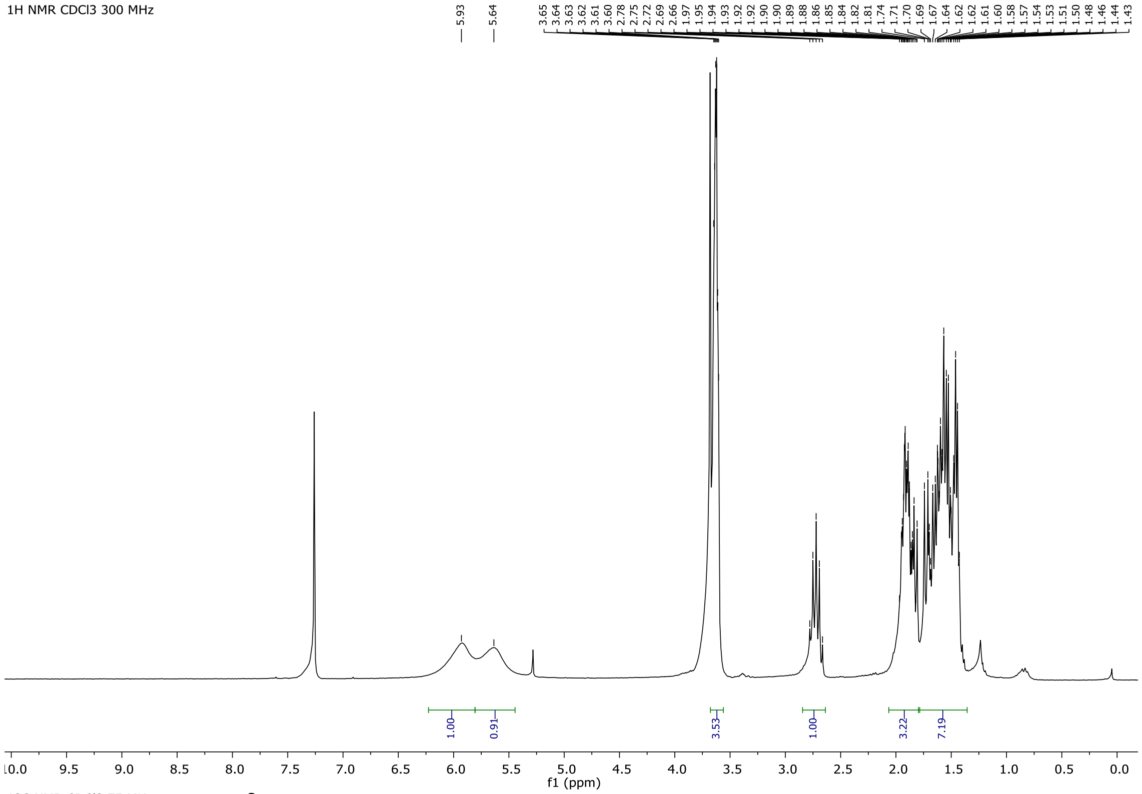

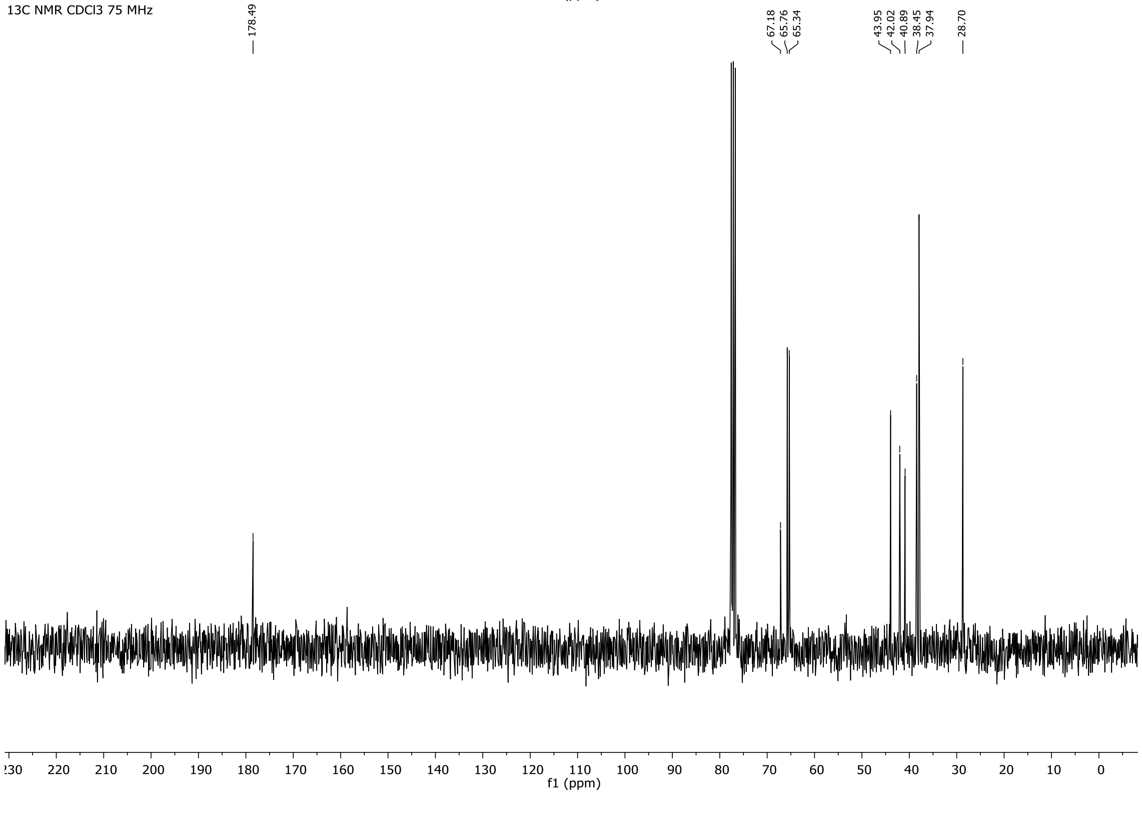

((8-oxaspiro[4.5]decan-2-yl)methyl)chloro-l5-azane (23a)

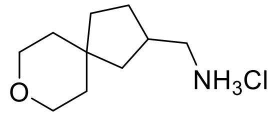

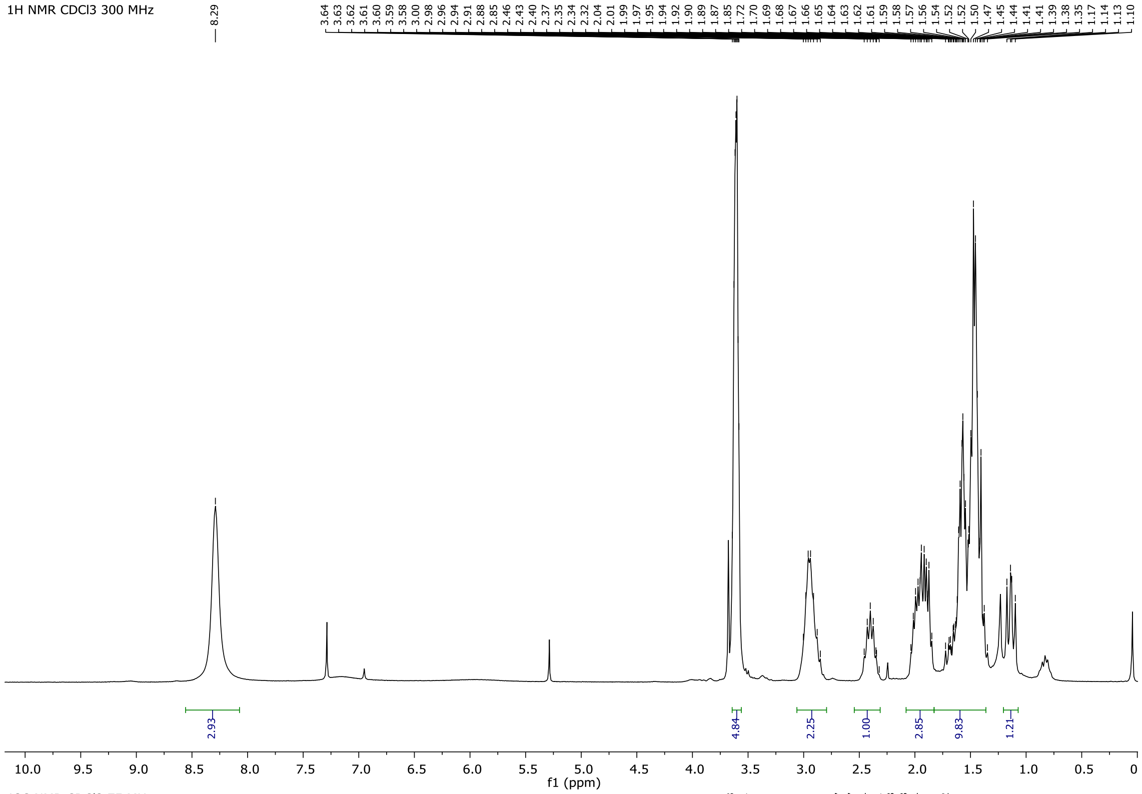

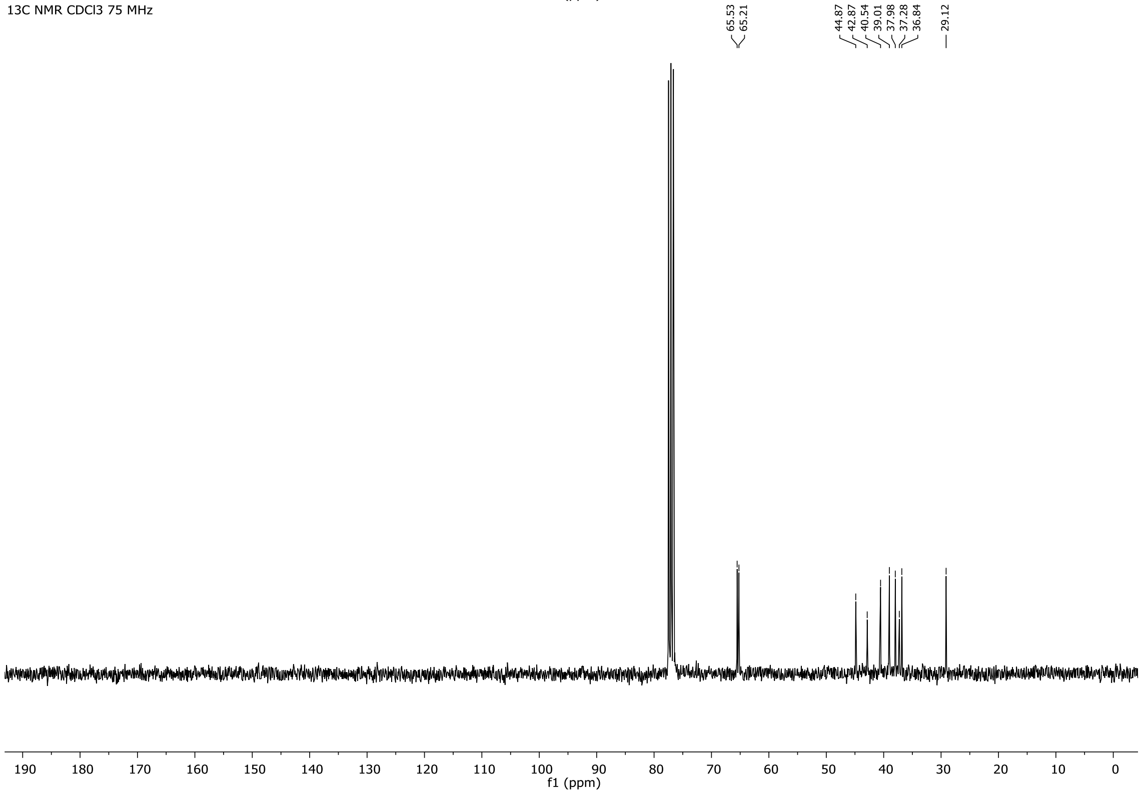

8-oxaspiro[4.5]decan-2-amine (24a-NH_2)

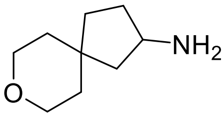

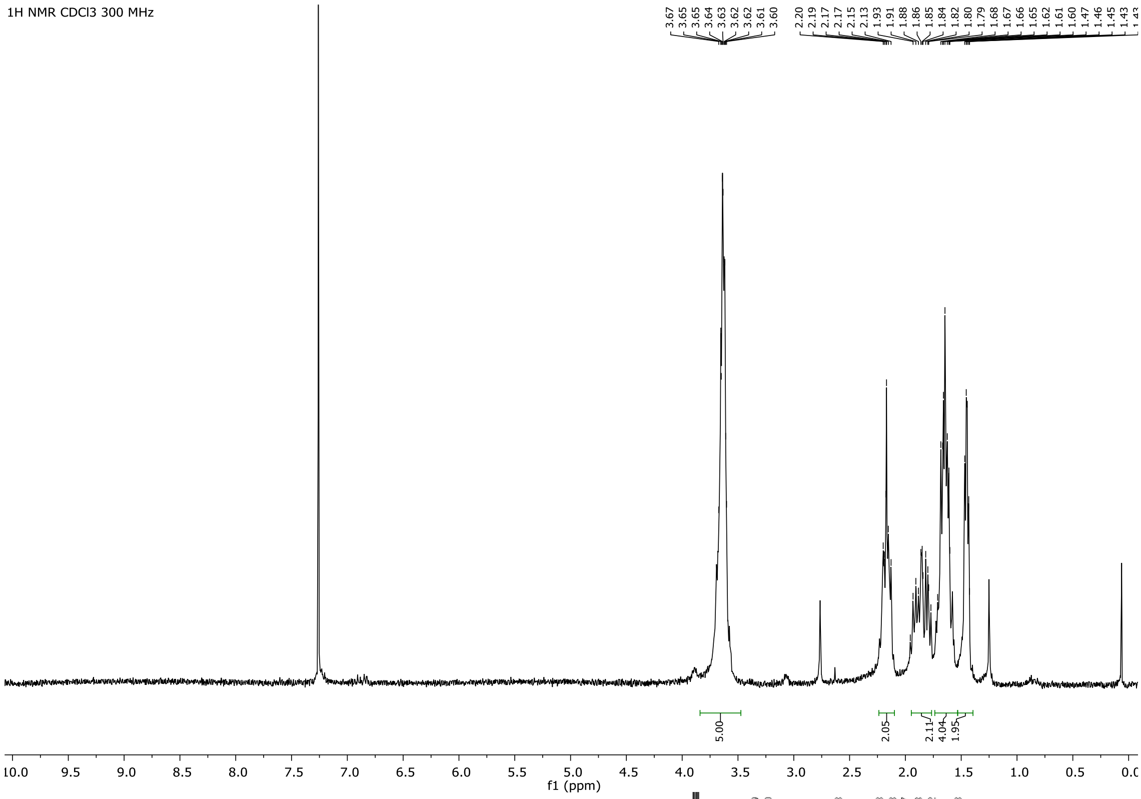

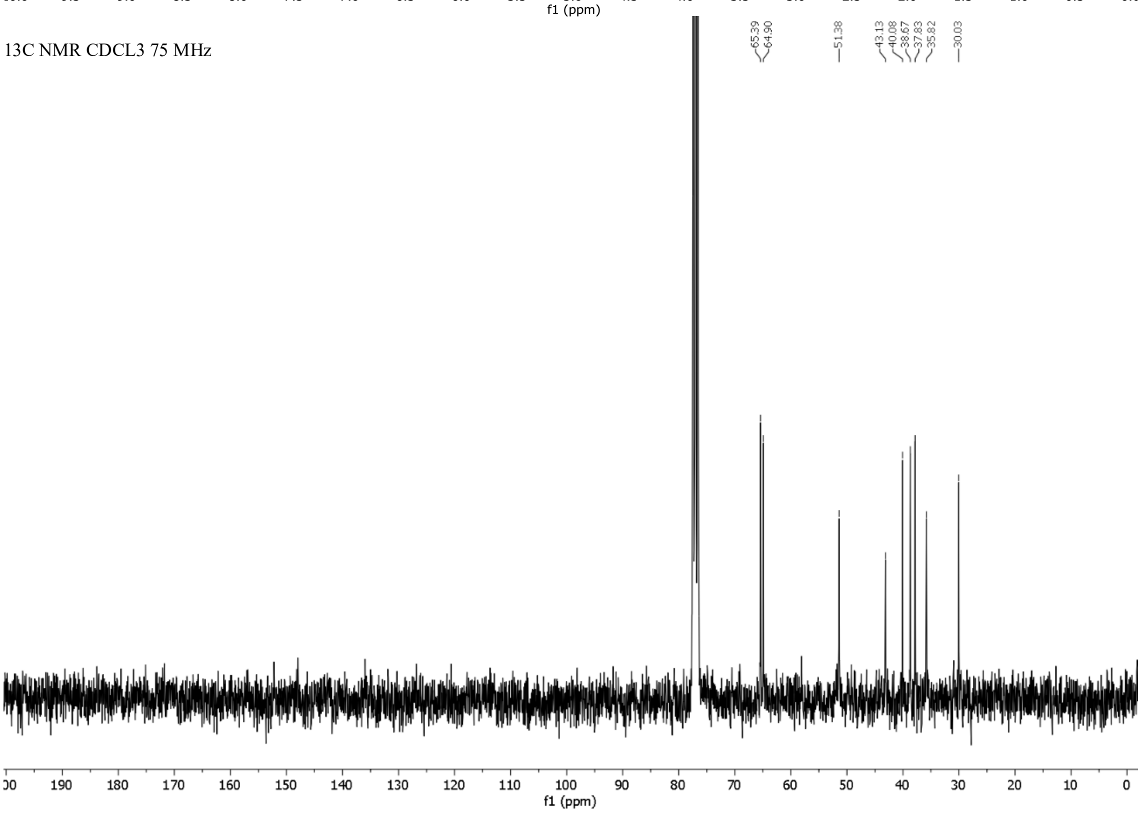

8-oxaspiro[4.5]decan-2-aminium chloride (24a)

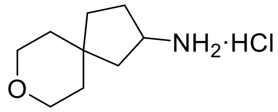

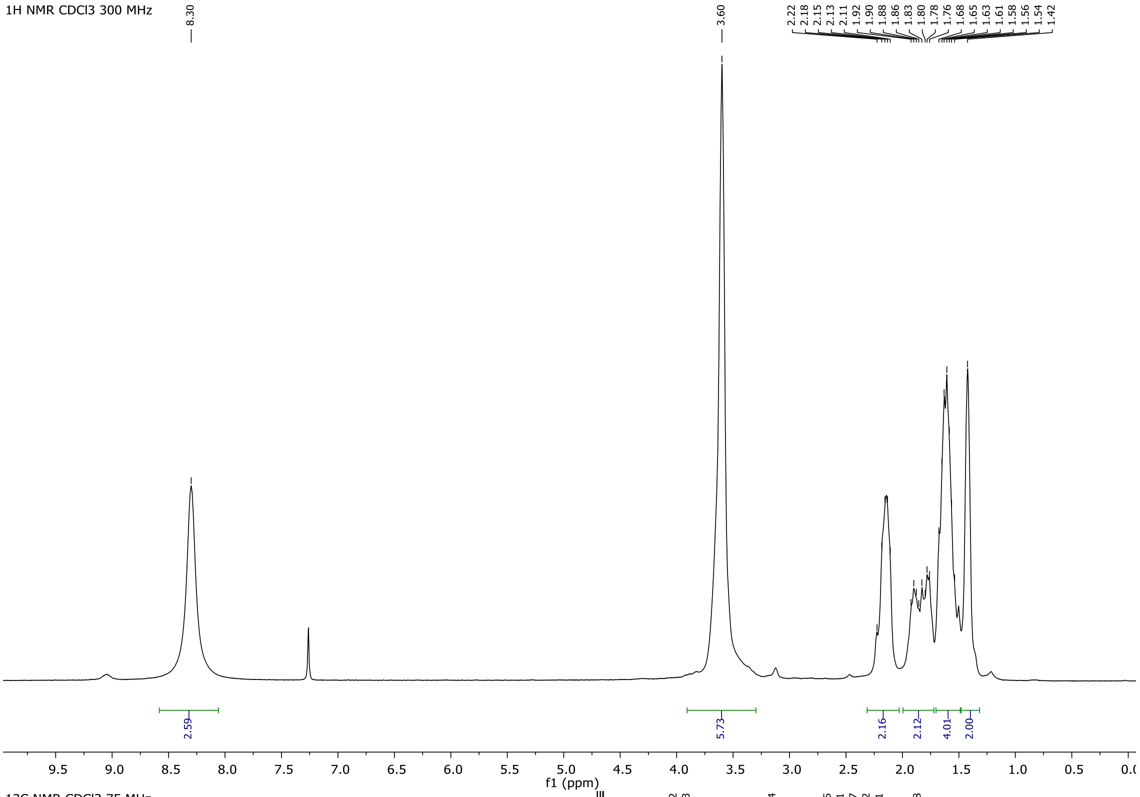

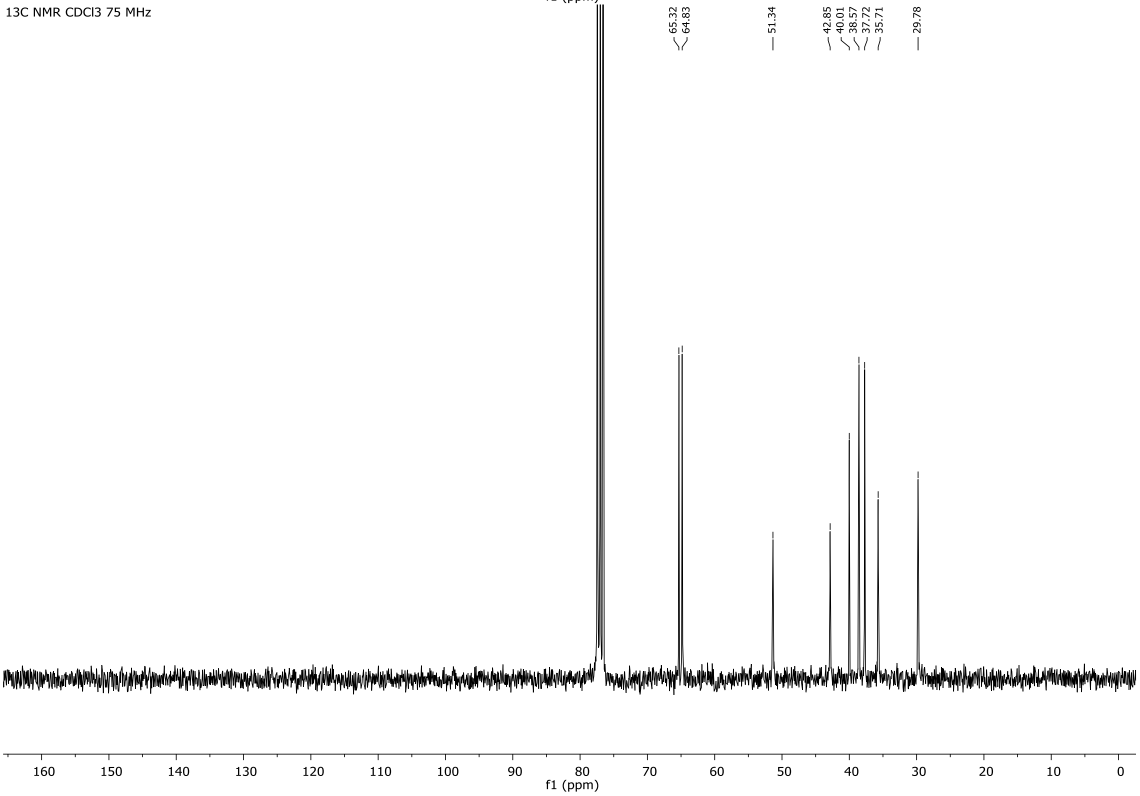

2-(8-oxaspiro[4.5]decan-2-yl)acetic acid (25a)

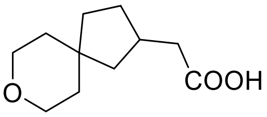

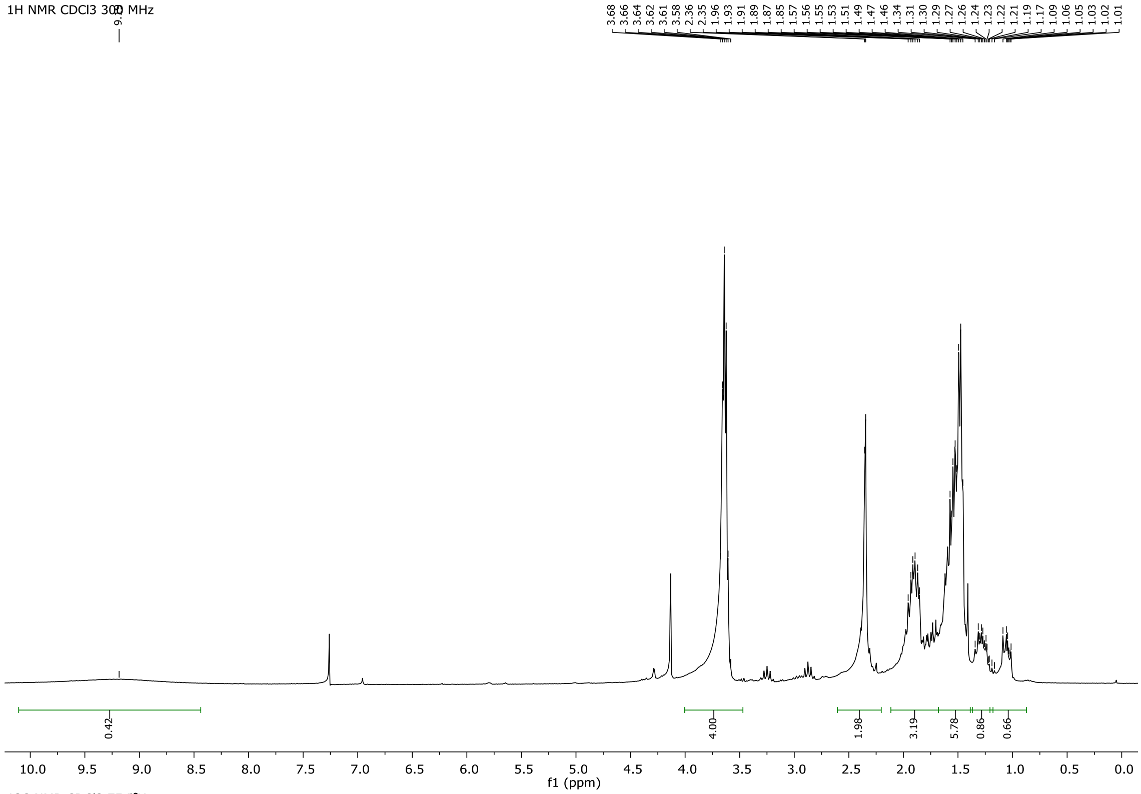

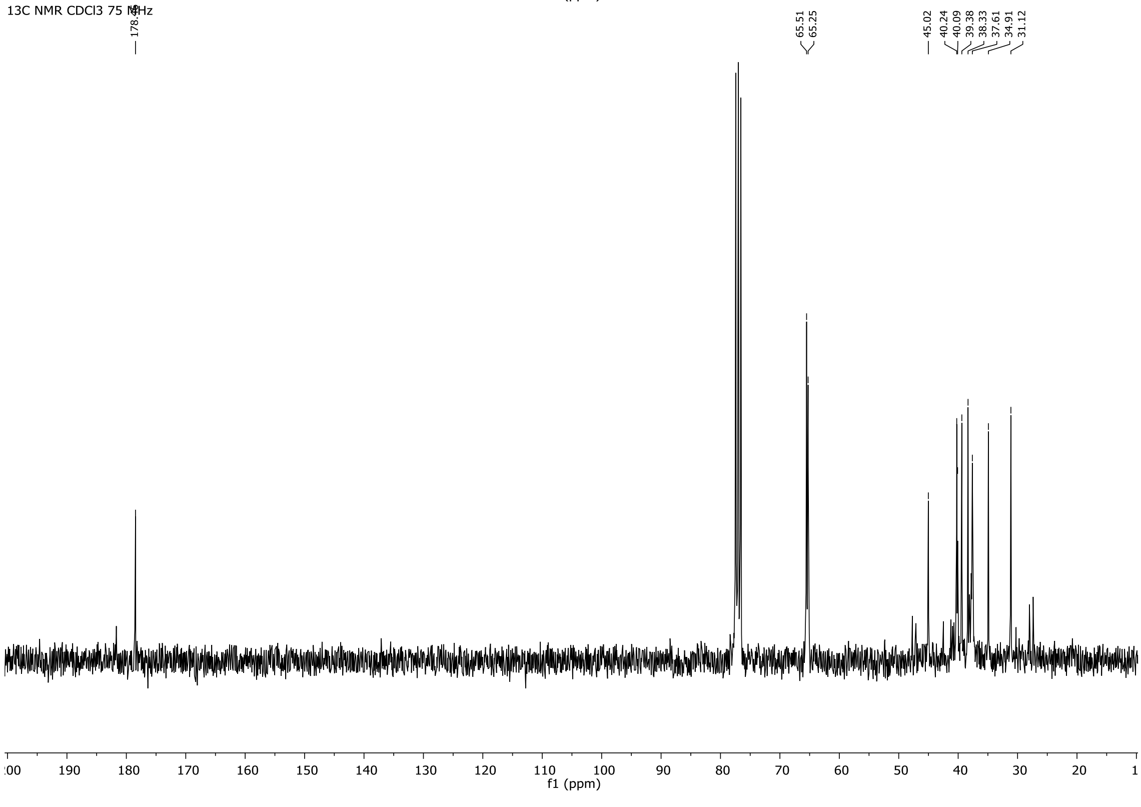

4,4,5,5-tetramethyl-2-(8-oxaspiro[4.5]dec-1-en-2-yl)-1,3,2-dioxaborolane (26a)

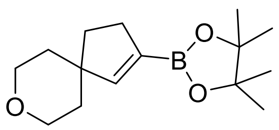

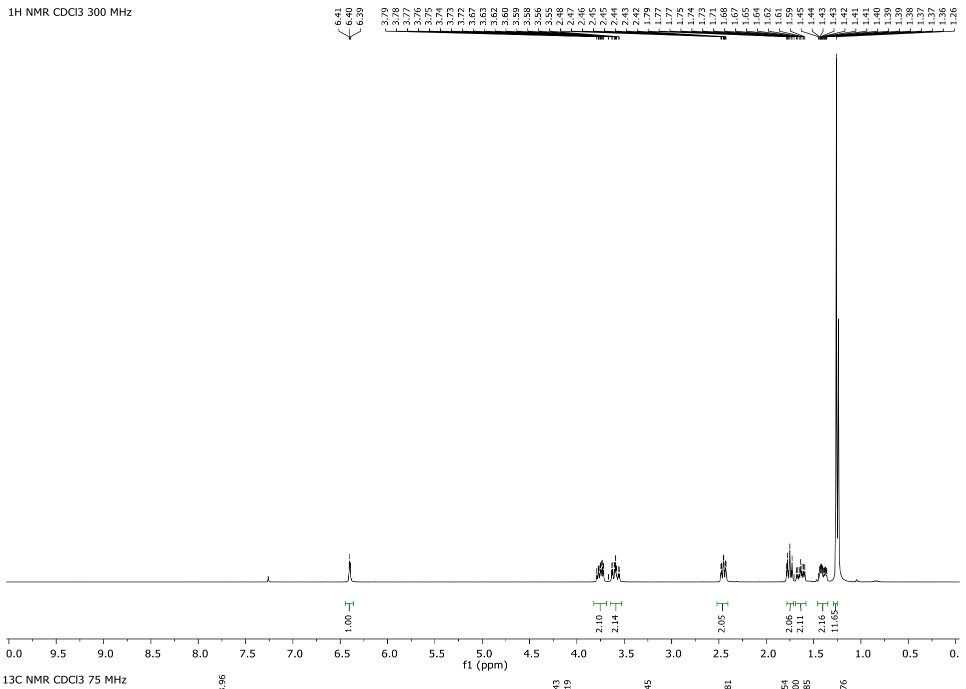

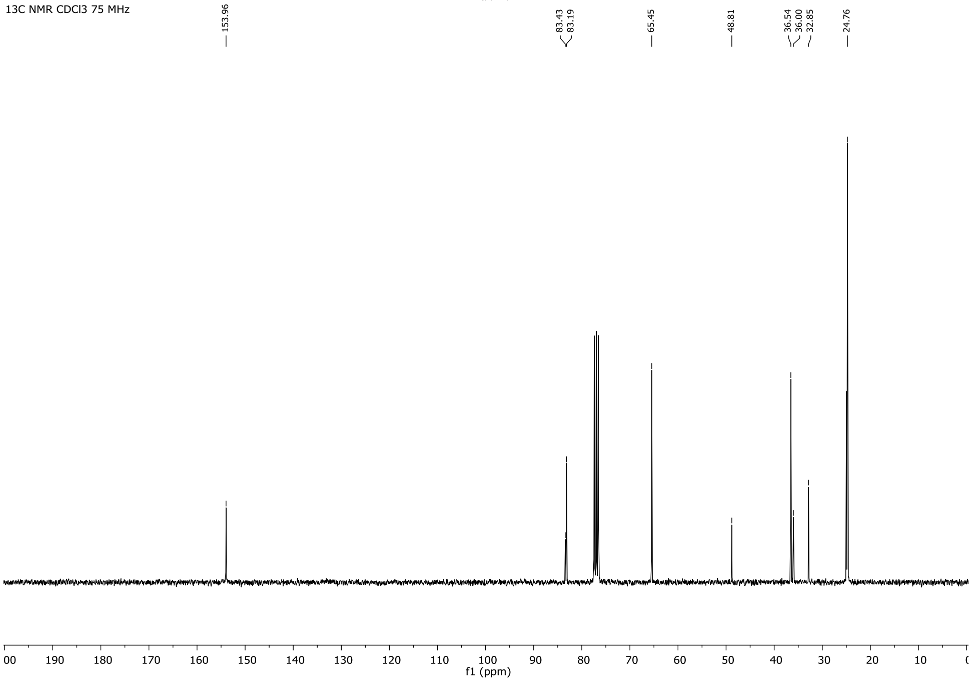

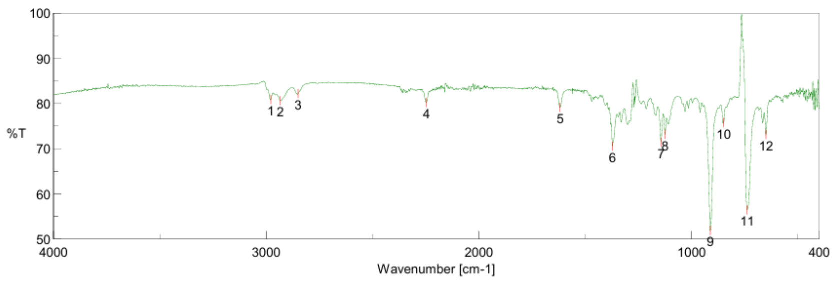

4,4,5,5-tetramethyl-2-(8-oxaspiro[4.5]decan-2-yl)-1,3,2-dioxaborolane (27a)

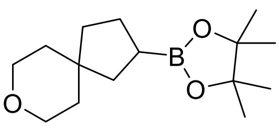

1H NMR CDCl3 300 MHz

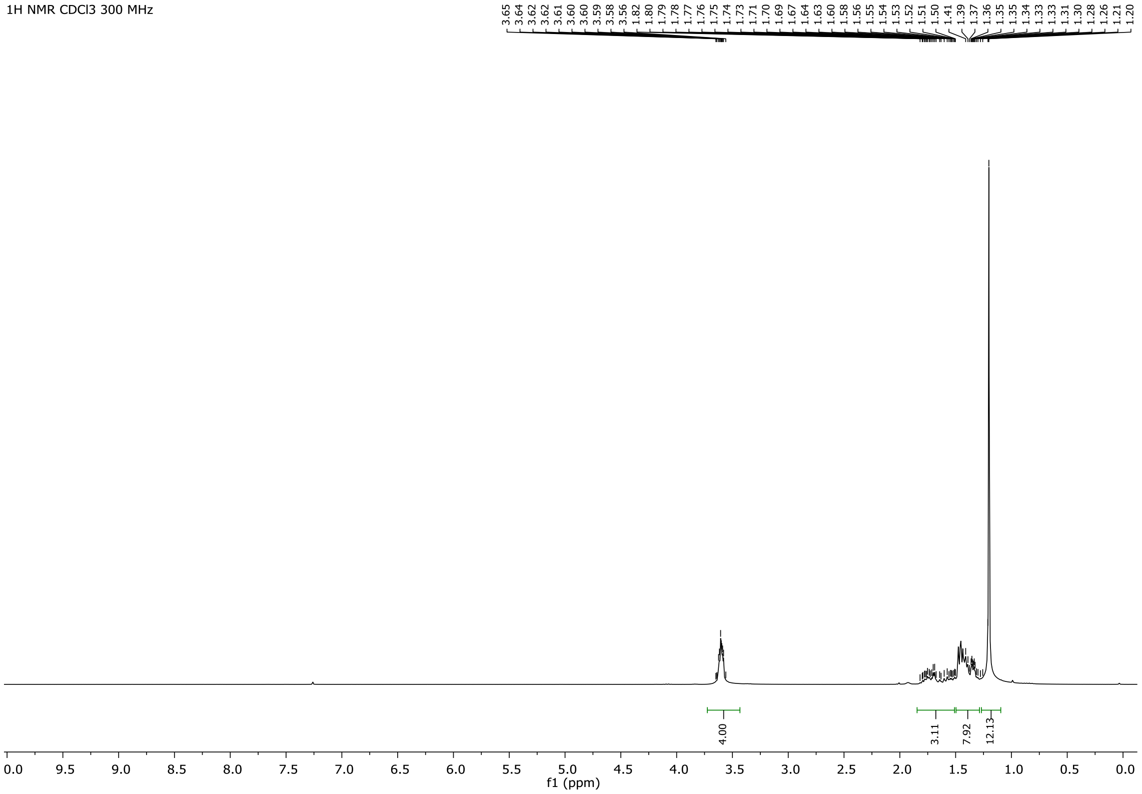

13C NMR CDCl3 75 MHz

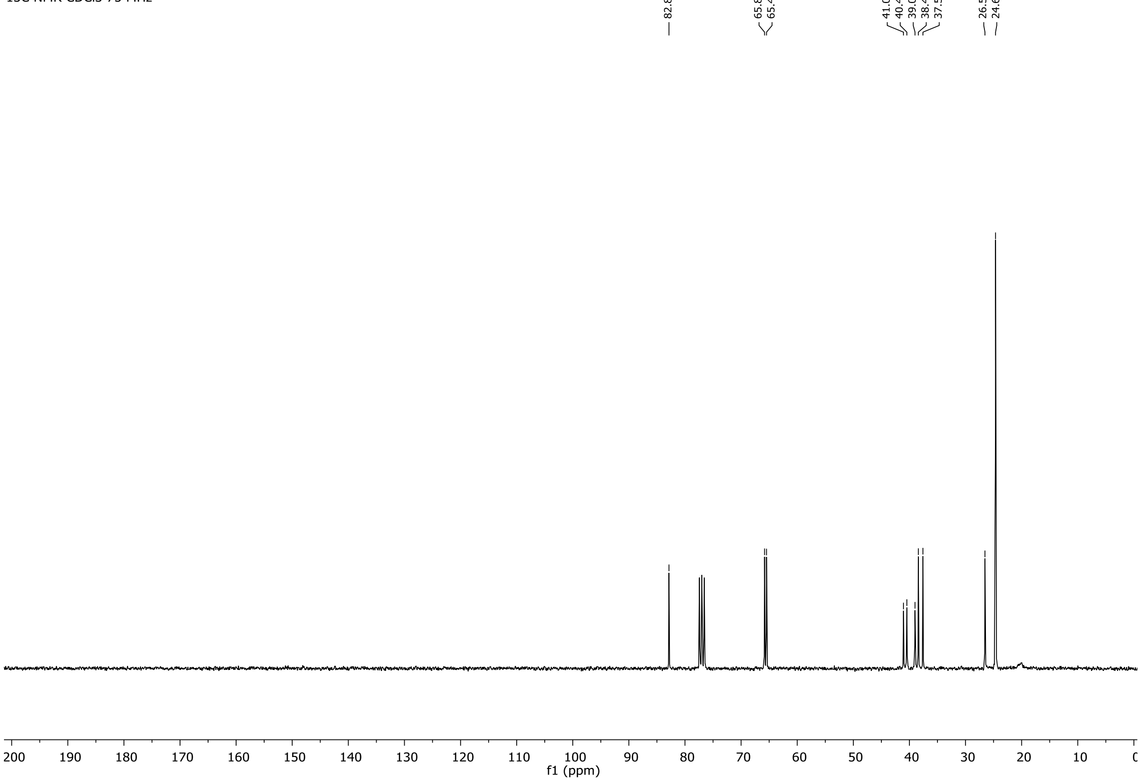

8-oxaspiro[4.5]decan-2-one (28a)

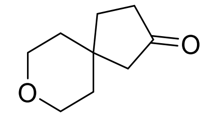

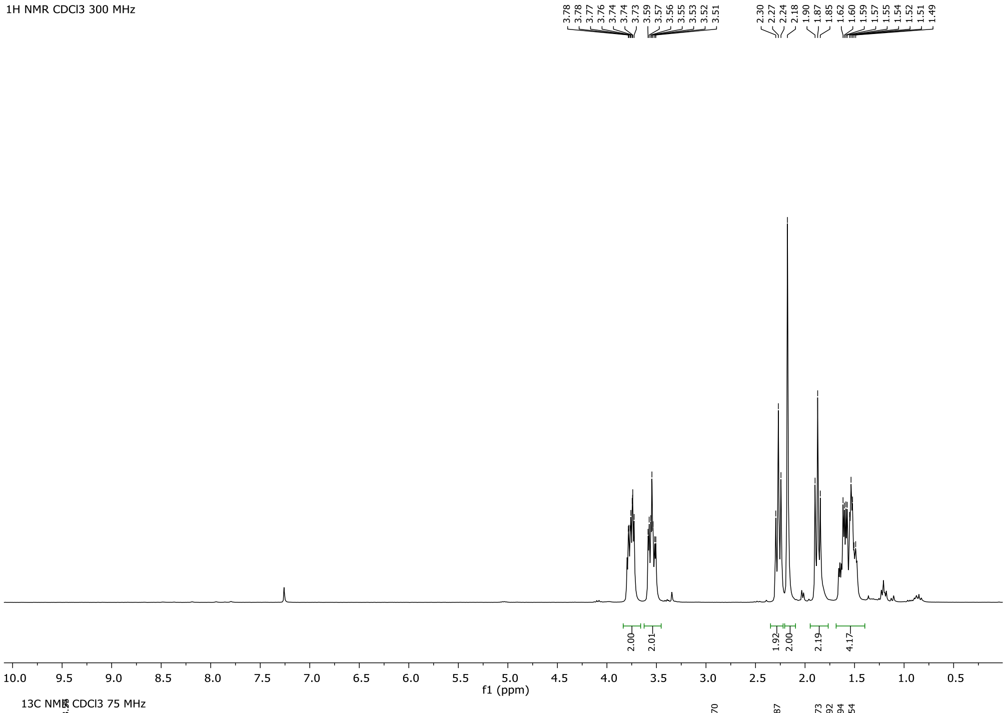

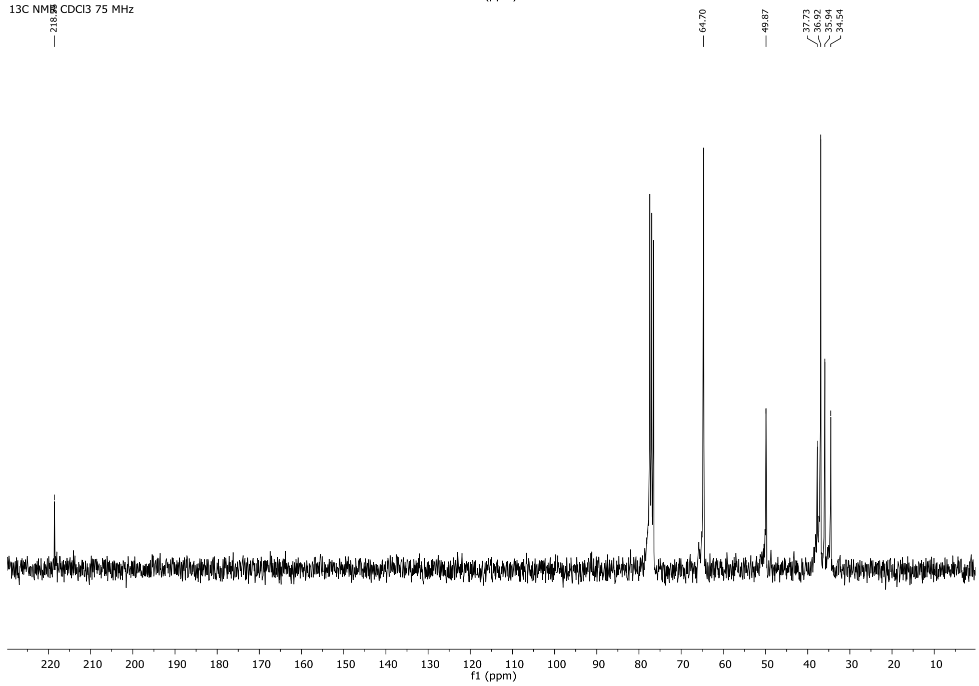

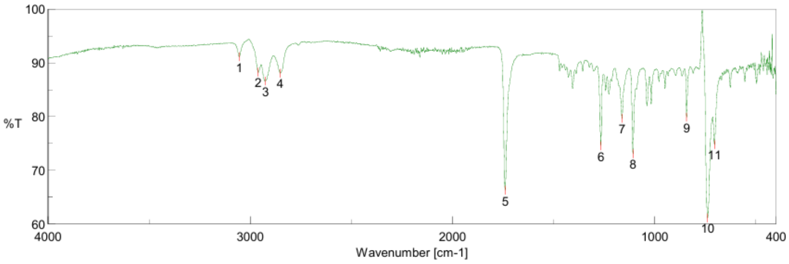

2-(trifluoromethyl)-8-oxaspiro[4.5]decan-2-ol (29a)

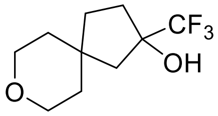

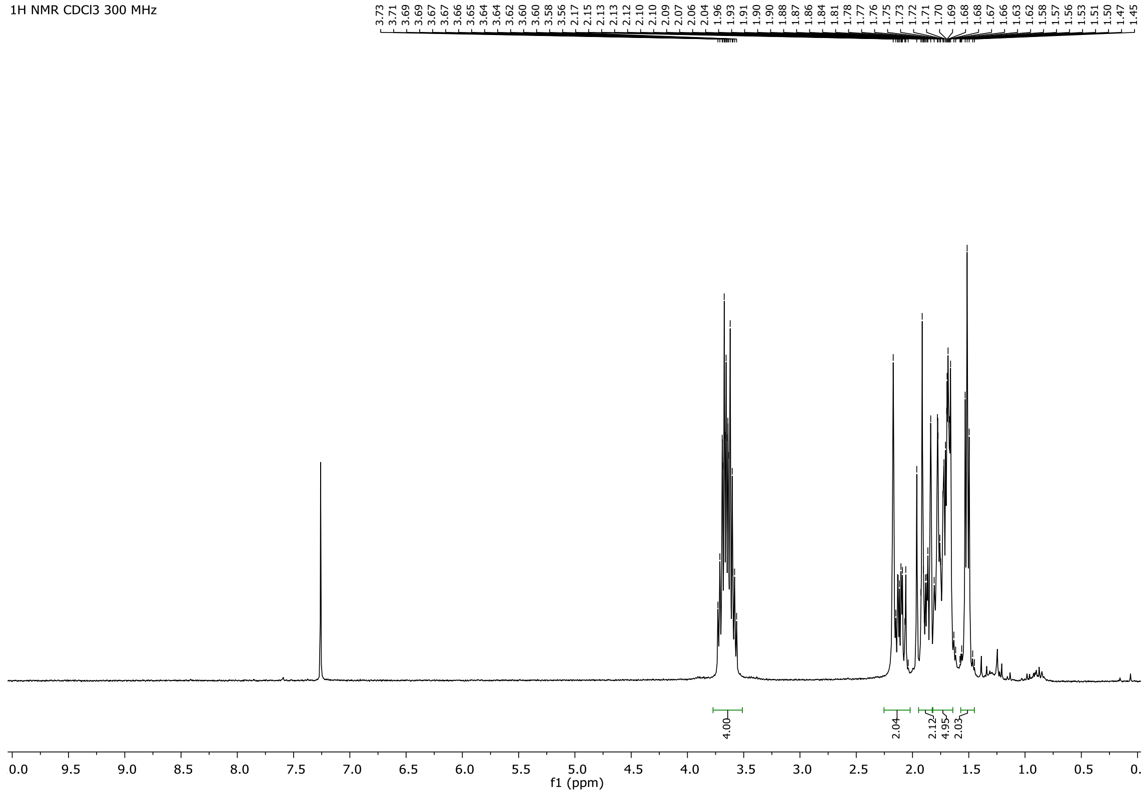

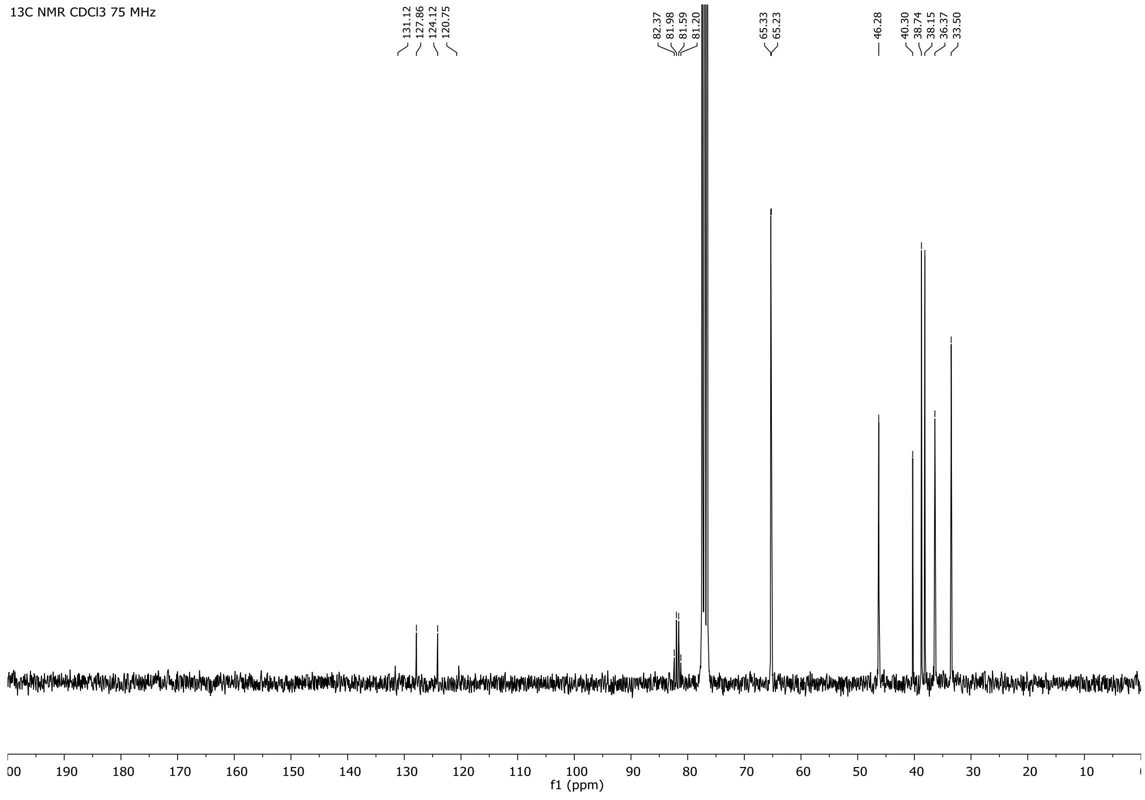

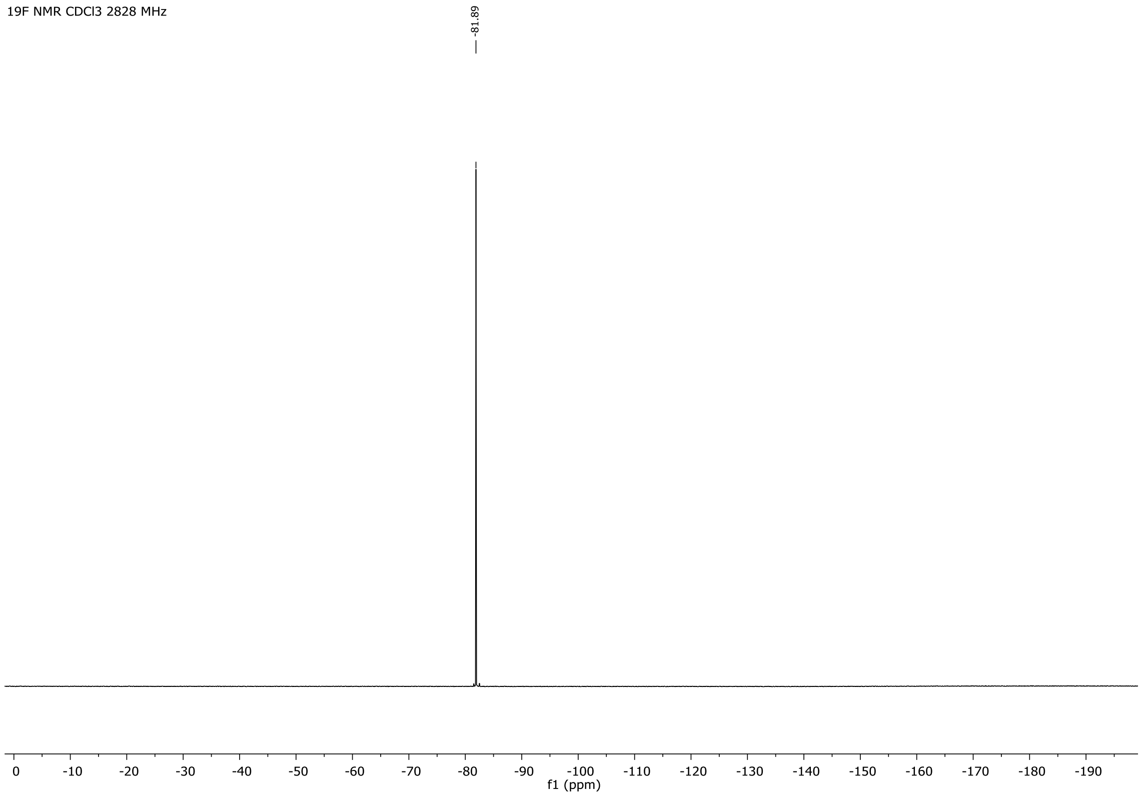
19F NMR CDCl3 2828 MHz
-81.89
0
-10
-20
-30
-40
-50
-60
-70
-80
-90
-100
-110
-120
-130
-140
-150
-160
-170
-180
-190
f1 (ppm)

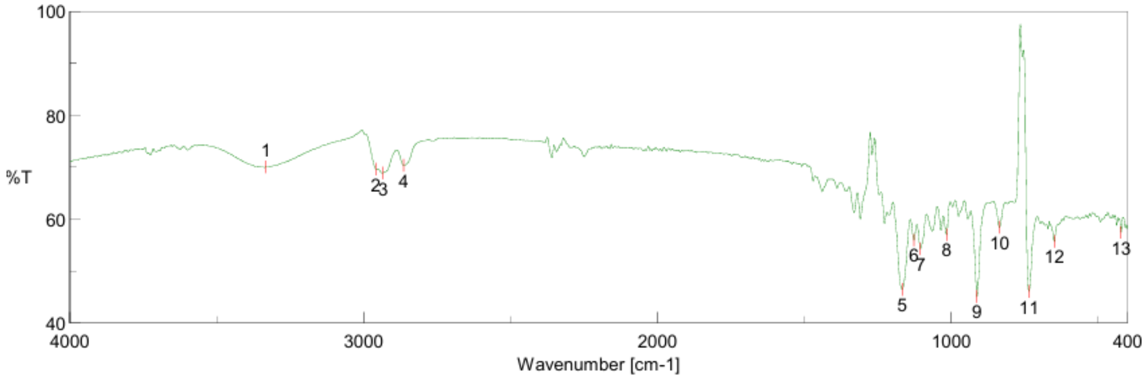
100
80
60
40
%T
1
2
3
4
5
6
7
8
9
10
11
12
13
4000
3000
2000
1000
400
Wavenumber [cm-1]

2-bromo-8-azaspiro[4.5]dec-1-ene (7-NH)

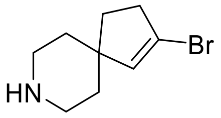

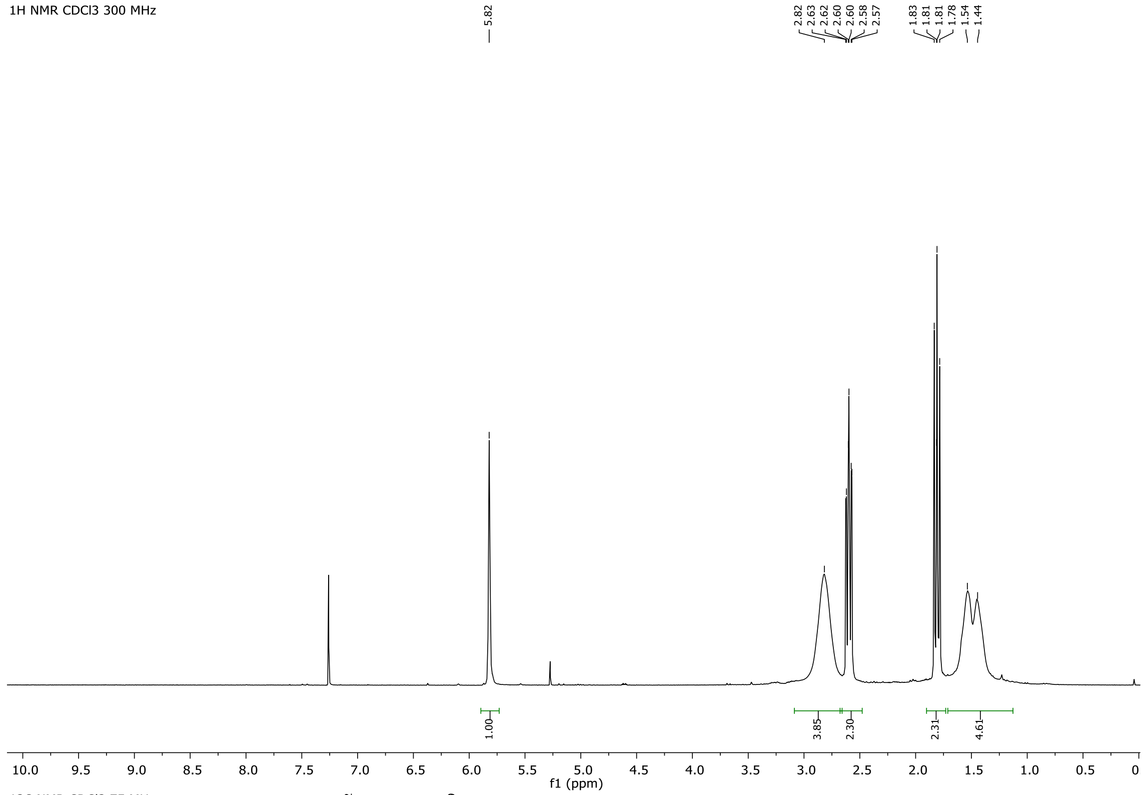

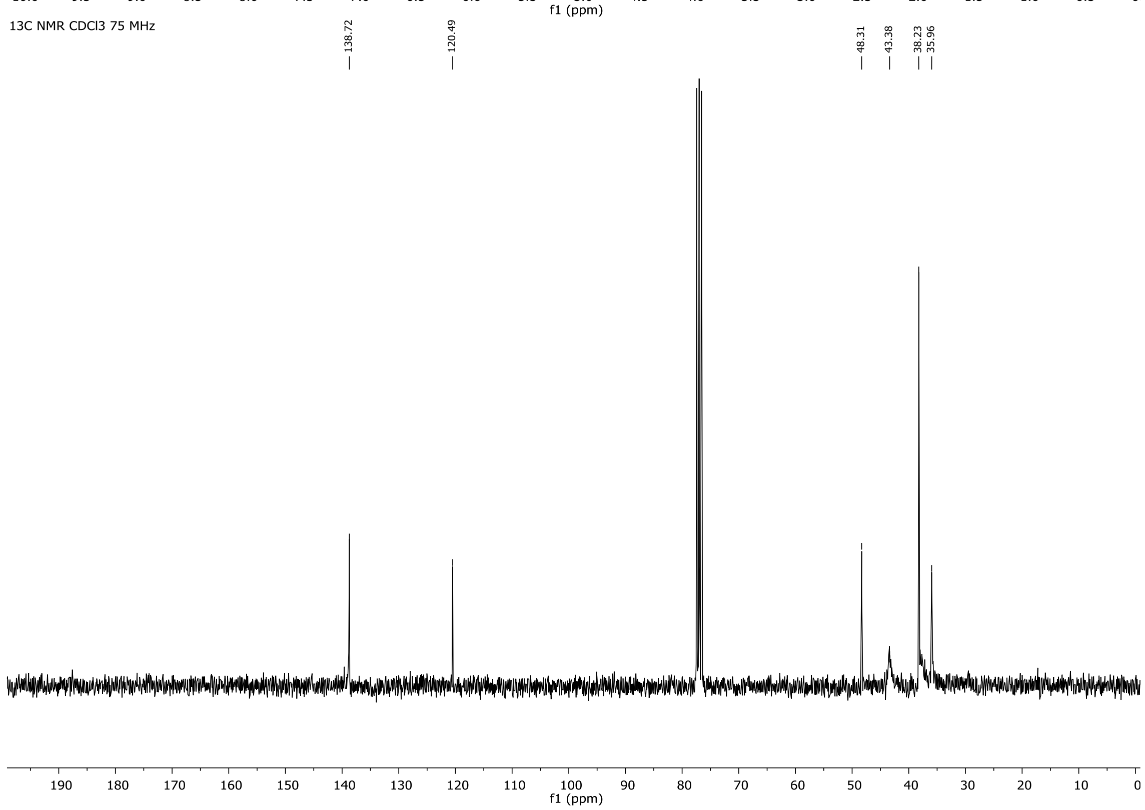

tert-butyl 2-bromo-8-azaspiro[4.5]dec-1-ene-8-carboxylate (20a)

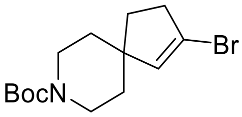

1H NMR CDCl3 300 MHz

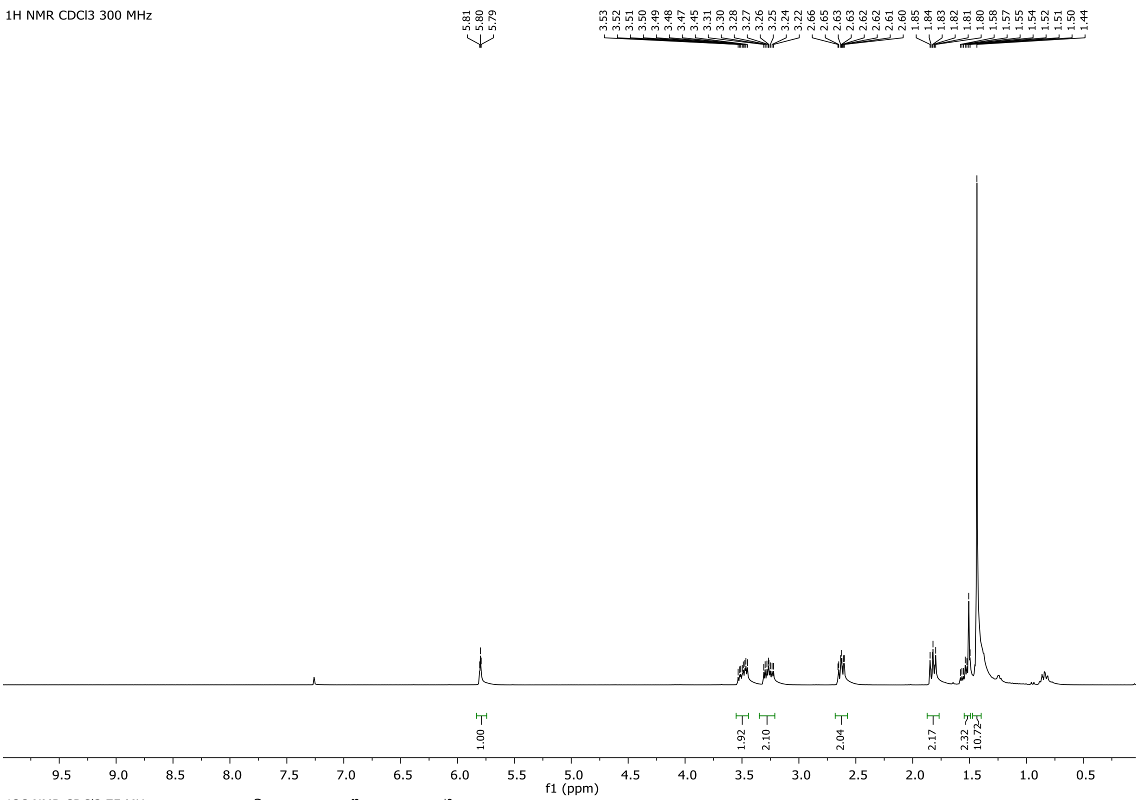

13C NMR CDCl3 75 MHz

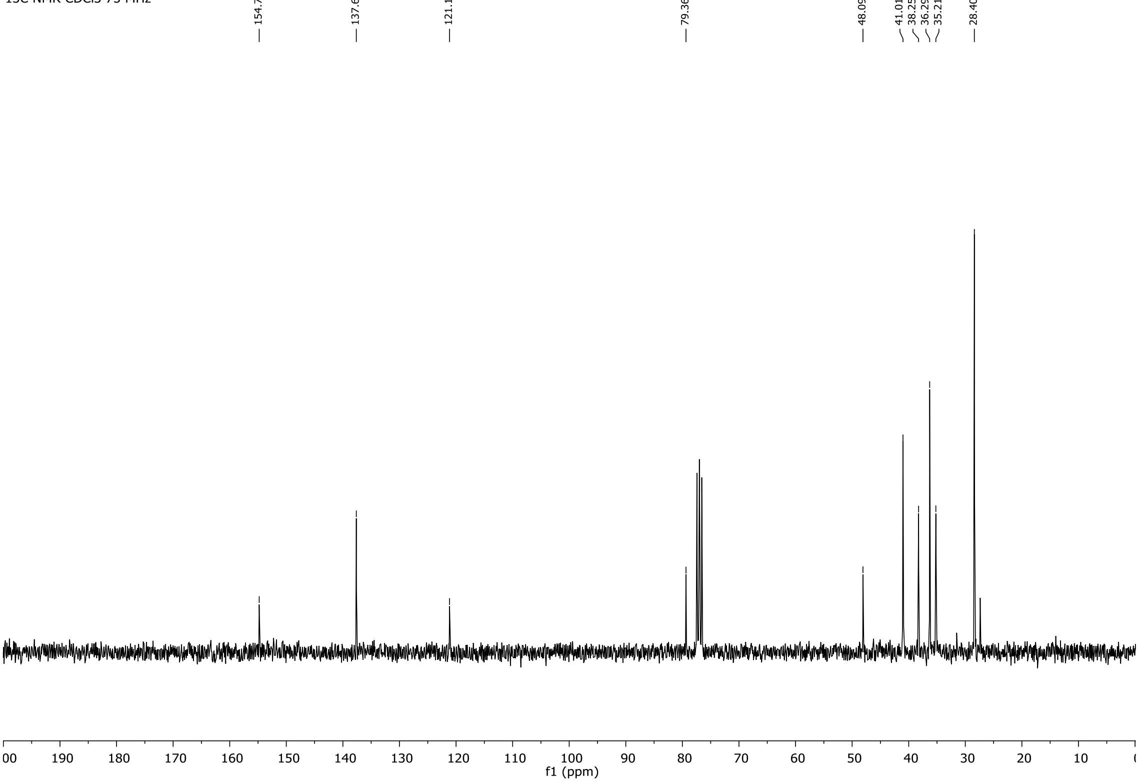

8-(tert-butoxycarbonyl)-8-azaspiro[4.5]dec-1-ene-2-carboxylic acid (21b-C=C)

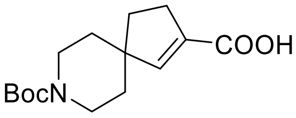

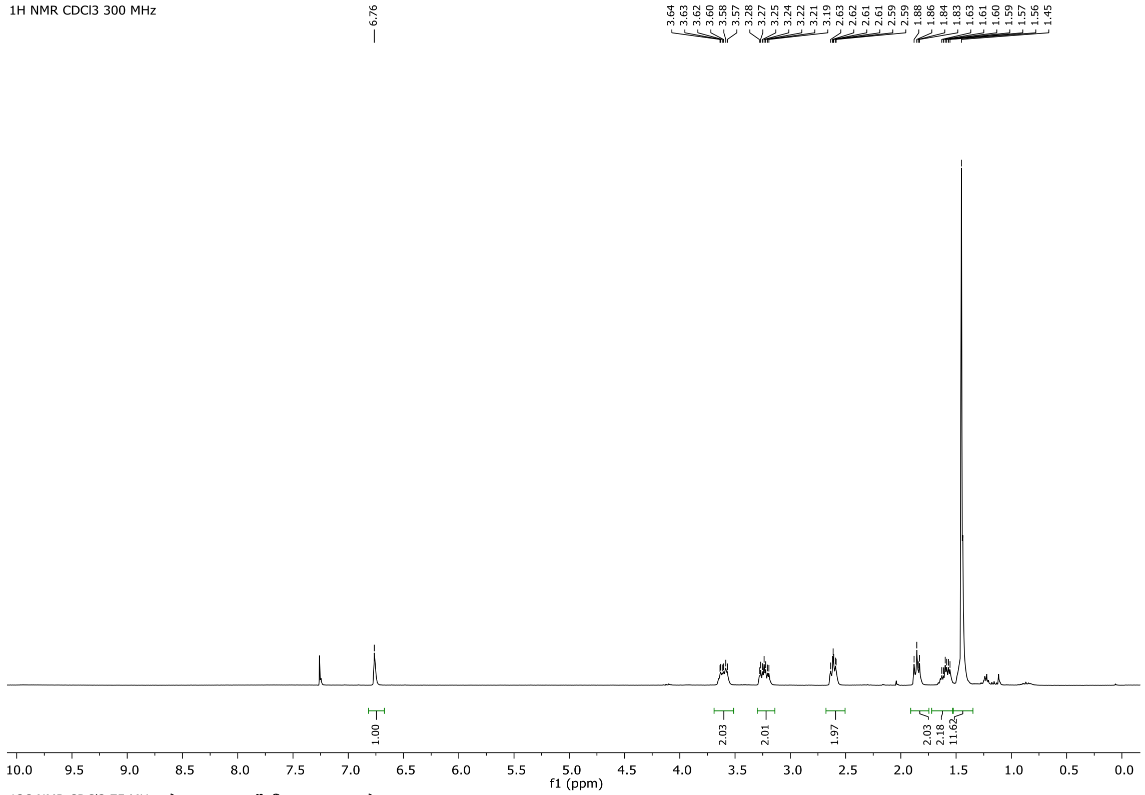

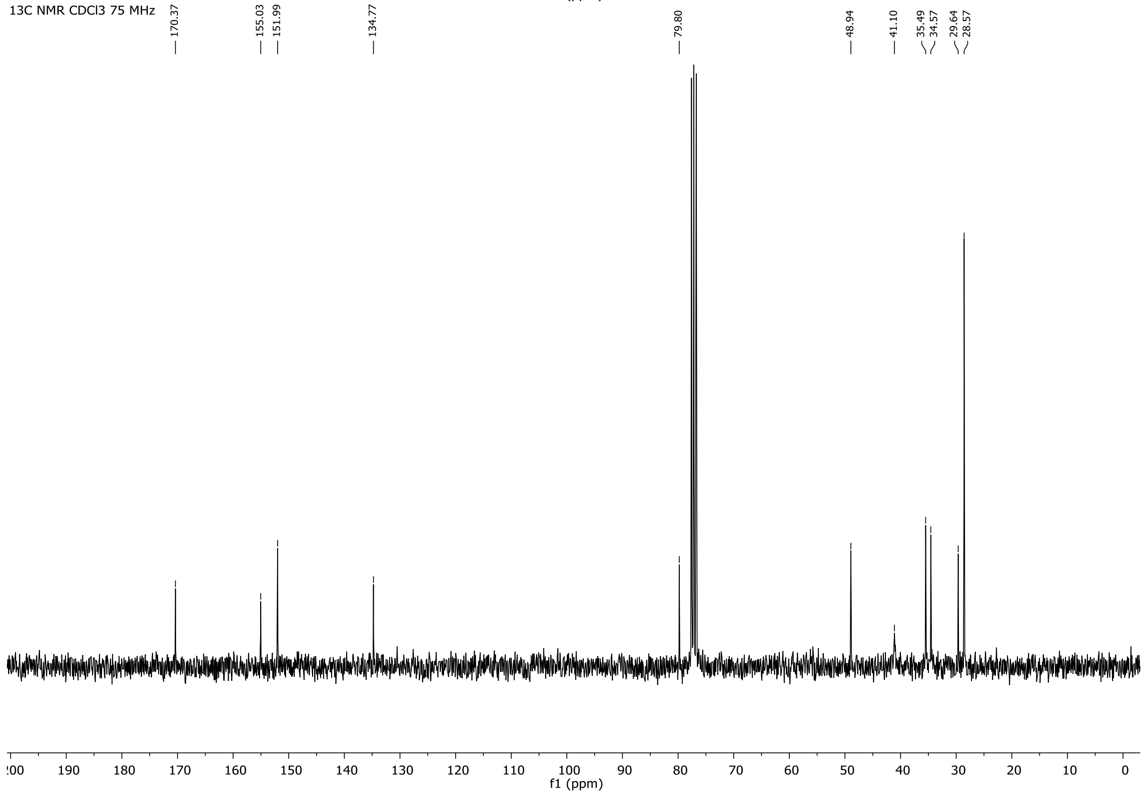

8-(tert-butoxycarbonyl)-8-azaspiro[4.5]decane-2-carboxylic acid (21b)

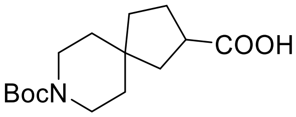

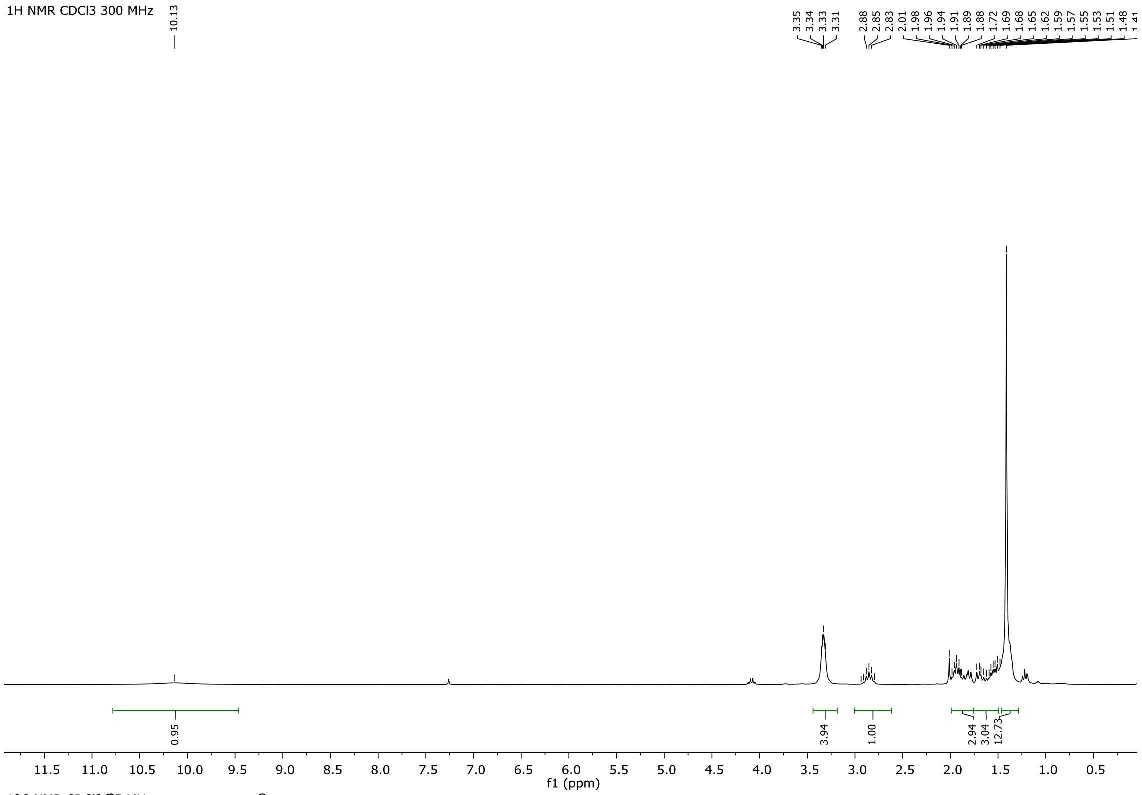

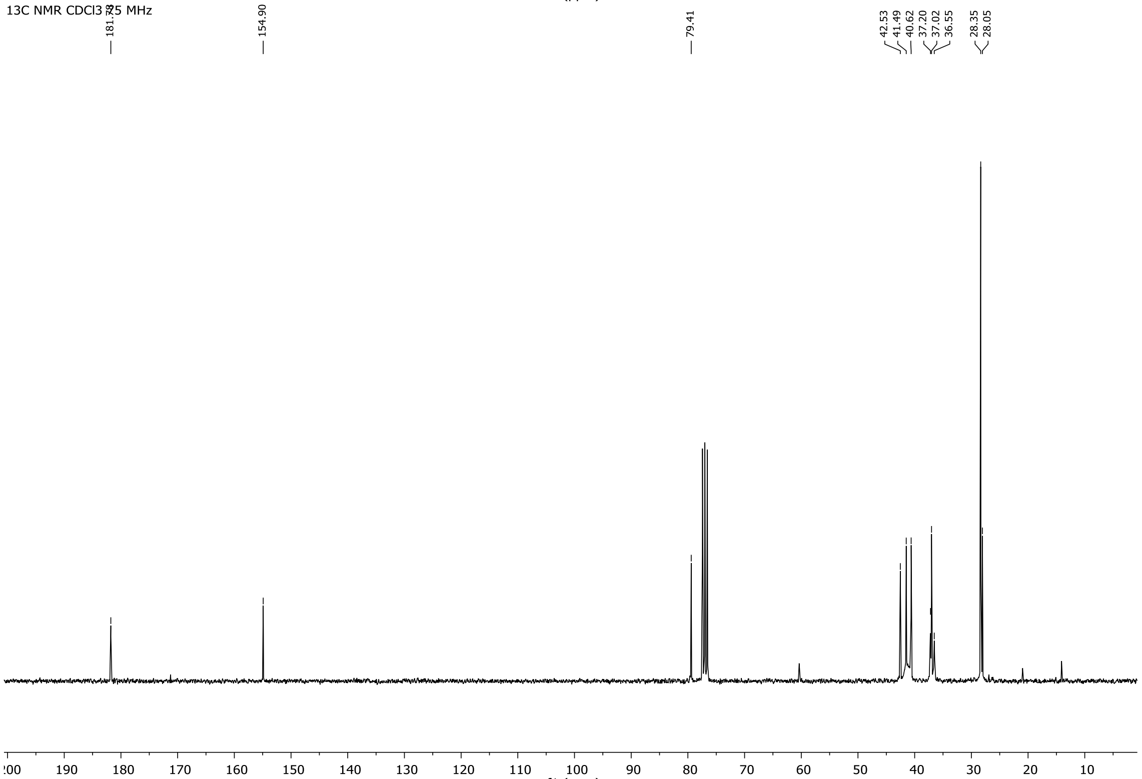

tert-butyl 2-carbamoyl-8-azaspiro[4.5]decane-8-carboxylate (21b-NH_2)

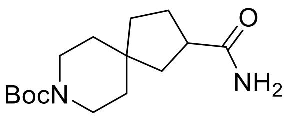

1H NMR CDCl3 300 MHz

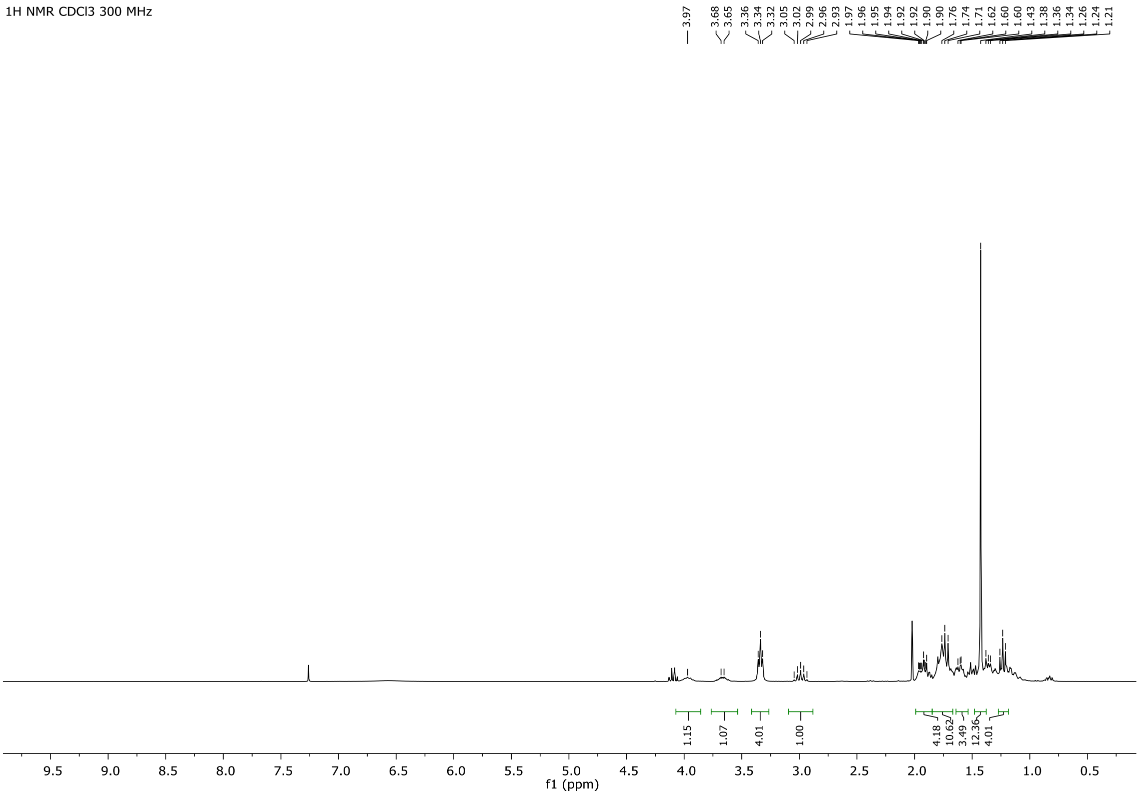

tert-butyl 2-(aminomethyl)-8-azaspiro[4.5]decane-8-carboxylate (23b)

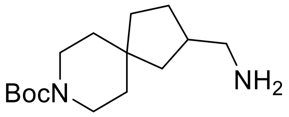

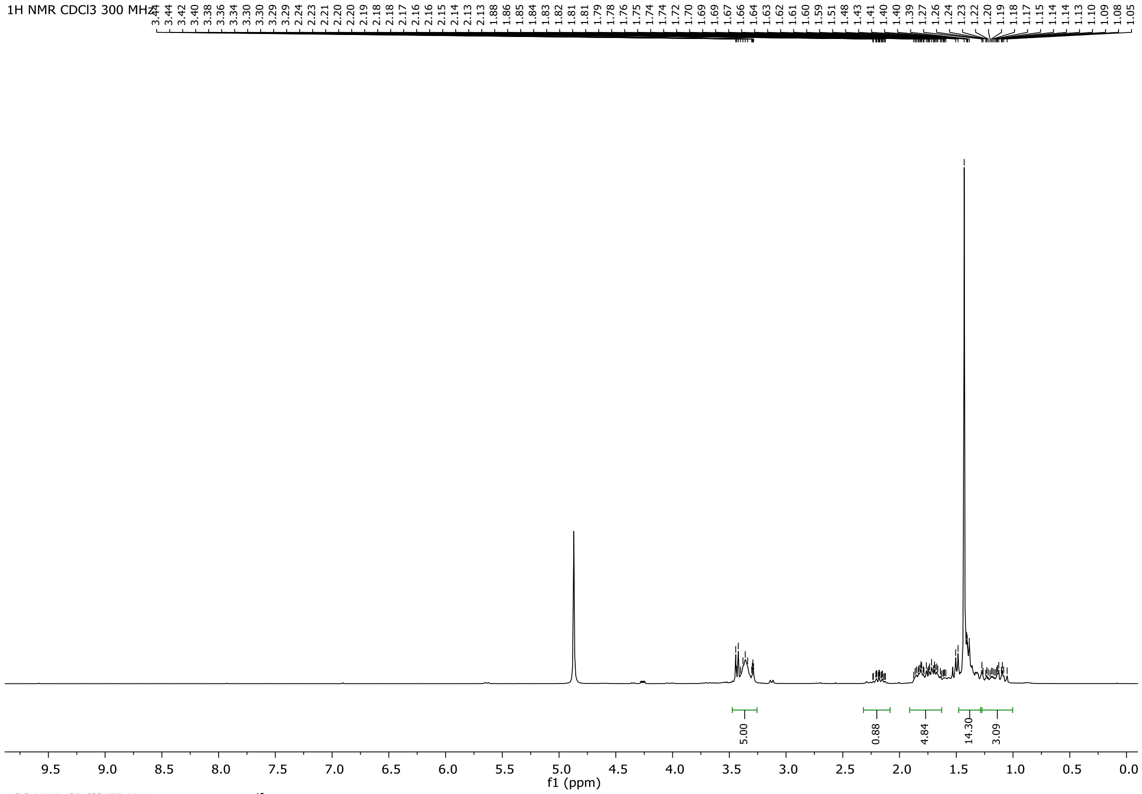

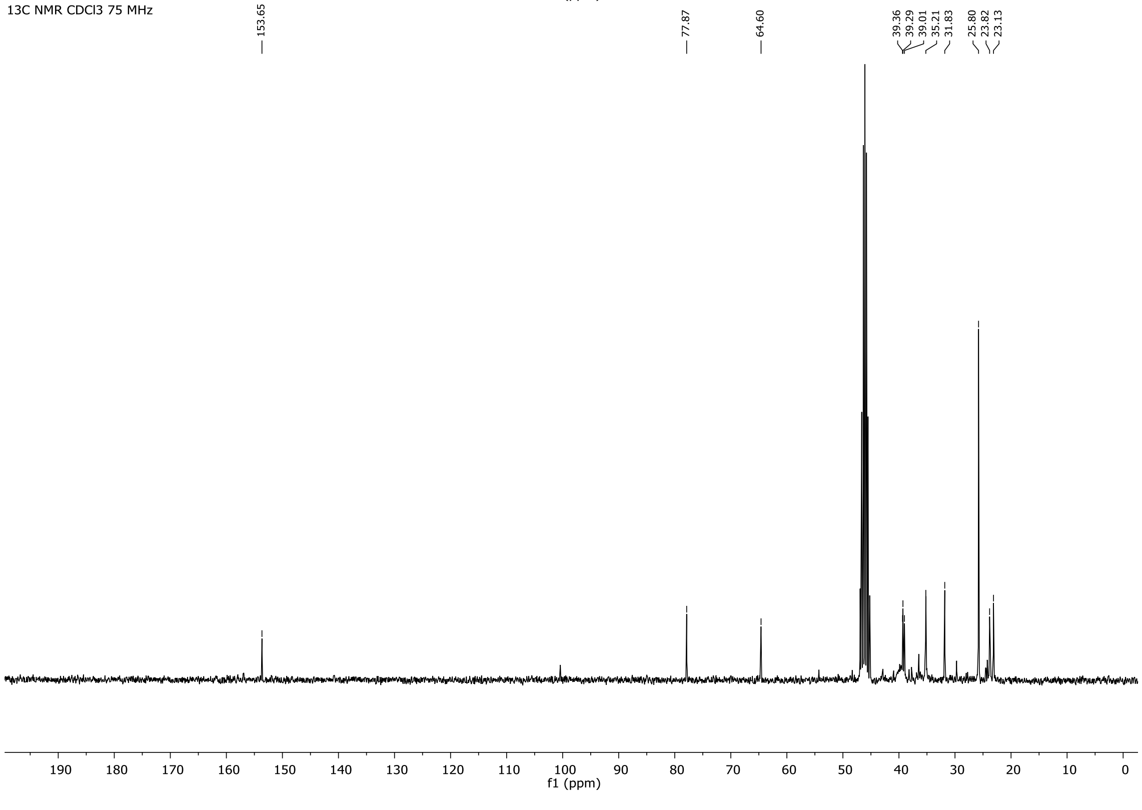

tert-butyl 2-(((benzyloxy)carbonyl)amino)-8-azaspiro[4.5]decane-8-carboxylate (24b-int)

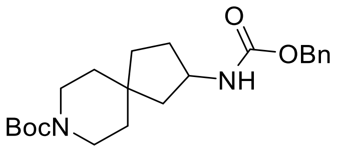

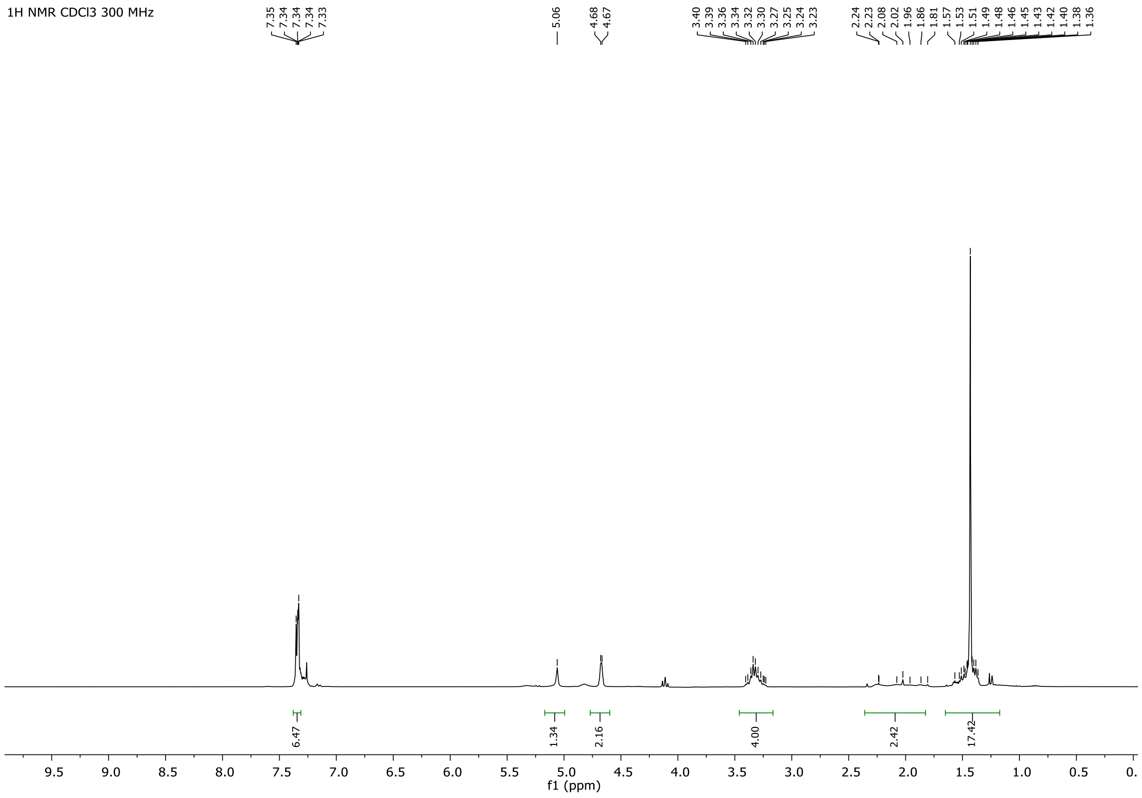

tert-butyl 2-amino-8-azaspiro[4.5]decane-8-carboxylate (24b)

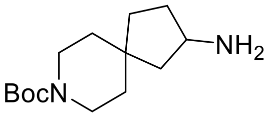

1H NMR CDCl3 300 MHz

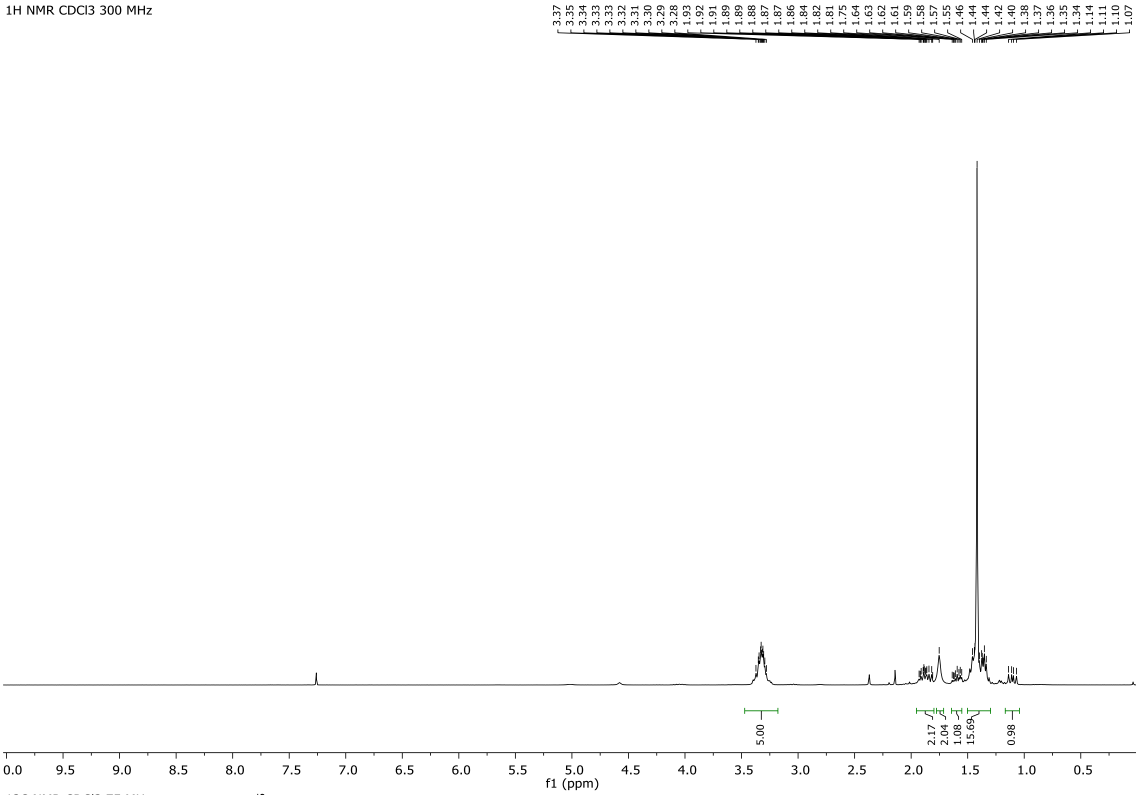

13C NMR CDCl3 75 MHz

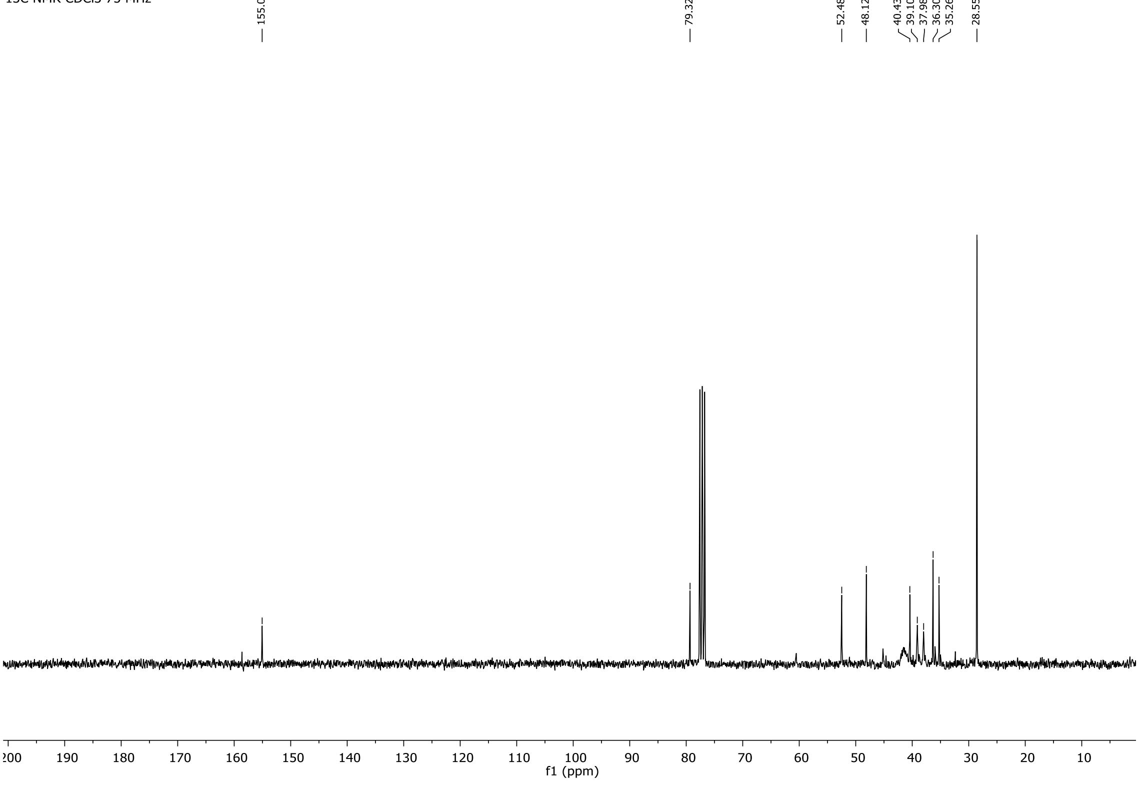

2,2,2-trifluoro-1-(2-(4,4,5,5-tetramethyl-1,3,2-dioxaborolan-2-yl)-8-azaspiro[4.5]dec-1-en-8-yl)ethan-1-one (26b)

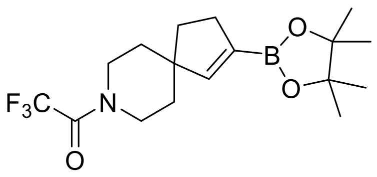

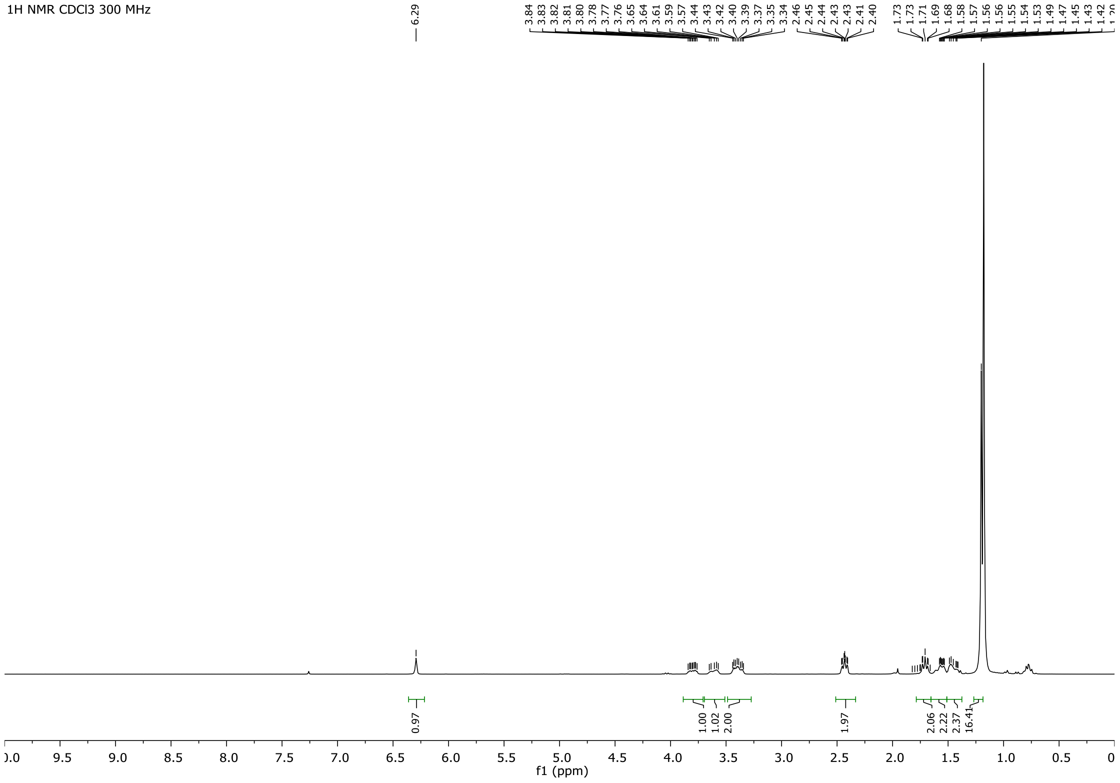

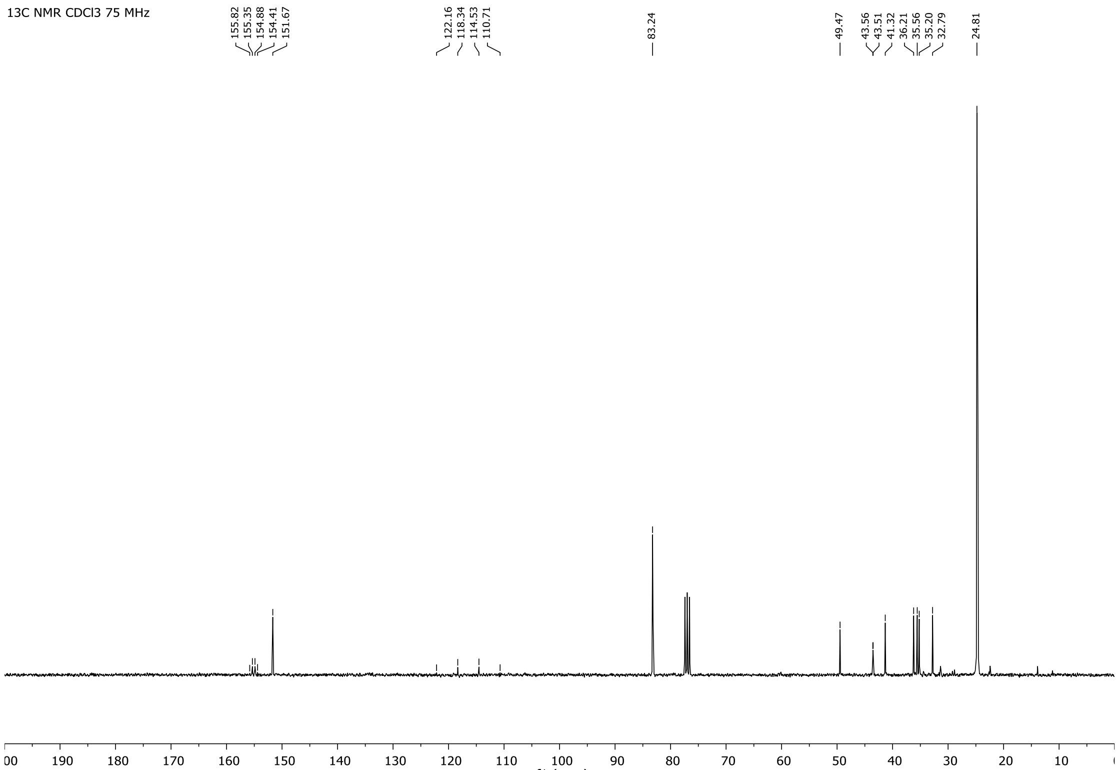

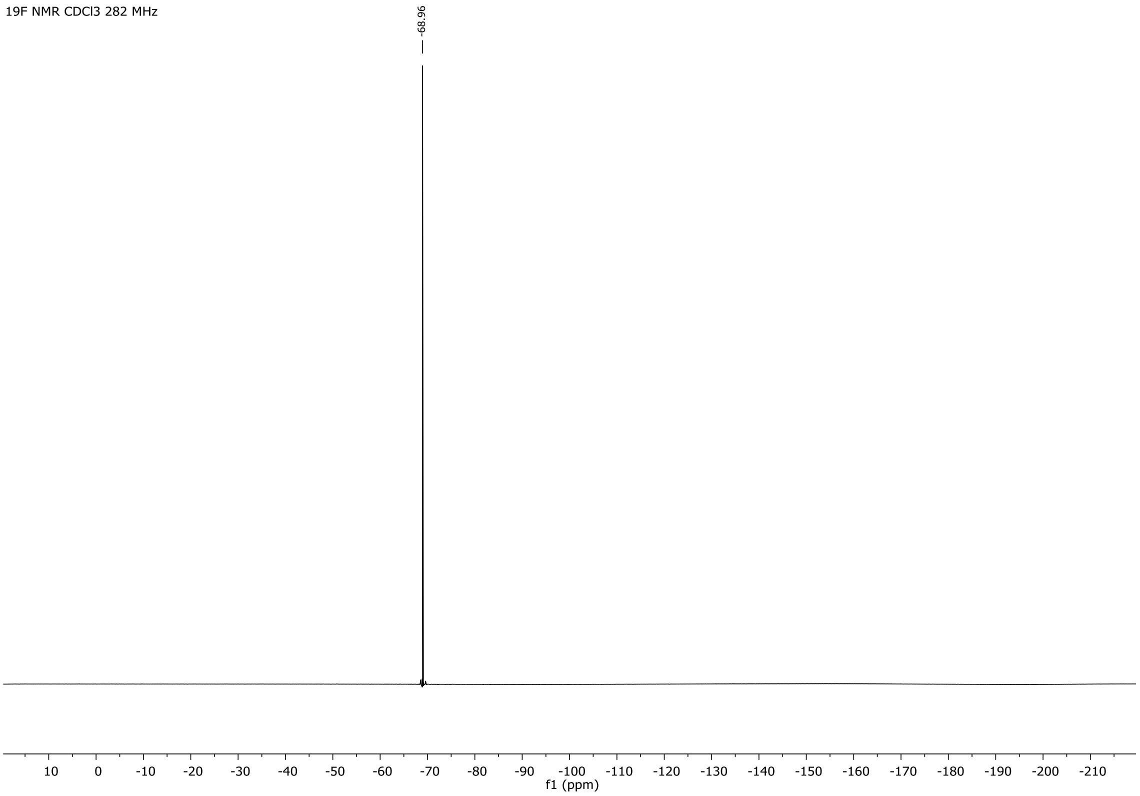
19F NMR CDCl3 282 MHz
-68.96
10 0 -10 -20 -30 -40 -50 -60 -70 -80 -90 -100 -110 -120 -130 -140 -150 -160 -170 -180 -190 -200 -210
f1 (ppm)

8-(2,2,2-trifluoroacetyl)-8-azaspiro[4.5]decan-2-one (28b)

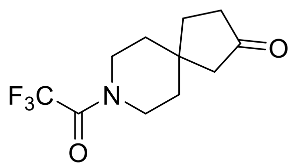

1H NMR CDCl3 300 MHz

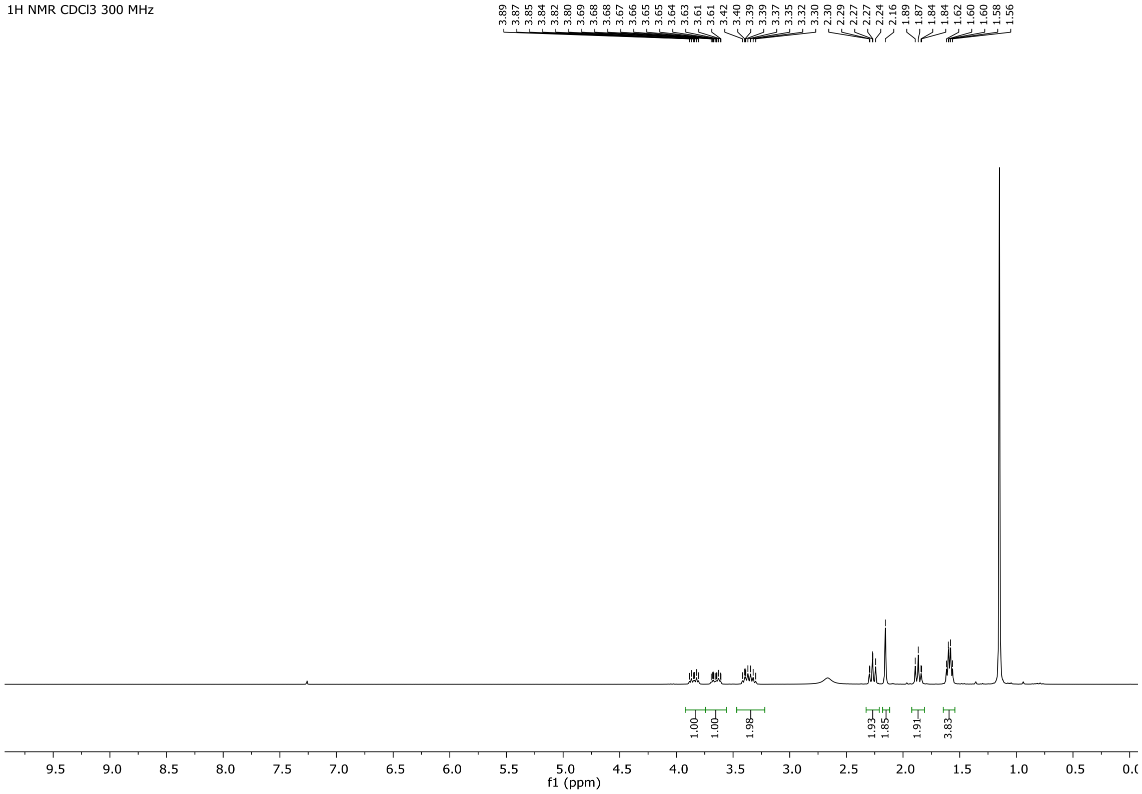

13C NMR CDCl3 75 MHz

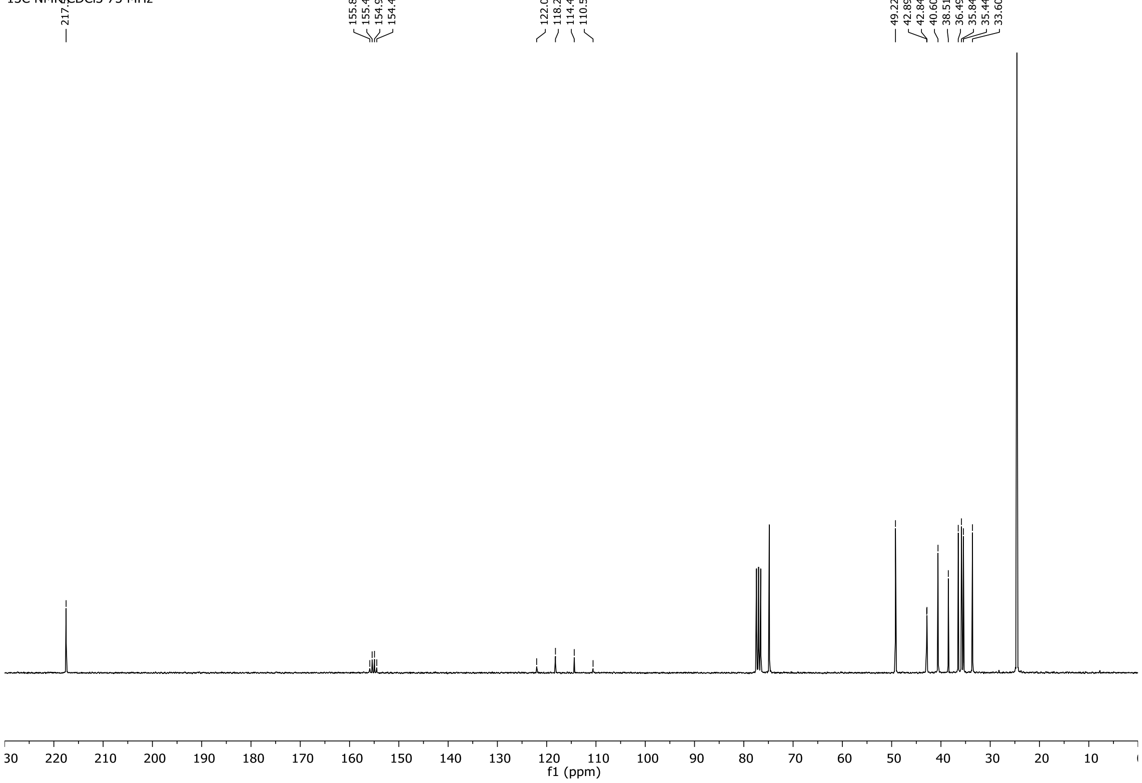

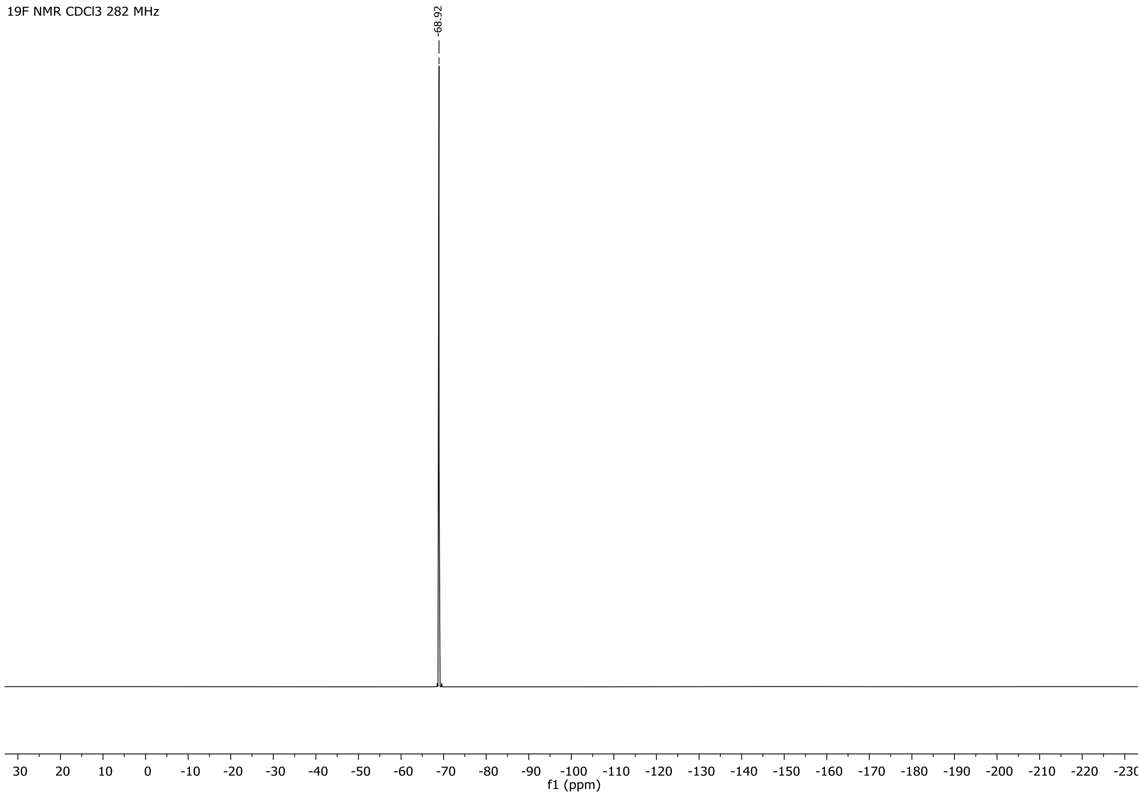
19F NMR CDCl3 282 MHz
-68.92
30 20 10 0 -10 -20 -30 -40 -50 -60 -70 -80 -90 -100 -110 -120 -130 -140 -150 -160 -170 -180 -190 -200 -210 -220 -230
f1 (ppm)

N-phenyl-8-oxaspiro[4.5]decane-2-carboxamide (31)

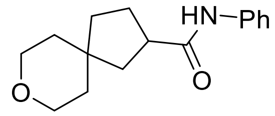

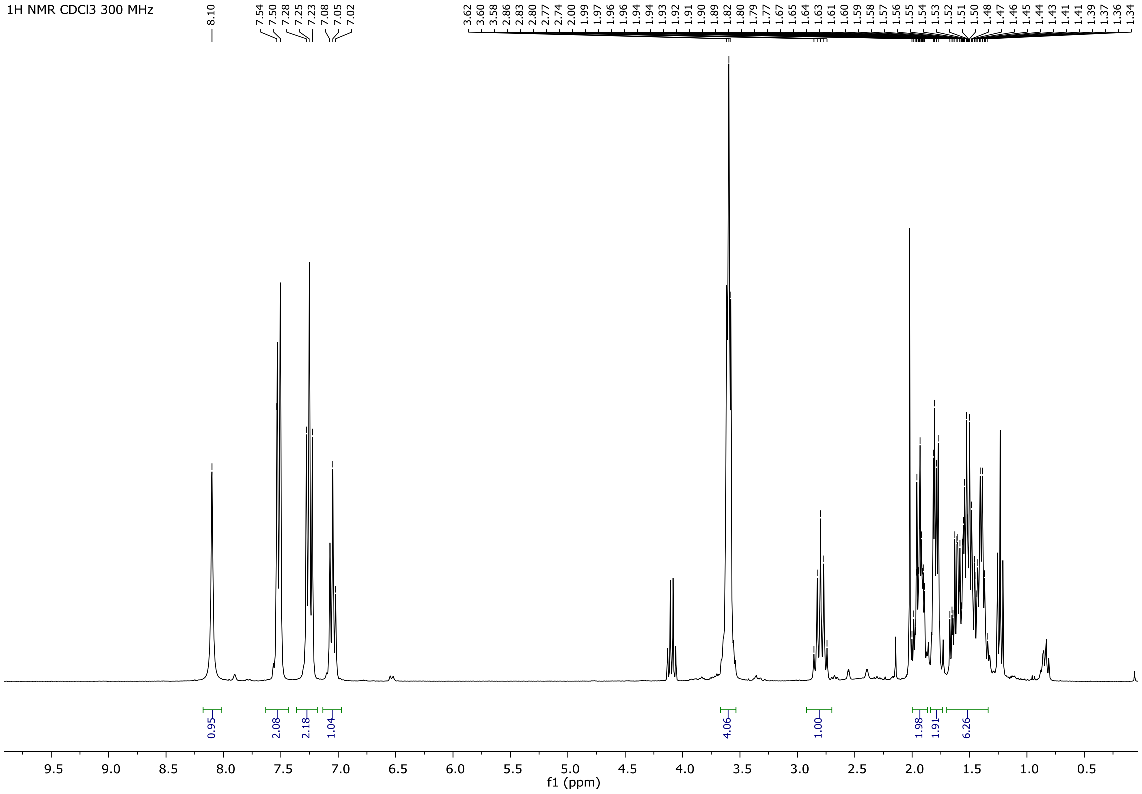

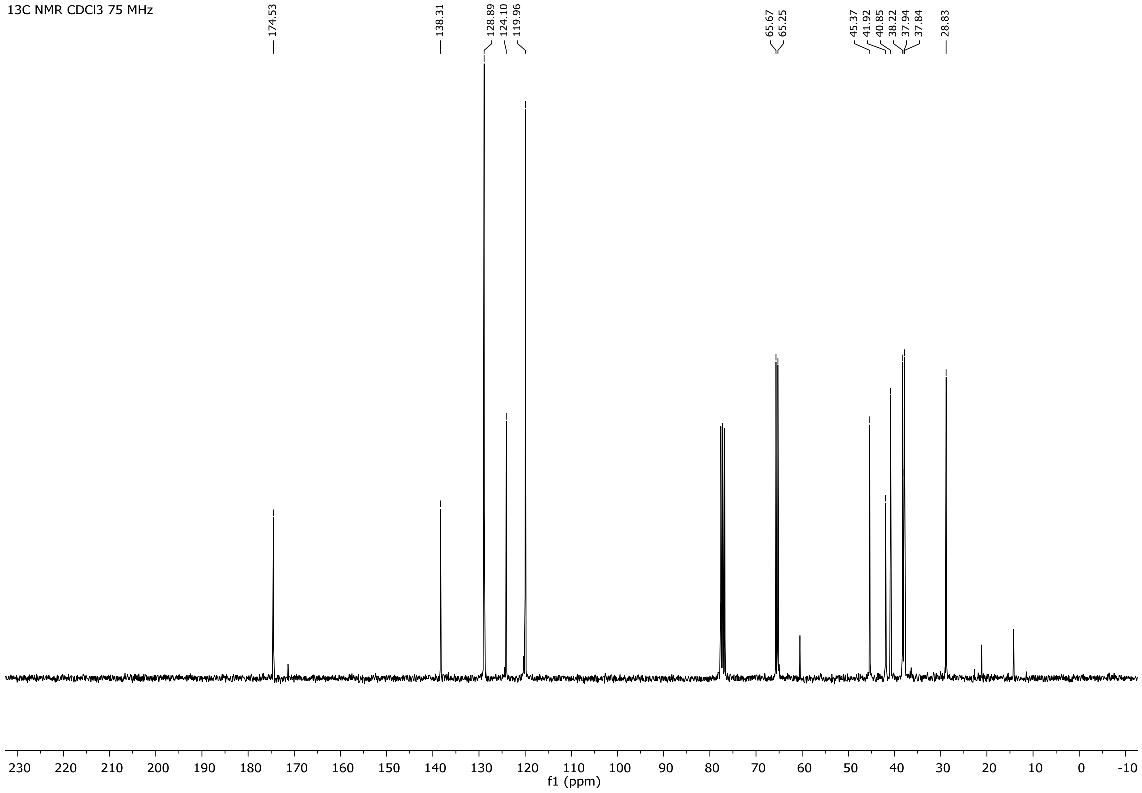

N-(8-oxaspiro[4.5]decan-2-yl)benzamide (33)

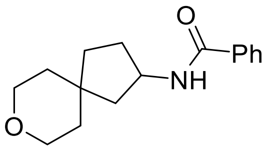

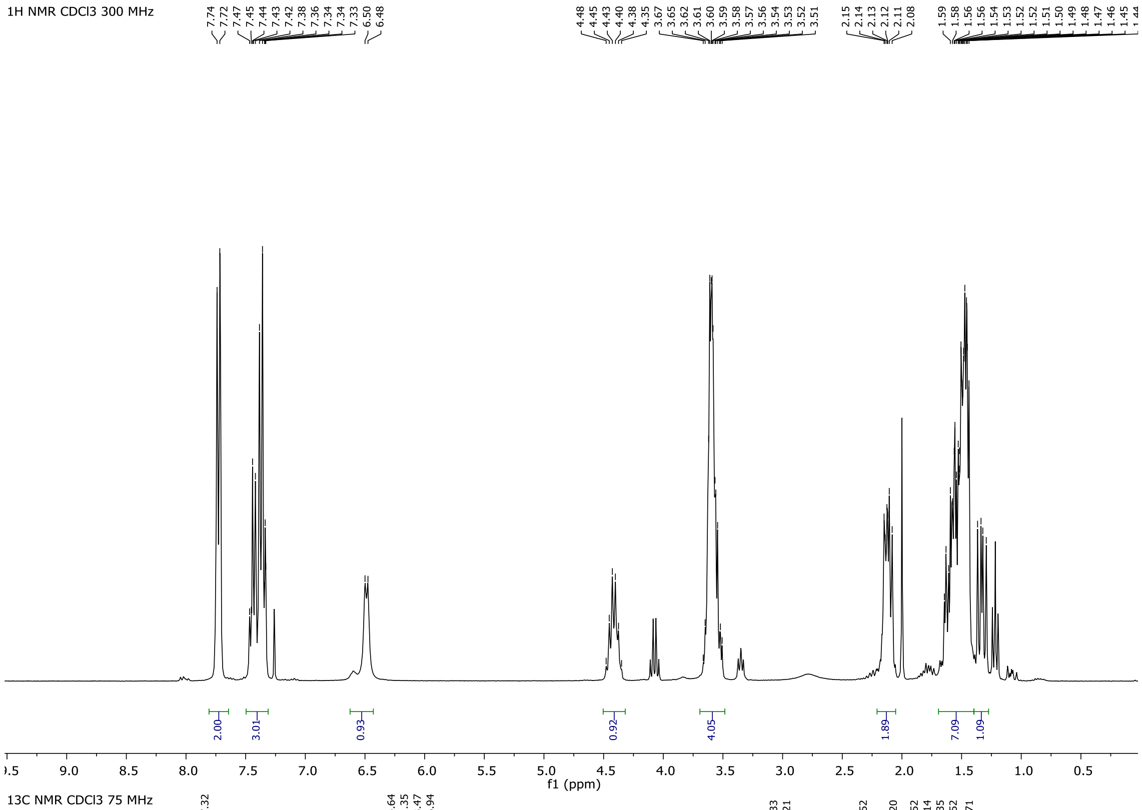

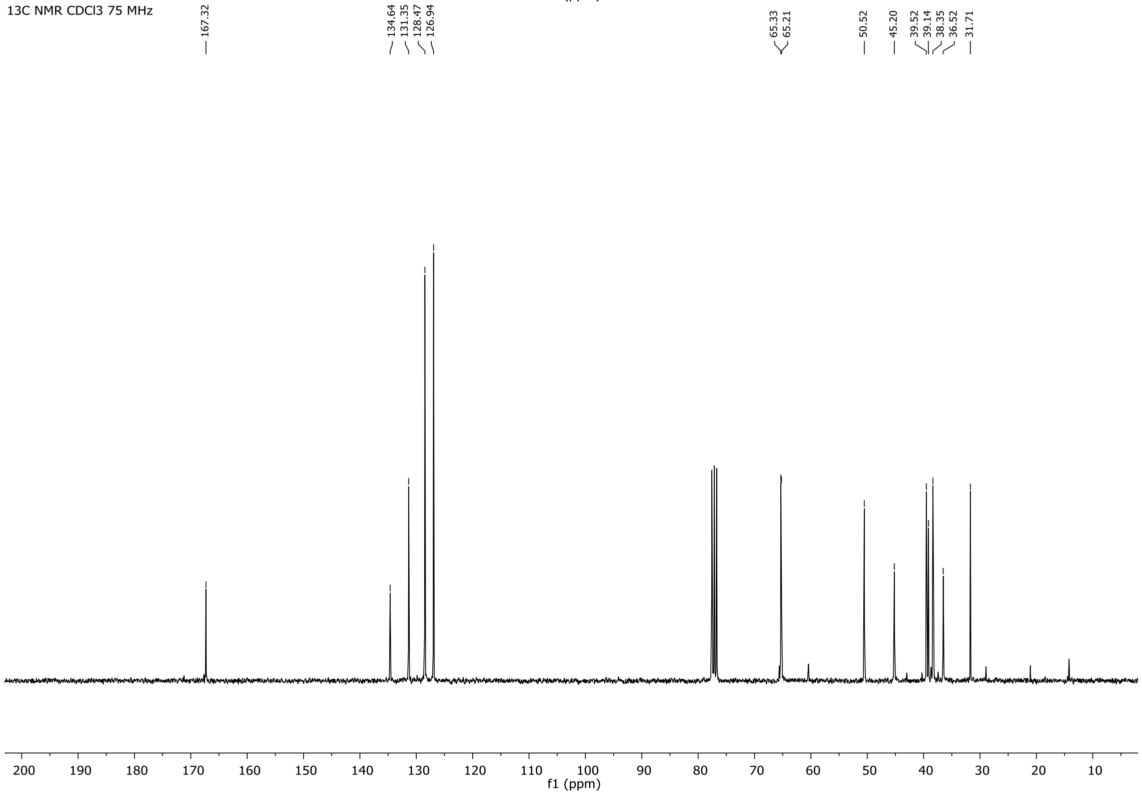

N-((8-oxaspiro[4.5]decan-2-yl)methyl)benzamide (34)

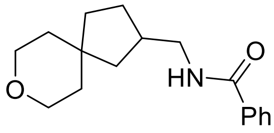

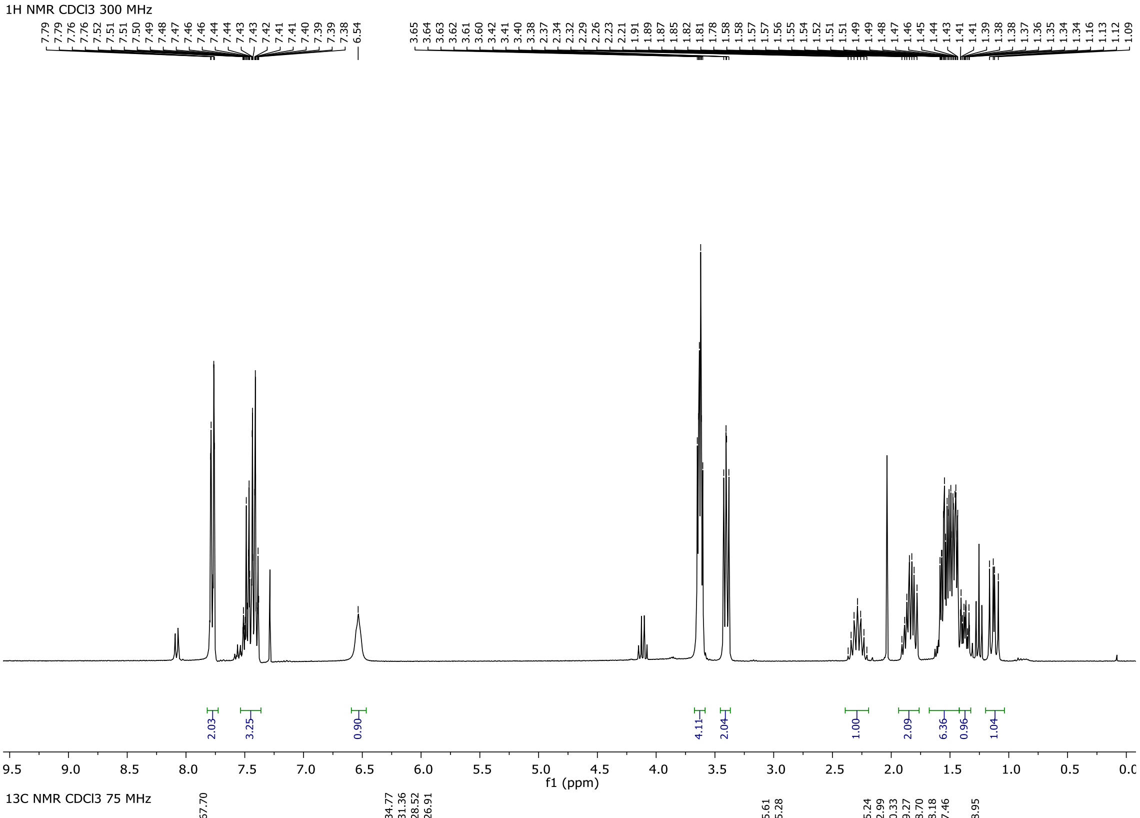

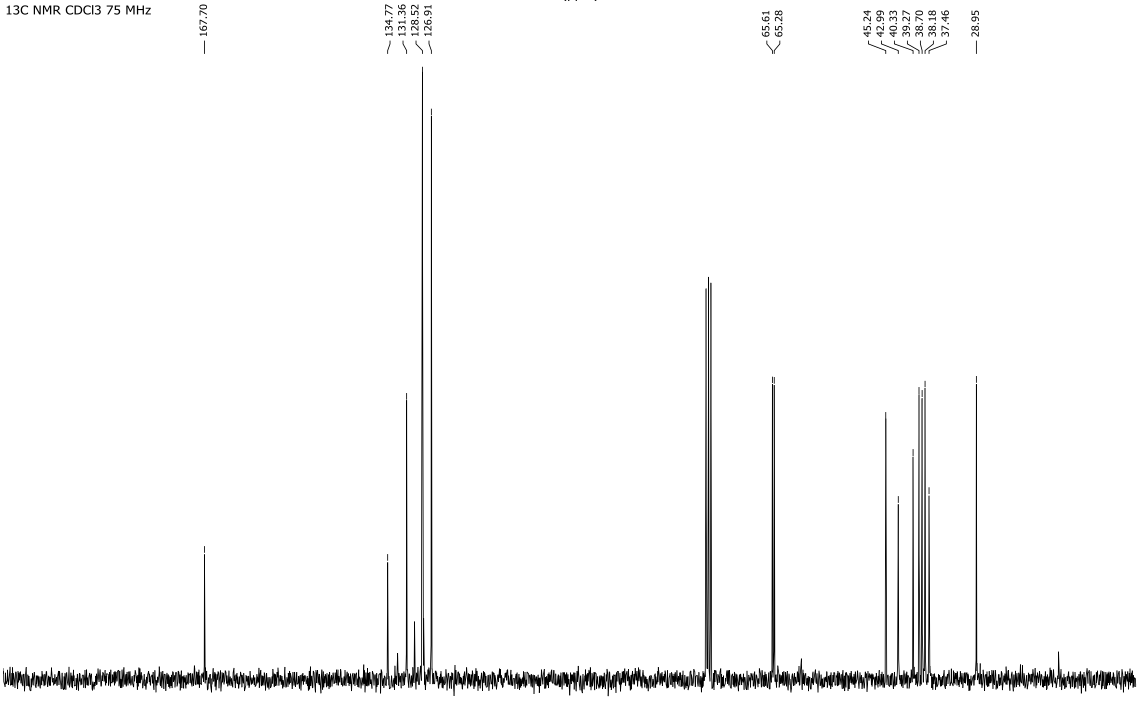

tert-butyl 2-(benzamidomethyl)-8-azaspiro[4.5]decane-8-carboxylate (35)

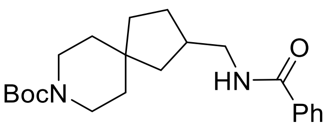

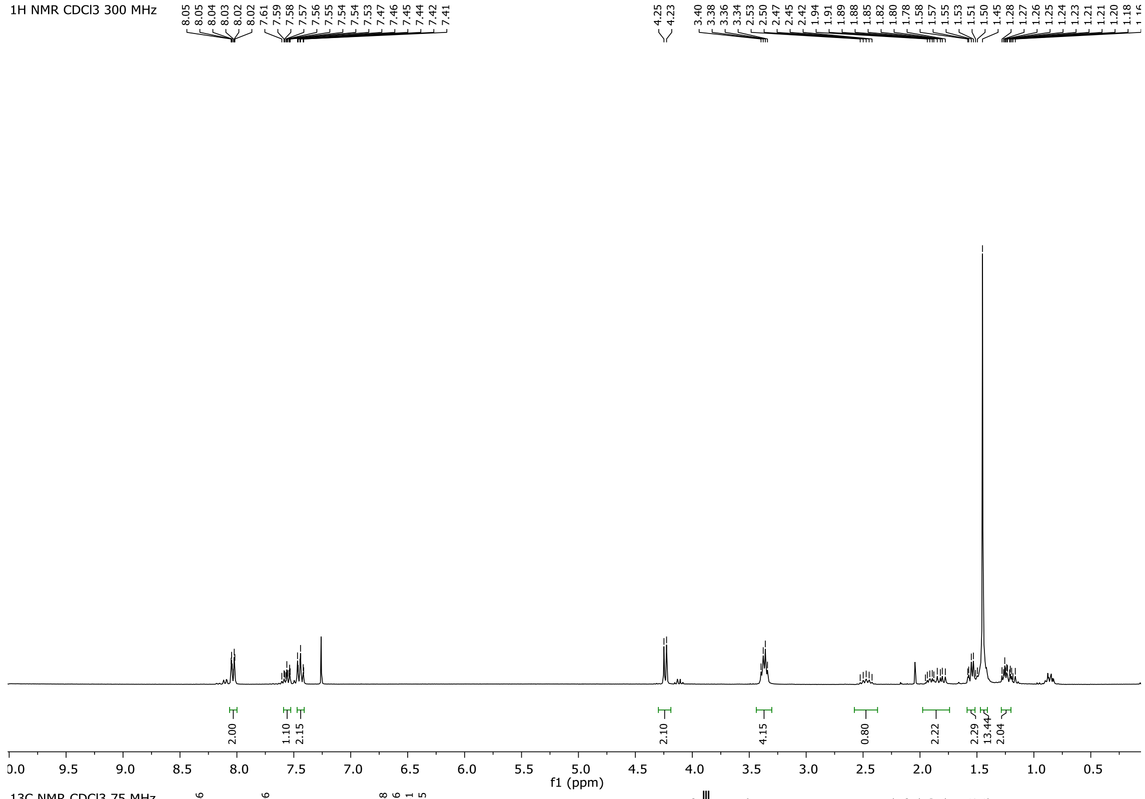

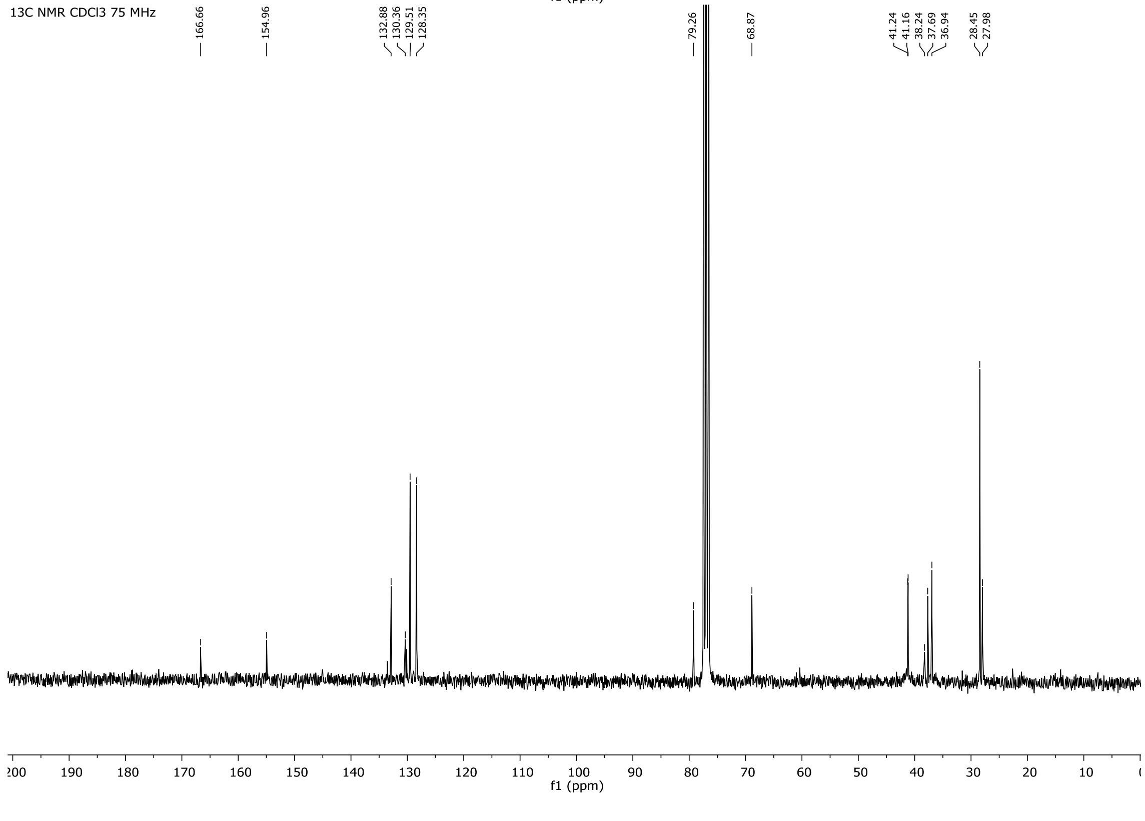

tert-butyl 2-benzamido-8-azaspiro[4.5]decane-8-carboxylate (36)

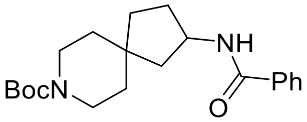

1H NMR CDCl3 300 MHz

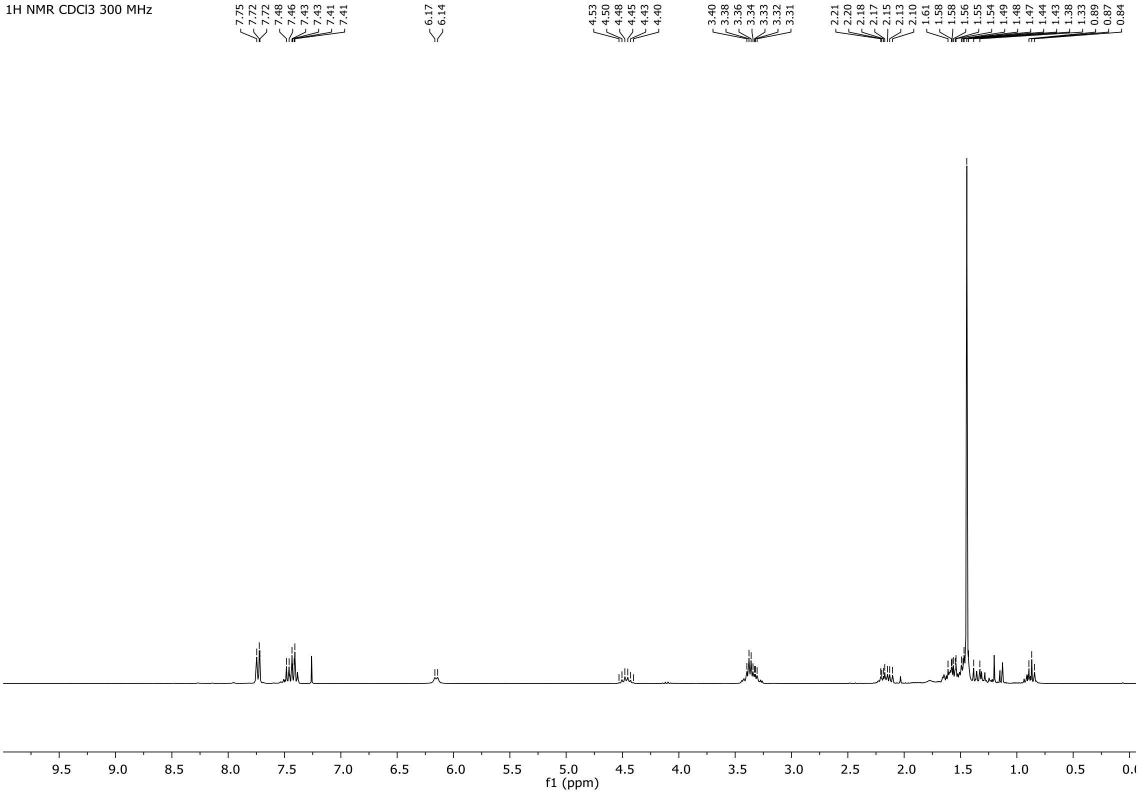

13C NMR CDCl3 75 MHz

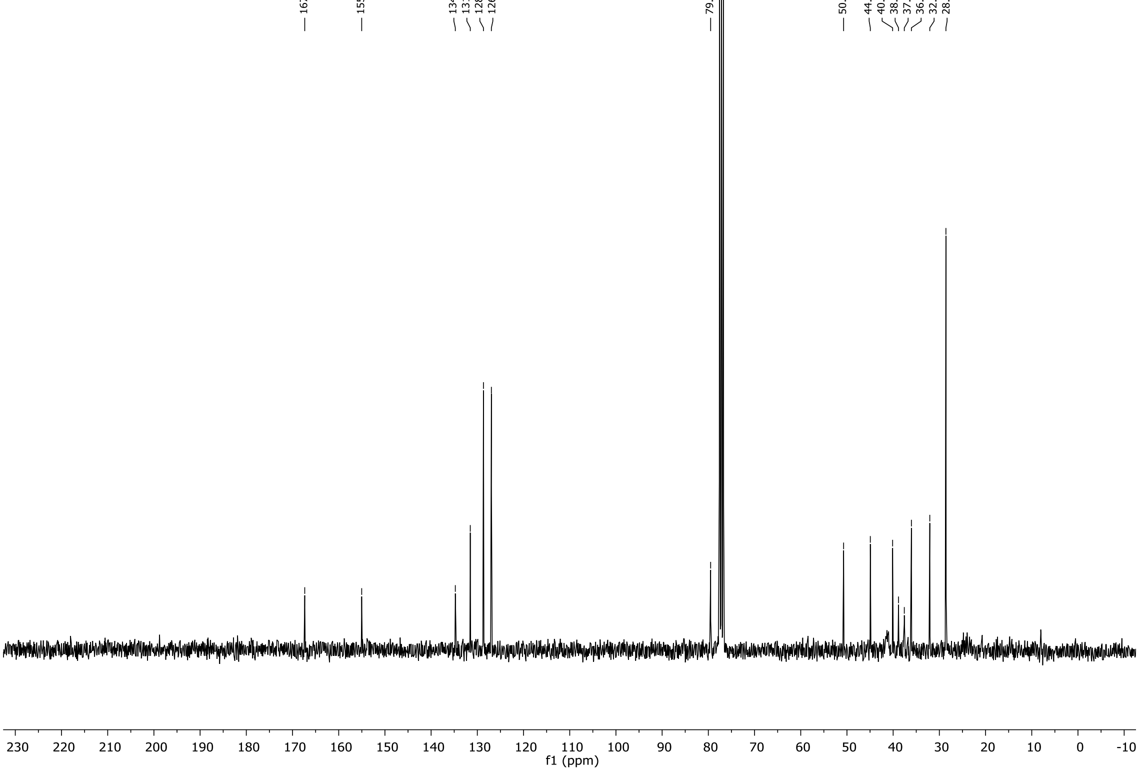

tert-butyl 2-(phenylcarbamoyl)-8-azaspiro[4.5]decane-8-carboxylate (37)

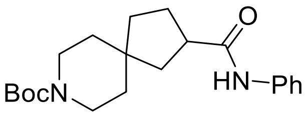

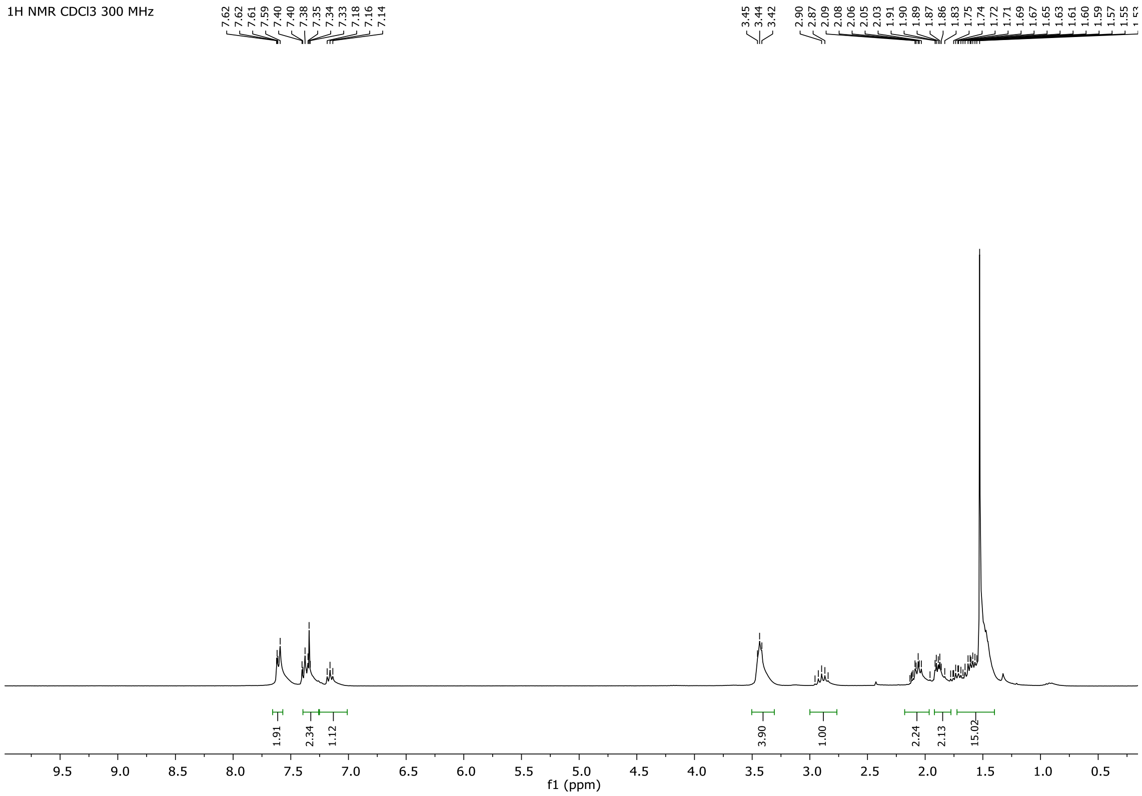

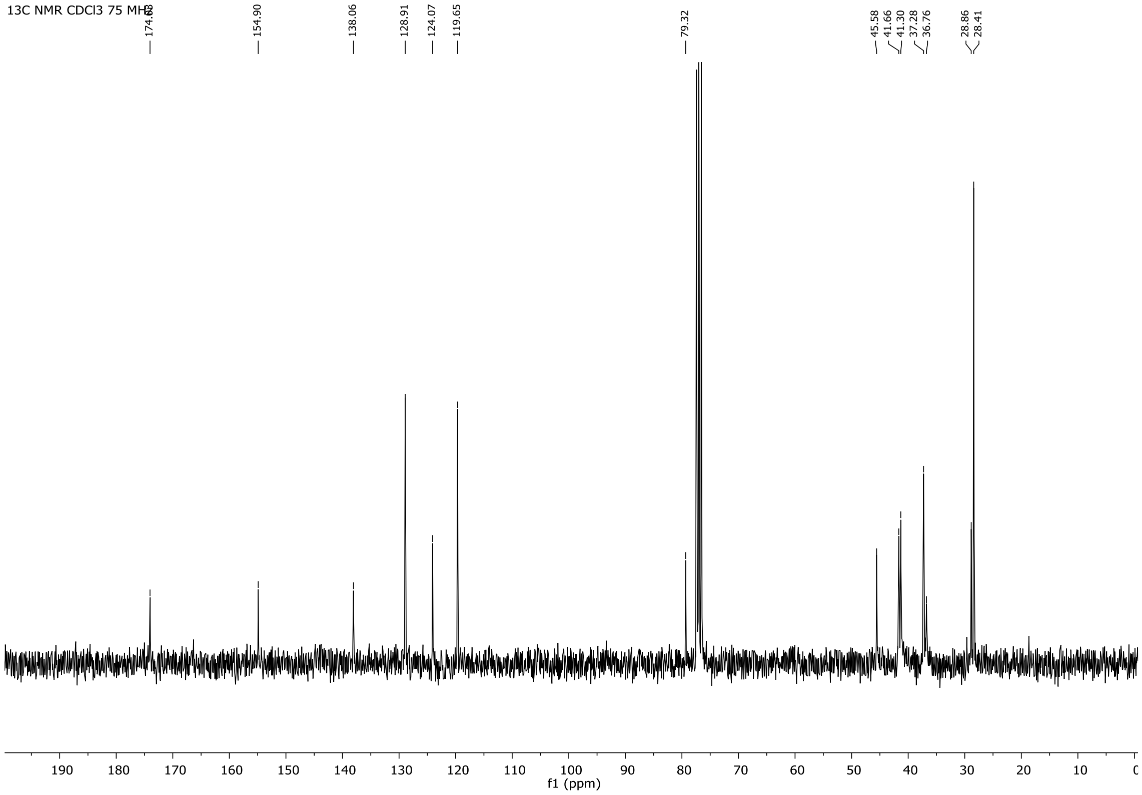

LIMITATIONS

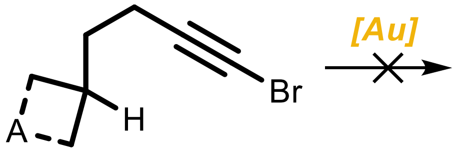

[bromo alkynes]

tert-butyl 3-(2-hydroxyethyl)azetidine-1-carboxylate (38-OH)

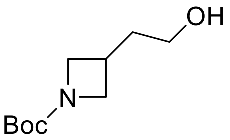

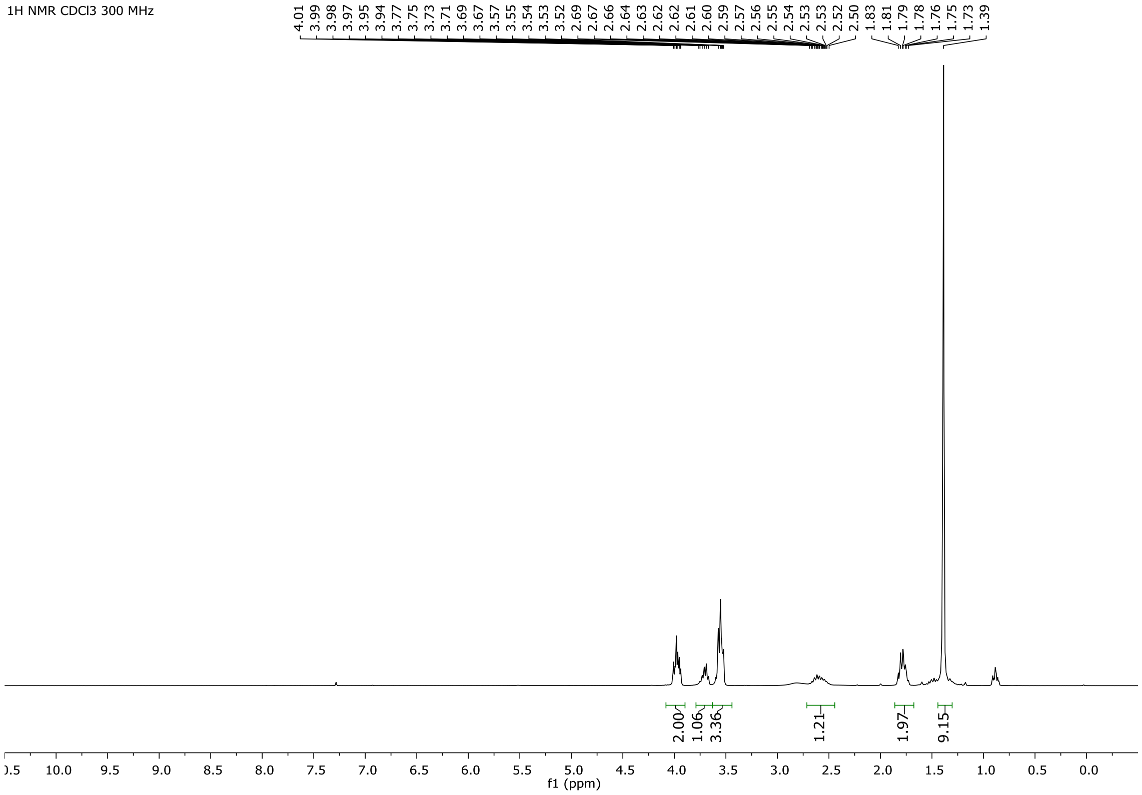

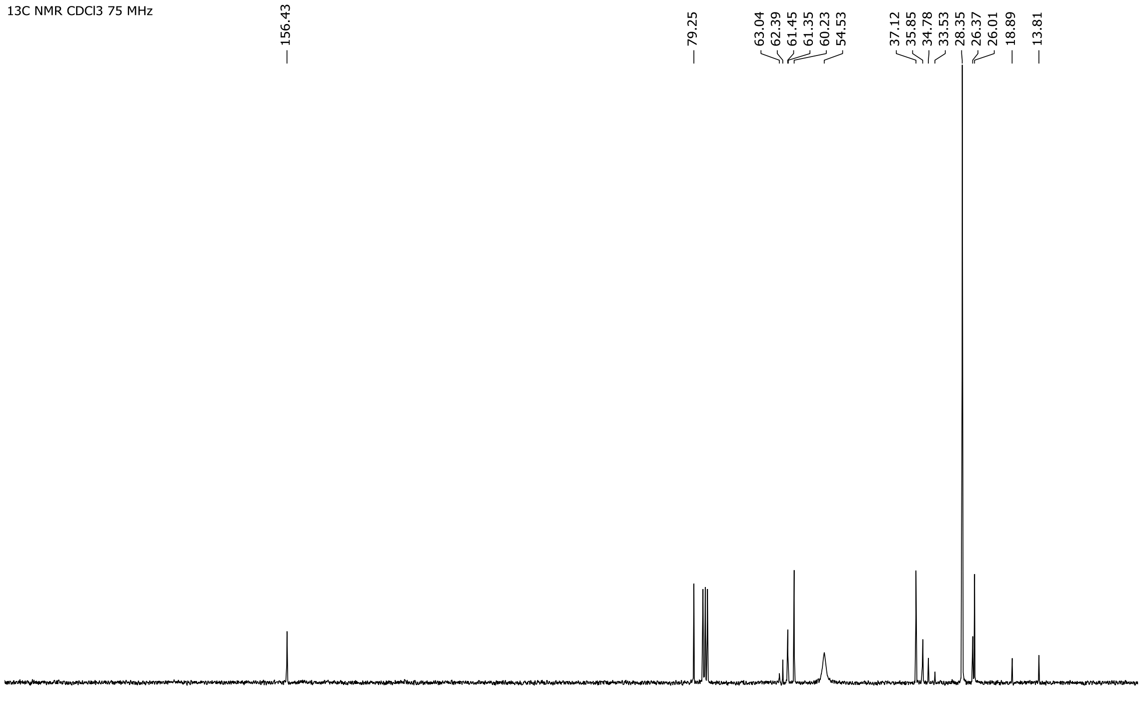

tert-butyl 3-(2-bromoethyl)azetidine-1-carboxylate (38-Br)

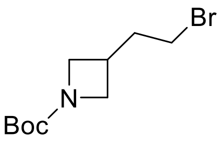

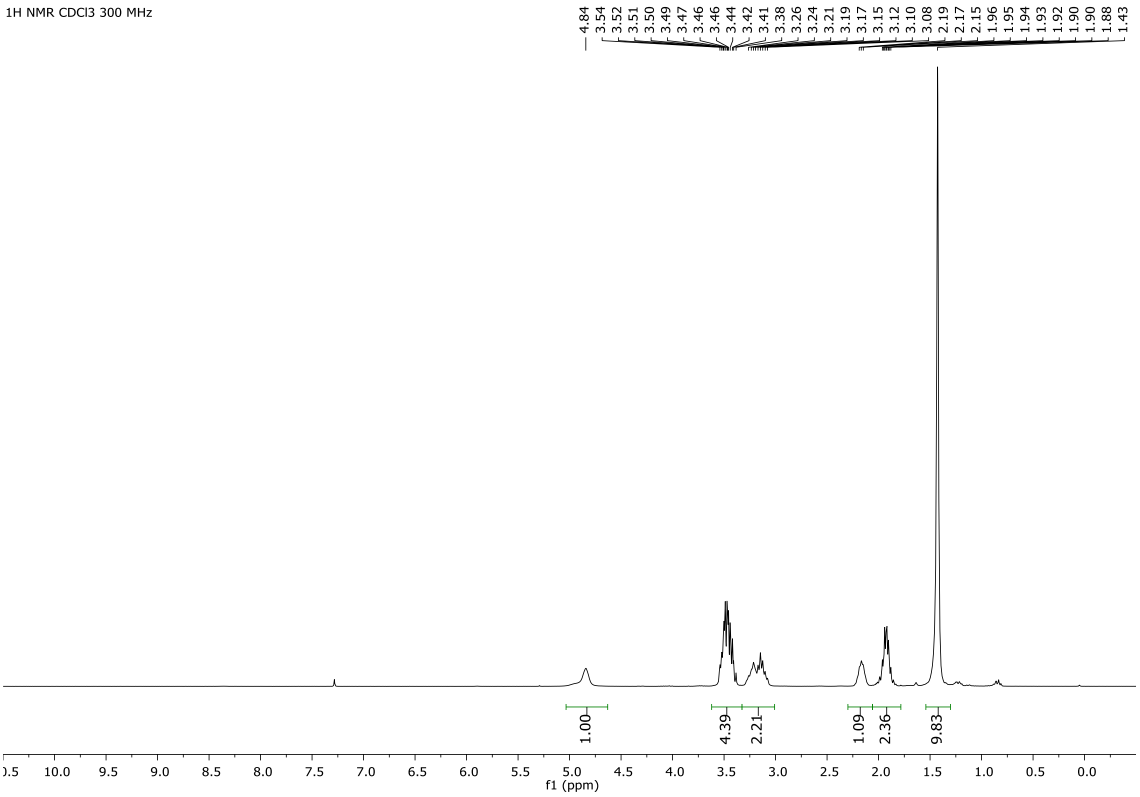

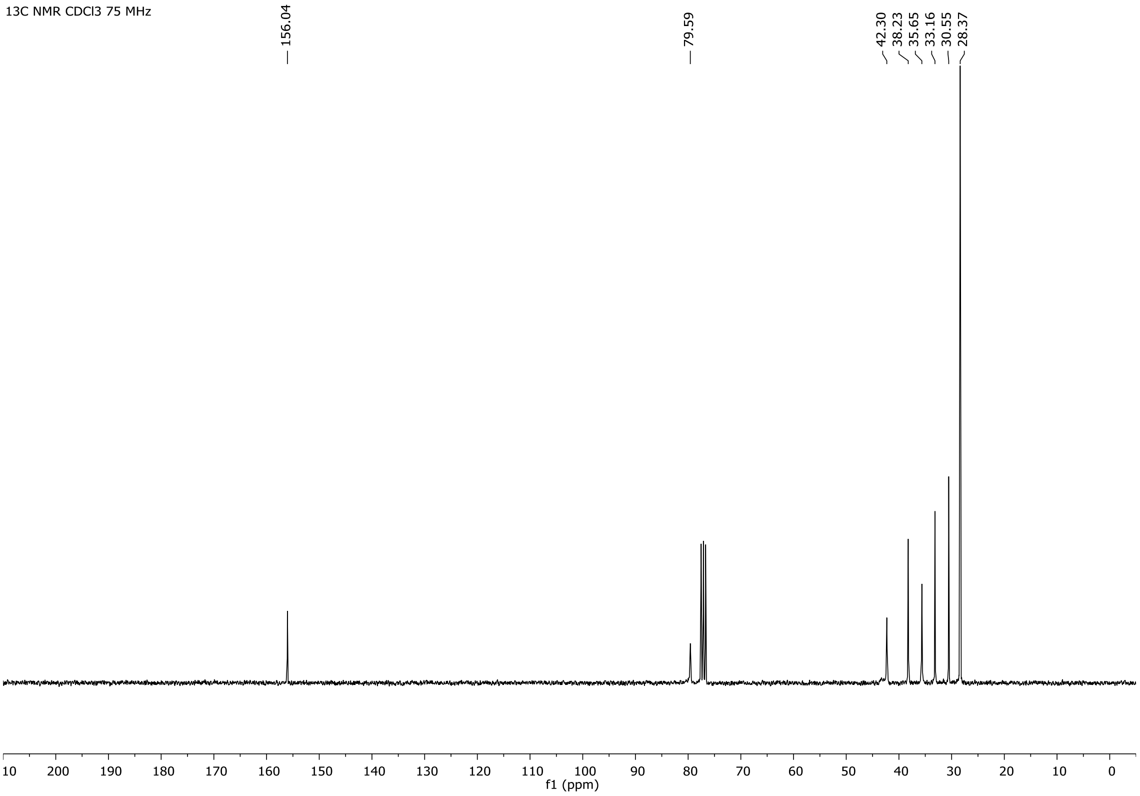

tert-butyl 3-(4-(trimethylsilyl)but-3-yn-1-yl)azetidine-1-carboxylate (38-CCTMS)

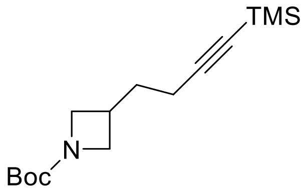

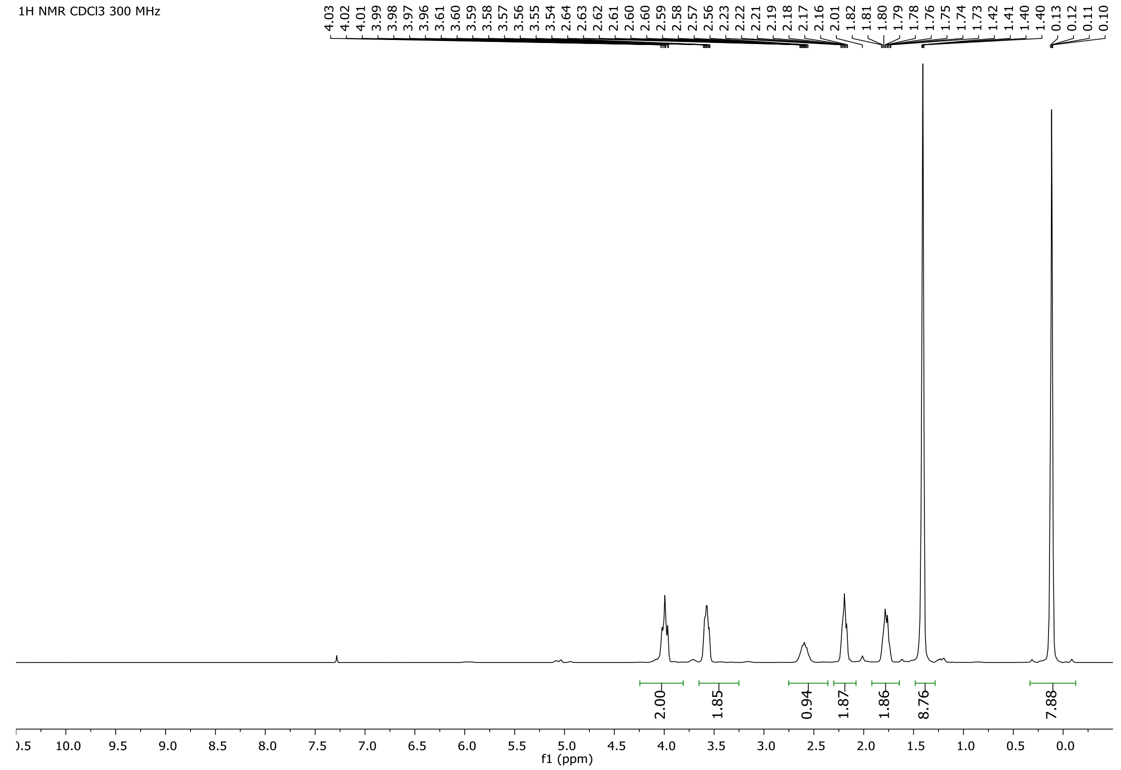

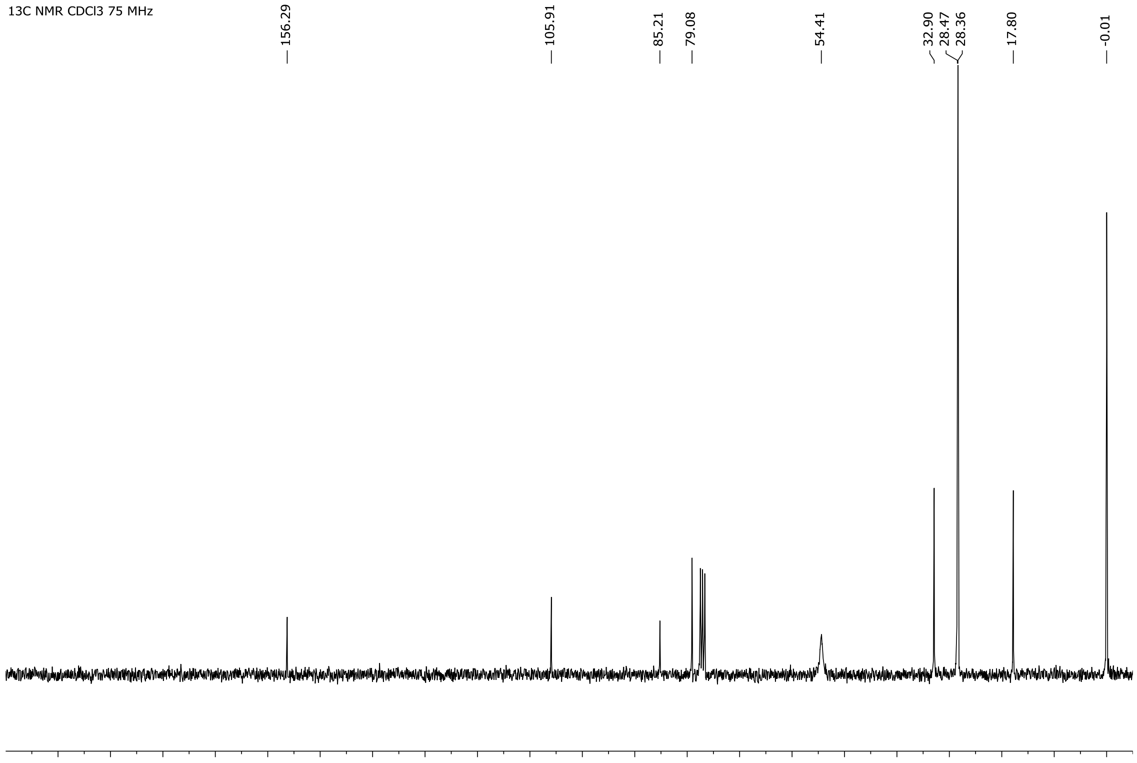

tert-butyl 3-(4-bromobut-3-yn-1-yl)azetidine-1-carboxylate (38-NBoc)

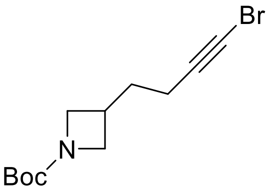

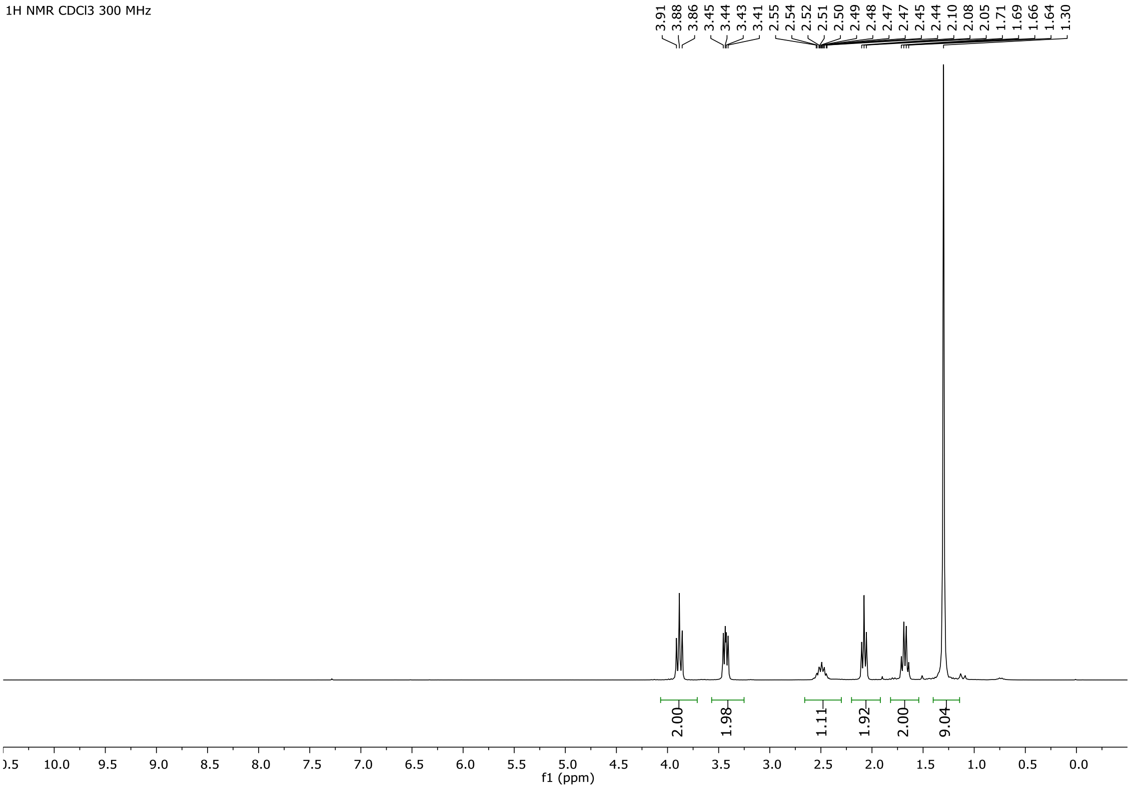

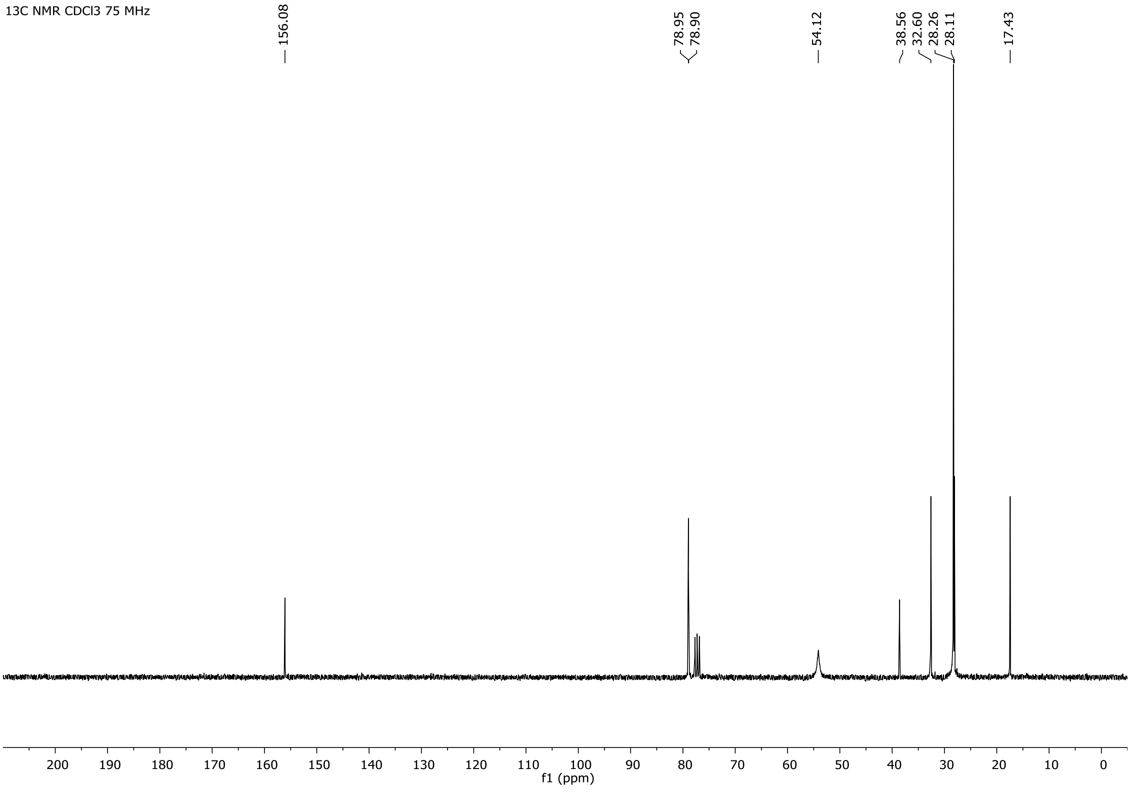

1-(3-(4-bromobut-3-yn-1-yl)azetidin-1-yl)-2,2,2-trifluoroethan-1-one (38)

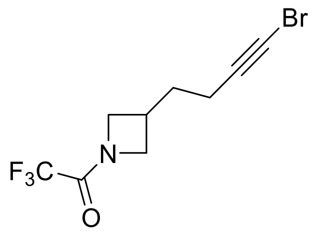

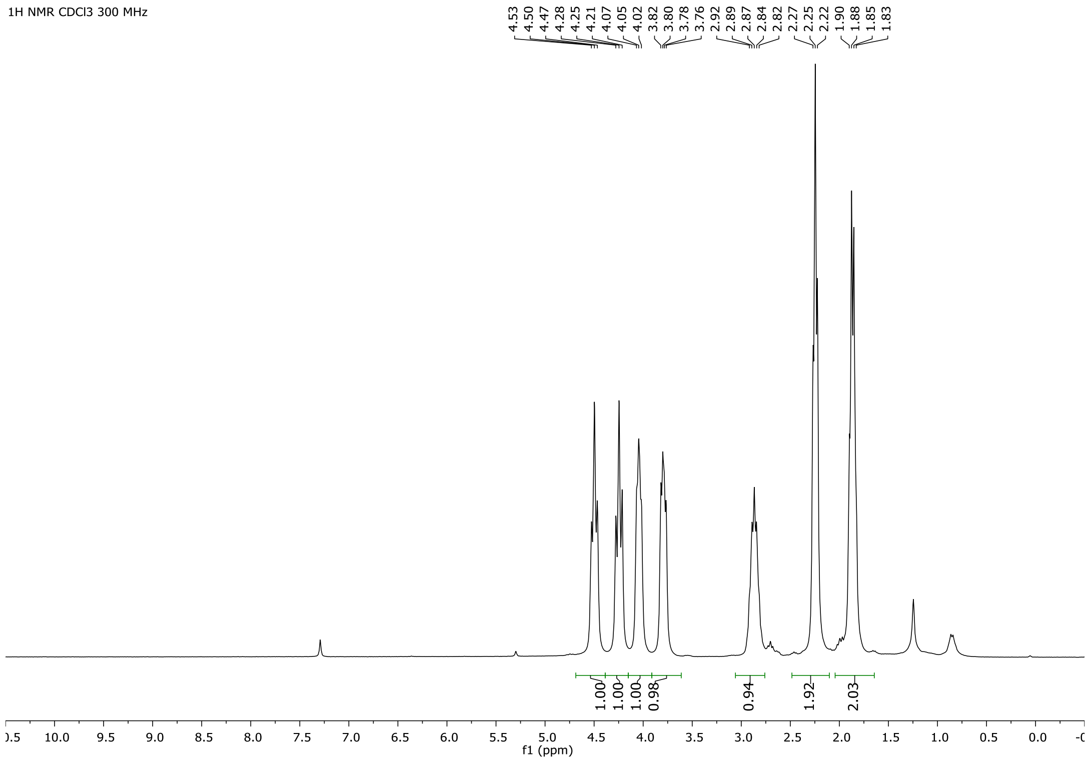

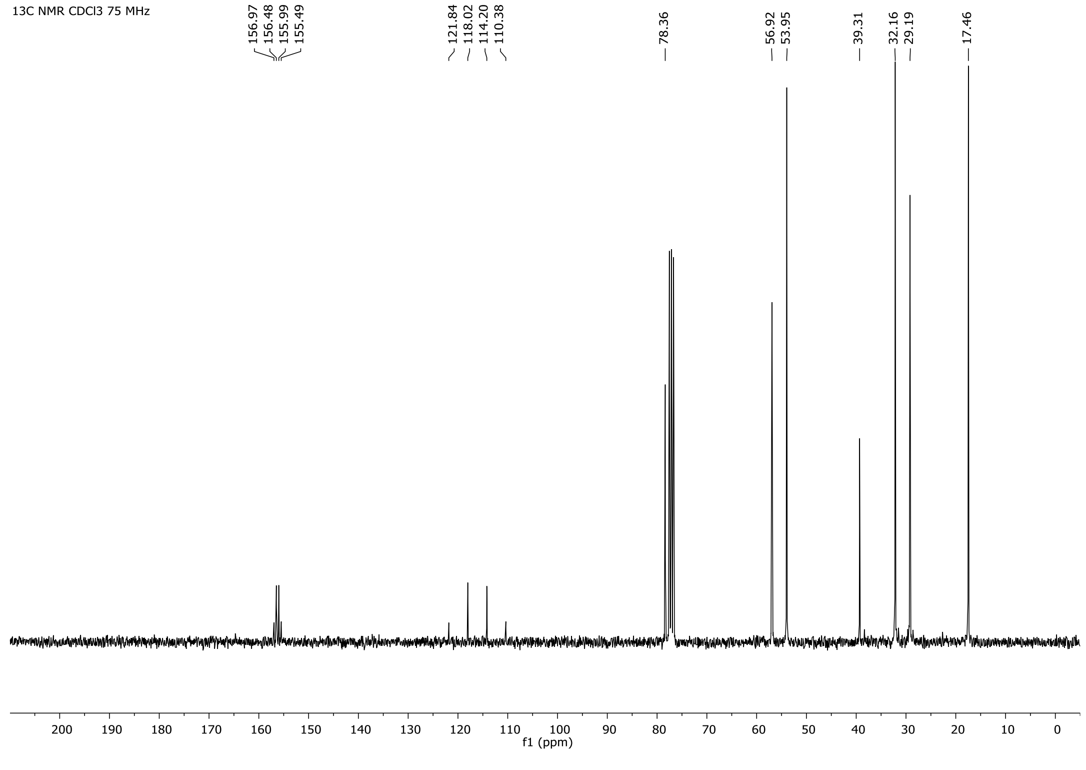

((3-bromoprop-2-yn-1-yl)oxy)cyclohexane (39)

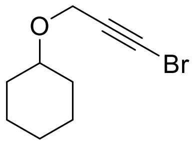

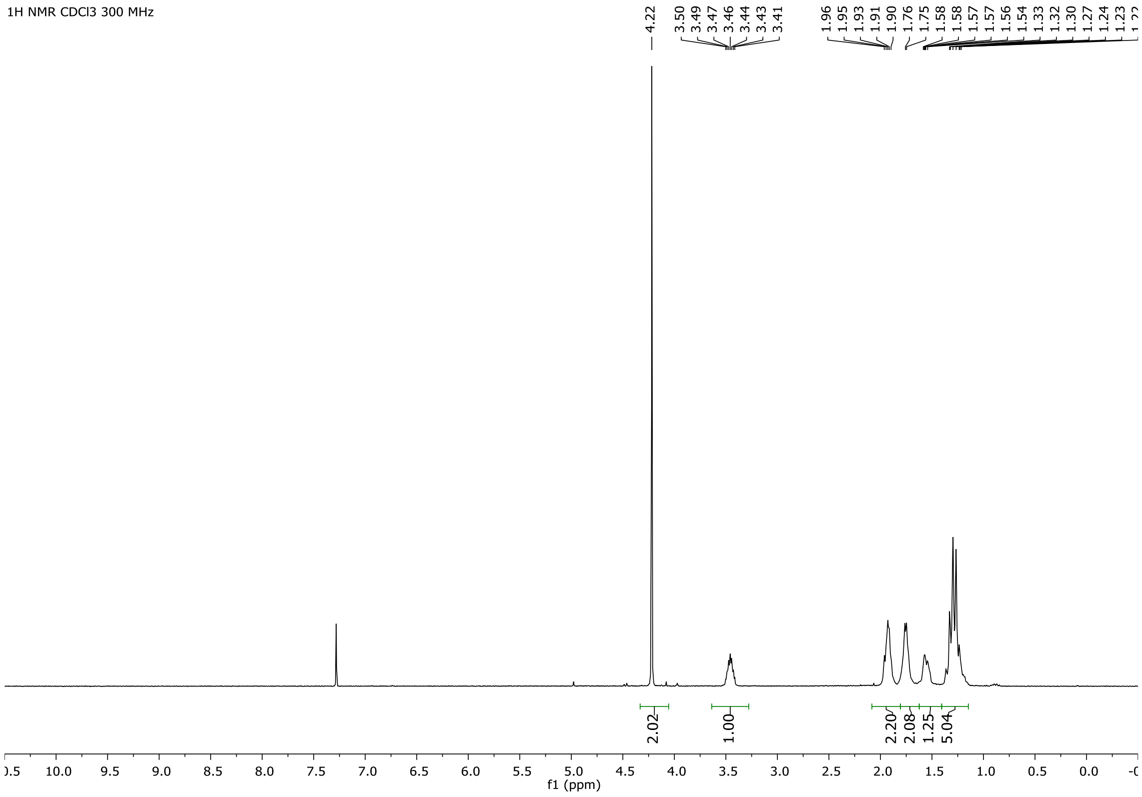

2-(2-oxaspiro[3.3]heptan-6-yl)ethan-1-ol (40-OH)

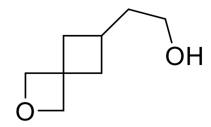

1H NMR CDCl3 300 MHz

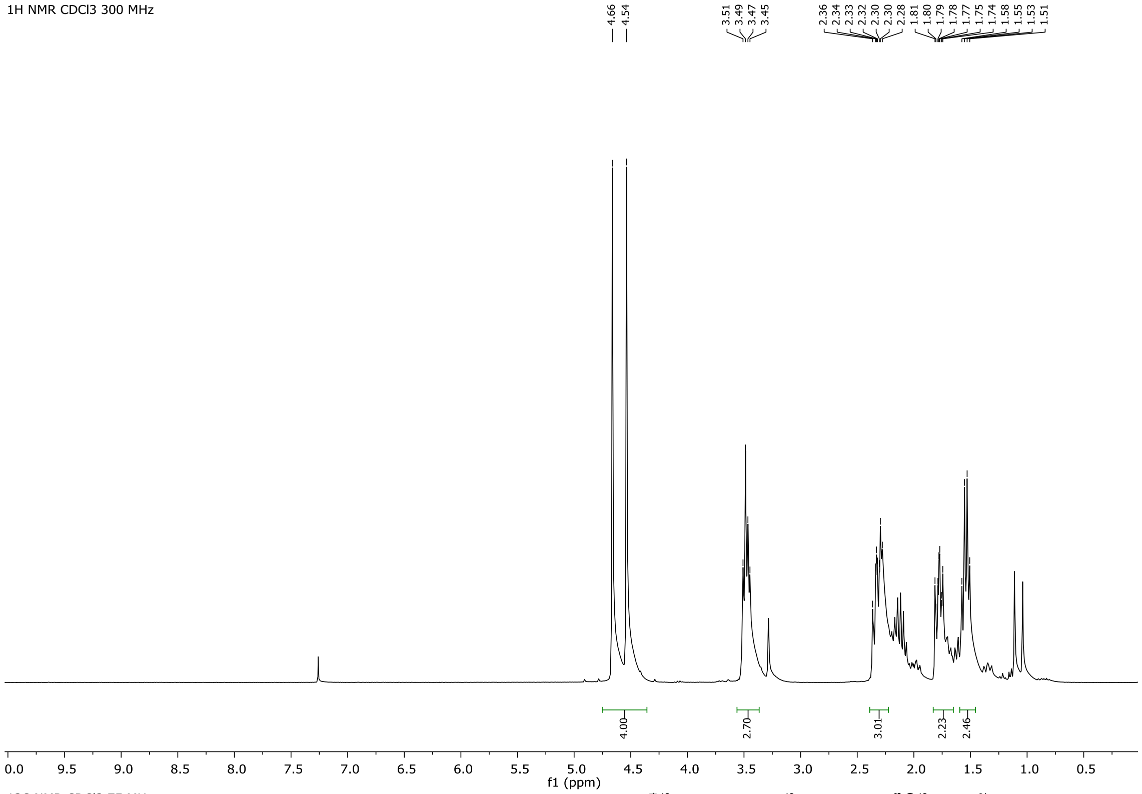

13C NMR CDCl3 75 MHz

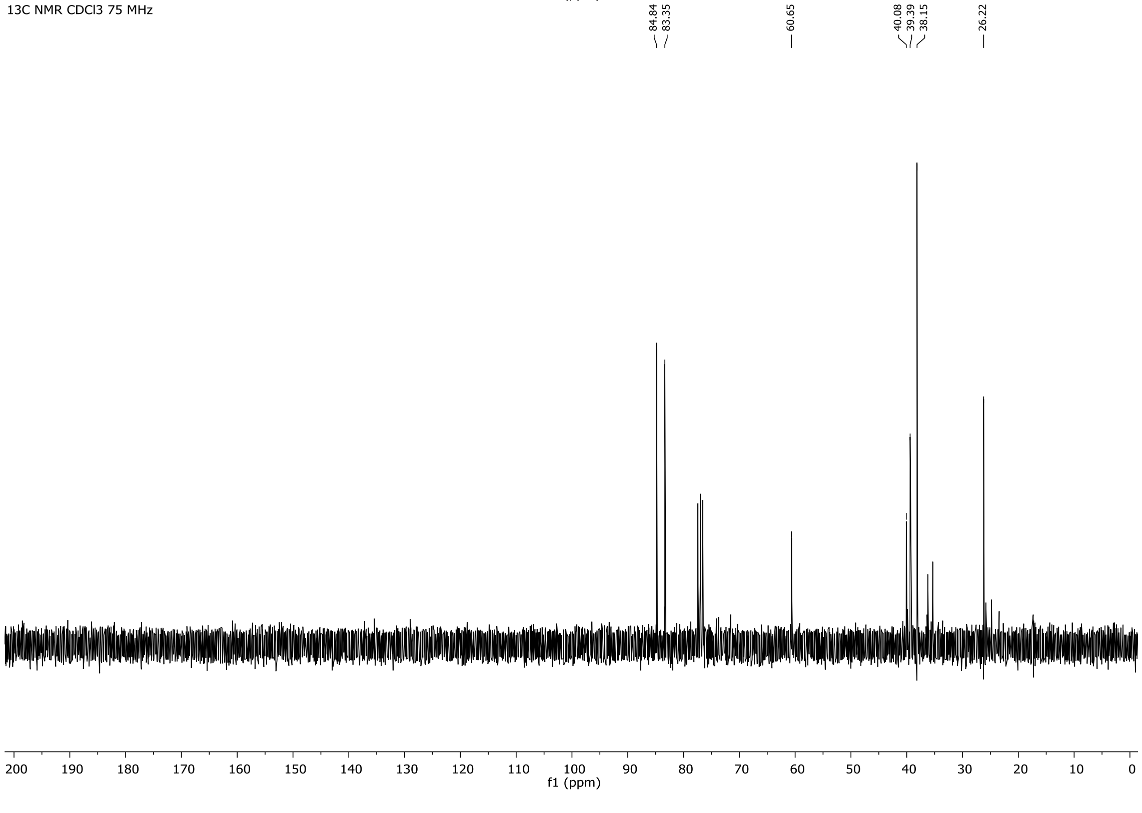

6-(2-bromoethyl)-2-oxaspiro[3.3]heptane (40-Br)

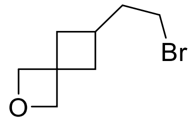

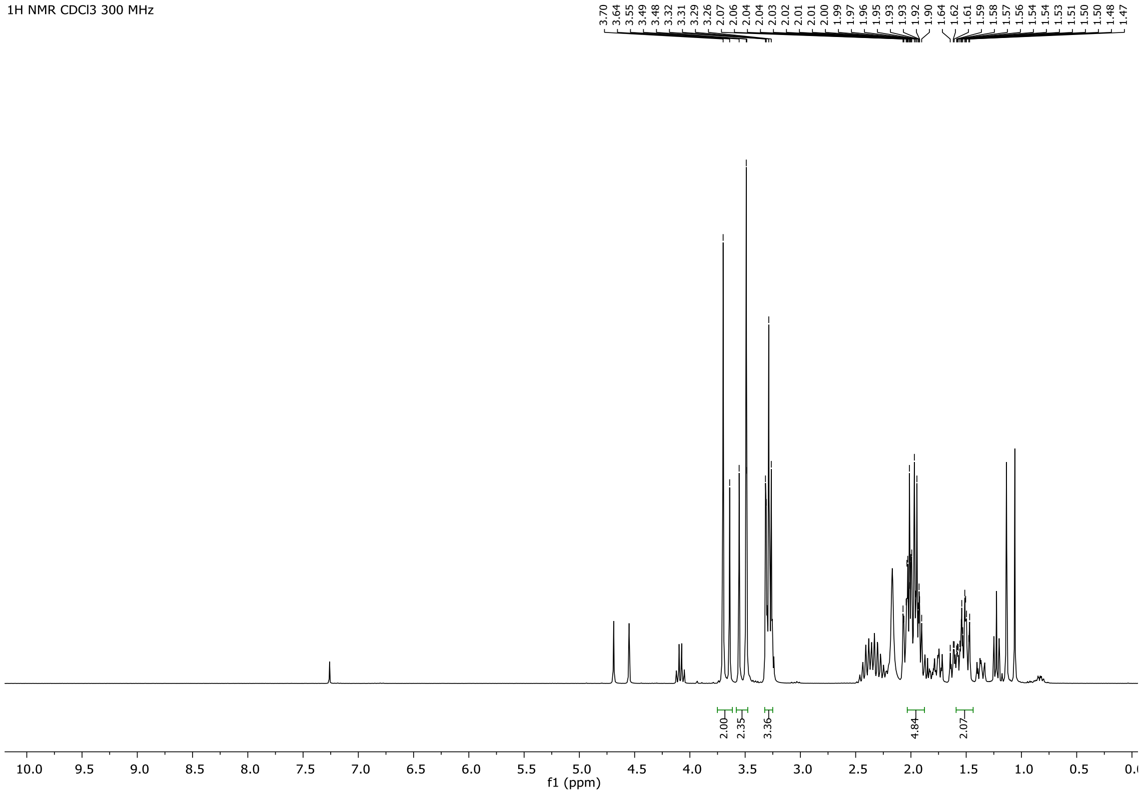

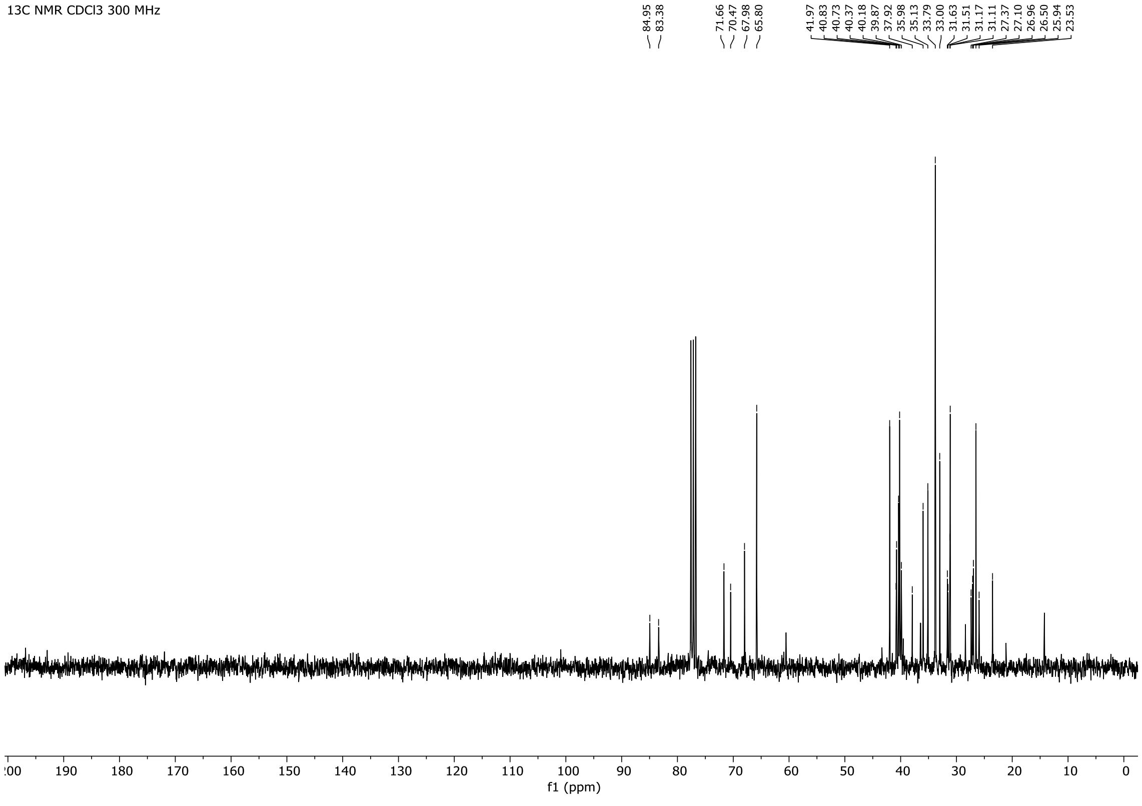

6-(but-3-yn-1-yl)-2-oxaspiro[3.3]heptane (40-CCH)

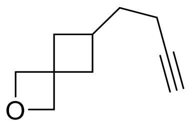

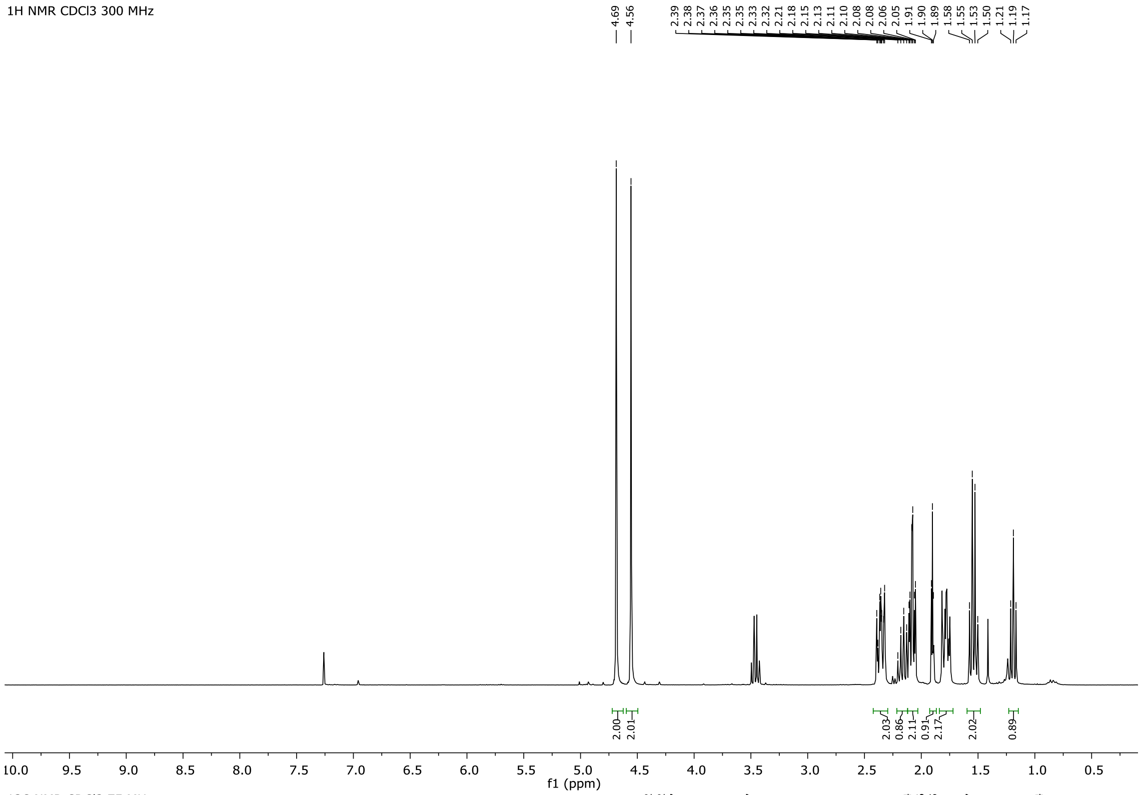

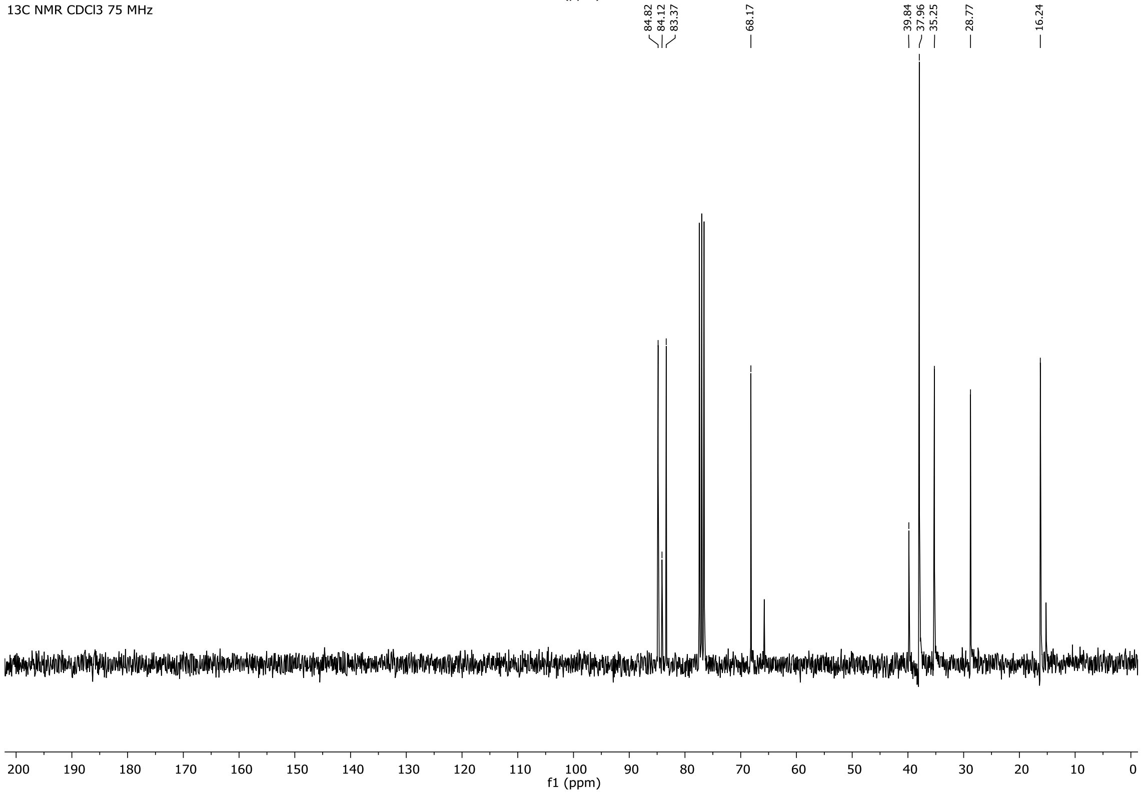

6-(4-bromobut-3-yn-1-yl)-2-oxaspiro[3.3]heptane (40)

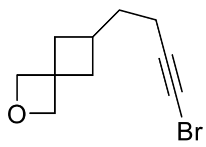

1H NMR CDCl3 300 MHz

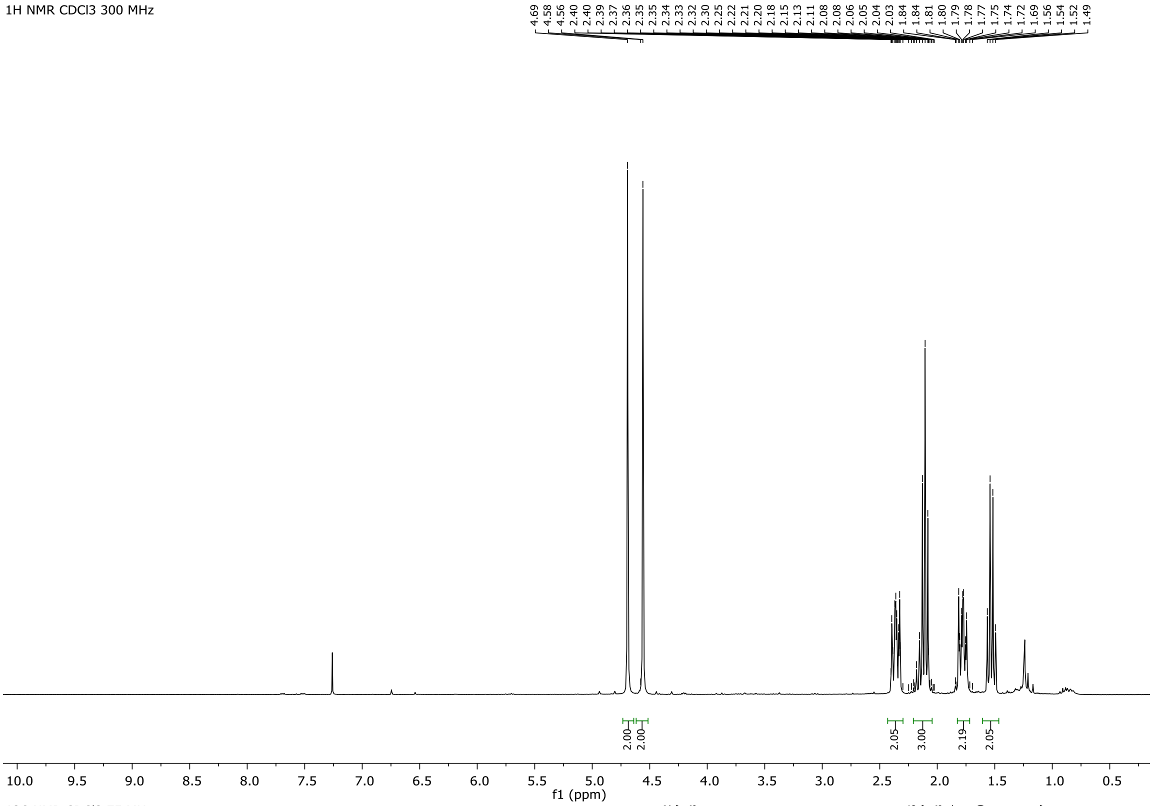

13C NMR CDCl3 75 MHz

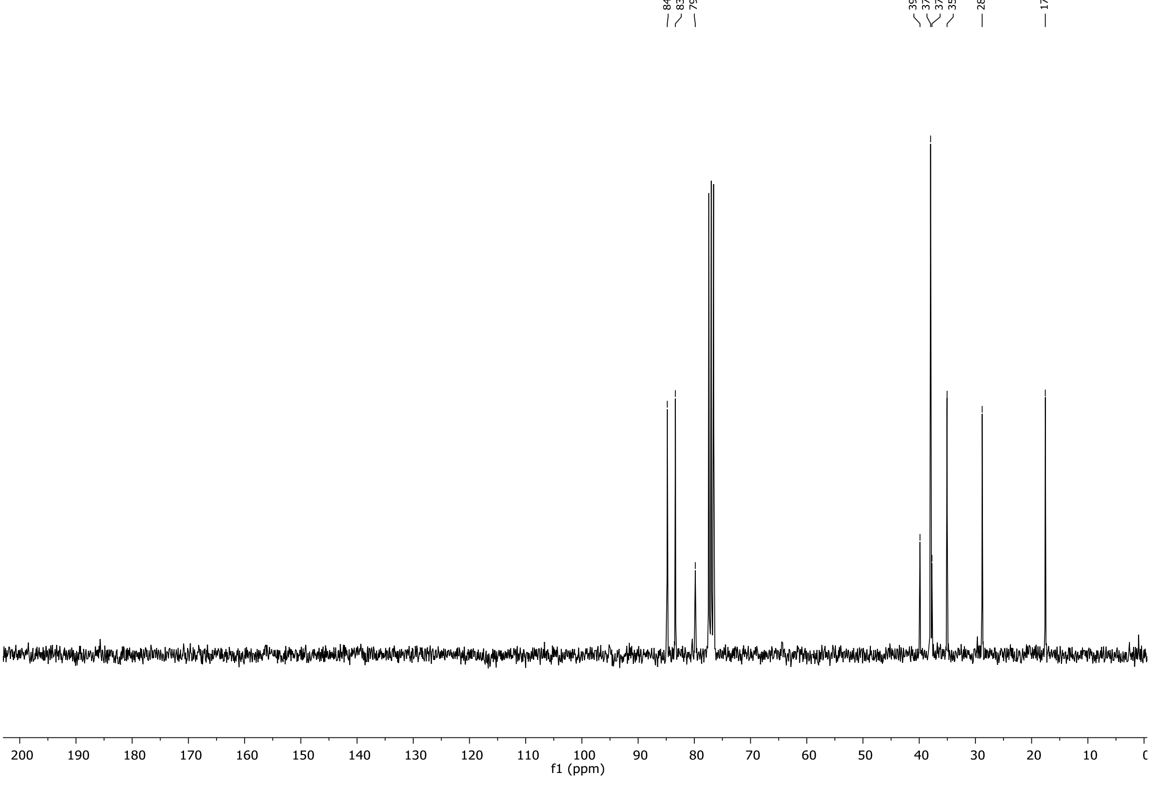

3-(but-3-yn-1-yl)tetrahydrofuran (41-CCH)

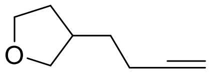

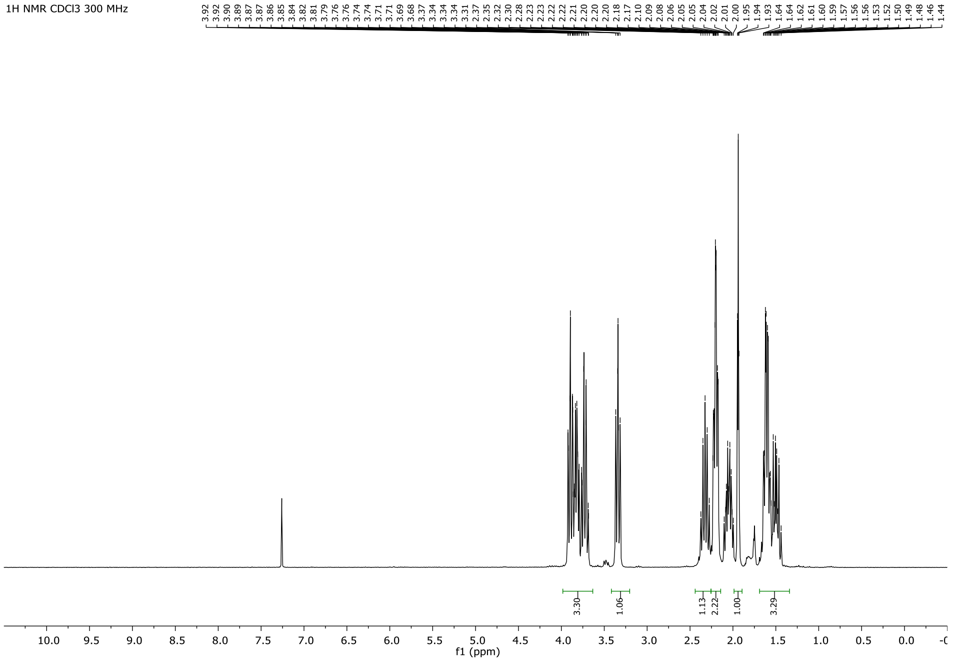

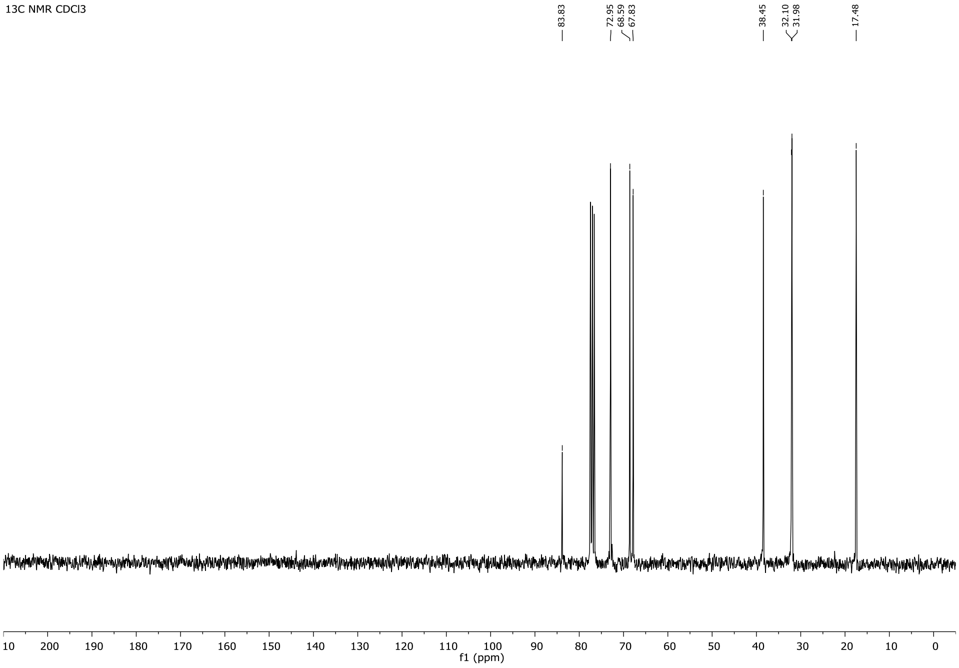

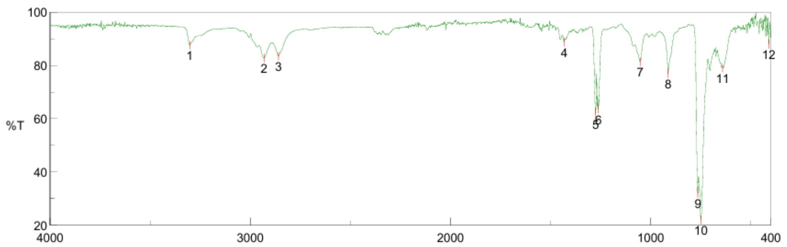

3-(4-bromobut-3-yn-1-yl)tetrahydrofuran (41)

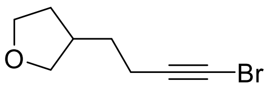

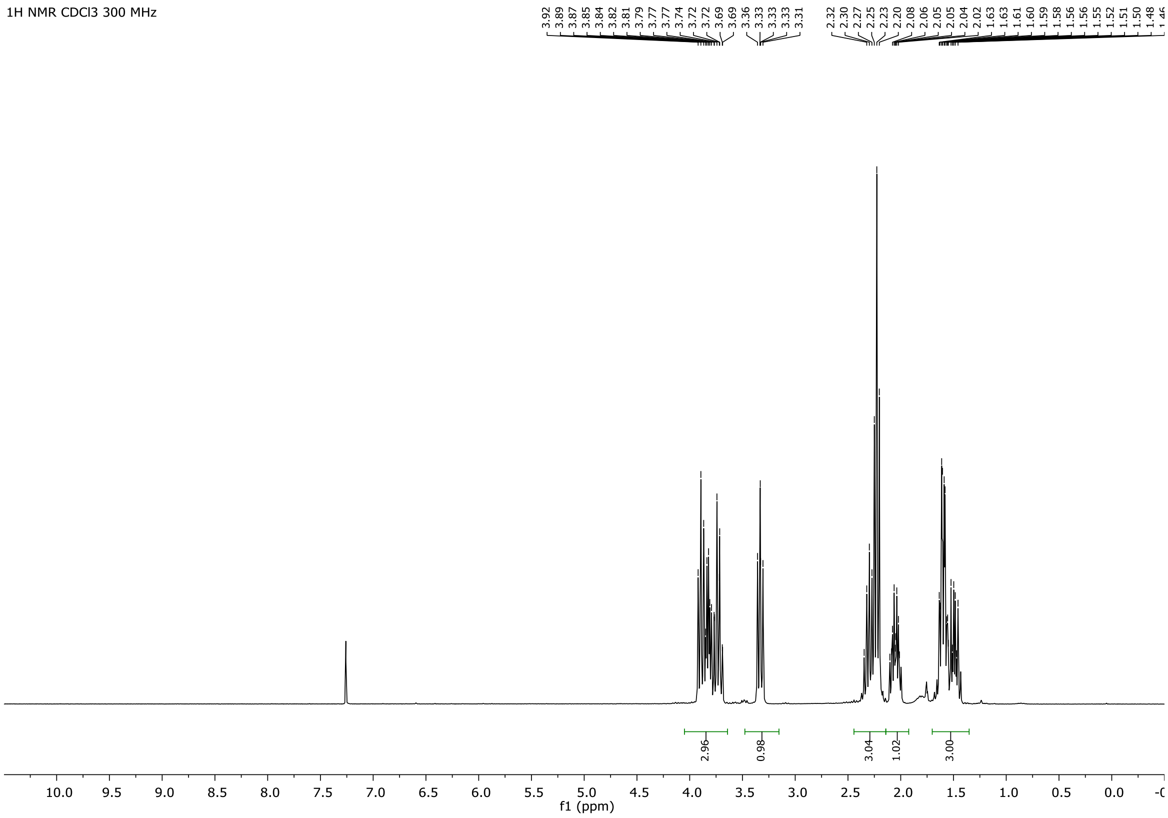

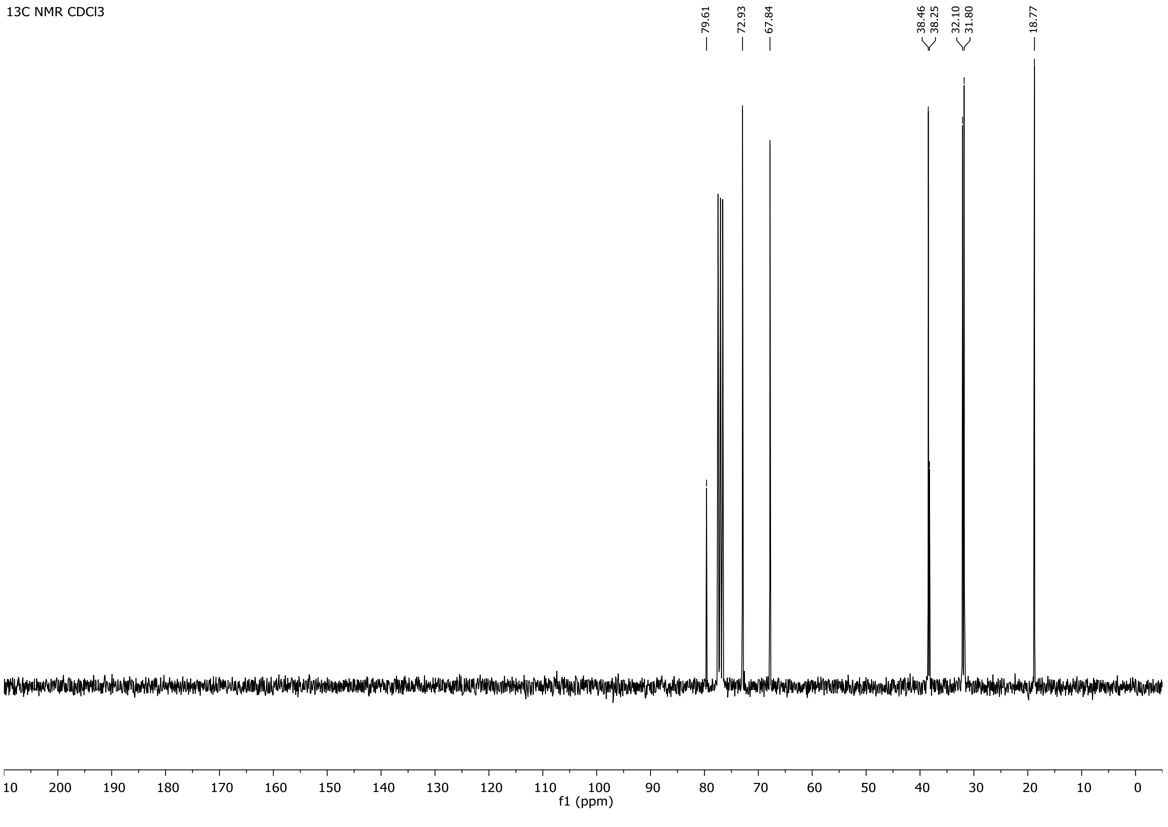

Supplement: Supplementary file 1 [file ol5c03125_si_001.pdf]
